# Iron-Mediated Photochemical Anti-Markovnikov Hydroazidation of Unactivated Olefins

**DOI:** 10.1021/jacs.3c09122

**Published:** 2023-10-09

**Authors:** Henry Lindner, Willi M. Amberg, Erick M. Carreira

**Affiliations:** Department of Chemistry and Applied Biosciences, Laboratory of Organic Chemistry, ETH Zürich, 8093 Zurich, Switzerland

## Abstract

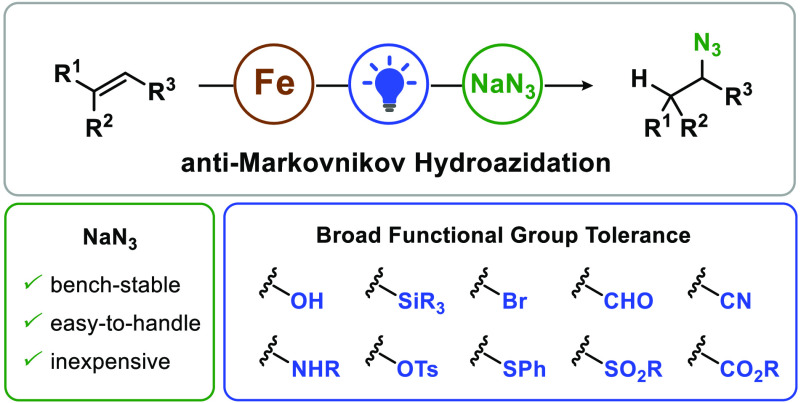

Unactivated olefins are converted to alkyl azides with bench-stable NaN_3_ in the presence of FeCl_3_·6H_2_O under blue-light irradiation. The products are obtained with anti-Markovnikov selectivity, and the reaction can be performed under mild ambient conditions in the presence of air and moisture. The transformation displays broad functional group tolerance, which renders it suitable for functionalization of complex molecules. Mechanistic investigations are conducted to provide insight into the hydroazidation reaction and reveal the role of water from the iron hydrate as the H atom source.

Organic azides are an integral part of an array of drug molecules, energetic materials, and chemical probes.^1^ They also are valuable building blocks in the synthesis of natural products, pharmaceuticals, and agrochemicals.^2^ As synthetic handles, azides have found widespread application in classic methods such as Staudinger reduction and ligation,^3^ Huisgen cycloaddition^4^ and click chemistry,^5^ as well as Schmidt^6^ and aza-Wittig reactions.^7^ More recently, azides have been utilized as nitrene precursors in transition-metal-catalyzed C–H bond aminations.^8^ Especially in the context of multistep synthesis, R–N_3_ can serve as a protected amine.^9^ Herein, we report the first iron- and light-mediated anti-Markovnikov hydroazidation of unactivated olefins (Figure 1). The transformation employs NaN_3_ as a bench-stable^10^ azide source, tolerates air and moisture, and proceeds under mild conditions allowing for a wide functional group compatibility.

**Figure 1:**
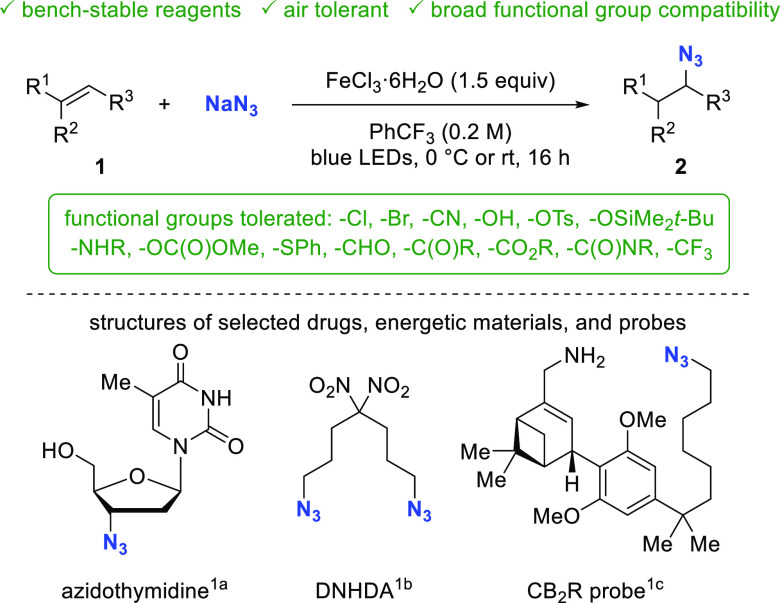
Iron-mediated photochemical anti-Markovnikov hydroazidation of unactivated olefins.

The widespread application of organic azides in synthetic chemistry and biology necessitates methodologies to access them directly from readily available starting materials. Traditionally, organic azides have been synthesized via nucleophilic substitution as well as diazo- and azido-transfer reactions.^11^ In a complementary approach, direct transformations of olefins to alkyl azides have been explored. Early studies by Hassner and Kropp focused on the addition of HN_3_ to alkenes, affording the corresponding Markovnikov products (Figure 2A).^12^

**Figure 2:**
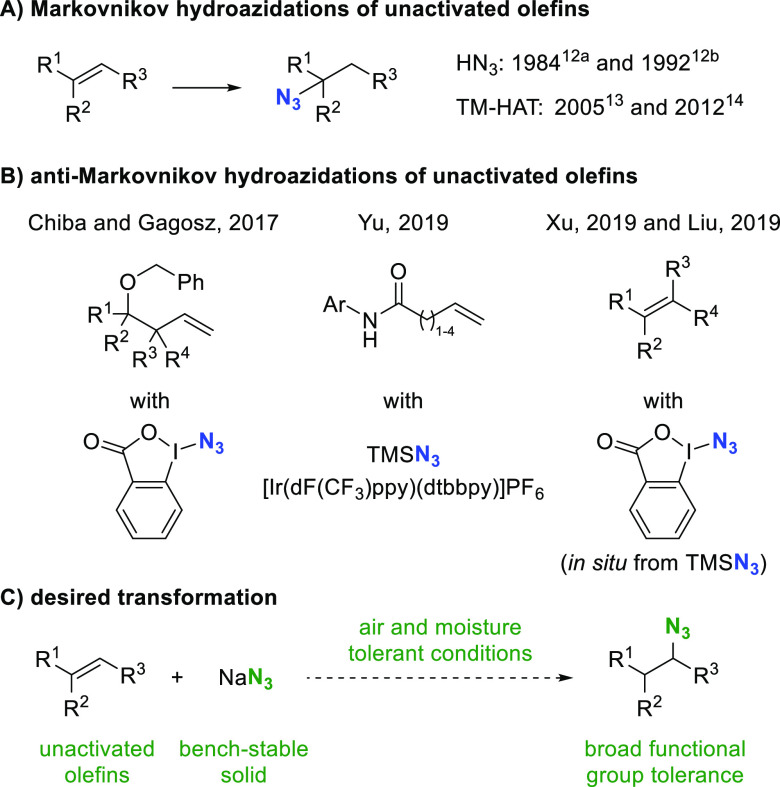
Approaches toward (A) Markovnikov and (B) anti-Markovnikov hydroazidation of unactivated olefins and (C) desired transformation.

Milder and more broadly applicable conditions for the Markovnikov hydroazidation of unactivated olefins were developed in our group which employ a cobalt catalyst, silane, and TsN_3_.^13^ Boger later disclosed a Markovnikov hydroazidation that is presumed to proceed via an iron hydride species.^14^ To obtain anti-Markovnikov addition products, multistep sequences were required. Only recently has the direct anti-Markovnikov azidation of double bonds been reported (Figure 2B). Chiba and Gagosz have investigated a hypervalent iodine reagent (azidobenziodoxolone, ABX) for the hydroazidation of homoallylic benzyl ethers with the latter serving as intramolecular H atom donor in the reaction.^15^ Yu and co-workers have documented a hydroazidation reaction of unsaturated aryl amides using an Ir photocatalyst and TMSN_3_.^16^ Most recently, Xu and Liu independently generated the active hypervalent iodine reagent ABX *in situ* from TMSN_3_ and a benziodoxolone to achieve anti-Markovnikov hydroazidation.^17^ Despite these important advances, convenient procedures using NaN_3_ as an off-the-shelf, inexpensive azide source are lacking.

Our interest in metal-mediated olefin hydroazidation reactions and, more recently, alkene functionalization and photochemical methods has led us to examine approaches to alkyl azides.^13,18^ Applications of transition-metal salts (e.g., Cu^II^, Ti^IV^, and Fe^III^) under visible-light irradiation caught our attention.^19^ The excitation of these salts with visible light induces ligand-to-metal charge transfer (LMCT), resulting in, for example, dichlorination, diazidation, or Giese reaction.^19c−19g,20^ We focused on the most abundant transition metal and hypothesized that the use of radicals generated from an iron complex under blue-light irradiation may be suitable to effect hydroazidation in a mild and selective manner. To this end, 4-phenylbutene (**1a**) was subjected to NaN_3_ (3.0 equiv) and Fe(NO_3_)_3_·9H_2_O (1.0 equiv) in dichloromethane (0.2 M) and irradiated (λ_max_ = 446 nm, 350 W blue LED photoreactor; for technical details see the Supporting Information) for 16 h at room temperature. In this experiment, primary azide **2a** was observed in 36% yield (Table 1, entry 1). Further studies revealed that FeCl_3_·6H_2_O in CH_2_Cl_2_ afforded the highest yield among all investigated iron salts (for details see the Supporting Information). As azides are known to displace the chlorides in CH_2_Cl_2_, subsequently generating highly explosive intermediates,^21^ solvent alternatives were investigated. The use of polar solvents such as acetone, ethyl acetate, and MeCN did not yield any hydroazidation, instead giving the diazide in a range of yields (for details see the Supporting Information).^19c,19d^ In contrast, employing less polar solvents such as haloarenes yielded monoazide in a good yield. Of the alternatives investigated, α,α,α-trifluorotoluene (PhCF_3_) performed best. Under optimized conditions (1.5 equiv of FeCl_3_·6H_2_O, 3.0 equiv of NaN_3_, PhCF_3_ (0.2 M), 0 °C for 16 h, blue LEDs, entry 2), **2a** was formed in 83% yield.

**Table 1:**
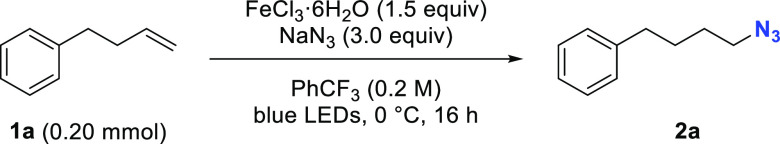
Optimization of the Reaction Conditions

entry	deviation from standard conditions	**2a**^a^ (%)
1	Fe(NO_3_)_3_·9H_2_O (1.0 equiv), CH_2_Cl_2_, rt	36
2	none	83
3	TMSN_3_ instead of NaN_3_	17
4	no iron salt	0
5	no light	0
6	FeCl_3_ instead of FeCl_3_·6H_2_O	<5
7	40 W blue LED Kessil light, rt	80

a Yield obtained by ^1^H NMR with mesitylene as internal standard.

Control experiments were performed to gain further insights into the transformation (for details including oxygen sensitivity, see the Supporting Information). When the reaction was conducted with TMSN_3_ under otherwise identical conditions, **2a** was produced in 17% yield (entry 3). The reaction did not provide the product in the absence of iron salt or light, and **1a** was fully recovered (entries 4 and 5). If anhydrous FeCl_3_ was used in oven-dried glassware under otherwise identical conditions, merely traces of the product were formed, indicating the necessity of water (entry 6). When using a 40 W blue LED Kessil light at 25% intensity, the best results were obtained at room temperature (entry 7; for details see the Supporting Information).

With the optimized conditions in hand, we set out to investigate the functional group tolerance of the hydroazidation reaction (Figure 3). To this end, a variety of alkyl azides were accessed in moderate to high yields from the corresponding alkenes.^22^ When vinyl silane **1b** was subjected to the reaction conditions, β-silyl azide **2b** was obtained in 80% yield. Alkyl azides **2c** and **2d** were isolated in yields of 73 and 80% from primary haloalkenes **1c** and **1d**. Notably, no substitution of either chloride or bromide was observed under the reaction conditions. 1-Dodecene yielded **2e** in 86% yield. A variety of electron-poor and -rich arenes were tolerated, furnishing **2f**–**2h** in 81–90% yield. Substrates containing protic groups, such as alcohol **1i**, tosyl amide **1j**, carbamate **1k**, and *N*-tosyl imide **1l**, were converted to the corresponding azides in 65–82% yield. Acid-labile *t*-BuMe_2_Si-protected alcohol **1m** afforded **2m** in 61% yield. Methyl carbonate **1n** and thioether **1o** were competent under reaction conditions, resulting in **2n** and **2o** in 85 and 43% yield, respectively. Azides **2p**–**2s** were accessed in 62–75% yield. Markedly, alkenes bearing a heterocycle, such as thiophene, furan, phthalimide, oxetane, and pyridine, were also well tolerated and gave rise to products **2t**–**2x** in 44–86% yield. The practical aspects of the method were demonstrated by the synthesis of **2a** on a larger scale (2.0 mmol) in 75% yield. All terminal olefins underwent anti-Markovnikov hydroazidation in excellent selectivity (rr =12:1 to >20:1; for details see the Supporting Information).

**Figure 3:**
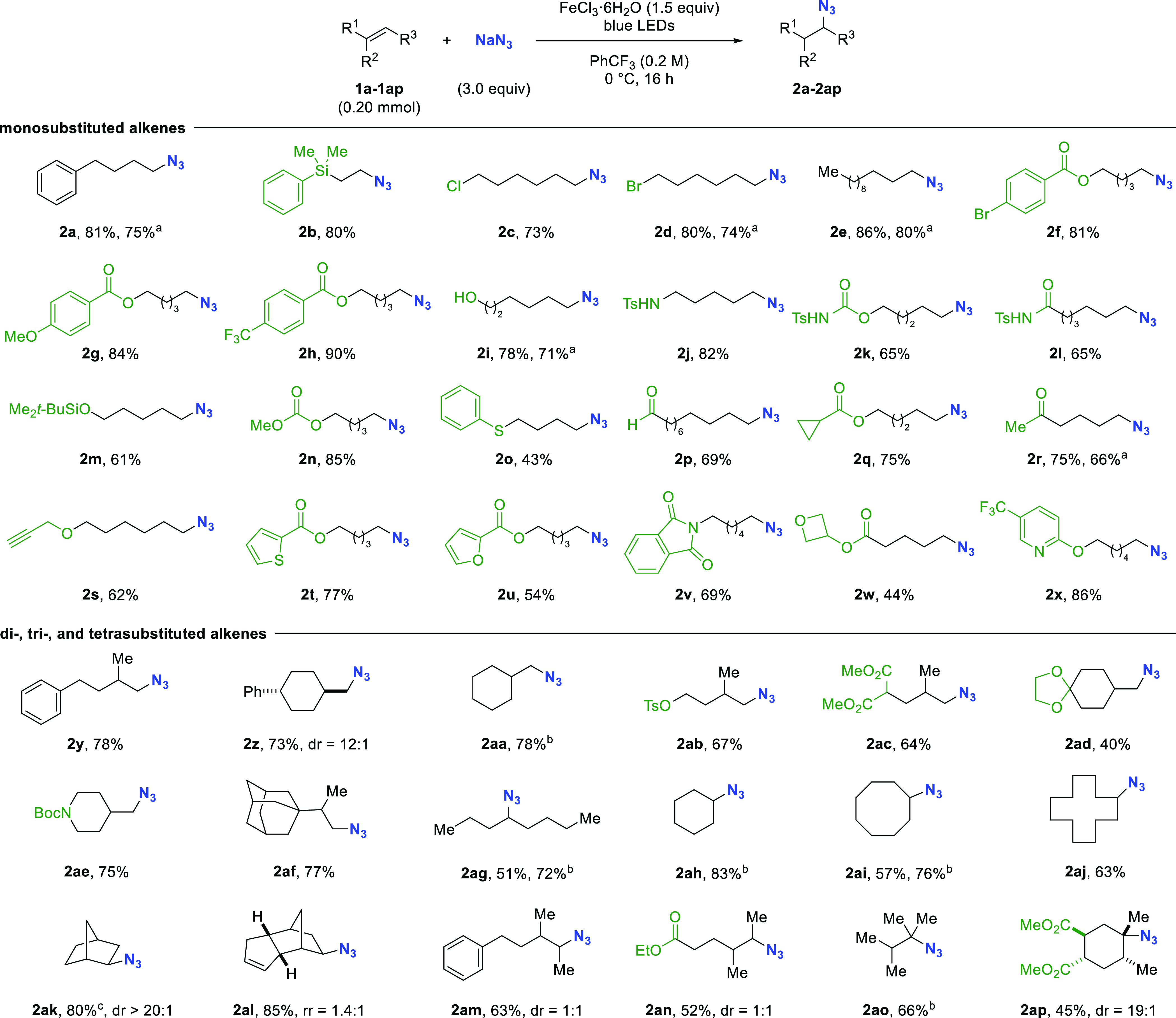
Substrate scope. Reaction conditions: olefin (0.20 mmol), NaN_3_ (0.60 mmol), FeCl_3_·6H_2_O (0.30 mmol) in PhCF_3_ (1.0 mL), irradiation in 350 W photoreactor at 0 °C for 16 h. dr determined by ^1^H NMR of the unpurified reaction mixture. ^*a*^Carried out on 2.0 mmol scale with a 40 W blue LED Kessil light at rt. ^*b*^Yield obtained by ^1^H NMR with mesitylene as internal standard. ^*c*^8 h reaction time.

Next, the effects of the alkene substitution patterns were examined. The reaction was amenable to di-, tri-, and tetrasubstituted olefins. 1,1-Disubstituted olefins **1y** to **1aa** afforded primary azides in 73–78% yield. Substrates bearing a tosylate, malonate, acetal, or carbamate were successfully converted to products **2ab**–**2ae** in 40–75% yield. β-Adamantyl azide **2af** was accessed in 77% yield. Acyclic and cyclic 1,2-disubstituted alkenes were subjected to the reaction conditions, furnishing the corresponding azides **2ag–2aj** in 51–83% yield. Strained olefins, in particular norbornene and dicyclopentadiene, were hydroazidated in high yield (80 and 85%, respectively).^23^ Trisubstituted olefins were well tolerated, giving rise to secondary azides with **2am** and **2an** isolated in 63 and 52% yield. All di- and trisubstituted olefins employed underwent anti-Markovnikov azidation in good regioselectivity (for details see the Supporting Information). Finally, tetrasubstituted alkenes **1ao** and **1ap** yielded **2ao** and **2ap** in 66 and 45% yield, respectively.

Encouraged by the broad functional group tolerance of the hydroazidation reaction, we set out to explore the generality of the protocol in a more complex setting. An array of alkenes derived from active pharmaceutical ingredients and natural products was subjected to the reaction conditions (Figure 4). To our delight, these substrates led to the formation of desired azides **4a**–**4u** in good yields (40–86%). In particular, molecules featuring diversely substituted arenes and heterocycles, such as oxazoles, indoles, thiazoles, β-lactams, and diazoles were well tolerated, indicating the potential of this method for late-stage application.

**Figure 4:**
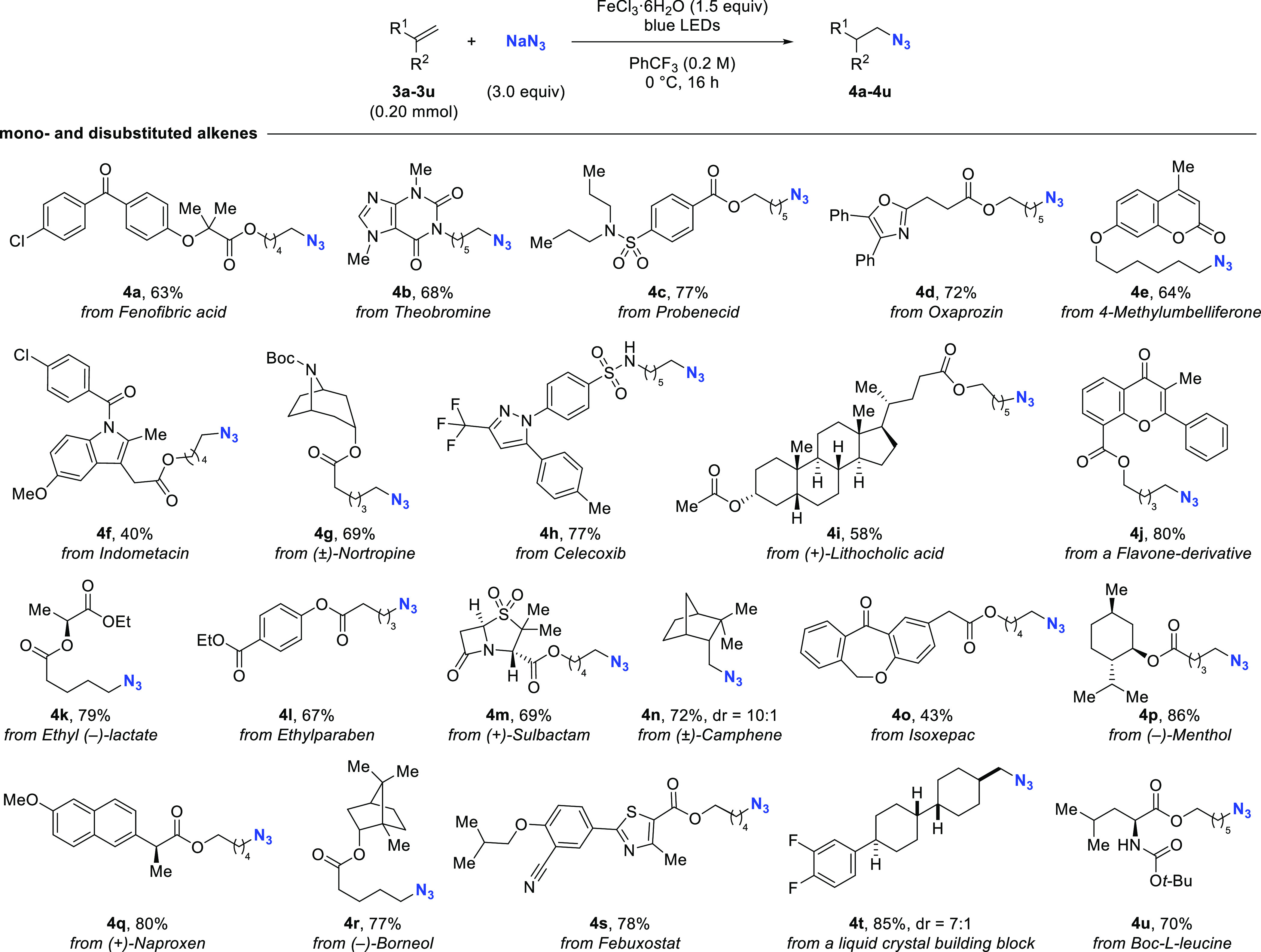
Substrate scope for olefins derived from natural products and drugs. dr determined by ^1^H NMR of the unpurified reaction mixture.

In order to deal with the volatility and inherent risk of low-molecular-weight azides, we examined several transformations subsequent to hydroazidation. One-pot procedures would avoid work-up, solvent evaporation, purification, isolation, and handling of the azide intermediates (Scheme 1). First, attempts to perform a Cu(I)-catalyzed azide–alkyne click reaction *in situ* were unsuccessful. We observed that the addition of NEt_3_ was crucial for the formation of triazole **7a** in 76% yield. When cyclooctyne was employed as a reaction partner, triazole **7b** was isolated in 54% yield from volatile azide **2ao**. Primary amine **7c** was accessed in 72% yield through Pd-catalyzed hydrogenation, and Staudinger reduction of cyclohexyl azide **2ah** with subsequent Boc-protection furnished the corresponding carbamate **7d** in 62% yield.

**Scheme 1:**
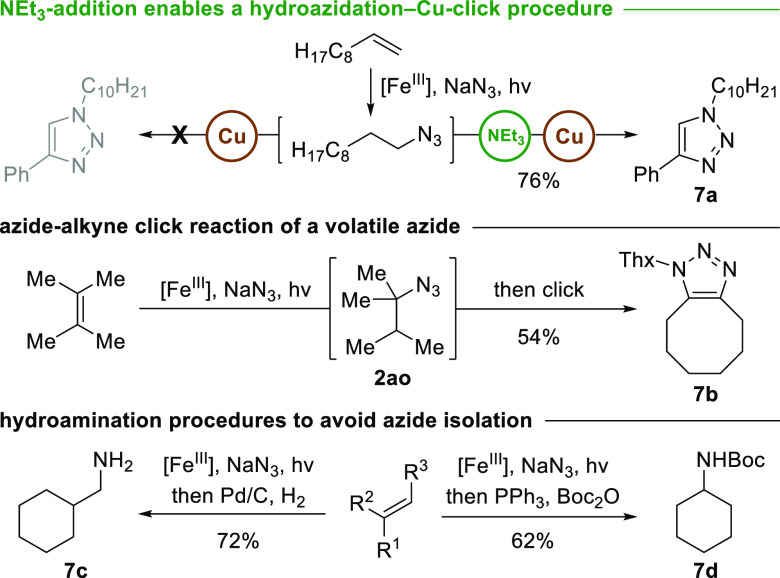
Sequential One-Pot Transformations

To gain mechanistic insights into the hydroazidation, a series of experiments was conducted (Figure 5). Initial investigations focused on determining whether radical species are involved in the reaction. The addition of 2.0 equiv TEMPO as radical scavenger^24^ under standard conditions suppressed the reaction, and no hydroazidation product could be detected by either ^1^H NMR or HRMS. Instead, alkene starting material was recovered.

**Figure 5:**
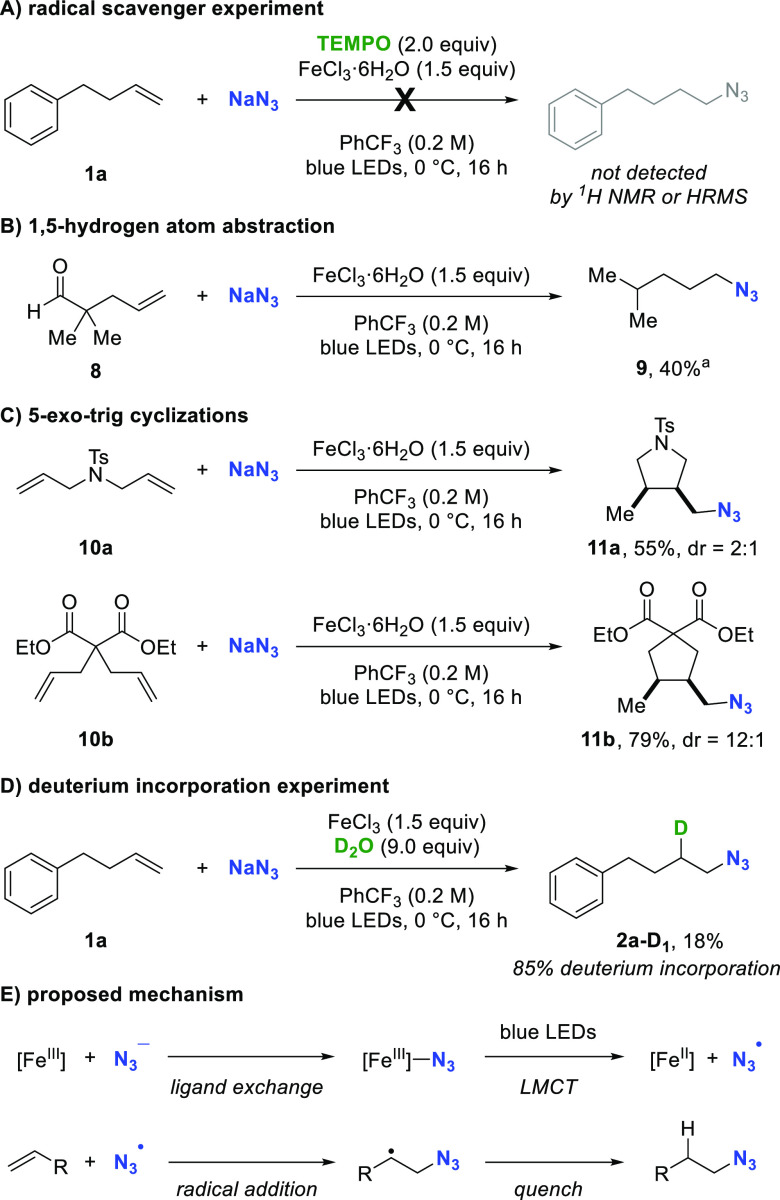
Mechanistic investigations and the proposal of a plausible mechanistic construct. dr determined by ^1^H NMR of the unpurified reaction mixture. ^*a*^Yield obtained by ^1^H NMR with mesitylene as internal standard.

In the context of our examination of the substrate scope, when aldehyde **8** was subjected to the standard reaction conditions, we observed 1-azido-4-methylpentane (**9**) as the sole product (Figure 5B). This is consistent with the formation of a secondary carbon-centered radical from the olefin followed by a 1,5-radical hydrogen atom abstraction from the aldehyde. Decarbonylation affords the more stable tertiary radical which is quenched.^25^

This result prompted us to examine substrates **10a** and **10b** which could undergo 5-exo-trig cyclization reactions (Figure 5C). *N*-Tosyl diallyl amine furnished pyrrolidine **11a** in 55% yield, and diethyl diallylmalonate delivered the corresponding cyclopentyl product **11b** in 79% yield. For both substrates, only cyclization products were isolated, suggesting that quenching is slower than cyclization for **10a** and **10b**.^26^

It has been proposed in the literature that under visible-light irradiation Fe(III) azides generate Fe(II) salts along with azidyl radicals.^19c,19d^ Based on these reports, in the system we describe the azidyl radical can then add (anti-Markovnikov) to the olefin to provide a reactive carbon-centered radical.^27^ This intermediate is ultimately quenched by an H atom donor. Accordingly, we set out to identify the origin of the hydrogen atom involved in the quenching.

It has previously been observed that hydrates of boron (Me_3_B·OH_2_) and titanium (Cp_2_ClTi·OH_2_) serve as H atom donors in radical reactions.^28^ Consequently, we hypothesized that the iron-bound water in FeCl_3_·6H_2_O might be involved as an H atom source. In an experiment in oven-dried glassware under a nitrogen atmosphere, D_2_O was added to anhydrous FeCl_3_ followed by addition of PhCF_3_/NaN_3_/4-phenylbutene (Figure 5D). After being stirred for 16 h under blue-light irradiation, azidodeuterated product **2a-D**_**1**_ was isolated in 18% yield. In parallel experiments using H_2_O under otherwise identical conditions, product **2a** was obtained in 77% yield. The difference in yield between the two experiments suggests a strong primary kinetic isotope effect (*k*_H_/*k*_D_ ≫ 1; for details see the Supporting Information). These data support water as the terminal H atom source and indicate that the H atom transfer to the secondary carbon-centered radical likely is the rate-limiting step of the transformation.^17a,29^ Although free water is not known to be an H atom source (*H*_BDE_(HO–H) = 118 kcal/mol), its coordination to iron dramatically decreases the bond-dissociation energy (*H*_BDE_(Fe^II^(H_2_O)_5_(HO–H)) = 77 kcal/mol).^30,31^ Our observation of solvent effects described in the optimization reactions suggests that the nature of the solvent affects speciation of Fe(III) complexes in the presence of azide and chloride counterions as well as water. Further mechanistic studies to understand the nature of Fe complexes formed, including μ_2_-bridged dimers, are clearly necessary as they may provide additional avenues for the development of new transformations.^32^

In conclusion, we have developed a photochemical anti-Markovnikov hydroazidation of unactivated alkenes with FeCl_3_·6H_2_O. The transformation shows broad functional group tolerance and was amenable to terminal as well as highly substituted olefins. Salient features of the reaction are its tolerance to air and moisture and the successful use of NaN_3_ as a bench-stable, low-cost, and easy-to-handle azide source. We demonstrated that the simplicity and generality of this method make it ideally positioned for late-stage applications, allowing for the efficient and versatile synthesis of diverse organic azides featuring biologically active motifs.

## Supporting Information Available

Experimental procedures and characterization data (PDF)
