# Supplementary material for: Iron-Mediated Photochemical Anti-Markovnikov Hydroazidation of Unactivated Olefins
Source: J Am Chem Soc. 2023 Oct 9;145(41):22347–53. doi: 10.1021/jacs.3c09122 (PMC10591317; doi:10.1021/jacs.3c09122)
Supplement: Supplementary file 1 — ja3c09122_si_001.pdf [file ja3c09122_si_001.pdf]

Supporting Information

Iron-Mediated Photochemical Anti-Markovnikov Hydroazidation of Unactivated Olefins

Henry Lindner[a], Willi M. Amberg[a], Erick M. Carreira[a*]

[a]ETH Zürich, Department of Chemistry and Applied Biosciences, Laboratory of Organic Chemistry, 8093 Zurich, Switzerland

Table of Contents

1. General Remarks 2
2. Photoreaction Set-up 5
3. Optimization Studies 7
4. Radical Scavenger Experiments 11
5. Deuterium Incorporation Experiments 13
6. 5-exo-trig Cyclizations 16
7. 1,5-Hydrogen Atom Abstraction Experiment 18
8. General Procedures for the Hydroazidation 20
9. Substrate Scope 22
10. Procedures for One-pot Transformations 85
11. Preparation of Starting Materials 91
12. ^{1}H, ^{13}C, and ^{19}F NMR Spectra 113
13. X-Ray Crystallographic Data 210
14. References 212

1. General Remarks

Procedure

Unless otherwise stated, all reactions were carried out under air. Reactions in the 350 W photoreactor were performed in 13x40 mm screw-thread vials (ROFRA GmbH, Mat. Nr. 14.020.92) that were charged with a magnetic stirrer bar (PTFE, 3x8 mm, Semadeni Plastics Group, Art. 244) and sealed with a screwcap. Reactions at 2.00 mmol scale were conducted in 27x37 mm screw-thread vials (infochroma ag, G075R-27/037-H) that were charged with a magnetic stirrer bar (PTFE, 6x15 mm, Semadeni Plastics Group, Art. 250) and sealed with a screwcap.

Unless otherwise stated, reagents and solvents were purchased from commercial suppliers (ABCR, Acros, Sigma Aldrich, Fluka, TCI, Strem, Alfa, Combi-Blocks or Fluorochem) and used as received. For flash column chromatography Sigma-Aldrich silica gel sorbent (high purity grade (9385), 230-400 mesh particle size, pore size 60) was used as a stationary phase.

Thin-Layer Chromatography

Analytical thin layer chromatography (TLC) was performed on glass plates from Supelco® (TLC silica gel 60 F_{254}: 25 glass plates, 20 x 20 cm) and visualized via exposure to ultraviolet light (254 nm or 365 nm) or TLC stain (aqueous potassium permanganate solution followed by heating or aqueous ceric ammonium molybdate solution followed by heating). Organic azides could be visualized via PPh_3-mediated reduction followed by staining with ninhydrin.[1]

Nuclear Magnetic Resonance Spectroscopy

All NMR spectra were measured in deuterated solvents at room temperature with a Bruker Avance 400 (400 MHz, equipped with 9.4 T magnet and BBFO probe), Bruker Ascend 400 (400 MHz, equipped with 9.4 T magnet and BBFO probe), Bruker Ultrashield 400 (400 MHz, equipped with 9.4 T magnet and BBFO probe) or Oxford 400 (400 MHz, equipped with 9.4 T magnet and BBFO probe). Chemical shifts are

referenced to the solvent residual signal ($CDCl_3$, ^{1}H: δ = 7.26 ppm, ^{13}C: δ = 77.16 ppm) and reported in parts per million (ppm). The following abbreviations are used in reporting NMR data: s = singlet, d = doublet, t = triplet, q = quartet, b = broad, dd = doublet of doublets, m = multiplet, etc.

High-Resolution Mass Spectrometry

High resolution mass spectrometric data were obtained by the mass spectrometry service of the Laboratory of Organic Chemistry at ETH Zurich on a Bruker Daltonics maXis ESI-QTOF or a Bruker Daltonics maXis II ESI-QTOF or a Thermo Q-Exactive GC Orbitrap instrument and are reported as (*m/z*).

IR Spectroscopy

Infrared spectra were recorded on a Perkin Elmer Two FT-IR spectrometer as thin films. Absorptions are reported as absorption maxima in wavenumbers (cm^{-1}).

X-Ray Crystallographic Analysis

The X-Ray diffraction was measured on a Rigaku Oxford Diffraction XtaLAB Synergy-S Dualflex kappa diffractometer equipped with a Dectris Pilatus 300 HPAD detector and using microfocus sealed tube Cu-Kα radiation with mirror optics (λ = 1.54178 Å). All measurements were carried out at 100K using an Oxford Cryosystems Cryostream 800 sample cryostat. Data collected on the Rigaku instrument were integrated using CrysAlisPro and corrected for absorption effects using a combination of empirical (ABSPACK) and numerical corrections. The structures were solved using SHELXT and refined by full-matrix least-squares analysis (SHELXL) using the program package OLEX2. All non-hydrogen atoms were refined anisotropically and hydrogen atoms were constrained to ideal geometries and refined with fixed isotropic displacement parameters (in terms of a riding model). The data was measured and analyzed by Dr. Michael Wörle, Dr. Nils Trapp, and Michael Solar (all Small Molecule Crystallography Center, ETH Zurich).

Optical Rotation

Optical rotations were measured on a Jasco P-2000 Polarimeter (dimensions of the cuvette: 10 cm, 1.5 mL).

Safety Statement

Sodium azide is highly toxic. A strip of paper impregnated with ferric chloride can be used to detect HN_3 (paper turns dark in presence of hydrazoic acid). The use of chlorinated alkyl solvents is highly discouraged for transformations involving inorganic azides due to the potential formation of diazidomethane.[2]

Low-molecular-weight organic azides are known to be potentially explosive compounds.[3] Azides with C/N ratio >3 are stable enough when handled with proper safety measures. Azides with a 1< C/N ratio <3 can be synthesized and isolated but should be stored below room temperature. Organic azides with C/N less than 1 should not be isolated.

All hydroazidation reactions on larger scale (2.0 mmol) were performed behind a blast shield. All isolated organic azides were stored in a freezer under exclusion of heat, light, pressure, and shock. While we did not encounter any issues during the entirety of the project, proper precautions were always taken.

2. Photoreaction Set-up

Reactions under blue-light irradiation were carried out in a custom-designed photoreactor.[4] It features ten circularly arranged 35 W blue LEDs, mounted on copper heat sinks, which surround the central reaction vessel holder (see left picture). The photoreactor is water cooled and fresh water (T < 15 °C) is continuously supplied to the reactor.

If only air cooling was used (four small fans, see right picture), the hydroazidation reactions heated up to 40°C internal temperature. For reactions at lower temperatures, a custom copper vessel holder was designed (see left picture) to constantly provide a more effective cooling. The cooper vessel holder is cooled with EtOH as medium and connected to a cryostat set to the appropriate temperature to achieve the desired internal temperatures.

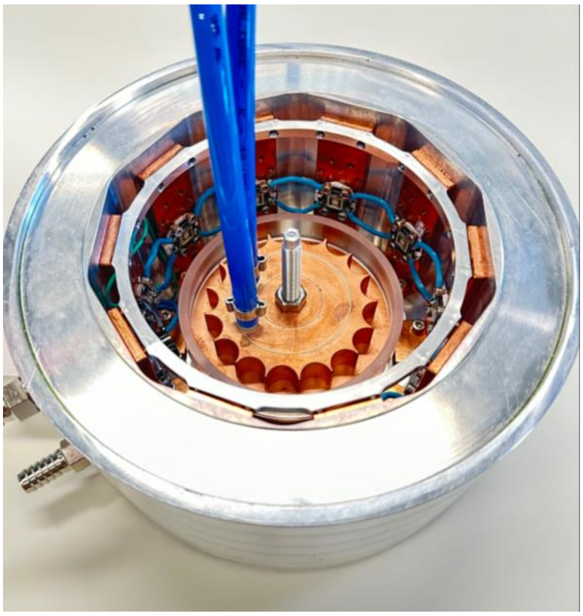
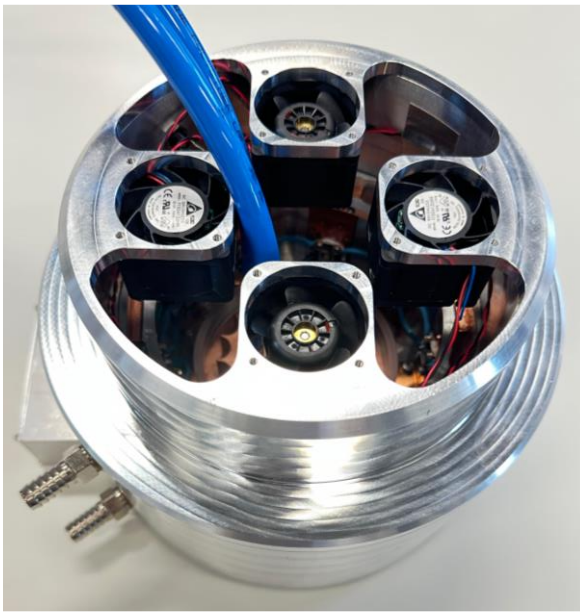

The emission spectrum (see below) of the blue LED reactor shows a maximum intensity at a wavelength of λ_{max} = 446 nm.[5]

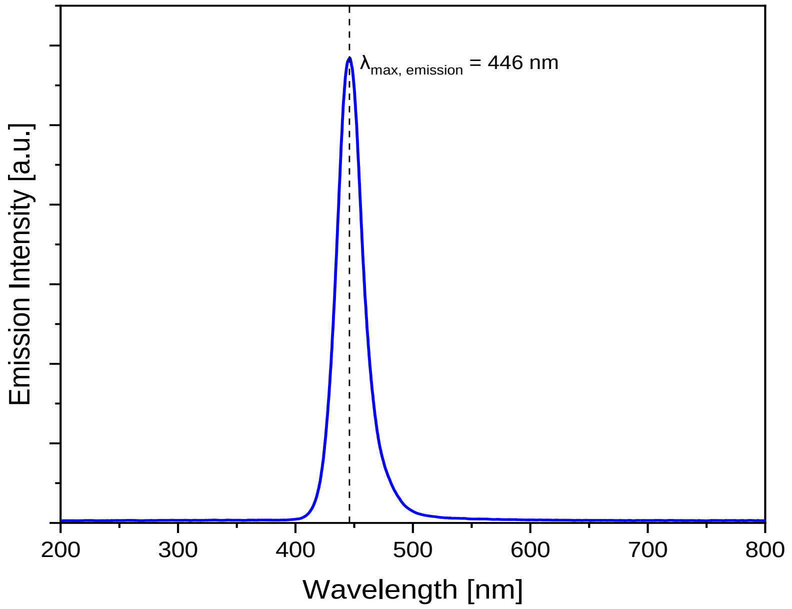

Note: The hydroazidation reaction on 0.200 mmol scale could also be conducted in comparable yield (80%) with a 40 W blue LED Kessil PR160L-440nm light (intensity set to 25%) placed 10 cm from the reaction vessel. For reactions on 2.00 mmol scale, the Kessil light (intensity set to 100%, see picture below for set-up) was placed 10 cm away from the reaction vessel. In both cases, air cooling was provided with a fan (15 cm diameter).

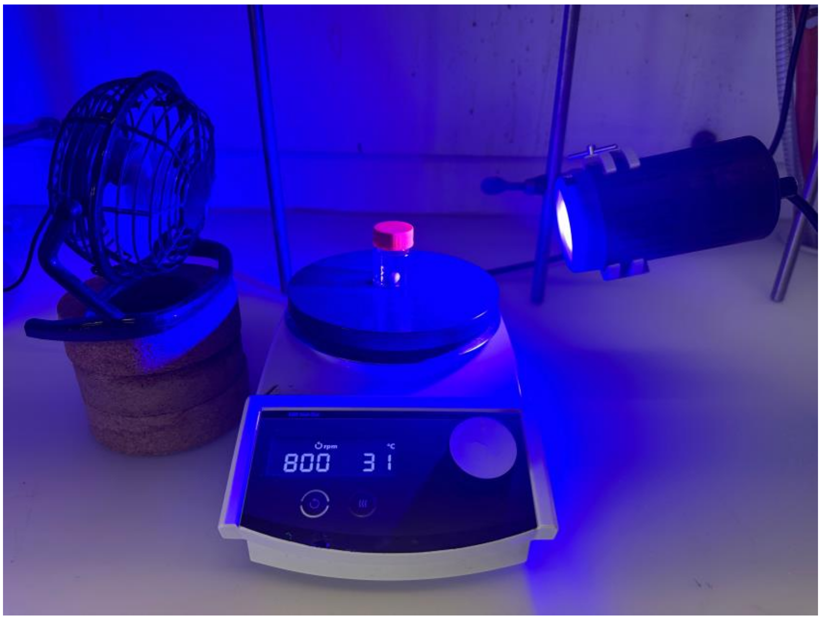

3. Optimization Studies

Example of procedure for optimization

To a glass vial charged with $FeCl_3 \cdot 6H_2O$ (81.0 mg, 0.300 mmol, 1.50 equiv) in $PhCF_3$ (1.00 mL, 0.200 M) were added 4-phenylbutene **1a** (30.0 µL, 26.4 mg, 0.200 mmol, 1.00 equiv), and NaN_3 (39.0 mg, 0.600 mmol, 3.00 equiv). The vial was equipped with a magnetic stirrer bar and capped with a screwcap. The reaction was stirred for 30 min at room temperature and irradiated in a 350 W photoreactor (*vide supra*) at 0 °C for 16 h. After addition of 27.8 µL mesitylene (24.0 mg, 0.200 mmol, 1.00 equiv) as internal standard, the reaction mixture was diluted with 1 mL $CDCl_3$ and filtered over Celite which was rinsed with additional $CDCl_3$. The reaction was analyzed by taking a 0.6 mL aliquot of this solution for ^{1}H NMR measurement.

Initial Experiments

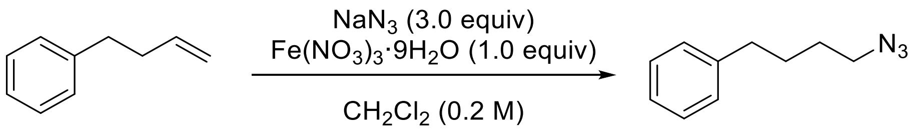

1a 2a

Entry	Change	1a [%]	2a [%]
1	none	44	36
2	$FeCl_3 \cdot 6H_2O$	38	48
3	$FeBr_3$	54	0
4	$Fe_2(SO_4)_3$	96	0
5	$Fe(ClO_4)_3 \cdot xH_2O$	85	8
6	$Fe_2(ox)_3$	94	0
7	$Fe(acac)_3$	93	0
8	0 °C	76	11
9	0 °C, $FeCl_3 \cdot 6H_2O$	6	75

Control Experiments

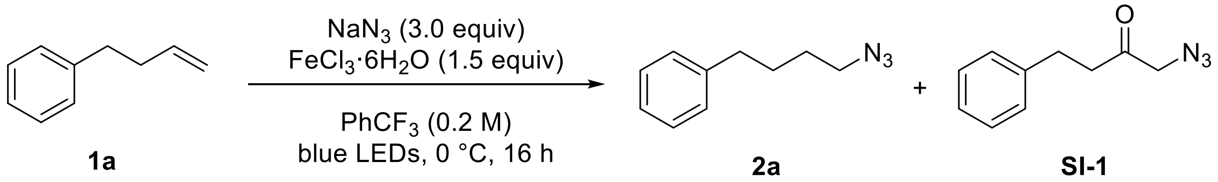

Entry	Change	1a [%]	2a [%]	SI-1 [%]
1	none	0	83	traces
2	no iron salt	95	0	0
3	in the dark, 0°C	93	0	0
4	in the dark, 40°C	76	0	0
5	degassed, under N_2	0	82	0
6	under O_2	12	52	8
7	40 W blue LED Kessil light (25%)	0	80	traces

Effect of Metal Salt

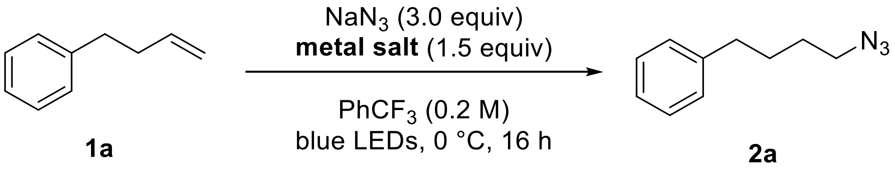

Entry	Iron Salt	1a [%]	2a [%]
1	$FeCl_3 \cdot 6H_2O$	0	83
2	$Fe(NO_3)_3 \cdot 9H_2O$	66	9
3	FeF_3	89	0
4	$FeCl_3$	72	traces
5	$FeBr_3$	0	traces
6	$Fe_2(ox)_3$	96	0
7	$Fe(acac)_3$	97	0
8	$Fe_2(SO_4)_3 \cdot xH_2O$	90	0
9	$(NH_4)Fe(SO_4)_2 \cdot xH_2O$	69	traces

10	$FeCl_2·4H_2O$	92	0
11	$FeSO_4·7H_2O$	94	0
12	Fe(citrate)	88	0
13	$CuCl_2·2H_2O$	98	0
14	$CuBr_2$	98	0
15	$CuSO_4·5H_2O$	97	0
16	$CoCl_2$	97	0
17	$NiCl_2$	95	0

Effect of Azide

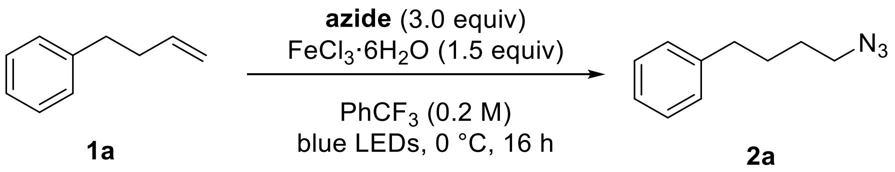

Entry	Azide	1a [%]	2a [%]
1	NaN_3	0	83
2	KN_3	21	61
3	$TMSN_3$	26	17
4	$(n\text{-Bu})_4N_3$	91	traces

Effect of Concentration

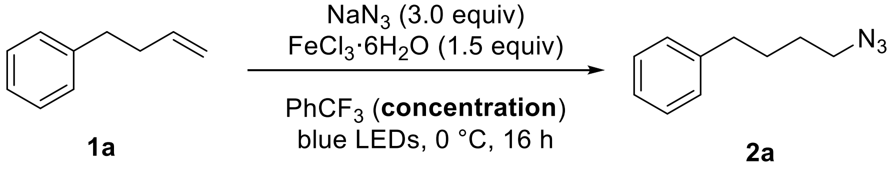

Entry	Concentration [M]	1a [%]	2a [%]
1	0.2	0	83
2	0.4	0	84
3	0.1	40	46

Effect of Solvent

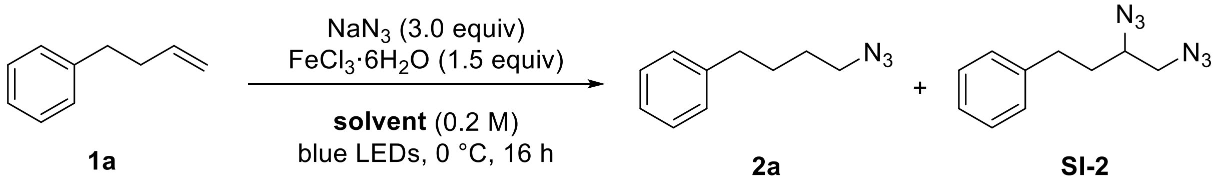

Entry	Solvent	1a [%]	2a [%]	SI-2 [%]
1	$PhCF_3$	0	83	0
2	hexane	78	5	0
3	CH_2Cl_2	0	83	0
4	DCE	0	83	0
5	$CHCl_3$	0	75	0
6	PhH	96	0	0
7	PhMe	90	0	0
8	PhF	0	81	0
9	1,4-difluorobenzene	6	54	0
10	1,3-bis(trifluoromethyl)benzene	15	66	0
11	PhCl	0	80	0
12	1,2-dichlorobenzene	0	74	0
13	1,2,4-trichlorobenzene	75	16	0
14	PhOMe	12	57	0
15	Et_2O	99	0	0
16	TFH	98	0	0
17	$MeNO_2$	36	18	traces
18	acetone	72	0	traces
19	PhCN	31	16	traces
20	MeCN	54	0	4
21	ethyl acetate	66	0	8
22	MeOH	81	0	0
23	DMF	84	0	0

4. Radical Scavenger Experiments

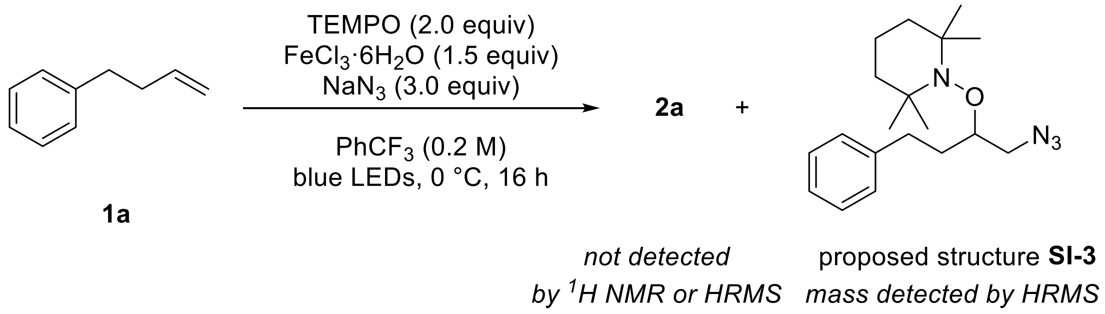

To a glass vial charged with $FeCl_3·6H_2O$ (81.0 mg, 0.300 mmol, 1.50 equiv) in $PhCF_3$ (1.00 mL, 0.200 M) were added 4-phenylbutene **1a** (30.0 µL, 26.4 mg, 0.200 mmol, 1.00 equiv), NaN_3 (39.0 mg, 0.600 mmol, 3.00 equiv), and 2,2,6,6-tetramethylpiperidinyloxyl (TEMPO, 62.5 mg, 0.400 mmol, 2.00 equiv). The vial was equipped with a magnetic stirrer bar and capped with a screwcap. The reaction was stirred for 30 min at room temperature and irradiated in a 350 W photoreactor (*vide supra*) at 0 °C for 16 h. After addition of 27.8 µL mesitylene (24.0 mg, 0.200 mmol, 1.00 equiv) as internal standard, the reaction mixture was diluted with 1 mL $CDCl_3$ and filtered over Celite which was rinsed with additional $CDCl_3$. The reaction was analyzed by taking a 0.6 mL aliquot of the solution. No product was observed in the ^1H NMR of the unpurified reaction mixture or could be detected via HRMS. Instead, the high-resolution mass of proposed TEMPO adduct **SI-3** could be identified:

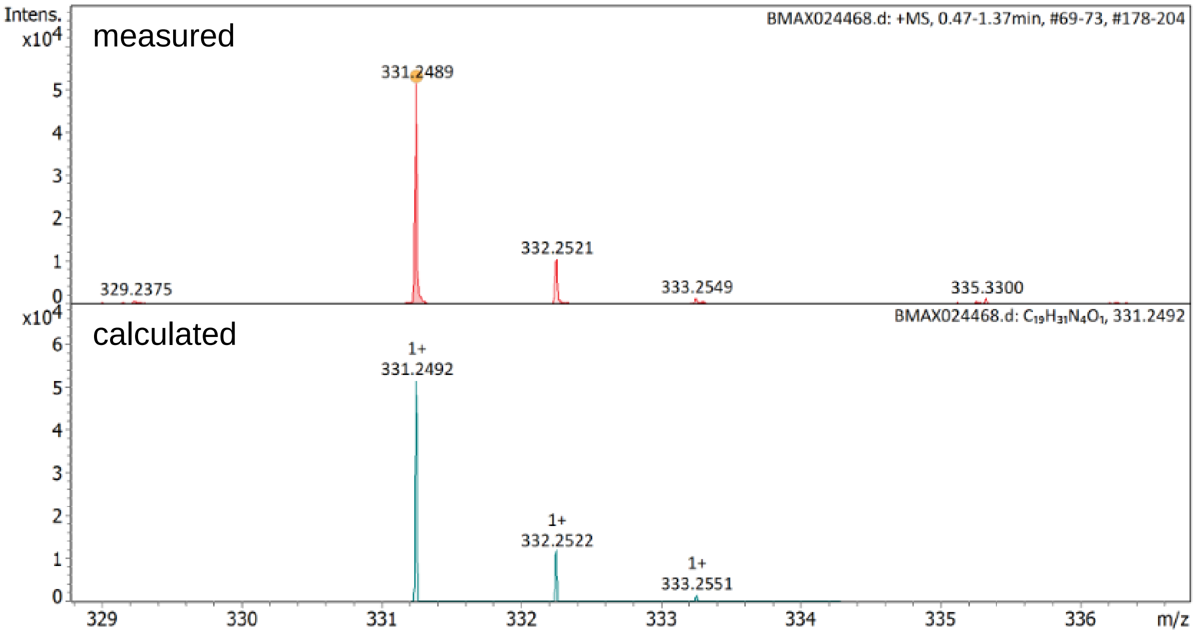

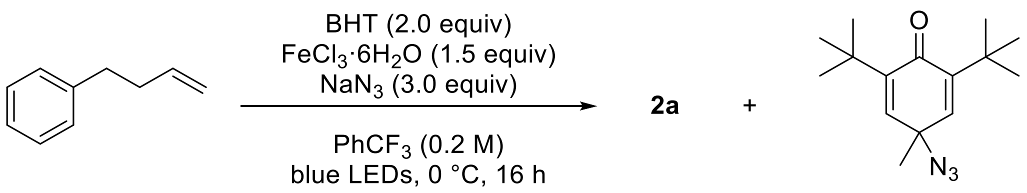

1a 2% *yield by* [1]*H NMR* proposed structure **SI-4** *mass detected by HRMS*

To a glass vial charged with $FeCl_3 \cdot 6H_2O$ (81.0 mg, 0.300 mmol, 1.50 equiv) in $PhCF_3$ (1.00 mL, 0.200 M) were added 4-phenylbutene **1a** (30.0 µL, 26.4 mg, 0.200 mmol, 1.00 equiv), NaN_3 (39.0 mg, 0.600 mmol, 3.00 equiv), and 2,6-di-tert-butyl-4-methylphenol (BHT, 88.1 mg, 0.400 mmol, 2.00 equiv). The vial was equipped with a magnetic stirrer bar and capped with a screwcap. The reaction was stirred for 30 min at room temperature and irradiated in a 350 W photoreactor (*vide supra*) at 0 °C for 16 h. After addition of 27.8 µL mesitylene (24.0 mg, 0.200 mmol, 1.00 equiv) as internal standard, the reaction mixture was diluted with 1 mL $CDCl_3$ and filtered over Celite which was rinsed with additional $CDCl_3$. The reaction was analyzed by taking a 0.6 mL aliquot of the solution. 2% of product **2a** were observed in the ^1H NMR of the unpurified reaction mixture. Furthermore, the high-resolution mass of proposed BHT adduct **SI-4** could be identified:

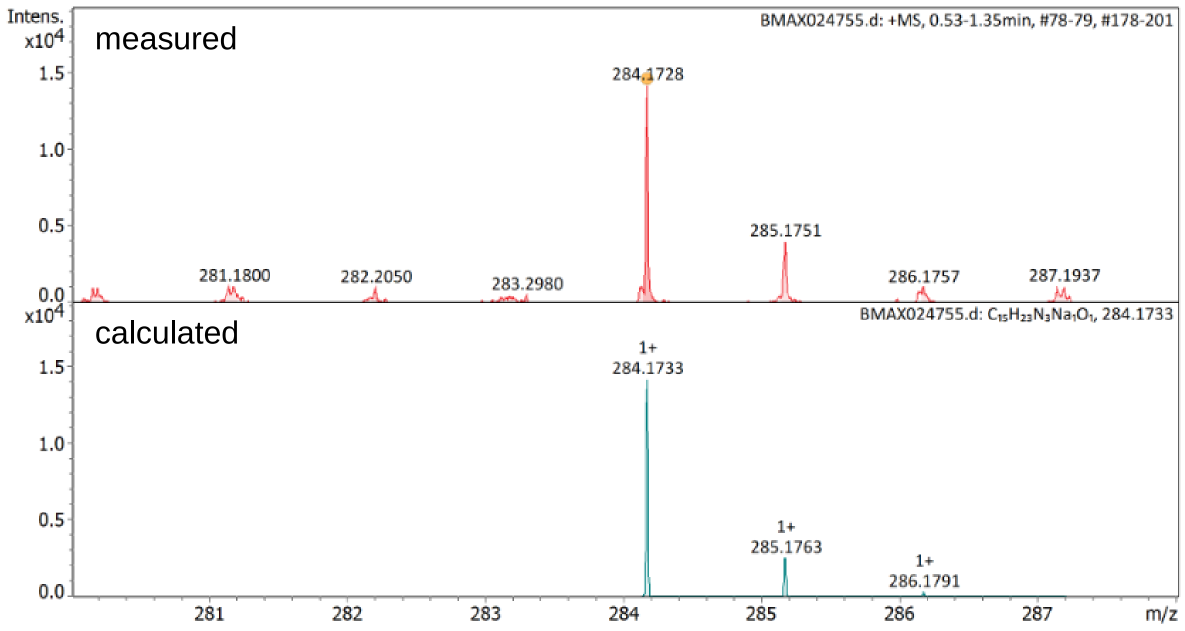

5. Deuterium Incorporation Experiments

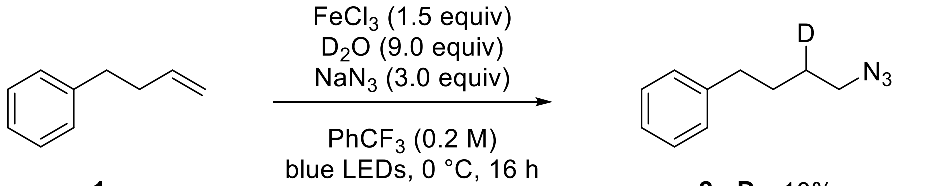

1a

2a-D$_1$, 18%
85% deuterium incorporation

In a nitrogen filled glovebox, to an oven-dried glass vial charged with $FeCl_3$ (48.7 mg, 0.300 mmol, 1.50 equiv) and D_2O (32.5 µL, 36.0 mg, 1.80 mmol, 9.00 equiv) in $PhCF_3$ (1.00 mL, 0.200 M) were added 4-phenylbutene **1a** (30.0 µL, 26.4 mg, 0.200 mmol, 1.00 equiv), and NaN_3 (39.0 mg, 0.600 mmol, 3.00 equiv). The vial was equipped with a magnetic stirrer bar, capped with a screwcap, and removed from the glovebox. The reaction was stirred for 30 min at room temperature and irradiated in a 350 W photoreactor (*vide supra*) at 0 °C for 16 h. The crude reaction mixture was filtered over Celite and rinsed with additional $PhCF_3$. The crude product was purified via flash column chromatography (0-2% EtOAc in hexane) to give **2a-D$_1$** as a colorless oil. The product was isolated as an inseparable 19:1 mixture of regioisomers.

Note: When, as a control, the reaction was conducted with H_2O instead of D_2O under otherwise identical conditions, product **2a** was isolated in 77% yield.

Yield: 6.3 mg, 0.036 mmol, 18%, rr = 19:1

^{1}H NMR (400 MHz, $CDCl_3$): δ (ppm) = 7.34 – 7.25 (m, 2H), 7.24 – 7.15 (m, 3H), 3.28 (dd, *J* = 6.8, 1.2 Hz, 2H), 2.69 – 2.62 (m, 2H), 1.77 – 1.67 (m, 2H), 1.67 – 1.59 (m, 1H).

^{13}C NMR (101 MHz, $CDCl_3$): δ (ppm) = 142.0, 128.5, 128.5, 126.1, 51.4, 35.5, 28.5, 28.22 (t, *J* = 19.6 Hz).

IR (thin film, cm^{-1}): 2933, 2862, 2094, 1496, 1454, 1273, 746, 699.

HRMS (EI): *m/z* for $C_{10}H_{11}DN$ $[M–HN_2]^+$: calc.: 147.1027, found: 147.1027.

TLC: R_f = 0.27 (SiO_2, 2% EtOAc in hexane).

Note: During the analysis of the ^{1}H NMR spectrum of deuterated product **2a-D$_1$**, a second inseparable species could be observed in traces (approx. 0.4% ^{1}H NMR yield). This side-product is hypothesized to be allyl azide **SI-5**.

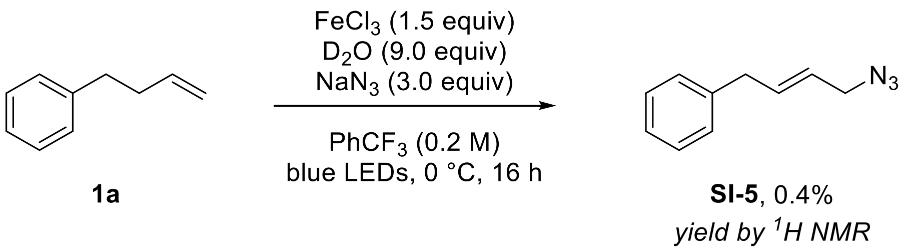

^{1}H NMR (400 MHz, $CDCl_3$): δ (ppm) = 7.34 – 7.25 (m, 2H), 7.24 – 7.15 (m, 3H), 6.02 – 5.87 (m, 1H), 5.66 – 5.54 (m, 1H), 3.75 (d, *J* = 6.6 Hz, 2H), 3.44 (d, *J* = 6.9 Hz, 2H).

To gain insight into the kinetic aspects of the deuterium incorporation by comparison of two reactions which are conducted separately, the reaction was set up as described above and removed from the photoreactor after 8 h, 16 h, and 32 h, respectively. After addition of 27.8 µL mesitylene (24.0 mg, 0.200 mmol, 1.00 equiv) as internal standard, the reaction mixture was diluted with 1 mL $CDCl_3$ and filtered over Celite which was rinsed with additional $CDCl_3$. The reaction was analyzed by taking a 0.6 mL aliquot of the solution. The ^{1}H NMR yields resulting from the experiments using D_2O and H_2O, respectively, are reported below:

**Time [h]**	**deuterium incorporation experiment with D_2O**		**control experiment with H_2O**	
	**2a-D$_1$ [%]**	**1a [%]**	**2a [%]**	**1a [%]**
8	12	64	75	9
16	20	55	82	0
32	37	39	83	0

A deuterium incorporation experiment which involves an intermolecular competition between a deuterated and a non-deuterated reagent (D_2O vs H_2O) in the same reaction flask was also conducted:

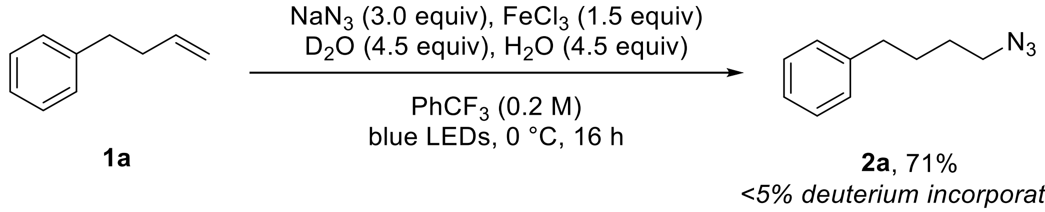

1a

2a, 71%
<5% deuterium incorporation

In a nitrogen filled glovebox, to an oven-dried glass vial charged with $FeCl_3$ (48.7 mg, 0.300 mmol, 1.50 equiv), D_2O (16.2 µL, 18.0 mg, 0.900 mmol, 4.50 equiv), and H_2O (16.3 µL, 16.2 mg, 0.900 mmol, 4.50 equiv) in $PhCF_3$ (1.00 mL, 0.200 M) were added 4-phenylbutene **1a** (30.0 µL, 26.4 mg, 0.200 mmol, 1.00 equiv), and NaN_3 (39.0 mg, 0.600 mmol, 3.00 equiv). The vial was equipped with a magnetic stirrer bar and capped with a screwcap. The vial was removed from the glovebox. The reaction was stirred for 30 min at room temperature and irradiated in a 350 W photoreactor (*vide supra*) at 0 °C for 16 h. After addition of 27.8 µL mesitylene (24.0 mg, 0.200 mmol, 1.00 equiv) as internal standard, the reaction mixture was diluted with 1 mL $CDCl_3$ and filtered over Celite which was rinsed with additional $CDCl_3$. The reaction was analyzed by taking a 0.6 mL aliquot of the solution. The ^1H NMR of the unpurified reaction mixture showed formation of **2a** in 71% yield but with <5% deuterium incorporation.

6. 5-exo-trig Cyclizations

Compound 11a:

(±)-(3R,4R)-3-(Azidomethyl)-4-methyl-1-tosylpyrrolidine

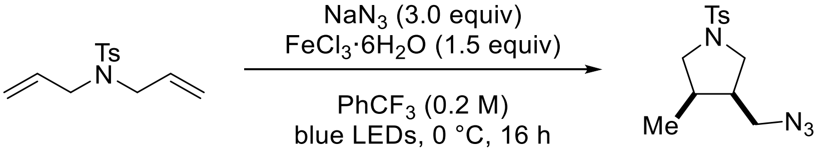

10a **11a**, 55% dr = 2:1

Azide **11a** was prepared via GP1 (*vide infra*) from alkene **10a** (50.3 mg, 0.200 mmol, 1.00 equiv) in $PhCF_3$. The crude product was purified via flash column chromatography (0-20% EtOAc in hexane) to give **11a** as a colorless oil. The product was isolated as an inseparable 2:1 mixture of diastereomers.

Yield: 32.2 mg, 0.109 mmol, 55%, dr = 2:1

^{1}H NMR (400 MHz, $CDCl_3$): δ (ppm) = 7.74 – 7.66 (m, 2H), 7.36 – 7.30 (m, 2H), 3.53 – 3.35 (m, 2H), 3.28 (ddd, *J* = 22.9, 12.3, 5.8 Hz, 1H), 3.17 – 3.08 (m, 1H), 3.03 (dd, *J* = 10.2, 7.3 Hz, $1H^{minor}$), 2.95 (ddd, *J* = 13.0, 7.1, 4.7 Hz, $2H^{major}$), 2.78 (dd, *J* = 9.8, 7.9 Hz, $1H^{minor}$), 2.43 (d, *J* = 1.8 Hz, 3H), 2.35 – 2.15 (m, $2H^{major}$), 1.94 – 1.76 (m, $2H^{minor}$), 0.95 (d, *J* = 6.4 Hz, $3H^{minor}$), 0.82 (d, *J* = 7.0 Hz, $3H^{major}$).

^{13}C NMR (101 MHz, $CDCl_3$): δ (ppm) = 143.7, 143.7, 133.8, 133.5, 129.8, 129.8, 127.7, 127.5, 54.7, 54.3, 52.6, 51.1, 50.0, 50.0, 45.4, 41.4, 36.3, 34.6, 21.6, 21.6, 16.8, 13.0.

IR (thin film, cm^{-1}): 2964, 2100, 1453, 1343, 1157, 1094, 1048, 817, 665, 590, 549.

HRMS (ESI+): *m/z* for $C_{13}H_{18}N_4NaO_2S$ $[M+Na]^+$: calc.: 317.1043, found: 317.1037.

TLC: R_f = 0.27 (SiO_2, 20% EtOAc in hexane).

Compound 11b:

(±)-Diethyl (3R,4S)-3-(azidomethyl)-4-methylcyclopentane-1,1-dicarboxylate

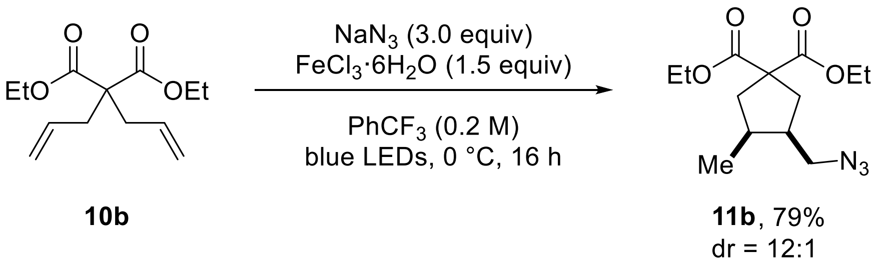

Azide **11b** was prepared via GP1 (*vide infra*) from alkene **10b** (45.4 µL, 48.6 mg, 0.200 mmol, 1.00 equiv) in $PhCF_3$. The crude product was purified via flash column chromatography (0-20% Et_2O in pentane) to give **11b** as a colorless oil. The product was isolated as an inseparable 12:1 mixture of diastereomers.

Yield: 44.9 mg, 0.158 mmol, 79%, dr = 12:1

^{1}H NMR (400 MHz, $CDCl_3$): δ (ppm) = 4.16 (qd, *J* = 7.1, 0.9 Hz, 4H), 3.29 (dd, *J* = 12.2, 6.8 Hz, 1H), 3.20 (dd, *J* = 12.3, 7.4 Hz, 1H), 2.41 (ddd, *J* = 18.5, 13.7, 7.2 Hz, 2H), 2.30 – 2.20 (m, 2H), 2.07 (dd, *J* = 13.6, 7.9 Hz, 1H), 1.94 (dd, *J* = 13.7, 6.3 Hz, 1H), 1.22 (t, *J* = 7.1 Hz, 6H), 0.89 (d, *J* = 6.9 Hz, 3H).

^{13}C NMR (101 MHz, $CDCl_3$): δ (ppm) = 172.6, 172.6, 61.6, 61.6, 59.0, 52.0, 42.0, 41.4, 36.8, 35.2, 14.8, 14.1.

IR (thin film, cm^{-1}): 2979, 2877, 2095, 1726, 1447, 1367, 1248, 1178, 1095, 859.

HRMS (ESI+): *m/z* for $C_{13}H_{21}N_3NaO_4$ $[M+Na]^+$: calc.: 306.1424, found: 306.1417.

TLC: R_f = 0.63 (SiO_2, 20% EtOAc in hexane).

7. 1,5-Hydrogen Atom Abstraction Experiment

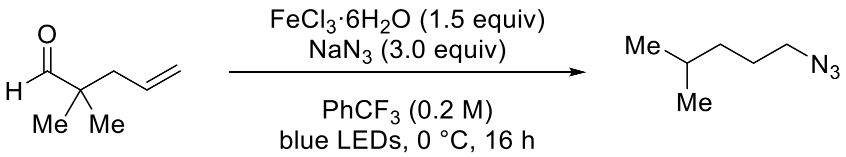

8 **9**, 40%

To a glass vial charged with $FeCl_3 \cdot 6H_2O$ (81.0 mg, 0.300 mmol, 1.50 equiv) in $PhCF_3$ (1.00 mL, 0.200 M) were added olefin **8** (27.2 µL, 22.4 mg, 0.200 mmol, 1.00 equiv), and NaN_3 (39.0 mg, 0.600 mmol, 3.00 equiv). The vial was equipped with a magnetic stirrer bar and capped with a screwcap. The reaction was stirred for 30 min at room temperature and irradiated in a 350 W photoreactor (*vide supra*) at 0 °C for 16 h. After addition of 27.8 µL mesitylene (24.0 mg, 0.200 mmol, 1.00 equiv) as internal standard, the reaction mixture was diluted with 1 mL $CDCl_3$ and filtered over Celite which was rinsed with additional $CDCl_3$. The reaction was analyzed by taking a 0.6 mL aliquot of the solution. 40% of product decarbonylated product **9** were observed in the ^1H NMR of the unpurified reaction mixture.

Note: To verify the structural assignment of the proposed decarbonylation product, a one-pot derivatization procedure was devised. After completion of the hydroazidation, cyclooctyne (43.3 mg, 0.400 mmol, 2.00 equiv) was added and the reaction was stirred for 2 h at room temperature. The reaction mixture was filtered over Celite, and the filtrate was concentrated *in vacuo*. The crude product was purified via flash column chromatography (0-20% EtOAc in hexane) to give **SI-6** as a white solid.

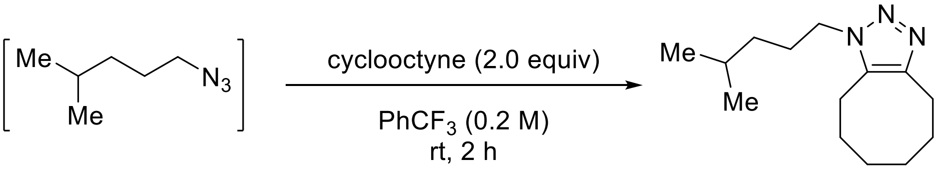

9 **SI-6**

^{1}H NMR (400 MHz, $CDCl_3$): δ (ppm) = 4.17 (t, *J* = 7.5 Hz, 2H), 2.94 – 2.84 (m, 2H), 2.80 – 2.70 (m, 2H), 1.90 – 1.70 (m, 6H), 1.59 – 1.40 (m, 5H), 1.26 – 1.17 (m, 2H), 0.88 (d, *J* = 6.6 Hz, 6H).

^{13}C NMR (101 MHz, $CDCl_3$): δ (ppm) = 144.8, 132.8, 48.2, 35.8, 28.5, 28.4, 27.8, 26.8, 26.1, 25.0, 24.8, 22.6, 21.8.

IR (thin film, cm^{-1}): 2930, 2854, 1457, 1367, 1232, 1207, 1171, 1088, 1006, 863.

HRMS (ESI+): *m/z* for $C_{14}H_{26}N_3$ $[M+H]^+$: calc.: 236.2121, found: 236.2121.

TLC: R_f = 0.47 (SiO_2, 35% acetone in hexane).

8. General Procedures for the Hydroazidation

GP1: Hydroazidation of Unactivated Alkenes in the 350 W photoreactor

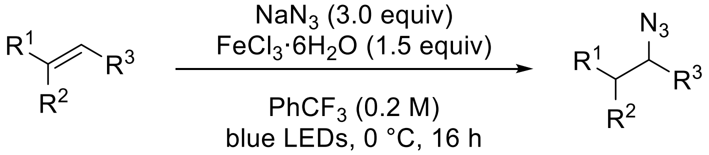

To a glass vial charged with $FeCl_3·6H_2O$ (810 mg, 0.300 mmol, 1.50 equiv) in $PhCF_3$ (1.00 mL, 0.200 M) were added alkene starting material (0.200 mmol, 1.00 equiv), and NaN_3 (39.0 mg, 0.600 mmol, 3.00 equiv). The vial was equipped with a magnetic stirrer bar and capped with a screwcap. The reaction was stirred for 30 min at room temperature and irradiated in a 350 W photoreactor (*vide supra*) at 0 °C for the indicated time. The crude reaction mixture was filtered over Celite which was rinsed with additional $PhCF_3$. The filtrate was concentrated *in vacuo* and purification using silica gel chromatography with the appropriate eluent was employed to obtain pure product.

GP2: Hydroazidation of Unactivated Alkenes with a 40 W blue LED Kessil light

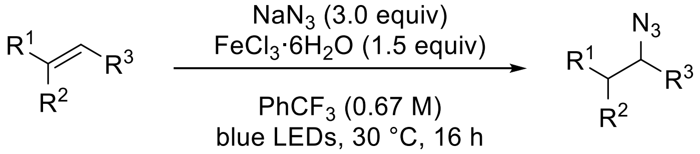

To a glass vial charged with $FeCl_3·6H_2O$ (810 mg, 3.00 mmol, 1.50 equiv) in $PhCF_3$ (3.00 mL, 0.667 M) were added alkene starting material (2.00 mmol, 1.00 equiv), and NaN_3 (390 mg, 6.00 mmol, 3.00 equiv). The vial was equipped with a magnetic stirrer bar and capped with a screwcap. The reaction was stirred for 30 min at room temperature and irradiated with a 40 W blue LED Kessil light (*vide supra*) at room temperature for the indicated time. The crude reaction mixture was filtered over Celite which was rinsed with additional $PhCF_3$. The filtrate was concentrated *in vacuo* and purification using silica gel chromatography with the appropriate eluent was employed to obtain pure product.

Note: When conducted at larger scale (2.00 mmol) with a 40 W Kessil light, the hydroazidation reaction warms up to approximately 30 °C internal temperature.

Note: The hydroazidation reaction is heterogeneous. In the optimization studies, higher yields were generally observed if the reaction mixture was stirred (900 rpm) at room temperature for 30 min before irradiation (see general procedure). During this time, the color of the undissolved solids changed from yellow/orange to black.

Note: In the optimization studies, the order of addition for alkene, iron salt, azide, and solvent did not significantly affect the observed yields. However, it is generally not advised to mix $FeCl_3 \cdot 6H_2O$ and the substrate directly without prior addition of solvent as this led to decomposition for certain starting materials.

Note: After completion of the reaction, hydrazoic acid was detected in the headspace of the vial. Consequently, every work-up should be conducted in a well-ventilated fume hood and the vial needs to be closed during the reaction.

Unsuccessful substrates

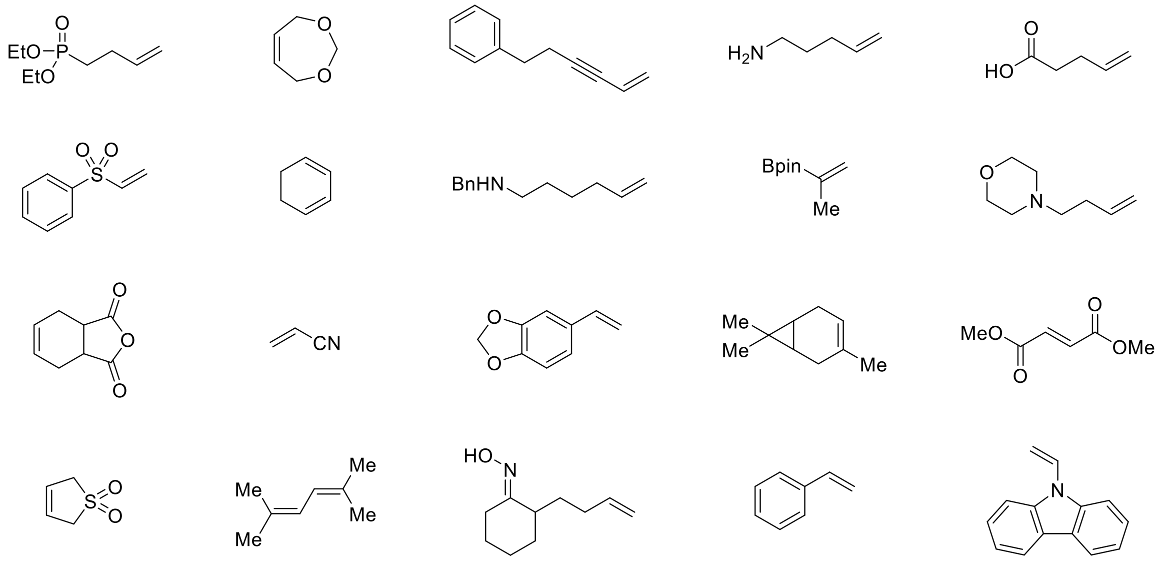

9. Substrate Scope

Compound 2a:

(4-Azidobutyl)benzene

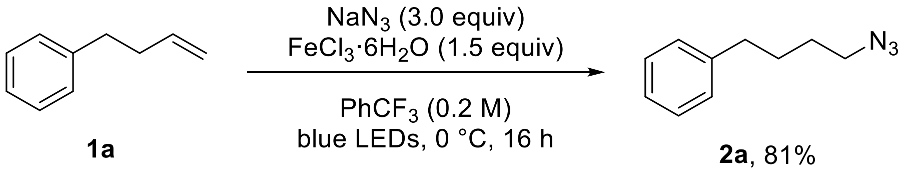

Azide **2a** was prepared via GP1 from alkene **1a** (30.0 µL, 26.4 mg, 0.200 mmol, 1.00 equiv) in $PhCF_3$. The crude product was purified via flash column chromatography (0-2% Et_2O in pentane) to give **2a** as a colorless oil. The product was isolated as an inseparable 19:1 mixture of regioisomers.

Note: Azide **2a** was prepared on larger scale via GP2 from alkene **1a** (300 µL, 264 mg, 2.00 mmol, 1.00 equiv) in $PhCF_3$. The crude product was purified via flash column chromatography (100% hexane) to give **2a** as a colorless oil in 75% (264 mg, 1.51 mmol) yield.

Yield: 28.5 mg, 0.163 mmol, 81%, rr = 19:1

^{1}H NMR (400 MHz, $CDCl_3$): δ (ppm) = 7.33 – 7.27 (m, 2H), 7.24 – 7.15 (m, 2H), 3.29 (t, J = 6.7 Hz, 2H), 2.66 (t, J = 7.4 Hz, 2H), 1.78 – 1.59 (m, 4H).

^{13}C NMR (101 MHz, $CDCl_3$): δ (ppm) = 142.0, 128.5, 128.5, 126.1, 51.5, 35.5, 28.6.

IR (thin film, cm^{-1}): 2938, 2861, 2093, 1496, 1453, 1265, 747, 699.

HRMS (EI): *m/z* for $C_{10}H_{12}N$ $[M–HN_2]^+$: calc.: 146.0964, found: 146.0964.

TLC: R_f = 0.27 (SiO_2, 2% EtOAc in hexane).

Compound 2b:

(2-Azidoethyl)dimethyl(phenyl)silane

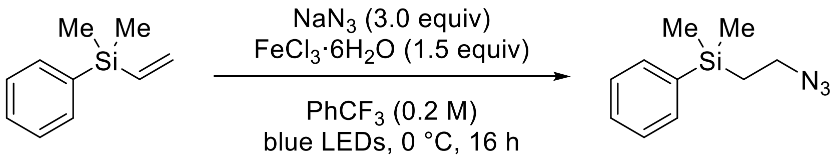

1b **2b**, 80%

Azide **2b** was prepared via GP1 from alkene **1b** (36.4 μL, 32.5 mg, 0.200 mmol, 1.00 equiv) in $PhCF_3$. The crude product was purified via flash column chromatography (100% hexane) to give **2b** as a colorless oil. The product was isolated as an inseparable 19:1 mixture of regioisomers.

Yield: 32.7 mg, 0.159 mmol, 80%, rr = 19:1

^{1}H NMR (400 MHz, $CDCl_3$): δ (ppm) = 7.53 – 7.48 (m, 2H), 7.41 – 7.36 (m, 3H), 3.33 – 3.23 (m, 2H), 1.23 – 1.16 (m, 2H), 0.34 (s, 6H).

^{13}C NMR (101 MHz, $CDCl_3$): δ (ppm) = 137.7, 133.6, 129.5, 128.1, 48.1, 15.9, -2.9.

IR (thin film, cm^{-1}): 2957, 2087, 1428, 1296, 1250, 1181, 1114, 1023, 929, 874, 836, 820, 780, 731, 700.

HRMS (EI): *m/z* for $C_{10}H_{14}NSi$ $[M–HN_2]^+$: calc.: 176.0890, found: 176.0890.

TLC: R_f = 0.85 (SiO_2, 20% EtOAc in hexane).

Compound 2c:

1-Azido-6-chlorohexane

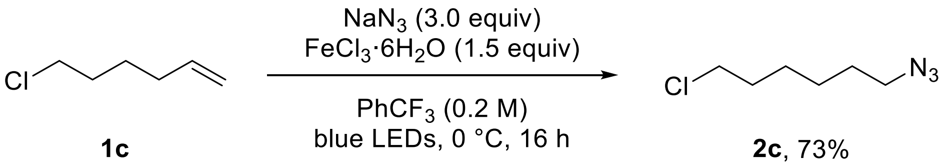

Azide **2c** was prepared via GP1 from alkene **1c** (26.5 µL, 23.7 mg, 0.200 mmol, 1.00 equiv) in $PhCF_3$. The crude product was purified via flash column chromatography (0-2% Et_2O in pentane) to give **2c** as a colorless oil. The product was isolated as an inseparable 19:1 mixture of regioisomers.

Note: Due to potential volatility of the product, the solvent was evaporated at 600 mbar at 35 °C.

Yield: 23.5 mg, 0.145 mmol, 73%, rr = 19:1

^{1}H NMR (400 MHz, $CDCl_3$): δ (ppm) = 3.54 (t, *J* = 6.6 Hz, 2H), 3.28 (t, *J* = 6.9 Hz, 2H), 1.84 – 1.73 (m, 2H), 1.68 – 1.56 (m, 2H), 1.53 – 1.36 (m, 4H).

^{13}C NMR (101 MHz, $CDCl_3$): δ (ppm) = 51.5, 45.0, 32.5, 28.9, 26.6, 26.2.

IR (thin film, cm^{-1}): 2938, 2862, 2093, 1455, 1349, 1259, 730, 651, 559.

HRMS (EI): *m/z* for $C_6H_{11}ClN$ $[M–HN_2]^+$: calc.: 132.0575, found: 132.0575

TLC: R_f = 0.61 (SiO_2, 5% EtOAc in hexane).

Compound 2d:

1-Azido-6-bromohexane

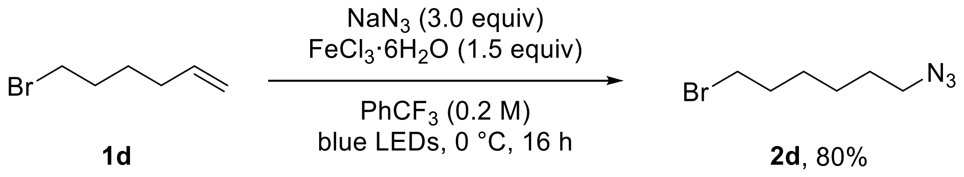

Azide **2d** was prepared via GP1 from alkene **1d** (26.7 µL, 32.6 mg, 0.200 mmol, 1.00 equiv) in $PhCF_3$. The crude product was purified via flash column chromatography (0-2% Et_2O in pentane) to give **2d** as a colorless oil. The product was isolated as an inseparable 16:1 mixture of regioisomers.

Note: Due to potential volatility of the product, the solvent was evaporated at 600 mbar at 35 °C.

Note: Azide **2d** was prepared on larger scale via GP2 from alkene **1d** (267 µL, 326 mg, 2.00 mmol, 1.00 equiv) in $PhCF_3$. The crude product was purified via flash column chromatography (100% pentane) to give **2d** as a colorless oil in 74% (304 mg, 1.48 mmol) yield.

Yield: 32.9 mg, 0.160 mmol, 80%, rr = 16:1

^{1}H NMR (400 MHz, $CDCl_3$): δ (ppm) = 3.41 (t, J = 6.7 Hz, 2H), 3.28 (t, J = 6.9 Hz, 2H), 1.97 – 1.81 (m, 2H), 1.66 – 1.58 (m, 2H), 1.52 – 1.36 (m, 4H).

^{13}C NMR (101 MHz, $CDCl_3$): δ (ppm) = 51.5, 33.8, 32.7, 28.8, 27.8, 26.0.

IR (thin film, cm^{-1}): 2936, 2860, 2090, 1455, 1252, 728, 644, 560.

HRMS (EI): *m/z* for $C_6H_{11}BrN$ $[M–HN_2]^+$: calc.: 176.0069, found: 176.0067.

TLC: R_f = 0.59 (SiO_2, 5% EtOAc in hexane).

Compound 2e:

1-Azidododecane

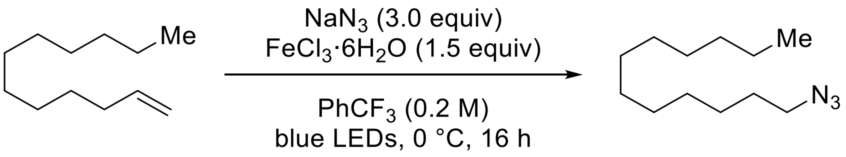

Azide **2e** was prepared via GP1 from alkene **1e** (44.3 µL, 33.7 mg, 0.200 mmol, 1.00 equiv) in $PhCF_3$. The crude product was purified via flash column chromatography (100% hexane) to give **2e** as a colorless oil. The product was isolated as an inseparable >20:1 mixture of regioisomers.

Note: Azide **2e** was prepared on larger scale via GP2 from alkene **1e** (443 µL, 337 mg, 2.00 mmol, 1.00 equiv) in $PhCF_3$. The crude product was purified via flash column chromatography (100% hexane) to give **2e** as a colorless oil in 80% (337 mg, 1.59 mmol) yield.

Yield: 36.5 mg, 0.173 mmol, 86%, rr > 20:1

^{1}H NMR (400 MHz, $CDCl_3$): δ (ppm) = 3.25 (t, J = 7.0 Hz, 2H), 1.64 – 1.53 (m, 2H), 1.27 (d, J = 6.3 Hz, 18H), 0.92 – 0.85 (m, 3H).

^{13}C NMR (101 MHz, $CDCl_3$): δ (ppm) = 51.7, 32.1, 29.8, 29.8, 29.7, 29.6, 29.5, 29.3, 29.0, 26.9, 22.8, 14.3.

IR (thin film, cm^{-1}): 2924, 2854, 2093, 1466, 1349, 1258, 722.

HRMS (EI): *m/z* for $C_{12}H_{24}N$ $[M–HN_2]^+$: calc.: 182.1903, found: 182.1902.

TLC: R_f = 0.50 (SiO_2, 2% EtOAc in hexane).

Compound 2f:

5-Azidopentyl 4-bromobenzoate

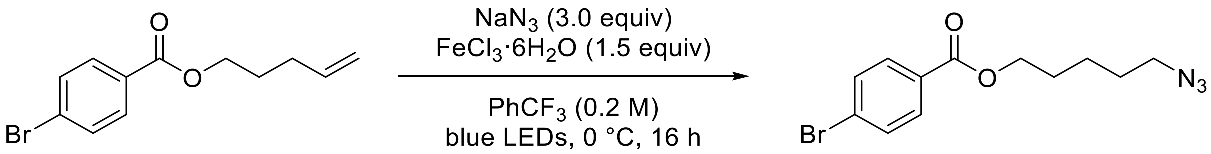

1f **2f**, 81%

Azide **2f** was prepared via GP1 from alkene **1f**[6] (53.8 mg, 0.200 mmol, 1.00 equiv) in $PhCF_3$. The crude product was purified via flash column chromatography (0-5% EtOAc in hexane) to give **2f** as a colorless oil. The product was isolated as an inseparable 12:1 mixture of regioisomers.

Yield: 50.7 mg, 0.162 mmol, 81%, rr = 12:1

^{1}H NMR (400 MHz, $CDCl_3$): δ (ppm) = 7.89 (d, J = 8.8 Hz, 2H), 7.58 (d, J = 8.7 Hz, 2H), 4.32 (t, J = 6.5 Hz, 2H), 3.31 (t, J = 6.7 Hz, 2H), 1.87 – 1.75 (m, 2H), 1.73 – 1.63 (m, 2H), 1.59 – 1.47 (m, 2H).

^{13}C NMR (101 MHz, $CDCl_3$): δ (ppm) = 166.0, 131.9, 131.2, 129.4, 128.2, 65.1, 51.4, 28.7, 28.4, 23.5.

IR (thin film, cm^{-1}): 2943, 2866, 2094, 1717, 1591, 1398, 1268, 1173, 1102, 1069, 1012, 848, 756, 683.

HRMS (ESI+): m/z for $C_{12}H_{14}BrN_3NaO_2$ $[M+Na]^+$: calc.: 334.0162, found: 334.0163.

TLC: R_f = 0.36 (SiO_2, 5% EtOAc in hexane).

Compound 2g:

5-Azidopentyl 4-methoxybenzoate

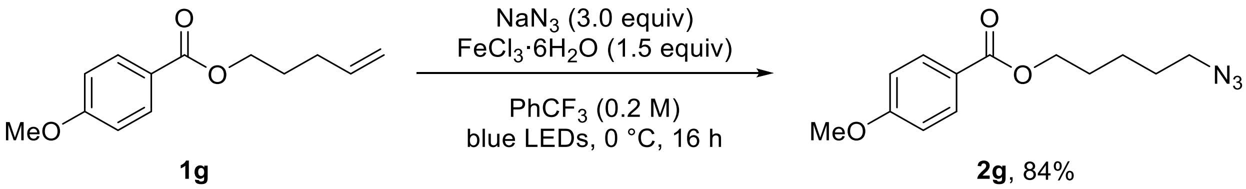

Azide **2g** was prepared via GP1 from alkene **1g**[7] (44.1 mg, 0.200 mmol, 1.00 equiv) in $PhCF_3$. The crude product was purified via flash column chromatography (0-5% EtOAc in hexane) to give **2g** as a colorless oil. The product was isolated as an inseparable 19:1 mixture of regioisomers.

Yield: 44.2 mg, 0.168 mmol, 84%, rr = 19:1

^{1}H NMR (400 MHz, $CDCl_3$): δ (ppm) = 7.99 (d, *J* = 9.1 Hz, 2H), 6.92 (d, *J* = 9.1 Hz, 2H), 4.30 (t, *J* = 6.5 Hz, 2H), 3.86 (s, 3H), 3.30 (t, *J* = 6.8 Hz, 2H), 1.84 – 1.75 (m, 2H), 1.73 – 1.64 (m, 2H), 1.58 – 1.49 (m, 2H).

^{13}C NMR (101 MHz, $CDCl_3$): δ (ppm) = 166.5, 163.5, 131.7, 122.9, 113.7, 64.5, 55.6, 51.4, 28.7, 28.5, 23.5.

IR (thin film, cm^{-1}): 2941, 2867, 2094, 1709, 1606, 1511, 1273, 1253, 1167, 1101, 1030, 848, 770, 697, 613.

HRMS (ESI+): *m/z* for $C_{13}H_{17}N_3NaO_3$ $[M+Na]^+$: calc.: 286.1162, found: 286.1162.

TLC: R_f = 0.22 (SiO_2, 5% EtOAc in hexane).

Compound 2h:

5-Azidopentyl 4-(trifluoromethyl)benzoate

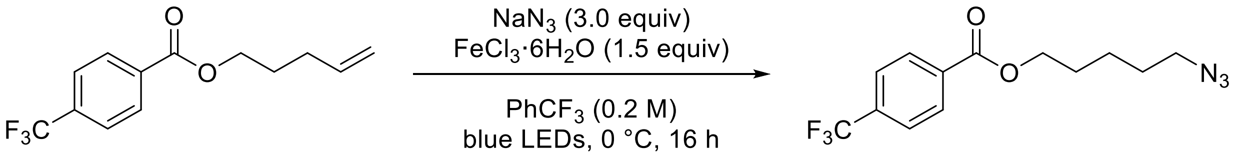

1h **2h**, 90%

Azide **2h** was prepared via GP1 from alkene **1h**[8] (51.6 mg, 0.200 mmol, 1.00 equiv) in $PhCF_3$. The crude product was purified via flash column chromatography (0-5% EtOAc in hexane) to give **2h** as a colorless oil. The product was isolated as an inseparable 19:1 mixture of regioisomers.

Yield: 54.1 mg, 0.180 mmol, 90%, rr = 19:1

^{1}H NMR (400 MHz, $CDCl_3$): δ (ppm) = 8.15 (d, J = 8.0 Hz, 2H), 7.71 (d, J = 8.1 Hz, 2H), 4.37 (t, J = 6.6 Hz, 2H), 3.31 (t, J = 6.7 Hz, 2H), 1.89 – 1.78 (m, 2H), 1.74 – 1.64 (m, 2H), 1.61 – 1.48 (m, 2H).

^{13}C NMR (101 MHz, $CDCl_3$): δ (ppm) = 165.5, 134.6 (q, J = 32.5 Hz), 133.7, 130.1, 125.6 (q, J = 3.8 Hz), 123.8 (q, J = 272.8 Hz), 65.4, 51.4, 28.7, 28.4, 23.4.

^{19}F NMR (377 MHz, $CDCl_3$): δ (ppm) = -63.12.

IR (thin film, cm^{-1}): 2945, 2869, 2096, 1723, 1412, 1325, 1274, 1167, 1126, 1100, 1066, 1018, 863, 776, 704, 593.

HRMS (ESI+): *m/z* for $C_{13}H_{14}F_3N_3NaO_2$ $[M+Na]^+$: calc.: 324.0930, found: 324.0929.

TLC: R_f = 0.37 (SiO_2, 5% EtOAc in hexane).

Compound 2i:

6-Azidohexan-1-ol

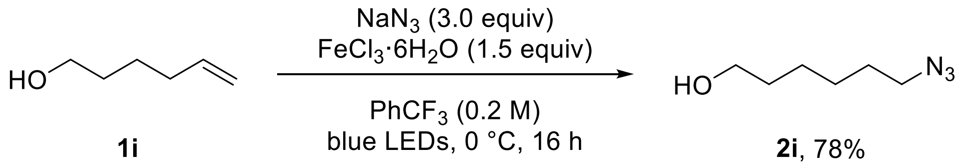

Azide **2i** was prepared via GP1 from alkene **1i** (24.0 µL, 20.0 mg, 0.200 mmol, 1.00 equiv) in $PhCF_3$. The crude product was purified via flash column chromatography (0-20% EtOAc in hexane) to give **2i** as a colorless oil. The product was isolated as an inseparable >20:1 mixture of regioisomers.

Note: Azide **2i** was prepared on larger scale via GP2 from alkene **1i** (240 µL, 200 mg, 2.00 mmol, 1.00 equiv) in $PhCF_3$. The crude product was purified via flash column chromatography (0-20% EtOAc in hexane) to give **2i** as a colorless oil in 71% (203 mg, 1.42 mmol) yield.

Yield: 22.4 mg, 0.156 mmol, 78%, rr > 20:1

^{1}H NMR (400 MHz, $CDCl_3$): δ (ppm) = 3.66 (q, J = 6.3 Hz, 2H), 3.27 (t, J = 6.9 Hz, 2H), 1.66 – 1.53 (m, 4H), 1.47 – 1.36 (m, 4H).

^{13}C NMR (101 MHz, $CDCl_3$): δ (ppm) = 63.0, 51.5, 32.7, 29.0, 26.7, 25.5.

IR (thin film, cm^{-1}): 3337, 2937, 2863, 2096, 1457, 1262, 1056.

HRMS (EI): *m/z* for $C_6H_{12}NO$ $[M–HN_2]^+$: calc.: 114.0913, found: 114.0913.

TLC: R_f = 0.14 (SiO_2, 20% EtOAc in hexane).

Compound 2j:

***N*-(5-Azidopentyl)-4-methylbenzenesulfonamide**

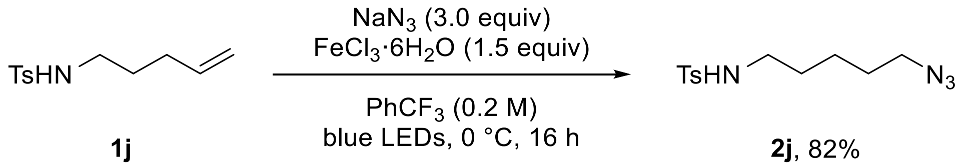

Azide **2j** was prepared via GP1 from alkene **1j**[9] (47.9 mg, 0.200 mmol, 1.00 equiv) in $PhCF_3$. The crude product was purified via flash column chromatography (10-30% EtOAc in hexane) to give **2j** as a colorless oil. The product was isolated as an inseparable 16:1 mixture of regioisomers.

Yield: 46.3 mg, 0.164 mmol, 82%, rr = 16:1

^{1}H NMR (400 MHz, $CDCl_3$): δ (ppm) = 7.75 (d, *J* = 8.4 Hz, 2H), 7.31 (d, *J* = 7.9 Hz, 2H), 4.56 (t, *J* = 6.2 Hz, 1H), 3.21 (t, *J* = 6.8 Hz, 2H), 2.97 – 2.91 (m, 2H), 2.43 (s, 3H), 1.58 – 1.45 (m, 4H), 1.39 – 1.30 (m, 2H).

^{13}C NMR (101 MHz, $CDCl_3$): δ (ppm) = 143.6, 137.1, 129.9, 127.2, 51.3, 43.1, 29.3, 28.4, 23.8, 21.7.

IR (thin film, cm^{-1}): 3282, 2941, 2866, 2096, 1425, 1324, 1158, 1093, 815, 663, 551.

HRMS (ESI+): *m/z* for $C_{12}H_{18}N_4NaO_2S$ $[M+Na]^+$: calc.: 305.1043, found: 305.1036.

TLC: R_f = 0.14 (SiO_2, 20% EtOAc in hexane).

Compound 2k:

5-Azidopentyl tosylcarbamate

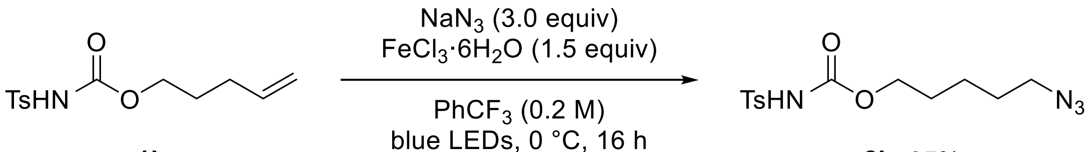

1k **2k**, 65%

Azide **2k** was prepared via GP1 from alkene **1k**[10] (56.7 mg, 0.200 mmol, 1.00 equiv) in $PhCF_3$. The crude product was purified via flash column chromatography (20-50% EtOAc in hexane) to give **2k** as a colorless oil. The product was isolated as an inseparable 16:1 mixture of regioisomers.

Yield: 42.2 mg, 0.129 mmol, 65%, rr = 16:1

^{1}H NMR (400 MHz, $CDCl_3$): δ (ppm) = 7.92 (d, *J* = 8.5 Hz, 2H), 7.35 (d, *J* = 7.9 Hz, 2H), 4.09 (t, *J* = 6.5 Hz, 2H), 3.25 (t, *J* = 6.8 Hz, 2H), 2.45 (s, 3H), 1.68 – 1.52 (m, 4H), 1.42 – 1.30 (m, 2H).

^{13}C NMR (101 MHz, $CDCl_3$): δ (ppm) = 150.5, 145.3, 135.6, 129.8, 128.5, 66.8, 51.3, 28.5, 28.1, 23.0, 21.8.

IR (thin film, cm^{-1}): 3242, 2942, 2098, 1750, 1448, 1349, 1225, 1161, 1091, 847, 816, 771, 662, 577, 547.

HRMS (ESI+): *m/z* for $C_{13}H_{18}N_4NaO_4S$ $[M+Na]^+$: calc.: 349.0941, found: 349.0934.

TLC: R_f = 0.12 (SiO_2, 20% EtOAc in hexane).

Compound 2l:

7-Azido-*N*-tosylheptanamide

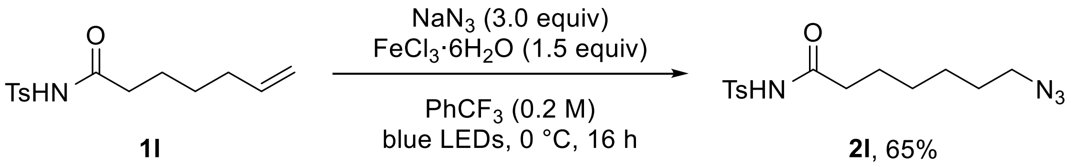

Azide **2l** was prepared via GP1 from alkene **1l**[10] (76.3 mg, 0.200 mmol, 1.00 equiv) in $PhCF_3$. The crude product was purified via flash column chromatography (0-30% EtOAc in hexane) to give **2l** as a colorless oil. The product was isolated as an inseparable 14:1 mixture of regioisomers.

Yield: 42.1 mg, 0.130 mmol, 65%, rr = 14:1

^{1}H NMR (400 MHz, $CDCl_3$): δ (ppm) = 8.70 (bs, 1H), 7.97 – 7.91 (m, 2H), 7.38 – 7.31 (m, 2H), 3.20 (t, *J* = 6.9 Hz, 2H), 2.45 (s, 3H), 2.26 (t, *J* = 7.4 Hz, 2H), 1.62 – 1.48 (m, 4H), 1.37 – 1.20 (m, 4H).

^{13}C NMR (101 MHz, $CDCl_3$): δ (ppm) = 170.9, 145.4, 135.6, 129.8, 128.5, 51.4, 36.2, 28.7, 28.4, 26.4, 24.2, 21.8.

IR (thin film, cm^{-1}): 6248, 2936, 2862, 2096, 1721, 1696, 1597, 1439, 1343, 1292, 1261, 1166, 1086, 852, 815, 662, 550.

HRMS (ESI+): *m/z* for $C_{14}H_{20}N_4NaO_3S$ $[M+Na]^+$: calc.: 347.1148, found: 347.1142.

TLC: R_f = 0.55 (SiO_2, 50% EtOAc in hexane).

Compound 2m:

((5-Azidopentyl)oxy)(tert-butyl)dimethylsilane

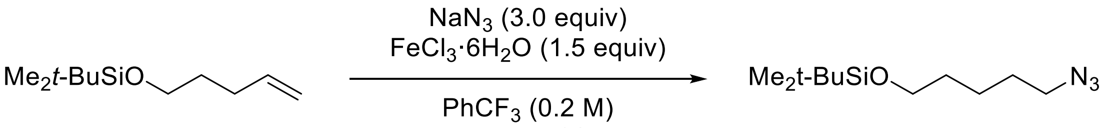

1m **2m**, 61%

Azide **2m** was prepared via GP1 from alkene **1m**[11] (40.1 mg, 0.200 mmol, 1.00 equiv) in $PhCF_3$. The crude product was purified via flash column chromatography (0-5% Et_2O in pentane) to give **2m** as a colorless oil. The product was isolated as an inseparable 16:1 mixture of regioisomers.

Yield: 29.8 mg, 0.122 mmol, 61%, rr = 16:1

^{1}H NMR (400 MHz, $CDCl_3$): δ (ppm) = 3.61 (t, *J* = 6.3 Hz, 2H), 3.27 (t, *J* = 7.0 Hz, 2H), 1.67 – 1.59 (m, 2H), 1.58 – 1.50 (m, 2H), 1.47 – 1.38 (m, 2H), 0.89 (s, 9H), 0.05 (s, 6H).

^{13}C NMR (101 MHz, $CDCl_3$): δ (ppm) = 63.0, 51.6, 32.4, 28.8, 26.1, 23.3, 18.5, -5.2.

IR (thin film, cm^{-1}): 2930, 2886, 2858, 2094, 1472, 1254, 1100, 834, 775, 662.

HRMS (ESI+): *m/z* for $C_{11}H_{25}N_3NaOSi$ $[M+Na]^+$: calc.: 266.1659, found: 266.1661.

TLC: R_f = 0.56 (SiO_2, 5% EtOAc in hexane).

Compound 2n:

5-Azidopentyl methyl carbonate

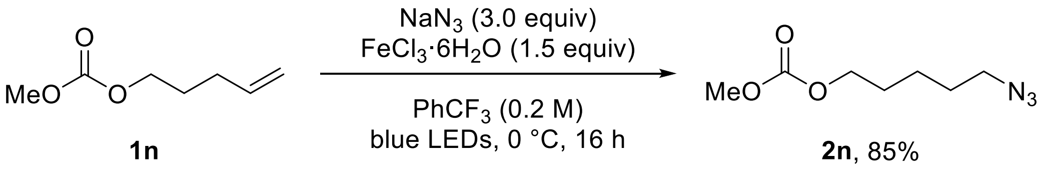

Azide **2n** was prepared via GP1 from alkene **1n**[12] (28.8 mg, 0.200 mmol, 1.00 equiv) in $PhCF_3$. The crude product was purified via flash column chromatography (0-20% Et_2O in pentane) to give **2n** as a colorless oil. The product was isolated as an inseparable 19:1 mixture of regioisomers.

Yield: 31.8 mg, 0.170 mmol, 85%, rr = 19:1

^{1}H NMR (400 MHz, $CDCl_3$): δ (ppm) = 4.13 (t, J = 6.5 Hz, 2H), 3.76 (s, 3H), 3.26 (t, J = 6.8 Hz, 2H), 1.74 – 1.57 (m, 4H), 1.50 – 1.41 (m, 2H).

^{13}C NMR (101 MHz, $CDCl_3$): δ (ppm) = 155.9, 67.8, 54.8, 51.3, 28.6, 28.3, 23.1.

IR (thin film, cm^{-1}): 2956, 2868, 2094, 1746, 1443, 1259, 961, 793.

HRMS (ESI+): *m/z* for $C_7H_{13}N_3NaO_3$ $[M+Na]^+$: calc.: 210.0849, found: 210.0851.

TLC: R_f = 0.60 (SiO_2, 20% EtOAc in hexane).

Compound 2o:

(4-Azidobutyl)(phenyl)sulfane

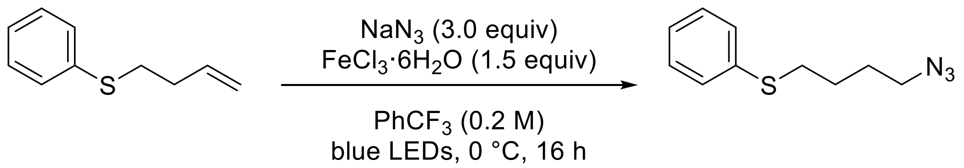

1o **2o**, 43%

Azide **2i** was prepared via GP1 from alkene **1i**[13] (32.9 mg, 0.200 mmol, 1.00 equiv) in $PhCF_3$. The crude product was purified via flash column chromatography (0-10% Et_2O in pentane) to give **2i** as a colorless oil. The product was isolated as an inseparable 17:1 mixture of regioisomers.

Yield: 17.9 mg, 0.086 mmol, 43%, rr = 17:1

^{1}H NMR (400 MHz, $CDCl_3$): δ (ppm) = 7.36 – 7.26 (m, 4H), 7.22 – 7.16 (m, 1H), 3.32 – 3.26 (m, 2H), 2.98 – 2.92 (m, 2H), 1.76 – 1.71 (m, 4H).

^{13}C NMR (101 MHz, $CDCl_3$): δ (ppm) = 136.4, 129.5, 129.1, 126.2, 51.1, 33.4, 28.0, 26.4.

IR (thin film, cm^{-1}): 2935, 2865, 2093, 1584, 1480, 1438, 1266, 1092, 1025, 738, 690.

HRMS (EI): *m/z* for $C_{10}H_{13}NS$ $[M–N_2]^+$: calc.: 179.0763, found: 179.0758.

TLC: R_f = 0.48 (SiO_2, 10% EtOAc in hexane).

Compound 2p:

11-Azidoundecanal

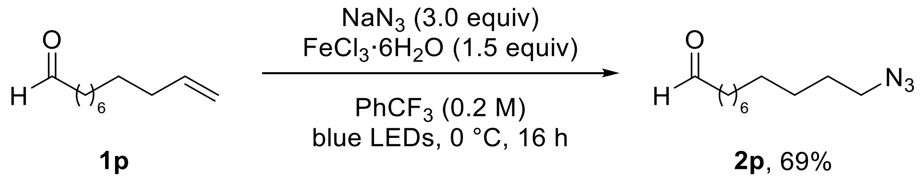

1p **2p**, 69%

Azide **2p** was prepared via GP1 from alkene **1p** (39.8 µL, 33.6 mg, 0.200 mmol, 1.00 equiv) in $PhCF_3$. The crude product was purified via flash column chromatography (0-5% EtOAc in hexane) to give **2p** as a colorless oil. The product was isolated as an inseparable 12:1 mixture of regioisomers.

Yield: 29.2 mg, 0.138 mmol, 69%, rr = 12:1

^{1}H NMR (400 MHz, $CDCl_3$): δ (ppm) = 9.76 (t, *J* = 1.9 Hz, 1H), 3.25 (t, *J* = 7.0 Hz, 2H), 2.42 (td, *J* = 7.3, 1.9 Hz, 2H), 1.68 – 1.55 (m, 4H), 1.38 – 1.25 (m, 12H).

^{13}C NMR (101 MHz, $CDCl_3$): δ (ppm) = 203.0, 51.6, 44.1, 29.5, 29.4, 29.4, 29.3, 29.3, 29.0, 26.8, 22.2.

IR (thin film, cm^{-1}): 2929, 2856, 2719, 2268, 2096, 1726, 1465, 1350, 1258, 723.

HRMS (ESI+): *m/z* for $C_{11}H_{21}N_3NaO$ $[M+Na]^+$: calc.: 234.1577, found: 234.1575.

TLC: R_f = 0.33 (SiO_2, 5% EtOAc in hexane).

Compound 2q:

5-Azidopentyl cyclopropanecarboxylate

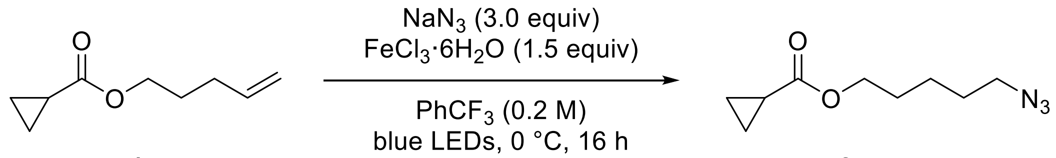

1q **2q**, 75%

Azide **2q** was prepared via GP1 from alkene **1q** (30.8 mg, 0.200 mmol, 1.00 equiv) in $PhCF_3$. The crude product was purified via flash column chromatography (0-10% EtOAc in hexane) to give **2q** as a colorless oil. The product was isolated as an inseparable 12:1 mixture of regioisomers.

Yield: 29.5 mg, 0.150 mmol, 75%, rr = 12:1

^{1}H NMR (400 MHz, $CDCl_3$): δ (ppm) = 4.07 (t, *J* = 6.5 Hz, 2H), 3.28 (t, *J* = 6.9 Hz, 2H), 1.71 – 1.55 (m, 5H), 1.49 – 1.40 (m, 2H), 1.02 – 0.95 (m, 2H), 0.88 – 0.82 (m, 2H).

^{13}C NMR (101 MHz, $CDCl_3$): δ (ppm) = 175.1, 64.3, 51.4, 28.7, 28.4, 23.4, 13.0, 8.5.

IR (thin film, cm^{-1}): 2945, 2868, 2096, 1726, 1456, 1404, 1269, 1200, 1174, 1077, 1032, 901,825, 747.

HRMS (ESI+): *m/z* for $C_9H_{15}N_3NaO_2$ $[M+Na]^+$: calc.: 220.1056, found: 220.1055.

TLC: R_f = 0.33 (SiO_2, 5% EtOAc in hexane).

Compound 2r:

6-Azidohexan-2-one

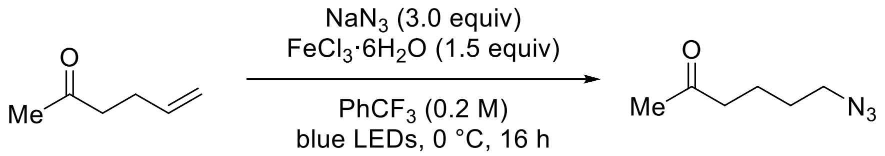

1r **2r**, 75%

Azide **2r** was prepared via GP1 from alkene **1r** (23.2 µL, 19.6 mg, 0.200 mmol, 1.00 equiv) in $PhCF_3$. The crude product was purified via flash column chromatography (0-40% Et_2O in pentane) to give **2r** as a colorless oil. The product was isolated as an inseparable 13:1 mixture of regioisomers.

Note: Due to potential volatility of the product, the solvent was evaporated at 600 mbar at 35 °C.

Note: Azide **2r** was prepared on larger scale via GP2 from alkene **1r** (232 µL, 196 mg, 2.00 mmol, 1.00 equiv) in $PhCF_3$. The crude product was purified via flash column chromatography (0-40% Et_2O in pentane) to give **2r** as a colorless oil in 66% (185 mg, 1.31 mmol) yield.

Yield: 21.1 mg, 0.149 mmol, 75%, rr = 13:1

^{1}H NMR (400 MHz, $CDCl_3$): δ (ppm) = 3.28 (t, J = 6.6 Hz, 2H), 2.47 (t, J = 7.0 Hz, 2H), 2.14 (s, 3H), 1.70 – 1.54 (m, 4H).

^{13}C NMR (101 MHz, $CDCl_3$): δ (ppm) = 208.3, 51.3, 43.0, 30.0, 28.4, 21.0.

IR (thin film, cm^{-1}): 2942, 2873, 2093, 1714, 1453, 1411, 1355, 1252, 1161, 956, 898, 725.

HRMS (ESI+): m/z for $C_6H_{11}N_3NaO$ $[M+Na]^+$: calc.: 164.0794, found: 164.0796.

TLC: R_f = 0.16 (SiO_2, 5% EtOAc in hexane).

Compound 2s:

1-Azido-6-(prop-2-yn-1-yloxy)hexane

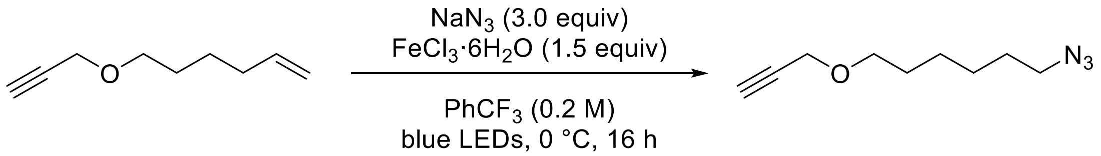

1s **2s**, 62%

Azide **2s** was prepared via GP1 from alkene **1s**[14] (27.6 mg, 0.200 mmol, 1.00 equiv) in $PhCF_3$. The crude product was purified via flash column chromatography (0-15% Et_2O in pentane) to give **2s** as a colorless oil. The product was isolated as an inseparable 12:1 mixture of regioisomers.

Yield: 22.6 mg, 0.125 mmol, 62%, rr = 12:1

^{1}H NMR (400 MHz, $CDCl_3$): δ (ppm) = 4.13 (d, J = 2.4 Hz, 2H), 3.51 (t, J = 6.5 Hz, 2H), 3.26 (t, J = 6.9 Hz, 2H), 2.42 (t, J = 2.4 Hz, 1H), 1.67 – 1.57 (m, 4H), 1.45 – 1.36 (m, 4H).

^{13}C NMR (101 MHz, $CDCl_3$): δ (ppm) = 80.1, 74.3, 70.1, 58.2, 51.5, 29.5, 28.9, 26.7, 25.8.

IR (thin film, cm^{-1}): 3299, 2937, 2860, 2092, 1456, 1352, 1260, 1097, 1018, 635.

HRMS (ESI+): *m/z* for $C_9H_{15}N_3NaO$ $[M+Na]^+$: calc.: 204.1107, found: 204.1105.

TLC: R_f = 0.70 (SiO_2, 20% EtOAc in hexane).

Compound 2t:

5-Azidopentyl thiophene-2-carboxylate

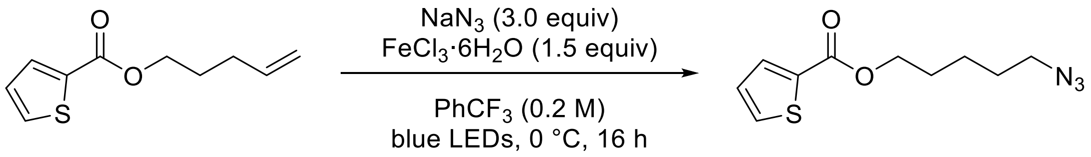

Azide **2t** was prepared via GP1 from alkene **1t**[15] (39.3 mg, 0.200 mmol, 1.00 equiv) in $PhCF_3$. The crude product was purified via flash column chromatography (0-5% EtOAc in hexane) to give **2t** as a colorless oil. The product was isolated as an inseparable 15:1 mixture of regioisomers.

Yield: 36.8 mg, 0.154 mmol, 77%, rr = 15:1

^{1}H NMR (400 MHz, $CDCl_3$): δ (ppm) = 7.79 (dd, J = 3.8, 1.2 Hz, 1H), 7.54 (dd, J = 5.0, 1.2 Hz, 1H), 7.09 (dd, J = 5.0, 3.7 Hz, 1H), 4.29 (t, J = 6.5 Hz, 2H), 3.29 (t, J = 6.8 Hz, 2H), 1.82 – 1.73 (m, 2H), 1.70 – 1.62 (m, 2H), 1.56 – 1.46 (m, 2H).

^{13}C NMR (101 MHz, $CDCl_3$): δ (ppm) = 162.3, 134.0, 133.4, 132.4, 127.8, 64.9, 51.3, 28.6, 28.3, 23.3.

IR (thin film, cm^{-1}): 2943, 2866, 2092, 1704, 1526, 1419, 1357, 1256, 1224, 1091, 1075, 1037, 859, 750, 719.

HRMS (ESI+): m/z for $C_{10}H_{13}N_3NaO_2S$ $[M+Na]^+$: calc.: 262.0621, found: 262.0614.

TLC: R_f = 0.57 (SiO_2, 20% EtOAc in hexane).

Compound 2u:

5-Azidopentyl furan-2-carboxylate

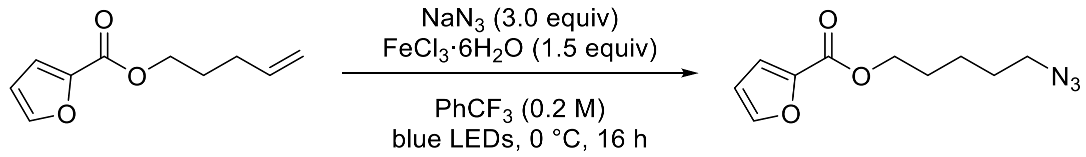

1u **2u**, 54%

Azide **2u** was prepared via GP1 from alkene **1u**[15] (36.0 mg, 0.200 mmol, 1.00 equiv) in $PhCF_3$. The crude product was purified via flash column chromatography (0-5% EtOAc in hexane) to give **2u** as a colorless oil. The product was isolated as an inseparable 12:1 mixture of regioisomers.

Yield: 24.0 mg, 0.108 mmol, 54%, rr = 12:1

^{1}H NMR (400 MHz, $CDCl_3$): δ (ppm) = 7.57 (dd, J = 1.7, 0.9 Hz, 1H), 7.17 (dd, J = 3.5, 0.9 Hz, 1H), 6.50 (dd, J = 3.5, 1.7 Hz, 1H), 4.31 (t, J = 6.6 Hz, 2H), 3.29 (t, J = 6.8 Hz, 2H), 1.83 – 1.73 (m, 2H), 1.70 – 1.62 (m, 2H), 1.55 – 1.47 (m, 2H).

^{13}C NMR (101 MHz, $CDCl_3$): δ (ppm) = 158.9, 146.4, 144.8, 118.0, 111.9, 64.7, 51.4, 28.6, 28.4, 23.3.

IR (thin film, cm^{-1}): 2944, 1868, 2096, 1721, 1581, 1475, 1399, 1295, 1231, 1180, 1119, 1014, 885, 763, 597.

HRMS (ESI+): m/z for $C_{10}H_{13}N_3NaO_3$ $[M+Na]^+$: calc.: 246.0849, found: 246.0849.

TLC: R_f = 0.52 (SiO_2, 20% EtOAc in hexane).

Compound 2v:

2-(6-Azidohexyl)isoindoline-1,3-dione

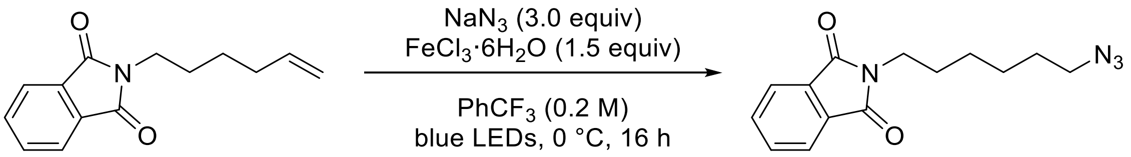

1v **2v**, 69%

Azide **2v** was prepared via GP1 from alkene **1v**[16] (45.9 mg, 0.200 mmol, 1.00 equiv) in $PhCF_3$. The crude product was purified via flash column chromatography (0-10% EtOAc in hexane) to give **2v** as a colorless oil. The product was isolated as an inseparable 14:1 mixture of regioisomers.

Yield: 37.7 mg, 0.138 mmol, 69%, rr = 14:1

^{1}H NMR (400 MHz, $CDCl_3$): δ (ppm) = 7.87 – 7.80 (m, 2H), 7.75 – 7.66 (m, 2H), 3.69 (t, J = 7.2 Hz, 2H), 3.25 (t, J = 6.9 Hz, 2H), 1.75 – 1.63 (m, 2H), 1.65 – 1.54 (m, 2H), 1.48 – 1.32 (m, 4H).

^{13}C NMR (101 MHz, $CDCl_3$): δ (ppm) = 168.6, 134.0, 132.3, 123.3, 51.5, 38.0, 28.8, 28.6, 26.5, 26.4.

IR (thin film, cm^{-1}): 2937, 2861, 2093, 1771, 1707, 1467, 427, 1395, 1370, 1245, 1056, 889, 718, 530.

HRMS (ESI+): *m/z* for $C_{14}H_{16}N_4NaO_2$ [M+Na]$^+$: calc.: 295.1165, found: 295.1168.

TLC: R_f = 0.36 (SiO_2, 20% EtOAc in hexane).

Compound 2w:

Oxetan-3-yl 5-azidopentanoate

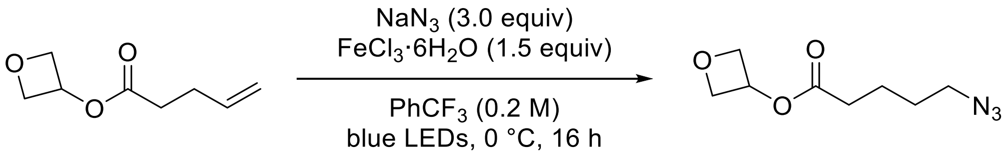

1w **2w**, 44%

Azide **2w** was prepared via GP1 from alkene **1w** (31.2 mg, 0.200 mmol, 1.00 equiv) in $PhCF_3$. The crude product was purified via flash column chromatography (0-15% EtOAc in hexane) to give **2w** as a colorless oil. The product was isolated as an inseparable 12:1 mixture of regioisomers.

Yield: 17.5 mg, 0.088 mmol, 44%, rr = 12:1

^{1}H NMR (400 MHz, $CDCl_3$): δ (ppm) = 5.44 (tt, *J* = 6.4, 5.3 Hz, 1H), 4.89 (ddd, *J* = 7.6, 6.4, 1.0 Hz, 2H), 4.63 (ddd, *J* = 7.5, 5.3, 1.0 Hz, 2H), 3.31 (t, *J* = 6.6 Hz, 2H), 2.41 (t, *J* = 7.2 Hz, 2H), 1.78 – 1.69 (m, 2H), 1.69 – 1.60 (m, 2H).

^{13}C NMR (101 MHz, $CDCl_3$): δ (ppm) = 172.5, 77.7, 68.0, 51.1, 33.5, 28.4, 22.1.

IR (thin film, cm^{-1}): 2953, 2881, 2096, 1737, 1456, 1351, 1251, 1159, 1101, 973, 899, 871.

HRMS (ESI+): *m/z* for $C_8H_{13}N_3NaO_3$ $[M+Na]^+$: calc.: 222.0849, found: 222.0848.

TLC: R_f = 0.07 (SiO_2, 5% EtOAc in hexane).

Compound 2x:

2-((6-Azidohexyl)oxy)-5-(trifluoromethyl)pyridine

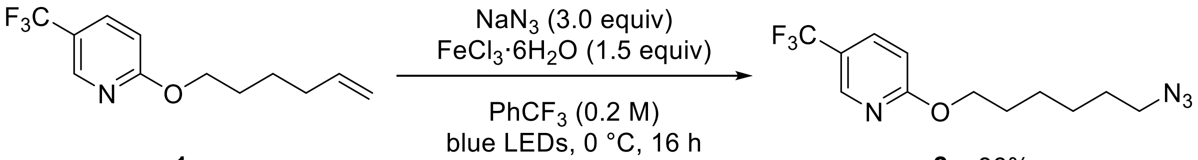

1x **2x**, 86%

Azide **2x** was prepared via GP1 from alkene **1x**[17] (49.0 mg, 0.200 mmol, 1.00 equiv) in $PhCF_3$. The crude product was purified via flash column chromatography (0-5% EtOAc in hexane) to give **2x** as a colorless oil. The product was isolated as an inseparable 17:1 mixture of regioisomers.

Yield: 49.8 mg, 0.172 mmol, 86%, rr = 17:1

^{1}H NMR (400 MHz, $CDCl_3$): δ (ppm) = 8.42 (dt, *J* = 2.7, 1.0 Hz, 1H), 7.79 – 7.71 (m, 1H), 6.79 (dt, *J* = 8.8, 0.8 Hz, 1H), 4.34 (t, *J* = 6.6 Hz, 2H), 3.27 (t, *J* = 6.9 Hz, 2H), 1.85 – 1.76 (m, 2H), 1.68 – 1.58 (m, 2H), 1.53 – 1.41 (m, 3H).

^{13}C NMR (101 MHz, $CDCl_3$): δ (ppm) = 166.1 (q, *J* = 1.3 Hz), 145.1 (q, *J* = 4.5 Hz), 135.7 (q, *J* = 3.0 Hz), 124.2 (d, *J* = 271.0 Hz), 119.9 (q, *J* = 33.0 Hz), 111.3, 66.7, 51.5, 28.9, 28.9, 26.6, 25.7.

^{19}F NMR (377 MHz, $CDCl_3$): δ (ppm) = -61.53.

IR (thin film, cm^{-1}): 2941, 2863, 2096, 1614, 1573, 1502, 1317, 1292, 1268, 1159, 1123, 1078, 1011, 836, 661.

HRMS (ESI+): *m/z* for $C_{12}H_{16}F_3N_4O$ $[M+H]^+$: calc.: 289.1271, found: 289.1266.

TLC: R_f = 0.56 (SiO_2, 10% EtOAc in hexane).

Compound 2y:

(4-Azido-3-methylbutyl)benzene

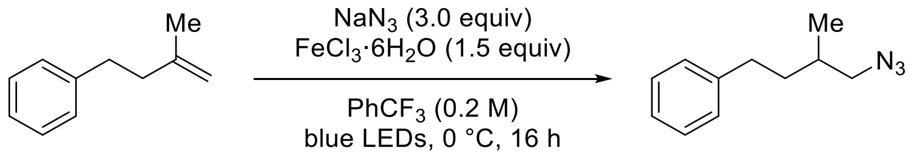

1y **2y**, 78%

Azide **2y** was prepared via GP1 from alkene **1y**[18] (29.2 mg, 0.200 mmol, 1.00 equiv) in $PhCF_3$. The crude product was purified via flash column chromatography (0-5% EtOAc in hexane) to give **2y** as a colorless oil. The product was isolated as an inseparable >20:1 mixture of regioisomers.

Yield: 29.6 mg, 0.156 mmol, 78%, rr > 20:1

^{1}H NMR (400 MHz, $CDCl_3$): δ (ppm) = 7.32 – 7.27 (m, 2H), 7.23 – 7.18 (m, 3H), 3.26 (dd, J = 12.0, 5.7 Hz, 1H), 3.17 (dd, J = 12.0, 6.5 Hz, 1H), 2.75 – 2.56 (m, 2H), 1.84 – 1.70 (m, 2H), 1.57 – 1.45 (m, 1H), 1.03 (d, J = 6.7 Hz, 3H).

^{13}C NMR (101 MHz, $CDCl_3$): δ (ppm) = 142.2, 128.5, 128.4, 126.0, 57.8, 36.0, 33.3, 33.3, 17.7.

IR (thin film, cm^{-1}): 2961, 2930, 2860, 2096, 1497, 1454, 1285, 747, 699.

HRMS (ESI+): m/z for $C_{11}H_{14}N$ $[M–HN_2]^+$: calc.: 160.1121, found: 160.1118.

TLC: R_f = 0.56 (SiO_2, 5% EtOAc in hexane).

Compound 2z:

((1r,4r)-4-(Azidomethyl)cyclohexyl)benzene

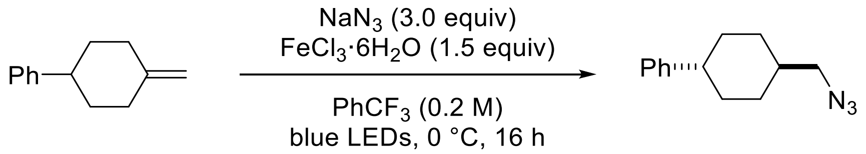

1z **2z**, 73%

Azide **2z** was prepared via GP1 from alkene **1z**[19] (34.5 mg, 0.200 mmol, 1.00 equiv) in $PhCF_3$. The crude product was purified via flash column chromatography (0-2% EtOAc in hexane) to give **2z** as a colorless oil. The corresponding regioisomer could not be identified in the ^{1}H NMR spectrum of the unpurified reaction mixture.

Yield: 31.4 mg, 0.146 mmol, 73%, dr = 12:1

^{1}H NMR (400 MHz, $CDCl_3$): δ (ppm) = 7.35 – 7.27 (m, 2H), 7.25 – 7.18 (m, 3H), 3.20 (d, J = 6.6 Hz, 2H), 2.50 (tt, J = 12.2, 3.3 Hz, 1H), 2.01 – 1.91 (m, 4H), 1.69 – 1.60 (m, 1H), 1.57 – 1.45 (m, 2H), 1.26 – 1.11 (m, 2H).

^{13}C NMR (101 MHz, $CDCl_3$): δ (ppm) = 147.2, 128.5, 126.9, 126.1, 58.1, 44.3, 37.9, 33.7, 31.0.

IR (thin film, cm^{-1}): 3027, 2923, 2854, 2091, 1493, 1450, 1254, 966, 891, 755, 698, 666, 537.

HRMS (EI): *m/z* for $C_{13}H_{16}N$ $[M–HN_2]^+$: calc.: 186.1277, found: 186.1275.

TLC: R_f = 0.57 (SiO_2, 5% EtOAc in hexane).

Compound 2aa:

(Azidomethyl)cyclohexane

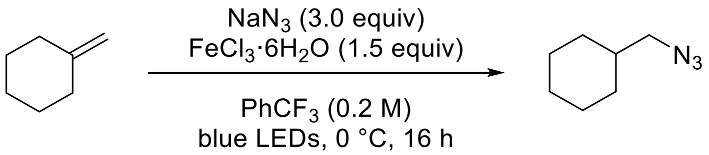

1aa **2aa**, 78%

Azide **2aa** was prepared via GP1 from alkene **1aa** (24.0 μL, 19.2 mg, 0.200 mmol, 1.00 equiv) in $PhCF_3$. Due to the volatility of the product, **2aa** was not isolated and the yield was determined via ^1H NMR with mesitylene as internal standard. Azide **2aa** is a known compound and the characterization data of the unpurified reaction mixture matched with the literature.[20] To verify the structural assignment, a one-pot derivatization procedure was devised (*vide infra*).

^{1}H NMR yield: 78%

Compound 2ab:

4-Azido-3-methylbutyl 4-methylbenzenesulfonate

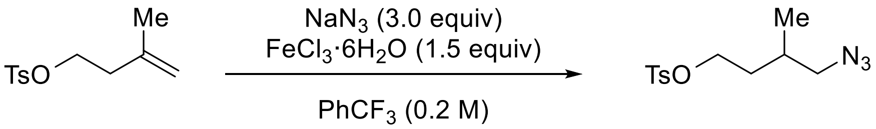

1ab **2ab**, 67%

Azide **2ab** was prepared via GP1 from alkene **1ab**[18] (48.1 mg, 0.200 mmol, 1.00 equiv) in $PhCF_3$. The crude product was purified via flash column chromatography (0-25% EtOAc in hexane) to give **2ab** as a colorless oil. The product was isolated as an inseparable 13:1 mixture of regioisomers.

Yield: 38.0 mg, 0.134 mmol, 67%, rr = 13:1

^{1}H NMR (400 MHz, $CDCl_3$): δ (ppm) = 7.81 – 7.76 (m, 2H), 7.40 – 7.32 (m, 2H), 4.14 – 4.02 (m, 2H), 3.15 (dd, *J* = 6.0, 2.5 Hz, 2H), 2.45 (s, 3H), 1.89 – 1.71 (m, 2H), 1.54 – 1.44 (m, 1H), 0.91 (d, *J* = 6.7 Hz, 3H).

^{13}C NMR (101 MHz, $CDCl_3$): δ (ppm) = 145.0, 133.1, 130.0, 128.0, 68.3, 57.3, 33.1, 30.2, 21.8, 17.3.

IR (thin film, cm^{-1}): 2965, 2931, 2098, 1598, 1459, 1357, 1290, 1189, 1175, 1097, 947, 885, 815, 762, 664, 554.

HRMS (ESI+): *m/z* for $C_{12}H_{17}N_3NaO_3S$ $[M+Na]^+$: calc.: 306.0883, found: 306.0884.

TLC: R_f = 0.41 (SiO_2, 20% EtOAc in hexane).

Compound 2ac:

Dimethyl 2-(3-azido-2-methylpropyl)malonate

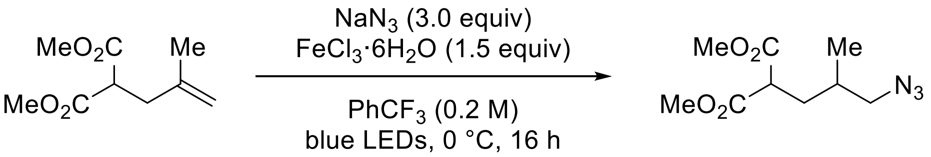

1ac **2ac**, 64%

Azide **2ac** was prepared via GP1 from alkene **1ac**[21] (37.2 mg, 0.200 mmol, 1.00 equiv) in $PhCF_3$. The crude product was purified via flash column chromatography (0-20% EtOAc in hexane) to give **2ac** as a colorless oil. The product was isolated as an inseparable 16:1 mixture of regioisomers.

Yield: 29.4 mg, 0.128 mmol, 64%, rr = 16:1

^{1}H NMR (400 MHz, $CDCl_3$): δ (ppm) = 3.75 (s, 3H), 3.74 (s, 3H), 3.48 (dd, *J* = 8.7, 6.5 Hz, 1H), 3.29 – 3.15 (m, 2H), 2.09 – 2.00 (m, 1H), 1.81 – 1.67 (m, 2H), 0.98 (d, *J* = 6.5 Hz, 3H).

^{13}C NMR (101 MHz, $CDCl_3$): δ (ppm) = 169.9, 169.7, 57.4, 52.8, 52.8, 49.6, 33.2, 31.7, 17.5.

IR (thin film, cm^{-1}): 2957, 2098, 1734, 1436, 1272, 1249, 1201, 1156, 1015, 831, 558.

HRMS (ESI+): *m/z* for $C_9H_{15}N_3NaO_4$ $[M+Na]^+$: calc.: 252.0955, found: 252.0959.

TLC: R_f = 0.16 (SiO_2, 5% EtOAc in hexane).

Compound 2ad:

8-(Azidomethyl)-1,4-dioxaspiro[4.5]decane

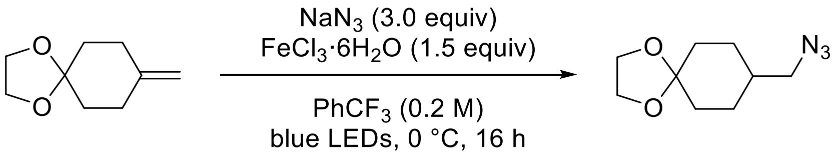

1ad **2ad**, 40%

Azide **2ad** was prepared via GP1 from alkene **1ad**[22] (30.8 mg, 0.200 mmol, 1.00 equiv) in $PhCF_3$. The crude product was purified via flash column chromatography (0-5% EtOAc in hexane) to give **2ad** as a colorless oil. The product was isolated as an inseparable >20:1 mixture of regioisomers.

Yield: 15.6 mg, 0.079 mmol, 40%, rr > 20:1

^{1}H NMR (400 MHz, $CDCl_3$): δ (ppm) = 3.93 (td, J = 2.7, 0.6 Hz, 4H), 3.16 (d, J = 6.7 Hz, 2H), 1.81 – 1.73 (m, 4H), 1.62 – 1.48 (m, 3H), 1.37 – 1.24 (m, 2H).

^{13}C NMR (101 MHz, $CDCl_3$): δ (ppm) = 108.7, 64.4, 64.4, 57.2, 36.9, 34.2, 27.8.

IR (thin film, cm^{-1}): 2933, 2879, 2097, 1448, 1269, 1146, 1108, 1035, 934.

HRMS (ESI+): m/z for $C_9H_{15}N_3NaO_2$ $[M+Na]^+$: calc.: 220.1056, found: 220.1052.

TLC: R_f = 0.44 (SiO_2, 20% EtOAc in hexane).

Compound 2ae:

Tert-butyl 4-(azidomethyl)piperidine-1-carboxylate

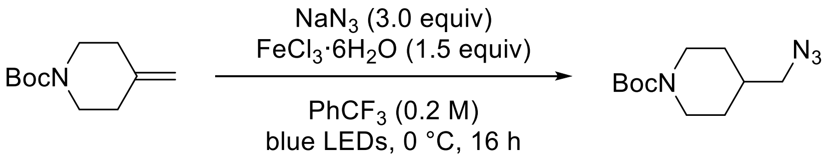

1ae **2ae**, 75%

Azide **2ae** was prepared via GP1 from alkene **1ae**[23] (39.5 mg, 0.200 mmol, 1.00 equiv) in $PhCF_3$. The crude product was purified via flash column chromatography (0-5% EtOAc in hexane) to give **2ae** as a colorless oil. The corresponding regioisomer could not be identified in the ^1H NMR spectrum of the unpurified reaction mixture.

Yield: 35.9 mg, 0.149 mmol, 75%

^{1}H NMR (400 MHz, $CDCl_3$): δ (ppm) = 4.13 (d, J = 13.3 Hz, 2H), 3.19 (d, J = 6.2 Hz, 2H), 2.69 (t, J = 12.8 Hz, 2H), 1.75 – 1.66 (m, 3H), 1.45 (s, 9H), 1.22 – 1.08 (m, 2H).

^{13}C NMR (101 MHz, $CDCl_3$): δ (ppm) = 154.9, 79.6, 57.2, 43.6, 36.7, 29.7, 28.6.

IR (thin film, cm^{-1}): 2976, 2930, 2856, 2098, 1689, 1420, 1365, 1277, 1244, 1169, 1137, 971, 865, 770.

HRMS (ESI+): *m/z* for $C_{11}H_{20}N_4NaO_2$ $[M+Na]^+$: calc.: 263.1478, found: 263.1477.

TLC: R_f = 0.50 (SiO_2, 20% EtOAc in hexane).

Compound 2af:

(3r,5r,7r)-1-(1-Azidopropan-2-yl)adamantane

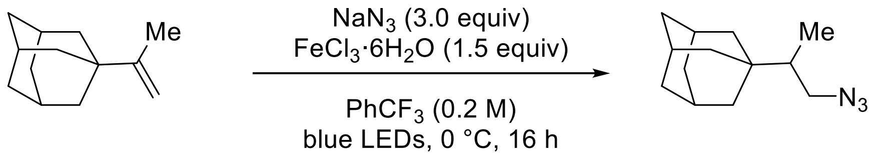

1a **2af**, 77%

Azide **2af** was prepared via GP1 from alkene **1af**[24] (35.3 mg, 0.200 mmol, 1.00 equiv) in $PhCF_3$. The crude product was purified via flash column chromatography (100% pentane) to give **2af** as a colorless oil. The corresponding regioisomer could not be identified in the ^1H NMR spectrum of the unpurified reaction mixture.

Yield: 33.7 mg, 0.154 mmol, 77%

^{1}H NMR (400 MHz, $CDCl_3$): δ (ppm) = 3.56 (dd, *J* = 11.9, 3.8 Hz, 1H), 2.93 (dd, *J* = 11.9, 9.6 Hz, 1H), 2.01 – 1.93 (m, 3H), 1.75 – 1.67 (m, 3H), 1.66 – 1.59 (m, 3H), 1.58 – 1.45 (m, 6H), 1.29 (dtd, *J* = 13.7, 6.9, 3.7 Hz, 1H), 0.97 – 0.89 (m, 3H).

^{13}C NMR (101 MHz, $CDCl_3$): δ (ppm) = 54.0, 43.5, 39.7, 37.3, 34.3, 28.7, 12.0.

IR (thin film, cm^{-1}): 2901, 2848, 2092, 1448, 1278.

HRMS (EI): *m/z* for $C_{13}H_{20}N$ $[M–HN_2]^+$: calc.: 190.1590, found: 190.1588.

TLC: R_f = 0.67 (SiO_2, 2% EtOAc in hexane).

Compound 2ag:

4-Azidooctane

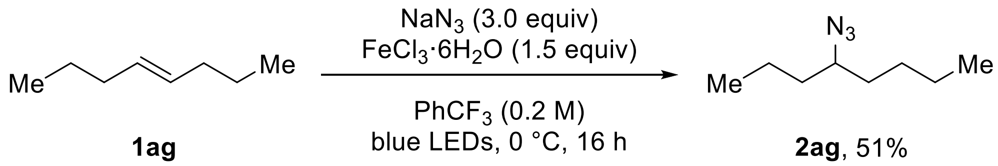

1ag

2ag, 51%

Azide **2ag** was prepared via GP1 from alkene **1ag** (31.3 μL, 22.4 mg, 0.200 mmol, 1.00 equiv) in $PhCF_3$. The crude product was purified via flash column chromatography (100% pentane) to give **2ag** as a colorless oil.

Note: Due to potential volatility of the product, the solvent was evaporated at 600 mbar at 35 °C.

Yield: 15.9 mg, 0.102 mmol, 51%

^{1}H NMR yield: 72%

^{1}H NMR (400 MHz, $CDCl_3$): δ (ppm) = 3.24 (p, J = 6.4 Hz, 1H), 1.54 – 1.29 (m, 10H), 0.97 – 0.87 (m, 6H).

^{13}C NMR (101 MHz, $CDCl_3$): δ (ppm) = 63.0, 36.7, 34.3, 28.4, 22.7, 19.5, 14.1, 14.0.

IR (thin film, cm^{-1}): 2960, 2934, 2874, 2094, 1466, 1273, 1253.

HRMS (EI): *m/z* for $C_8H_{16}N$ $[M–HN_2]^+$: calc.: 126.1277, found: 126.1277.

TLC: R_f = 0.54 (SiO_2, 2% EtOAc in hexane).

Compound 2ah:

Azidocyclohexane

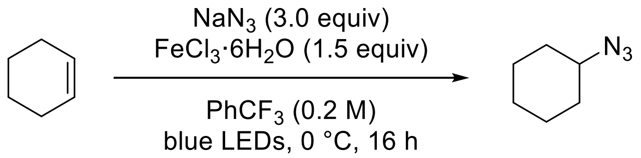

1ah **2ah**, 83%

Azide **2ah** was prepared via GP1 from alkene **1ah** (20.2 μL, 16.4 mg, 0.200 mmol, 1.00 equiv) in $PhCF_3$. Due to the volatility of the product, **2ah** was not isolated and the yield was determined via ^1H NMR with mesitylene as internal standard. Azide **2ah** is a known compound and the characterization data of the unpurified reaction mixture matched with the literature.[25] To verify the structural assignment, a one-pot derivatization procedure was devised (*vide infra*).

^{1}H NMR yield: 83%

Compound 2ai:

Azidocyclooctane

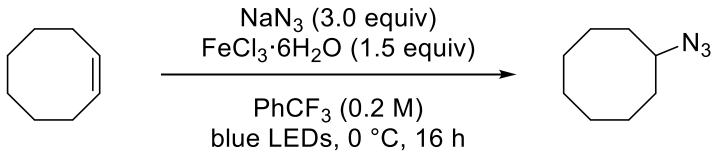

1ai **2ai**, 57%

Azide **2ai** was prepared via GP1 from alkene **1ai** (27.5 µL, 22.0 mg, 0.200 mmol, 1.00 equiv) in $PhCF_3$. The crude product was purified via flash column chromatography (100% pentane) to give **2ai** as a colorless oil.

Note: Due to potential volatility of the product, the solvent was evaporated at 600 mbar at 35 °C.

Yield: 17.6 mg, 0.115 mmol, 57%

^{1}H NMR yield: 76%

^{1}H NMR (400 MHz, $CDCl_3$): δ (ppm) = 3.56 (tt, *J* = 8.4, 4.0 Hz, 1H), 1.92 – 1.82 (m, 2H), 1.78 – 1.66 (m, 4H), 1.61 – 1.45 (m, 8H).

^{13}C NMR (101 MHz, $CDCl_3$): δ (ppm) = 62.4, 31.0, 27.4, 25.3, 23.3.

IR (thin film, cm^{-1}): 2924, 2855, 2096, 1251, 720.

HRMS (EI): *m/z* for $C_8H_{14}N$ $[M–HN_2]^+$: calc.: 124.1121, found: 124.1121.

TLC: R_f = 0.46 (SiO_2, 2% EtOAc in hexane).

Compound 2aj:

Azidododecane

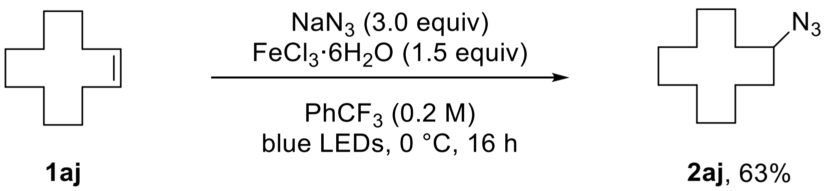

Azide **2aj** was prepared via GP1 from alkene **1aj** (38.6 µL, 33.3 mg, 0.200 mmol, 1.00 equiv) in $PhCF_3$. The crude product was purified via flash column chromatography (100% pentane) to give **2aj** as a colorless oil.

Yield: 26.3 mg, 0.126 mmol, 63%

^{1}H NMR (400 MHz, $CDCl_3$): δ (ppm) = 3.49 (tt, J = 7.4, 4.8 Hz, 1H), 1.75 – 1.62 (m, 2H), 1.58 – 1.25 (m, 20H).

^{13}C NMR (101 MHz, $CDCl_3$): δ (ppm) = 59.4, 29.2, 24.1, 23.9, 23.5, 23.4, 21.5.

IR (thin film, cm^{-1}): 2932, 2864, 2087, 1470, 1446, 1249, 719.

HRMS (EI): *m/z* for $C_{12}H_{22}N$ [M–HN_2]$^+$: calc.: 180.1747, found: 180.1746.

TLC: R_f = 0.55 (SiO_2, 2% EtOAc in hexane).

Compound 2ak:

(±)-(1R,2R,4S)-2-Azidobicyclo[2.2.1]heptane

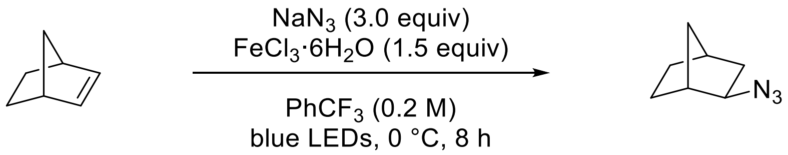

1ak **2ak**, 80%

Azide **2ak** was prepared via GP1 from alkene **1ak** (18.8 mg, 0.200 mmol, 1.00 equiv) in $PhCF_3$. The crude product was purified via flash column chromatography (100% pentane) to give **2ak** as a colorless oil. The product was isolated as single diastereomer.

Note: Due to potential volatility of the product, the solvent was evaporated at 600 mbar at 35 °C.

Note: The obtained exo selectivity was determined by comparison with the literature.[26]

Yield: 21.9 mg, 0.160 mmol, 80%

^{1}H NMR (400 MHz, $CDCl_3$): δ (ppm) = 3.50 – 3.44 (m, 1H), 2.33 – 2.27 (m, 2H), 1.67 – 1.39 (m, 5H), 1.22 – 1.15 (m, 1H), 1.15 – 1.05 (m, 2H).

^{13}C NMR (101 MHz, $CDCl_3$): δ (ppm) = 64.5, 42.0, 38.3, 35.9, 35.4, 28.5, 26.0.

IR (thin film, cm^{-1}): 2955, 2926, 2873, 2106, 1457, 1378, 1248, 971, 737.

HRMS (EI): *m/z* for $C_7H_{10}N$ $[M–HN_2]^+$: calc.: 108.0808, found: 108.0806.

TLC: R_f = 0.54 (SiO_2, 2% EtOAc in hexane).

Compound 2al:

(±)-(3aR,4S,5R,7S,7aR)-2-Azido-2,3,3a,4,7,7a-hexahydro-1H-4,7-methanoindene

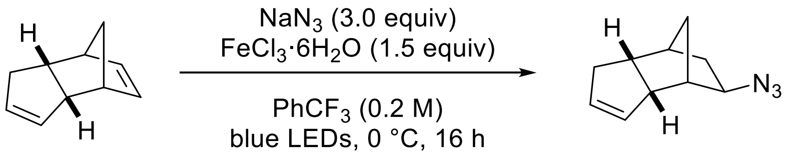

1al **2al**, 85%

Azide **2al** was prepared via GP1 from alkene **1al** (26.9 µL, 26.4 mg, 0.200 mmol, 1.00 equiv) in $PhCF_3$. The crude product was purified via flash column chromatography (100% hexane) to give **2al** as a colorless oil. The product was isolated as an inseparable 1.4:1 mixture of regioisomers.

Note: The obtained exo selectivity for both regioisomers was determined via X-ray crystallographic analysis after one-pot diversification to the corresponding triazole (*vide infra*).

Yield: 29,8 mg, 0.170 mmol, 85%, rr = 1.4:1

^{1}H NMR (400 MHz, $CDCl_3$): δ (ppm) = 5.68 – 5.62 (m, 2H), 5.58 – 5.54 (m, 1H), 5.52 – 5.48 (m, 1H), 3.52 (dq, *J* = 7.6, 1.4 Hz, 1H), 3.45 (ddd, *J* = 7.8, 3.7, 1.5 Hz, 1H), 3.15 – 3.07 (m, 1H), 3.05 – 2.98 (m, 1H), 2.64 – 2.46 (m, 2H), 2.43 – 2.35 (m, 2H), 2.33 – 2.17 (m, 4H), 2.11 (p, *J* = 2.3 Hz, 1H), 2.08 – 2.04 (m, 1H), 1.81 (ddd, *J* = 13.0, 7.8, 2.7 Hz, 1H), 1.74 – 1.64 (m, 2H), 1.61 (dt, *J* = 9.9, 1.8 Hz, 1H), 1.47 – 1.40 (m, 2H), 1.32 – 1.18 (m, 2H).

^{13}C NMR (101 MHz, $CDCl_3$): δ (ppm) = 132.5, 131.7, 131.4, 130.7, 61.4, 59.0, 51.8, 51.7, 46.8, 45.0, 41.2, 41.1, 40.5, 39.7, 38.6, 38.2, 34.3, 32.6, 31.7, 30.9.

IR (thin film, cm^{-1}): 3047, 2955, 2090, 1454, 1342, 1248, 1025, 967, 946, 738, 698, 558.

HRMS (EI): *m/z* for $C_{10}H_{12}N$ $[M–HN_2]^+$: calc.: 146.0964, found: 146.0964.

TLC: R_f = 0.34 (SiO_2, 100% hexane).

Compound 2am:

(4-Azido-3-methylpentyl)benzene

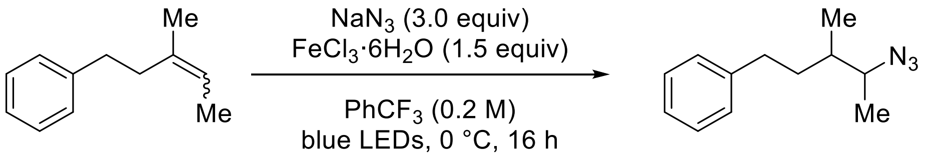

1am **2am**, 63%

Azide **2am** was prepared via GP1 from alkene **1am**[27] (32.1 mg, 0.200 mmol, 1.00 equiv) in $PhCF_3$. The crude product was purified via flash column chromatography (0-2% EtOAc in hexane) to give **2am** as a colorless oil. The product was isolated as an inseparable 2:1 mixture of regioisomers.

Yield: 25.7 mg, 0.126 mmol, 63%, dr = 1:1, rr = 2:1

^{1}H NMR (400 MHz, $CDCl_3$): δ (ppm) = 7.32 – 7.27 (m, 4H), 7.23 – 7.16 (m, 6H), 3.51 (qd, J = 6.7, 4.3 Hz, 1H), 3.43 (qd, J = 6.7, 5.5 Hz, 1H), 2.76 – 2.49 (m, 4H), 1.84 – 1.73 (m, 3H), 1.62 – 1.53 (m, 2H), 1.52 – 1.44 (m, 1H), 1.24 (d, J = 6.7 Hz, 3H), 1.21 (d, J = 6.7 Hz, 3H), 0.99 – 0.97 (m, 6H).

^{13}C NMR (101 MHz, $CDCl_3$): δ (ppm) = 128.6, 128.5, 128.5, 128.5, 126.1, 126.0, 126.0, 62.5, 62.2, 37.7, 35.2, 34.9, 33.5, 30.6, 16.5, 15.5, 15.3, 14.9, 8.5.

IR (thin film, cm^{-1}): 3028, 2971, 2935, 2094, 1497, 1455, 1382, 1256, 747, 699.

HRMS (ESI+): *m/z* for $C_{12}H_{16}N$ $[M–HN_2]^+$: calc.: 174.1277, found: 174.1276.

TLC: R_f = 0.60 (SiO_2, 5% EtOAc in hexane).

Compound 2an:

Ethyl 5-azido-4-methylhexanoate

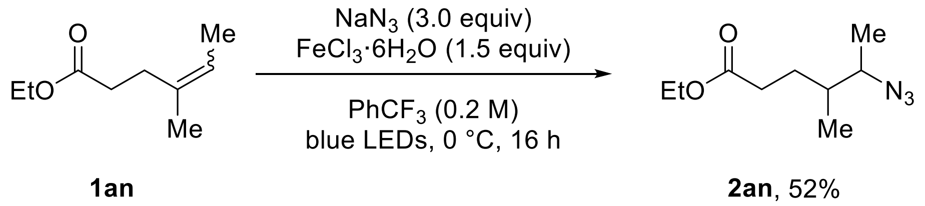

Azide **2an** was prepared via GP1 from alkene **1an**[28] (31.2 mg, 0.200 mmol, 1.00 equiv) in $PhCF_3$. The crude product was purified via flash column chromatography (0-4% EtOAc in hexane) to give **2an** as a colorless oil. The product was isolated as an inseparable 2:1 mixture of regioisomers.

Yield: 20.6 mg, 0.103 mmol, 52%, dr = 1:1, rr = 2:1

^{1}H NMR (400 MHz, $CDCl_3$): δ (ppm) = 4.16 – 4.10 (m, 4H), 3.47 (qd, *J* = 6.7, 4.4 Hz, 1H), 3.44 – 3.37 (m, 1H), 2.35 – 2.22 (m, 3H), 1.80 – 1.74 (m, 1H), 1.53 – 1.38 (m, 3H), 1.28 – 1.22 (m, 15H), 0.91 (d, *J* = 6.6 Hz, 6H).

^{13}C NMR (101 MHz, $CDCl_3$): δ (ppm) = 173.6, 173.6, 62.3, 61.9, 60.5, 60.5, 37.9, 37.7, 32.2, 32.2, 29.4, 28.0, 16.4, 15.6, 15.2, 14.6, 14.4, 14.3.

IR (thin film, cm^{-1}): 2975, 2937, 2880, 2094, 1734, 1463, 1378, 1256, 1180, 1032, 856, 783, 561.

HRMS (ESI+): *m/z* for $C_9H_{17}N_3NaO_2$ $[M+Na]^+$: calc.: 222.1213, found: 222.1213.

TLC: R_f = 0.33 (SiO_2, 5% EtOAc in hexane).

Compound 2ao:

2-Azido-2,3-dimethylbutane

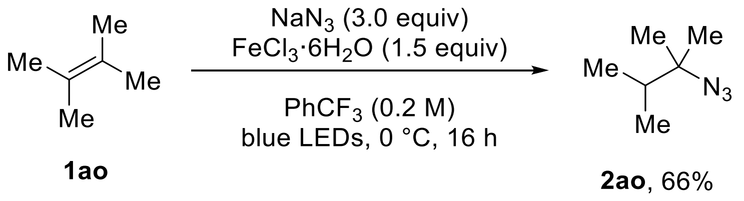

Azide **2ao** was prepared via GP1 from alkene **1ao** (23.7 µL, 16.8 mg, 0.200 mmol, 1.00 equiv) in $PhCF_3$. Due to the volatility of the product, **2ao** was not isolated and the yield was determined via ^1H NMR with mesitylene as internal standard. Azide **2ao** is a known compound and the characterization data of the unpurified reaction mixture matched with the literature.[29] To verify the structural assignment, a one-pot derivatization procedure was devised (*vide infra*).

^1H NMR yield: 66%

Compound 2ap:

(±)-Dimethyl (1S,2S)-4-azido-4,5-dimethylcyclohexane-1,2-dicarboxylate

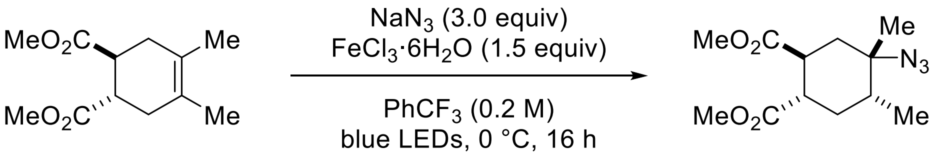

1ap **2ap**, 45%

Azide **2ap** was prepared via GP1 from alkene **1ap**[30] (45.3 mg, 0.200 mmol, 1.00 equiv) in $PhCF_3$. The crude product was purified via flash column chromatography (0-4% EtOAc in hexane) to give **2ap** as a colorless oil. The product was isolated as an inseparable 19:1 mixture of diastereomers.

Yield: 24.1 mg, 0.089 mmol, 45% dr = 19:1

^{1}H NMR (400 MHz, $CDCl_3$): δ (ppm) = 3.68 (s, 3H), 3.68 (s, 3H), 2.98 (ddd, *J* = 13.1, 11.5, 3.8 Hz, 1H), 2.69 – 2.58 (m, 1H), 2.17 (dd, *J* = 14.0, 3.8 Hz, 1H), 1.83 – 1.72 (m, 1H), 1.55 – 1.43 (m, 3H), 1.37 (s, 3H), 0.95 (d, *J* = 6.3 Hz, 3H).

^{13}C NMR (101 MHz, $CDCl_3$): δ (ppm) = 175.0, 174.6, 62.8, 52.2, 52.1, 44.7, 40.9, 40.4, 40.0, 32.7, 23.8, 15.4.

IR (thin film, cm^{-1}): 2954, 2106, 1737, 1436, 1299, 1256, 1197, 1174, 1112, 1008.

HRMS (ESI+): *m/z* for $C_{12}H_{19}N_3NaO_4$ $[M+Na]^+$: calc.: 292.1268, found: 292.1262.

TLC: R_f = 0.18 (SiO_2, 10% EtOAc in hexane).

Compound 4a:

5-Azidopentyl 2-(4-(4-chlorobenzoyl)phenoxy)-2-methylpropanoate

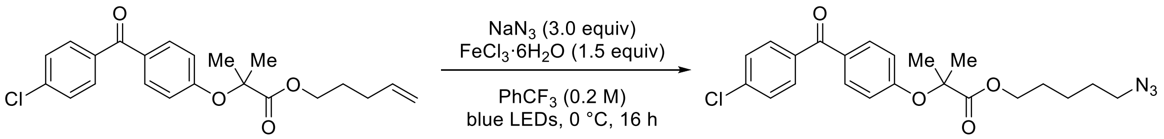

Azide **4a** was prepared via GP1 from alkene **3a** (77.4 mg, 0.200 mmol, 1.00 equiv) in $PhCF_3$. The crude product was purified via flash column chromatography (0-5% EtOAc in hexane) to give **4a** as a colorless oil. The product was isolated as an inseparable >20:1 mixture of regioisomers.

Yield: 54.4 mg, 0.127 mmol, 63%, rr > 20:1

^{1}H NMR (400 MHz, $CDCl_3$): δ (ppm) = 7.78 – 7.66 (m, 4H), 7.48 – 7.41 (m, 2H), 6.90 – 6.82 (m, 2H), 4.16 (t, J = 6.5 Hz, 2H), 3.18 (t, J = 6.8 Hz, 2H), 1.68 (s, 6H), 1.66 – 1.48 (m, 4H), 1.37 – 1.23 (m, 2H).

^{13}C NMR (101 MHz, $CDCl_3$): δ (ppm) = 194.3, 173.9, 159.8, 138.5, 136.5, 132.1, 131.3, 130.5, 128.7, 117.3, 79.5, 65.5, 51.3, 28.5, 28.1, 25.6, 23.2.

IR (thin film, cm^{-1}): 2942, 2867, 2094, 1732, 1654, 1598, 1506, 1284, 1249, 1172, 1140, 1089, 1015, 927, 853, 762.

HRMS (ESI+): *m/z* for $C_{22}H_{24}ClN_3NaO_4$ $[M+Na]^+$: calc.: 452.1348, found: 452.1347.

TLC: R_f = 0.41 (SiO_2, 20% EtOAc in hexane).

Compound 4b:

1-(6-Azidohexyl)-3,7-dimethyl-3,7-dihydro-1H-purine-2,6-dione

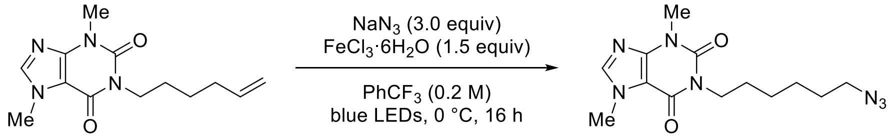

3b
from Theobromine

4b, 68%

Azide **4b** was prepared via GP1 from alkene **3b** (52.2 mg, 0.200 mmol, 1.00 equiv) in $PhCF_3$. The crude product was purified via flash column chromatography (50-100% EtOAc in hexane) to give **4b** as a colorless oil. The product was isolated as an inseparable 13:1 mixture of regioisomers.

Yield: 41.6 mg, 0.136 mmol, 68%, rr = 13:1

^{1}H NMR (400 MHz, $CDCl_3$): δ (ppm) = 7.48 (s, 1H), 4.04 – 3.92 (m, 5H), 3.55 (s, 3H), 3.23 (t, *J* = 7.0 Hz, 2H), 1.71 – 1.55 (m, 4H), 1.46 – 1.33 (m, 4H).

^{13}C NMR (101 MHz, $CDCl_3$): δ (ppm) = 155.4, 151.6, 148.9, 141.5, 107.8, 51.5, 41.3, 33.7, 29.8, 28.8, 28.0, 26.6, 26.5.

IR (thin film, cm^{-1}): 3112, 2939, 2861, 2094, 1699, 1651, 1549, 1456, 1357, 1234, 1056, 763, 750, 613.

HRMS (ESI+): *m/z* for $C_{13}H_{19}N_7NaO_2$ $[M+Na]^+$: calc.: 328.1492, found: 328.1485.

TLC: R_f = 0.20 (SiO_2, 60% EtOAc in hexane).

Compound 4c:

6-Azidohexyl 4-(N,N-dipropylsulfamoyl)benzoate

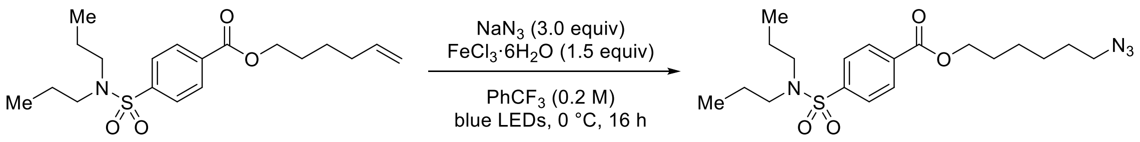

3c
from Probenecid

4c, 77%

Azide **4c** was prepared via GP1 from alkene **3c** (73.5 mg, 0.200 mmol, 1.00 equiv) in $PhCF_3$. The crude product was purified via flash column chromatography (0-5% EtOAc in hexane) to give **4c** as a colorless oil. The product was isolated as an inseparable 19:1 mixture of regioisomers.

Yield: 62.9 mg, 0.153 mmol, 77%, rr = 19:1

^{1}H NMR (400 MHz, $CDCl_3$): δ (ppm) = 8.18 – 8.10 (m, 2H), 7.89 – 7.84 (m, 2H), 4.35 (t, J = 6.6 Hz, 2H), 3.28 (t, J = 6.8 Hz, 2H), 3.12 – 3.05 (m, 4H), 1.79 (tt, J = 8.5, 6.6 Hz, 2H), 1.67 – 1.59 (m, 2H), 1.58 – 1.50 (m, 4H), 1.50 – 1.43 (m, 4H), 0.86 (t, J = 7.4 Hz, 6H).

^{13}C NMR (101 MHz, $CDCl_3$): δ (ppm) = 165.4, 144.3, 133.8, 130.3, 127.1, 65.6, 51.4, 50.0, 28.9, 28.6, 26.5, 25.7, 22.0, 11.3.

IR (thin film, cm^{-1}): 2965, 2936, 2875, 2094, 1721, 1466, 1343, 1271, 1158, 1106, 1087, 992, 863, 741, 694, 602, 561.

HRMS (ESI+): *m/z* for $C_{19}H_{31}N_4O_4S$ $[M+H]^+$: calc.: 411.2061, found: 411.2052.

TLC: R_f = 0.44 (SiO_2, 20% EtOAc in hexane).

Compound 4d:

6-Azidohexyl 3-(4,5-diphenyloxazol-2-yl)propanoate

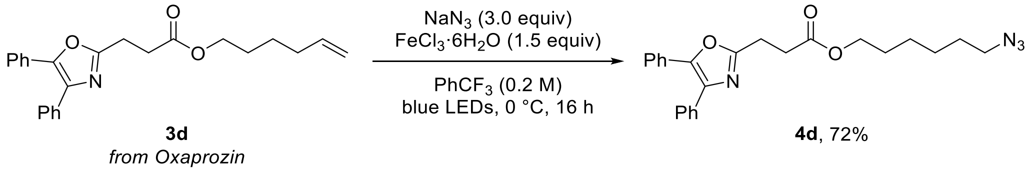

Azide **4d** was prepared via GP1 from alkene **3d** (75.1 mg, 0.200 mmol, 1.00 equiv) in $PhCF_3$. The crude product was purified via flash column chromatography (0-5% EtOAc in hexane) to give **4d** as a colorless oil. The product was isolated as an inseparable >20:1 mixture of regioisomers.

Yield: 60.1 mg, 0.144 mmol, 72%, rr > 20:1

^{1}H NMR (400 MHz, $CDCl_3$): δ (ppm) = 7.66 – 7.60 (m, 2H), 7.60 – 7.54 (m, 2H), 7.39 – 7.29 (m, 6H), 4.13 (t, *J* = 6.6 Hz, 2H), 3.25 – 3.15 (m, 4H), 2.95 – 2.87 (m, 2H), 1.68 – 1.60 (m, 2H), 1.56 (t, *J* = 6.6 Hz, 2H), 1.40 – 1.33 (m, 4H).

^{13}C NMR (101 MHz, $CDCl_3$): δ (ppm) = 172.2, 161.9, 145.5, 135.3, 132.6, 129.1, 128.8, 128.7, 128.6, 128.2, 128.0, 126.6, 64.8, 51.5, 31.3, 28.8, 28.6, 26.5, 25.6, 23.7.

IR (thin film, cm^{-1}): 2936, 2861, 2095, 1735, 1604, 1571, 1445, 1222, 1169, 1058, 1025, 962, 764, 694, 674.

HRMS (ESI+): *m/z* for $C_{24}H_{27}N_4O_3$ $[M+H]^+$: calc.: 419.2078, found: 419.2074.

TLC: R_f = 0.29 (SiO_2, 20% EtOAc in hexane).

Compound 4e:

7-((6-Azidohexyl)oxy)-4-methyl-2H-chromen-2-one

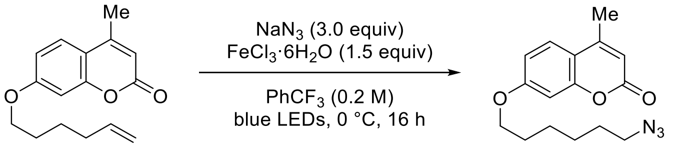

3e
from 4-Methylumbelliferone

4e, 64%

Azide **4e** was prepared via GP1 from alkene **3e** (51.7 mg, 0.200 mmol, 1.00 equiv) in $PhCF_3$. The crude product was purified via flash column chromatography (0-2% EtOAc in hexane) to give **4e** as a colorless oil. The product was isolated as an inseparable 14:1 mixture of regioisomers.

Yield: 38.5 mg, 0.128 mmol, 64%, rr = 14:1

^{1}H NMR (400 MHz, $CDCl_3$): δ (ppm) = 7.48 (d, J = 8.8 Hz, 1H), 6.84 (dd, J = 8.8, 2.5 Hz, 1H), 6.79 (d, J = 2.5 Hz, 1H), 6.12 (q, J = 1.2 Hz, 1H), 4.01 (t, J = 6.4 Hz, 2H), 3.29 (t, J = 6.9 Hz, 2H), 2.39 (d, J = 1.2 Hz, 3H), 1.82 (dt, J = 8.1, 6.3 Hz, 2H), 1.69 – 1.60 (m, 2H), 1.56 – 1.43 (m, 4H).

^{13}C NMR (101 MHz, $CDCl_3$): δ (ppm) = 162.3, 161.5, 155.4, 152.7, 125.6, 113.6, 112.8, 112.0, 101.5, 68.5, 51.5, 29.0, 28.9, 26.6, 25.7, 18.8.

IR (thin film, cm^{-1}): 2939, 2863, 2094, 1718, 1612, 1387, 1293, 1280, 1264, 1200, 1146, 1070, 1015, 877, 847, 814.

HRMS (ESI+): *m/z* for $C_{16}H_{19}N_3NaO_3$ $[M+Na]^+$: calc.: 324.1319, found: 324.1320.

TLC: R_f = 0.22 (SiO_2, 20% EtOAc in hexane).

Compound 4f:

5-Azidopentyl 2-(1-(4-chlorobenzoyl)-5-methoxy-2-methyl-1H-indol-3-yl)acetate

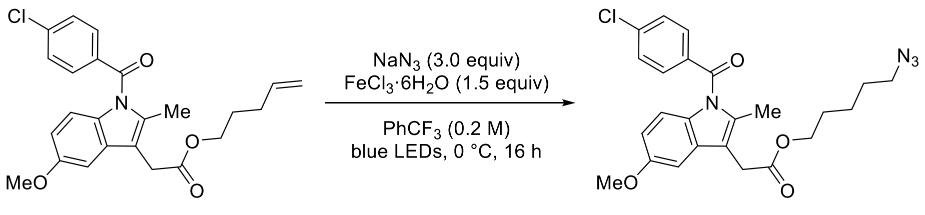

Azide **4f** was prepared via GP1 from alkene **3f** (85.2 mg, 0.200 mmol, 1.00 equiv) in $PhCF_3$. The crude product was purified via flash column chromatography (0-5% EtOAc in hexane) to give **4f** as a yellow oil. The product was isolated as an inseparable 12:1 mixture of regioisomers.

Yield: 37.7 mg, 0.080 mmol, 40%, rr = 12:1

^{1}H NMR (400 MHz, $CDCl_3$): δ (ppm) = 7.70 – 7.62 (m, 2H), 7.51 – 7.43 (m, 2H), 6.96 (d, *J* = 2.5 Hz, 1H), 6.87 (d, *J* = 9.0 Hz, 1H), 6.67 (dd, *J* = 9.0, 2.6 Hz, 1H), 4.11 (t, *J* = 6.5 Hz, 2H), 3.83 (s, 3H), 3.66 (s, 2H), 3.20 (t, *J* = 6.8 Hz, 2H), 2.39 (s, 3H), 1.70 – 1.51 (m, 4H), 1.42 – 1.33 (m, 2H).

^{13}C NMR (101 MHz, $CDCl_3$): δ (ppm) = 171.0, 168.4, 156.2, 139.4, 136.1, 134.0, 131.3, 130.9, 130.8, 129.3, 115.1, 112.8, 111.6, 101.6, 64.8, 55.8, 51.3, 30.5, 28.6, 28.3, 23.3, 13.5.

IR (thin film, cm^{-1}): 2936, 2866, 2095, 1732, 1682, 1591, 1477, 1456, 1355, 1314, 1259, 1222, 1165, 1142, 1036, 1014, 925, 833, 754, 689, 548.

HRMS (ESI+): *m/z* for $C_{24}H_{25}ClN_4NaO_4$ $[M+Na]^+$: calc.: 491.1457, found: 491.1446.

TLC: R_f = 0.27 (SiO_2, 20% EtOAc in hexane).

Compound 4g:

(±)-Tert-butyl (1R,3r,5S)-3-((6-azidohexanoyl)oxy)-8-azabicyclo[3.2.1]octane-8-carboxylate

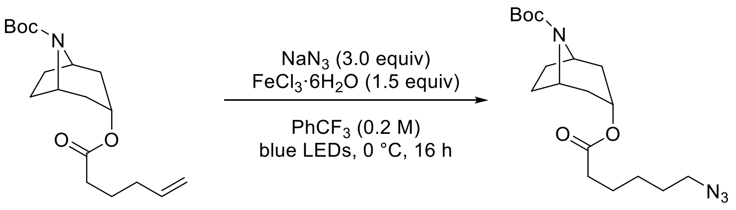

3g
from (±)-Nortropine

4g, 69%

Azide **4g** was prepared via GP1 from alkene **3g** (64.7 mg, 0.200 mmol, 1.00 equiv) in $PhCF_3$. The crude product was purified via flash column chromatography (0-2% EtOAc in hexane) to give **4g** as a colorless oil. The product was isolated as an inseparable 16:1 mixture of regioisomers.

Yield: 50.4 mg, 0.138 mmol, 69%, rr = 16:1

^{1}H NMR (400 MHz, $CDCl_3$): δ (ppm) = 5.08 (t, *J* = 5.0 Hz, 1H), 4.17 (bs, 2H), 3.27 (t, *J* = 6.8 Hz, 2H), 2.31 (t, *J* = 7.5 Hz, 2H), 2.11 (bs, 2H), 1.97 (d, *J* = 2.4 Hz, 4H), 1.74 – 1.56 (m, 6H), 1.44 (s, 9H), 1.44 – 1.37 (m, 2H).

^{13}C NMR (101 MHz, $CDCl_3$): δ (ppm) = 172.6, 153.4, 79.5, 68.0, 52.7, 52.0, 51.3, 35.9, 35.3, 34.8, 28.7, 28.6, 28.4, 27.8, 26.4, 24.5.

IR (thin film, cm^{-1}): 2973, 2944, 2868, 2094, 1731, 1691, 1392, 1365, 1327, 1318, 1246, 1180, 1161, 1099, 1078, 1031, 867, 778.

HRMS (ESI+): *m/z* for $C_{18}H_{30}N_4NaO_4$ $[M+Na]^+$: calc.: 389.2159, found: 389.2150.

TLC: R_f = 0.29 (SiO_2, 20% EtOAc in hexane).

Compound 4h:

***N*-(6-Azidohexyl)-4-(5-(p-tolyl)-3-(trifluoromethyl)-1H-pyrazol-1-yl)benzene-sulfonamide**

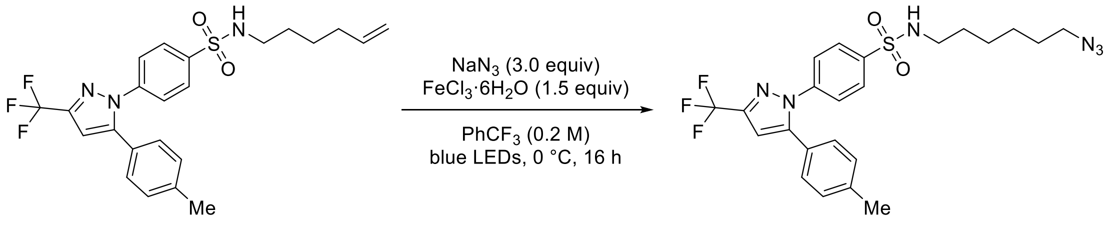

3h
from Celecoxib

4h, 77%

Azide **4h** was prepared via GP1 from alkene **3h** (92.7 mg, 0.200 mmol, 1.00 equiv) in $PhCF_3$. The crude product was purified via flash column chromatography (10-30% EtOAc in hexane) to give **4h** as a white solid. The product was isolated as an inseparable 12:1 mixture of regioisomers.

Yield: 78.5 mg, 0.155 mmol, 77%, rr = 12:1

^{1}H NMR (400 MHz, $CDCl_3$): δ (ppm) = 7.84 (d, *J* = 8.9 Hz, 2H), 7.47 (d, *J* = 9.0 Hz, 2H), 7.17 (d, *J* = 7.9 Hz, 2H), 7.10 (d, *J* = 8.4 Hz, 2H), 6.74 (s, 1H), 4.58 (t, *J* = 6.1 Hz, 1H), 3.24 (t, *J* = 6.8 Hz, 2H), 3.02 – 2.87 (m, 2H), 2.38 (s, 3H), 1.59 – 1.41 (m, 4H), 1.39 – 1.25 (m, 4H)

^{13}C NMR (101 MHz, $CDCl_3$): δ (ppm) = 145.4, 144.3 (q, *J* = 38.7 Hz), 142.7, 140.0, 139.6, 129.9, 128.8, 128.2, 125.8, 125.7, 121.2 (q, *J* = 269.3 Hz), 106.4 (q, *J* = 1.6 Hz), 51.4, 43.3, 29.6, 28.8, 26.3, 26.2, 21.5.

^{19}F NMR (376 MHz, $CDCl_3$): δ (ppm) = -62.45.

IR (thin film, cm^{-1}): 3286, 2938, 2863, 2096, 1598, 1499, 1472, 1236, 1159, 1134, 1097, 976, 843, 809, 760, 627, 616.

HRMS (ESI+): *m/z* for $C_{23}H_{26}F_3N_6O_2S$ $[M+H]^+$: calc.: 507.1785, found: 507.1782.

TLC: R_f = 0.29 (SiO_2, 20% EtOAc in hexane).

Compound 4i:

6-Azidohexyl (4R)-4-((3R,8R,9S,10S,13R,14S,17R)-3-acetoxy-10,13-dimethyl-hexadecahydro-1H-cyclopenta[a]phenanthren-17-yl)pentanoate

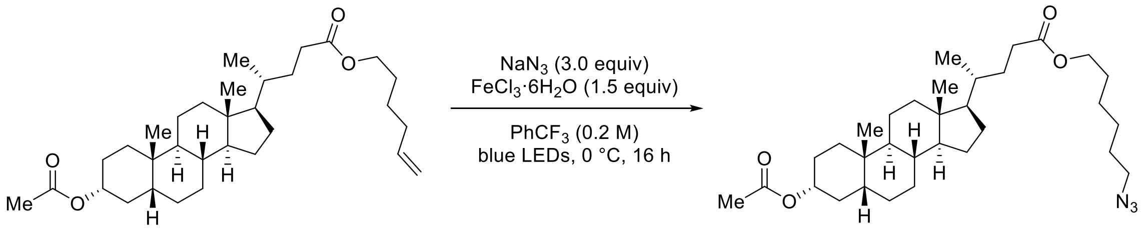

3i
from Lithocholic acid

4i, 58%

Azide **4i** was prepared via GP1 from alkene **3i** (100.2 mg, 0.200 mmol, 1.00 equiv) in $PhCF_3$. The crude product was purified via flash column chromatography (0-2% EtOAc in hexane) to give **4i** as a white solid. The corresponding regioisomer could not be identified in the ^1H NMR spectrum of the unpurified reaction mixture.

Yield: 63.4 mg, 0.117 mmol, 58%

^{1}H NMR (400 MHz, $CDCl_3$): δ (ppm) = 4.71 (tt, *J* = 11.4, 4.7 Hz, 1H), 4.05 (t, *J* = 6.6 Hz, 2H), 3.26 (t, *J* = 6.9 Hz, 2H), 2.33 (ddd, *J* = 15.2, 10.0, 5.2 Hz, 1H), 2.20 (ddd, *J* = 15.3, 9.5, 6.7 Hz, 1H), 2.02 (s, 3H), 1.95 (dt, *J* = 12.2, 2.9 Hz, 1H), 1.90 – 1.73 (m, 5H), 1.72 – 1.48 (m, 8H), 1.48 – 1.16 (m, 15H), 1.15 – 0.96 (m, 5H), 0.93 – 0.88 (m, 6H), 0.63 (s, 3H).

^{13}C NMR (101 MHz, $CDCl_3$): δ (ppm) = 174.5, 170.8, 74.5, 64.2, 56.6, 56.2, 51.5, 42.9, 42.0, 40.5, 40.3, 35.9, 35.5, 35.2, 34.7, 32.4, 31.4, 31.2, 28.9, 28.7, 28.3, 27.2, 26.8, 26.5, 26.5, 25.7, 24.3, 23.5, 21.6, 21.0, 18.4, 12.2.

IR (thin film, cm^{-1}): 2936, 2866, 2095, 1735, 1450, 1379, 1362, 1241, 1167, 1027, 616.

HRMS (ESI+): *m/z* for $C_{32}H_{53}N_3NaO_4$ $[M+Na]^+$: calc.: 566.3928, found: 566.3913.

$[\alpha]_D^{28}$ = +31.3 (c = 1.0, $CHCl_3$).

TLC: R_f = 0.60 (SiO_2, 20% EtOAc in hexane).

Compound 4j:

5-Azidopentyl 3-methyl-4-oxo-2-phenyl-4H-chromene-8-carboxylate

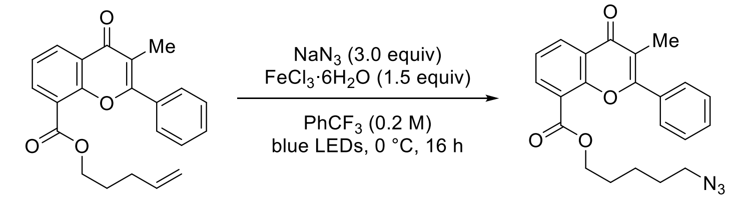

3j *from a Flavone-derivative* **4j**, 80%

Azide **4j** was prepared via GP1 from alkene **3j** (49.7 mg, 0.200 mmol, 1.00 equiv) in $PhCF_3$. The crude product was purified via flash column chromatography (0-5% EtOAc in hexane) to give **4j** as a colorless oil. The product was isolated as an inseparable 19:1 mixture of regioisomers.

Yield: 62.6 mg, 0.160 mmol, 80%, rr = 19:1

^{1}H NMR (400 MHz, $CDCl_3$): δ (ppm) = 8.46 (dd, *J* = 7.9, 1.8 Hz, 1H), 8.25 (dd, *J* = 7.5, 1.8 Hz, 1H), 7.81 – 7.74 (m, 2H), 7.55 – 7.50 (m, 3H), 7.44 (t, *J* = 7.7 Hz, 1H), 4.35 (t, *J* = 6.6 Hz, 2H), 3.18 (t, *J* = 6.8 Hz, 2H), 2.22 (s, 3H), 1.79 – 1.69 (m, 2H), 1.60 – 1.50 (m, 2H), 1.45 – 1.35 (m, 2H).

^{13}C NMR (101 MHz, $CDCl_3$): δ (ppm) = 178.3, 164.7, 161.1, 154.5, 136.2, 133.2, 130.9, 130.6, 129.4, 128.5, 124.1, 123.4, 120.8, 117.8, 65.4, 51.2, 28.6, 28.3, 23.4, 11.9.

IR (thin film, cm^{-1}): 2944, 2866, 2093, 1725, 1708, 1636, 1478, 1439, 1391, 1372, 1283, 1262, 1178, 1126, 1068, 1023, 759, 699.

HRMS (ESI+): *m/z* for $C_{22}H_{22}N_3O_4$ $[M+H]^+$: calc.: 392.1605, found: 392.1600.

TLC: R_f = 0.22 (SiO_2, 20% EtOAc in hexane).

Compound 4k:

(*S*)-1-Ethoxy-1-oxopropan-2-yl 5-azidopentanoate

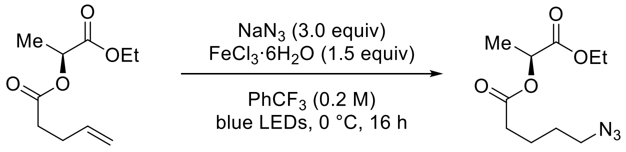

3k
from Ethyl (–)-lactate

4k, 79%

Azide **4k** was prepared via GP1 from alkene **3k** (40.0 mg, 0.200 mmol, 1.00 equiv) in $PhCF_3$. The crude product was purified via flash column chromatography (0-5% EtOAc in hexane) to give **4k** as a colorless oil. The product was isolated as an inseparable >20:1 mixture of regioisomers.

Yield: 38.5 mg, 0.158 mmol, 79%, rr > 20:1

^{1}H NMR (400 MHz, $CDCl_3$): δ (ppm) = 5.06 (q, *J* = 7.1 Hz, 1H), 4.19 (q, *J* = 7.1 Hz, 2H), 3.29 (t, *J* = 6.6 Hz, 2H), 2.51 – 2.33 (m, 2H), 1.80 – 1.58 (m, 4H), 1.47 (d, *J* = 7.1 Hz, 3H), 1.26 (t, *J* = 7.1 Hz, 3H).

^{13}C NMR (101 MHz, $CDCl_3$): δ (ppm) = 172.6, 170.9, 68.7, 61.5, 51.1, 33.4, 28.3, 22.1, 17.0, 14.2.

IR (thin film, cm^{-1}): 2986, 2942, 2875, 2094, 1737, 1452, 1371, 1349, 1270, 1202, 1156, 1133, 1097, 1048, 1019, 861, 752.

HRMS (ESI+): *m/z* for $C_{10}H_{17}N_3NaO_4$ $[M+Na]^+$: calc.: 266.1111, found: 266.1104.

$[\alpha]_D^{27}$ = –29.7 (c = 1.0, $CHCl_3$).

TLC: R_f = 0.41 (SiO_2, 20% EtOAc in hexane).

Compound 4l:

Ethyl 4-((5-azidopentanoyl)oxy)benzoate

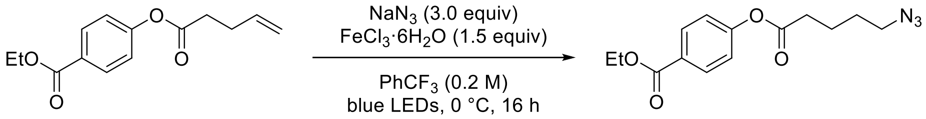

3l
from Ethylparaben

4l, 67%

Azide **4l** was prepared via GP1 from alkene **3l** (49.7 mg, 0.200 mmol, 1.00 equiv) in $PhCF_3$. The crude product was purified via flash column chromatography (0-5% EtOAc in hexane) to give **4l** as a colorless oil. The product was isolated as an inseparable 19:1 mixture of regioisomers.

Yield: 39.0 mg, 0.134 mmol, 67%, rr = 19:1

^{1}H NMR (400 MHz, $CDCl_3$): δ (ppm) = 8.07 (d, J = 9.0 Hz, 2H), 7.16 (d, J = 9.0 Hz, 2H), 4.37 (q, J = 7.2 Hz, 2H), 3.35 (t, J = 6.6 Hz, 2H), 2.62 (t, J = 7.3 Hz, 2H), 1.92 – 1.79 (m, 2H), 1.78 – 1.67 (m, 2H), 1.38 (t, J = 7.1 Hz, 3H).

^{13}C NMR (101 MHz, $CDCl_3$): δ (ppm) = 171.2, 165.9, 154.3, 131.2, 128.2, 121.6, 61.2, 51.1, 33.9, 28.4, 22.1, 14.4.

IR (thin film, cm^{-1}): 2940, 2874, 2095, 1760, 1716, 1604, 1505, 1272, 1202, 1161, 1106, 1018, 911, 862, 768, 698.

HRMS (ESI+): *m/z* for $C_{14}H_{17}N_3NaO_4$ $[M+Na]^+$: calc.: 314.1111, found: 314.1110.

TLC: R_f = 0.16 (SiO_2, 5% EtOAc in hexane).

Compound 4m:

5-Azidopentyl (2S,5R)-3,3-dimethyl-7-oxo-4-thia-1-azabicyclo[3.2.0]heptane-2-carboxylate 4,4-dioxide

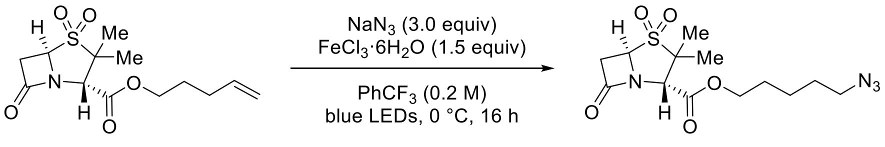

3m *from Sulbactam* **4m**, 69%

Azide **4m** was prepared via GP1 from alkene **3m** (60.3 mg, 0.200 mmol, 1.00 equiv) in $PhCF_3$. The crude product was purified via flash column chromatography (0-20% EtOAc in hexane) to give **4m** as a colorless oil. The product was isolated as an inseparable 14:1 mixture of regioisomers.

Yield: 47.2 mg, 0.137 mmol, 69%, rr = 14:1

^{1}H NMR (400 MHz, $CDCl_3$): δ (ppm) = 4.61 (dd, *J* = 4.2, 2.2 Hz, 1H), 4.37 (s, 1H), 4.21 (td, *J* = 6.7, 1.4 Hz, 2H), 3.49 (dd, *J* = 16.3, 4.3 Hz, 1H), 3.43 (dd, *J* = 16.2, 2.2 Hz, 1H), 3.29 (t, *J* = 6.7 Hz, 2H), 1.77 – 1.57 (m, 7H), 1.51 – 1.42 (m, 2H), 1.41 (s, 3H).

^{13}C NMR (101 MHz, $CDCl_3$): δ (ppm) = 170.9, 167.1, 66.2, 63.4, 62.8, 61.2, 51.2, 38.4, 28.5, 28.1, 23.2, 20.5, 18.7.

IR (thin film, cm^{-1}): 2941, 2869, 2095, 1793, 1751, 1464, 1319, 1288, 1187, 1155, 1118, 1084, 949, 709, 628, 550, 521.

HRMS (ESI+): *m/z* for $C_{13}H_{20}N_4NaO_5S$ $[M+Na]^+$: calc.: 367.1047, found: 367.1041.

$[\alpha]_D^{28}$ = +143.9 (c = 1.0, $CHCl_3$).

TLC: R_f = 0.58 (SiO_2, 50% EtOAc in hexane).

Compound 4n:

(±)- (1S,3R,4R)-3-(azidomethyl)-2,2-dimethylbicyclo[2.2.1]heptane

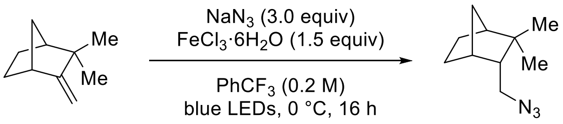

3n
from (±)-Camphene

4n, 72%

Azide **4n** was prepared via GP1 from alkene **3n** (27.2 mg, 0.200 mmol, 1.00 equiv) in $PhCF_3$. The crude product was purified via flash column chromatography (100% hexane) to give **4n** as a colorless oil. The corresponding regioisomer could not be identified in the ^1H NMR spectrum of the unpurified reaction mixture.

Note: The obtained endo selectivity was determined by comparison with the literature.[26]

Yield: 25.9 mg, 0.144 mmol, 72%, dr = 10:1

^{1}H NMR (400 MHz, $CDCl_3$): δ (ppm) = 3.25 (d, J = 8.2 Hz, 2H), 2.28 – 2.24 (m, 1H), 1.80 – 1.75 (m, 1H), 1.70 – 1.62 (m, 2H), 1.57 (dd, J = 12.0, 3.4 Hz, 1H), 1.37 – 1.26 (m, 3H), 1.22 (dt, J = 9.8, 1.7 Hz, 1H), 1.00 (s, 3H), 0.85 (s, 3H).

^{13}C NMR (101 MHz, $CDCl_3$): δ (ppm) = 50.1, 49.7, 49.1, 40.9, 37.2, 32.3, 24.6, 20.9, 20.4.

IR (thin film, cm^{-1}): 2955, 2879, 2087, 1464, 1366, 1264, 895, 658.

HRMS (EI): m/z for $C_{10}H_{16}N$ $[M–HN_2]^+$: calc.: 150.1277, found: 150.1277.

TLC: R_f = 0.55 (SiO_2, 2% EtOAc in hexane).

Compound 4o:

5-Azidopentyl 2-(11-oxo-6,11-dihydrodibenzo[b,e]oxepin-2-yl)acetate

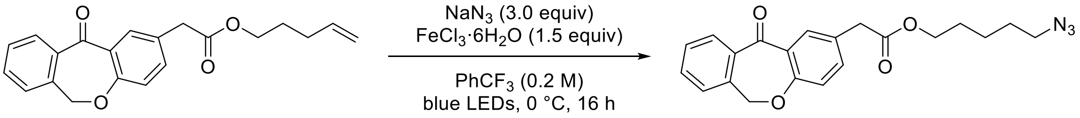

3o
from Isoxepac

4o, 43%

Azide **4o** was prepared via GP1 from alkene **3o** (67.3 mg, 0.200 mmol, 1.00 equiv) in $PhCF_3$. The crude product was purified via flash column chromatography (0-5% EtOAc in hexane) to give **4o** as a colorless oil. The product was isolated as an inseparable 12:1 mixture of regioisomers.

Yield: 32.6 mg, 0.086 mmol, 43%, rr = 12:1

^{1}H NMR (400 MHz, $CDCl_3$): δ (ppm) = 8.12 (d, *J* = 2.4 Hz, 1H), 7.89 (dd, *J* = 7.7, 1.4 Hz, 1H), 7.55 (td, *J* = 7.4, 1.4 Hz, 1H), 7.47 (td, *J* = 7.6, 1.3 Hz, 1H), 7.42 (dd, *J* = 8.4, 2.4 Hz, 1H), 7.38 – 7.34 (m, 1H), 7.03 (d, *J* = 8.4 Hz, 1H), 5.18 (s, 2H), 4.11 (t, *J* = 6.5 Hz, 2H), 3.64 (s, 2H), 3.25 (t, *J* = 6.8 Hz, 2H), 1.71 – 1.55 (m, 4H), 1.46 – 1.37 (m, 2H).

^{13}C NMR (101 MHz, $CDCl_3$): δ (ppm) 190.9, 171.5, 160.6, 140.6, 136.4, 135.7, 132.9, 132.5, 129.6, 129.4, 128.0, 127.9, 125.3, 121.2, 73.7, 64.8, 51.4, 40.4, 28.6, 28.2, 23.3.

IR (thin film, cm^{-1}): 2939, 2869, 2094, 1729, 1728, 1634, 1606, 1455, 1392, 1264, 1176, 1158, 1032, 854, 812.

HRMS (ESI+): *m/z* for $C_{21}H_{21}N_3NaO_4$ $[M+Na]^+$: calc.: 402.1424, found: 402.1419.

TLC: R_f = 0.32 (SiO_2, 20% EtOAc in hexane).

Compound 4p:

(1R,2S,5R)-2-Isopropyl-5-methylcyclohexyl 5-azidopentanoate

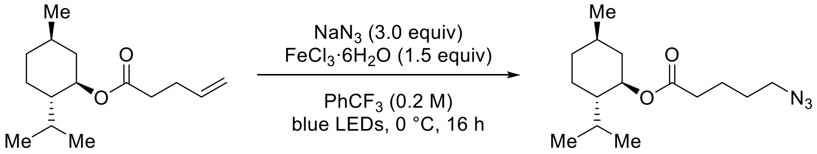

3p
from (–)-Menthol

4p, 86%

Azide **4p** was prepared via GP1 from alkene **3p** (47.7 mg, 0.200 mmol, 1.00 equiv) in $PhCF_3$. The crude product was purified via flash column chromatography (0-2% EtOAc in hexane) to give **4p** as a colorless oil. The product was isolated as an inseparable >20:1 mixture of regioisomers.

Yield: 48.6 mg, 0.173 mmol, 86%, rr > 20:1

^{1}H NMR (400 MHz, $CDCl_3$): δ (ppm) = 4.68 (td, *J* = 10.9, 4.4 Hz, 1H), 3.28 (t, *J* = 6.6 Hz, 2H), 2.31 (t, *J* = 7.2 Hz, 2H), 1.97 (dddd, *J* = 11.9, 4.3, 3.4, 1.9 Hz, 1H), 1.84 (pd, *J* = 7.0, 2.8 Hz, 1H), 1.76 – 1.57 (m, 6H), 1.54 – 1.42 (m, 1H), 1.36 (ddt, *J* = 12.4, 10.8, 3.1 Hz, 1H), 1.12 – 0.78 (m, 9H), 0.75 (d, *J* = 7.0 Hz, 3H).

^{13}C NMR (101 MHz, $CDCl_3$): δ (ppm) = 172.8, 74.3, 51.2, 47.1, 41.1, 34.4, 34.2, 31.5, 28.4, 26.4, 23.5, 22.4, 22.1, 20.9, 16.4.

IR (thin film, cm^{-1}): 2955, 2930, 2870, 2096, 1729, 1455, 1370, 1252, 1180, 1151, 984.

HRMS (ESI+): *m/z* for $C_{15}H_{27}N_3NaO_2$ $[M+Na]^+$: calc.: 304.1995, found: 304.1988.

$[\alpha]_D^{29}$ = –55.6 (c = 1.0, $CHCl_3$).

TLC: R_f = 0.39 (SiO_2, 10% EtOAc in hexane).

Compound 4q:

5-Azidopentyl (S)-2-(6-methoxynaphthalen-2-yl)propanoate

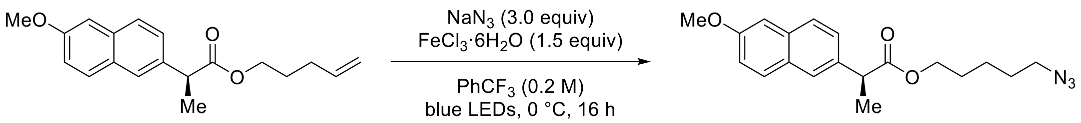

3q
from (+)-Naproxen

4q, 80%

Azide **4q** was prepared via GP1 from alkene **3q** (59.7 mg, 0.200 mmol, 1.00 equiv) in $PhCF_3$. The crude product was purified via flash column chromatography (0-5% EtOAc in hexane) to give **4q** as a colorless oil. The product was isolated as an inseparable 20:1 mixture of regioisomers.

Yield: 54.4 mg, 0.159 mmol, 80%, rr = 20:1

^{1}H NMR (400 MHz, $CDCl_3$): δ (ppm) = 7.71 (d, *J* = 8.6 Hz, 2H), 7.67 (d, *J* = 1.8 Hz, 1H), 7.41 (dd, *J* = 8.5, 1.8 Hz, 1H), 7.18 – 7.09 (m, 2H), 4.15 – 4.01 (m, 2H), 3.91 (s, 3H), 3.85 (q, *J* = 7.1 Hz, 1H), 3.10 (t, *J* = 6.9 Hz, 2H), 1.64 – 1.53 (m, 5H), 1.48 (dq, *J* = 8.8, 6.4 Hz, 2H), 1.34 – 1.23 (m, 2H).

^{13}C NMR (101 MHz, $CDCl_3$): δ (ppm) = 174.8, 157.8, 135.9, 133.8, 129.4, 129.0, 127.2, 126.4, 126.1, 119.1, 105.7, 64.5, 55.4, 51.3, 45.6, 28.5, 28.2, 23.2, 18.5.

IR (thin film, cm^{-1}): 2939, 2869, 2094, 1729, 1634, 1606, 1506, 1484, 1455, 1392, 1263, 1231, 1217, 1176, 1158, 1032, 926, 891, 854, 812.

HRMS (ESI+): *m/z* for $C_{19}H_{23}N_3NaO_3$ $[M+Na]^+$: calc.: 364.1632, found: 364.1629.

$[\alpha]_D^{27}$ = +30.0 (c = 1.0, $CHCl_3$).

TLC: R_f = 0.33 (SiO_2, 20% EtOAc in hexane).

Compound 4r:

(1S,2S,4R)-1,7,7-Trimethylbicyclo[2.2.1]heptan-2-yl 5-azidopentanoate

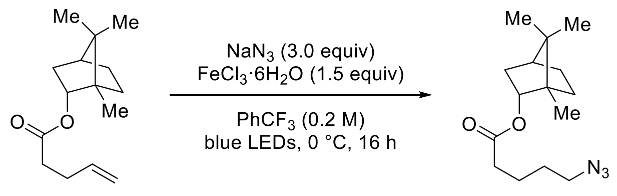

3r
from (–)-Borneol

4r, 77%

Azide **4r** was prepared via GP1 from alkene **3r** (47.3 mg, 0.200 mmol, 1.00 equiv) in $PhCF_3$. The crude product was purified via flash column chromatography (0-2% EtOAc in hexane) to give **4r** as a colorless oil. The product was isolated as an inseparable 14:1 mixture of regioisomers.

Yield: 42.9 mg, 0.154 mmol, 77%, rr = 14:1

^{1}H NMR (400 MHz, $CDCl_3$): δ (ppm) = 4.88 (ddd, *J* = 9.9, 3.6, 2.2 Hz, 1H), 3.29 (t, *J* = 6.6 Hz, 2H), 2.39 – 2.30 (m, 3H), 1.91 (ddd, *J* = 12.5, 9.3, 4.5 Hz, 1H), 1.78 – 1.59 (m, 6H), 1.34 – 1.17 (m, 2H), 0.94 (dd, *J* = 13.8, 3.5 Hz, 1H), 0.89 (s, 3H), 0.86 (s, 3H), 0.81 (s, 3H).

^{13}C NMR (101 MHz, $CDCl_3$): δ (ppm) = 173.5, 80.0, 51.2, 48.8, 47.9, 45.0, 36.9, 34.1, 28.4, 28.1, 27.2, 22.4, 19.8, 18.9, 13.6.

IR (thin film, cm^{-1}): 2953, 2875, 2094, 1730, 1454, 1386, 1349, 1252, 1182, 1157, 1114, 1023, 981.

HRMS (ESI+): *m/z* for $C_{15}H_{25}N_3NaO_2$ $[M+Na]^+$: calc.: 302.1839, found: 302.1833.

$[\alpha]_D^{29}$ = –29.7 (c = 1.0, $CHCl_3$).

TLC: R_f = 0.39 (SiO_2, 10% EtOAc in hexane).

Compound 4s:

5-Azidopentyl 2-(3-cyano-4-isobutoxyphenyl)-4-methylthiazole-5-carboxylate

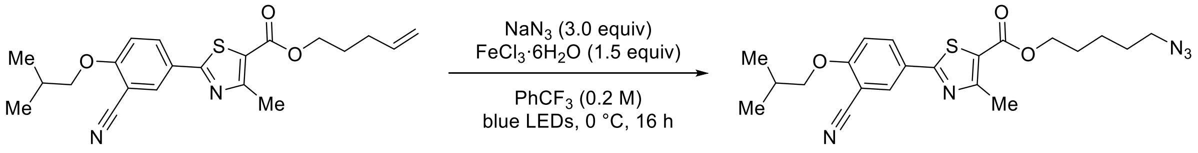

3s *from Febuxostat* — **4s**, 78%

Azide **4s** was prepared via GP1 from alkene **3s** (76.9 mg, 0.200 mmol, 1.00 equiv) in $PhCF_3$. The crude product was purified via flash column chromatography (0-10% EtOAc in hexane) to give **4s** as a white solid. The product was isolated as an inseparable 13:1 mixture of regioisomers.

Yield: 66.9 mg, 0.156 mmol, 78%, rr = 13:1

^{1}H NMR (400 MHz, $CDCl_3$): δ (ppm) = 8.17 (d, *J* = 2.3 Hz, 1H), 8.08 (dd, *J* = 8.8, 2.3 Hz, 1H), 7.00 (d, *J* = 8.9 Hz, 1H), 4.30 (t, *J* = 6.5 Hz, 2H), 3.89 (d, *J* = 6.5 Hz, 2H), 3.31 (t, *J* = 6.8 Hz, 2H), 2.76 (s, 3H), 2.20 (hept, *J* = 6.7 Hz, 1H), 1.85 – 1.73 (m, 2H), 1.73 – 1.63 (m, 2H), 1.58 – 1.48 (m, 2H), 1.08 (d, *J* = 6.7 Hz, 6H).

^{13}C NMR (101 MHz, $CDCl_3$): δ (ppm) = 167.4, 162.6, 162.2, 161.4, 132.7, 132.2, 126.1, 121.8, 115.5, 112.7, 103.1, 75.8, 65.1, 51.4, 28.6, 28.4, 28.3, 23.4, 19.2, 17.6.

IR (thin film, cm^{-1}): 2959, 2874, 2228, 2096, 1712, 1607, 1509, 1470, 1452, 1432, 1372, 1297, 1260, 1171, 1093, 1012, 827, 755.

HRMS (ESI+): *m/z* for $C_{21}H_{26}N_5O_3S$ $[M+H]^+$: calc.: 428.1751, found: 428.1741.

TLC: R_f = 0.26 (SiO_2, 20% EtOAc in hexane).

Compound 4t:

(±)-(1r,1'r,4R,4'R)-4-(Azidomethyl)-4'-(3,4-difluorophenyl)-1,1'-bi(cyclohexane)

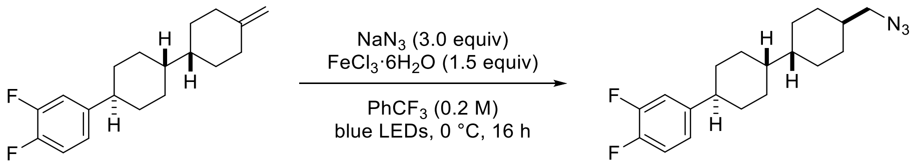

3t
from a liquid crystal building block

4t, 85%

Azide **4t** was prepared via GP1 from alkene **3t** (58.1 mg, 0.200 mmol, 1.00 equiv) in $PhCF_3$. The crude product was purified via flash column chromatography (0-2% EtOAc in hexane) to give **4t** as a colorless oil. The corresponding regioisomer could not be identified in the ^{1}H NMR spectrum of the unpurified reaction mixture.

Yield: 56.7 mg, 0.170 mmol, 85%, dr = 7:1

^{1}H NMR (400 MHz, $CDCl_3$): δ (ppm) = 7.08 – 6.95 (m, 2H), 6.91 – 6.86 (m, 1H), 3.12 (d, *J* = 6.7 Hz, 2H), 2.41 (tt, *J* = 12.2, 3.5 Hz, 1H), 1.93 – 1.76 (m, 8H), 1.54 – 1.45 (m, 1H), 1.42 – 1.29 (m, 2H), 1.19 – 0.86 (m, 8H).

^{13}C NMR (101 MHz, $CDCl_3$): δ (ppm) = 150.3 (dd, *J* = 246.9, 12.7 Hz), 148.7 (dd, *J* = 245.2, 12.7 Hz), 144.9, 122.6 (dd, *J* = 5.9, 3.3 Hz), 116.9 (d, *J* = 16.7 Hz), 115.5 (d, *J* = 16.7 Hz), 58.2, 44.0 (d, *J* = 1.1 Hz), 43.1, 42.8, 38.5, 34.7, 31.0, 30.3, 29.5.

^{19}F NMR (377 MHz, $CDCl_3$): δ (ppm) = -138.58 – -138.71 (m, 1F), -142.52 (dddd, *J* = 22.0, 10.4, 7.7, 4.3 Hz, 1F).

IR (thin film, cm^{-1}): 2921, 2853, 2094, 1607, 1517, 1449, 1276, 1207, 1116, 951, 939, 866, 817, 770, 626, 580.

HRMS (EI): *m/z* for $C_{19}H_{25}F_2N$ $[M–N_2]^+$: calc.: 305.1950, found: 305.1949.

TLC: R_f = 0.56 (SiO_2, 5% EtOAc in hexane).

Compound 4u:

6-Azidohexyl (tert-butoxycarbonyl)-L-leucinate

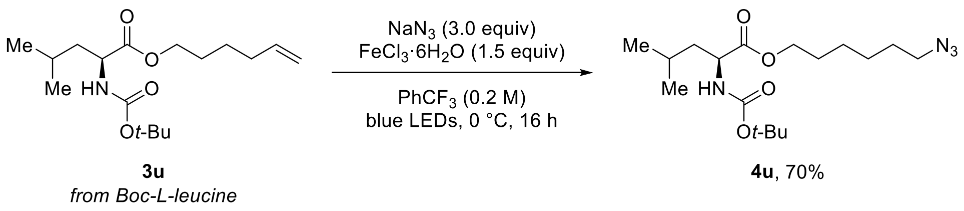

Azide **4u** was prepared via GP1 from alkene **3u** (62.7 mg, 0.200 mmol, 1.00 equiv) in $PhCF_3$. The crude product was purified via flash column chromatography (0-5% EtOAc in hexane) to give **4u** as a colorless oil. The product was isolated as an inseparable 12:1 mixture of regioisomers.

Yield: 49.6 mg, 0.139 mmol, 70%, rr = 12:1

^{1}H NMR (400 MHz, $CDCl_3$): δ (ppm) = 4.94 – 4.86 (m, 1H), 4.27 (td, *J* = 8.9, 5.7 Hz, 1H), 4.11 (t, *J* = 6.6 Hz, 2H), 3.26 (t, *J* = 6.9 Hz, 2H), 1.63 (dddd, *J* = 27.8, 14.2, 9.0, 7.0 Hz, 6H), 1.51 – 1.45 (m, 1H), 1.43 (s, 9H), 1.41 – 1.35 (m, 4H), 0.93 (dd, *J* = 6.5, 2.5 Hz, 6H).

^{13}C NMR (101 MHz, $CDCl_3$): δ (ppm) 173.7, 155.5, 79.9, 65.1, 52.3, 51.4, 42.0, 28.8, 28.5, 28.4, 26.4, 25.6, 24.9, 22.9, 22.1.

IR (thin film, cm^{-1}): 3365, 2975, 2937, 2870, 2095, 1712, 1504, 1470, 1455, 1366, 1249, 1161, 1047, 1021, 873, 780.

HRMS (ESI+): *m/z* for $C_{17}H_{32}N_4NaO_4$ $[M+Na]^+$: calc.: 379.2316, found: 379.2306.

$[\alpha]_D^{30}$ = –5.1 (c = 1.0, $CHCl_3$).

TLC: R_f = 0.43 (SiO_2, 20% EtOAc in hexane).

10. Procedures for One-pot Transformations

Compound 7a:

1-Decyl-4-phenyl-1H-1,2,3-triazole

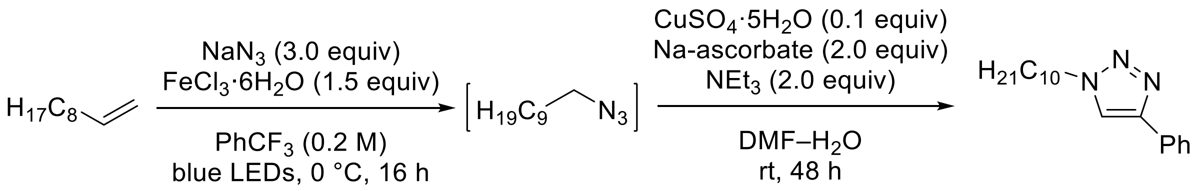

1aq **2aq** **7a**, 76%

Azide **2aq** was prepared via GP2 from alkene **1aq** (281 mg, 2.00 mmol, 1.00 equiv) in $PhCF_3$. A separate round-bottom flask under nitrogen atmosphere was charged with $CuSO_4·5H_2O$ (50.0 mg, 0.20 mmol, 0.100 equiv) and sodium ascorbate (792 mg, 4.0 mmol, 2.00 equiv), followed by 20 mL DMF and 2 mL H_2O. This suspension was stirred at room temperature for 30 min followed by addition of triethylamine (558 µL, 405 mg, 4.00 mmol, 2.00 equiv), phenylacetylene (408 mg, 4.00 mmol, 2.00 equiv), and the unpurified hydroazidation reaction mixture. The Cu-catalyzed alkyne-azide cycloaddition reaction was stirred for 48 h at room temperature, filtered over Celite, and the filtrate was concentrated *in vacuo*. The crude product was purified via flash column chromatography (0-2% MeOH in CH_2Cl_2) to give **7a** as a white solid.

Yield: 434 mg, 1.52 mmol, 76%

^{1}H NMR (400 MHz, $CDCl_3$): δ (ppm) = 7.86 – 7.80 (m, 2H), 7.74 (s, 1H), 7.45 – 7.38 (m, 2H), 7.36 – 7.29 (m, 1H), 4.38 (t, *J* = 7.2 Hz, 2H), 2.01 – 1.89 (m, 2H), 1.39 – 1.19 (m, 14H), 0.91 – 0.83 (m, 3H).

^{13}C NMR (101 MHz, $CDCl_3$): δ (ppm) = 147.8, 130.9, 128.9, 128.2, 125.8, 119.5, 50.6, 32.0, 30.5, 29.6, 29.5, 29.4, 29.1, 26.6, 22.8, 14.2.

IR (thin film, cm^{-1}): 2954, 2916, 2847, 1464, 1216, 1188, 1079, 1053, 977, 912, 839, 762, 695, 527, 517.

HRMS (ESI+): *m/z* for $C_{18}H_{28}N_3$ $[M+H]^+$: calc.: 286.2278, found: 286.2271.

TLC: R_f = 0.46 (SiO_2, 1% MeOH in CH_2Cl_2).

Compound 7b:

1-(2,3-Dimethylbutan-2-yl)-4,5,6,7,8,9-hexahydro-1H-cycloocta[d][1,2,3]triazole

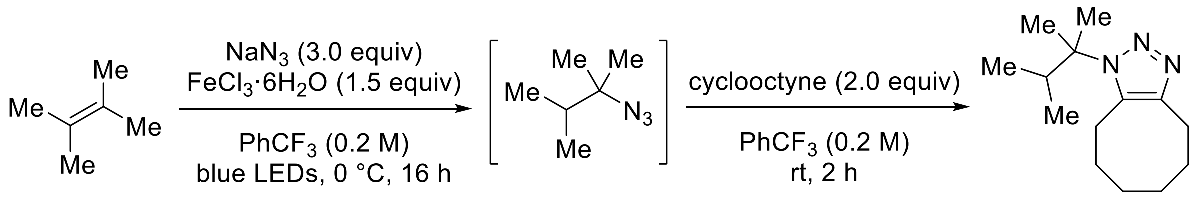

1ao **2ao** **7b**, 54%

Azide **2ao** was prepared via GP2 from alkene **1ao** (168 mg, 2.00 mmol, 1.00 equiv) in $PhCF_3$. After completion of the hydroazidation, cyclooctyne (433 mg, 4.00 mmol, 2.00 equiv) was added and the reaction was stirred for 2 h at room temperature. The reaction mixture was filtered over Celite, and the filtrate was concentrated *in vacuo*. The crude product was purified via flash column chromatography (0-20% EtOAc in hexane) to give **7b** as a white solid.

Yield: 253 mg, 1.07 mmol, 54%

^{1}H NMR (400 MHz, $CDCl_3$): δ (ppm) = 2.96 – 2.89 (m, 2H), 2.84 – 2.79 (m, 2H), 2.33 (hept, *J* = 6.8 Hz, 1H), 1.80 – 1.68 (m, 4H), 1.66 (s, 6H), 1.47 – 1.40 (m, 4H), 0.79 (d, *J* = 6.8 Hz, 6H).

^{13}C NMR (101 MHz, $CDCl_3$): δ (ppm) = 147.1, 132.5, 66.7, 37.2, 29.6, 28.8, 26.0, 25.7, 25.0, 24.6, 23.4, 17.6.

IR (thin film, cm^{-1}): 2928, 2853, 1560, 1459, 1398, 1384, 1372, 1238, 1200, 1120, 1046.

HRMS (ESI+): *m/z* for $C_{14}H_{26}N_3$ $[M+H]^+$: calc.: 236.2121, found: 236.2122.

TLC: R_f = 0.41 (SiO_2, 50% EtOAc in hexane).

Compound 7c:

Cyclohexylmethanamine

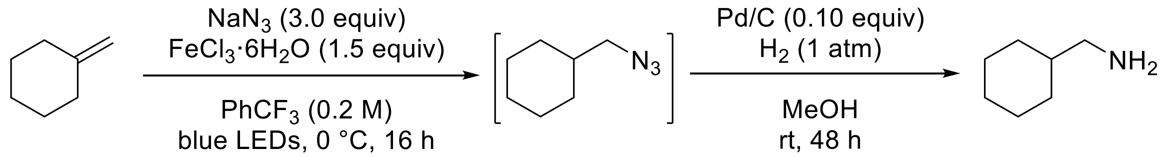

1aa **2aa** **7c**, 72%

Azide **2aa** was prepared via GP2 from alkene **1aa** (192 mg, 2.00 mmol, 1.00 equiv) in $PhCF_3$. After completion of the hydroazidation, the reaction mixture was transferred to a 100 mL round-bottom flask and 10.0 mL MeOH were added followed by Pd/C (10wt%, 213 mg, 0.200 mmol, 0.10 equiv). The reaction was stirred for 48 h under H_2 atmosphere (1 atm) at room temperature. The reaction mixture was filtered over Celite, and the filtrate was concentrated *in vacuo*. The crude product was purified via flash column chromatography (0-100% EtOAc in hexane) to give **7c** as a colorless oil.

Yield: 164 mg, 1.45 mmol, 72%

^{1}H NMR (400 MHz, $CDCl_3$): δ (ppm) = 2.49 (d, *J* = 6.5 Hz, 2H), 1.78 – 1.61 (m, 5H), 1.31 – 1.10 (m, 6H), 0.87 (qd, *J* = 12.7, 4.0 Hz, 2H).

^{13}C NMR (101 MHz, $CDCl_3$): δ (ppm) = 49.1, 41.5, 30.9, 26.8, 26.2.

IR (thin film, cm^{-1}): 2919, 2849, 1602, 1448, 962, 871, 817, 767.

HRMS (ESI+): *m/z* for $C_7H_{16}N$ $[M+H]^+$: calc.: 114.1277, found: 114.1277.

TLC: R_f = 0.15 (SiO_2, 100% EtOAc).

Compound 7d:

Tert-butyl cyclohexylcarbamate

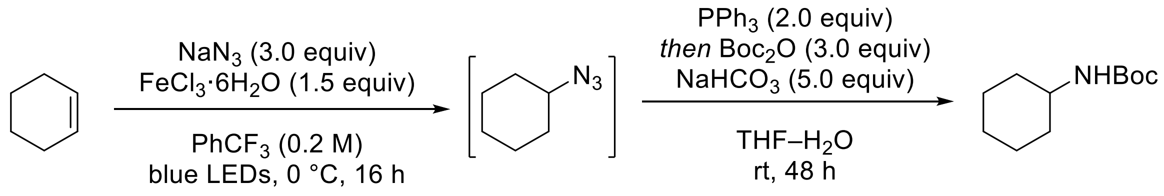

1ah **2ah** **7d**, 62%

Azide **2ah** was prepared via GP1 from alkene **1ah** (164 mg, 2.00 mmol, 1.00 equiv) in $PhCF_3$. After completion of the hydroazidation, the reaction mixture was transferred to a 100 mL round-bottom flask and 20.0 mL THF were added followed by 0.5 mL H_2O. The mixture was cooled to 0 °C and PPh_3 (1.05 g, 4.00 mmol, 2.00 equiv) dissolved in 10 mL THF was added dropwise. The reaction was allowed to warm to room temperature and stirred for 24 h. Subsequently, $NaHCO_3$ (840 mg, 10.0 mmol, 5.00 equiv) and Boc_2O (1.31 g, 6.00 mmol, 3.00 equiv) were added to the reaction at room temperature and the resulting mixture was stirred for additional 24 h. The reaction mixture was filtered over Celite, and the filtrate was concentrated *in vacuo*. The crude product was purified via flash column chromatography (0-5% EtOAc in hexane) to give **7d** as a white solid.

Yield: 247 mg, 1.24 mmol, 62%

^{1}H NMR (400 MHz, $CDCl_3$): δ (ppm) = 4.42 (bs, 1H), 3.39 (bs, 1H), 1.95 – 1.85 (m, 2H), 1.71 – 1.63 (m, 2H), 1.61 – 1.52 (m, 1H), 1.42 (s, 9H), 1.37 – 1.23 (m, 2H), 1.19 – 1.00 (m, 3H).

^{13}C NMR (101 MHz, $CDCl_3$): δ (ppm) = 155.3, 79.0, 49.5, 33.7, 28.6, 25.7, 25.0..

IR (thin film, cm^{-1}): 3364, 2932, 2854, 1681, 1520, 1449, 1365, 1316, 1252, 1233, 1167, 1046, 1026, 903, 780.

HRMS (ESI+): *m/z* for $C_{11}H_{21}NNaO_2$ $[M+Na]^+$: calc.: 222.1464, found: 222.1467.

TLC: R_f = 0.63 (SiO_2, 20% EtOAc in hexane).

Compound SI-7:

1-((3aR,4S,5R,7S,7aR)-3a,4,5,6,7,7a-hexahydro-1H-4,7-methanoinden-5-yl)-4-phenyl-1H-1,2,3-triazole

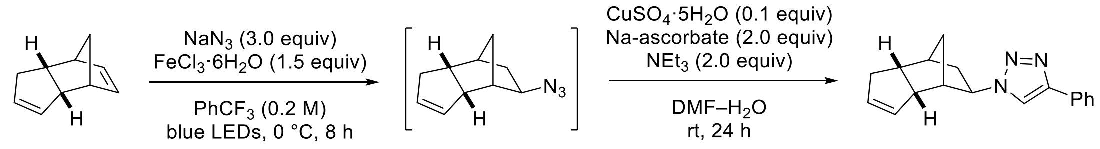

1al **2al** **SI-7**, 78%

Azide **2al** was prepared via GP2 from alkene **1al** (269 µL, 264 mg, 2.00 mmol, 1.00 equiv) in $PhCF_3$. A separate round-bottom flask under nitrogen atmosphere was charged with $CuSO_4{\cdot}5H_2O$ (50.0 mg, 0.20 mmol, 0.100 equiv) and sodium ascorbate (792 mg, 4.0 mmol, 2.00 equiv), followed by 20 mL DMF and 2 mL H_2O. This suspension was stirred at room temperature for 30 min followed by addition of triethylamine (558 µL, 405 mg, 4.00 mmol, 2.00 equiv), phenylacetylene (408 mg, 4.00 mmol, 2.00 equiv), and the unpurified hydroazidation reaction mixture. The Cu-catalyzed alkyne-azide cycloaddition reaction was stirred for 24 h at room temperature, filtered over Celite, and the filtrate was concentrated *in vacuo*. The crude product was purified via flash column chromatography (0-2% MeOH in CH_2Cl_2) to give **SI-7** as an off-white solid.

Yield: 435 mg, 1.57 mmol, 78%, rr = 1.4:1

^{1}H NMR (400 MHz, $CDCl_3$): δ (ppm) = 7.84 – 7.79 (m, 4H), 7.75 (s, 2H), 7.44 – 7.38 (m, 4H), 7.34 – 7.29 (m, 2H), 5.80 (dt, *J* = 5.8, 2.1 Hz, 1H), 5.78 – 5.73 (m, 1H), 5.70 (dtd, *J* = 5.7, 2.4, 0.8 Hz, 1H), 5.65 – 5.61 (m, 1H), 4.56 (ddd, *J* = 8.2, 4.2, 1.4 Hz, 1H), 4.50 (ddd, *J* = 8.6, 4.8, 1.4 Hz, 1H), 3.27 (dddd, *J* = 10.7, 5.5, 3.5, 2.0 Hz, 1H), 3.15 (ddd, *J* = 7.1, 5.0, 2.4 Hz, 1H), 2.79 – 2.61 (m, 3H), 2.61 – 2.52 (m, 2H), 2.45 – 2.38 (m, 3H), 2.38 – 2.30 (m, 1H), 2.24 (ddd, *J* = 5.6, 3.4, 2.3 Hz, 1H), 2.21 – 1.96 (m, 5H), 1.90 – 1.82 (m, 1H), 1.64 – 1.57 (m, 2H).

^{13}C NMR (101 MHz, $CDCl_3$): δ (ppm) = 147.6, 147.4, 132.7, 132.6, 131.1, 131.0, 131.0, 130.9, 128.9, 128.9, 128.1, 128.0, 125.7, 118.7, 118.3, 60.5, 58.2, 52.4, 51.7, 48.2, 46.4, 41.7, 41.4, 40.6, 39.8, 39.2, 38.8, 35.5, 32.6, 31.9, 31.4.

IR (thin film, cm^{-1}): 3137, 3044, 2954, 2925, 2852, 1633, 1483, 1457, 1355, 1230, 1077, 1045, 974, 946, 764, 695.

HRMS (ESI+): *m/z* for $C_{18}H_{20}N_3$ $[M+H]^+$: calc.: 278.1652, found: 278.1653.

TLC: R_f = 0.31 (SiO_2, 1% MeOH in CH_2Cl_2).

X-Ray crystallographic analysis: *vide infra* (page 210)

11. Preparation of Starting Materials

Compound 1q:

Pent-4-en-1-yl cyclopropanecarboxylate

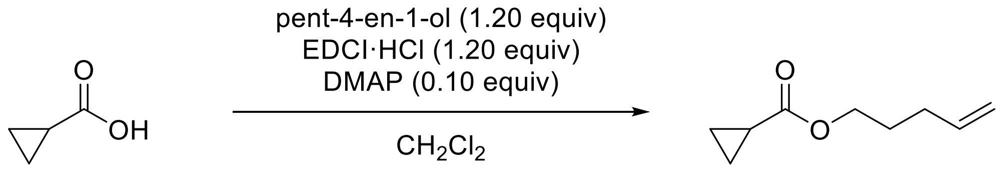

2q, 78%

To a 250 mL round-bottom flask charged with cyclopropane carboxylic acid (920 µl, 1.00 g, 11.6 mmol, 1.00 equiv) in CH_2Cl_2 (100 ml) were added pent-4-en-1-ol (1.20 g, 13.9 mmol, 1.20 equiv), 1-ethyl-3-(3-dimethylaminopropyl)carbodiimide hydrochloride (2.67 g, 13.9 mmol, 1.20 equiv), and 4-dimethylaminopyridine (142 mg, 1.16 mmol, 0.10 equiv) at 0 °C. After stirring for 24 h at room temperature, the reaction mixture was diluted with water (100 ml) and extracted with CH_2Cl_2 (3 x 150 mL). The combined organic phases were dried over Na_2SO_4. The solvent was removed *in vacuo* and the crude product was purified via flash column chromatography (0-5% EtOAc in hexane) to give **1q** as a colorless oil.

Yield: 1.39 g, 9.01 mmol, 78%

^{1}H NMR (400 MHz, $CDCl_3$): δ (ppm) = 5.80 (ddt, *J* = 16.9, 10.2, 6.6 Hz, 1H), 5.08 – 4.95 (m, 2H), 4.08 (t, *J* = 6.6 Hz, 2H), 2.13 (tdt, *J* = 7.9, 6.6, 1.4 Hz, 2H), 1.73 (dq, *J* = 8.3, 6.7 Hz, 2H), 1.59 (tt, *J* = 8.0, 4.7 Hz, 1H), 1.01 – 0.95 (m, 2H), 0.88 – 0.80 (m, 2H).

^{13}C NMR (101 MHz, $CDCl_3$): δ (ppm) = 175.0, 137.7, 115.4, 64.0, 30.2, 28.0, 13.0, 8.4.

IR (thin film, cm^{-1}): 3080, 3016, 2955, 2851, 1725, 1642, 1450, 1403, 1371, 1267, 1198, 1170, 1100, 1076, 1032, 993, 912, 853, 824, 746, 636.

HRMS (ESI+): *m/z* for $C_9H_{15}O_2$ $[M+H]^+$: calc.: 155.1067, found: 155.1068.

TLC: R_f = 0.32 (SiO_2, 4% EtOAc in hexane).

Compound 1w:

Pent-4-en-1-yl cyclopropanecarboxylate

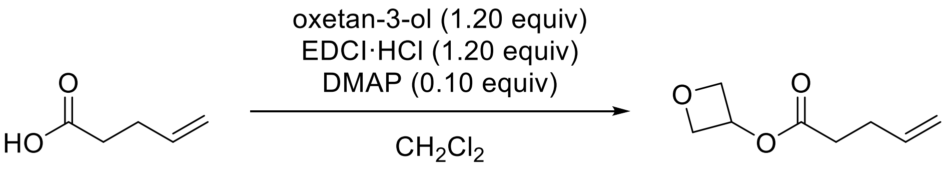

2q, 88%

To a 250 mL round-bottom flask charged with pent-4-enoic acid (1.02 ml, 1.00 g, 9.99 mmol, 1.00 equiv) in CH_2Cl_2 (100 ml) were added oxetan-3-ol (789 µl, 888 mg, 12.0 mmol, 1.20 equiv), 1-ethyl-3-(3-dimethylaminopropyl)carbodiimide hydrochloride (2,30 g, 12.0 mmol, 1.20 equiv), and 4-dimethylaminopyridine (122 mg, 1.00 mmol, 0.10 equiv) at 0 °C. After stirring for 24 h at room temperature, the reaction mixture was diluted with water (100 ml) and extracted with CH_2Cl_2 (3 x 150 mL). The combined organic phases were dried over Na_2SO_4. The solvent was removed *in vacuo* and the crude product was purified via flash column chromatography (0-10% EtOAc in hexane) to give **1w** as a colorless oil.

Yield: 1.38 g, 8.84 mmol, 88%

^{1}H NMR (400 MHz, $CDCl_3$): δ (ppm) = 5.81 (ddt, *J* = 17.1, 10.2, 6.2 Hz, 1H), 5.42 (tt, *J* = 6.4, 5.3 Hz, 1H), 5.12 – 4.96 (m, 2H), 4.87 (ddd, *J* = 7.5, 6.4, 1.0 Hz, 2H), 4.61 (ddd, *J* = 7.5, 5.3, 1.0 Hz, 2H), 2.48 – 2.43 (m, 2H), 2.41 – 2.35 (m, 2H).

^{13}C NMR (101 MHz, $CDCl_3$): δ (ppm) = 172.4, 136.4, 115.9, 77.7, 67.9, 33.3, 28.8.

IR (thin film, cm^{-1}): 3080, 2955, 2881, 1736, 1642, 1439, 1419, 1358, 1322, 1248, 1164, 1120, 1102, 1048, 971, 915, 870, 733, 647.

HRMS (ESI+): *m/z* for $C_8H_{13}O_3$ $[M+H]^+$: calc.: 157.0859, found: 157.0860.

TLC: R_f = 0.22 (SiO_2, 10% EtOAc in hexane).

Compound 3a:

Pent-4-en-1-yl 2-(4-(4-chlorobenzoyl)phenoxy)-2-methylpropanoate

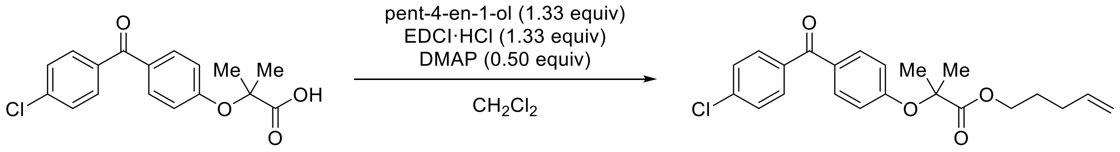

3a, 76%

To a 100 mL round-bottom flask charged with 2-(4-(4-chlorobenzoyl)phenoxy)-2-methylpropanoic acid (956 mg, 3.00 mmol, 1.00 equiv) in CH_2Cl_2 (50 ml) were added pent-4-en-1-ol (345 mg, 4.00 mmol, 1.33 equiv), 1-ethyl-3-(3-dimethylaminopropyl)carbodiimide hydrochloride (768 mg, 4.00 mmol, 1.33 equiv), and 4-dimethylaminopyridine (184 mg, 1.50 mmol, 0.500 equiv) at 0 °C. After stirring for 24 h at room temperature, the reaction mixture was diluted with water (100 ml) and extracted with CH_2Cl_2 (3 x 50 mL). The combined organic phases were dried over Na_2SO_4. The solvent was removed *in vacuo* and the crude product was purified via flash column chromatography (0-10% EtOAc in hexane) to give **3a** as a white solid.

Yield: 882 mg, 2.28 mmol, 76%

^{1}H NMR (400 MHz, $CDCl_3$): δ (ppm) = 7.75 – 7.68 (m, 4H), 7.46 – 7.42 (m, 2H), 6.89 – 6.84 (m, 2H), 5.79 – 5.64 (m, 1H), 4.99 – 4.90 (m, 2H), 4.17 (t, *J* = 6.5 Hz, 2H), 2.00 (dtt, *J* = 7.9, 6.5, 1.4 Hz, 2H), 1.76 – 1.64 (m, 8H).

^{13}C NMR (101 MHz, $CDCl_3$): δ (ppm) = 194.4, 173.9, 159.9, 138.5, 137.2, 136.5, 132.2, 131.3, 130.5, 128.7, 117.3, 115.7, 79.6, 65.2, 30.0, 27.7, 25.6.

IR (thin film, cm^{-1}): 2993, 2936, 1733, 1655, 1598, 1506, 1390, 1302, 1284, 1250, 1172, 1139, 1089, 1015, 927, 852, 838, 762, 639.

HRMS (ESI+): *m/z* for $C_{22}H_{24}ClO_4$ $[M+H]^+$: calc.: 387.1358, found: 387.1357.

TLC: R_f = 0.64 (SiO_2, 20% EtOAc in hexane).

Compound 3b:

1-(Hex-5-en-1-yl)-3,7-dimethyl-3,7-dihydro-1H-purine-2,6-dione

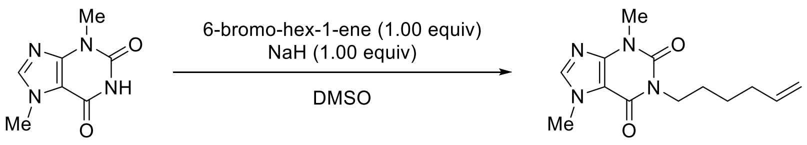

3b, 73%

To a 100 mL round-bottom flask charged with 3,7-dimethylxanthine (3.60 g, 20.0 mmol, 1.00 equiv) in DMSO (25 ml) were added 6-bromohex-1-ene (2.67 mL, 3.26 g, 20.0 mmol, 1.00 equiv) and sodium hydride (60% in mineral oil, 800 mg, 20.0 mmol, 1.00 equiv). After stirring for 48 h at room temperature, the reaction mixture was diluted with water (100 ml) and extracted with CH_2Cl_2 (3 x 100 mL). The combined organic phases were washed with 5% aq. LiCl solution (3 x 100 mL), water (100 mL), and dried over Na_2SO_4. The solvent was removed *in vacuo* and the crude product was purified via flash column chromatography (0-100% EtOAc in hexane) to give **3b** as a white solid.

Yield: 3.84 g, 14.6 mmol, 73%

^{1}H NMR (400 MHz, $CDCl_3$): δ (ppm) = 7.48 (d, *J* = 0.7 Hz, 1H), 5.79 (ddt, *J* = 16.9, 10.2, 6.7 Hz, 1H), 5.06 – 4.87 (m, 2H), 4.05 – 3.94 (m, 5H), 3.55 (s, 3H), 2.13 – 2.04 (m, 2H), 1.70 – 1.59 (m, 2H), 1.51 – 1.40 (m, 2H).

^{13}C NMR (101 MHz, $CDCl_3$): δ (ppm) = 155.4, 151.6, 148.9, 141.5, 138.7, 114.7, 107.8, 41.4, 33.7, 33.6, 29.8, 27.7, 26.4.

IR (thin film, cm^{-1}): 2924, 2856, 1698, 1651, 1548, 1486, 1456, 1414, 1357, 1324, 1286, 1233, 1048, 995, 866, 763, 749, 612.

HRMS (ESI+): *m/z* for $C_{13}H_{19}N_4O_2$ $[M+H]^+$: calc.: 263.1503, found: 263.1502.

TLC: R_f = 0.13 (SiO_2, 50% EtOAc in hexane).

Compound 3c:

Hex-5-en-1-yl 4-(N,N-dipropylsulfamoyl)benzoate

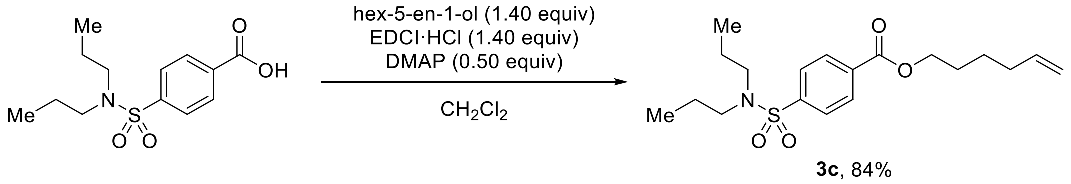

To a 100 mL round-bottom flask charged with 4-(N,N-dipropylsulfamoyl)benzoic acid (1.43 g, 5.00 mmol, 1.00 equiv) in CH_2Cl_2 (75 ml) were added hex-5-en-1-ol (701 mg, 7.00 mmol, 1.40 equiv), 1-ethyl-3-(3-dimethylaminopropyl)carbodiimide hydrochloride (1.34 g, 7.00 mmol, 1.40 equiv), and 4-dimethylaminopyridine (306 mg, 2.50 mmol, 0.500 equiv) at 0 °C. After stirring for 24 h at room temperature, the reaction mixture was diluted with water (100 ml) and extracted with CH_2Cl_2 (3 x 50 mL). The combined organic phases were dried over Na_2SO_4. The solvent was removed *in vacuo* and the crude product was purified via flash column chromatography (0-10% EtOAc in hexane) to give **3c** as a white solid.

Yield: 1.54 g, 4.19 mmol, 84%

^{1}H NMR (400 MHz, $CDCl_3$): δ (ppm) 8.17 – 8.11 (m, 2H), 7.89 – 7.83 (m, 2H), 5.80 (ddt, *J* = 16.9, 10.2, 6.7 Hz, 1H), 5.10 – 4.93 (m, 2H), 4.35 (t, *J* = 6.6 Hz, 2H), 3.12 – 3.06 (m, 4H), 2.18 – 2.07 (m, 2H), 1.84 – 1.74 (m, 2H), 1.60 – 1.47 (m, 6H), 0.85 (t, *J* = 7.4 Hz, 6H).

^{13}C NMR (101 MHz, $CDCl_3$): δ (ppm) = 165.4, 144.3, 138.3, 133.8, 130.3, 127.1, 115.1, 65.7, 50.0, 33.4, 28.2, 25.4, 22.0, 11.3.

IR (thin film, cm^{-1}): 2966, 2935, 2876, 1721, 1459, 1399, 1343, 1270, 1158, 1107, 1087, 1018, 991, 911, 862, 765, 740, 694.

HRMS (ESI+): *m/z* for $C_{19}H_{30}NO_4S$ $[M+H]^+$: calc.: 368.1890, found: 368.1890.

TLC: R_f = 0.58 (SiO_2, 20% EtOAc in hexane).

Compound 3d:

Hex-5-en-1-yl 3-(4,5-diphenyloxazol-2-yl)propanoate

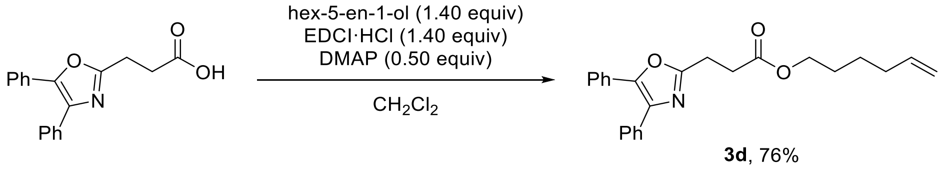

3d, 76%

To a 100 mL round-bottom flask charged with 3-(4,5-diphenyloxazol-2-yl)propanoic acid (1.47 g, 5.00 mmol, 1.00 equiv) in CH_2Cl_2 (50 ml) were added hex-5-en-1-ol (701 mg, 7.00 mmol, 1.40 equiv), 1-ethyl-3-(3-dimethylaminopropyl)carbodiimide hydrochloride (1.34 g, 7.00 mmol, 1.40 equiv), and 4-dimethylaminopyridine (306 mg, 2.50 mmol, 0.500 equiv) at 0 °C. After stirring for 24 h at room temperature, the reaction mixture was diluted with water (100 ml) and extracted with CH_2Cl_2 (3 x 50 mL). The combined organic phases were dried over Na_2SO_4. The solvent was removed *in vacuo* and the crude product was purified via flash column chromatography (0-20% EtOAc in hexane) to give **3d** as a colorless oil.

Yield: 1.43 g, 3.81 mmol, 76%

^{1}H NMR (400 MHz, $CDCl_3$): δ (ppm) =7.66 – 7.60 (m, 2H), 7.60 – 7.54 (m, 2H), 7.40 – 7.28 (m, 6H), 5.76 (ddt, *J* = 16.9, 10.2, 6.7 Hz, 1H), 5.03 – 4.91 (m, 2H), 4.13 (t, *J* = 6.6 Hz, 2H), 3.23 – 3.16 (m, 2H), 2.96 – 2.88 (m, 2H), 2.06 (dtt, *J* = 8.7, 7.1, 1.4 Hz, 2H), 1.73 – 1.57 (m, 2H), 1.50 – 1.40 (m, 2H).

^{13}C NMR (101 MHz, $CDCl_3$): δ (ppm) = 172.2, 161.9, 145.5, 138.4, 135.3, 132.6, 129.1, 128.8, 128.7, 128.6, 128.2, 128.0, 126.6, 115.0, 64.9, 33.4, 31.3, 28.2, 25.3, 23.7.

IR (thin film, cm^{-1}): 3063, 2935, 259, 1736, 1571, 1444, 1357, 1325, 1220, 1172, 1058, 1026, 995, 962, 914, 765, 694.

HRMS (ESI+): *m/z* for $C_{24}H_{26}NO_3$ $[M+H]^+$: calc.: 376.1907, found: 376.1903.

TLC: R_f = 0.31 (SiO_2, 20% EtOAc in hexane).

Compound 3e:

7-(Hex-5-en-1-yloxy)-4-methyl-2H-chromen-2-one

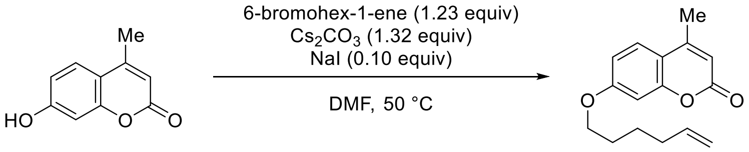

3e, 90%

To a 100 mL round-bottom flask charged with 7-hydroxy-4-methyl-2H-chromen-2-one (1.94 g, 11.0 mmol, 1.00 equiv) dissolved in DMF (50 mL) were added Cs_2CO_3 (4.73 g, 14.5 mmol, 1.32 equiv), 6-bromohex-1-ene (2.21 g, 13.5 mmol, 1.23 equiv), and NaI (165 mg, 1.10 mmol, 0.10 equiv). After stirring for 16 h at 50 °C, the reaction mixture was diluted with water (100 ml) and extracted with Et_2O (3 x 100 mL). The combined organic phases were washed with 1M aq. NaOH solution (3 x 100 mL), water (100 mL), and dried over Na_2SO_4. The solvent was removed *in vacuo* and the crude product was purified via flash column chromatography (0-20% EtOAc in hexane) to give **3e** as a off-white solid.

Yield: 2.57 g, 9.95 mmol, 90%

^{1}H NMR (400 MHz, $CDCl_3$): δ (ppm) = 7.46 (d, *J* = 8.8 Hz, 1H), 6.83 (dd, *J* = 8.8, 2.5 Hz, 1H), 6.77 (d, *J* = 2.5 Hz, 1H), 6.10 (q, *J* = 1.2 Hz, 1H), 5.81 (ddt, *J* = 16.9, 10.2, 6.7 Hz, 1H), 5.08 – 4.93 (m, 2H), 4.00 (t, *J* = 6.4 Hz, 2H), 2.38 (d, *J* = 1.2 Hz, 3H), 2.13 (tdt, *J* = 7.7, 6.6, 1.4 Hz, 2H), 1.87 – 1.77 (m, 2H), 1.62 – 1.52 (m, 2H).

^{13}C NMR (101 MHz, $CDCl_3$): δ (ppm) = 162.3, 161.4, 155.4, 152.7, 138.4, 125.6, 115.0, 113.5, 112.7, 111.9, 101.4, 68.5, 33.5, 28.5, 25.3, 18.8.

IR (thin film, cm^{-1}): 3076, 2940, 2871, 1714, 1610, 1510, 1386, 1368, 1293, 1280, 1263, 1200, 1145, 1137, 1069, 1014, 992, 946, 747, 706, 638.

HRMS (ESI+): *m/z* for $C_{16}H_{19}O_3$ $[M+H]^+$: calc.: 259.1329, found: 259.1329.

TLC: R_f = 0.32 (SiO_2, 20% EtOAc in hexane).

Compound 3f:

Pent-4-en-1-yl 2-(1-(4-chlorobenzoyl)-5-methoxy-2-methyl-1H-indol-3-yl)acetate

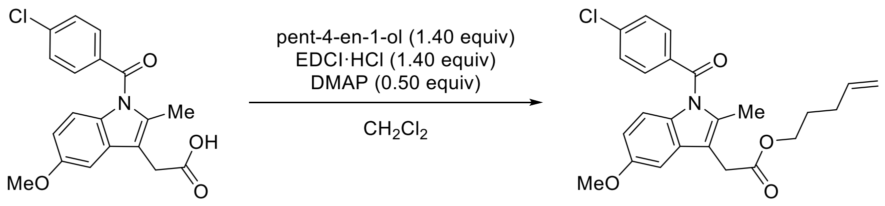

3f, 64%

To a 100 mL round-bottom flask charged with 2-(1-(4-chlorobenzoyl)-5-methoxy-2-methyl-1H-indol-3-yl)acetic acid (1.79 g, 5.00 mmol, 1.00 equiv) in CH_2Cl_2 (75 ml) were added pent-4-en-1-ol (603 mg, 7.00 mmol, 1.40 equiv), 1-ethyl-3-(3-dimethylaminopropyl)carbodiimide hydrochloride (1.34 g, 7.00 mmol, 1.40 equiv), and 4-dimethylaminopyridine (306 mg, 2.50 mmol, 0.500 equiv) at 0 °C. After stirring for 24 h at room temperature, the reaction mixture was diluted with water (100 ml) and extracted with CH_2Cl_2 (3 x 50 mL). The combined organic phases were dried over Na_2SO_4. The solvent was removed *in vacuo* and the crude product was purified via flash column chromatography (0-20% EtOAc in hexane) to give **3f** as a white solid.

Yield: 1.37 g, 3.22 mmol, 64%

^{1}H NMR (400 MHz, $CDCl_3$): δ (ppm) = 7.68 – 7.63 (m, 2H), 7.50 – 7.44 (m, 2H), 6.97 (d, *J* = 2.5 Hz, 1H), 6.87 (dd, *J* = 9.0, 0.5 Hz, 1H), 6.67 (dd, *J* = 9.0, 2.6 Hz, 1H), 5.84 – 5.68 (m, 1H), 5.02 – 4.92 (m, 2H), 4.11 (t, *J* = 6.6 Hz, 2H), 3.83 (s, 3H), 3.66 (s, 2H), 2.39 (s, 3H), 2.11 – 2.02 (m, 2H), 1.78 – 1.67 (m, 2H).

^{13}C NMR (101 MHz, $CDCl_3$): δ (ppm) = 171.0, 168.4, 156.2, 139.4, 137.4, 136.0, 134.1, 131.3, 131.0, 130.8, 129.3, 115.5, 115.1, 112.8, 111.8, 101.5, 64.6, 55.8, 30.6, 30.1, 28.0, 13.5.

IR (thin film, cm^{-1}): 2933, 2830, 1732, 1682, 1591, 1477, 1456, 1400, 1356, 1315, 1260, 1222, 1166, 1142, 1067, 1014, 924, 833, 754.

HRMS (ESI+): *m/z* for $C_{24}H_{25}ClNO_4$ $[M+H]^+$: calc.: 426.1467, found: 426.1464.

TLC: R_f = 0.44 (SiO_2, 20% EtOAc in hexane).

Compound 3g:

(±)-Tert-butyl (1R,3r,5S)-3-(hex-5-enoyloxy)-8-azabicyclo[3.2.1]octane-8-carboxylate

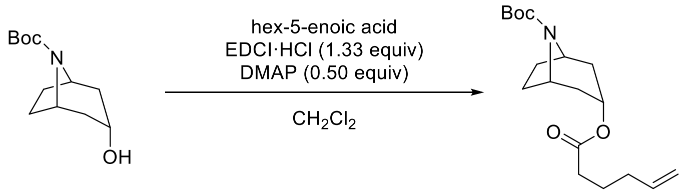

(1.33 equiv) **3g**, 69%

To a 100 mL round-bottom flask charged with hex-5-enoic acid (571 mg, 3.00 mmol, 1.00 equiv) in CH_2Cl_2 (50 ml) were added (±)-tert-butyl (1R,3r,5S)-3-hydroxy-8-azabicyclo[3.2.1]octane-8-carboxylate[31] (1.59 g, 4.00 mmol, 1.33 equiv), 1-ethyl-3-(3-dimethylaminopropyl)carbodiimide hydrochloride (768 mg, 4.00 mmol, 1.33 equiv), and 4-dimethylaminopyridine (183 mg, 1.50 mmol, 0.500 equiv) at 0 °C. After stirring for 24 h at room temperature, the reaction mixture was diluted with water (100 ml) and extracted with CH_2Cl_2 (3 x 50 mL). The combined organic phases were dried over Na_2SO_4. The solvent was removed *in vacuo* and the crude product was purified via flash column chromatography (0-20% EtOAc in hexane) to give **3g** as a colorless oil.

Yield: 670 mg, 2.07 mmol, 69%

^{1}H NMR (400 MHz, $CDCl_3$): δ (ppm) = 5.78 (ddt, *J* = 16.9, 10.2, 6.7 Hz, 1H), 5.14 – 4.94 (m, 3H), 4.18 (bd, *J* = 29.1 Hz, 2H), 2.35 – 2.27 (m, 2H), 2.23 – 2.04 (m, 4H), 2.02 – 1.92 (m, 4H), 1.80 – 1.66 (m, 4H), 1.46 (s, 9H).

^{13}C NMR (101 MHz, $CDCl_3$): δ (ppm) = 172.8, 153.5, 137.7, 115.6, 79.5, 67.9, 52.8, 52.1, 36.0, 35.3, 34.3, 33.2, 28.6, 28.4, 27.8, 24.1.

IR (thin film, cm^{-1}): 2975, 1732, 1694, 1392, 1365, 1328, 1318, 1244, 1162, 1100, 1032, 913, 867.

HRMS (ESI+): *m/z* for $C_{18}H_{29}NNaO_4$ $[M+Na]^+$: calc.: 346.1989, found: 346.1984.

TLC: R_f = 0.40 (SiO_2, 20% EtOAc in hexane).

Compound 3h:

N-(Hex-5-en-1-yl)-4-(5-(p-tolyl)-3-(trifluoromethyl)-1H-pyrazol-1-yl)benzene-sulfonamide

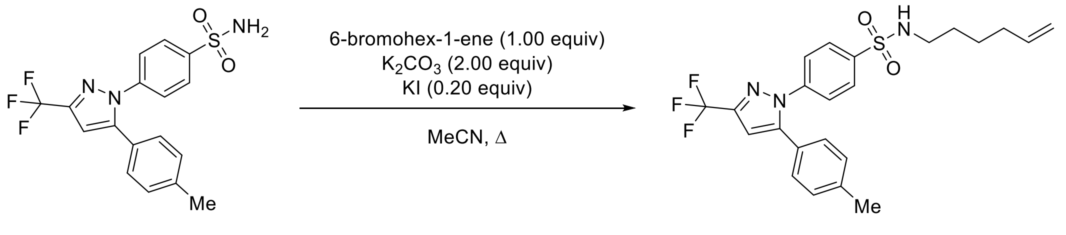

To a 100 mL round-bottom flask charged with 4-(5-(p-tolyl)-3-(trifluoromethyl)-1H-pyrazol-1-yl)benzenesulfonamide (1.91 g, 5.00 mmol, 1.00 equiv) in MeCN (25 ml) were added 6-bromohex-1-ene (817 mg, 5.00 mmol, 1.00 equiv), K_2CO_3 (1.38 g, 10.00 mmol, 2.00 equiv), and KI (166 mg, 1.00 mmol, 0.20 equiv). After stirring for 12 h under reflux, the reaction mixture was diluted with water (200 ml) and extracted with CH_2Cl_2 (3 x 100 mL). The combined organic phases were dried over Na_2SO_4. The solvent was removed *in vacuo* and the crude product was purified via flash column chromatography (0-20% EtOAc in hexane) to give **3h** as a white solid.

Yield: 1.24 g, 2.68 mmol, 54%

^{1}H NMR (400 MHz, $CDCl_3$): δ (ppm) = 7.88 – 7.81 (m, 2H), 7.50 – 7.45 (m, 2H), 7.20 – 7.15 (m, 2H), 7.13 – 7.08 (m, 2H), 6.74 (s, 1H), 5.73 (ddt, *J* = 17.0, 10.2, 6.7 Hz, 1H), 5.02 – 4.91 (m, 2H), 4.40 (t, *J* = 6.2 Hz, 1H), 2.96 (q, *J* = 6.9 Hz, 2H), 2.38 (s, 3H), 2.06 – 1.96 (m, 2H), 1.53 – 1.43 (m, 2H), 1.42 – 1.33 (m, 2H).

^{13}C NMR (101 MHz, $CDCl_3$): δ (ppm) = 145.4, 144.3 (q, *J* = 38.6 Hz), 142.6, 140.0, 139.6, 138.1, 129.9, 128.9, 128.2, 125.8, 125.7, 121.2 (q, *J* = 269.1 Hz), 115.3, 106.4 (q, *J* = 1.8 Hz), 43.3, 33.2, 29.1, 25.8, 21.5.

^{19}F NMR (377 MHz, $CDCl_3$): δ (ppm) = -62.46.

IR (thin film, cm^{-1}): 2936, 1598, 1472, 1374, 1331, 1272, 1236, 1159, 1134, 1096, 975, 842, 807, 626, 616.

HRMS (ESI+): *m/z* for $C_{23}H_{25}F_3N_3O_2S$ $[M+H]^+$: calc.: 464.1614, found: 464.1611.

TLC: R_f = 0.28 (SiO_2, 20% EtOAc in hexane).

Compound 3i:

Hex-5-en-1-yl (4R)-4-((3R,8R,9S,10S,13R,14S,17R)-3-acetoxy-10,13-dimethylhexadecahydro-1H-cyclopenta[a]phenanthren-17-yl)pentanoate

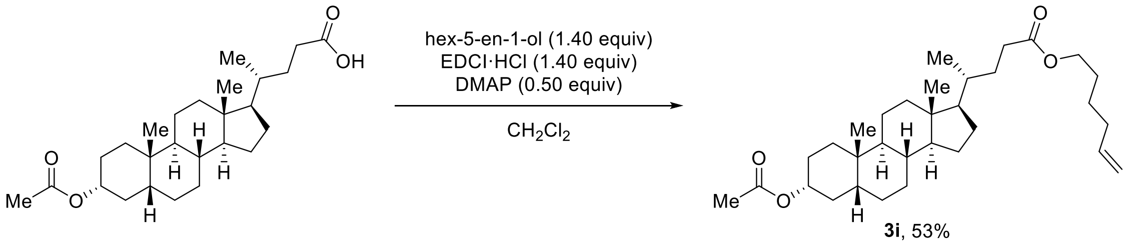

To a 250 mL round-bottom flask charged with (4R)-4-((3R,8R,9S,10S,13R,14S,17R)-3-acetoxy-10,13-dimethylhexadecahydro-1H-cyclopenta[a]phenanthren-17-yl) pentanoic acid[32] (2.09 g, 5.00 mmol, 1.00 equiv) in CH_2Cl_2 (100 ml) were added hex-5-en-1-ol (701 mg, 7.00 mmol, 1.40 equiv), 1-ethyl-3-(3-dimethylaminopropyl)-carbodiimide hydrochloride (1.34 g, 7.00 mmol, 1.40 equiv), and 4-dimethylaminopyridine (306 mg, 2.50 mmol, 0.500 equiv) at 0 °C. After stirring for 24 h at room temperature, the reaction mixture was diluted with water (100 ml) and extracted with CH_2Cl_2 (3 x 50 mL). The combined organic phases were dried over Na_2SO_4. The solvent was removed *in vacuo* and the crude product was purified via flash column chromatography (0-10% EtOAc in hexane) to give **3i** as a white solid.

Yield: 1.33 g, 2.66 mmol, 53%

^{1}H NMR (400 MHz, $CDCl_3$): δ (ppm) = 5.80 (ddt, *J* = 16.9, 10.2, 6.7 Hz, 1H), 5.06 – 4.93 (m, 2H), 4.72 (tt, *J* = 11.3, 4.7 Hz, 1H), 4.06 (t, *J* = 6.6 Hz, 2H), 2.34 (ddd, *J* = 15.2, 10.0, 5.2 Hz, 1H), 2.21 (ddd, *J* = 15.3, 9.5, 6.6 Hz, 1H), 2.12 – 2.05 (m, 2H), 2.03 (s, 3H), 2.00 – 1.93 (m, 1H), 1.90 – 1.74 (m, 5H), 1.72 – 1.59 (m, 3H), 1.60 – 1.50 (m, 3H), 1.50 – 1.20 (m, 13H), 1.19 – 0.97 (m, 5H), 0.94 – 0.89 (m, 6H), 0.64 (s, 3H).

^{13}C NMR (101 MHz, $CDCl_3$): δ (ppm) = 174.5, 170.8, 138.5, 115.0, 74.6, 64.3, 56.7, 56.2, 42.9, 42.0, 40.6, 40.3, 35.9, 35.5, 35.2, 34.7, 33.4, 32.4, 31.5, 31.2, 28.3, 28.3, 27.2, 26.8, 26.5, 25.4, 24.3, 23.5, 21.6, 21.0, 18.4, 12.2.

IR (thin film, cm^{-1}): 2936, 2866, 1735, 1448, 1379, 1362, 1240, 1167, 1027, 910.

HRMS (ESI+): *m/z* for $C_{32}H_{52}NaO_4$ $[M+Na]^+$: calc.: 523.3758, found: 523.3750.

$[\alpha]_D^{31}$ = +34.9 (c = 1.0, $CHCl_3$).

TLC: R_f = 0.64 (SiO_2, 20% EtOAc in hexane).

Compound 3j:

Pent-4-en-1-yl 3-methyl-4-oxo-2-phenyl-4H-chromene-8-carboxylate

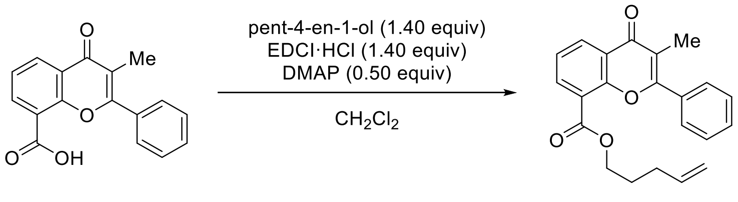

3j, 69%

To a 100 mL round-bottom flask charged with 3-methyl-4-oxo-2-phenyl-4H-chromene-8-carboxylic acid (1.40 g, 5.00 mmol, 1.00 equiv) in CH_2Cl_2 (50 ml) were added pent-4-en-1-ol (603 mg, 7.00 mmol, 1.40 equiv), 1-ethyl-3-(3-dimethylaminopropyl)carbodiimide hydrochloride (1.34 g, 7.00 mmol, 1.40 equiv), and 4-dimethylaminopyridine (306 mg, 2.50 mmol, 0.500 equiv) at 0 °C. After stirring for 24 h at room temperature, the reaction mixture was diluted with water (100 ml) and extracted with CH_2Cl_2 (3 x 50 mL). The combined organic phases were dried over Na_2SO_4. The solvent was removed *in vacuo* and the crude product was purified via flash column chromatography (0-20% EtOAc in hexane) to give **3j** as an off-white solid.

Yield: 1.20 g, 3.44 mmol, 69%

^{1}H NMR (400 MHz, $CDCl_3$): δ (ppm) = 8.47 (dd, *J* = 7.9, 1.8 Hz, 1H), 8.26 (dd, *J* = 7.5, 1.8 Hz, 1H), 7.80 – 7.75 (m, 2H), 7.56 – 7.51 (m, 3H), 7.45 (dd, *J* = 7.9, 7.5 Hz, 1H), 5.76 (ddt, *J* = 16.9, 10.2, 6.6 Hz, 1H), 5.05 – 4.94 (m, 2H), 4.36 (t, *J* = 6.7 Hz, 2H), 2.24 (s, 3H), 2.16 – 2.08 (m, 2H), 1.88 – 1.79 (m, 2H).

^{13}C NMR (101 MHz, $CDCl_3$): δ (ppm) = 178.5, 164.7, 161.3, 154.6, 137.4, 136.3, 133.2, 130.9, 130.6, 129.5, 128.6, 124.2, 123.5, 121.0, 117.8, 115.6, 65.2, 30.2, 27.9, 11.9.

IR (thin film, cm^{-1}): 2957, 1727, 1710, 1639, 1479, 1440, 1393, 1284, 1263, 1178, 1127, 1024, 914, 759, 699.

HRMS (ESI+): *m/z* for $C_{22}H_{20}NaO_4$ $[M+Na]^+$: calc.: 371.1254, found: 371.1253.

TLC: R_f = 0.36 (SiO_2, 20% EtOAc in hexane).

Compound 3k:

(S)-1-Ethoxy-1-oxopropan-2-yl pent-4-enoate

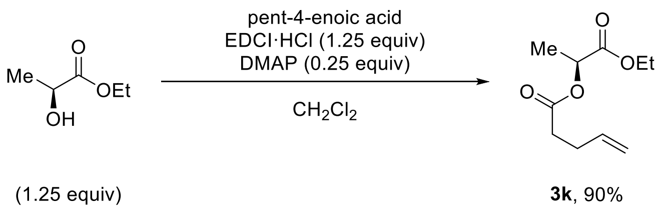

To a 100 mL round-bottom flask charged with pent-4-enoic acid (801 mg, 8.00 mmol, 1.00 equiv) in CH_2Cl_2 (50 ml) were added ethyl (S)-2-hydroxypropanoate (1.18 g, 10.0 mmol, 1.25 equiv), 1-ethyl-3-(3-dimethylaminopropyl)carbodiimide hydrochloride (1.92 g, 10.0 mmol, 1.25 equiv), and 4-dimethylaminopyridine (244 mg, 2.00 mmol, 0.25 equiv) at 0 °C. After stirring for 24 h at room temperature, the reaction mixture was diluted with water (100 ml) and extracted with CH_2Cl_2 (3 x 50 mL). The combined organic phases were dried over Na_2SO_4. The solvent was removed *in vacuo* and the crude product was purified via flash column chromatography (0-10% EtOAc in hexane) to give **3k** as a colorless oil.

Yield: 1.44 g, 7.19 mmol, 90%

^{1}H NMR (400 MHz, $CDCl_3$): δ (ppm) = 5.90 – 5.76 (m, 1H), 5.11 – 4.96 (m, 3H), 4.18 (q, J = 7.1 Hz, 2H), 2.55 – 2.44 (m, 2H), 2.44 – 2.35 (m, 2H), 1.47 (d, J = 7.1 Hz, 3H), 1.26 (t, J = 7.1 Hz, 3H).

^{13}C NMR (101 MHz, $CDCl_3$): δ (ppm) = 172.5, 170.9, 136.6, 115.7, 68.7, 61.4, 33.3, 28.8, 17.0, 14.2.

IR (thin film, cm^{-1}): 2985, 1739, 1449, 1371, 1270, 1204, 1165, 1133, 1097, 1051, 1020, 916, 862.

HRMS (ESI+): *m/z* for $C_{10}H_{16}NaO_4$ $[M+Na]^+$: calc.: 223.0941, found: 223.0940.

$[\alpha]_D^{31}$ = –35.2 (c = 1.0, $CHCl_3$).

TLC: R_f = 0.57 (SiO_2, 20% EtOAc in hexane).

Compound 3l:

Ethyl 4-(pent-4-enoyloxy)benzoate

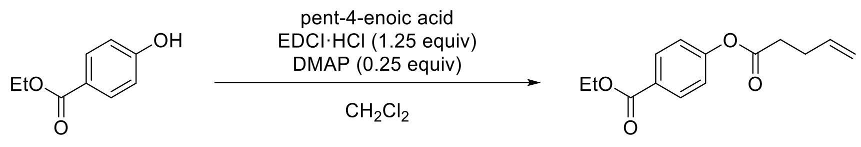

To a 100 mL round-bottom flask charged with pent-4-enoic acid (801 mg, 8.00 mmol, 1.00 equiv) in CH_2Cl_2 (50 ml) were added ethyl 4-hydroxybenzoate (1.67 g, 10.0 mmol, 1.25 equiv), 1-ethyl-3-(3-dimethylaminopropyl)carbodiimide hydrochloride (1.92 g, 10.0 mmol, 1.25 equiv), and 4-dimethylaminopyridine (244 mg, 2.00 mmol, 0.25 equiv) at 0 °C. After stirring for 24 h at room temperature, the reaction mixture was diluted with water (100 ml) and extracted with CH_2Cl_2 (3 x 50 mL). The combined organic phases were dried over Na_2SO_4. The solvent was removed *in vacuo* and the crude product was purified via flash column chromatography (0-15% EtOAc in hexane) to give **3l** as a colorless oil.

Yield: 1.91 g, 7.69 mmol, 96%

^{1}H NMR (400 MHz, $CDCl_3$): δ (ppm) = 8.10 – 8.04 (m, 2H), 7.17 – 7.12 (m, 2H), 5.90 (ddt, J = 16.8, 10.2, 6.5 Hz, 1H), 5.19 – 5.04 (m, 2H), 4.37 (q, J = 7.1 Hz, 2H), 2.68 (td, J = 7.4, 0.8 Hz, 2H), 2.56 – 2.47 (m, 2H), 1.38 (t, J = 7.1 Hz, 3H).

^{13}C NMR (101 MHz, $CDCl_3$): δ (ppm) = 171.1, 165.9, 154.4, 136.3, 131.2, 128.2, 121.7, 116.2, 61.2, 33.8, 28.9, 14.4.

IR (thin film, cm^{-1}): 2982, 1760, 1716, 1604, 1504, 1414, 1367, 1271, 1202, 1161, 1095, 1018, 926, 861, 765, 701.

HRMS (ESI+): *m/z* for $C_{14}H_{17}O_4$ $[M+H]^+$: calc.: 249.1121, found: 249.1125.

TLC: R_f = 0.59 (SiO_2, 20% EtOAc in hexane).

Compound 3m:

Pent-4-en-1-yl (2S,5R)-3,3-dimethyl-7-oxo-4-thia-1-azabicyclo[3.2.0]heptane-2-carboxylate 4,4-dioxide

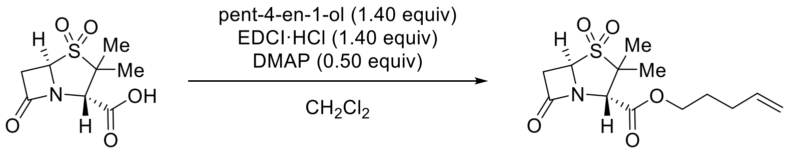

3m, 76%

To a 100 mL round-bottom flask charged with (2S,5R)-3,3-dimethyl-7-oxo-4-thia-1-azabicyclo[3.2.0]heptane-2-carboxylic acid 4,4-dioxide (1.17 g, 5.00 mmol, 1.00 equiv) in CH_2Cl_2 (50 ml) were added pent-4-en-1-ol (603 mg, 7.00 mmol, 1.40 equiv), 1-ethyl-3-(3-dimethylaminopropyl)carbodiimide hydrochloride (1.34 g, 7.00 mmol, 1.40 equiv), and 4-dimethylaminopyridine (306 mg, 2.50 mmol, 0.500 equiv) at 0 °C. After stirring for 24 h at room temperature, the reaction mixture was diluted with water (100 ml) and extracted with CH_2Cl_2 (3 x 50 mL). The combined organic phases were dried over Na_2SO_4. The solvent was removed *in vacuo* and the crude product was purified via flash column chromatography (0-30% EtOAc in hexane) to give **3m** as a colorless oil.

Yield: 1.14 g, 3.78 mmol, 76%

^{1}H NMR (400 MHz, $CDCl_3$): δ (ppm) = 5.78 (ddt, *J* = 16.9, 10.2, 6.7 Hz, 1H), 5.09 – 4.98 (m, 2H), 4.61 (dd, *J* = 4.2, 2.2 Hz, 1H), 4.37 (s, 1H), 4.21 (td, *J* = 6.7, 1.0 Hz, 2H), 3.49 (dd, *J* = 16.2, 4.2 Hz, 1H), 3.42 (dd, *J* = 16.2, 2.2 Hz, 1H), 2.18 – 2.08 (m, 2H), 1.84 – 1.73 (m, 2H), 1.60 (s, 3H), 1.40 (s, 3H).

^{13}C NMR (101 MHz, $CDCl_3$): δ (ppm) = 170.8, 167.1, 136.8, 116.0, 65.9, 63.4, 62.8, 61.2, 38.4, 30.0, 27.7, 20.5, 18.7.

IR (thin film, cm^{-1}): 2978, 2939, 1793, 1752, 1465, 1319, 1290, 1188, 1156, 1118, 1084, 996, 949, 917, 709, 629.

HRMS (ESI+): *m/z* for $C_{13}H_{19}NNaO_5S$ $[M+Na]^+$: calc.: 324.0876, found: 324.0880.

$[\alpha]_D^{31}$ = +159.7 (c = 1.0, $CHCl_3$).

TLC: R_f = 0.63 (SiO_2, 50% EtOAc in hexane).

Compound 3o:

Pent-4-en-1-yl 2-(11-oxo-6,11-dihydrodibenzo[b,e]oxepin-2-yl)acetate

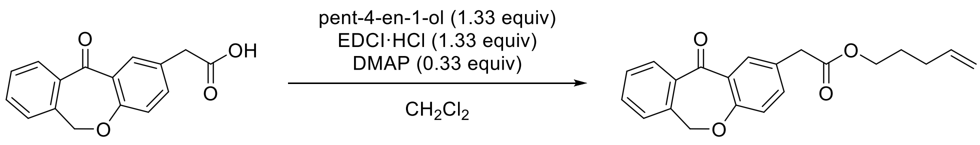

3o, 74%

To a 100 mL round-bottom flask charged with 2-(11-oxo-6,11-dihydrodibenzo[b,e]oxepin-2-yl)acetic acid (2.01 g, 7.50 mmol, 1.00 equiv) in CH_2Cl_2 (75 ml) were added pent-4-en-1-ol (862 mg, 10.0 mmol, 1.33 equiv), 1-ethyl-3-(3-dimethylaminopropyl)carbodiimide hydrochloride (1.92 mg, 10.0 mmol, 1.33 equiv), and 4-dimethylaminopyridine (306 mg, 2.50 mmol, 0.33 equiv) at 0 °C. After stirring for 24 h at room temperature, the reaction mixture was diluted with water (100 ml) and extracted with CH_2Cl_2 (3 x 50 mL). The combined organic phases were dried over Na_2SO_4. The solvent was removed *in vacuo* and the crude product was purified via flash column chromatography (0-20% EtOAc in hexane) to give **3o** as a white solid.

Yield: 1.86 g, 5.53 mmol, 74%

^{1}H NMR (400 MHz, $CDCl_3$): δ (ppm) = 8.12 (d, *J* = 2.4 Hz, 1H), 7.89 (dd, *J* = 7.7, 1.4 Hz, 1H), 7.56 (td, *J* = 7.5, 1.5 Hz, 1H), 7.47 (td, *J* = 7.6, 1.4 Hz, 1H), 7.43 (dd, *J* = 8.5, 2.4 Hz, 1H), 7.36 (dd, *J* = 7.6, 1.3 Hz, 1H), 7.03 (d, *J* = 8.4 Hz, 1H), 5.78 (ddt, *J* = 16.9, 10.2, 6.7 Hz, 1H), 5.18 (s, 2H), 5.05 – 4.94 (m, 2H), 4.11 (t, *J* = 6.6 Hz, 2H), 3.64 (s, 2H), 2.14 – 2.05 (m, 2H), 1.78 – 1.68 (m, 2H).

^{13}C NMR (101 MHz, $CDCl_3$): δ (ppm) = 191.0, 171.6, 160.6, 140.6, 137.5, 136.5, 135.7, 132.9, 132.6, 129.6, 129.4, 128.1, 127.9, 125.3, 121.2, 115.5, 73.8, 64.5, 40.4, 30.1, 27.9.

IR (thin film, cm^{-1}): 2978, 2920, 1732, 1646, 1611, 1599, 1489, 1455, 1413, 1380, 1299, 1221, 1160, 1138, 1121, 1014, 915, 829, 760, 698, 641, 622.

HRMS (ESI+): *m/z* for $C_{21}H_{20}NaO_4$ $[M+H]^+$: calc.: 359.1254, found: 359.1259.

TLC: R_f = 0.39 (SiO_2, 20% EtOAc in hexane).

Compound 3p:

(1R,2S,5R)-2-Isopropyl-5-methylcyclohexyl pent-4-enoate

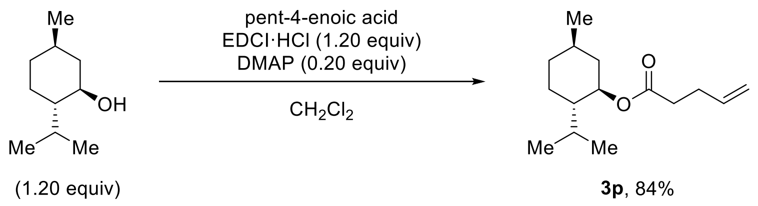

To a 250 mL round-bottom flask charged with pent-4-enoic acid (1.00 g, 10.0 mmol, 1.00 equiv) in CH_2Cl_2 (100 ml) were added (1R,2S,5R)-2-isopropyl-5-methylcyclohexan-1-ol (1.88 g, 12.0 mmol, 1.20 equiv), 1-ethyl-3-(3-dimethylaminopropyl)carbodiimide hydrochloride (2.30 g, 12.0 mmol, 1.20 equiv), and 4-dimethylaminopyridine (244 mg, 2.00 mmol, 0.20 equiv) at 0 °C. After stirring for 24 h at room temperature, the reaction mixture was diluted with water (100 ml) and extracted with CH_2Cl_2 (3 x 50 mL). The combined organic phases were dried over Na_2SO_4. The solvent was removed *in vacuo* and the crude product was purified via flash column chromatography (0-10% EtOAc in hexane) to give **3p** as a colorless oil.

Yield: 2.01 g, 8.43 mmol, 84%

^{1}H NMR (400 MHz, $CDCl_3$): δ (ppm) = 5.88 – 5.75 (m, 1H), 5.11 – 4.95 (m, 2H), 4.68 (td, *J* = 10.9, 4.4 Hz, 1H), 2.39 – 2.35 (m, 4H), 1.97 (dddd, *J* = 12.0, 4.3, 3.4, 1.9 Hz, 1H), 1.86 (pd, *J* = 7.0, 2.7 Hz, 1H), 1.72 – 1.63 (m, 2H), 1.48 (dddt, *J* = 15.3, 8.7, 6.7, 3.4 Hz, 1H), 1.36 (ddt, *J* = 12.4, 10.8, 3.1 Hz, 1H), 1.11 – 0.80 (m, 9H), 0.75 (d, *J* = 7.0 Hz, 3H).

^{13}C NMR (101 MHz, $CDCl_3$): δ (ppm) = 172.8, 136.9, 115.5, 74.3, 47.2, 41.1, 34.4, 34.1, 31.5, 29.2, 26.4, 23.6, 22.2, 20.9, 16.4.

IR (thin film, cm^{-1}): 2955, 2928, 2870, 1733, 1642, 1456, 1370, 1252, 1175, 1098, 1011, 984, 913, 844.

HRMS (ESI+): *m/z* for $C_{15}H_{26}NaO_2$ $[M+Na]^+$: calc.: 261.1825, found: 261.1828.

$[\alpha]_D^{31}$ = –70.1 (c = 1.0, $CHCl_3$).

TLC: R_f = 0.81 (SiO_2, 20% EtOAc in hexane).

Compound 3q:

Pent-4-en-1-yl (S)-2-(6-methoxynaphthalen-2-yl)propanoate

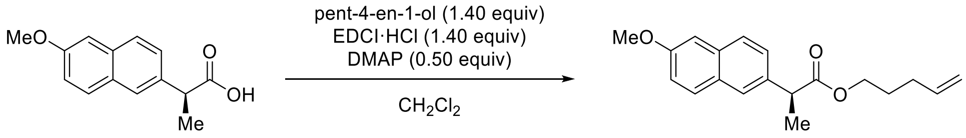

3q, 95%

To a 100 mL round-bottom flask charged with (S)-2-(6-methoxynaphthalen-2-yl)propanoic acid (1.15 g, 5.00 mmol, 1.00 equiv) in CH_2Cl_2 (75 ml) were added pent-4-en-1-ol (603 mg, 7.00 mmol, 1.40 equiv), 1-ethyl-3-(3-dimethylaminopropyl)carbodiimide hydrochloride (1.34 g, 7.00 mmol, 1.40 equiv), and 4-dimethylaminopyridine (306 mg, 2.50 mmol, 0.500 equiv) at 0 °C. After stirring for 24 h at room temperature, the reaction mixture was diluted with water (100 ml) and extracted with CH_2Cl_2 (3 x 50 mL). The combined organic phases were dried over Na_2SO_4. The solvent was removed *in vacuo* and the crude product was purified via flash column chromatography (0-10% EtOAc in hexane) to give **3q** as a white solid.

Yield: 1.41 g, 4.73 mmol, 95%

^{1}H NMR (400 MHz, $CDCl_3$): δ (ppm) = 7.75 – 7.65 (m, 3H), 7.42 (dd, *J* = 8.5, 1.9 Hz, 1H), 7.17 – 7.10 (m, 2H), 5.72 (ddt, *J* = 17.6, 9.7, 6.6 Hz, 1H), 4.97 – 4.89 (m, 2H), 4.09 (td, *J* = 6.6, 0.9 Hz, 2H), 3.92 (s, 3H), 3.86 (q, *J* = 7.1 Hz, 1H), 2.02 (tdt, *J* = 8.0, 6.6, 1.5 Hz, 2H), 1.72 – 1.63 (m, 2H), 1.58 (d, *J* = 7.2 Hz, 3H).

^{13}C NMR (101 MHz, $CDCl_3$): δ (ppm) = 174.8, 157.7, 137.5, 135.9, 133.8, 129.4, 129.1, 127.2, 126.4, 126.0, 119.1, 115.4, 105.7, 64.3, 55.4, 45.6, 30.1, 27.9, 18.6.

IR (thin film, cm^{-1}): 2976, 2937, 1729, 1634, 1606, 1506, 1484, 1454, 1392, 1326, 1264, 1231, 1218, 1176, 1157, 1091, 1032, 925, 853, 811, 747.

HRMS (ESI+): *m/z* for $C_{19}H_{22}NaO_3$ $[M+Na]^+$: calc.: 321.1461, found: 321.1460.

$[\alpha]_D^{31}$ = +28.3 (c = 1.0, $CHCl_3$).

TLC: R_f = 0.56 (SiO_2, 20% EtOAc in hexane).

Compound 3r:

(1R,2R,4S)-1,7,7-Trimethylbicyclo[2.2.1]heptan-2-yl pent-4-enoate

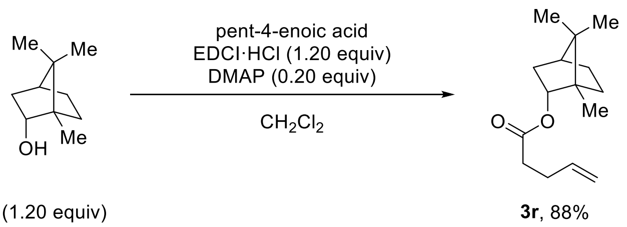

To a 250 mL round-bottom flask charged with pent-4-enoic acid (1.00 g, 10.0 mmol, 1.00 equiv) in CH_2Cl_2 (100 ml) were added (1R,2R,4S)-1,7,7-trimethylbicyclo[2.2.1]heptan-2-ol (1.85 g, 12.0 mmol, 1.20 equiv), 1-ethyl-3-(3-dimethylaminopropyl)carbodiimide hydrochloride (2.30 g, 12.0 mmol, 1.20 equiv), and 4-dimethylaminopyridine (244 mg, 2.00 mmol, 0.20 equiv) at 0 °C. After stirring for 24 h at room temperature, the reaction mixture was diluted with water (100 ml) and extracted with CH_2Cl_2 (3 x 50 mL). The combined organic phases were dried over Na_2SO_4. The solvent was removed *in vacuo* and the crude product was purified via flash column chromatography (0-10% EtOAc in hexane) to give **3r** as a colorless oil.

Yield: 2.07 g, 8.76 mmol, 88%

^{1}H NMR (400 MHz, $CDCl_3$): δ (ppm) = 5.91 – 5.77 (m, 1H), 5.11 – 4.97 (m, 2H), 4.89 (ddd, *J* = 10.0, 3.5, 2.1 Hz, 1H), 2.47 – 2.29 (m, 5H), 1.94 (ddd, *J* = 12.3, 9.2, 4.5 Hz, 1H), 1.80 – 1.69 (m, 1H), 1.66 (t, *J* = 4.6 Hz, 1H), 1.35 – 1.17 (m, 2H), 0.95 (dd, *J* = 13.8, 3.5 Hz, 1H), 0.90 (s, 3H), 0.87 (s, 3H), 0.82 (s, 3H).

^{13}C NMR (101 MHz, $CDCl_3$): δ (ppm) = 173.5, 136.9, 115.6, 80.0, 48.9, 47.9, 45.0, 36.9, 34.0, 29.2, 28.2, 27.3, 19.8, 19.0, 13.6.

IR (thin film, cm^{-1}): 2955, 2881, 1735, 1455, 1354, 1303, 1256, 1178, 1160, 1114, 1027, 992, 914.

HRMS (ESI+): *m/z* for $C_{15}H_{24}NaO_2$ $[M+Na]^+$: calc.: 259.1669, found: 259.1667.

$[\alpha]_D^{31}$ = –38.5 (c = 1.0, $CHCl_3$).

TLC: R_f = 0.78 (SiO_2, 20% EtOAc in hexane).

Compound 3s:

Pent-4-en-1-yl 2-(3-cyano-4-isobutoxyphenyl)-4-methylthiazole-5-carboxylate

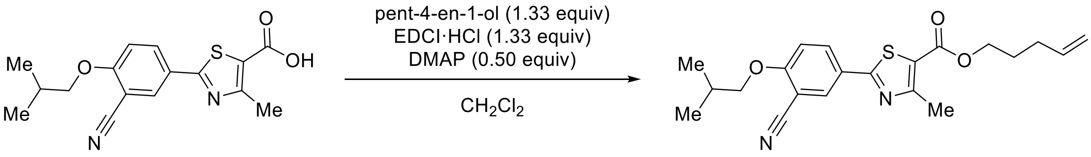

3s, 55%

To a 100 mL round-bottom flask charged with 2-(3-cyano-4-isobutoxyphenyl)-4-methylthiazole-5-carboxylic acid (950 mg, 3.00 mmol, 1.00 equiv) in CH_2Cl_2 (25 ml) were added pent-4-en-1-ol (345 mg, 4.00 mmol, 1.33 equiv), 1-ethyl-3-(3-dimethylaminopropyl)carbodiimide hydrochloride (767 mg, 4.00 mmol, 1.33 equiv), and 4-dimethylaminopyridine (183 mg, 1.50 mmol, 0.500 equiv) at 0 °C. After stirring for 24 h at room temperature, the reaction mixture was diluted with water (100 ml) and extracted with CH_2Cl_2 (3 x 50 mL). The combined organic phases were dried over Na_2SO_4. The solvent was removed *in vacuo* and the crude product was purified via flash column chromatography (0-10% EtOAc in hexane) to give **3s** as a white solid.

Yield: 635 mg, 1.65 mmol, 55%

^{1}H NMR (400 MHz, $CDCl_3$): δ (ppm) = 8.18 (d, *J* = 2.3 Hz, 1H), 8.09 (dd, *J* = 8.9, 2.3 Hz, 1H), 7.01 (d, *J* = 8.9 Hz, 1H), 5.84 (ddt, *J* = 16.9, 10.2, 6.6 Hz, 1H), 5.12 – 5.00 (m, 2H), 4.31 (t, *J* = 6.6 Hz, 2H), 3.90 (d, *J* = 6.5 Hz, 2H), 2.77 (s, 3H), 2.27 – 2.15 (m, 3H), 1.86 (dq, *J* = 8.2, 6.6 Hz, 2H), 1.09 (d, *J* = 6.7 Hz, 6H).

^{13}C NMR (101 MHz, $CDCl_3$): δ (ppm) = 167.4, 162.6, 162.2, 161.3, 137.4, 132.7, 132.3, 126.2, 122.0, 115.7, 115.5, 112.8, 103.2, 77.5, 64.9, 30.2, 28.3, 28.0, 19.2, 17.6.

IR (thin film, cm^{-1}): 2958, 2878, 2230, 1704, 1604, 1509, 1432, 1372, 1290, 1262, 1174, 1130, 1104, 1006, 914, 827, 761.

HRMS (ESI+): *m/z* for $C_{21}H_{25}N_2O_3S$ $[M+H]^+$: calc.: 385.1580, found: 385.1578.

TLC: R_f = 0.48 (SiO_2, 20% EtOAc in hexane).

Compound 3t:

(1r,4r)-4-(3,4-difluorophenyl)-4'-methylene-1,1'-bi(cyclohexane)

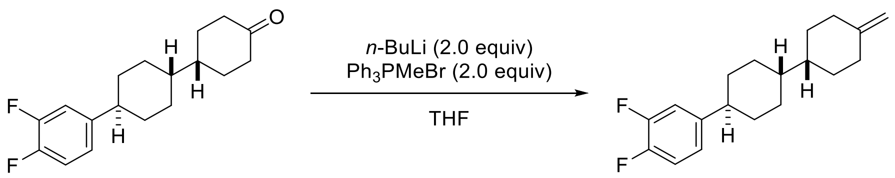

3t, 95%

To a 100 mL round-bottom flask charged with methyltriphenylphosphonium bromide (4.89 g, 13.7 mmol, 2.00 equiv) in THF (15 ml) was added *n*-BuLi (1.6 M in hexane, 8.55 mL, 13.7 mmol, 2.00 equiv) at 0 °C. The cooling bath was removed, and the reaction was stirred for 1 h before (1'r,4'r)-4'-(3,4-difluorophenyl)-[1,1'-bi(cyclohexan)]-4-one (2.00 g, 6.84 mmol, 1.00 equiv) in 15 mL THF was added. After stirring for 24 h at room temperature, the reaction was quenched with sat. aq. NH_4Cl solution (50 ml) and extracted with Et_2O (3 x 50 mL). The combined organic phases were dried over Na_2SO_4. The solvent was removed *in vacuo* and the crude product was purified via flash column chromatography (100% hexane) to give **3t** as colorless oil.

Yield: 1.89 g, 6.51 mmol, 95%

^{1}H NMR (400 MHz, $CDCl_3$): δ (ppm) = 7.10 – 6.95 (m, 2H), 6.89 (ddt, *J* = 8.2, 3.9, 1.7 Hz, 1H), 4.60 (t, *J* = 1.7 Hz, 2H), 2.42 (tt, *J* = 12.2, 3.4 Hz, 1H), 2.37 – 2.29 (m, 2H), 2.07 – 1.97 (m, 2H), 1.93 – 1.80 (m, 6H), 1.43 – 1.25 (m, 3H), 1.22 – 1.07 (m, 5H).

^{13}C NMR (101 MHz, $CDCl_3$): δ (ppm) = 150.3 (dd, *J* = 246.8, 12.6 Hz), 150.2, 148.7 (dd, *J* = 245.2, 12.7 Hz), 144.9 (dd, *J* = 5.0, 3.7 Hz), 122.6 (dd, *J* = 5.9, 3.3 Hz), 116.9 (d, *J* = 16.7 Hz), 115.5 (d, *J* = 16.6 Hz), 106.6, 44.0, 42.9, 42.4, 35.1, 34.7, 31.6, 30.3.

^{19}F NMR (376 MHz, $CDCl_3$): δ (ppm) = -138.55 – -138.71 (m), -142.51 (dddd, *J* = 22.0, 10.4, 7.7, 4.3 Hz).

IR (thin film, cm^{-1}): 3069, 2921, 2852, 1651, 1607, 1516, 1448, 1430, 1276, 1209, 1115, 990, 939, 886, 865, 815, 803, 770, 751, 627, 612.

HRMS (ESI+): *m/z* for $C_{19}H_{25}F_2$ $[M+H]^+$: calc.: 291.1919, found: 291.1919.

TLC: R_f = 0.48 (SiO_2, 100% hexane).

Compound 3u:

Hex-5-en-1-yl (tert-butoxycarbonyl)-L-leucinate

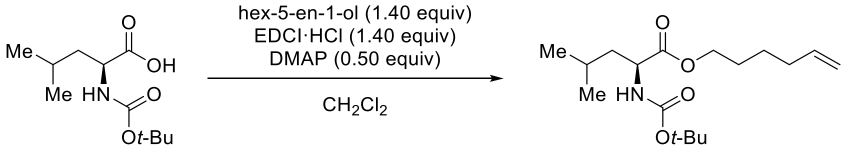

3u, 78%

To a 100 mL round-bottom flask charged with Boc-L-leucine (1.16 g, 5.00 mmol, 1.00 equiv) in CH_2Cl_2 (50 ml) were added hex-5-en-1-ol (701 mg, 7.00 mmol, 1.40 equiv), 1-ethyl-3-(3-dimethylaminopropyl)carbodiimide hydrochloride (1.34 g, 7.00 mmol, 1.40 equiv), and 4-dimethylaminopyridine (305 mg, 2.50 mmol, 0.500 equiv) at 0 °C. After stirring for 24 h at room temperature, the reaction mixture was diluted with water (100 ml) and extracted with CH_2Cl_2 (3 x 50 mL). The combined organic phases were dried over Na_2SO_4. The solvent was removed *in vacuo* and the crude product was purified via flash column chromatography (0-10% EtOAc in hexane) to give **3u** as a colorless oil.

Yield: 1.23 g, 3.92 mmol, 78%

^{1}H NMR (400 MHz, $CDCl_3$): δ (ppm) = 5.78 (ddt, *J* = 16.9, 10.2, 6.7 Hz, 1H), 5.07 – 4.94 (m, 2H), 4.88 (d, *J* = 8.8 Hz, 1H), 4.35 – 4.23 (m, 1H), 4.12 (td, *J* = 6.7, 1.6 Hz, 2H), 2.14 – 2.03 (m, 2H), 1.77 – 1.55 (m, 5H), 1.52 – 1.40 (m, 11H), 0.94 (dd, *J* = 6.5, 2.6 Hz, 6H).

^{13}C NMR (101 MHz, $CDCl_3$): δ (ppm) = 173.7, 155.5, 138.4, 115.0, 79.9, 65.2, 52.3, 42.1, 33.3, 28.5, 28.1, 25.2, 24.9, 22.9, 22.1.

IR (thin film, cm^{-1}): 2958, 2871, 1738, 1715, 1503, 1471, 1455, 1366, 1250, 1162, 1048, 1022, 994, 911, 874.

HRMS (ESI+): *m/z* for $C_{17}H_{31}NNaO_4$ $[M+Na]^+$: calc.: 336.2145, found: 336.2146.

$[\alpha]_D^{31}$ = –6.0 (c = 1.0, $CHCl_3$).

TLC: R_f = 0.57 (SiO_2, 20% EtOAc in hexane).

12. ^{1}H, ^{13}C, and ^{19}F NMR Spectra

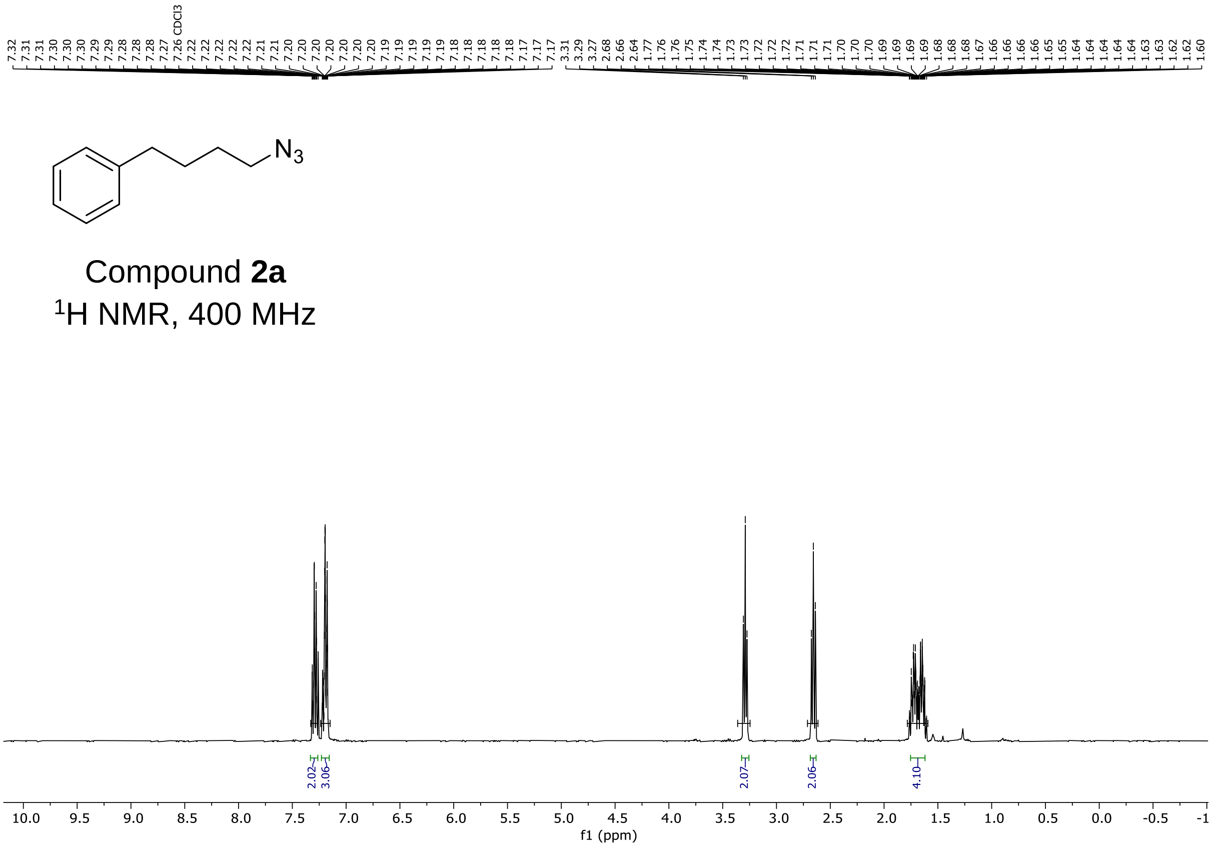

Compound **2a**
^{1}H NMR, 400 MHz

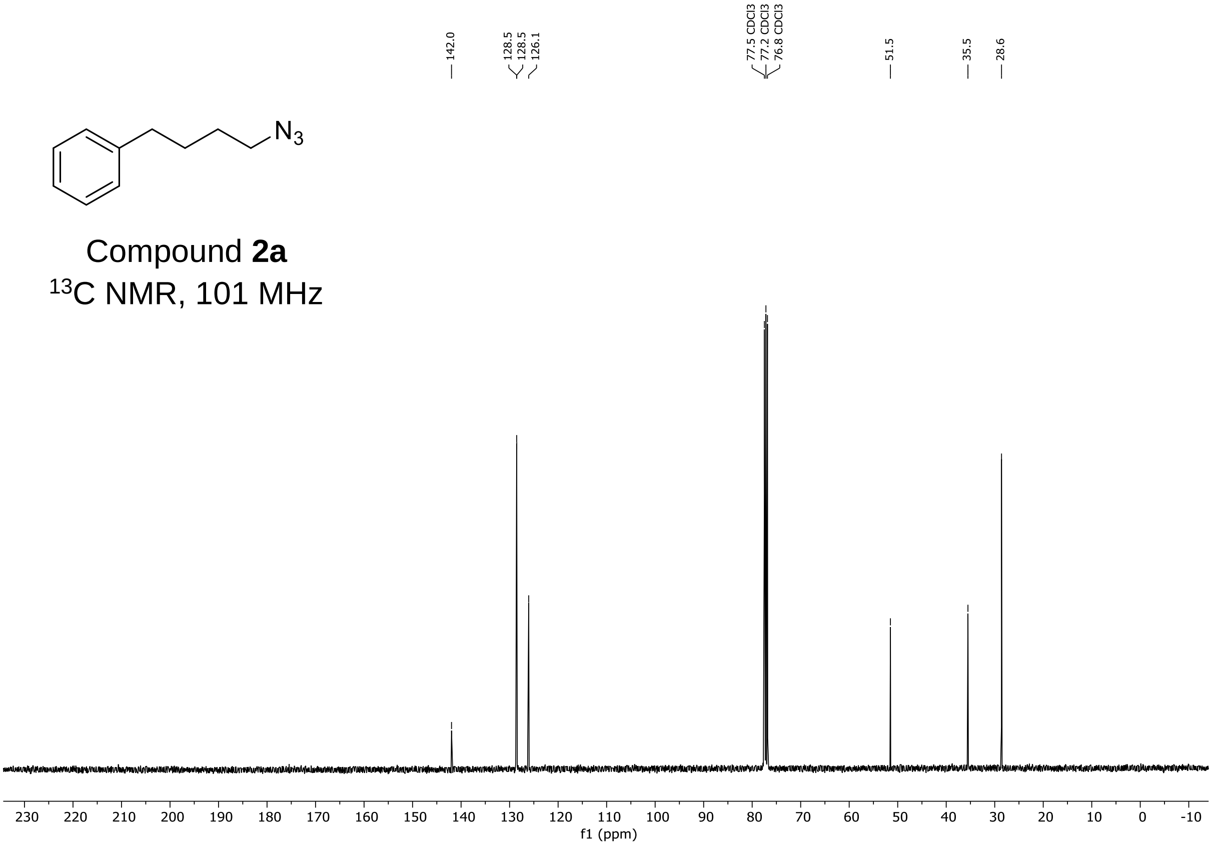

Compound **2a**
^{13}C NMR, 101 MHz

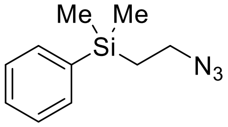

Compound **2b**
^1H NMR, 400 MHz

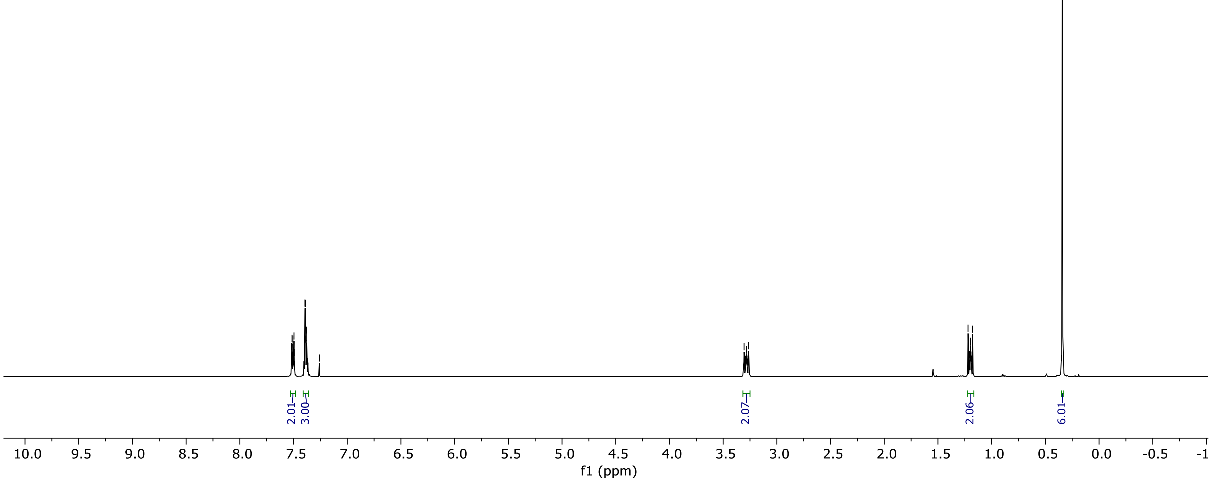

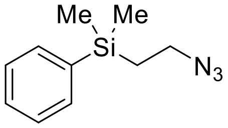

Compound **2b**
^{13}C NMR, 101 MHz

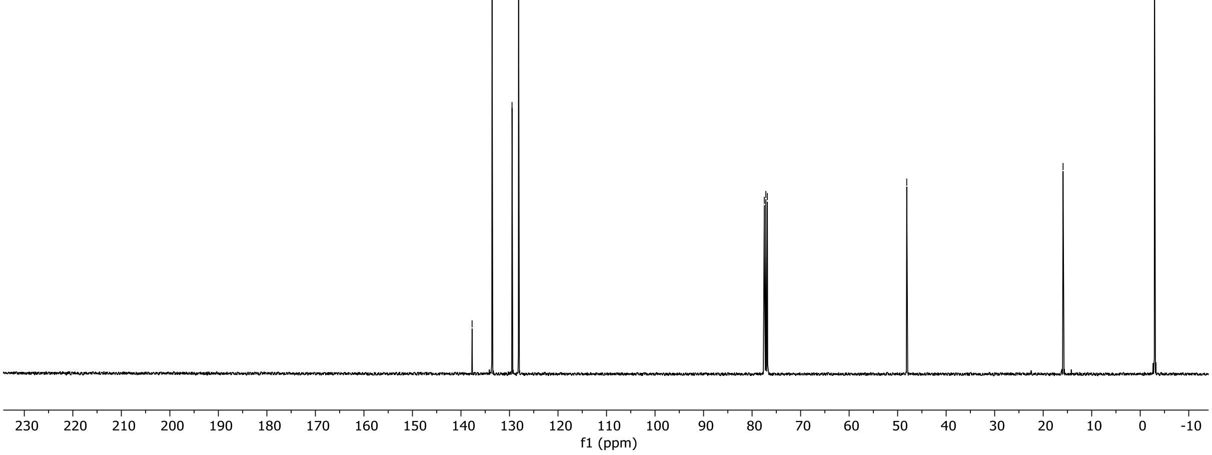

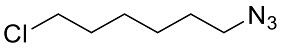

Compound **2c**
^{1}H NMR, 400 MHz

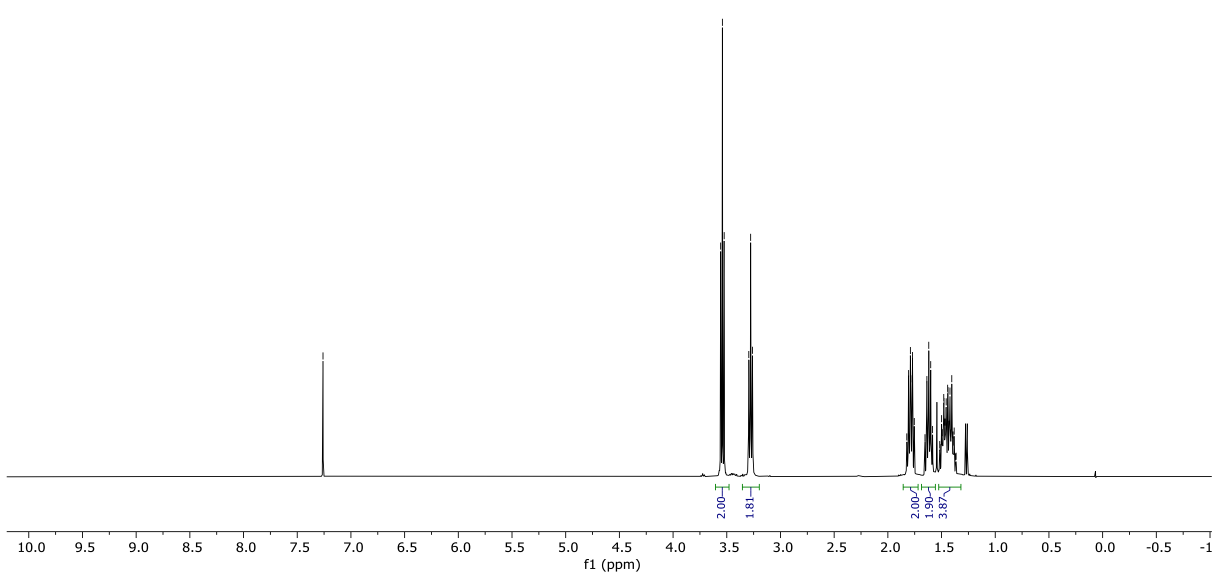

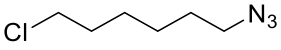

Compound **2c**
^{13}C NMR, 101 MHz

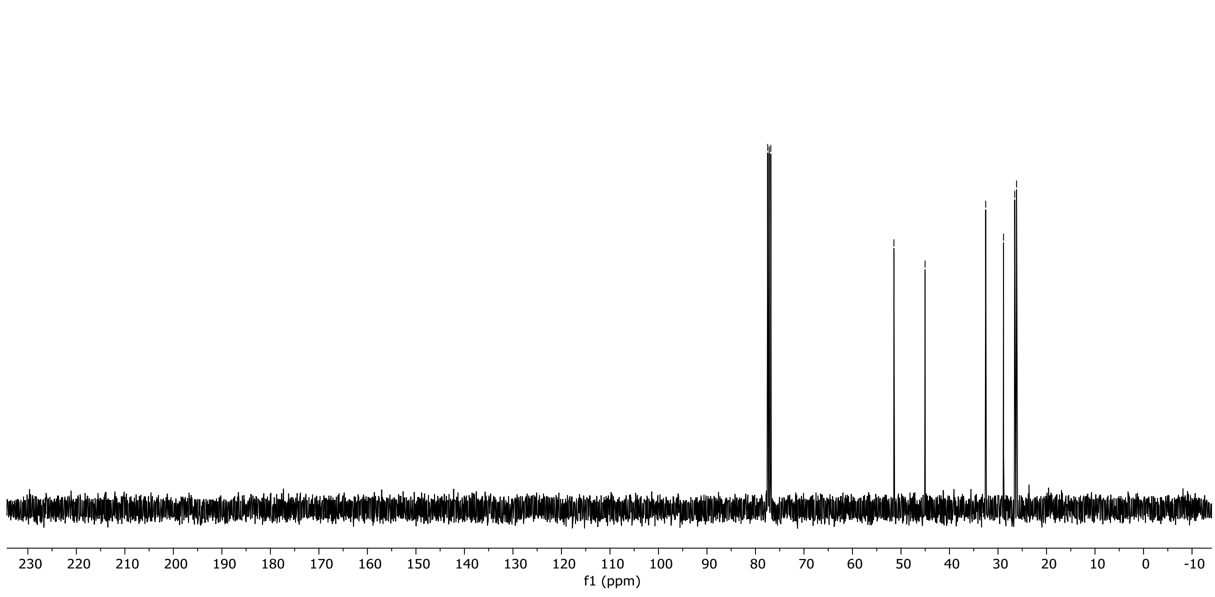

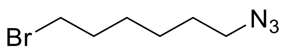

Compound **2d**

^{1}H NMR, 400 MHz

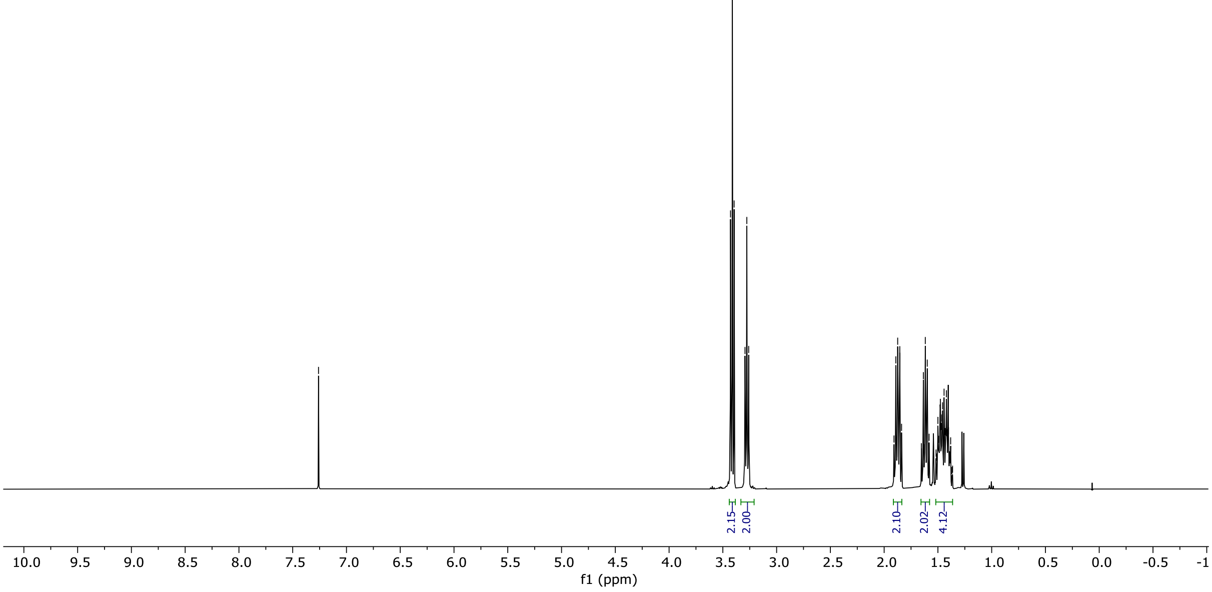

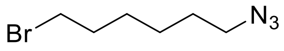

Compound **2d**

^{13}C NMR, 101 MHz

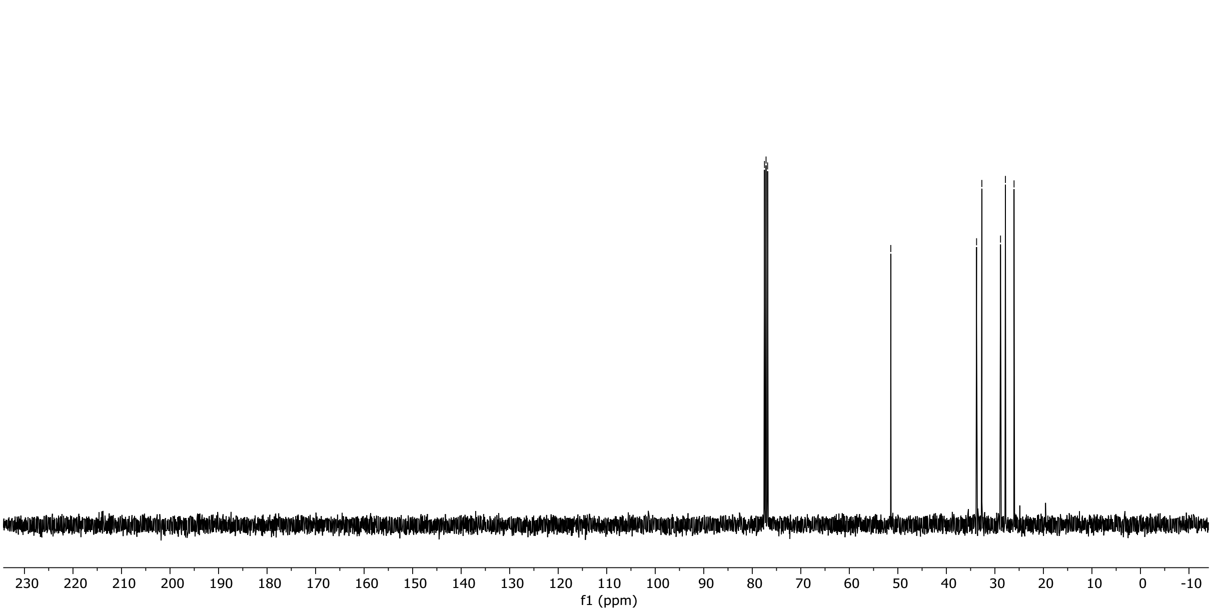

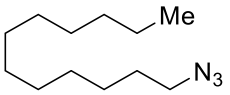

Compound **2e**
^{1}H NMR, 400 MHz

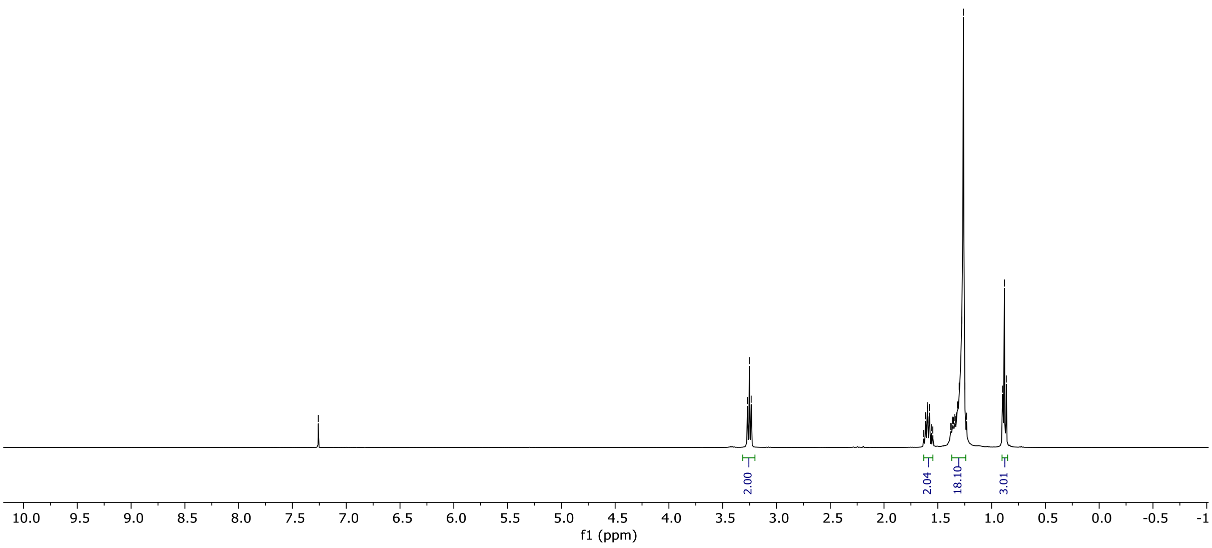

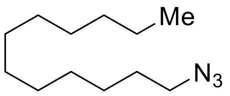

Compound **2e**
^{13}C NMR, 101 MHz

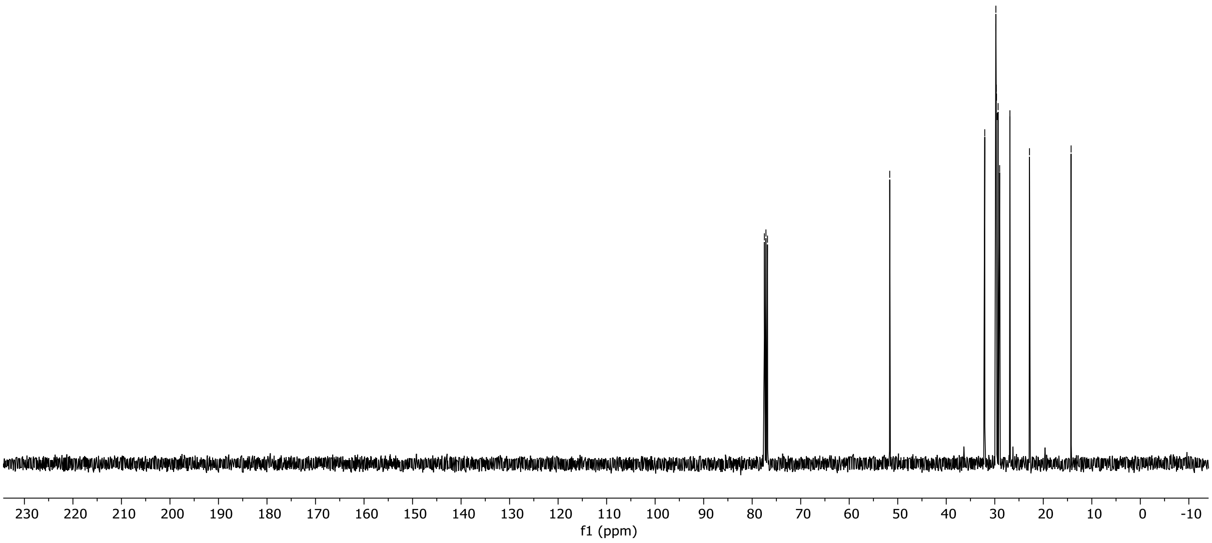

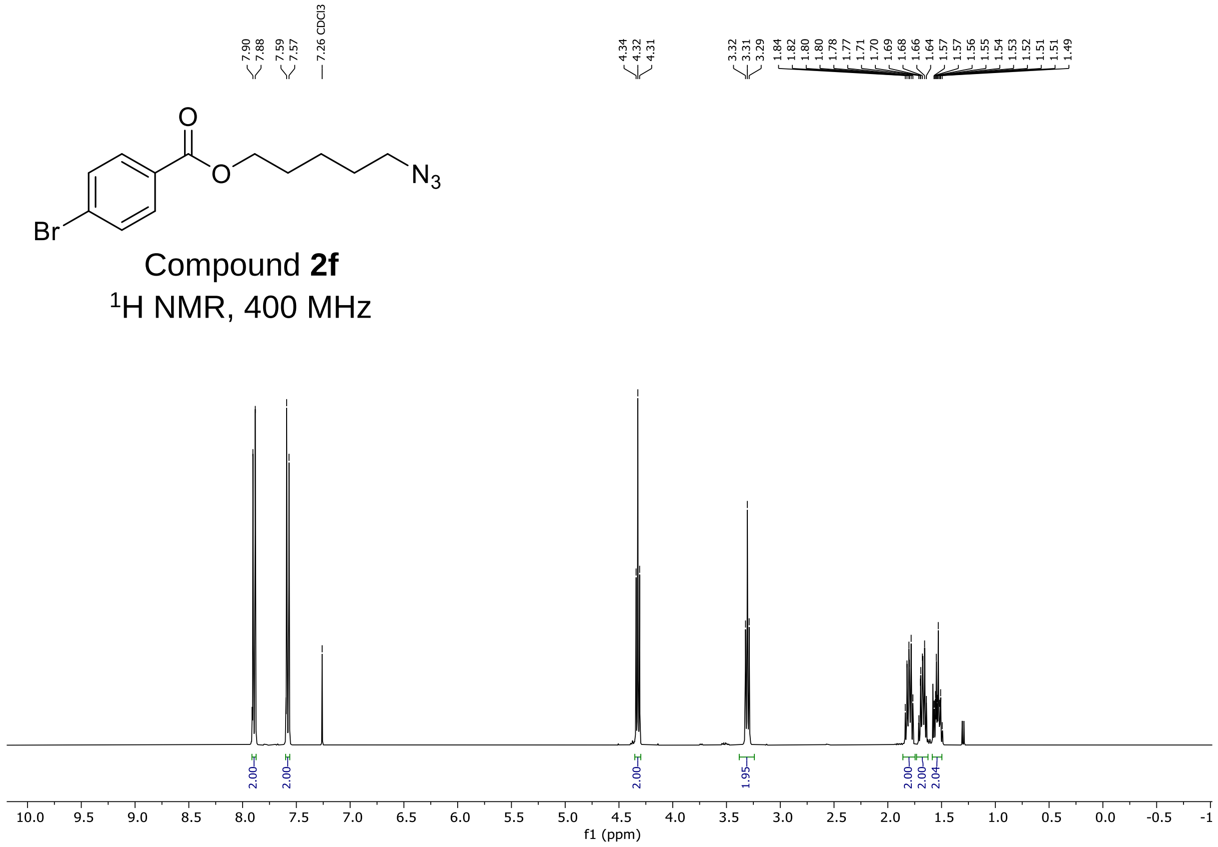
Compound **2f**
^{1}H NMR, 400 MHz
f1 (ppm)

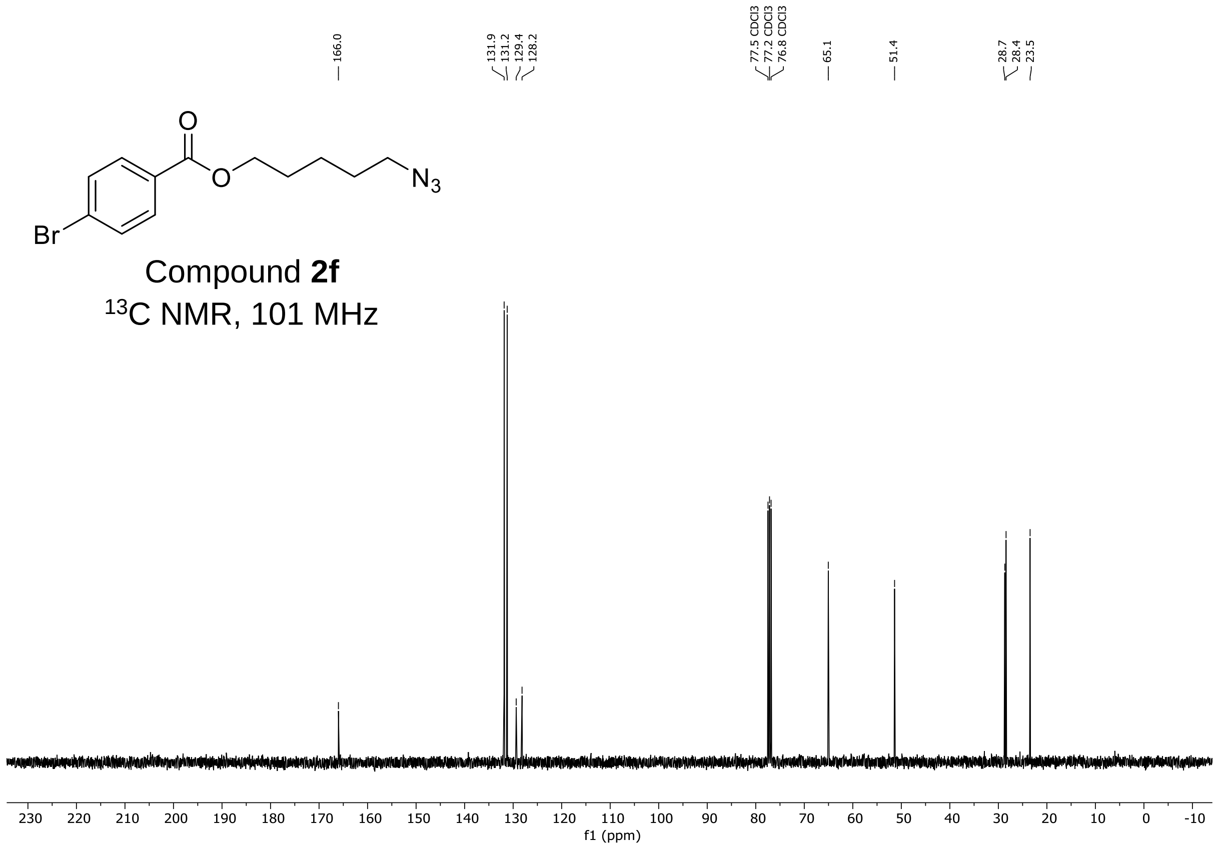
Compound **2f**
^{13}C NMR, 101 MHz
f1 (ppm)

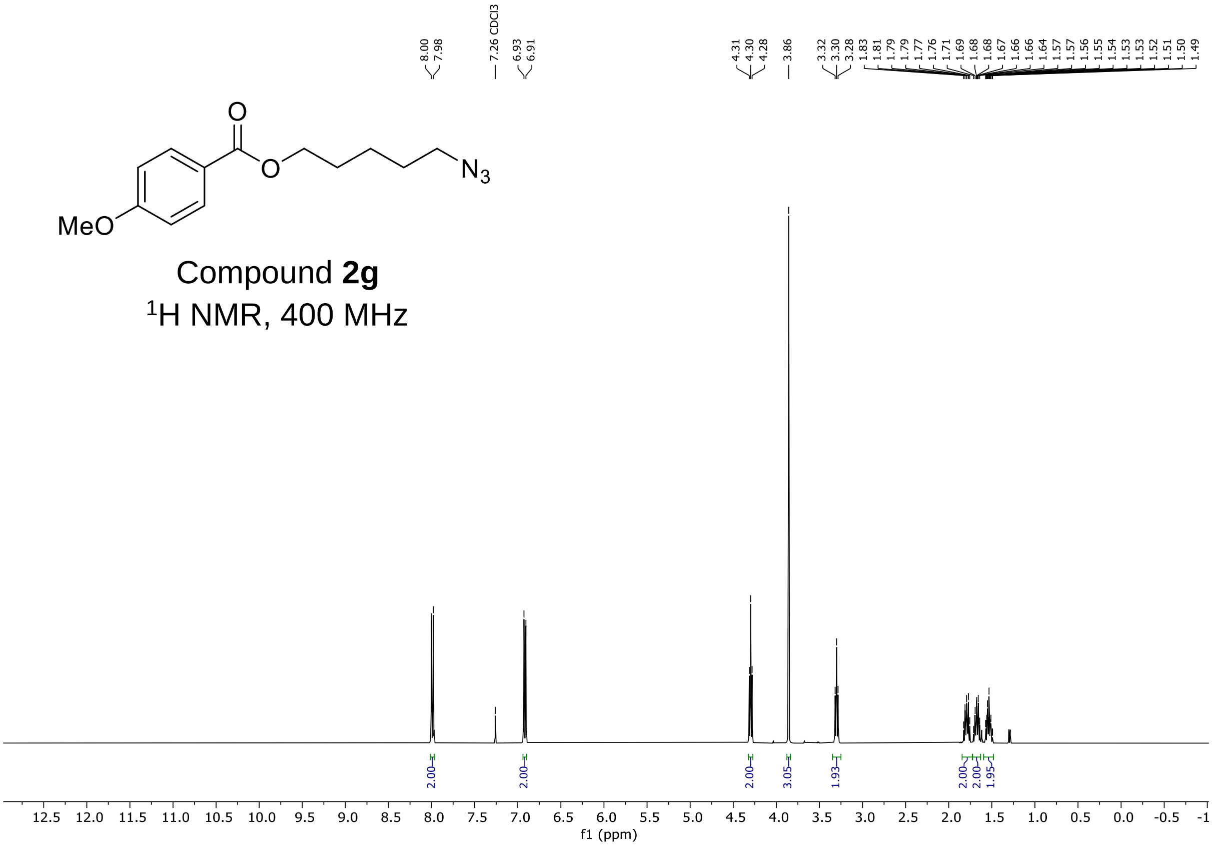
MeO
O
O
N3
Compound 2g
1H NMR, 400 MHz
8.00
7.98
7.26 CDCl3
6.93
6.91
4.31
4.30
4.28
3.86
3.32
3.30
3.28
2.00
2.00
2.00
3.05
1.93
2.00
2.00
1.95
f1 (ppm)

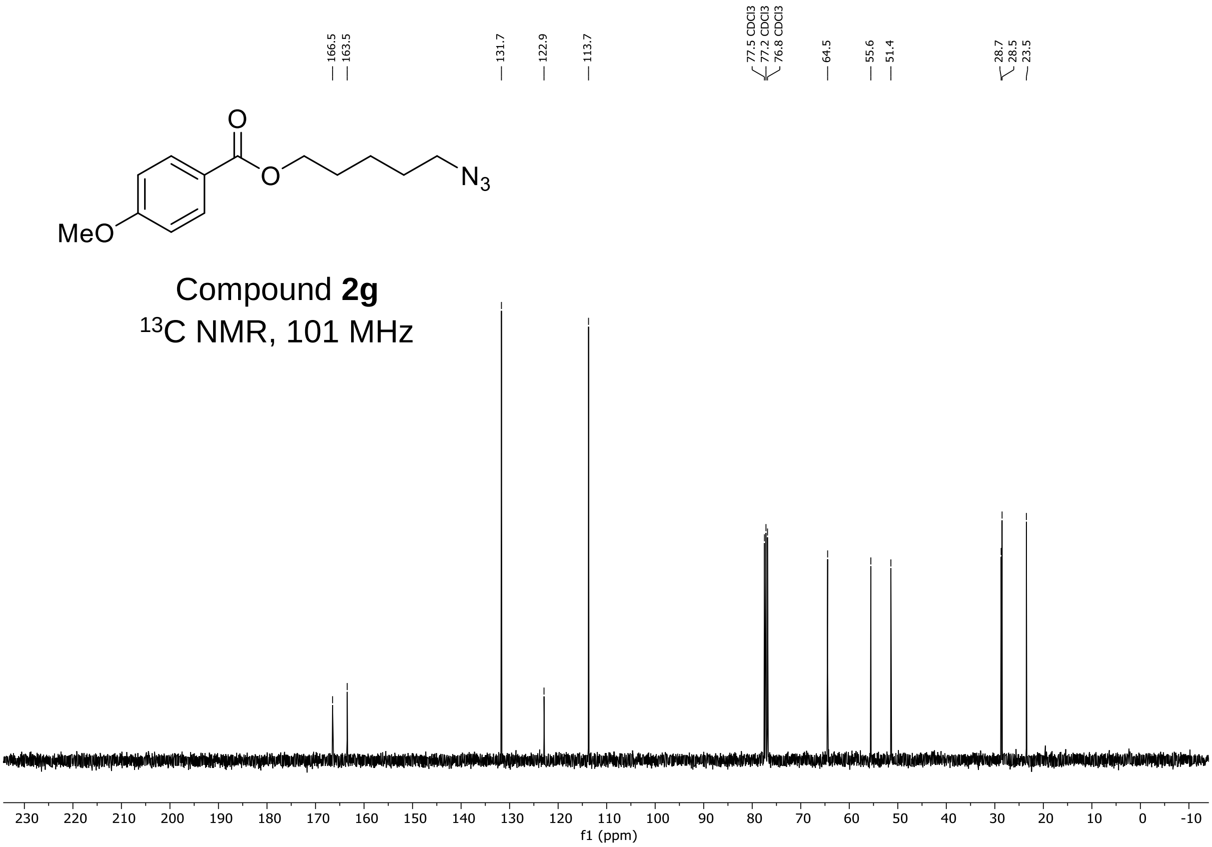
MeO
O
O
N3
Compound 2g
13C NMR, 101 MHz
166.5
163.5
131.7
122.9
113.7
77.5 CDCl3
77.2 CDCl3
76.8 CDCl3
64.5
55.6
51.4
28.7
28.5
23.5
f1 (ppm)

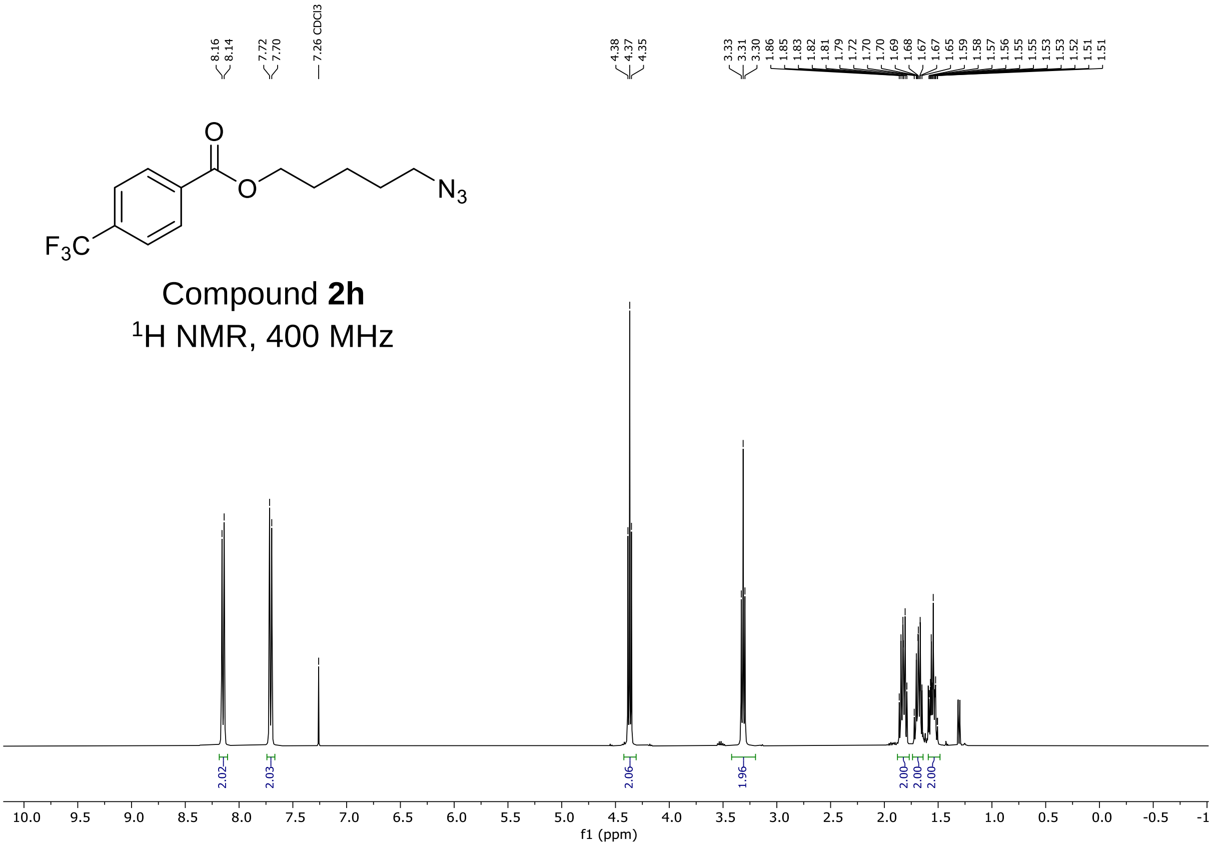

Compound **2h**
^{1}H NMR, 400 MHz

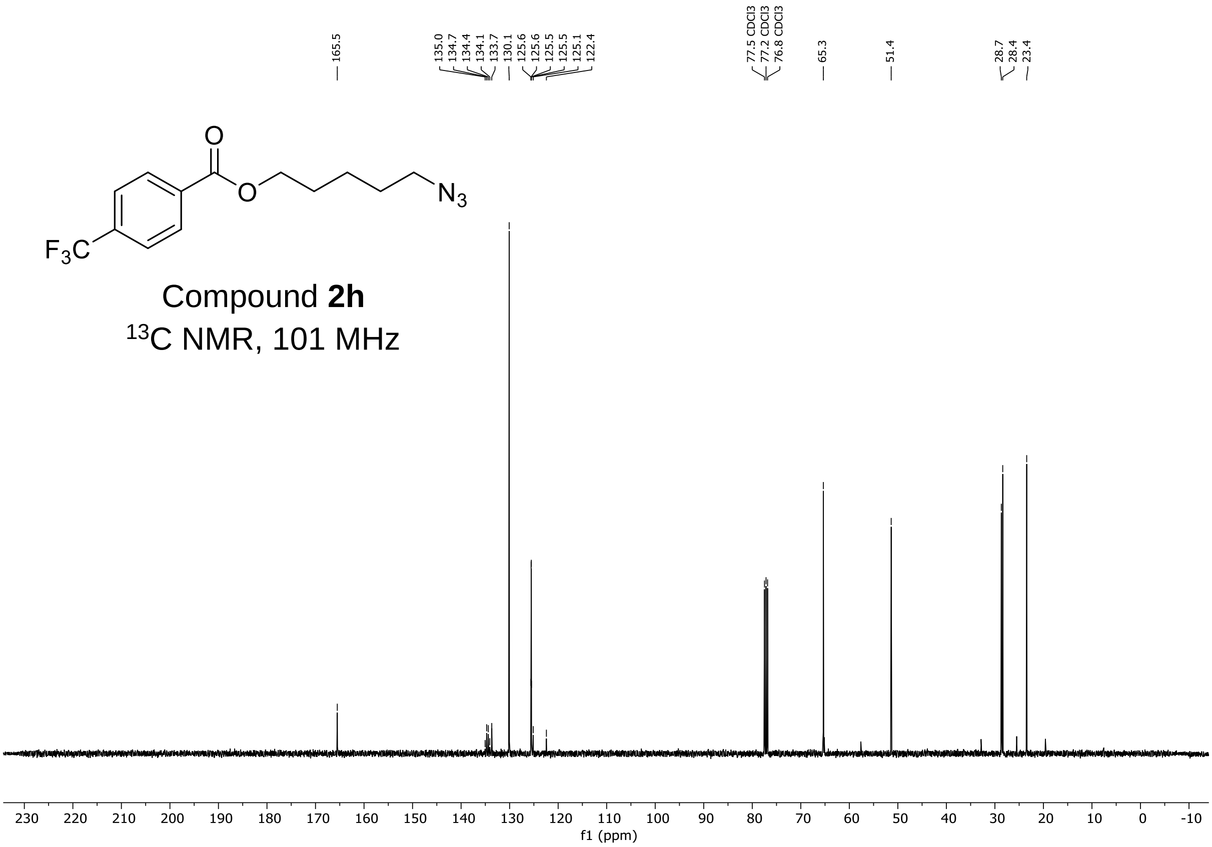

Compound **2h**
^{13}C NMR, 101 MHz

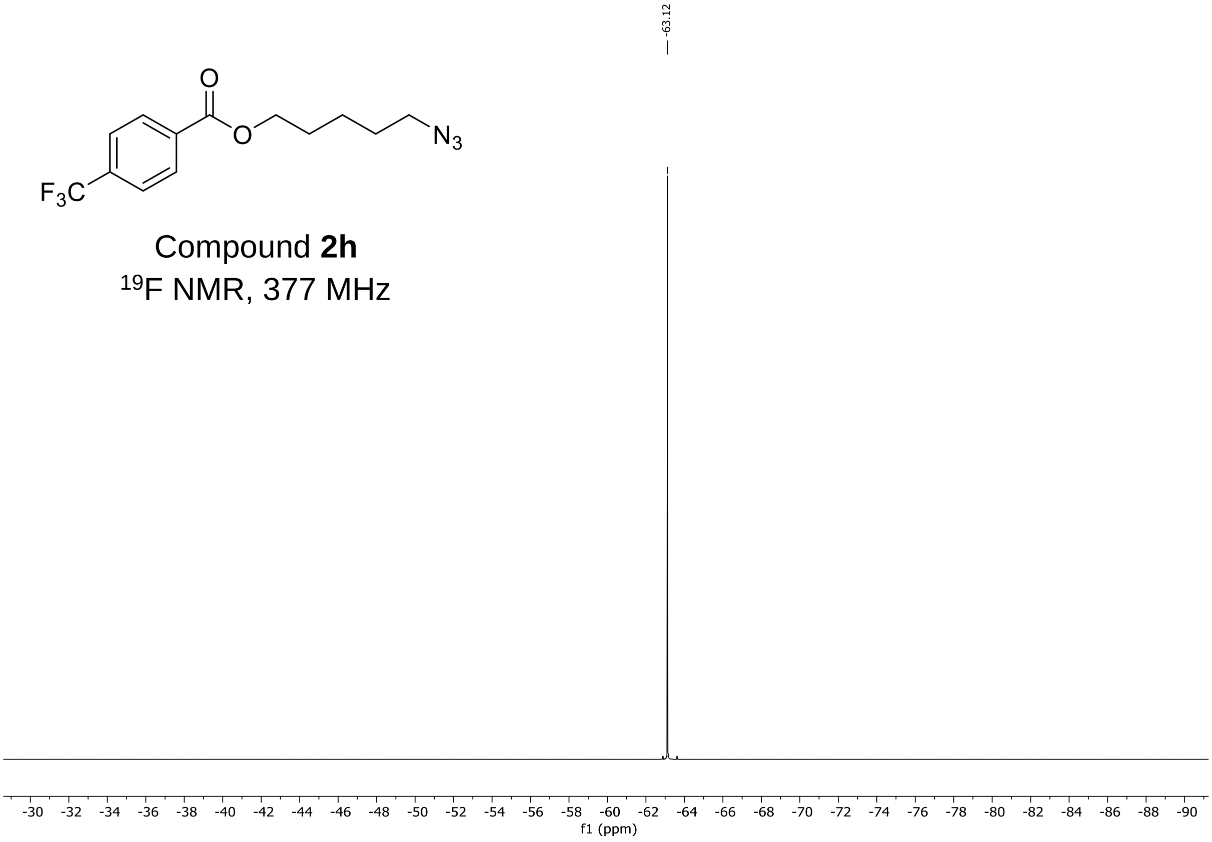

Compound **2h**

^{19}F NMR, 377 MHz

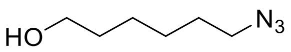

Compound **2i**
^{1}H NMR, 400 MHz

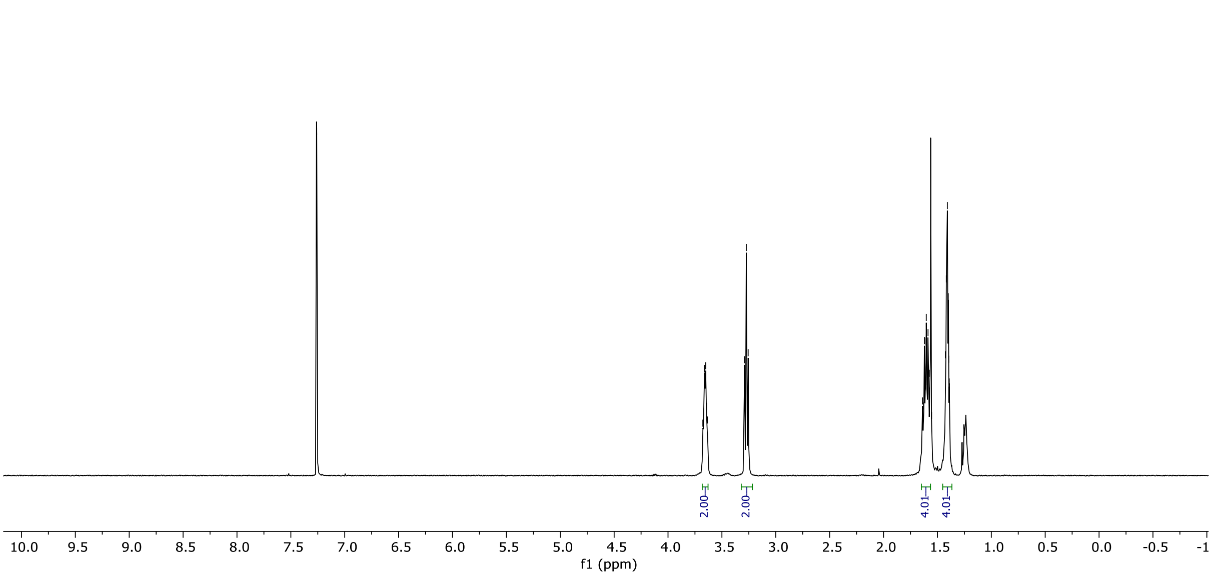

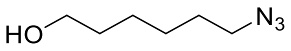

Compound **2i**
^{13}C NMR, 101 MHz

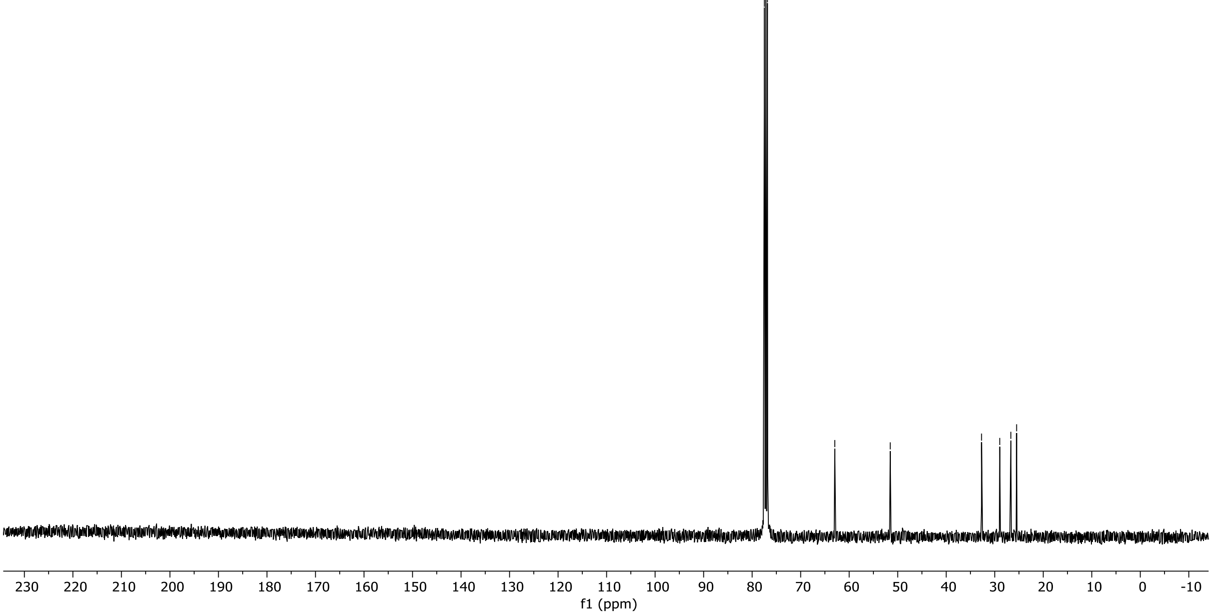

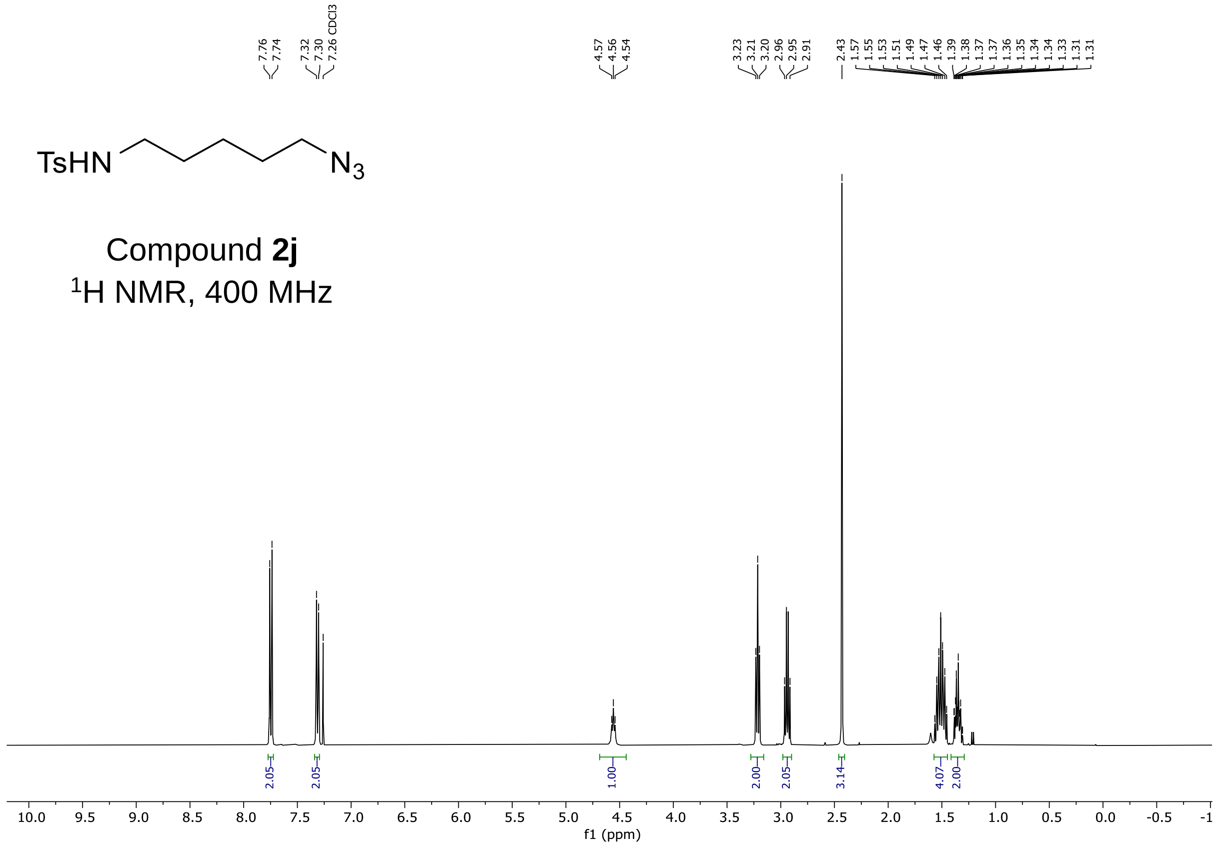
7.76
7.74
7.32
7.30
7.26 CDCl3
4.57
4.56
4.54
3.23
3.21
3.20
2.96
2.95
2.91
2.43
1.57
1.55
1.53
1.51
1.49
1.47
1.46
1.39
1.38
1.37
1.37
1.36
1.35
1.34
1.34
1.33
1.31
1.31
TsHN
N3
Compound 2j
1H NMR, 400 MHz
2.05
2.05
1.00
2.00
2.05
3.14
4.07
2.00
f1 (ppm)

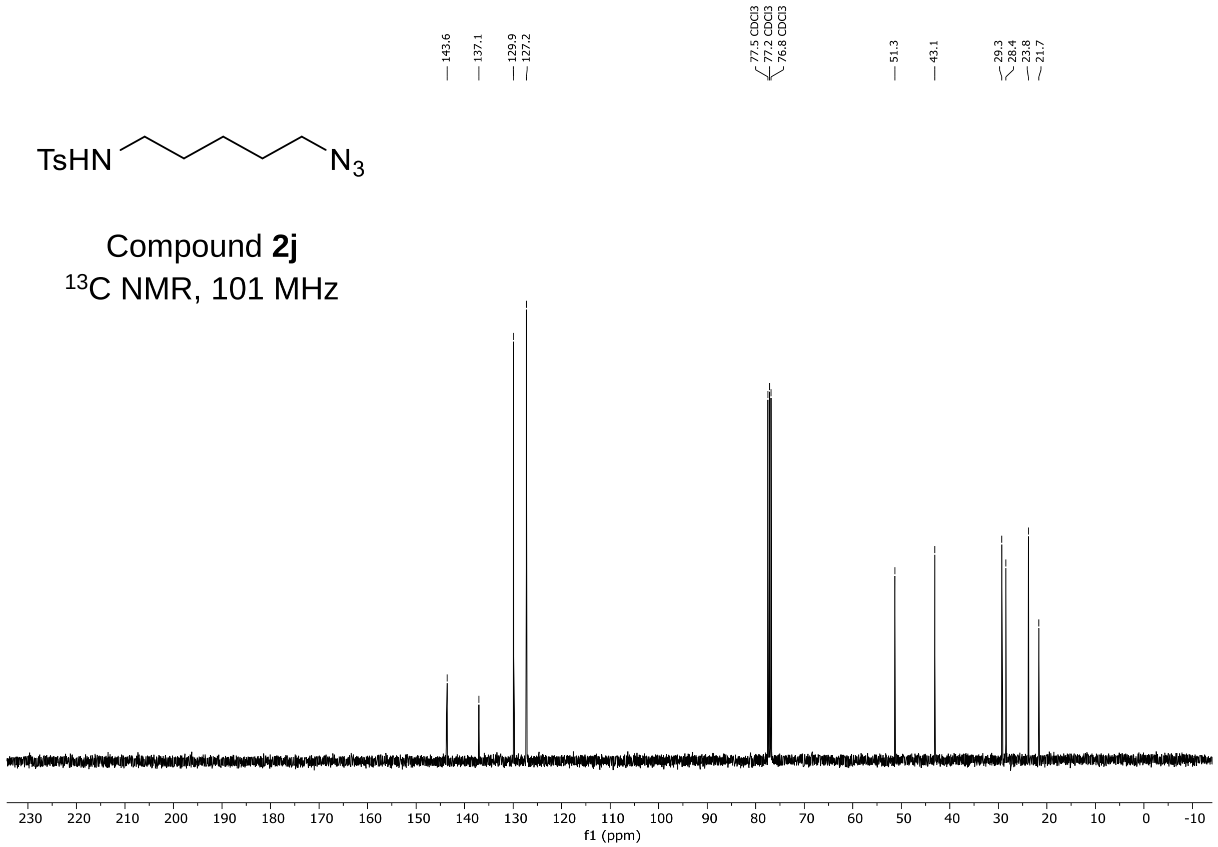
143.6
137.1
129.9
127.2
77.5 CDCl3
77.2 CDCl3
76.8 CDCl3
51.3
43.1
29.3
28.4
23.8
21.7
TsHN
N3
Compound 2j
13C NMR, 101 MHz
f1 (ppm)

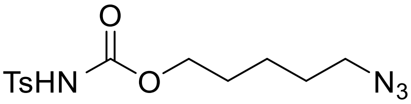

Compound **2k**
^{1}H NMR, 400 MHz

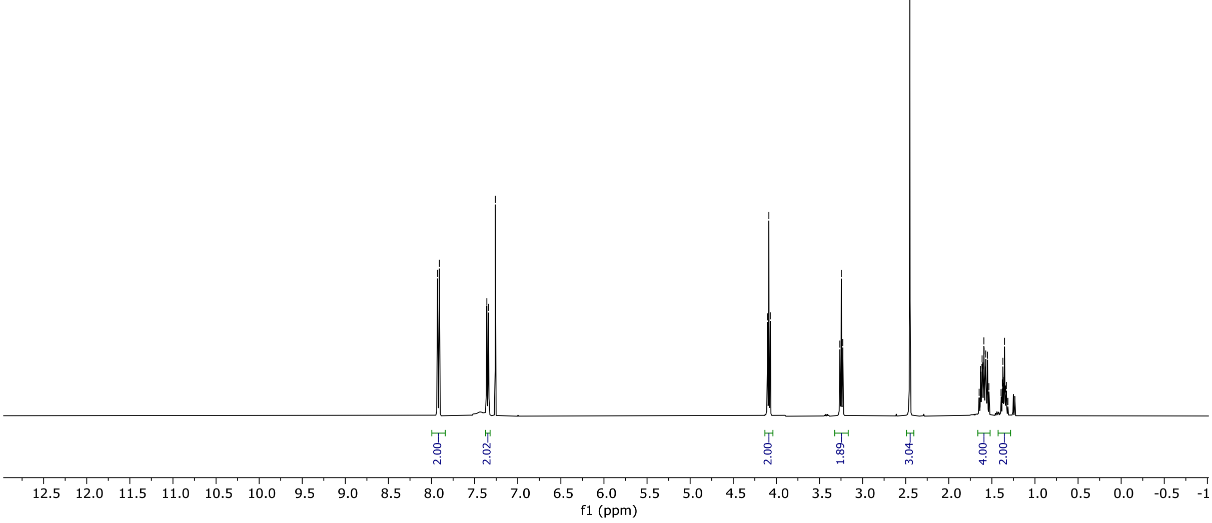

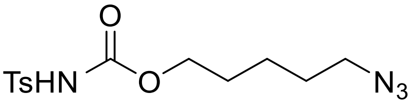

Compound **2k**
^{13}C NMR, 101 MHz

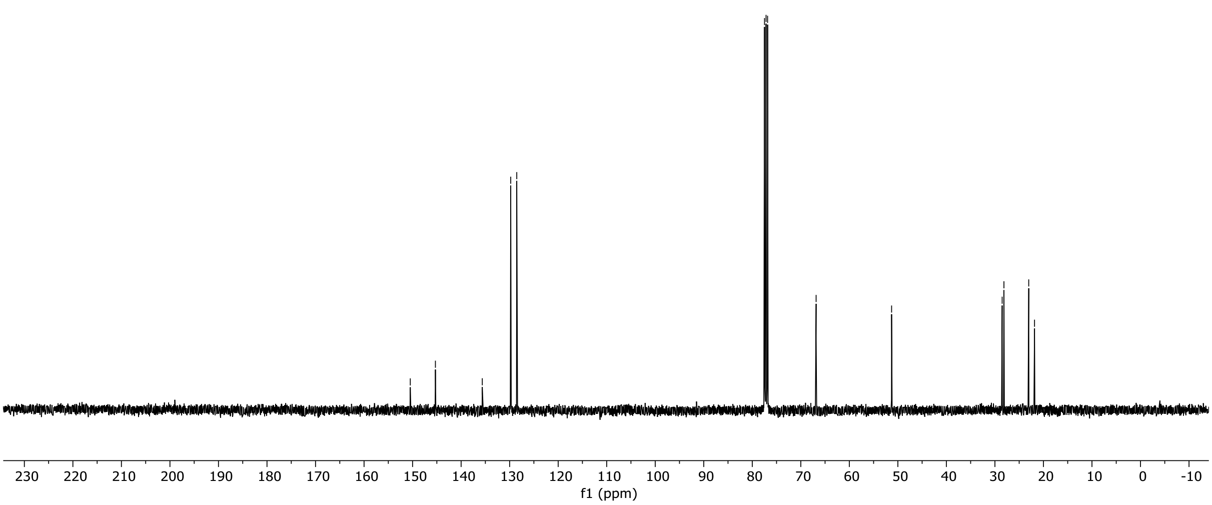

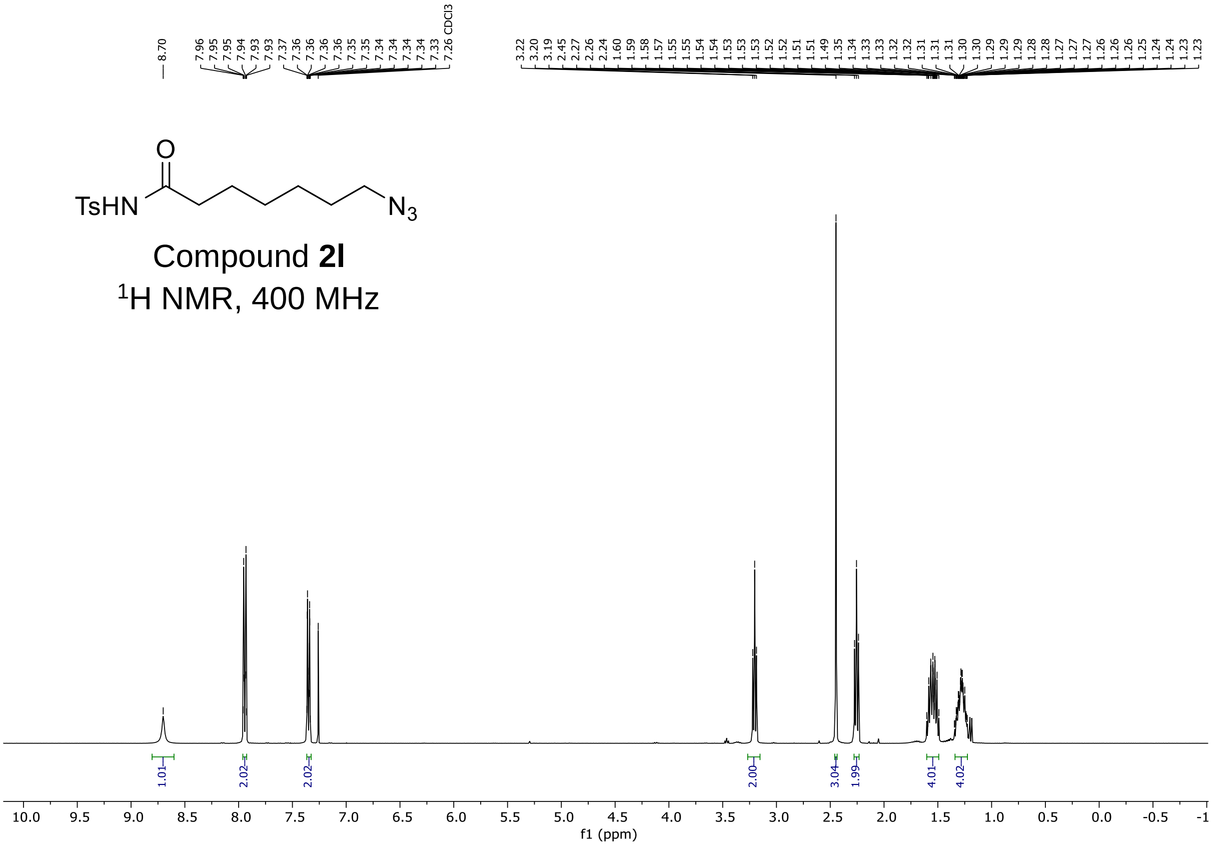

Compound **2l**
^{1}H NMR, 400 MHz

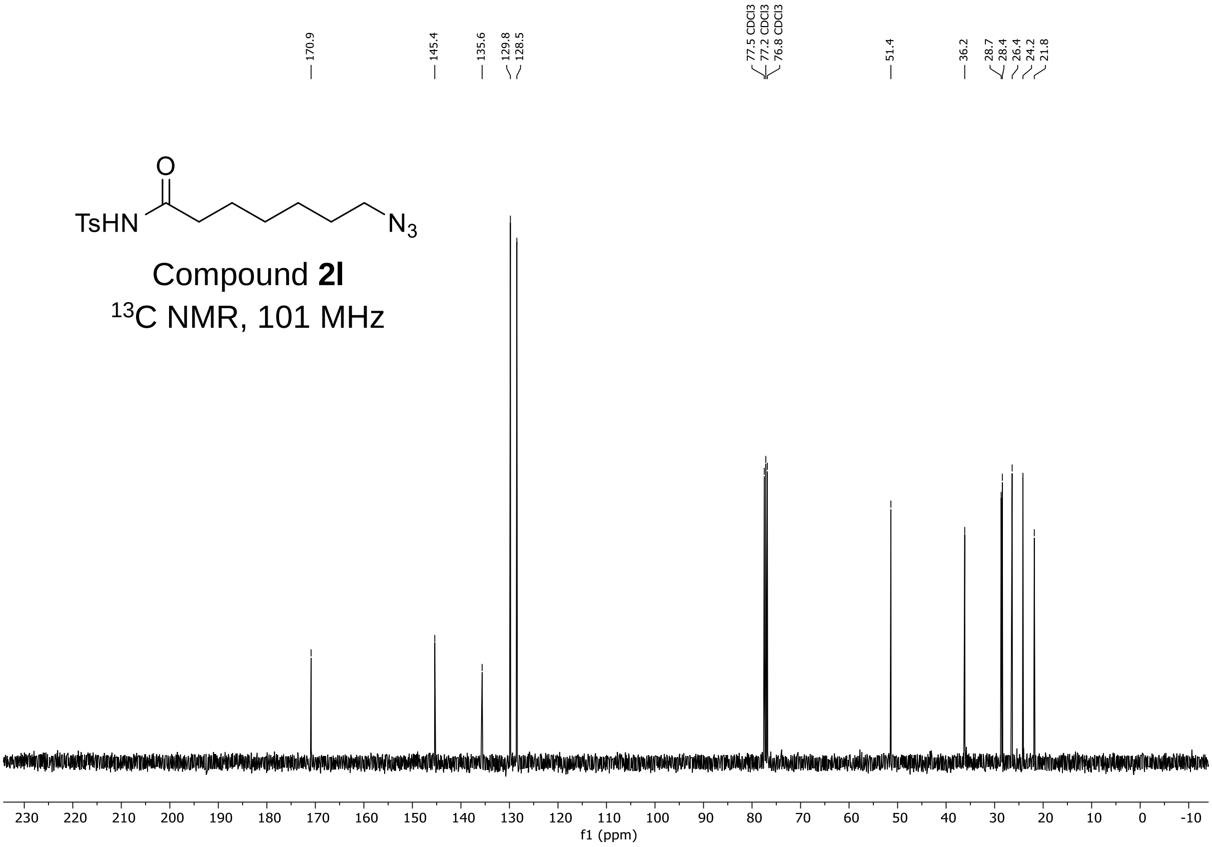

Compound **2l**
^{13}C NMR, 101 MHz

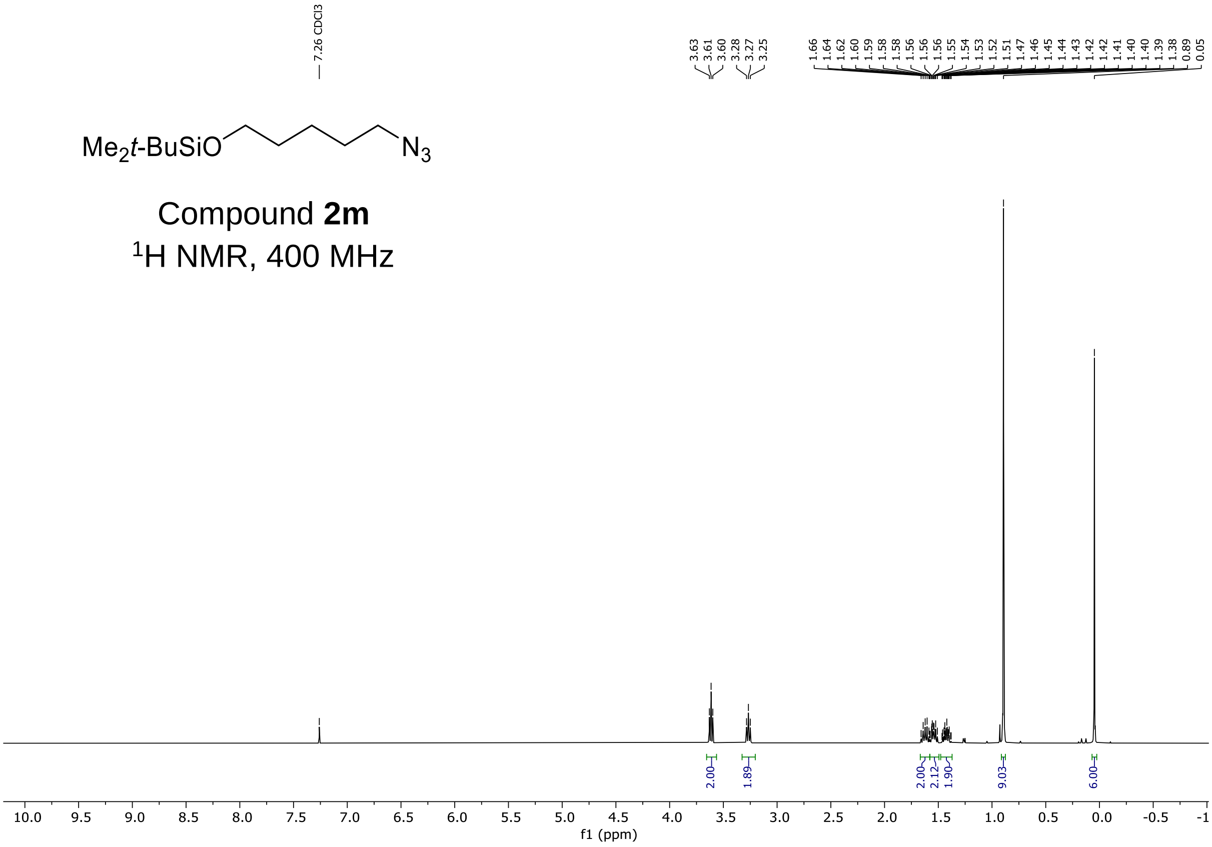
Me2t-BuSiO
N3
Compound 2m
1H NMR, 400 MHz
7.26 CDCl3
f1 (ppm)

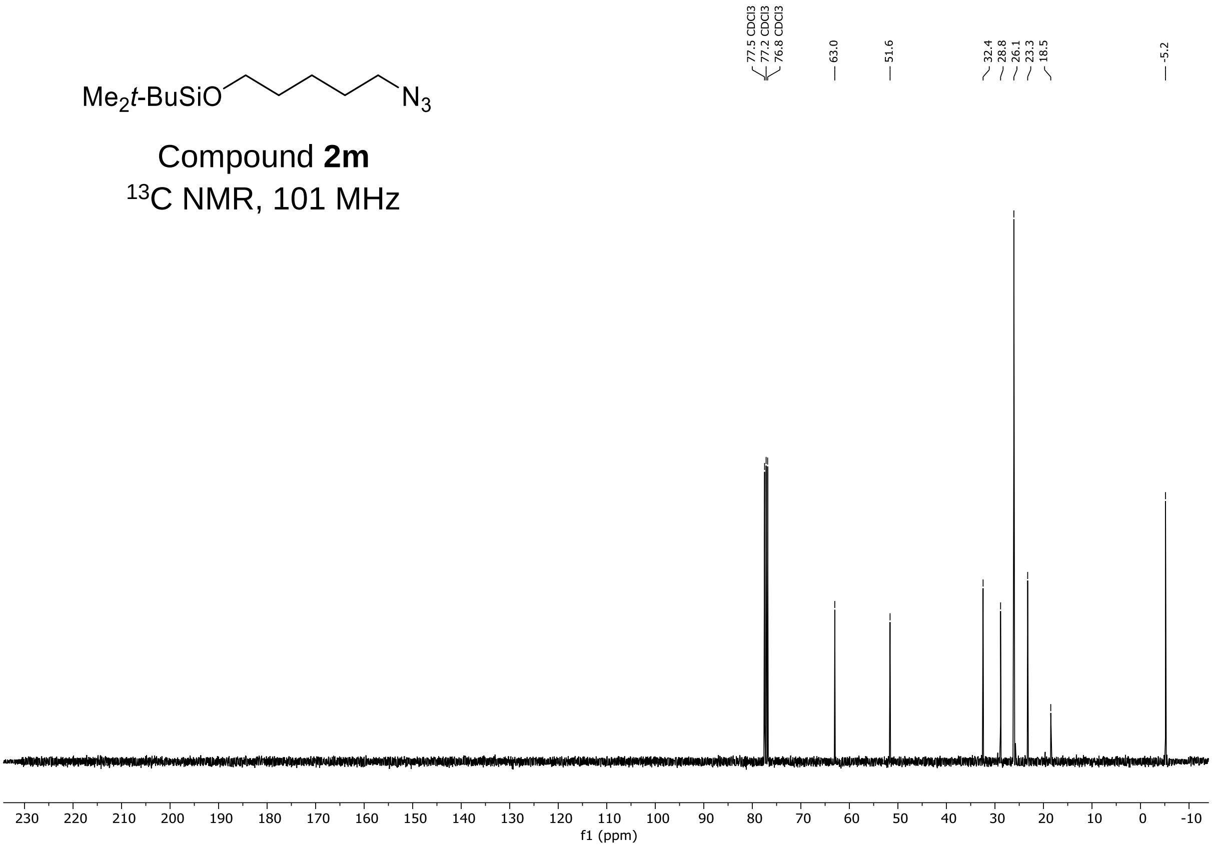
Me2t-BuSiO
N3
Compound 2m
13C NMR, 101 MHz
77.5 CDCl3
77.2 CDCl3
76.8 CDCl3
63.0
51.6
32.4
28.8
26.1
23.3
18.5
-5.2
f1 (ppm)

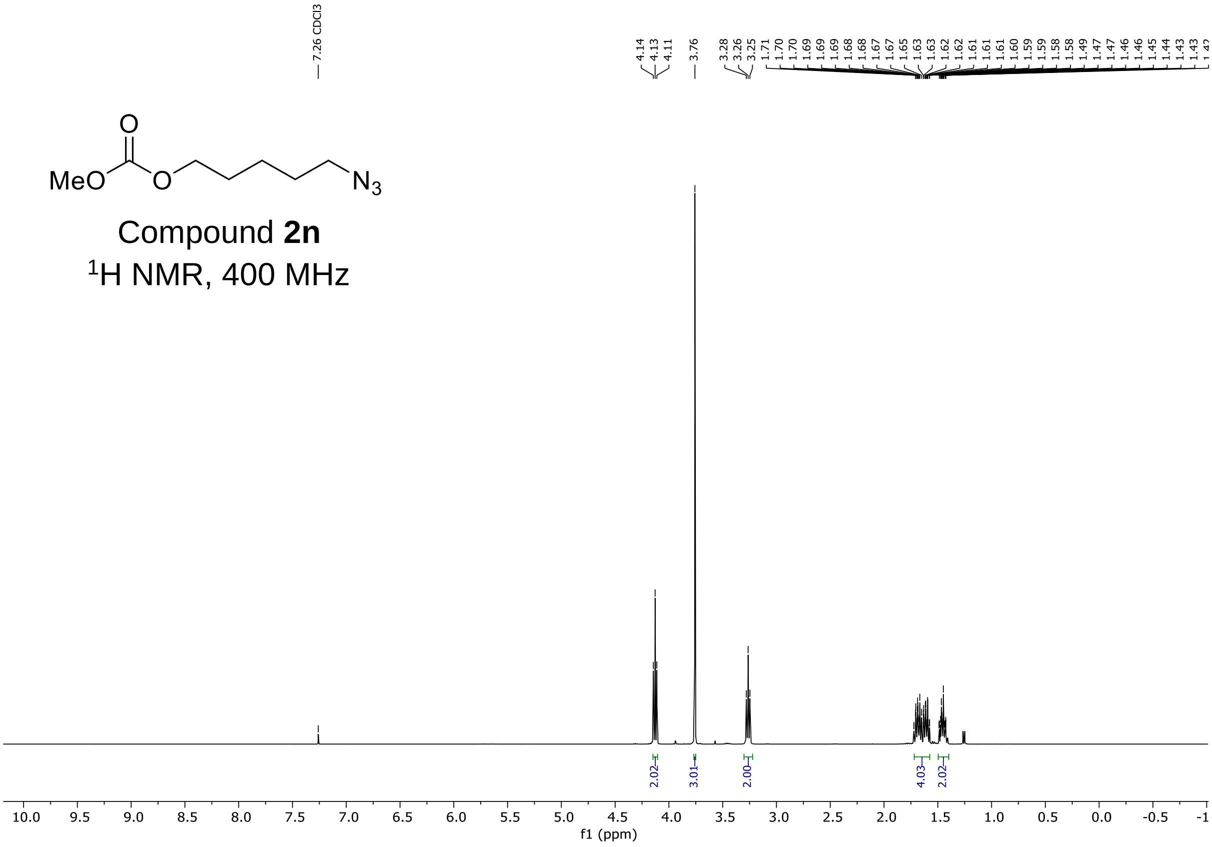
Compound **2n**
^{1}H NMR, 400 MHz
7.26 CDCl3
4.14
4.13
4.11
3.76
3.28
3.26
3.25
2.02
3.01
2.00
4.03
2.02
f1 (ppm)

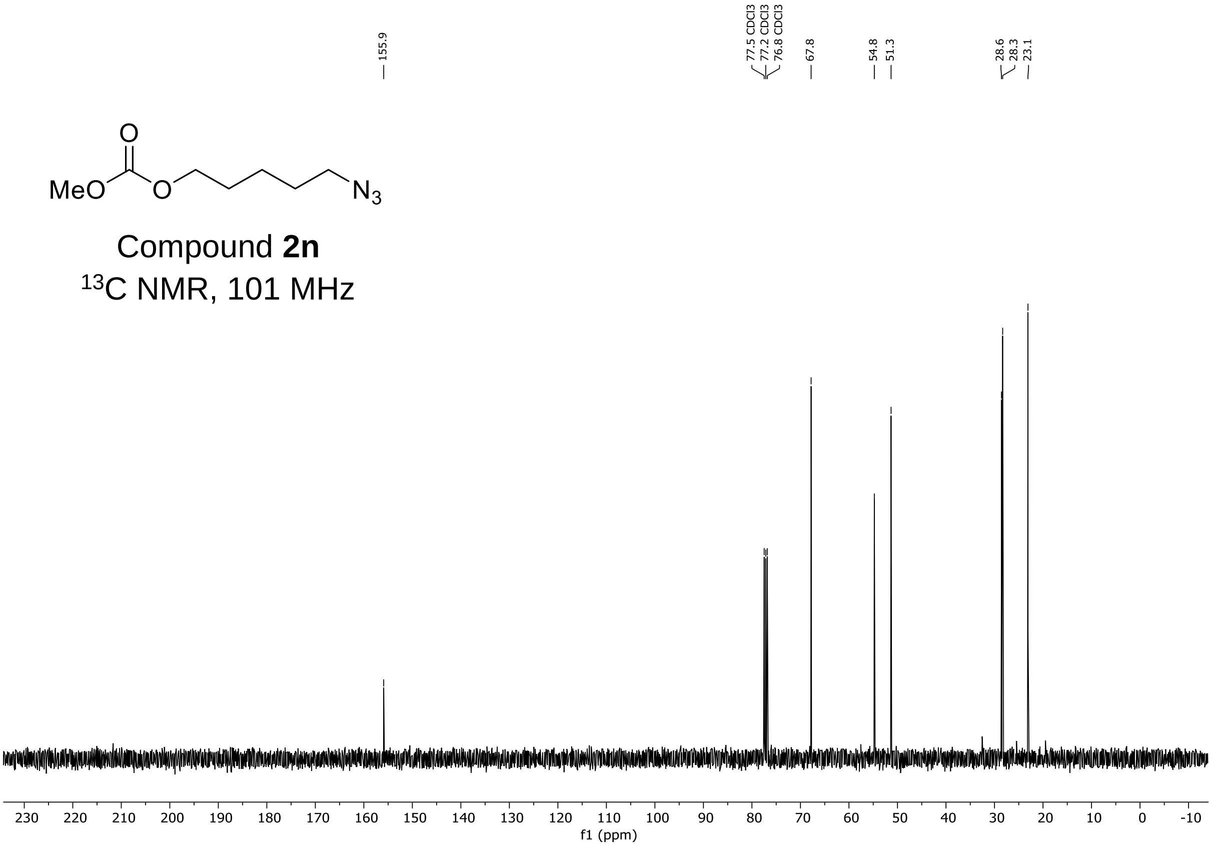
Compound **2n**
^{13}C NMR, 101 MHz
155.9
77.5 CDCl3
77.2 CDCl3
76.8 CDCl3
67.8
54.8
51.3
28.6
28.3
23.1
f1 (ppm)

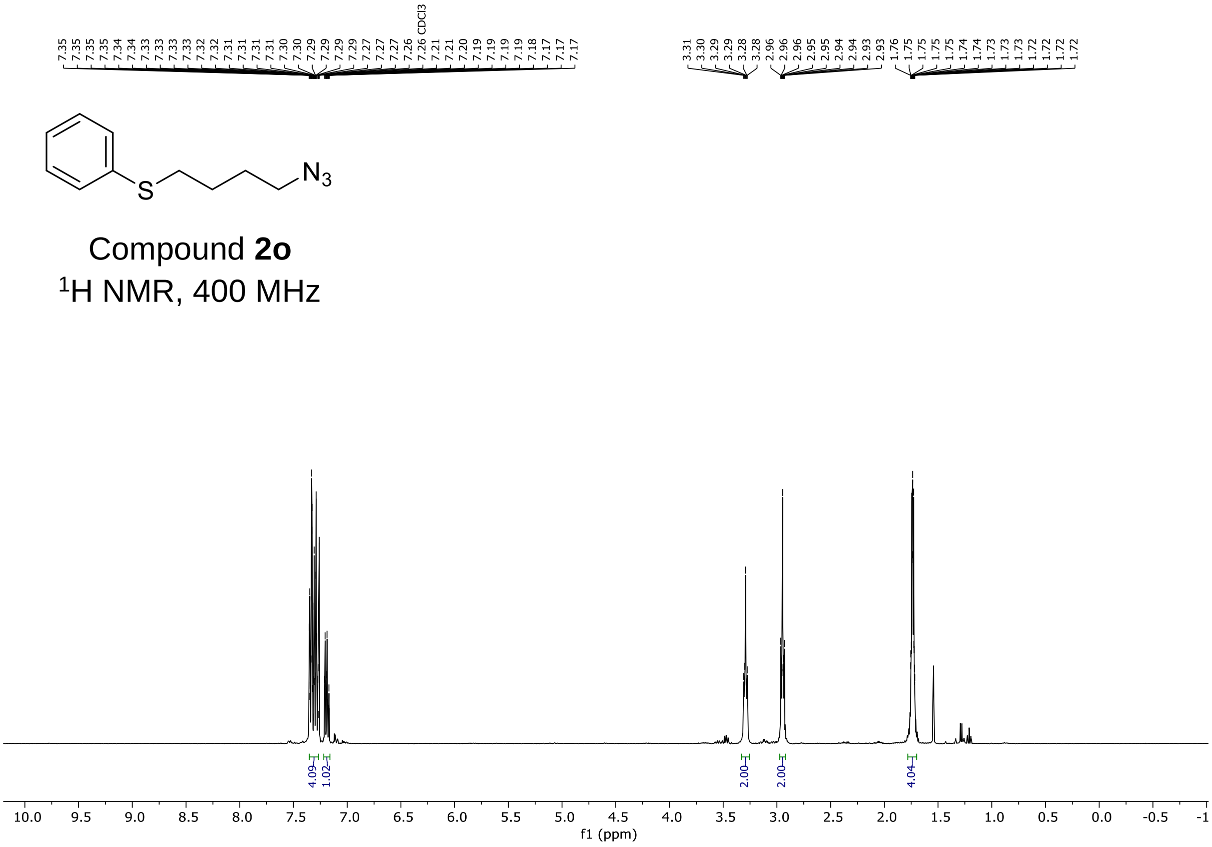

7.35 7.35 7.35 7.35 7.34 7.34 7.33 7.33 7.33 7.33 7.32 7.32 7.31 7.31 7.31 7.31 7.30 7.30 7.29 7.29 7.29 7.29 7.27 7.27 7.27 7.26 CDCl3 7.21 7.21 7.20 7.19 7.19 7.19 7.19 7.18 7.17 7.17 7.17
3.31 3.30 3.29 3.29 3.28 3.28 2.96 2.96 2.96 2.95 2.95 2.94 2.94 2.93 2.93 1.76 1.75 1.75 1.75 1.75 1.74 1.74 1.73 1.73 1.73 1.73 1.72 1.72 1.72 1.72
S
N3
Compound **2o**
^{1}H NMR, 400 MHz
4.09 1.02 2.00 2.00 4.04
f1 (ppm)

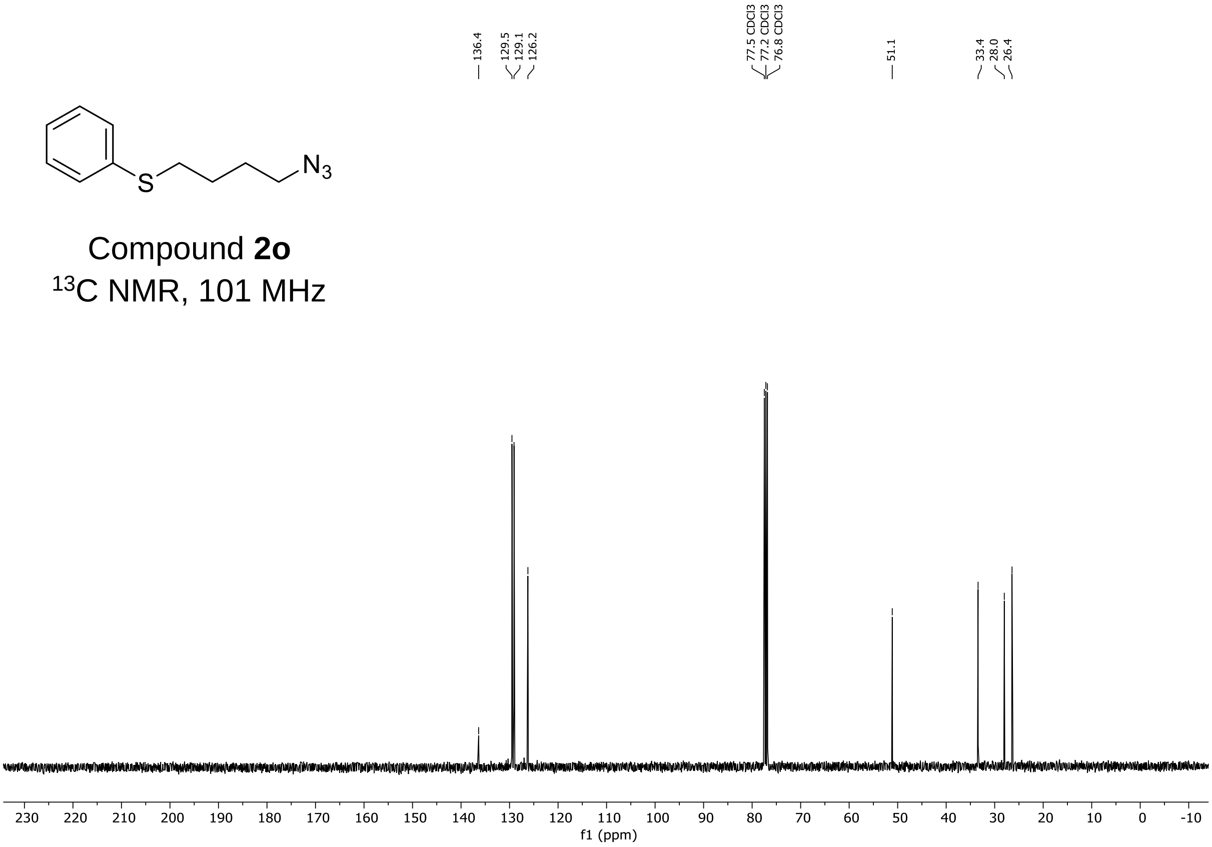

136.4 129.5 129.1 126.2 77.5 CDCl3 77.2 CDCl3 76.8 CDCl3 51.1 33.4 28.0 26.4
S
N3
Compound **2o**
^{13}C NMR, 101 MHz
f1 (ppm)

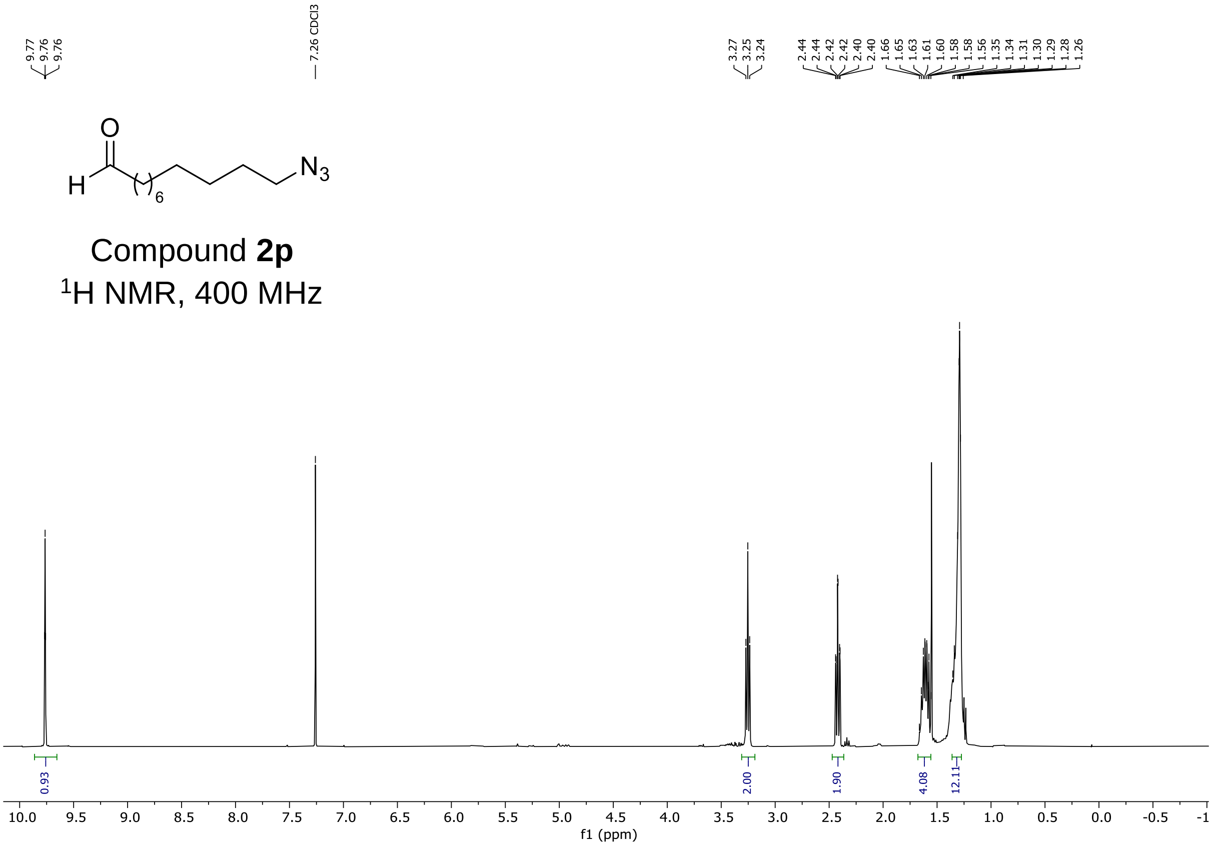

Compound **2p**
^{1}H NMR, 400 MHz

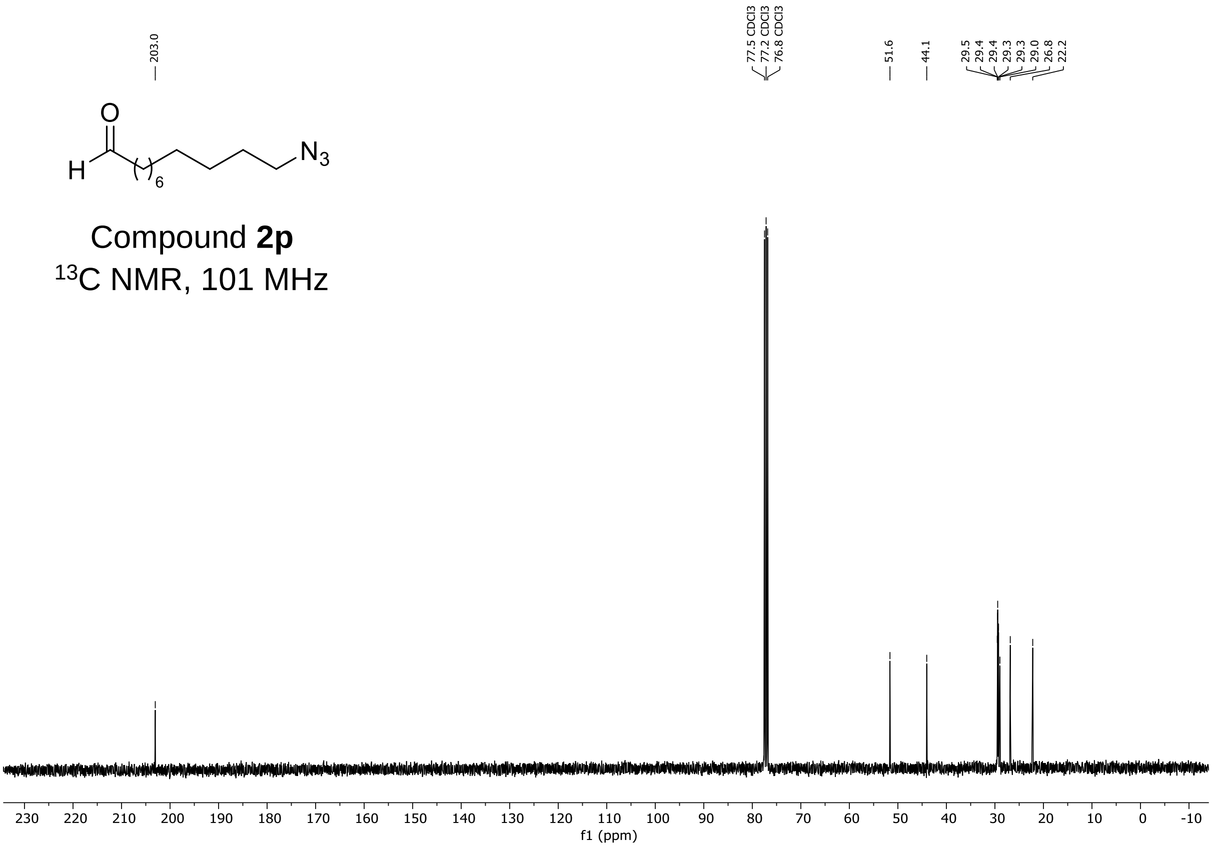

Compound **2p**
^{13}C NMR, 101 MHz

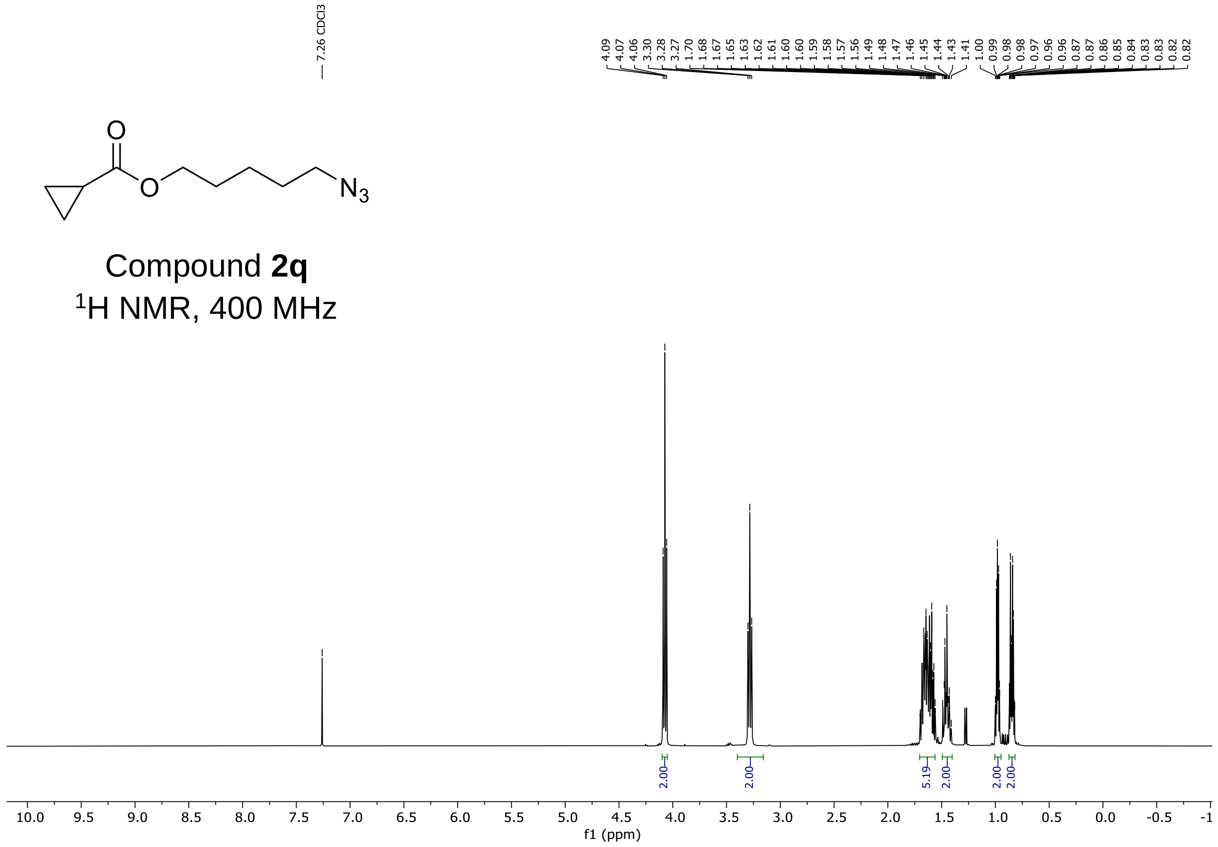

Compound **2q**
^{1}H NMR, 400 MHz

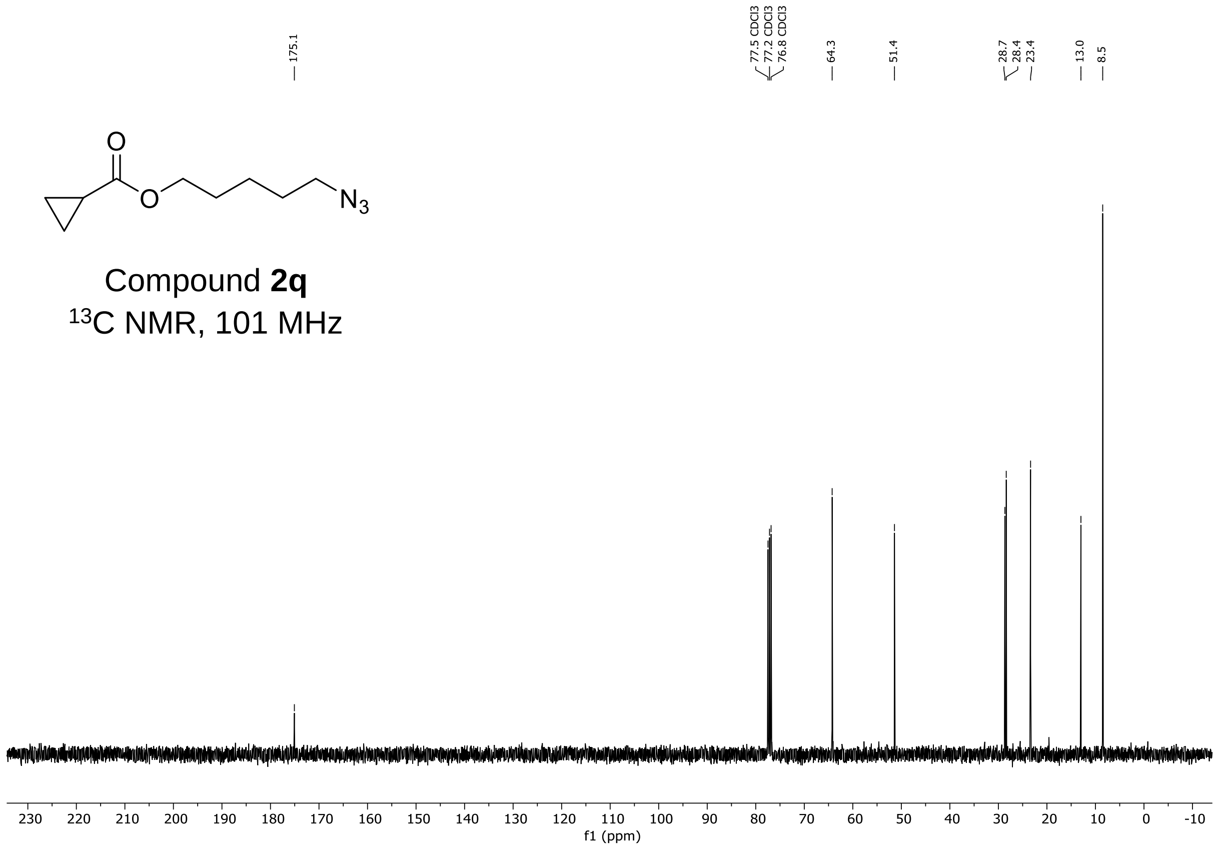

Compound **2q**
^{13}C NMR, 101 MHz

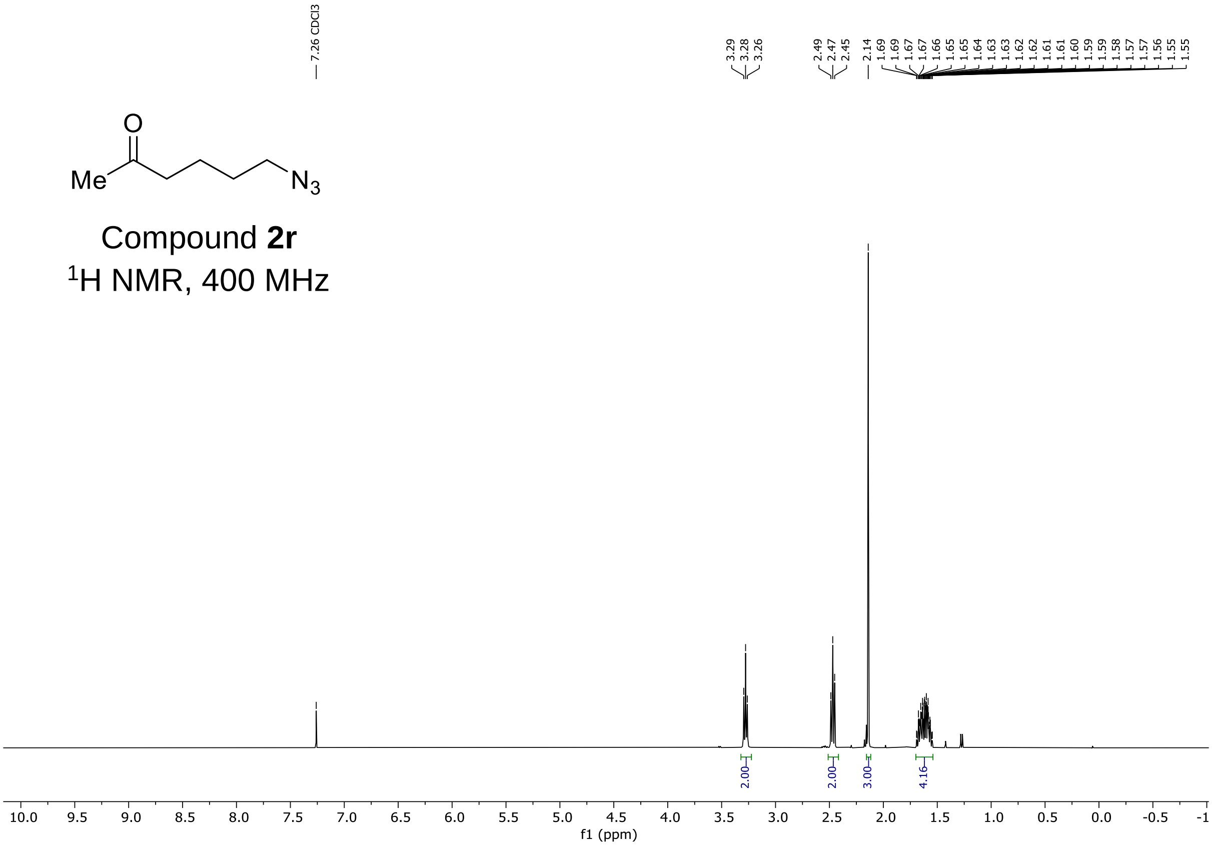
7.26 CDCl3
3.29
3.28
3.26
2.49
2.47
2.45
2.14
1.69
1.69
1.67
1.67
1.66
1.65
1.65
1.64
1.63
1.63
1.62
1.62
1.61
1.61
1.60
1.59
1.59
1.58
1.57
1.57
1.56
1.55
1.55
O
Me
N3
Compound 2r
1H NMR, 400 MHz
2.00
2.00
3.00
4.16
f1 (ppm)

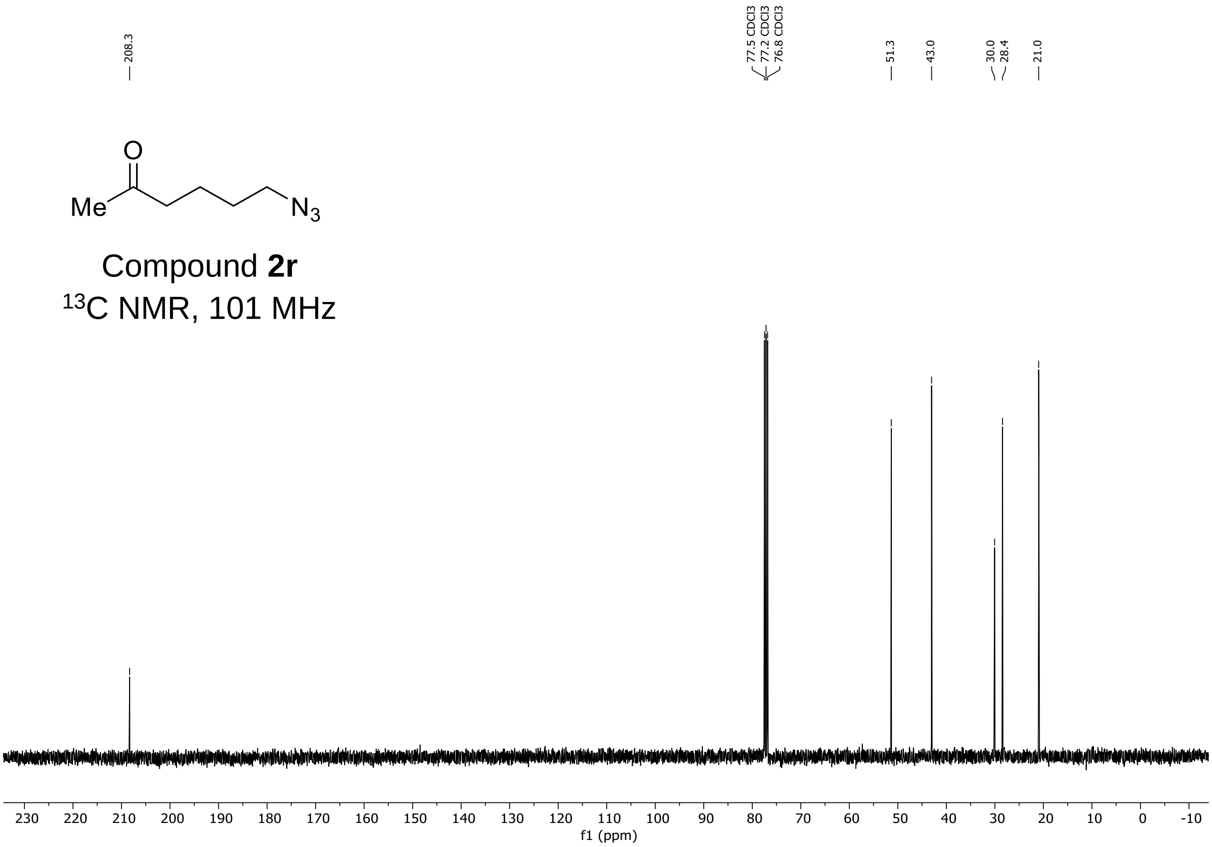
208.3
77.5 CDCl3
77.2 CDCl3
76.8 CDCl3
51.3
43.0
30.0
28.4
21.0
O
Me
N3
Compound 2r
13C NMR, 101 MHz
f1 (ppm)

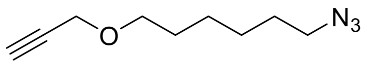

Compound **2s**
^{1}H NMR, 400 MHz

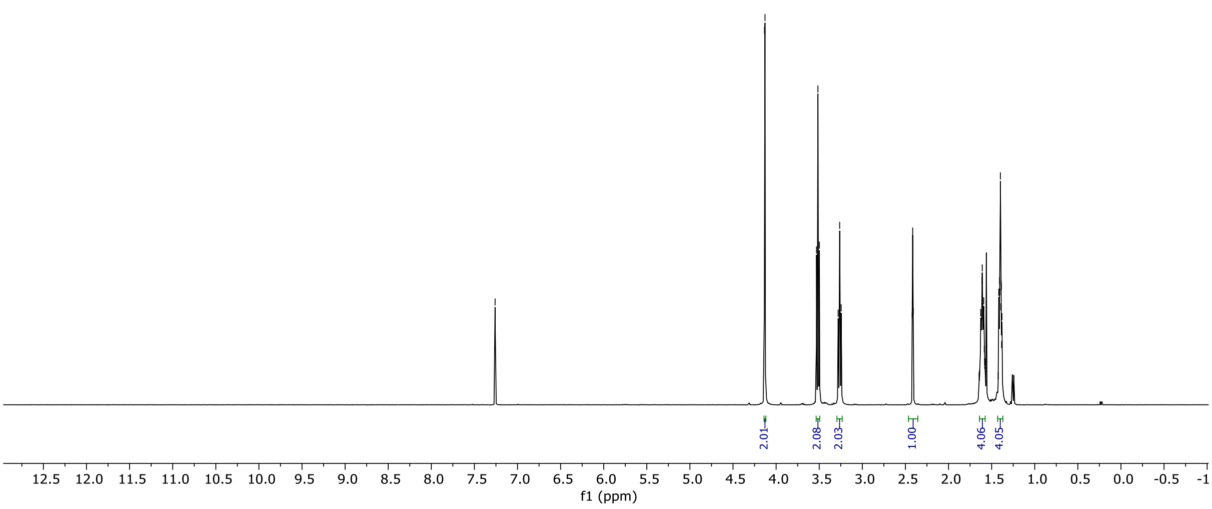

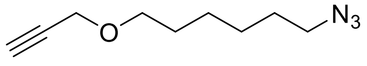

Compound **2s**
^{13}C NMR, 101 MHz

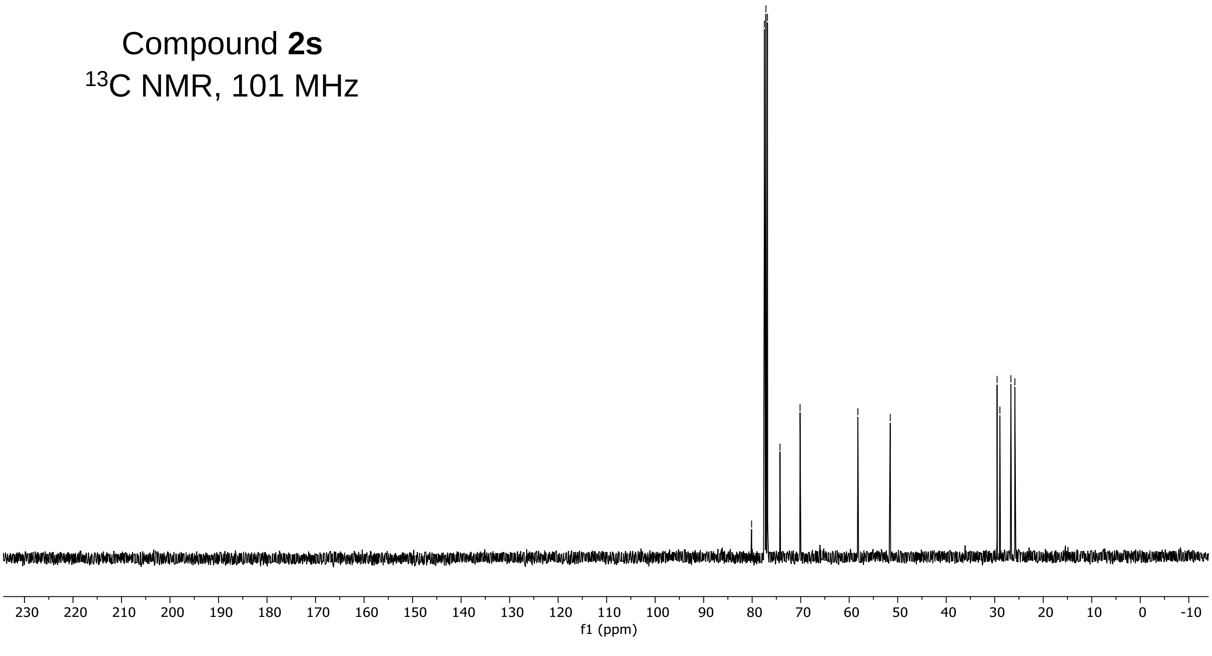

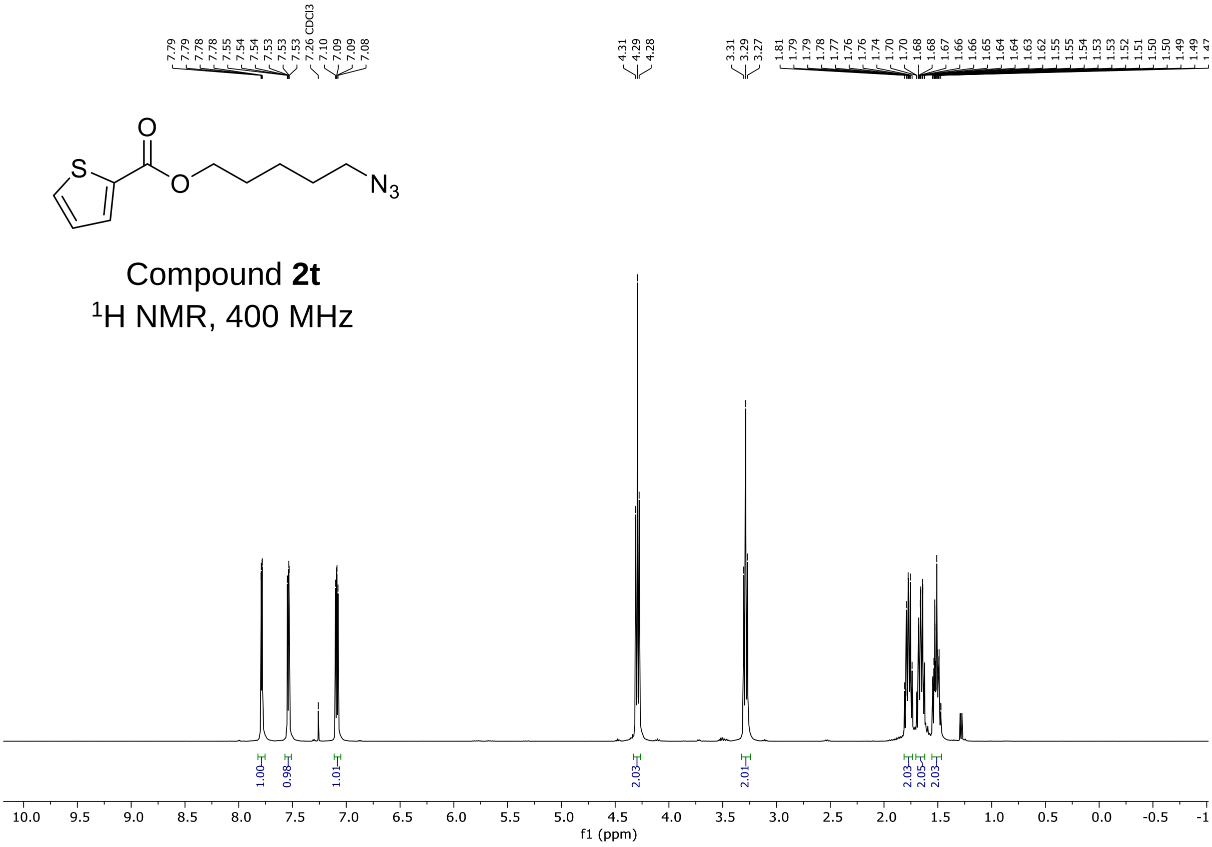
7.79 7.79 7.78 7.78 7.55 7.54 7.54 7.53 7.53 7.53 7.26 CDCl3 7.10 7.09 7.09 7.08
4.31 4.29 4.28
3.31 3.29 3.27
1.81 1.79 1.78 1.77 1.76 1.76 1.74 1.70 1.68 1.68 1.67 1.66 1.66 1.65 1.64 1.64 1.63 1.62 1.55 1.55 1.54 1.53 1.53 1.52 1.51 1.50 1.49 1.49 1.47
S
O
O
N3
Compound 2t
1H NMR, 400 MHz
1.00 0.98 1.01 2.03 2.01 2.03 2.05 2.03
10.0 9.5 9.0 8.5 8.0 7.5 7.0 6.5 6.0 5.5 5.0 4.5 4.0 3.5 3.0 2.5 2.0 1.5 1.0 0.5 0.0 -0.5 -1
f1 (ppm)

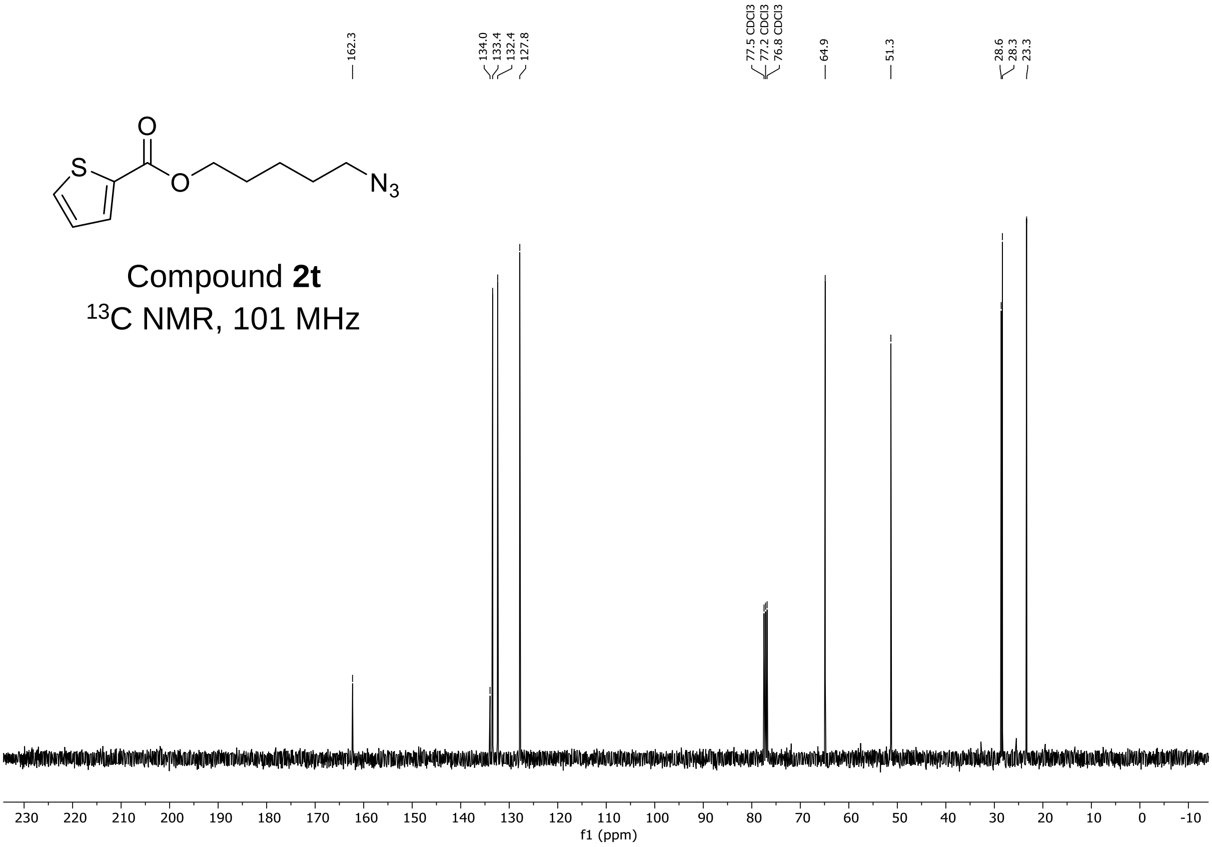
162.3
134.0 133.4 132.4 127.8
77.5 CDCl3 77.2 CDCl3 76.8 CDCl3
64.9
51.3
28.6 28.3 23.3
S
O
O
N3
Compound 2t
13C NMR, 101 MHz
230 220 210 200 190 180 170 160 150 140 130 120 110 100 90 80 70 60 50 40 30 20 10 0 -10
f1 (ppm)

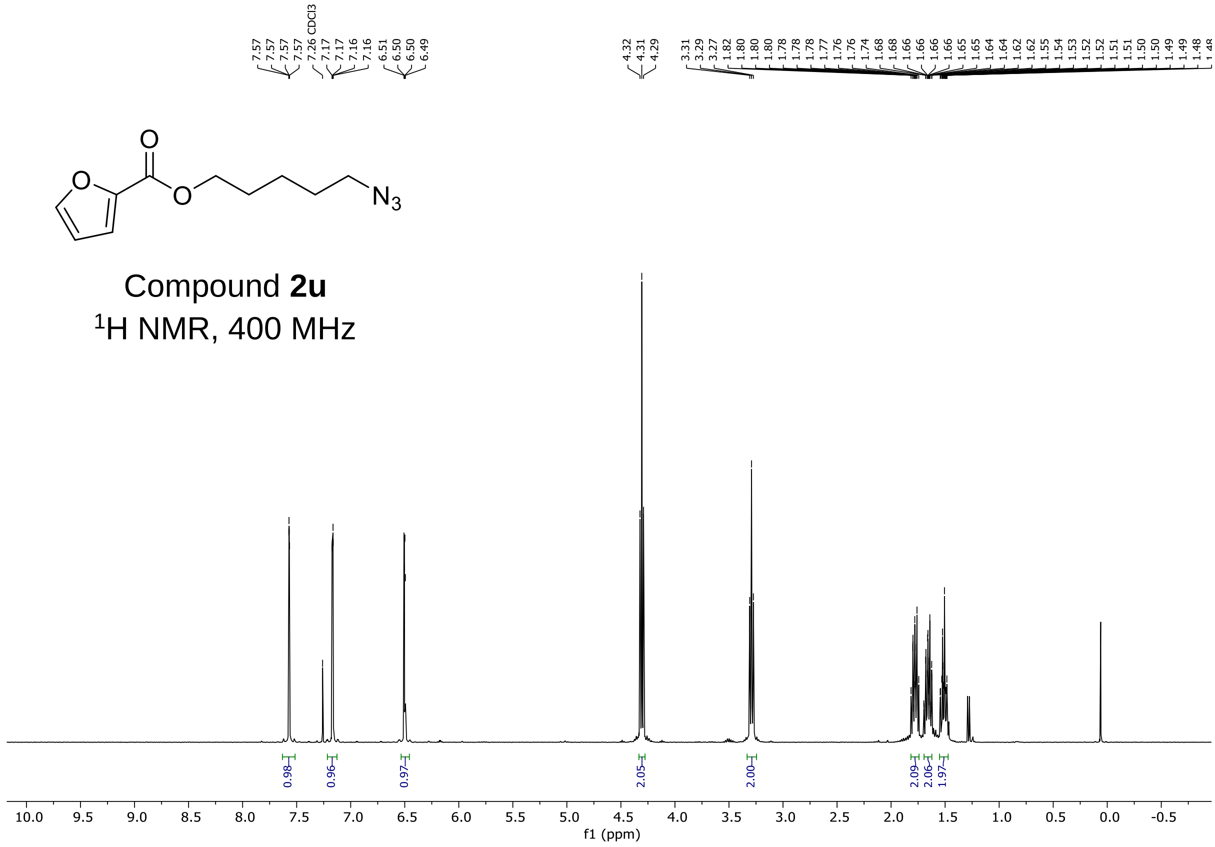
Compound **2u**
^{1}H NMR, 400 MHz
f1 (ppm)

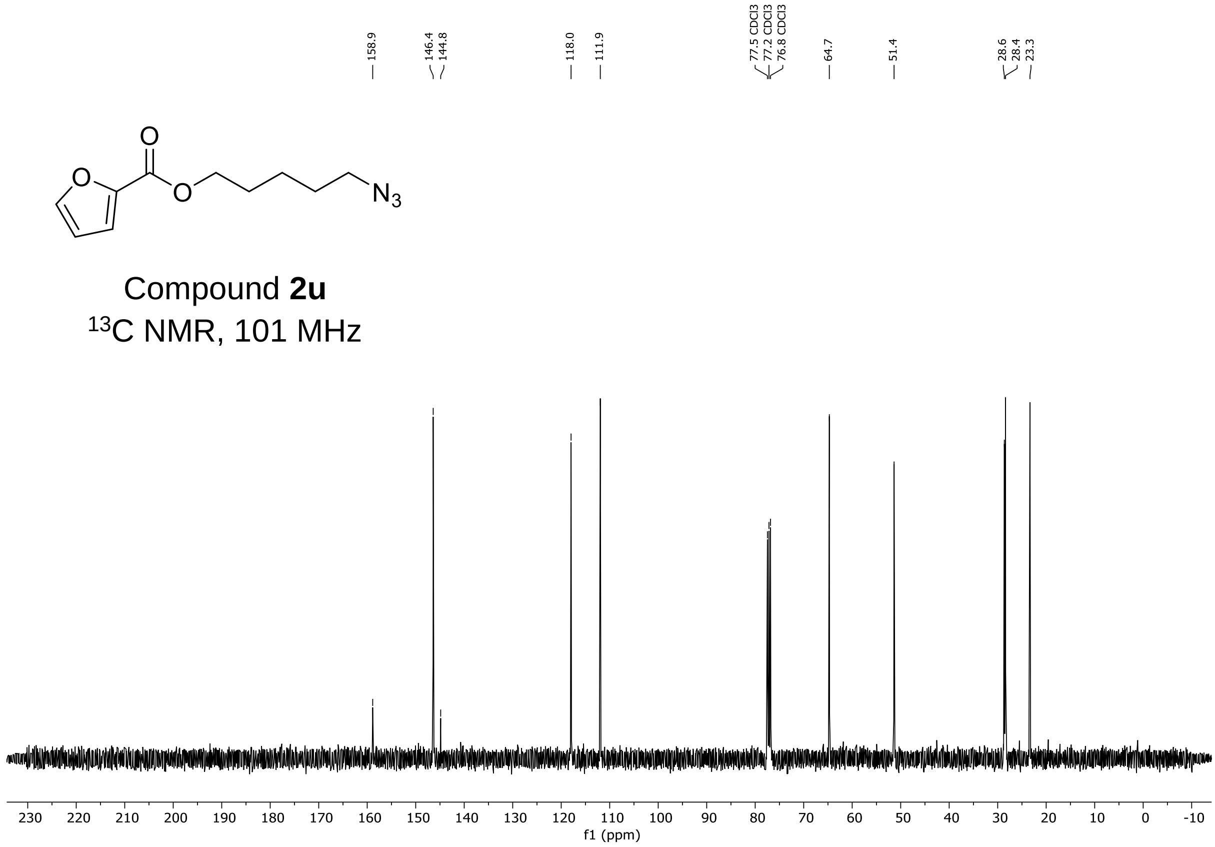
Compound **2u**
^{13}C NMR, 101 MHz
f1 (ppm)

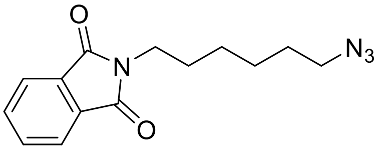

Compound **2v**
^{1}H NMR, 400 MHz

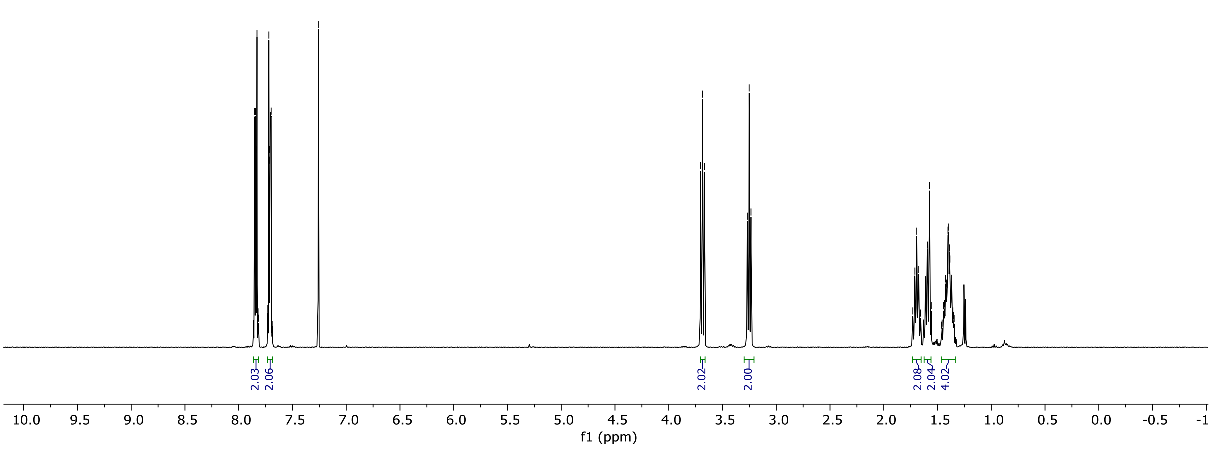

Compound **2v**
^{13}C NMR, 101 MHz

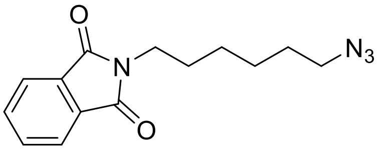

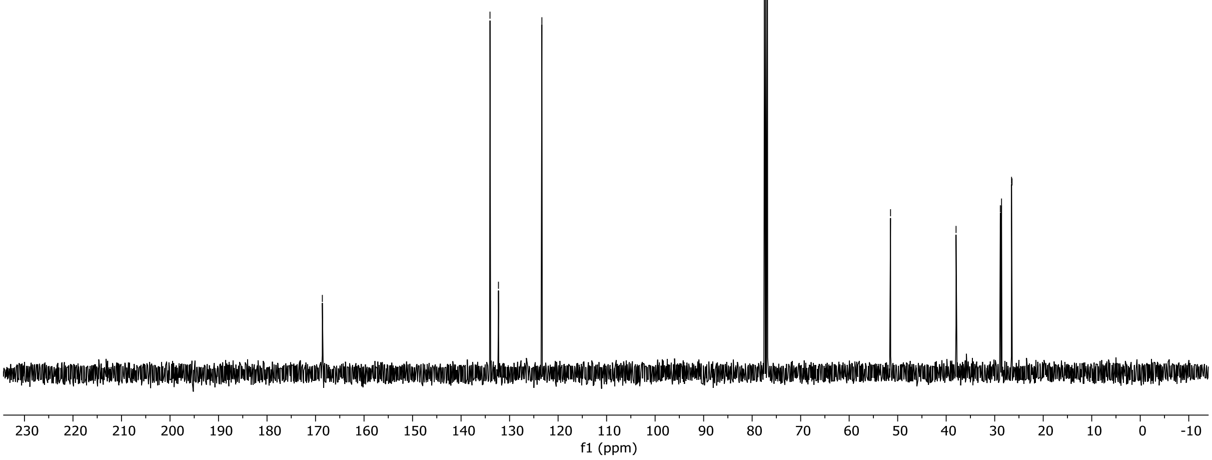

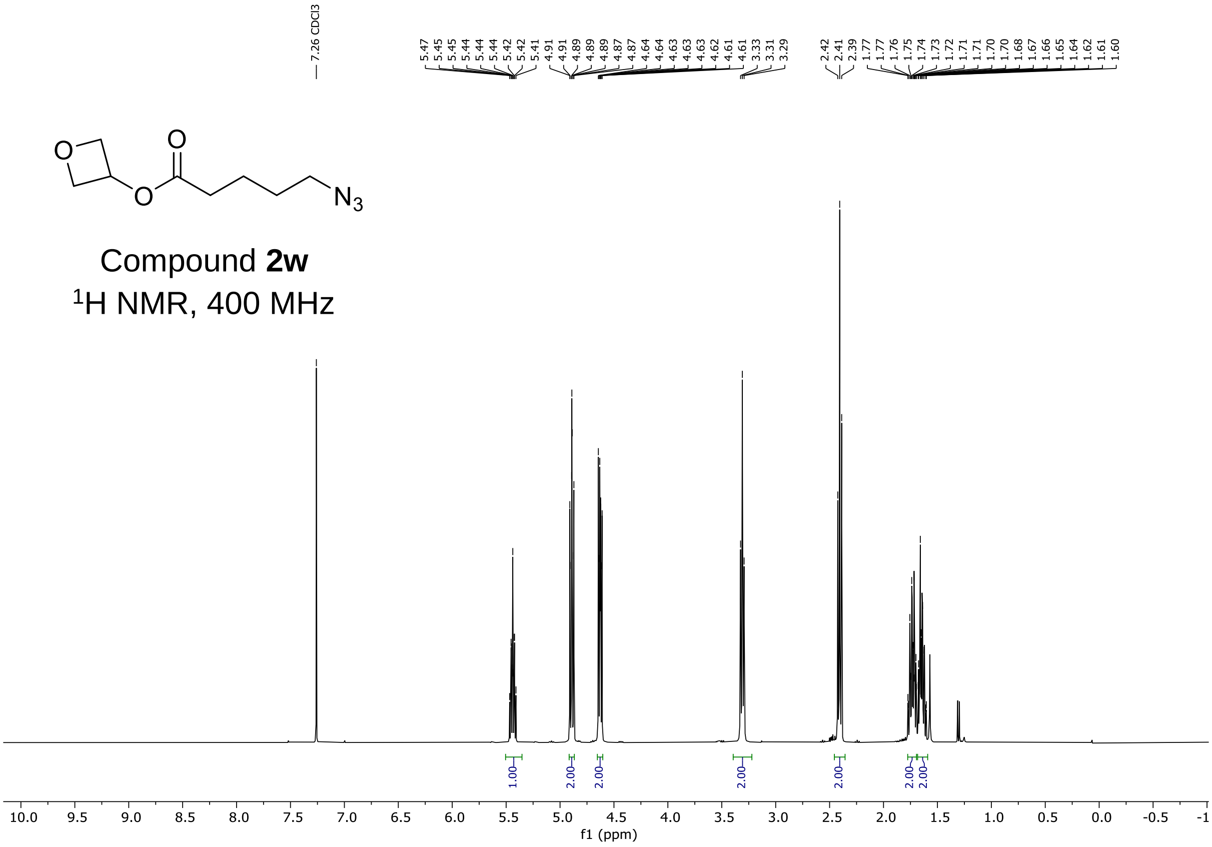
Compound **2w**
^{1}H NMR, 400 MHz
7.26 CDCl3
f1 (ppm)

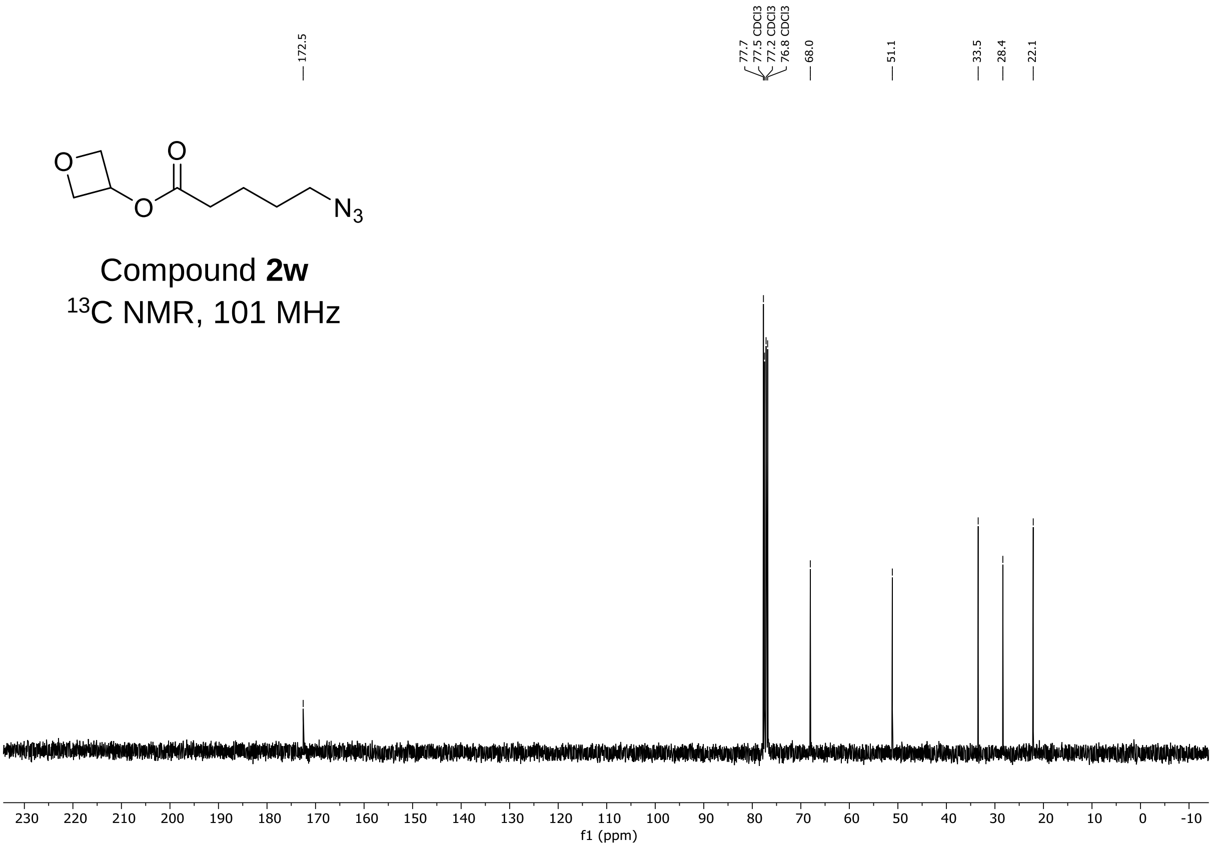
Compound **2w**
^{13}C NMR, 101 MHz
172.5
77.7 CDCl3
77.5 CDCl3
77.2 CDCl3
76.8 CDCl3
68.0
51.1
33.5
28.4
22.1
f1 (ppm)

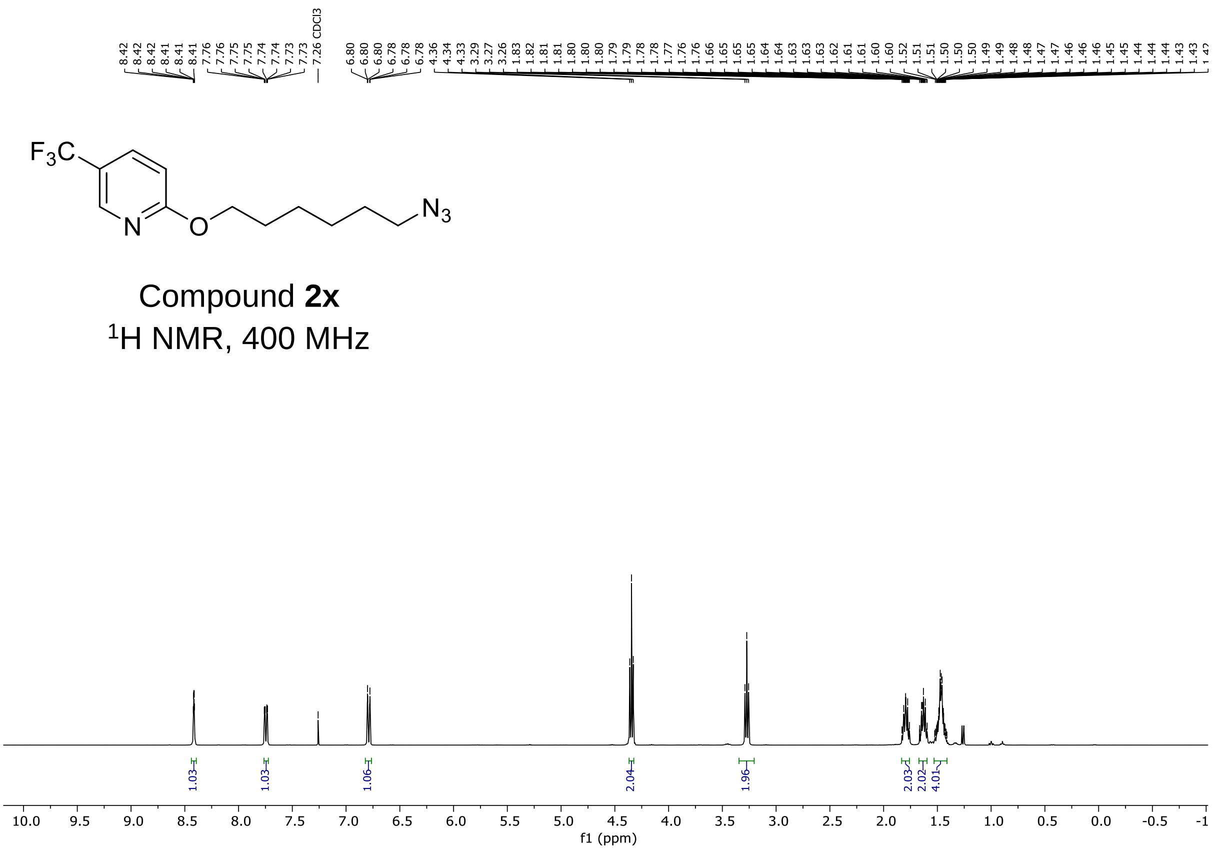

Compound **2x**
^{1}H NMR, 400 MHz

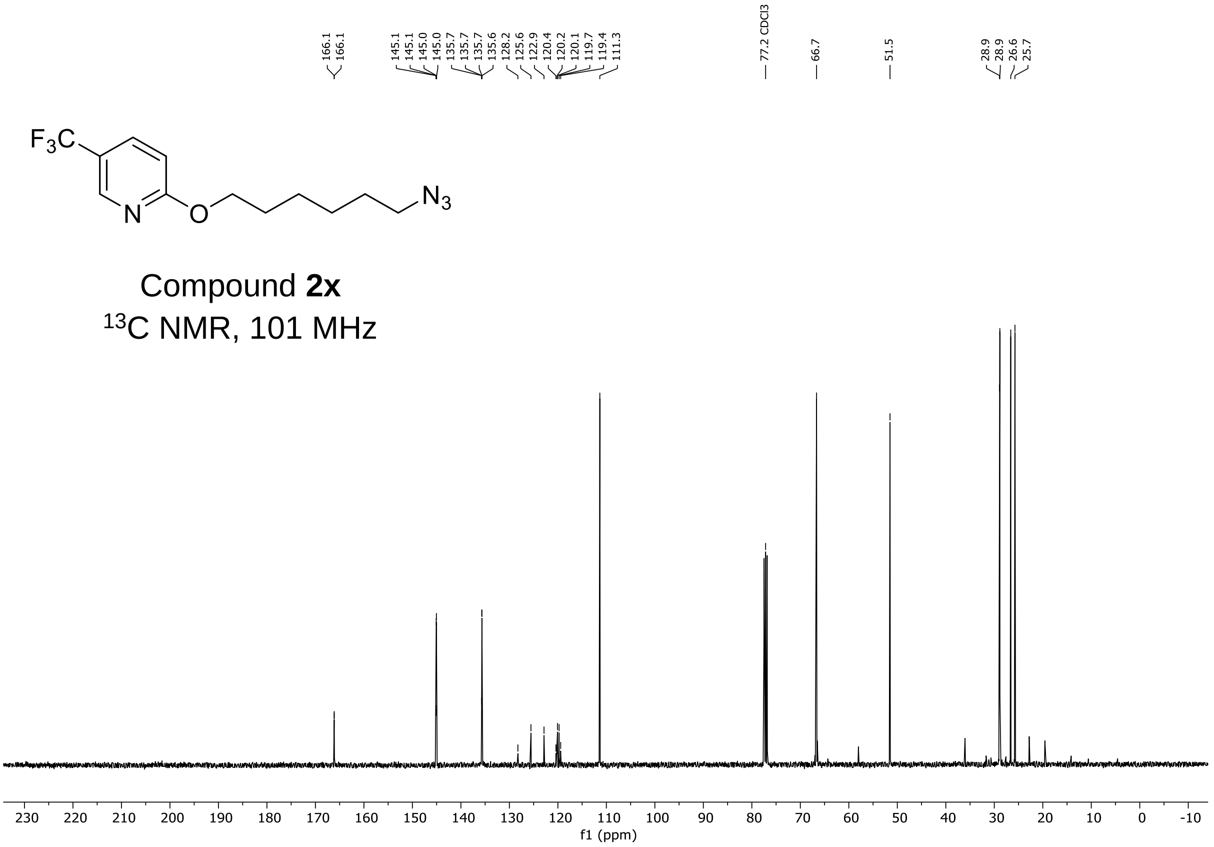

Compound **2x**
^{13}C NMR, 101 MHz

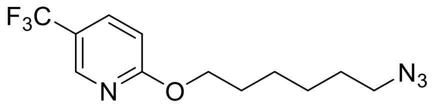

Compound **2x**
^{19}F NMR, 376 MHz

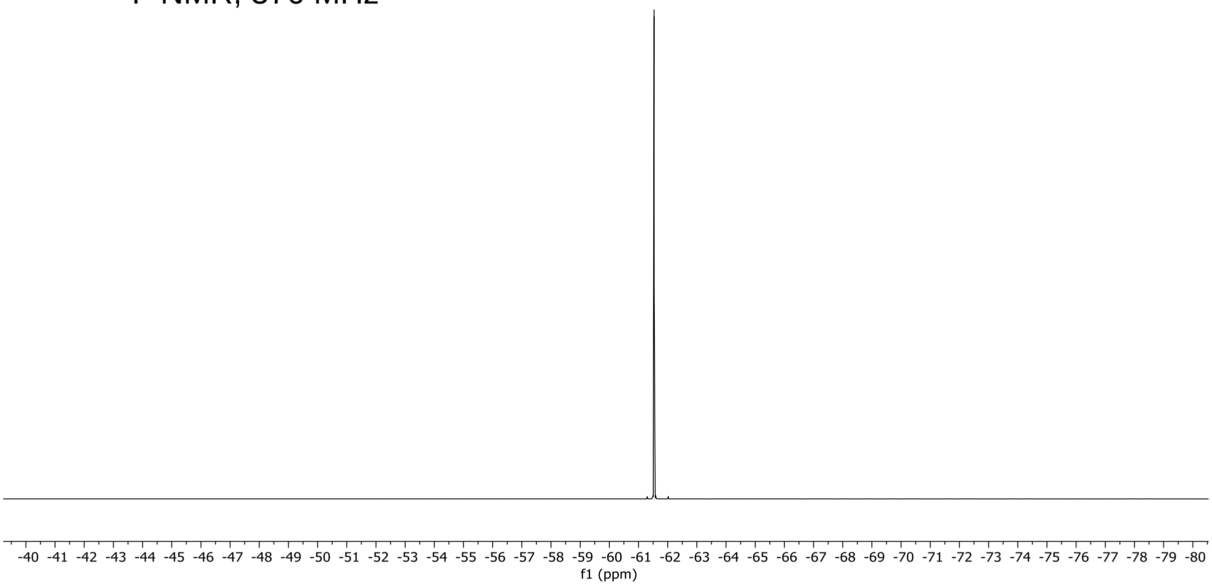

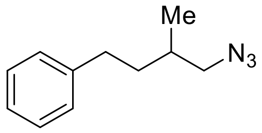

Compound **2y**
^{1}H NMR, 400 MHz

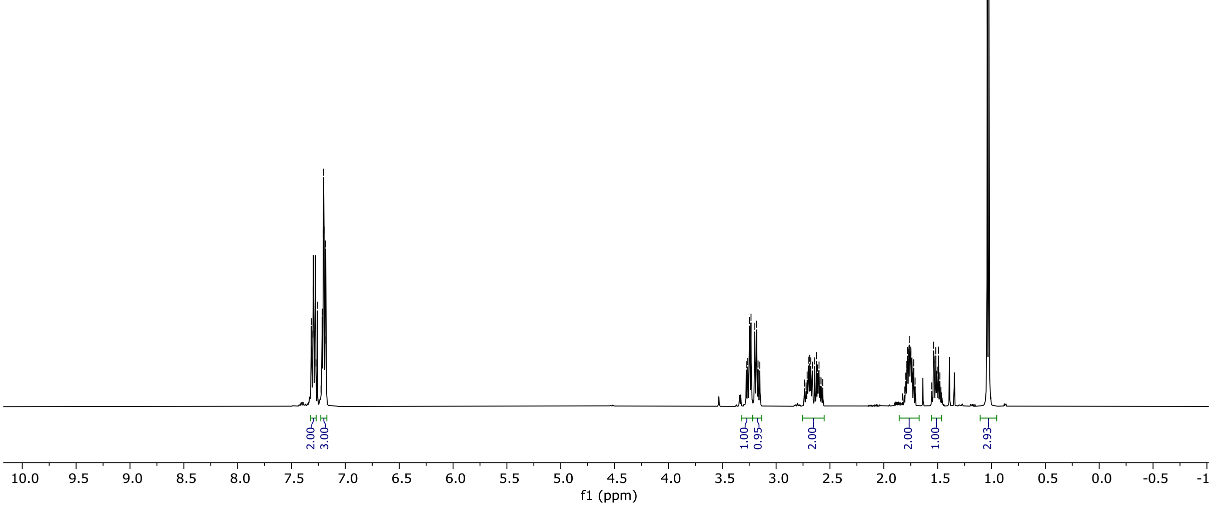

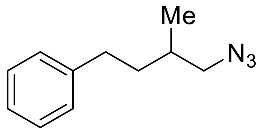

Compound **2y**
^{13}C NMR, 101 MHz

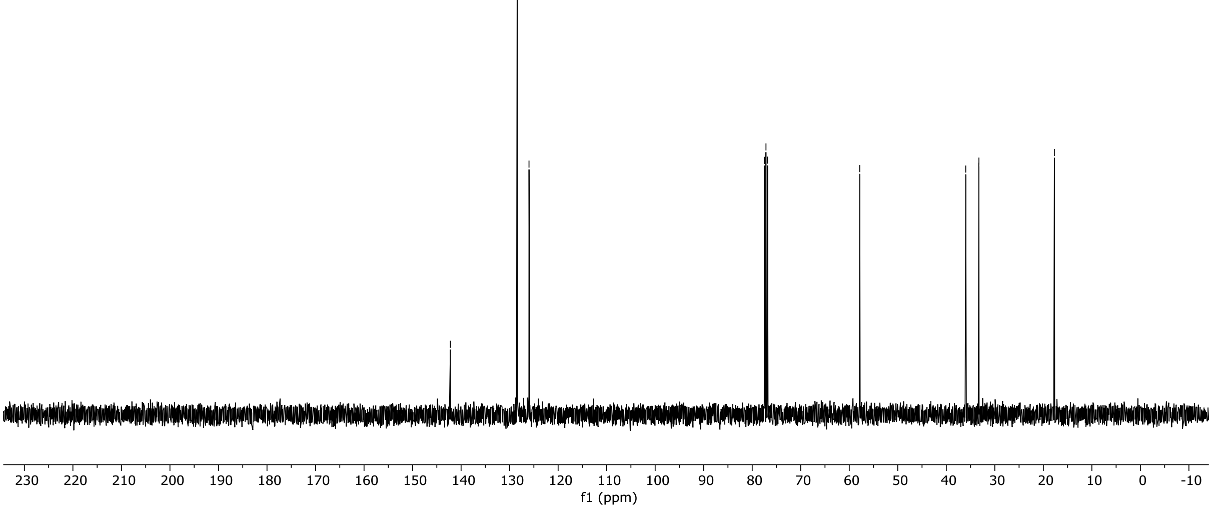

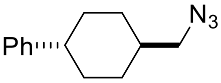

Compound **2z**
^{1}H NMR, 400 MHz

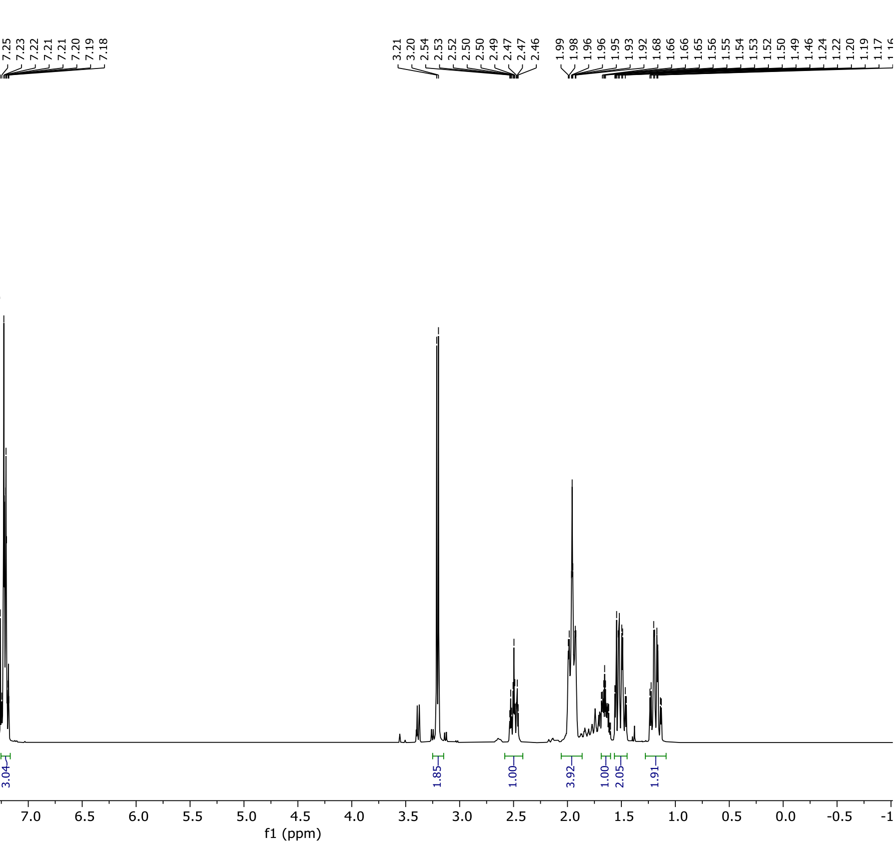

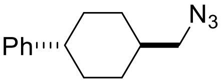

Compound **2z**
^{13}C NMR, 101 MHz

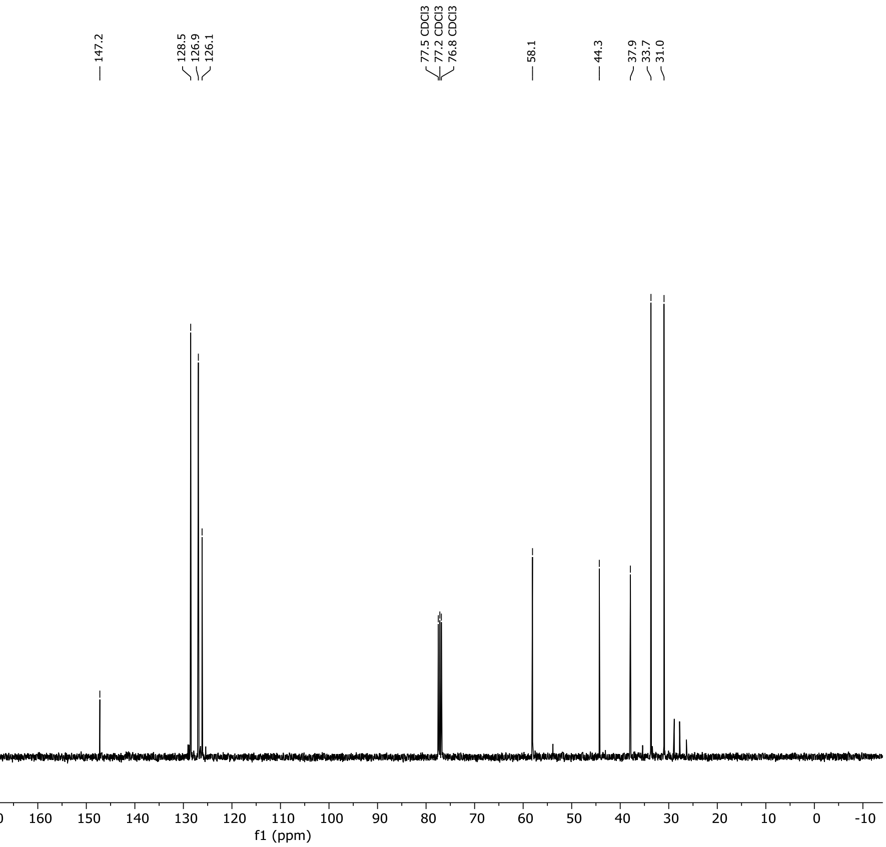

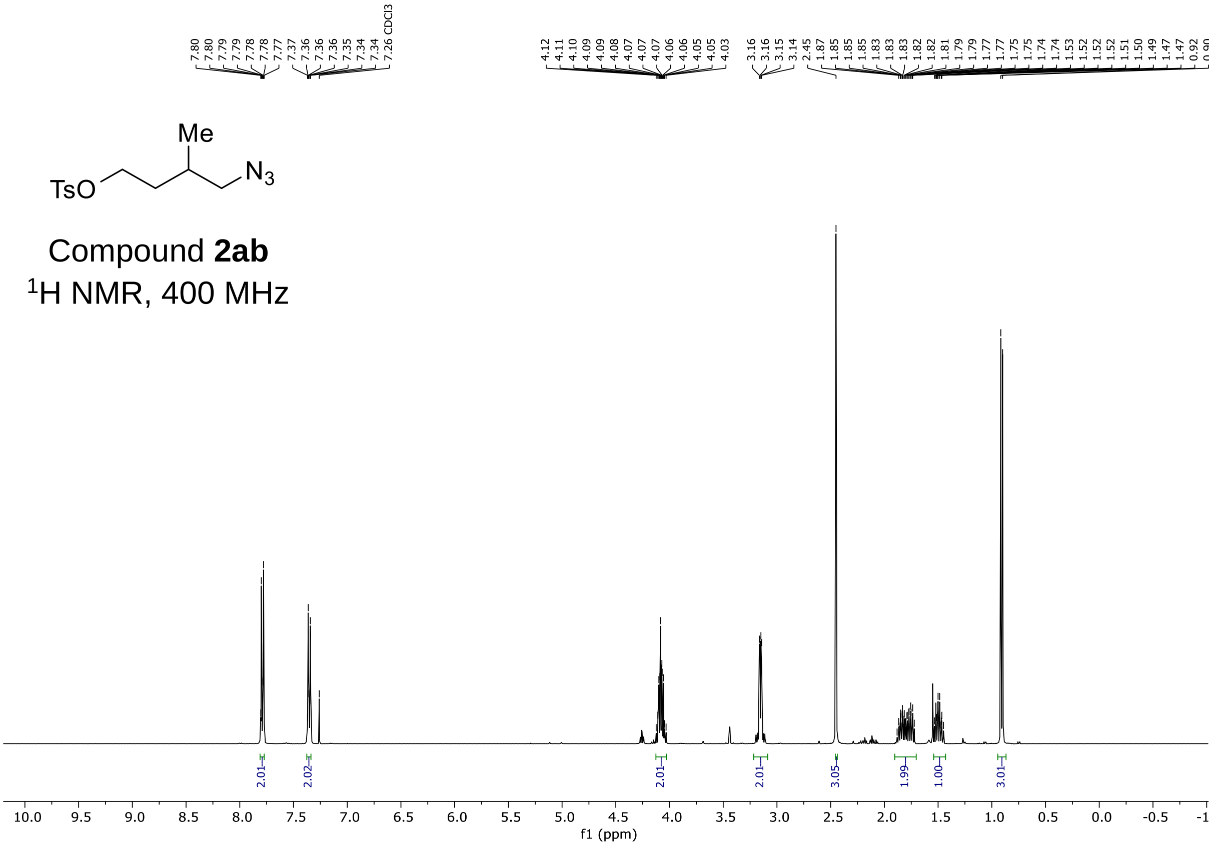
Me
TsO
N3
Compound 2ab
1H NMR, 400 MHz
f1 (ppm)

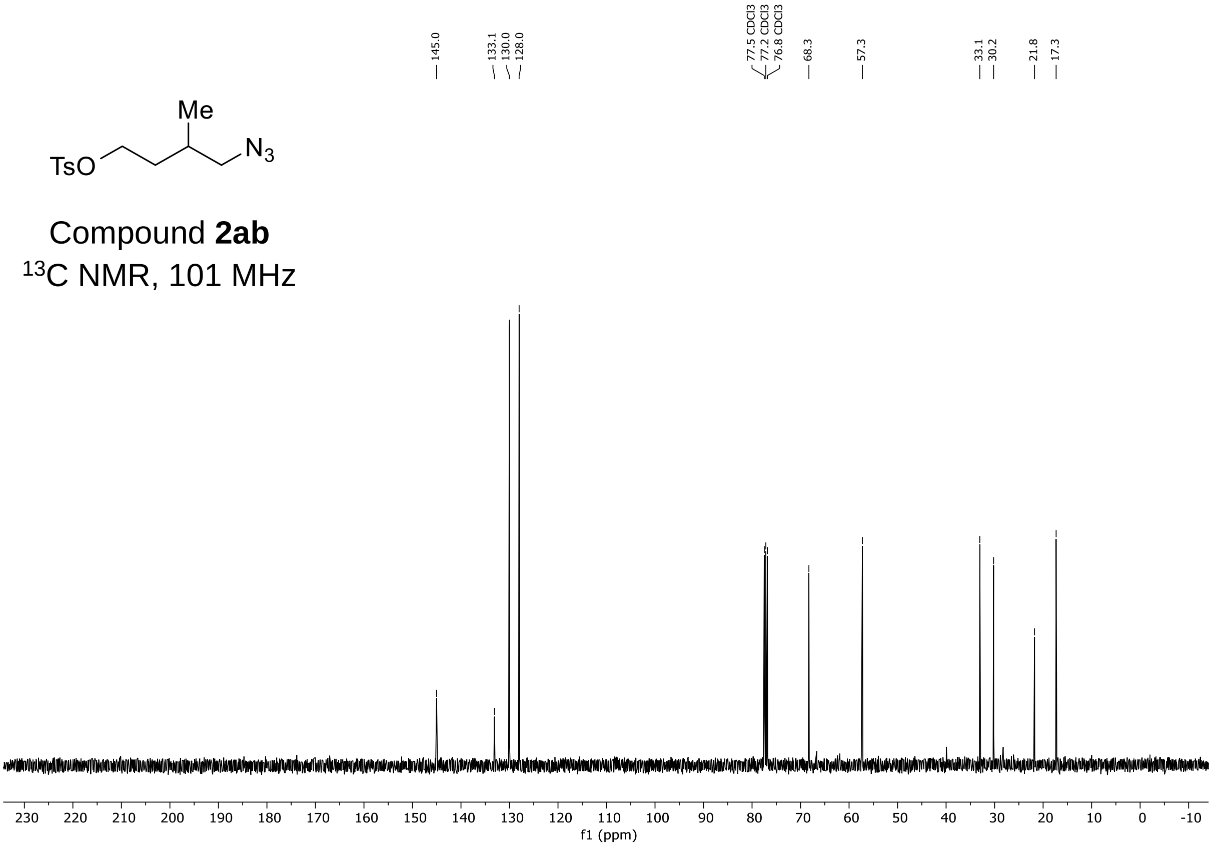
Me
TsO
N3
Compound 2ab
13C NMR, 101 MHz
f1 (ppm)

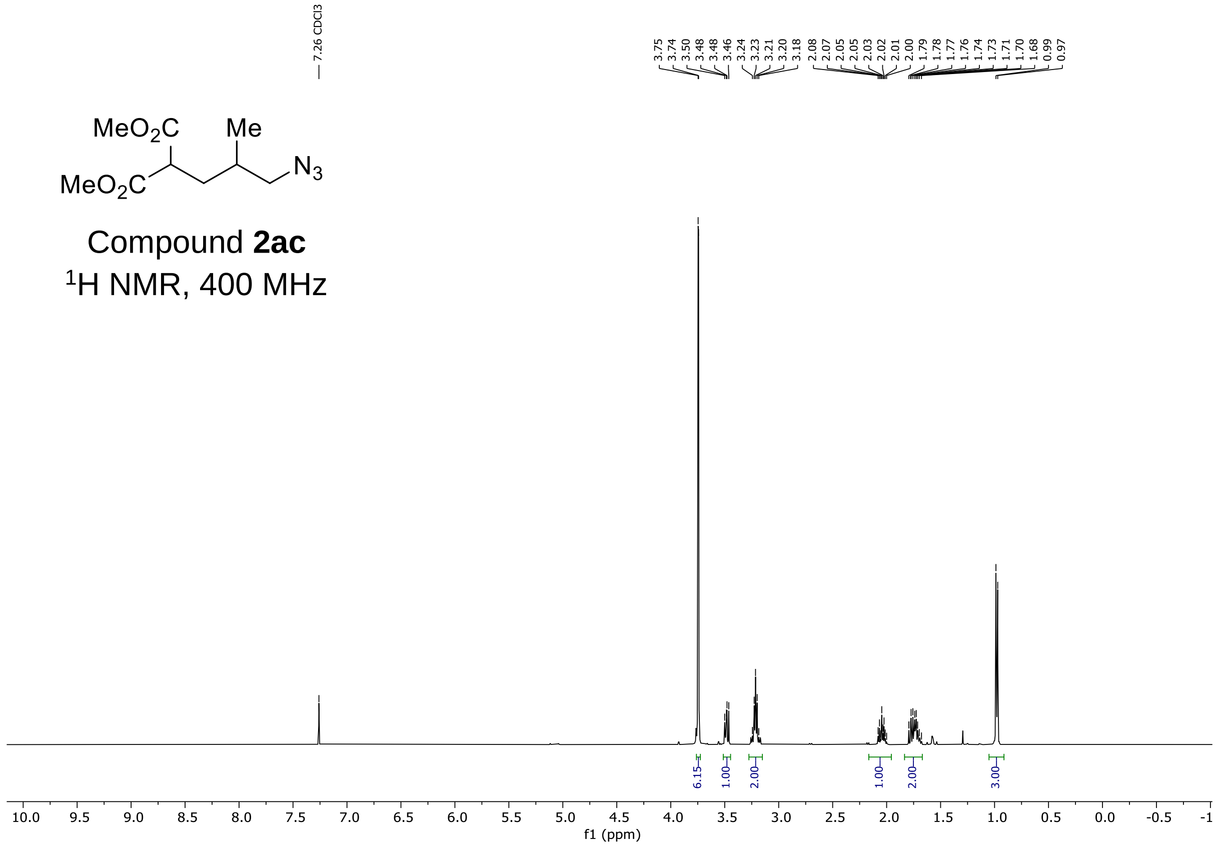

Compound **2ac**
^{1}H NMR, 400 MHz

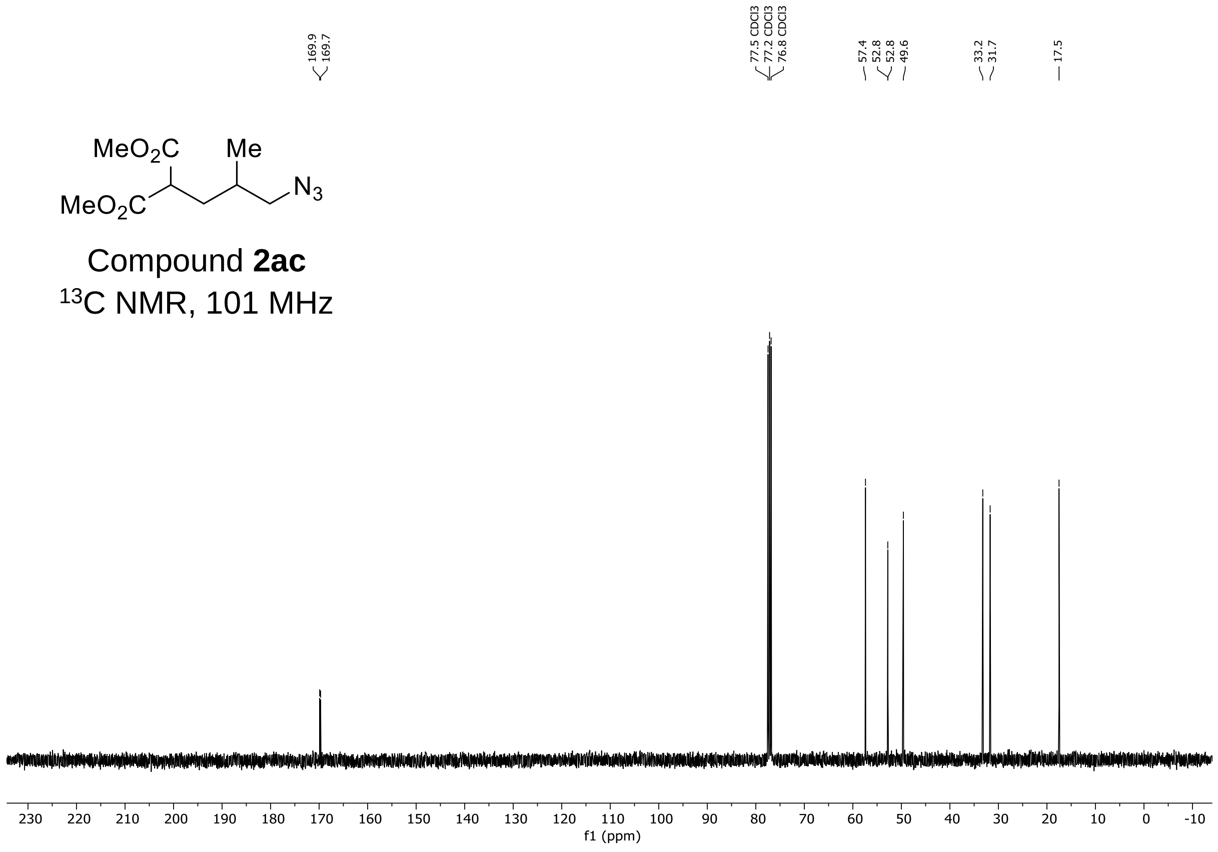

Compound **2ac**
^{13}C NMR, 101 MHz

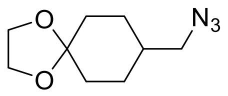

Compound **2ad**

¹H NMR, 400 MHz

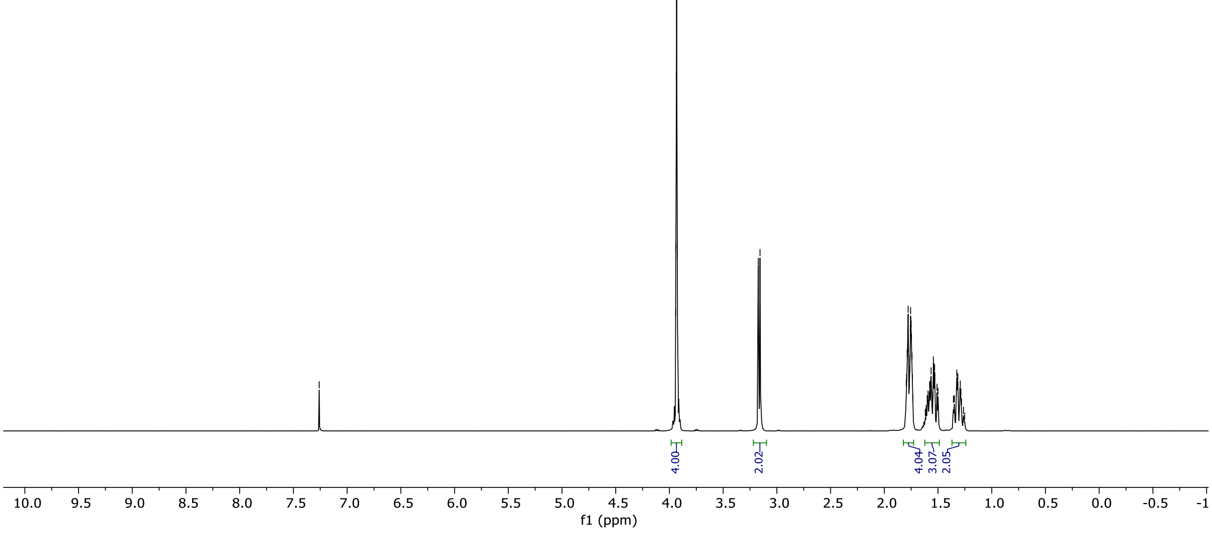

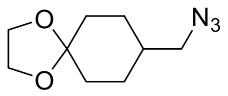

Compound **2ad**

¹³C NMR, 101 MHz

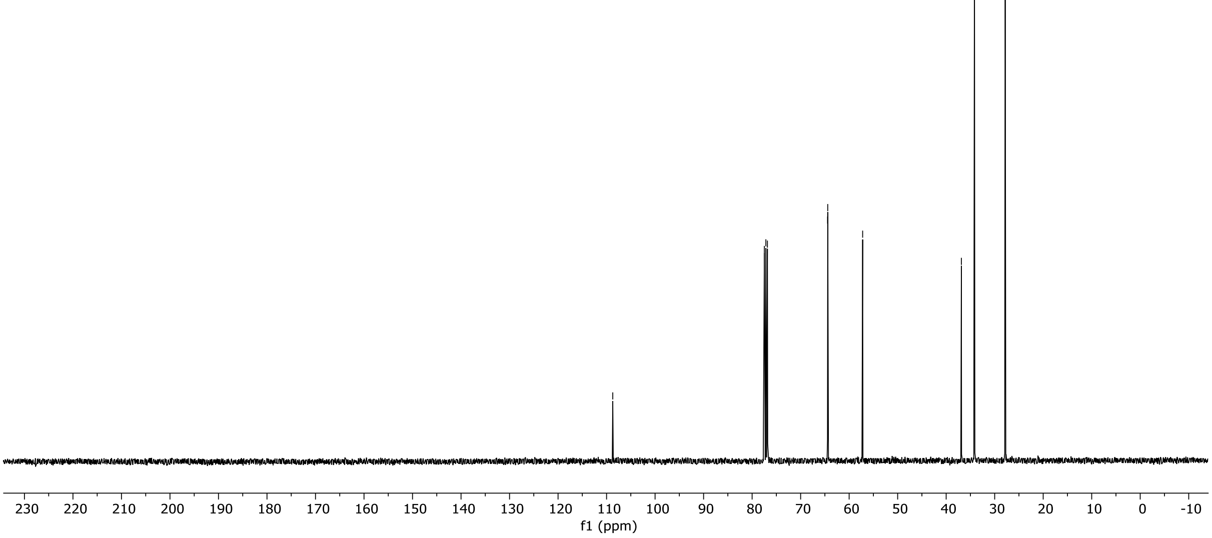

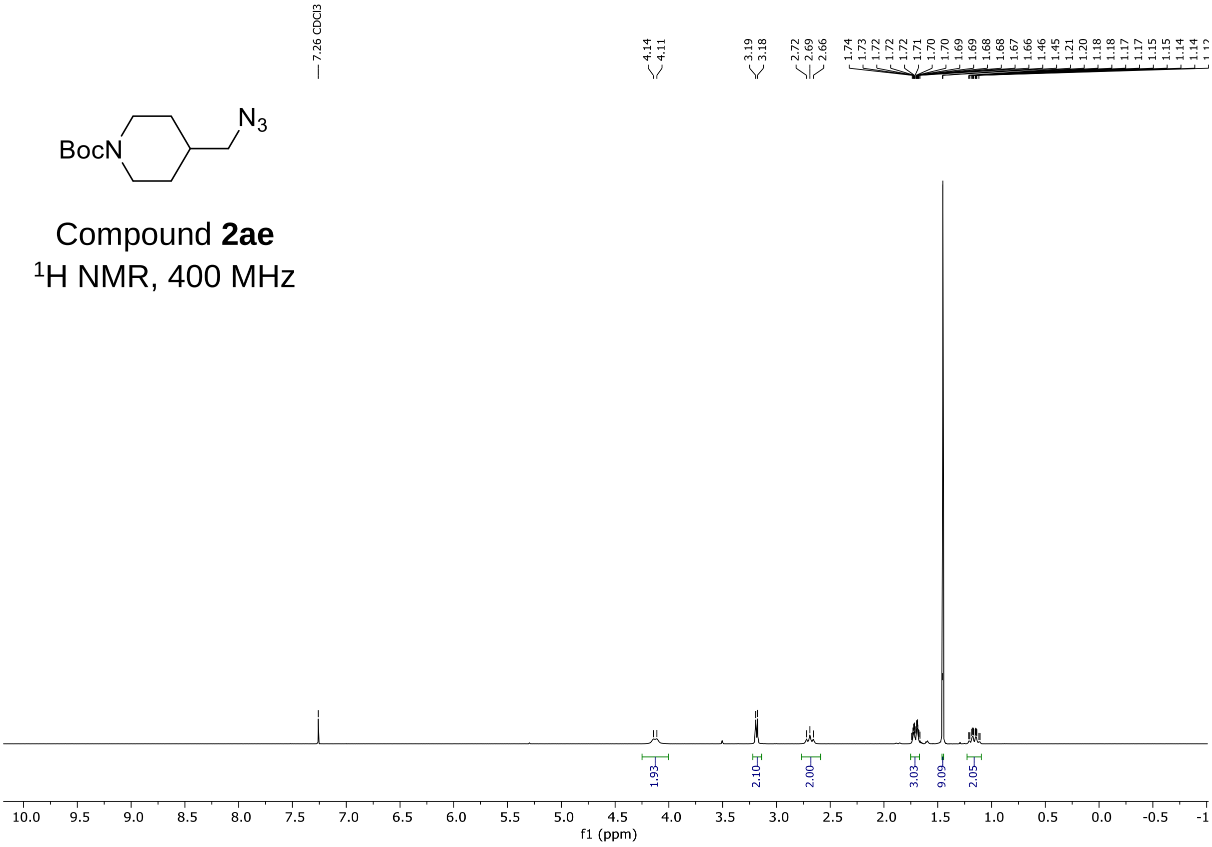

Compound **2ae**
^{1}H NMR, 400 MHz

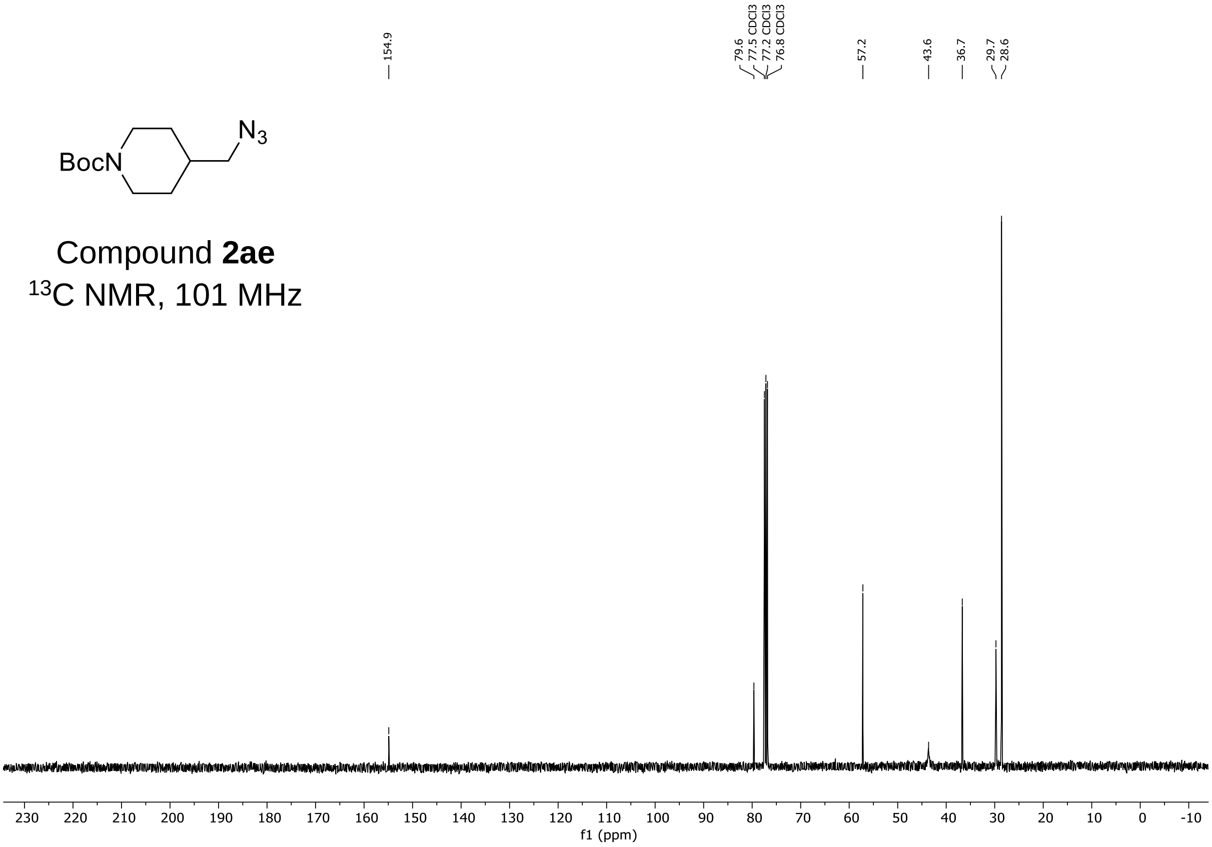

Compound **2ae**
^{13}C NMR, 101 MHz

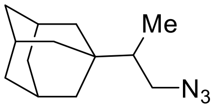

Compound **2af**

^{1}H NMR, 400 MHz

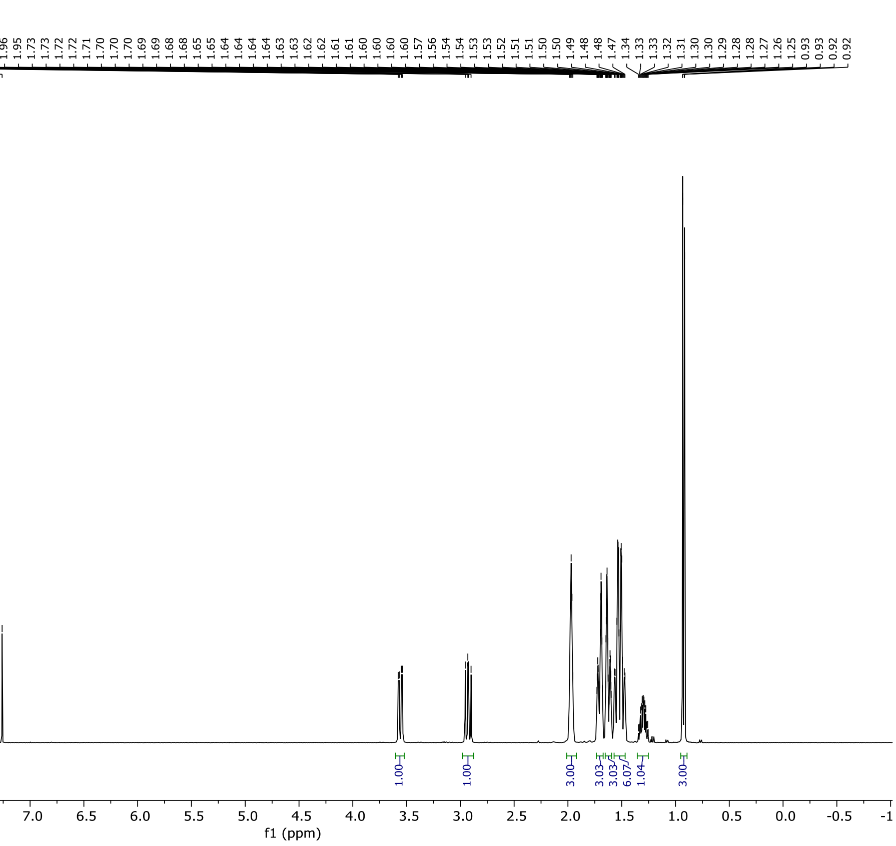

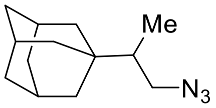

Compound **2af**

^{13}C NMR, 101 MHz

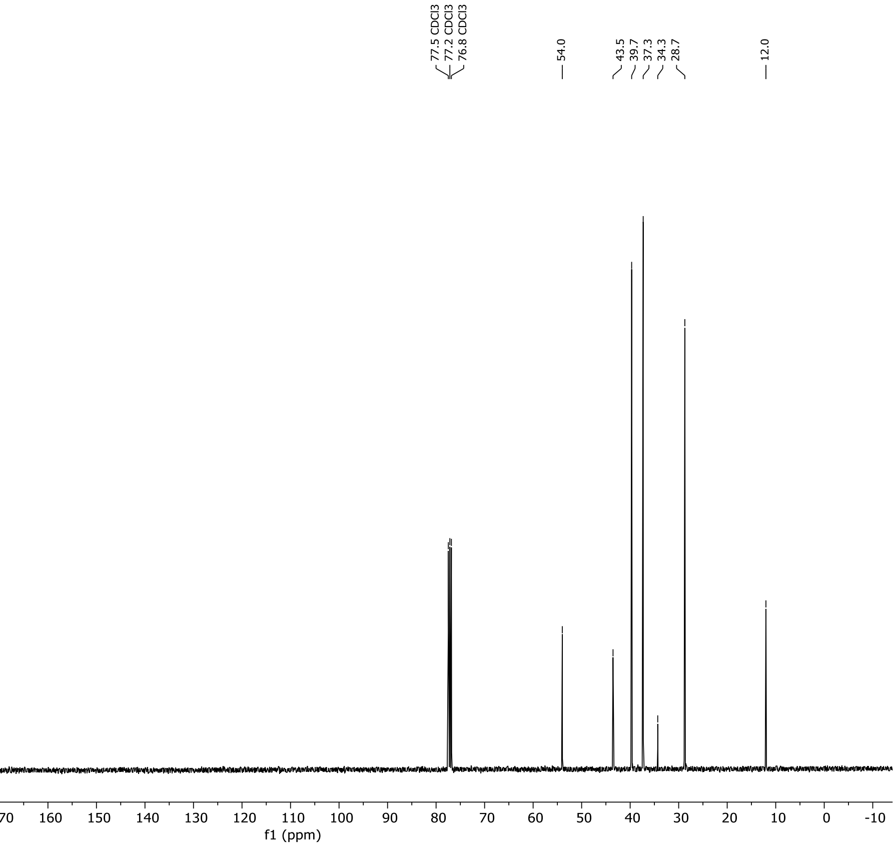

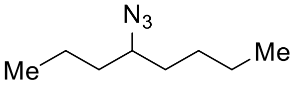

Compound **2ag**
^{1}H NMR, 400 MHz

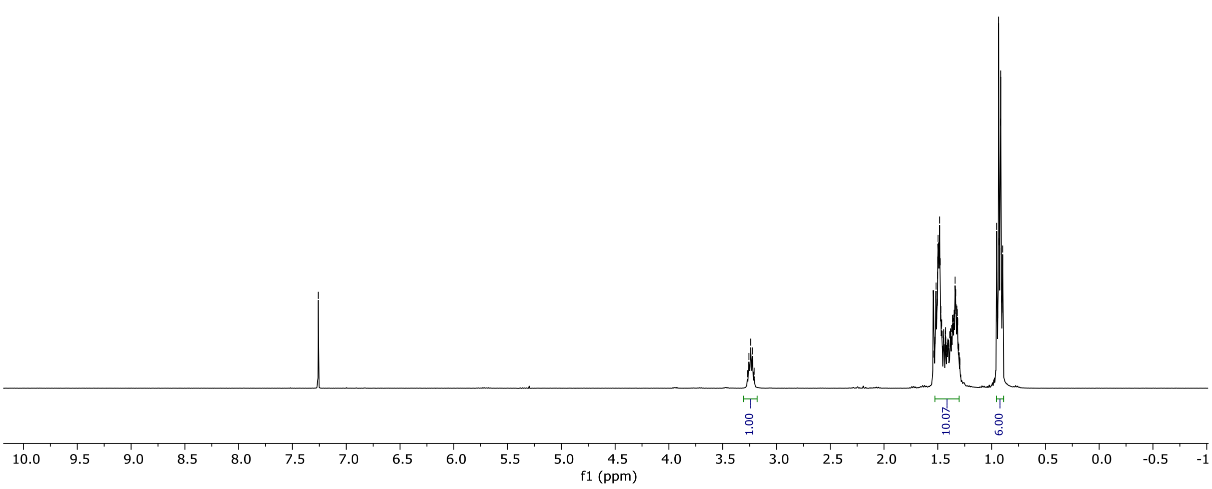

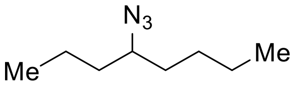

Compound **2ag**
^{13}C NMR, 101 MHz

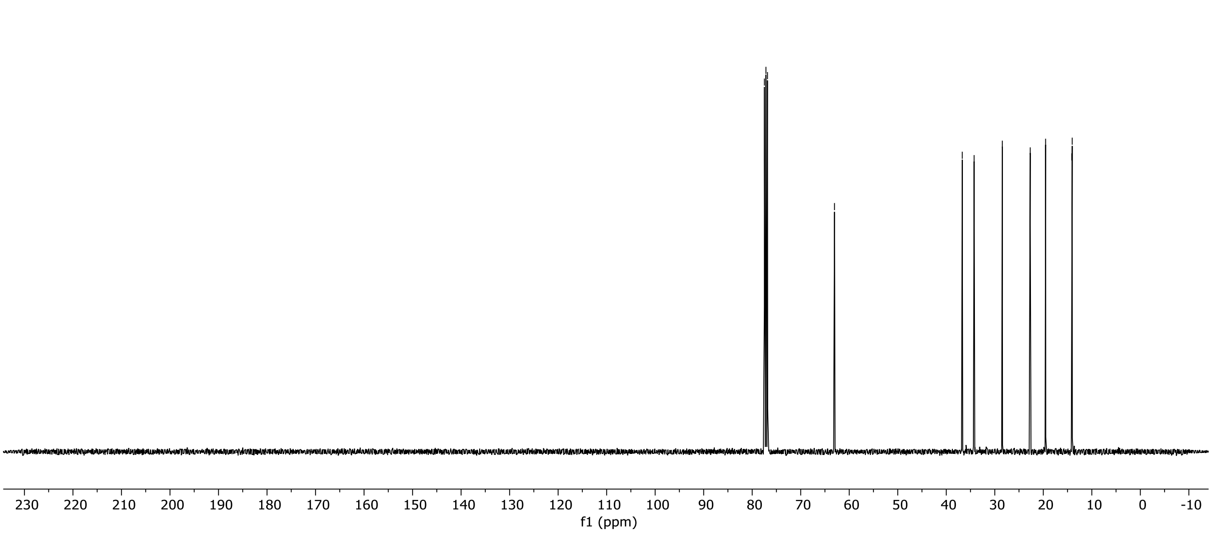

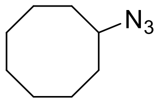

Compound **2ai**
^{1}H NMR, 400 MHz

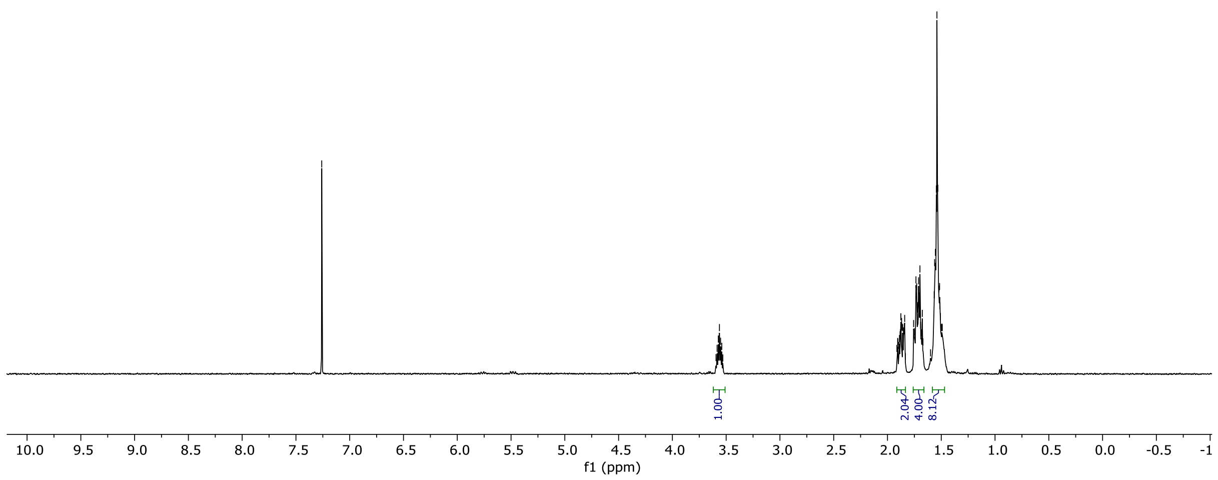

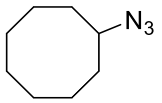

Compound **2ai**
^{13}C NMR, 101 MHz

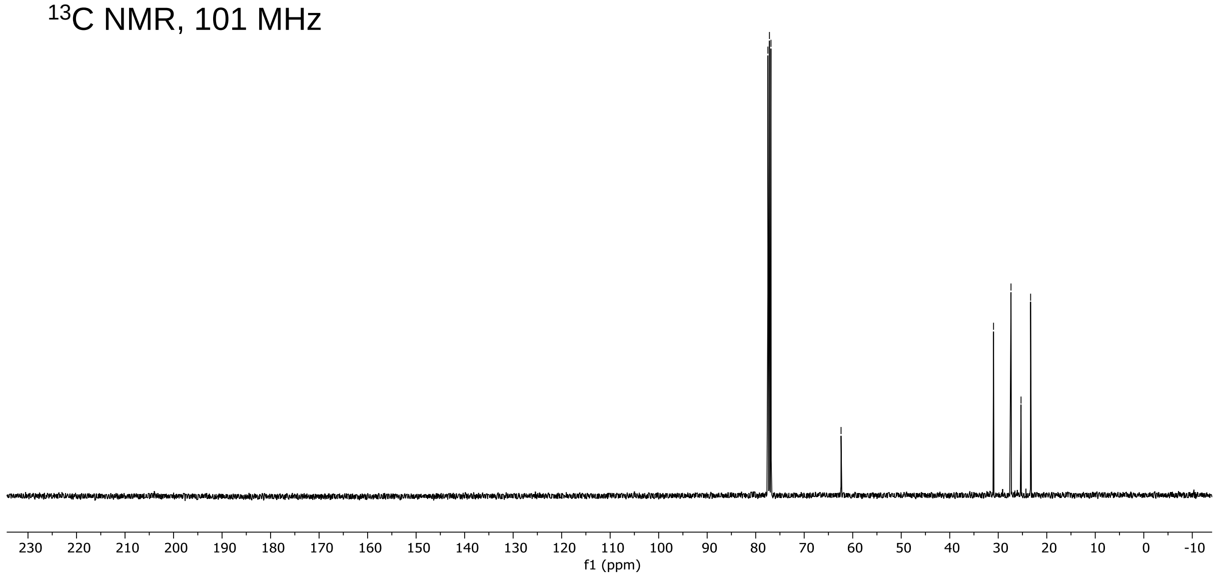

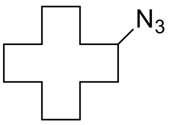

Compound **2aj**
^{1}H NMR, 400 MHz

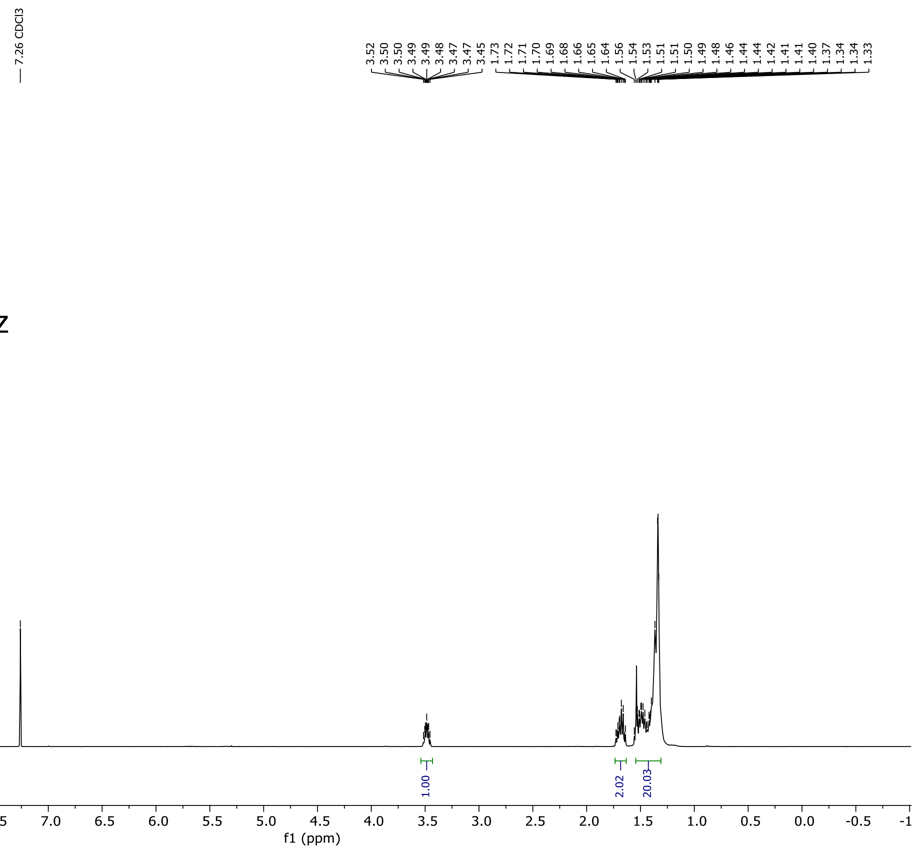

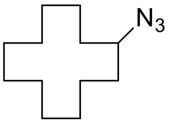

Compound **2aj**
^{13}C NMR, 101 MHz

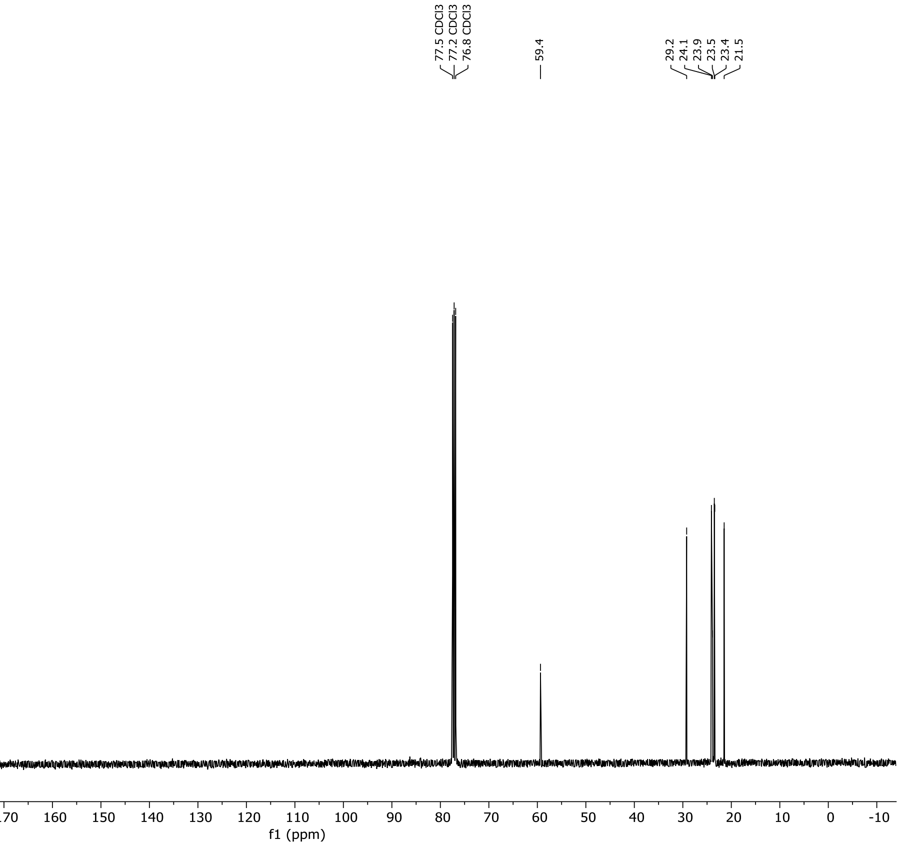

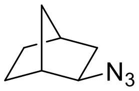

Compound **2ak**
^1H NMR, 400 MHz

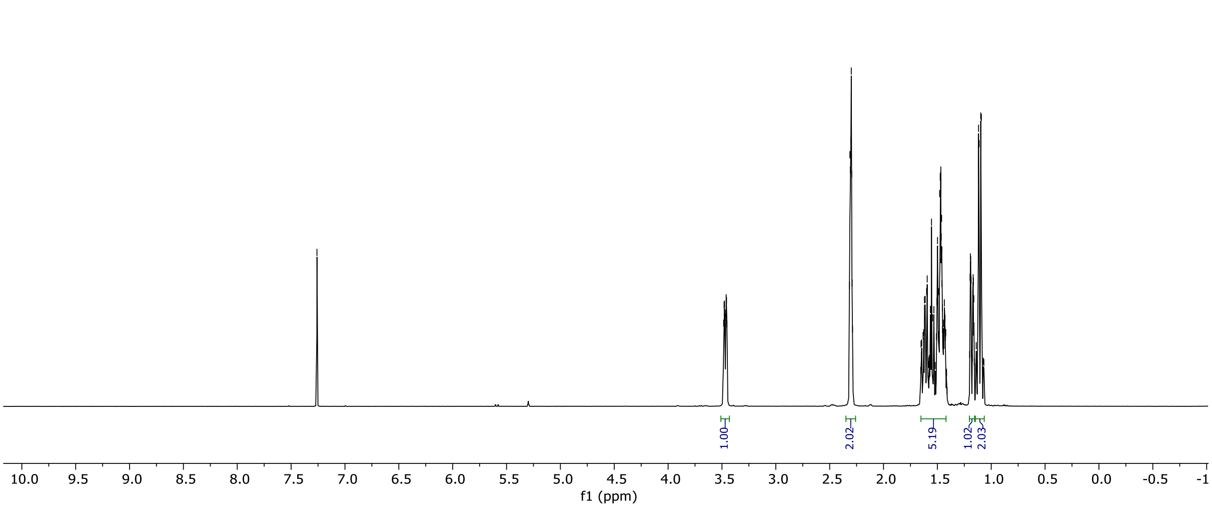

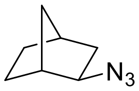

Compound **2ak**
^{13}C NMR, 101 MHz

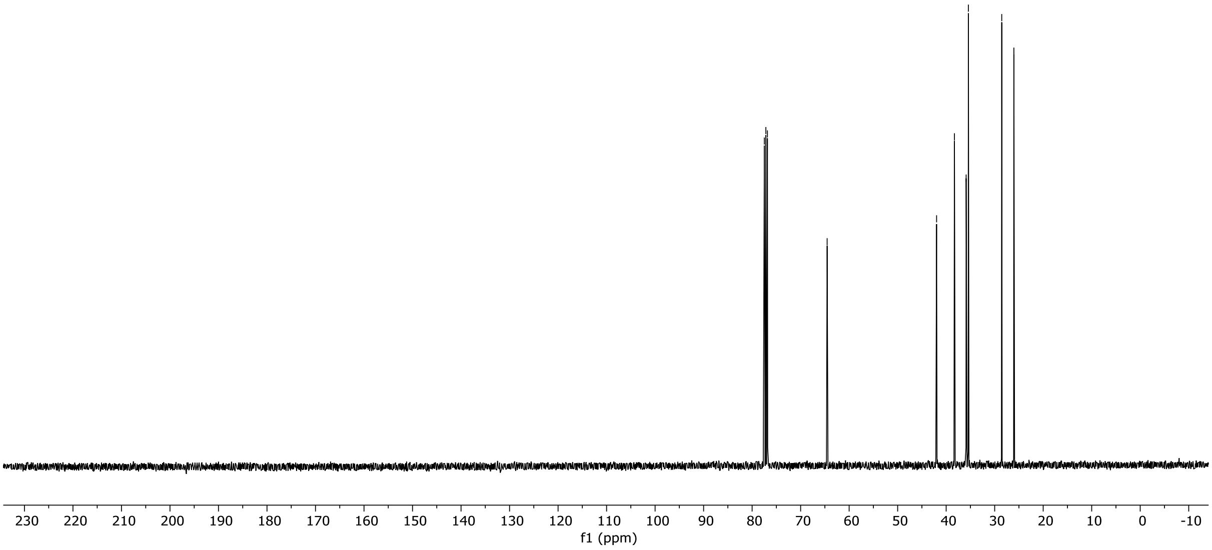

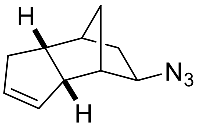

Compound **2al**
^1H NMR, 400 MHz

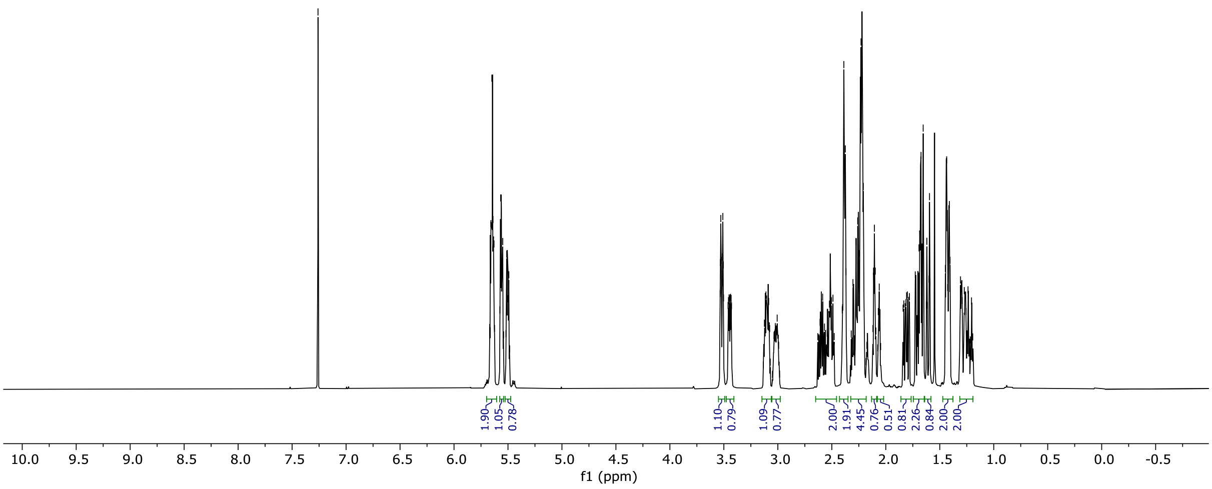

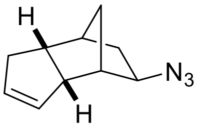

Compound **2al**
^{13}C NMR, 101 MHz

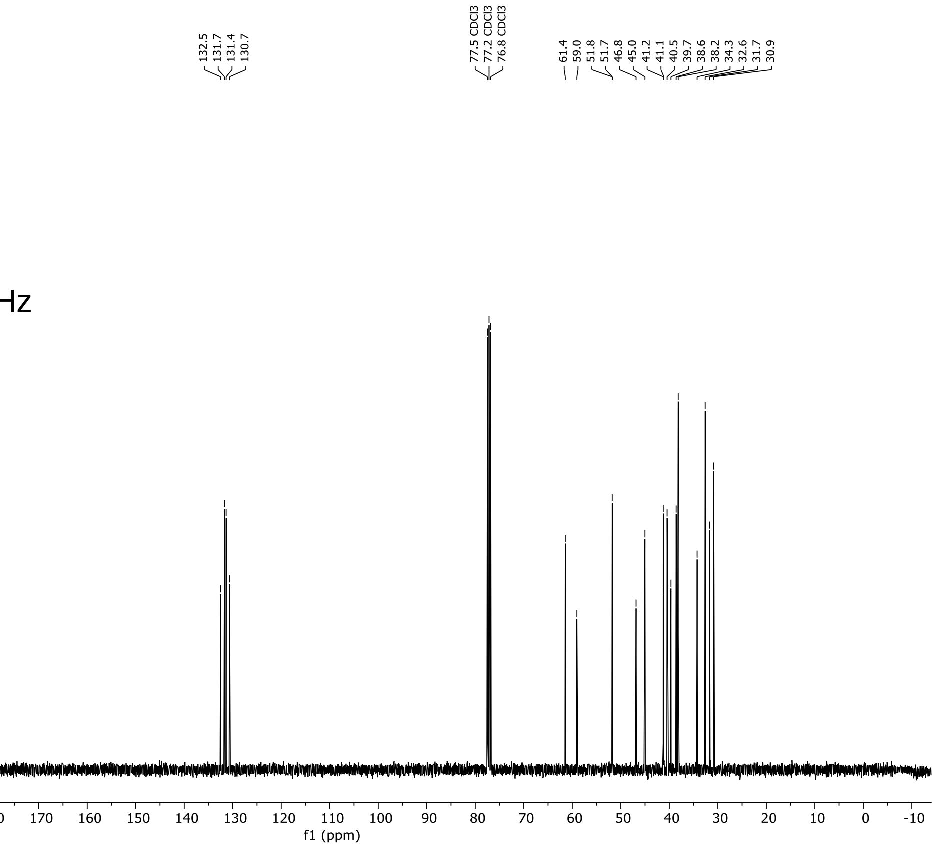

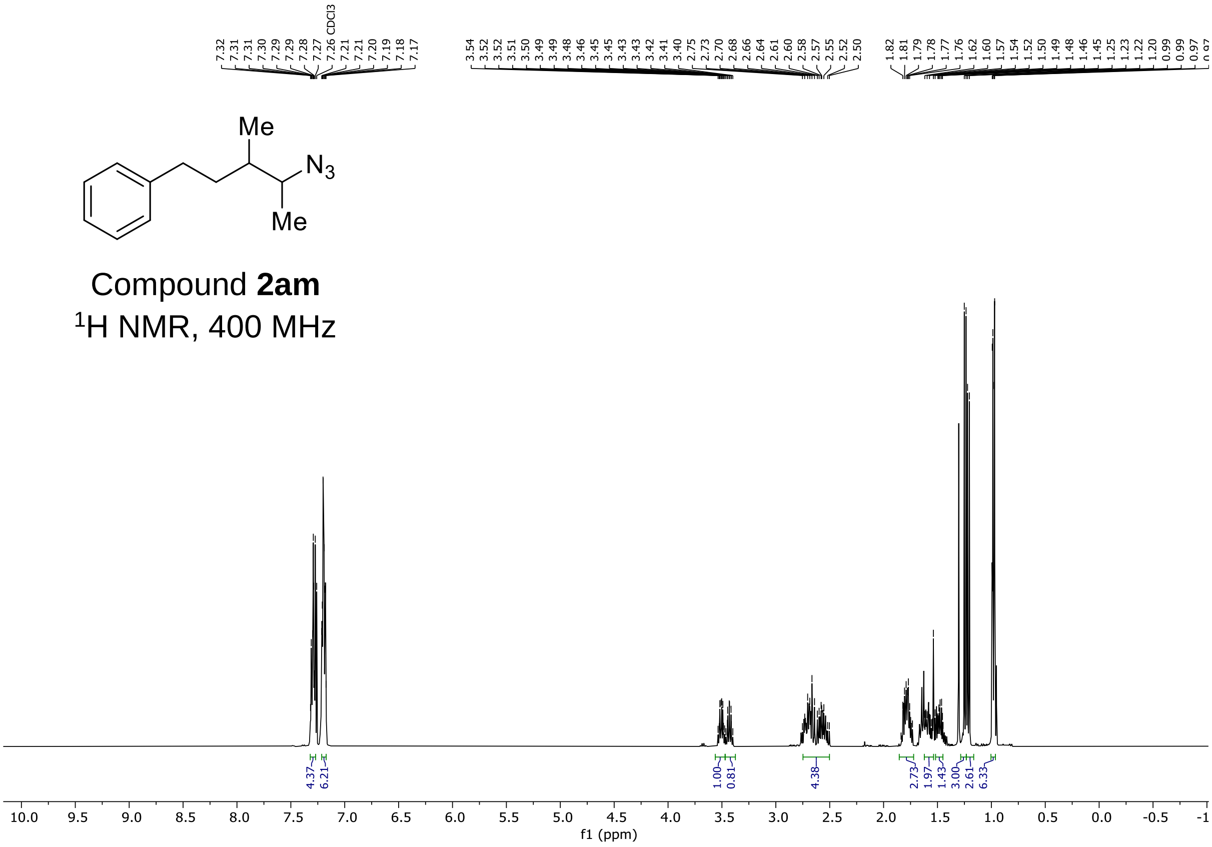

Compound **2am**
^1H NMR, 400 MHz

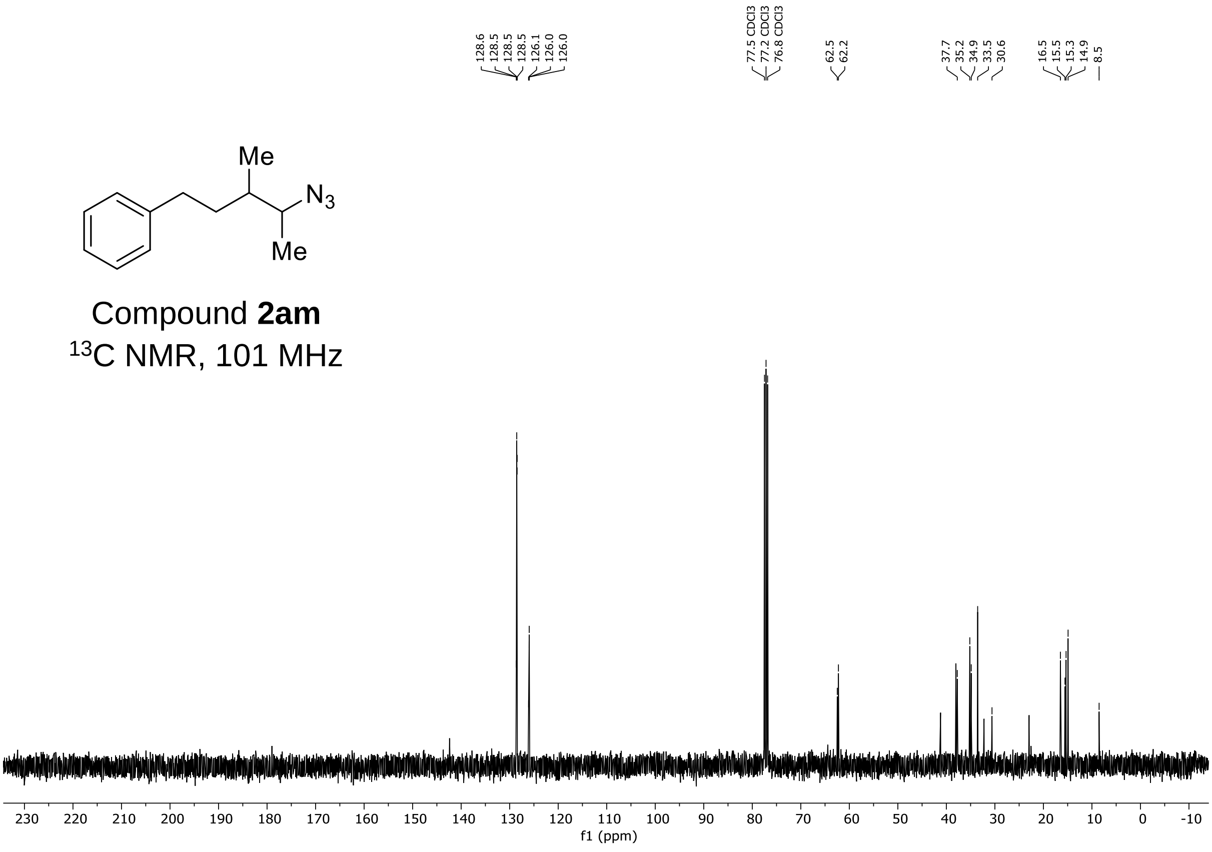

Compound **2am**
^{13}C NMR, 101 MHz

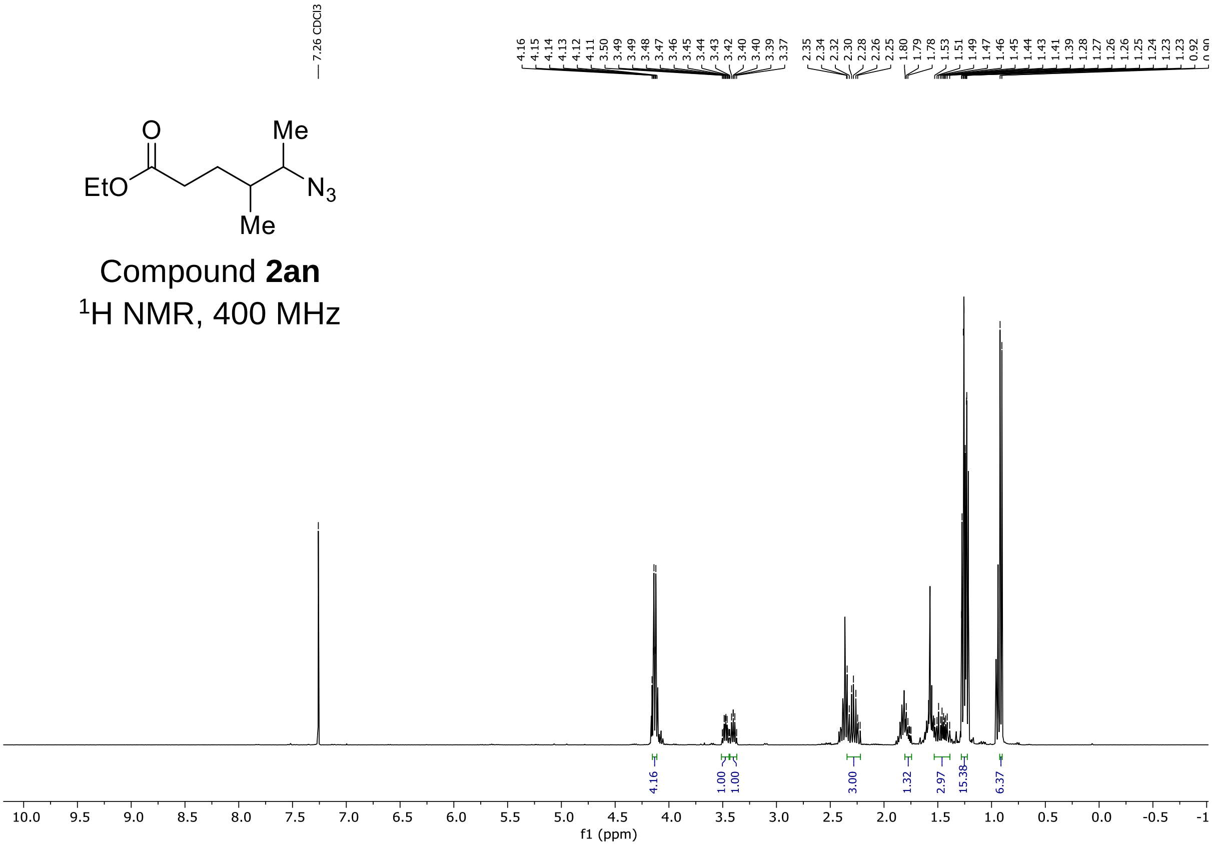
Compound **2an**
^{1}H NMR, 400 MHz
f1 (ppm)

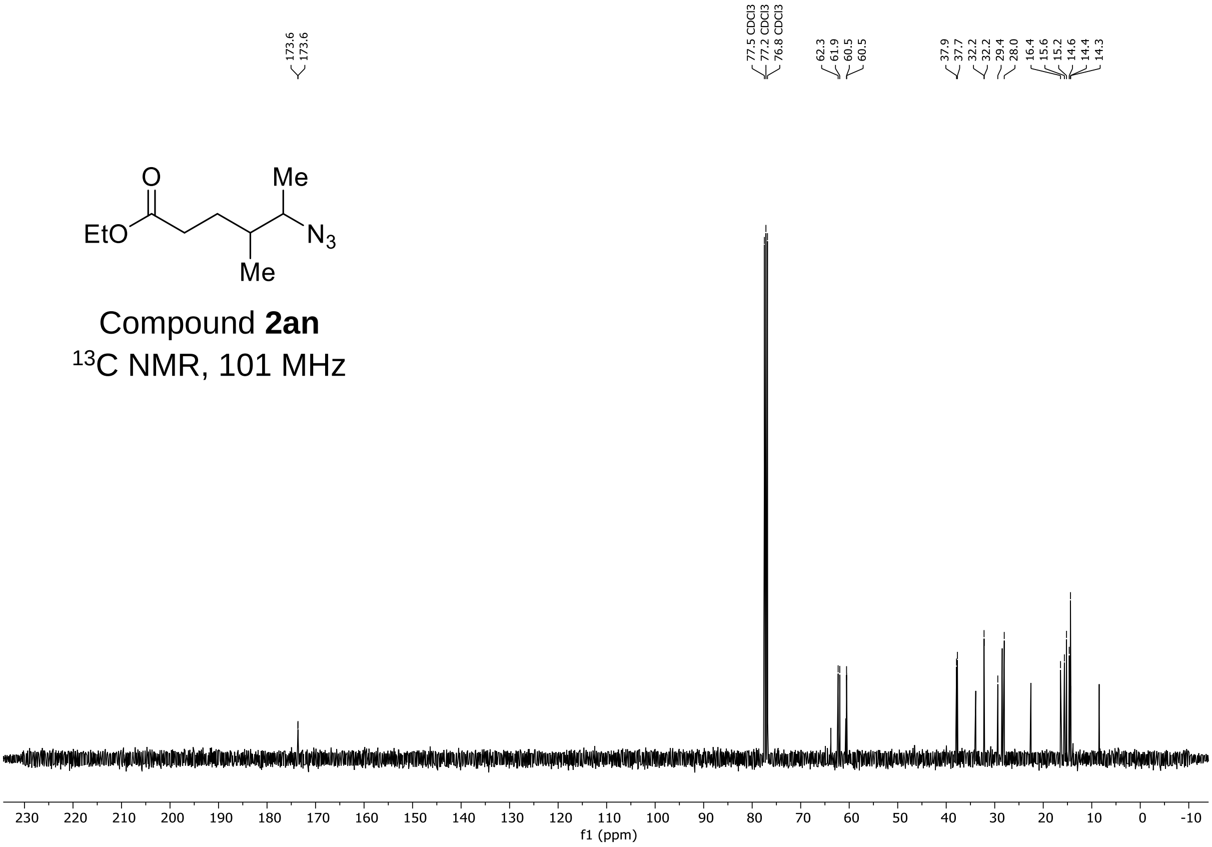
Compound **2an**
^{13}C NMR, 101 MHz
f1 (ppm)

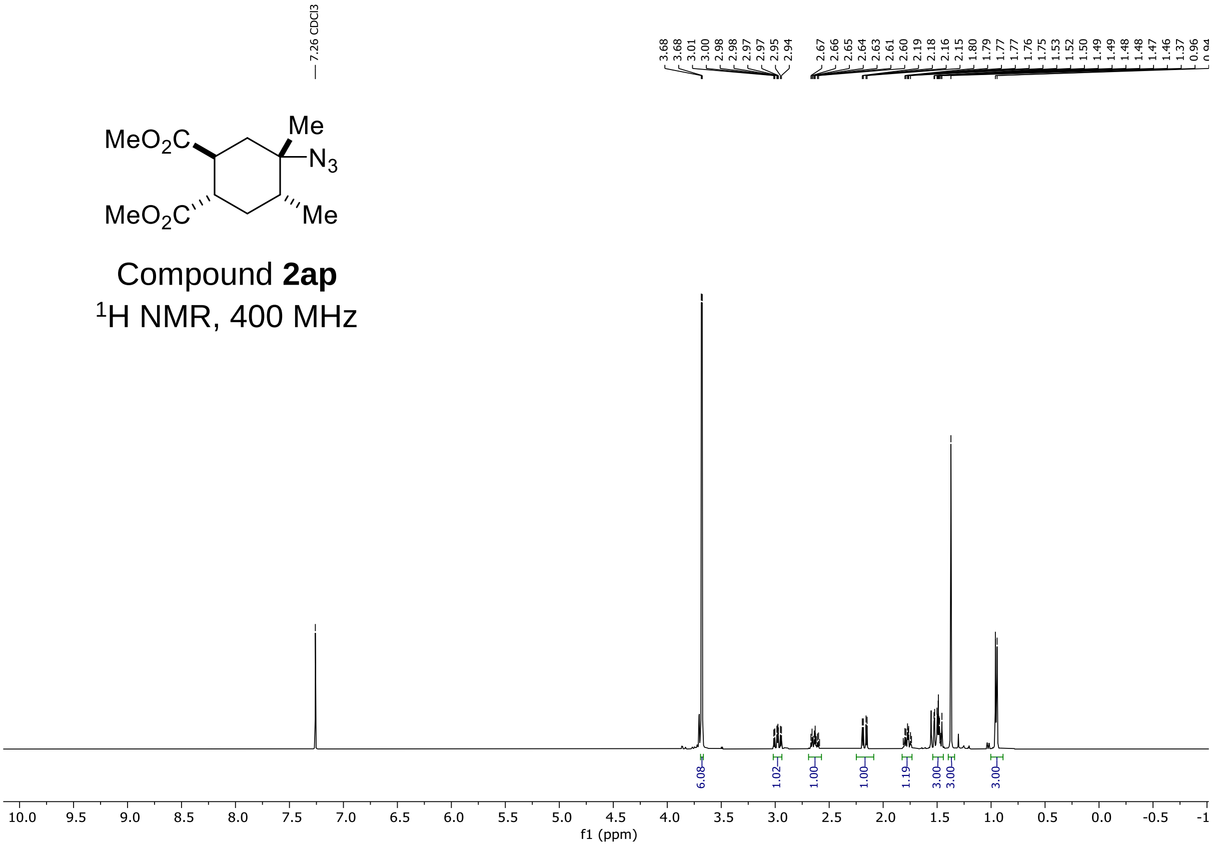

Compound **2ap**
^{1}H NMR, 400 MHz

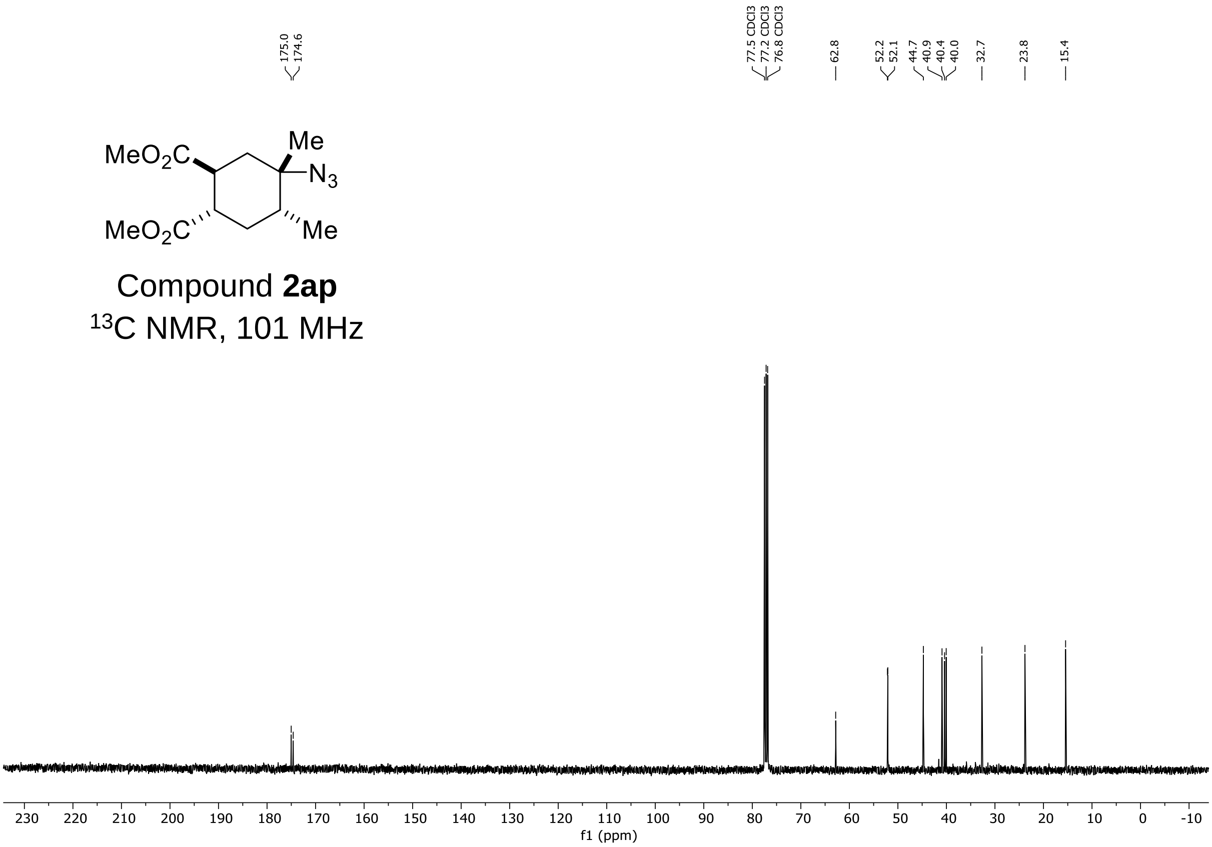

Compound **2ap**
^{13}C NMR, 101 MHz

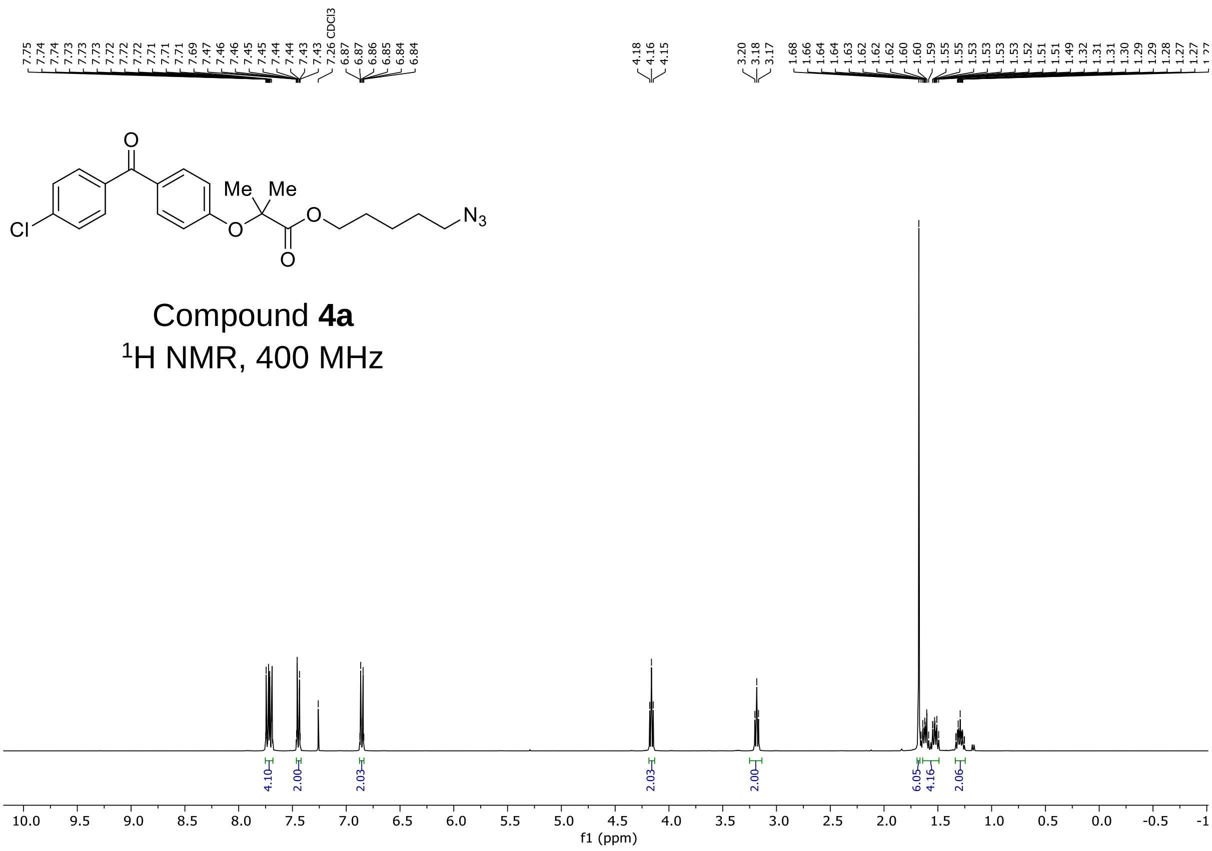

Compound **4a**
^{1}H NMR, 400 MHz

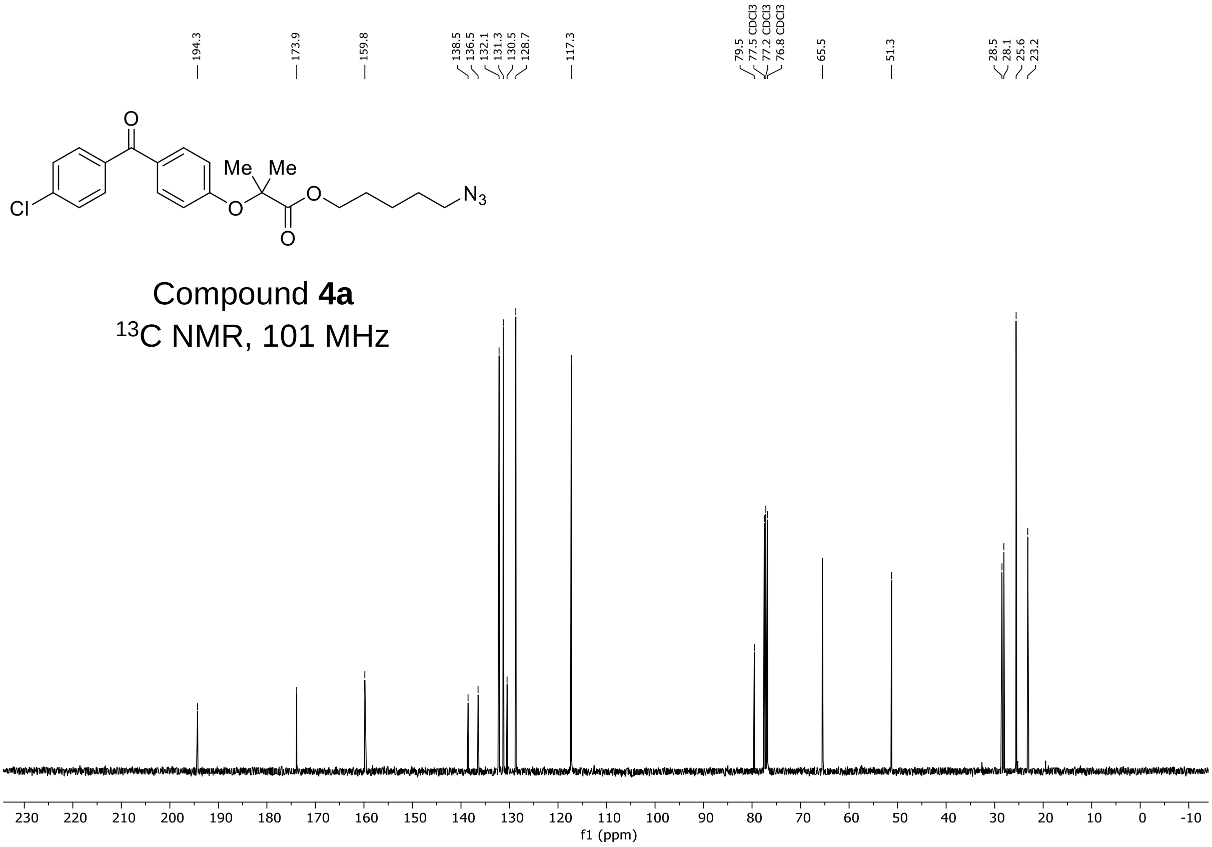

Compound **4a**
^{13}C NMR, 101 MHz

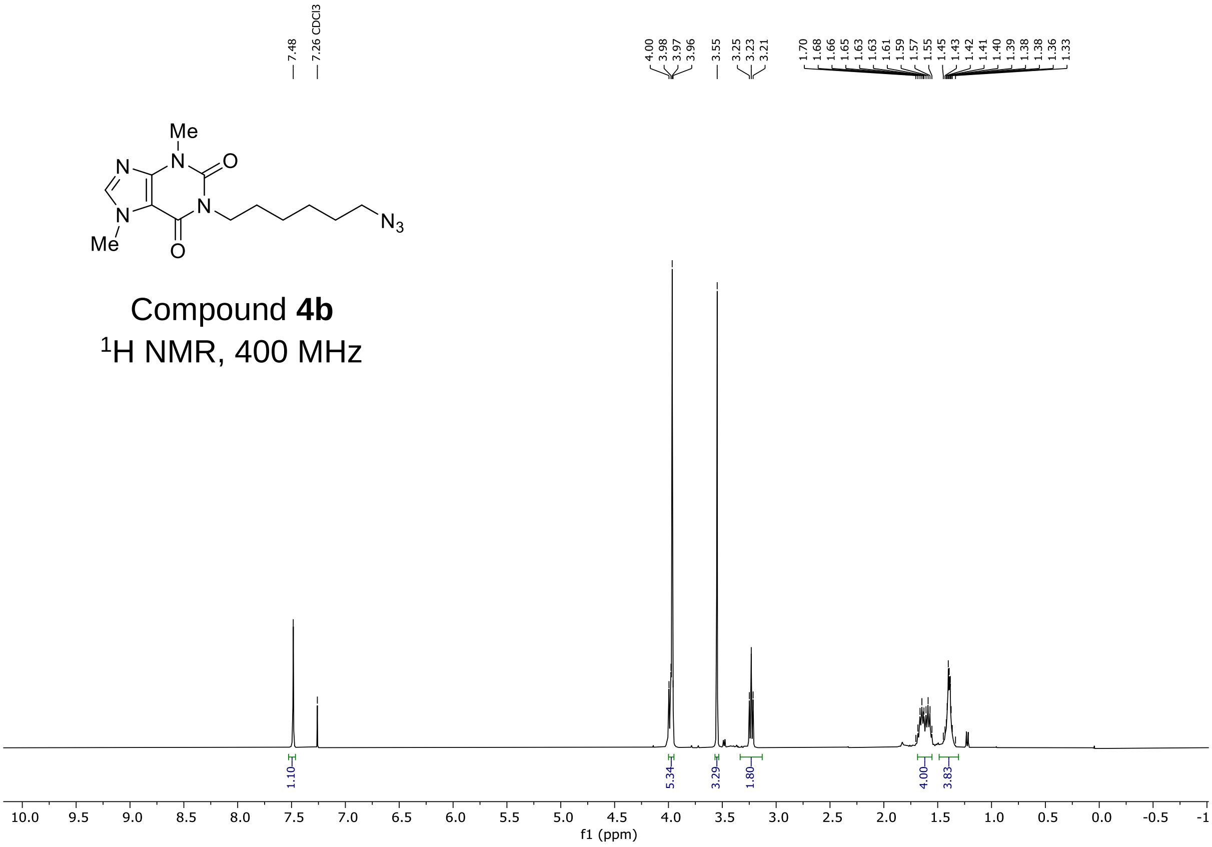
7.48
7.26 CDCl3
4.00
3.98
3.97
3.96
3.55
3.25
3.23
3.21
1.70
1.68
1.66
1.65
1.63
1.63
1.61
1.59
1.57
1.55
1.45
1.43
1.42
1.41
1.40
1.39
1.38
1.38
1.36
1.33
Me
N
O
N
N
N
N3
Me
O
Compound 4b
1H NMR, 400 MHz
1.10
5.34
3.29
1.80
4.00
3.83
10.0 9.5 9.0 8.5 8.0 7.5 7.0 6.5 6.0 5.5 5.0 4.5 4.0 3.5 3.0 2.5 2.0 1.5 1.0 0.5 0.0 -0.5 -1
f1 (ppm)

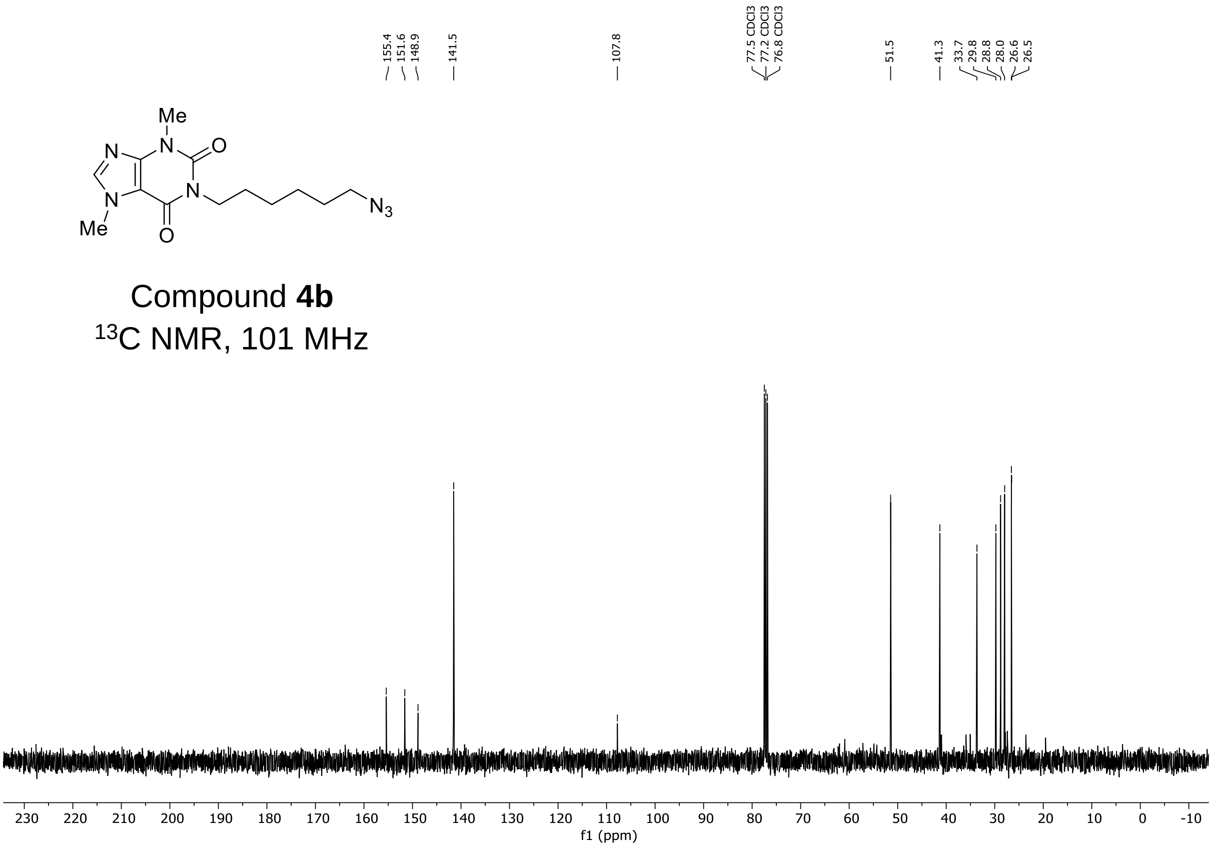
155.4
151.6
148.9
141.5
107.8
77.5 CDCl3
77.2 CDCl3
76.8 CDCl3
51.5
41.3
33.7
29.8
28.8
28.0
26.6
26.5
Me
N
O
N
N
N
N3
Me
O
Compound 4b
13C NMR, 101 MHz
230 220 210 200 190 180 170 160 150 140 130 120 110 100 90 80 70 60 50 40 30 20 10 0 -10
f1 (ppm)

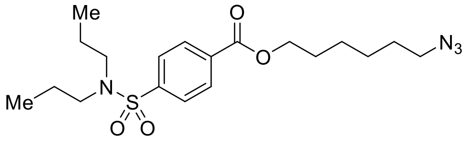

Compound **4c**
^{1}H NMR, 400 MHz

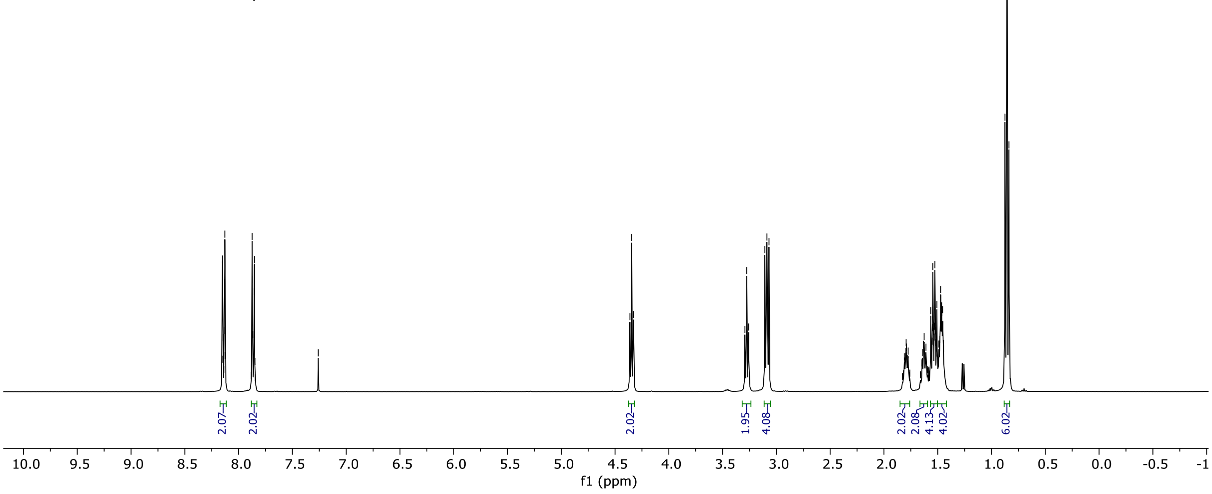

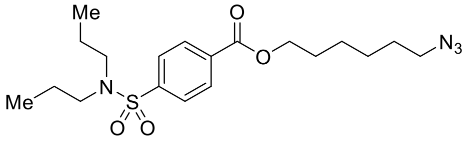

Compound **4c**
^{13}C NMR, 101 MHz

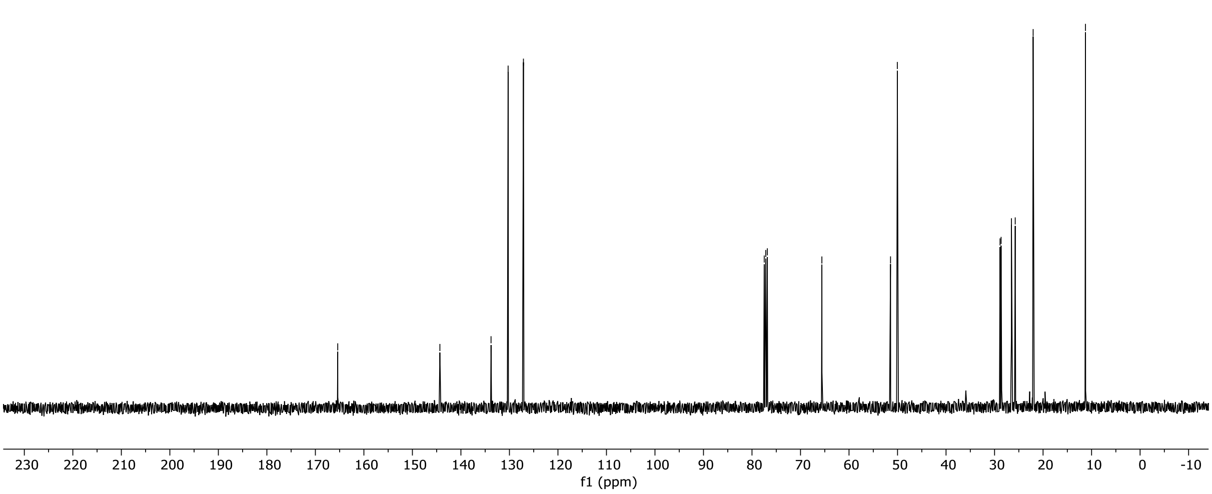

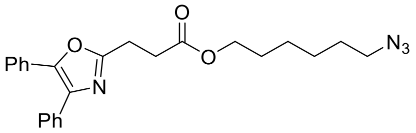

Compound **4d**

^1H NMR, 400 MHz

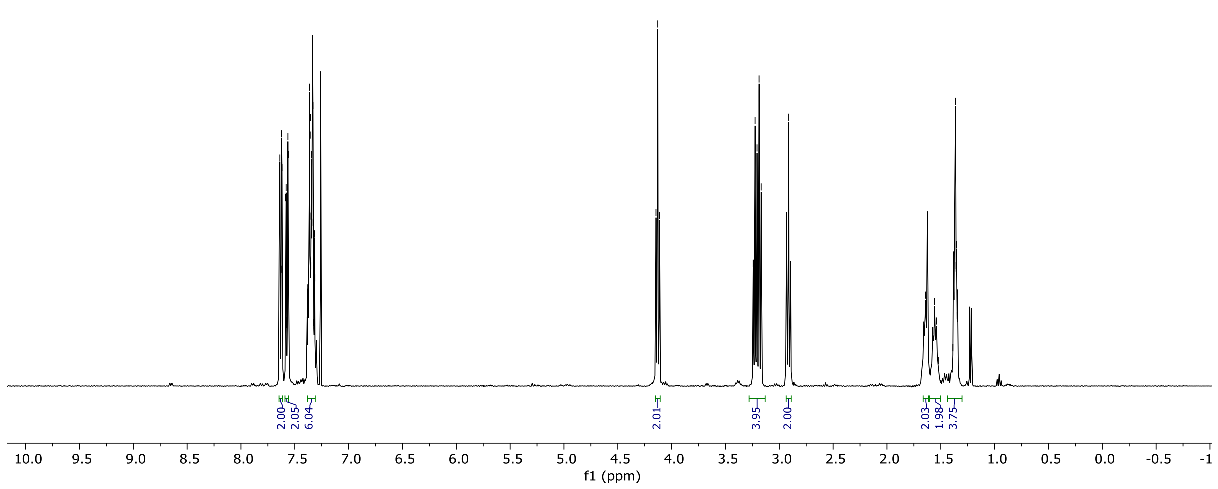

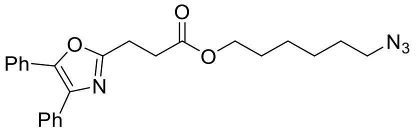

Compound **4d**

^{13}C NMR, 101 MHz

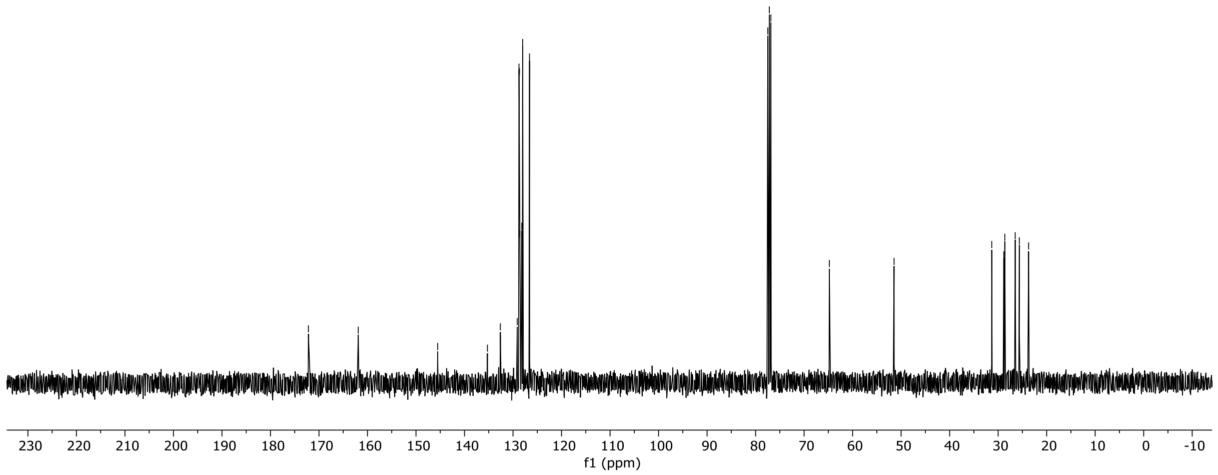

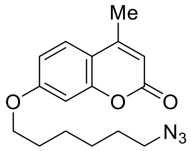

Compound **4e**
^{1}H NMR, 400 MHz

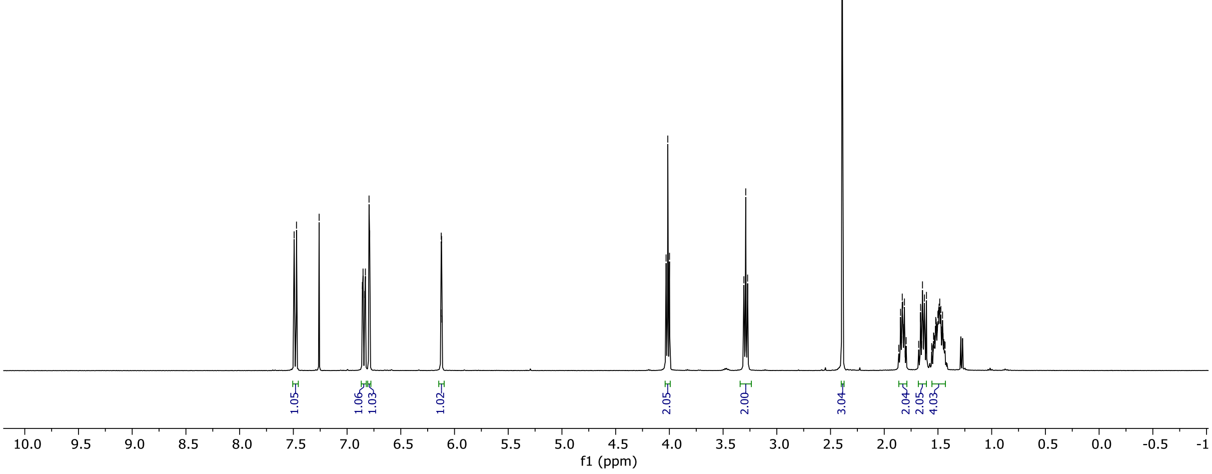

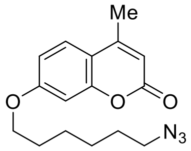

Compound **4e**
^{13}C NMR, 101 MHz

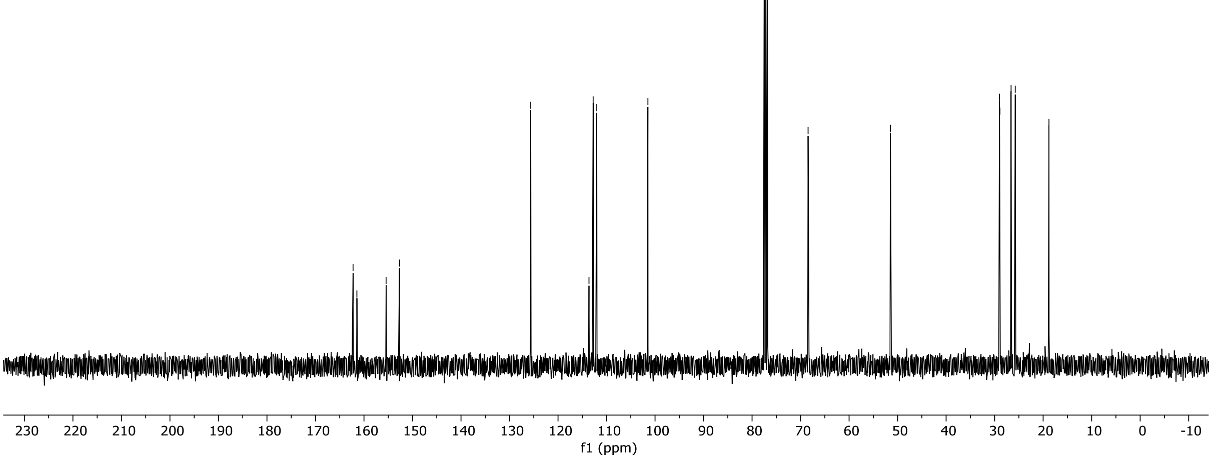

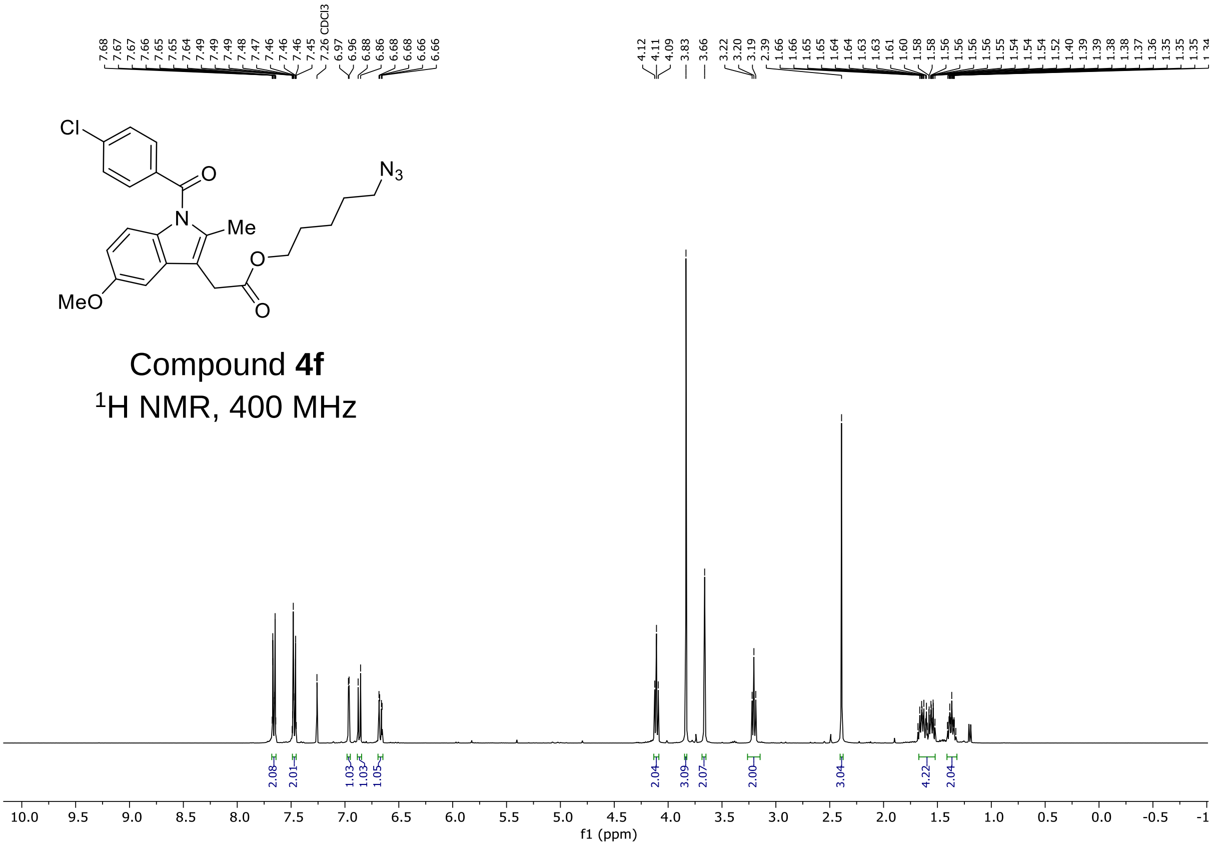
7.68 7.67 7.67 7.66 7.65 7.65 7.64 7.49 7.49 7.48 7.47 7.46 7.46 7.46 7.45 7.26 CDCl3 6.97 6.96 6.88 6.86 6.68 6.68 6.66 6.66
4.12 4.11 4.09 3.83 3.66 3.22 3.20 3.19 2.39 1.66 1.66 1.65 1.65 1.64 1.64 1.63 1.63 1.61 1.60 1.58 1.58 1.56 1.56 1.56 1.55 1.54 1.54 1.54 1.52 1.40 1.39 1.39 1.38 1.38 1.37 1.36 1.35 1.35 1.35
Cl
O
N
Me
N3
O
O
MeO
Compound **4f**
^{1}H NMR, 400 MHz
2.08 2.01 1.03 1.03 1.05 2.04 3.09 2.07 2.00 3.04 4.22 2.04
f1 (ppm)

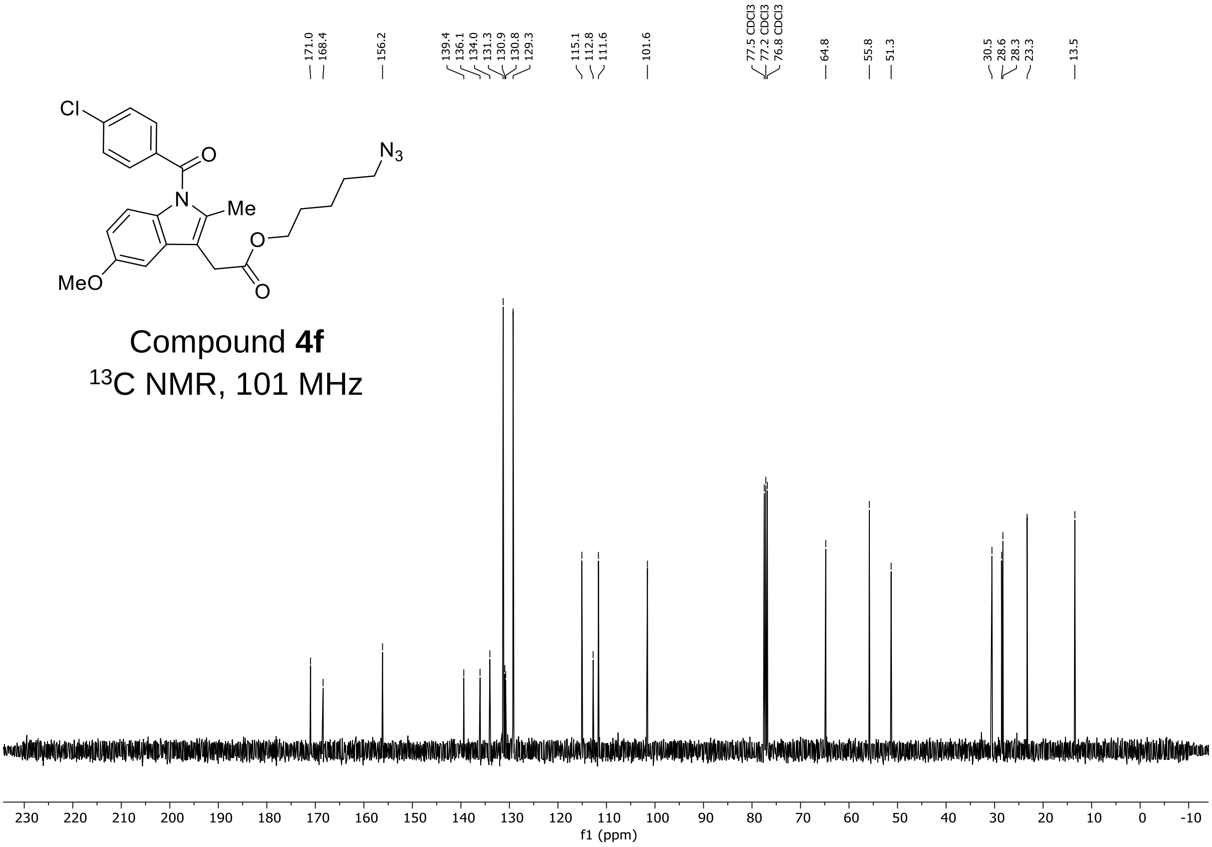
171.0 168.4 156.2 139.4 136.1 134.0 131.3 130.9 130.8 129.3 115.1 112.8 111.6 101.6 77.5 CDCl3 77.2 CDCl3 76.8 CDCl3 64.8 55.8 51.3 30.5 28.6 28.3 23.3 13.5
Cl
O
N
Me
N3
O
O
MeO
Compound **4f**
^{13}C NMR, 101 MHz
f1 (ppm)

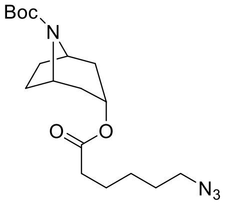

Compound **4g**
^{1}H NMR, 400 MHz

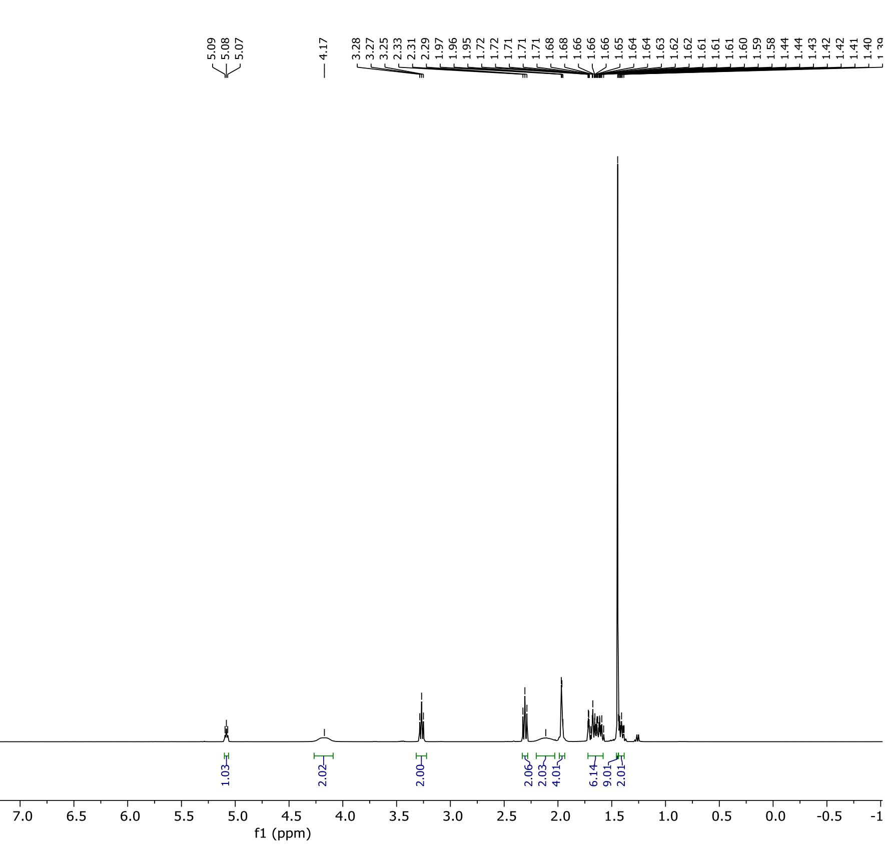

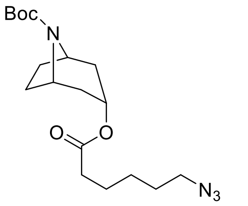

Compound **4g**
^{13}C NMR, 101 MHz

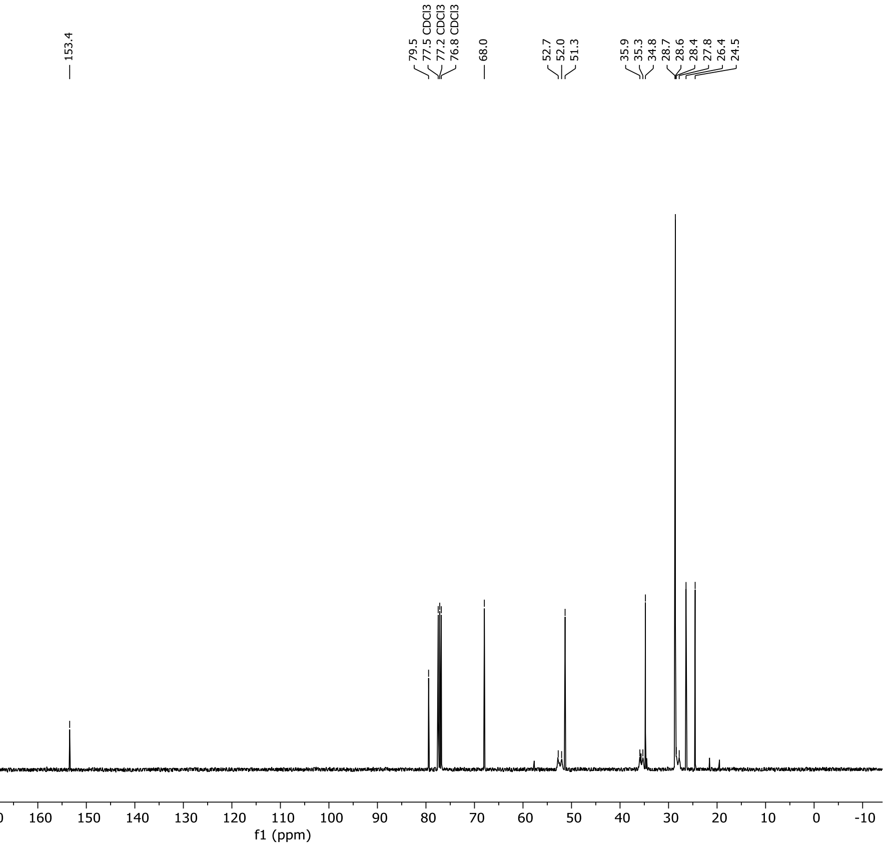

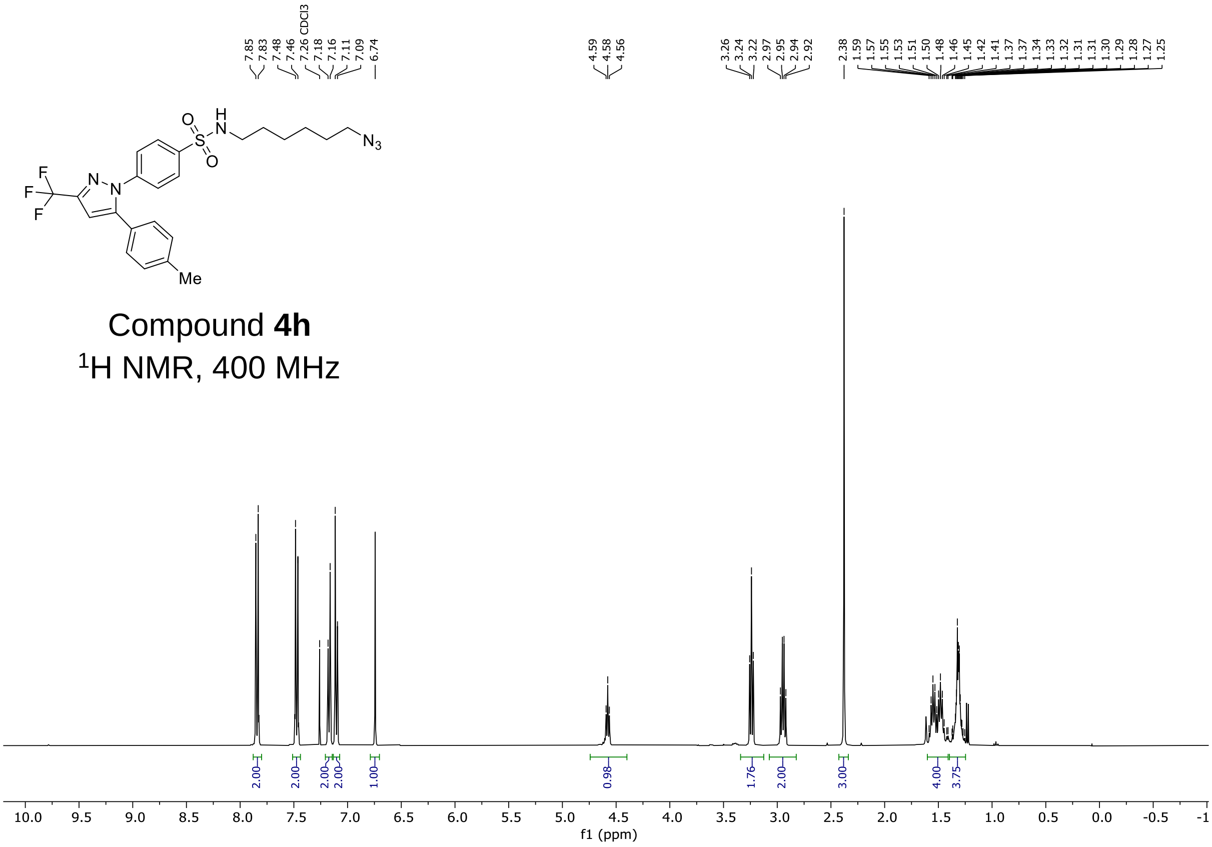

Compound **4h**
^{1}H NMR, 400 MHz

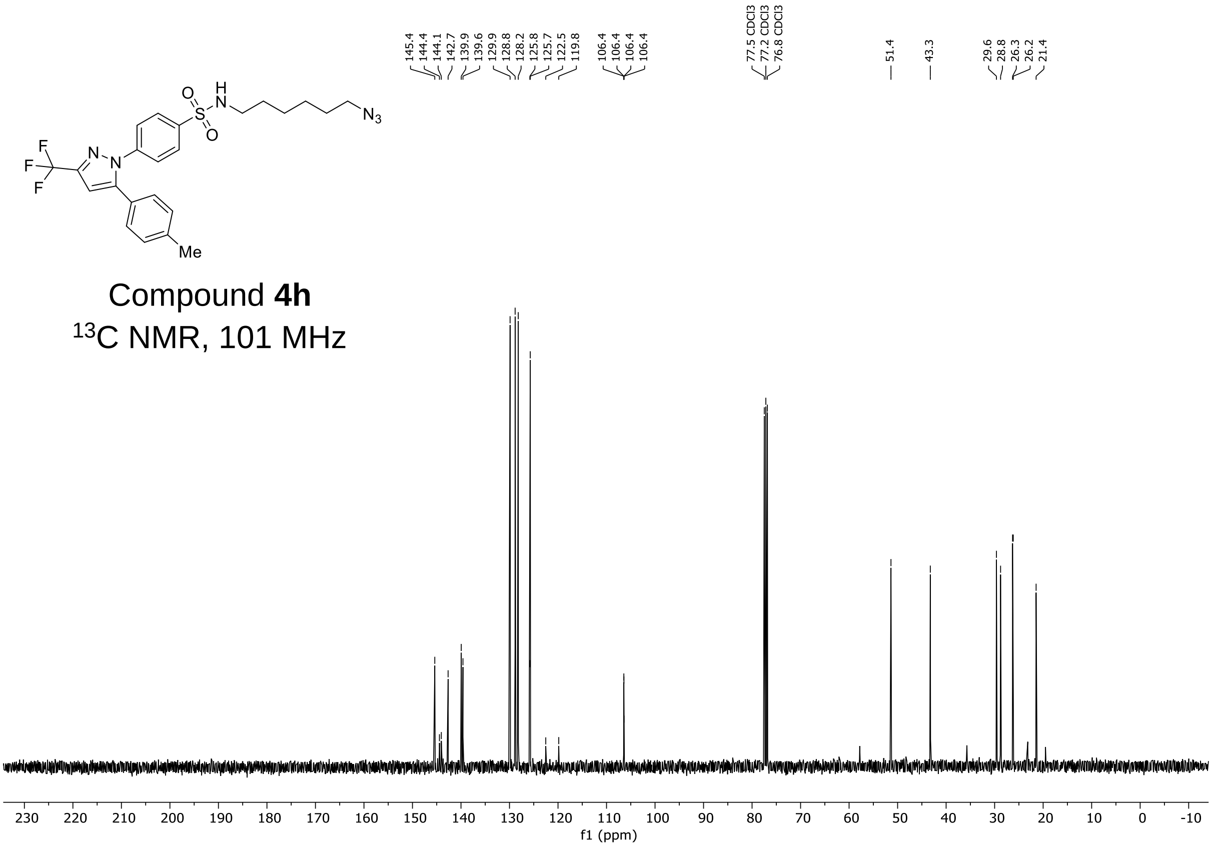

Compound **4h**
^{13}C NMR, 101 MHz

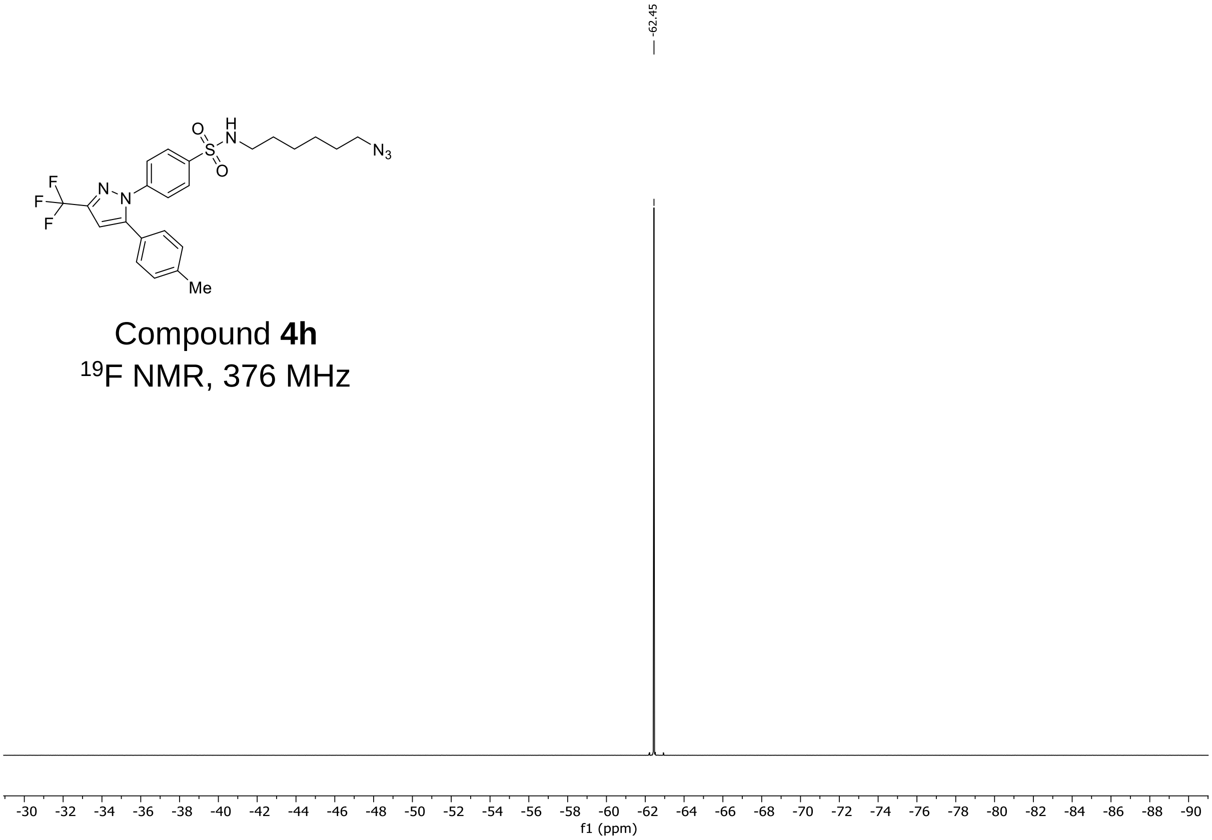

Compound **4h**
^{19}F NMR, 376 MHz

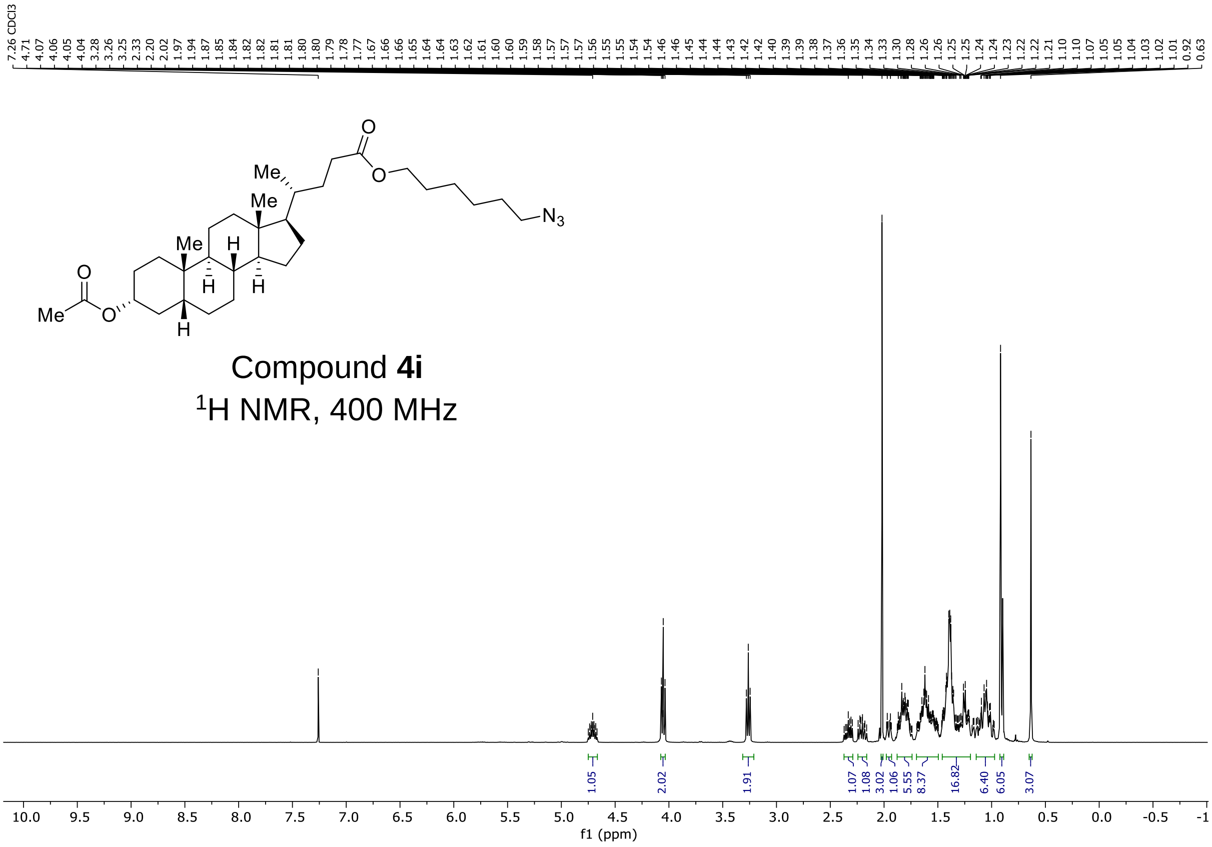
7.26 CDCl3
4.71 4.07 4.06 4.05 4.04 3.28 3.26 3.25 2.33 2.20 2.02 1.97 1.94 1.87 1.85 1.84 1.82 1.82 1.81 1.81 1.80 1.79 1.78 1.77 1.67 1.66 1.66 1.65 1.64 1.64 1.63 1.62 1.61 1.60 1.60 1.59 1.58 1.57 1.57 1.57 1.56 1.55 1.55 1.54 1.54 1.46 1.46 1.45 1.44 1.44 1.43 1.42 1.42 1.40 1.39 1.39 1.38 1.37 1.36 1.35 1.34 1.33 1.30 1.28 1.26 1.26 1.25 1.25 1.25 1.24 1.24 1.23 1.22 1.22 1.21 1.10 1.10 1.07 1.05 1.05 1.04 1.03 1.02 1.01 0.92 0.63
Me
Me
Me
H
H
H
H
O
O
O
O
Me
N3
Compound 4i
1H NMR, 400 MHz
1.05 2.02 1.91 1.07 1.08 3.02 1.06 5.55 8.37 16.82 6.40 6.05 3.07
f1 (ppm)

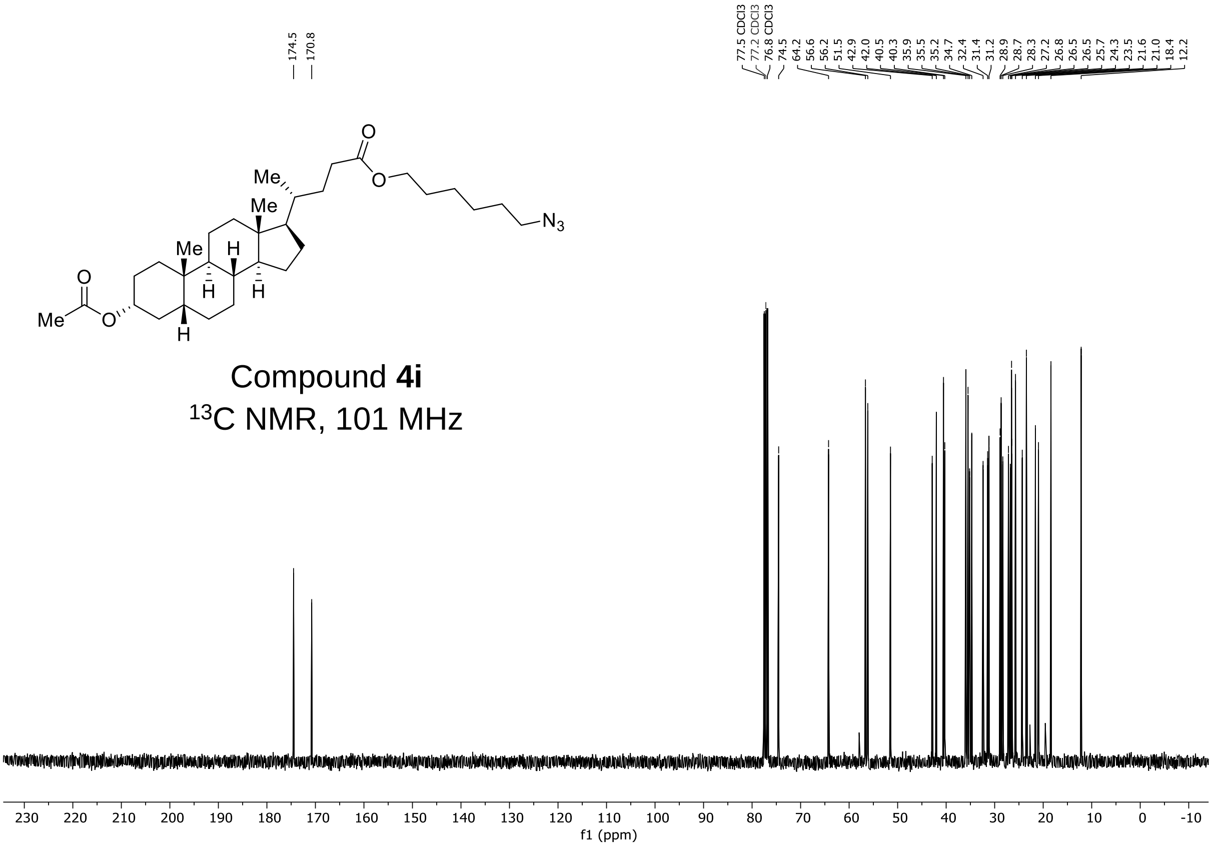
174.5
170.8
77.5 CDCl3
77.2 CDCl3
76.8 CDCl3
74.5 64.2 56.6 56.2 51.5 42.9 42.0 40.5 40.3 35.9 35.5 35.2 34.7 32.4 31.4 31.2 28.9 28.7 28.3 27.2 26.8 26.5 25.7 24.3 23.5 21.6 21.0 18.4 12.2
Me
Me
Me
H
H
H
H
O
O
O
O
Me
N3
Compound 4i
13C NMR, 101 MHz
f1 (ppm)

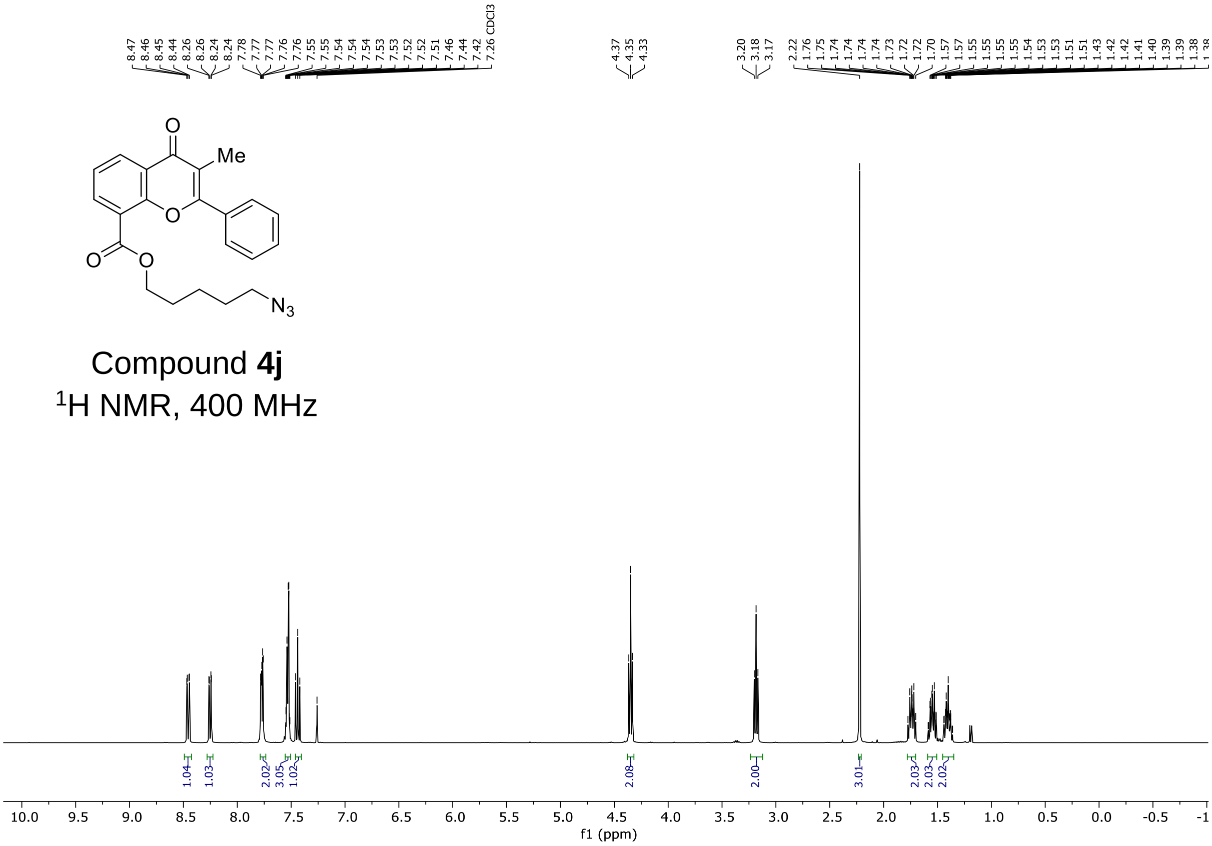

Compound **4j**
^{1}H NMR, 400 MHz

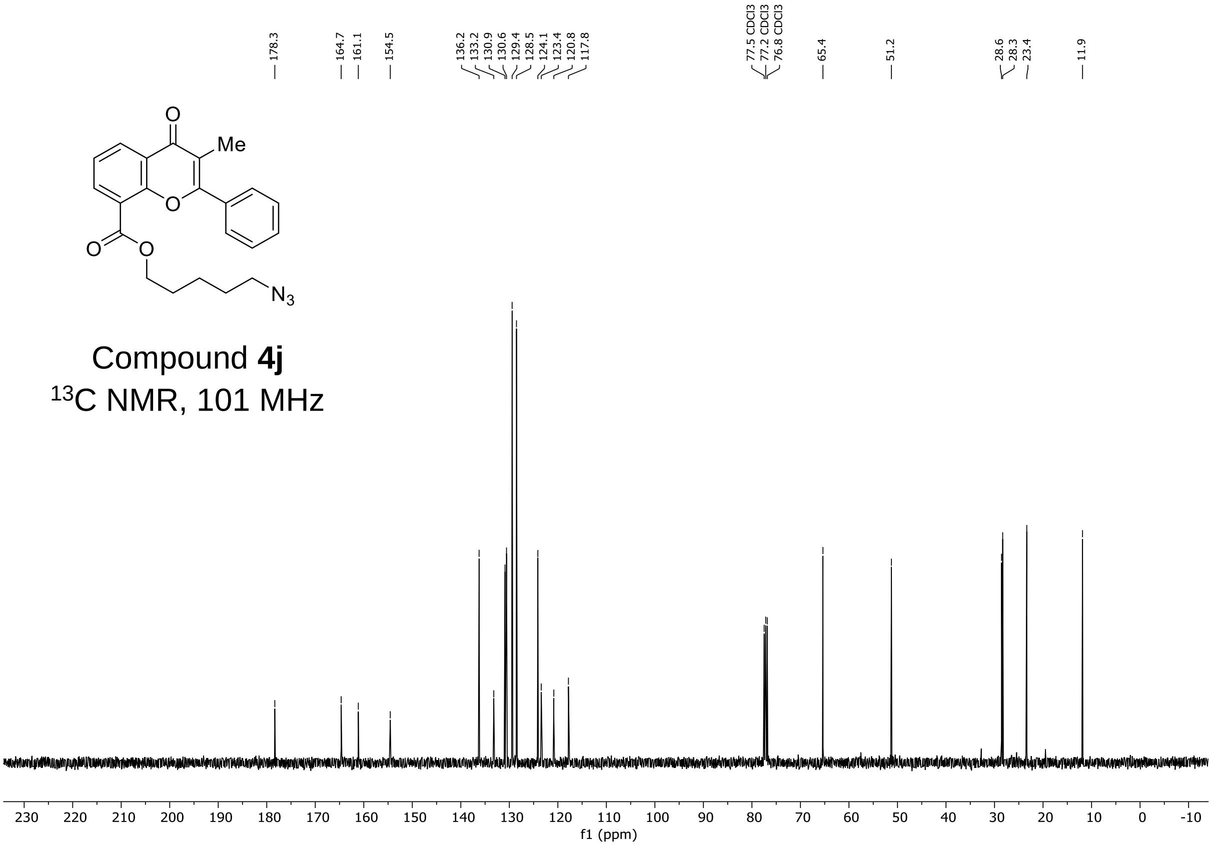

Compound **4j**
^{13}C NMR, 101 MHz

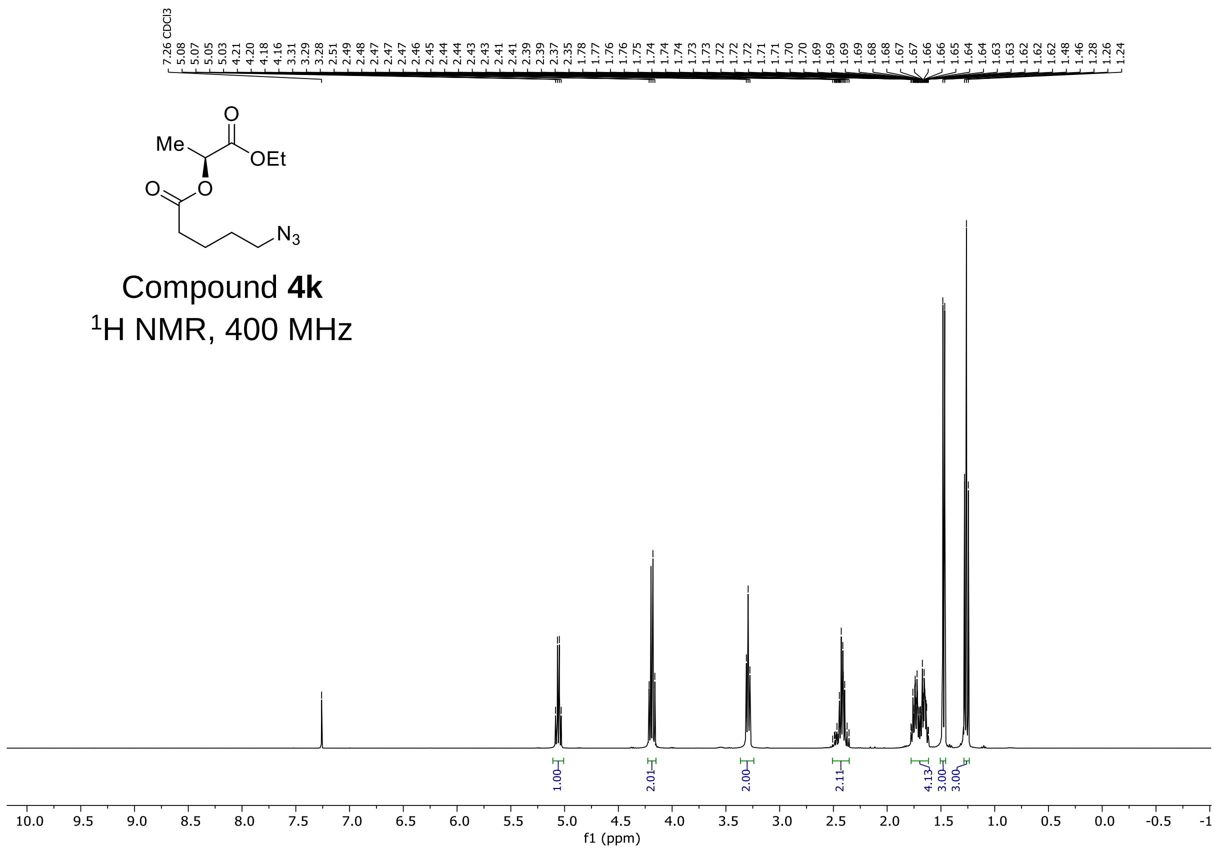

Compound **4k**
^1H NMR, 400 MHz

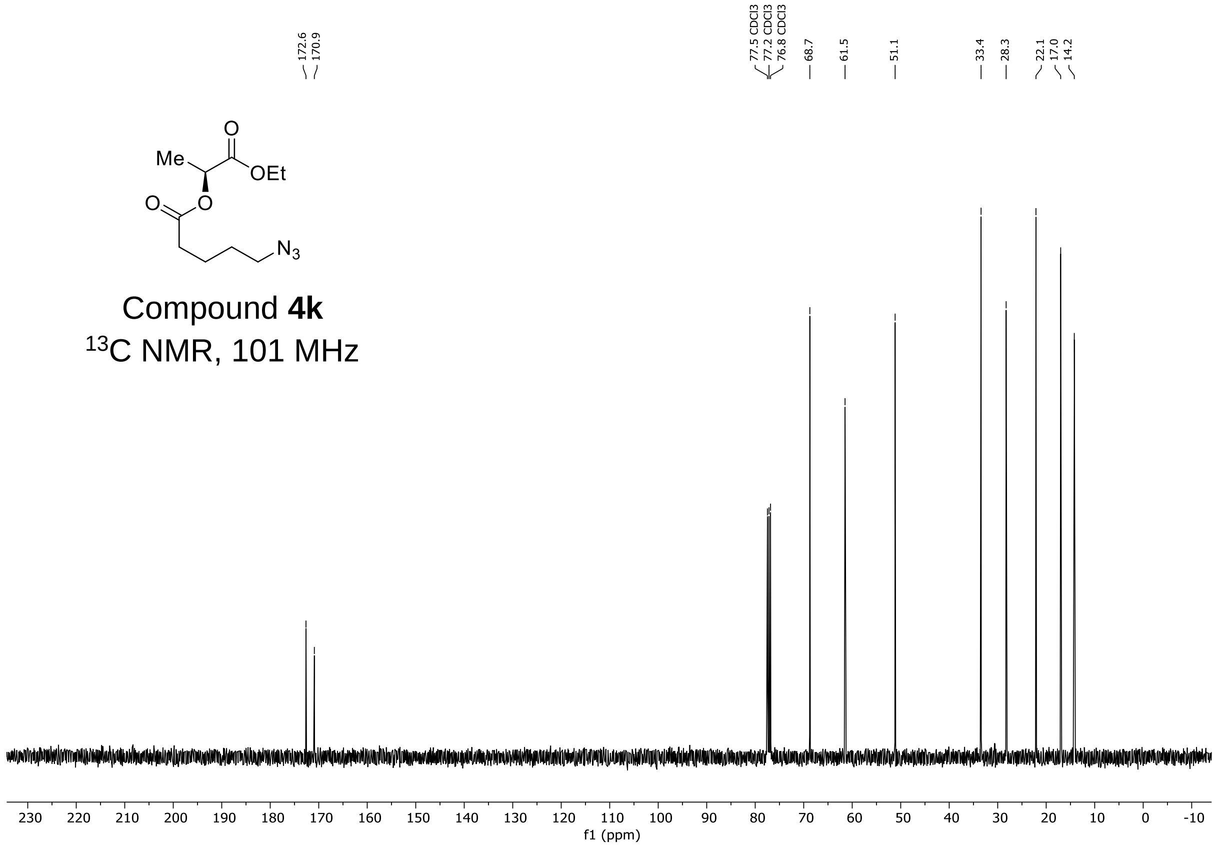

Compound **4k**
^{13}C NMR, 101 MHz

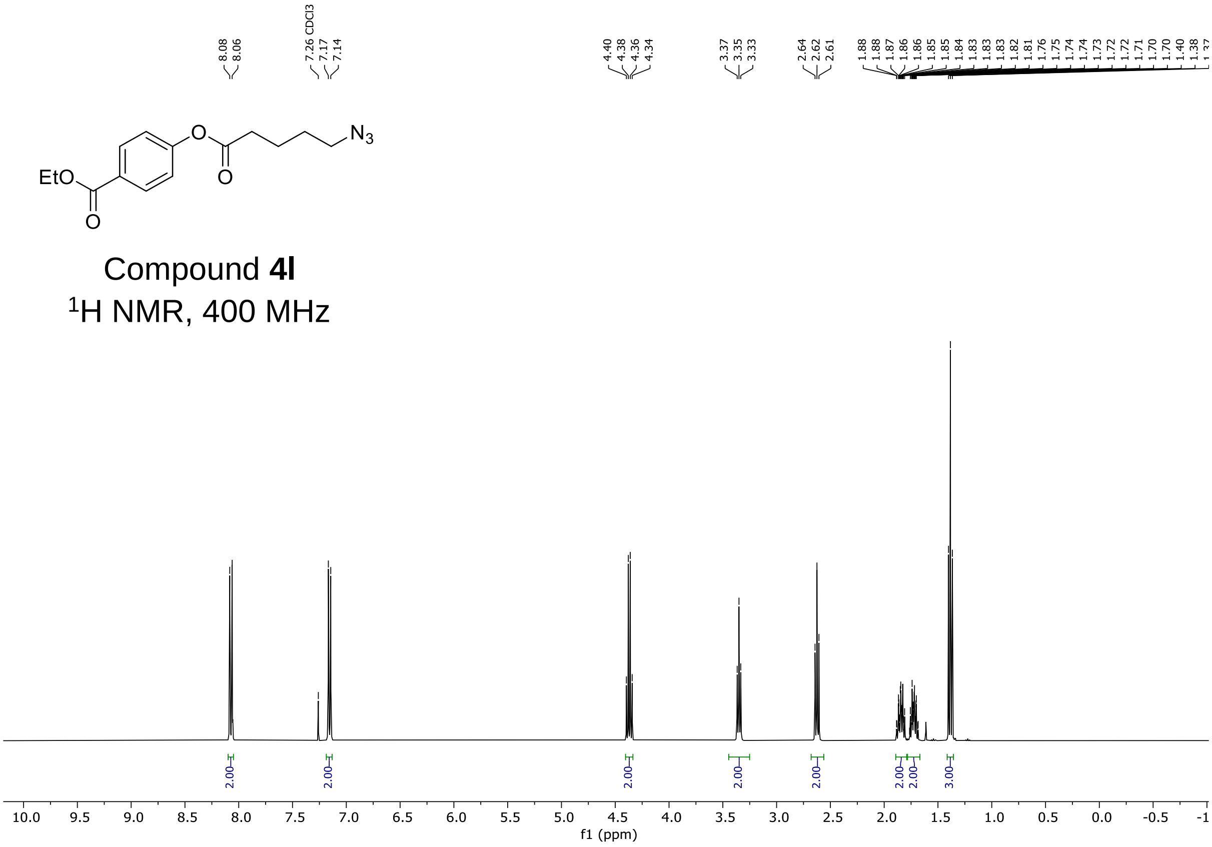
8.08
8.06
7.26 CDCl3
7.17
7.14
4.40
4.38
4.36
4.34
3.37
3.35
3.33
2.64
2.62
2.61
EtO
O
O
O
N3
Compound 4l
1H NMR, 400 MHz
2.00
2.00
2.00
2.00
2.00
2.00
2.00
3.00
f1 (ppm)

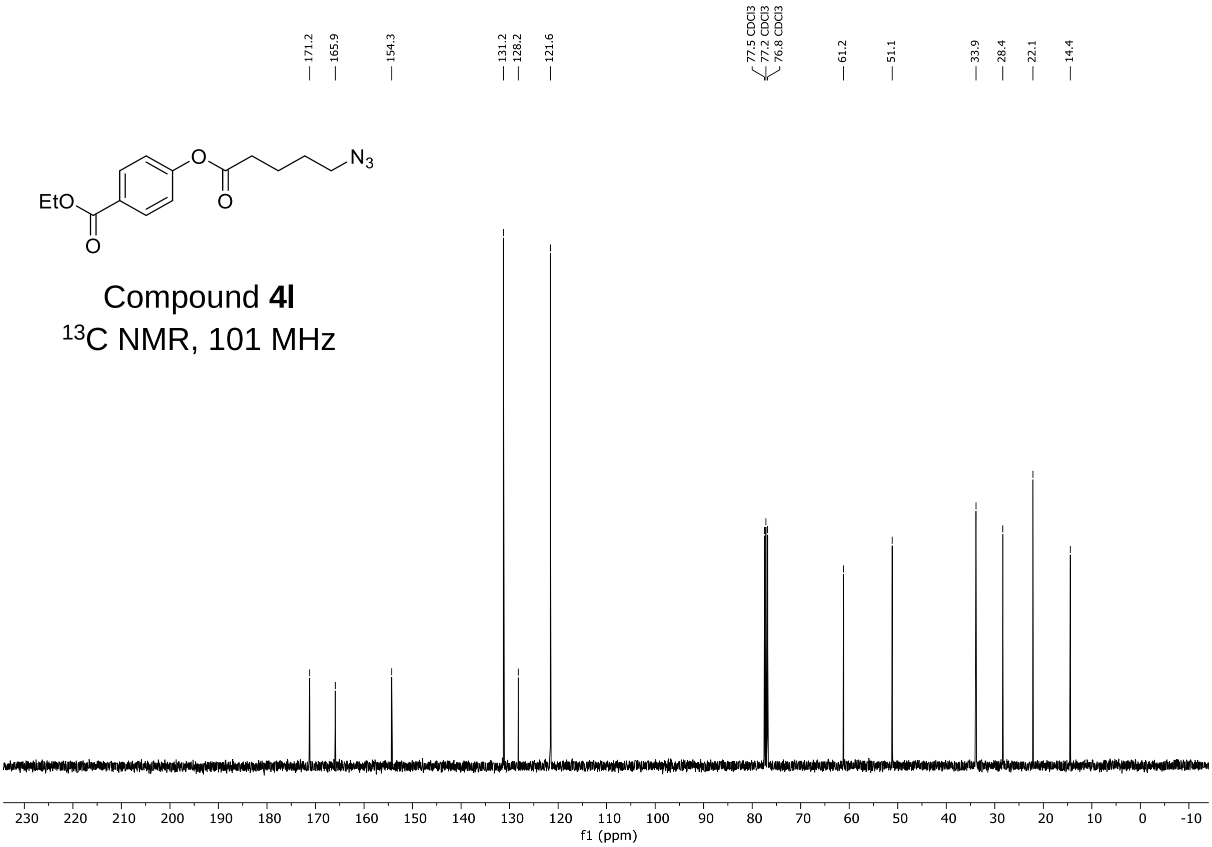
171.2
165.9
154.3
131.2
128.2
121.6
77.5 CDCl3
77.2 CDCl3
76.8 CDCl3
61.2
51.1
33.9
28.4
22.1
14.4
EtO
O
O
O
N3
Compound 4l
13C NMR, 101 MHz
f1 (ppm)

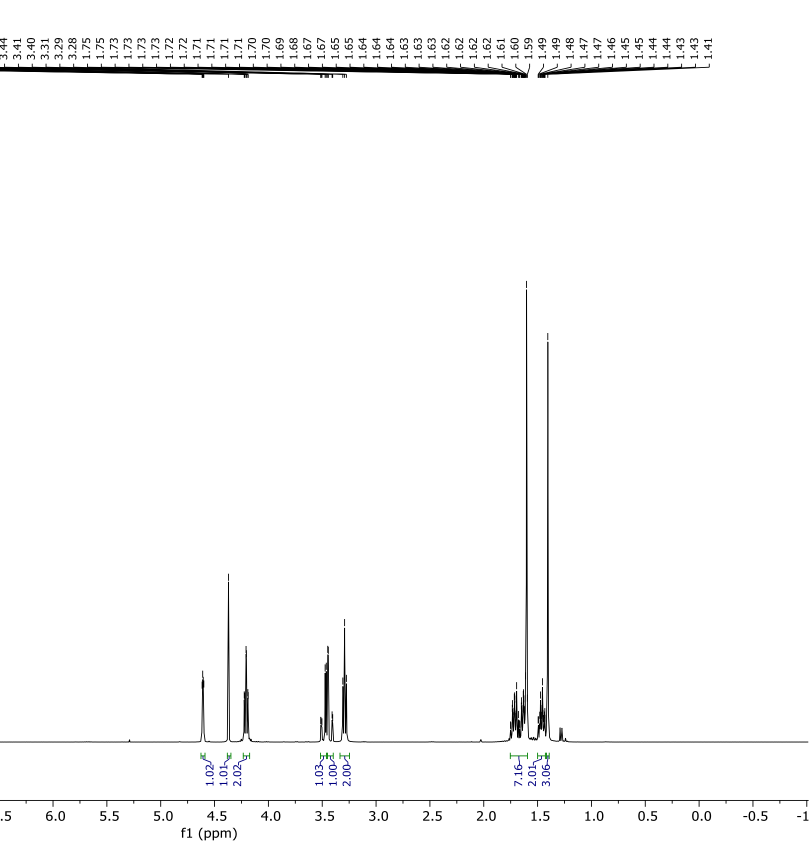

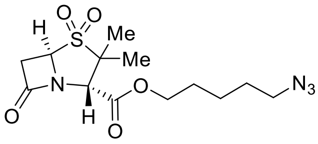

Compound **4m**
^{1}H NMR, 400 MHz

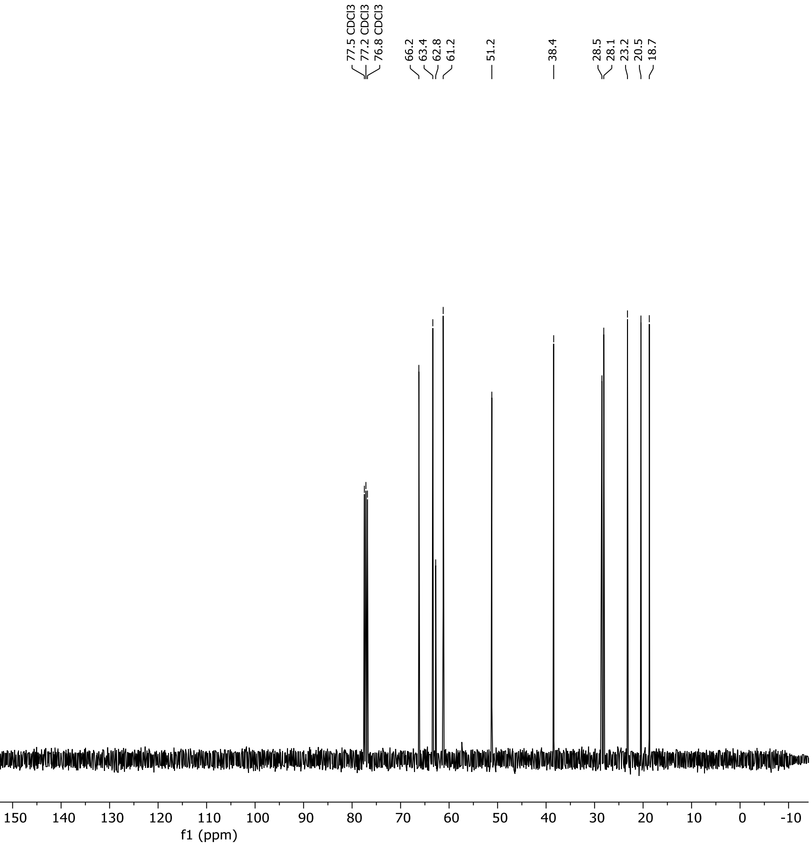

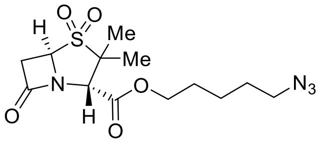

Compound **4m**
^{13}C NMR, 101 MHz

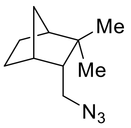

Compound **4n**
^{1}H NMR, 400 MHz

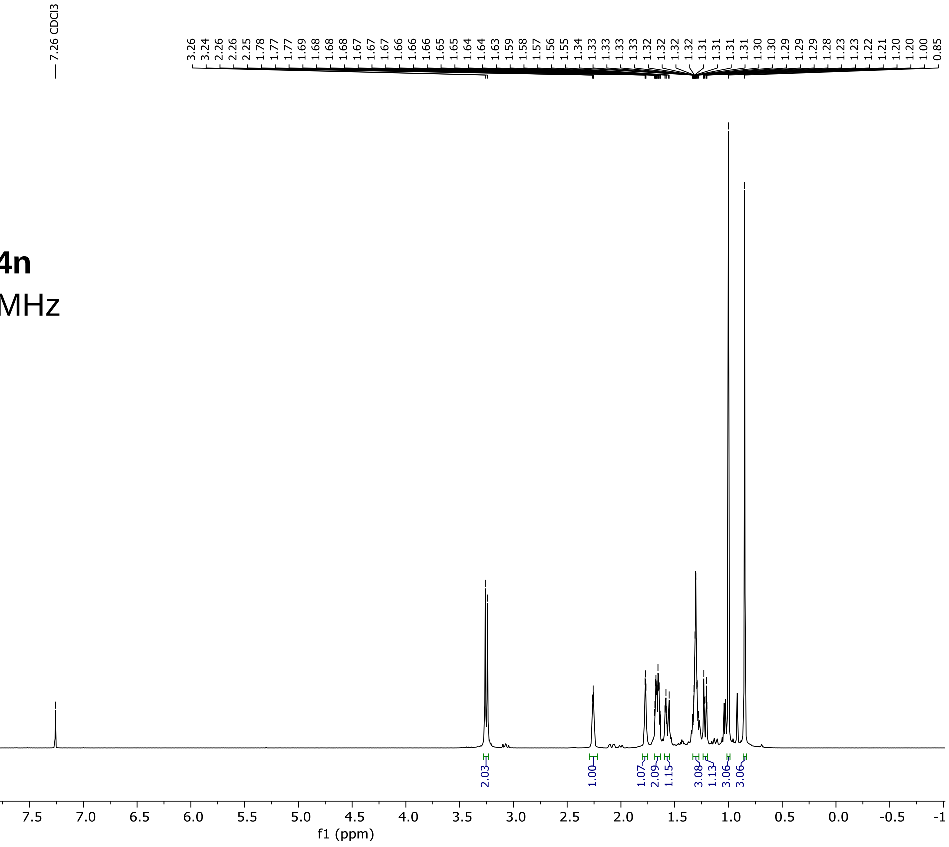

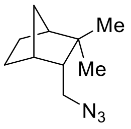

Compound **4n**
^{13}C NMR, 101 MHz

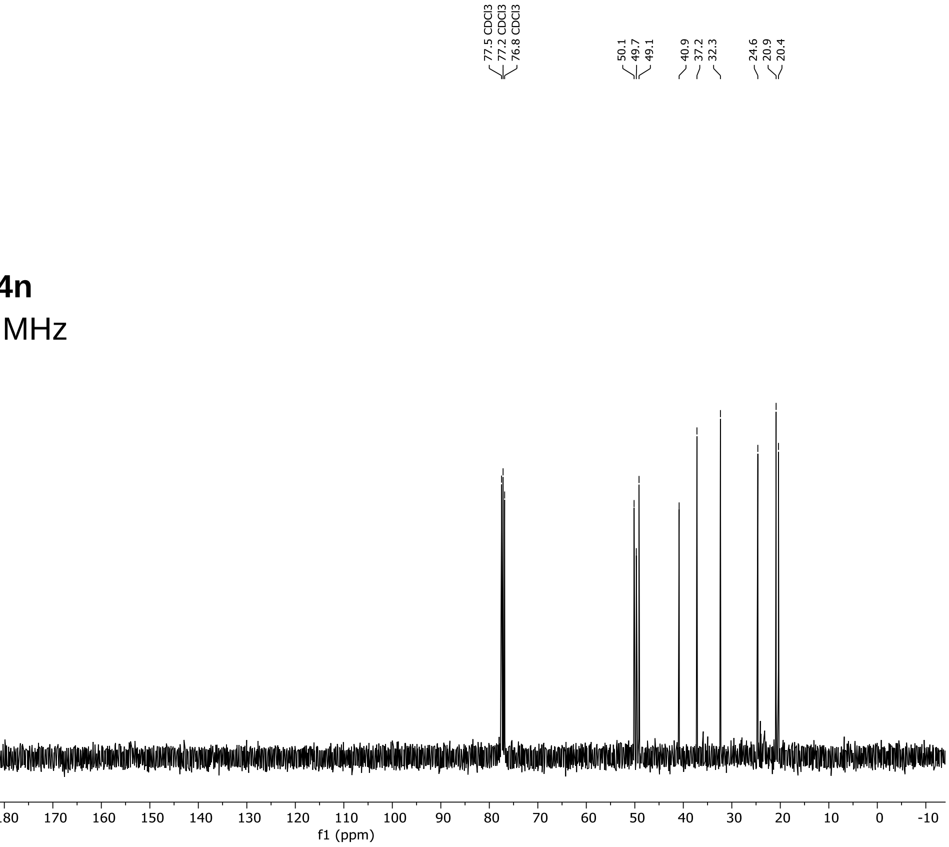

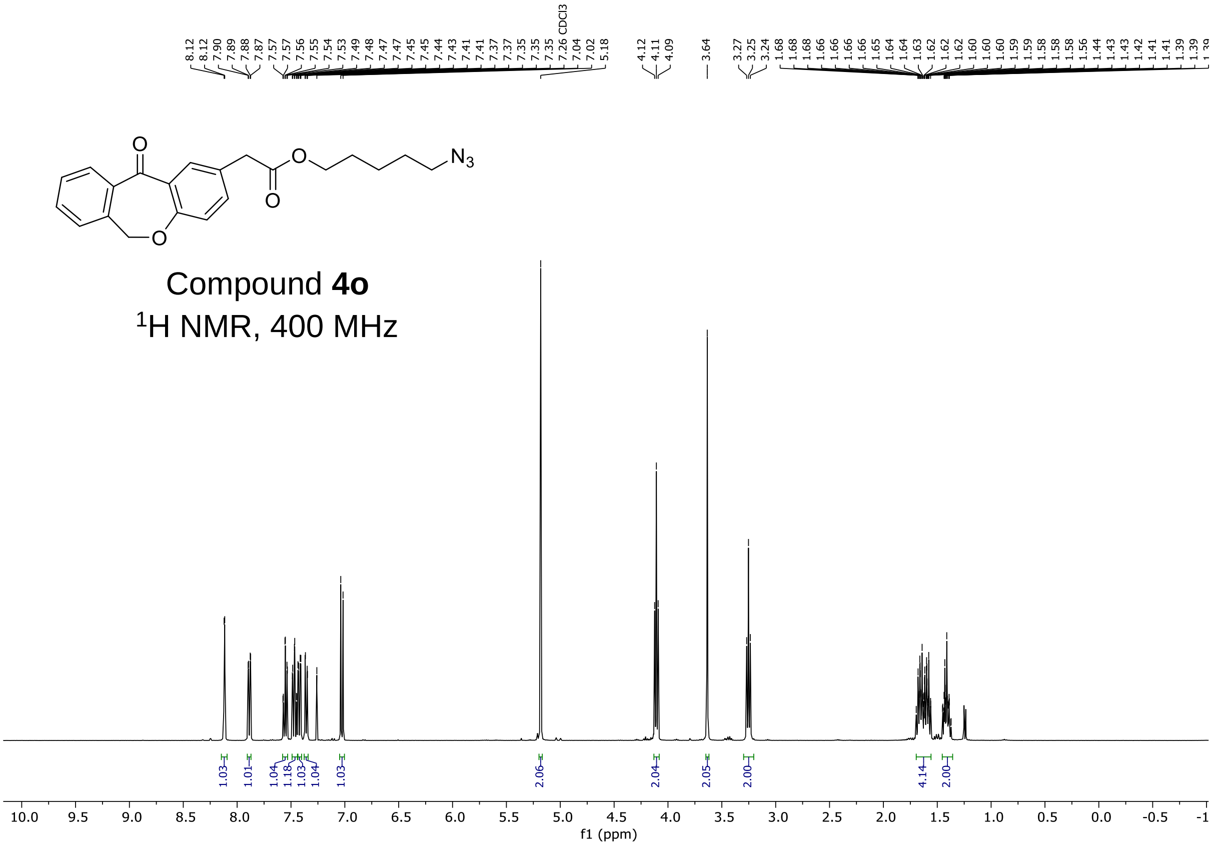
8.12
8.12
7.90
7.89
7.88
7.87
7.57
7.57
7.56
7.55
7.54
7.53
7.49
7.48
7.47
7.45
7.45
7.44
7.43
7.41
7.41
7.37
7.37
7.35
7.35
7.26 CDCl3
7.04
7.02
5.18
4.12
4.11
4.09
3.64
3.27
3.25
3.24
1.68
1.68
1.66
1.66
1.66
1.65
1.64
1.64
1.63
1.62
1.62
1.62
1.60
1.60
1.60
1.59
1.59
1.58
1.58
1.58
1.56
1.44
1.43
1.43
1.42
1.41
1.41
1.39
1.39
O
O
O
O
N3
Compound 4o
1H NMR, 400 MHz
1.03
1.01
1.04
1.18
1.03
1.04
1.03
2.06
2.04
2.05
2.00
4.14
2.00
10.0 9.5 9.0 8.5 8.0 7.5 7.0 6.5 6.0 5.5 5.0 4.5 4.0 3.5 3.0 2.5 2.0 1.5 1.0 0.5 0.0 -0.5 -1
f1 (ppm)

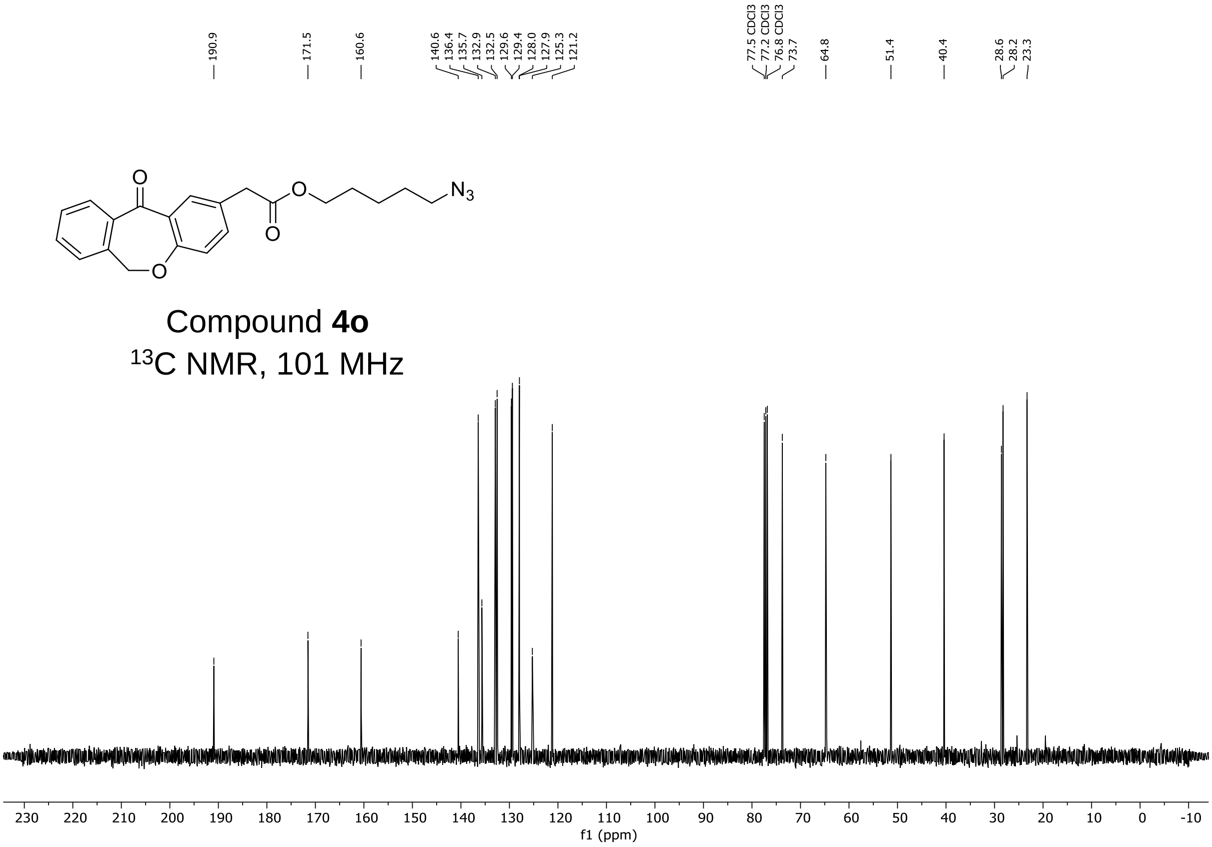
190.9
171.5
160.6
140.6
136.4
135.7
132.9
132.5
129.6
129.4
128.0
127.9
125.3
121.2
77.5 CDCl3
77.2 CDCl3
76.8 CDCl3
73.7
64.8
51.4
40.4
28.6
28.2
23.3
O
O
O
O
N3
Compound 4o
13C NMR, 101 MHz
230 220 210 200 190 180 170 160 150 140 130 120 110 100 90 80 70 60 50 40 30 20 10 0 -10
f1 (ppm)

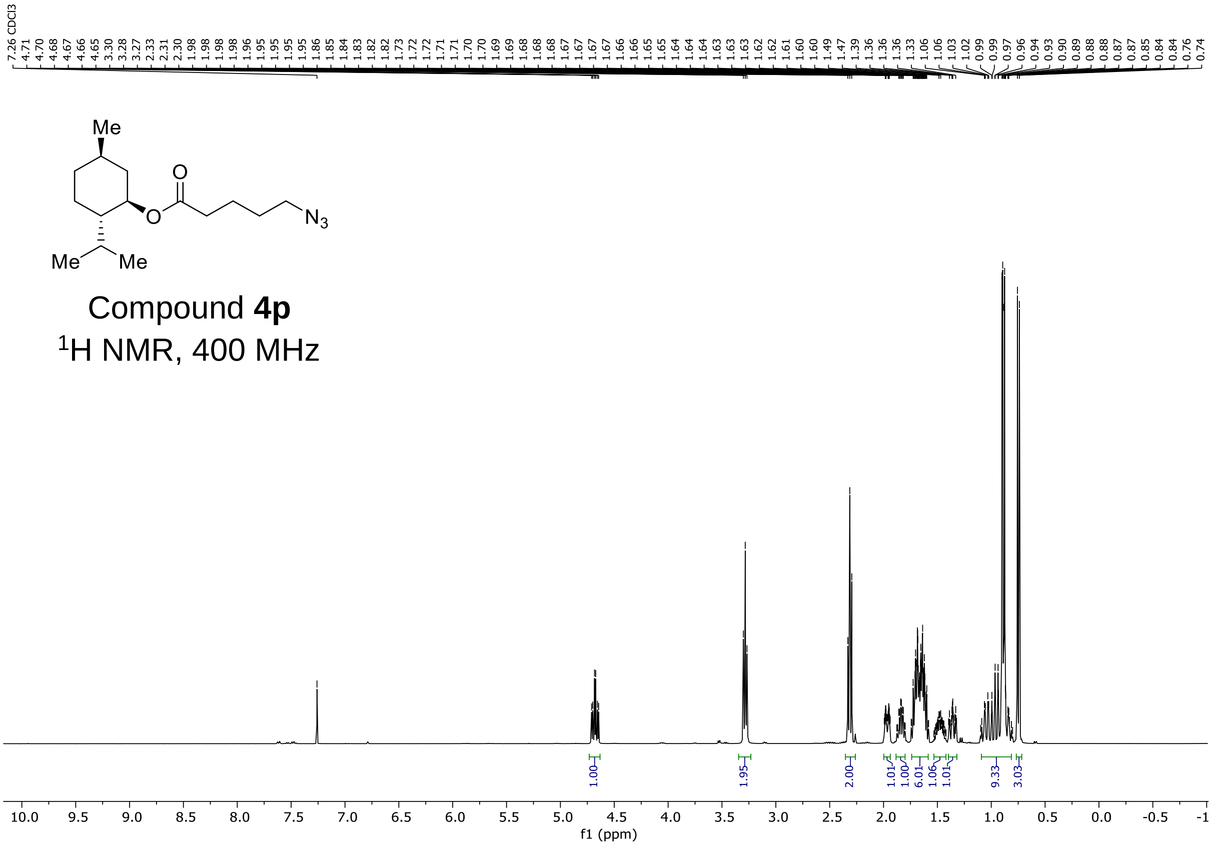

Compound **4p**
^{1}H NMR, 400 MHz

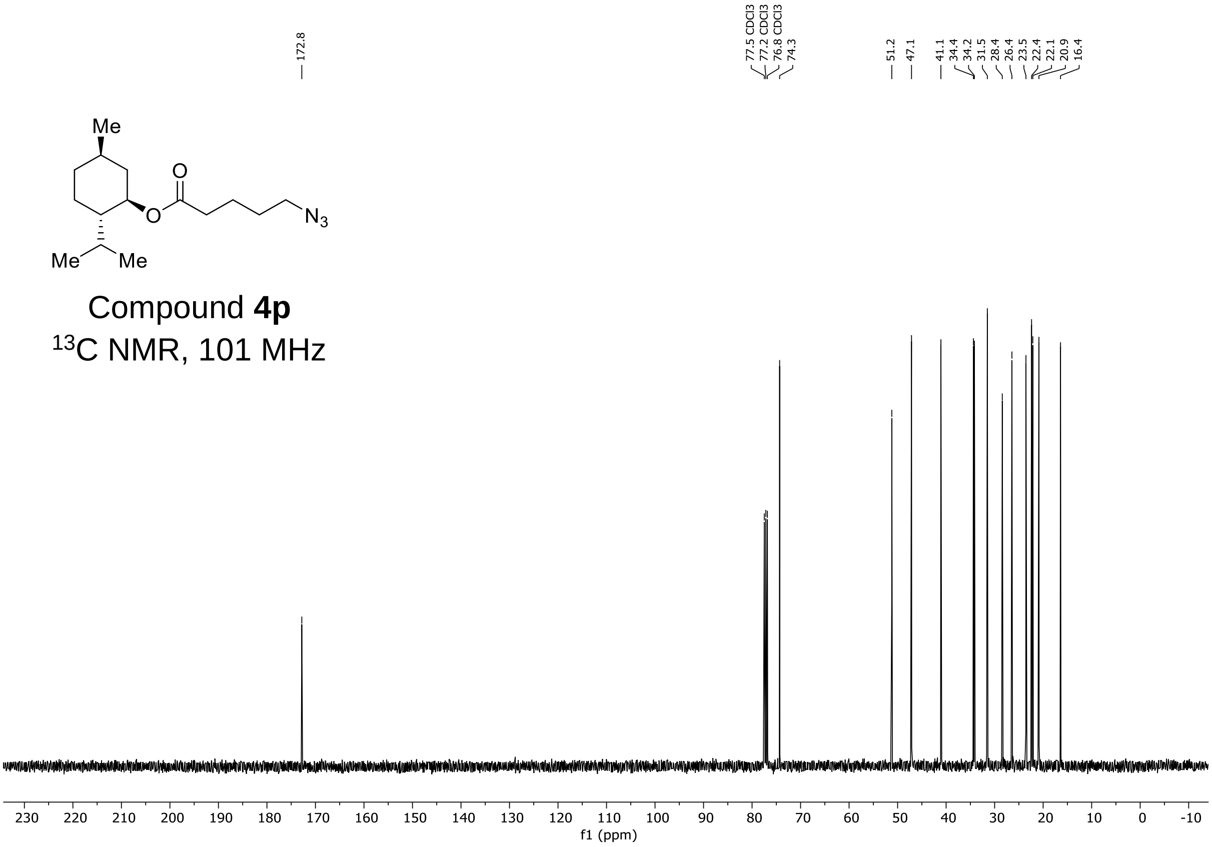

Compound **4p**
^{13}C NMR, 101 MHz

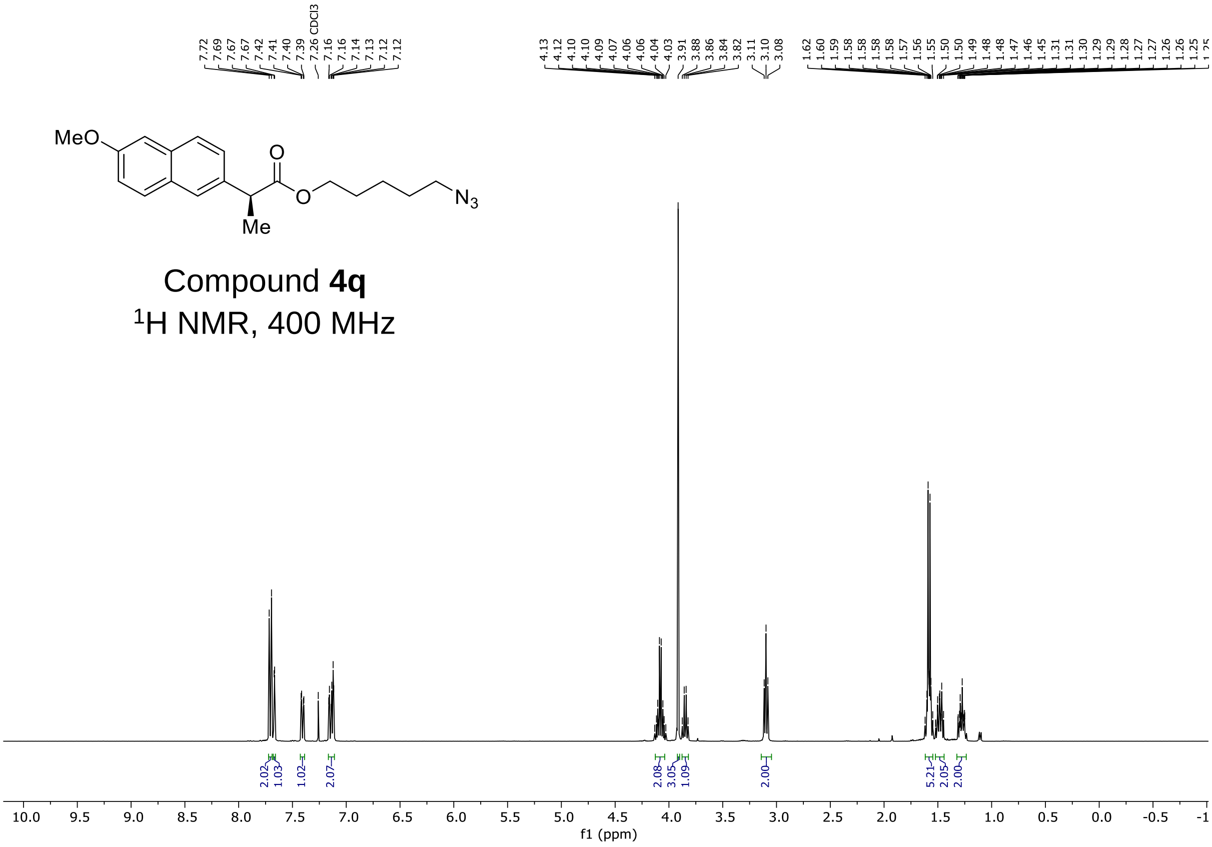

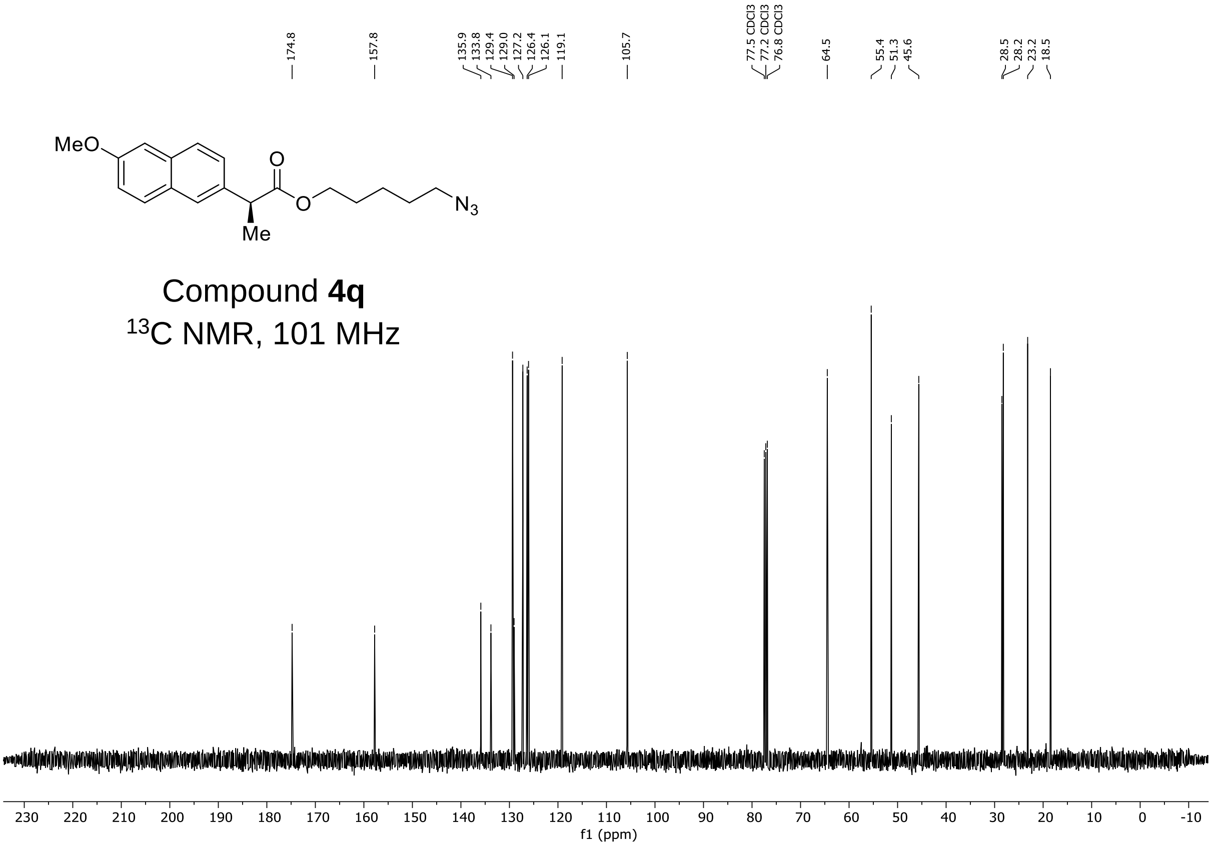

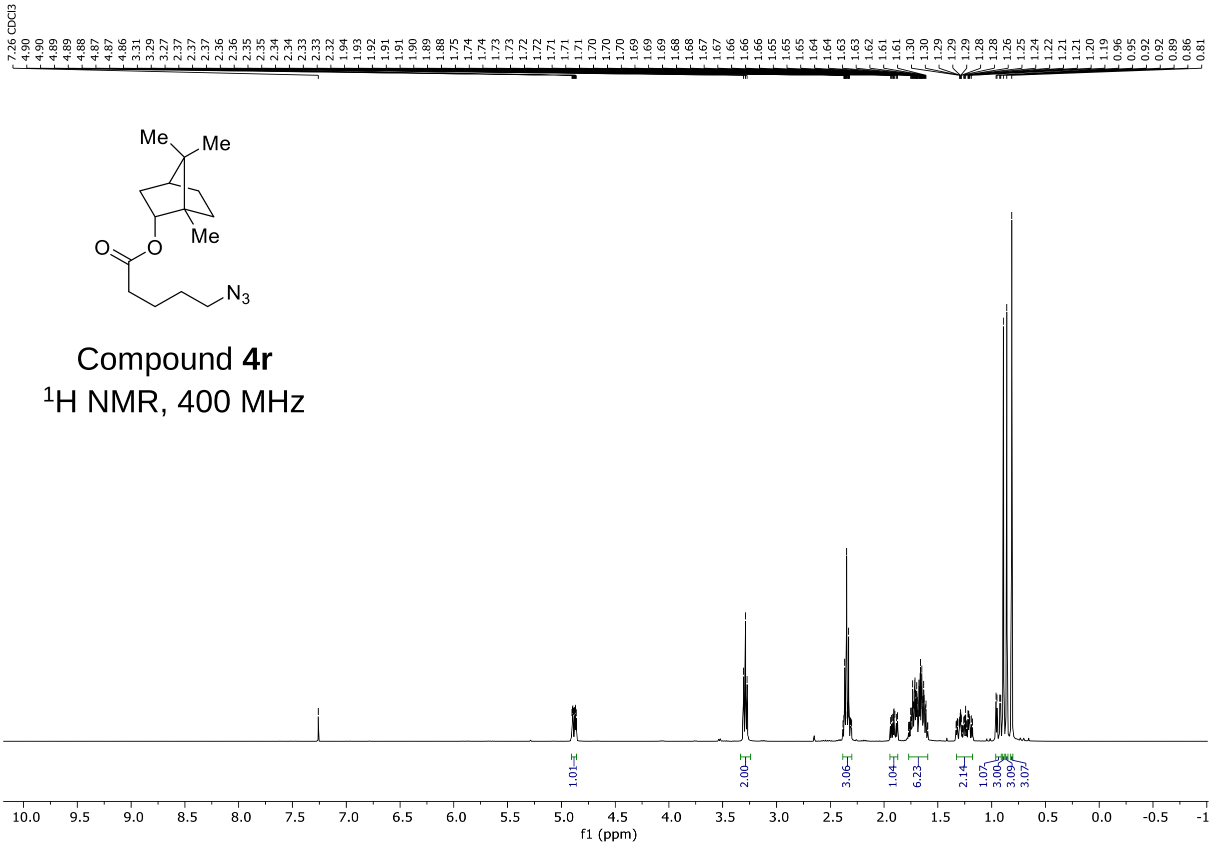
7.26 CDCl3
4.90 4.90 4.89 4.89 4.88 4.87 4.87 4.86 3.31 3.29 3.27 2.37 2.37 2.37 2.36 2.35 2.35 2.34 2.34 2.33 2.33 2.32 1.94 1.93 1.92 1.91 1.91 1.90 1.89 1.88 1.75 1.74 1.74 1.73 1.73 1.72 1.72 1.71 1.71 1.71 1.70 1.70 1.69 1.69 1.69 1.68 1.68 1.67 1.67 1.66 1.66 1.65 1.65 1.65 1.64 1.64 1.63 1.63 1.62 1.61 1.61 1.30 1.30 1.29 1.29 1.29 1.28 1.28 1.26 1.25 1.24 1.22 1.21 1.21 1.20 1.19 0.96 0.95 0.92 0.92 0.89 0.86 0.81
Me
Me
Me
O
O
N3
Compound 4r
1H NMR, 400 MHz
1.01 2.00 3.06 1.04 6.23 2.14 1.07 3.00 3.09 3.07
10.0 9.5 9.0 8.5 8.0 7.5 7.0 6.5 6.0 5.5 5.0 4.5 4.0 3.5 3.0 2.5 2.0 1.5 1.0 0.5 0.0 -0.5 -1
f1 (ppm)

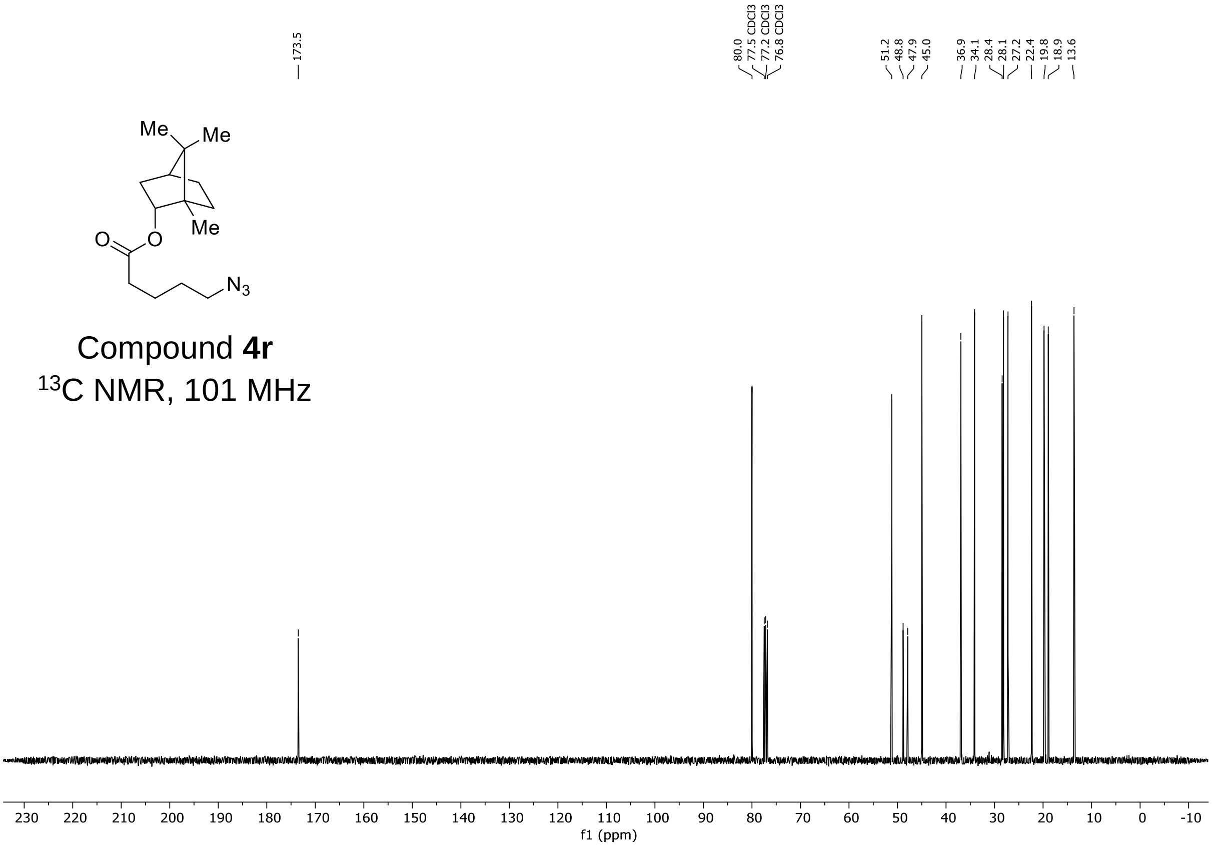
173.5
80.0 77.5 CDCl3 77.2 CDCl3 76.8 CDCl3
51.2 48.8 47.9 45.0
36.9 34.1 28.4 28.1 27.2 22.4 19.8 18.9 13.6
Me
Me
Me
O
O
N3
Compound 4r
13C NMR, 101 MHz
230 220 210 200 190 180 170 160 150 140 130 120 110 100 90 80 70 60 50 40 30 20 10 0 -10
f1 (ppm)

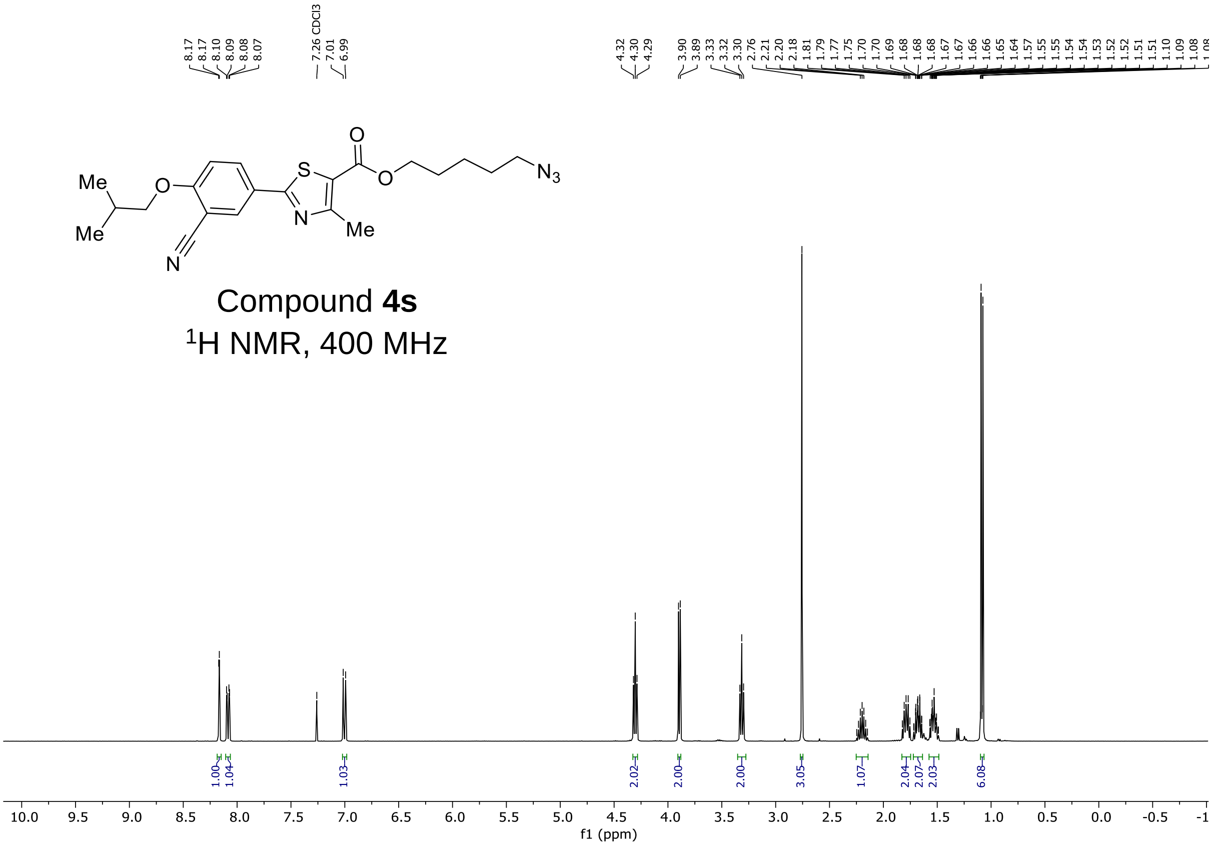
8.17
8.17
8.10
8.09
8.08
8.07
7.26 CDCl3
7.01
6.99
4.32
4.30
4.29
3.90
3.89
3.33
3.32
3.30
2.76
2.21
2.20
2.18
1.81
1.79
1.77
1.75
1.70
1.70
1.69
1.68
1.68
1.67
1.67
1.66
1.65
1.64
1.57
1.55
1.55
1.54
1.54
1.53
1.52
1.52
1.51
1.51
1.10
1.09
1.08
Me
O
Me
N
S
N
Me
O
O
N3
Compound 4s
1H NMR, 400 MHz
1.00
1.04
1.03
2.02
2.00
2.00
3.05
1.07
2.04
2.07
2.03
6.08
f1 (ppm)

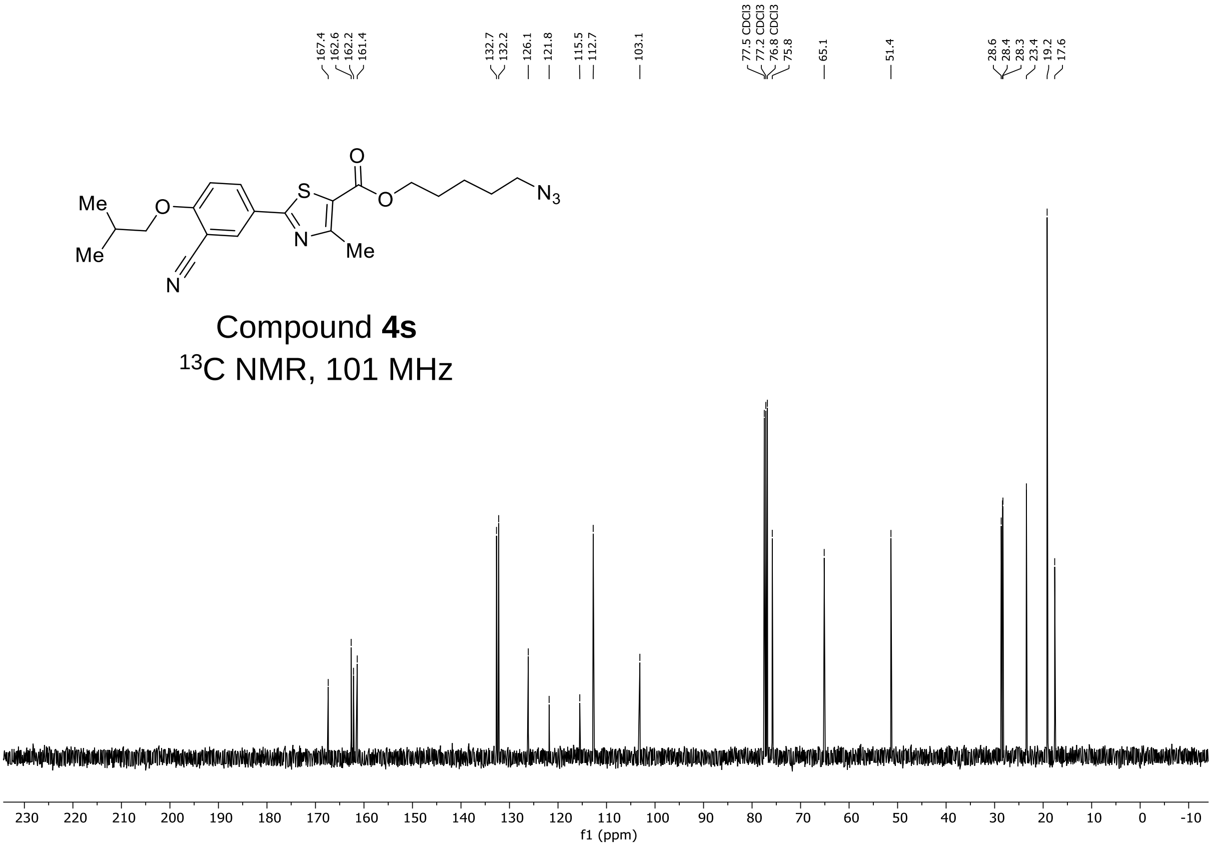
167.4
162.6
162.2
161.4
132.7
132.2
126.1
121.8
115.5
112.7
103.1
77.5 CDCl3
77.2 CDCl3
76.8 CDCl3
75.8
65.1
51.4
28.6
28.4
28.3
23.4
19.2
17.6
Me
O
Me
N
S
N
Me
O
O
N3
Compound 4s
13C NMR, 101 MHz
f1 (ppm)

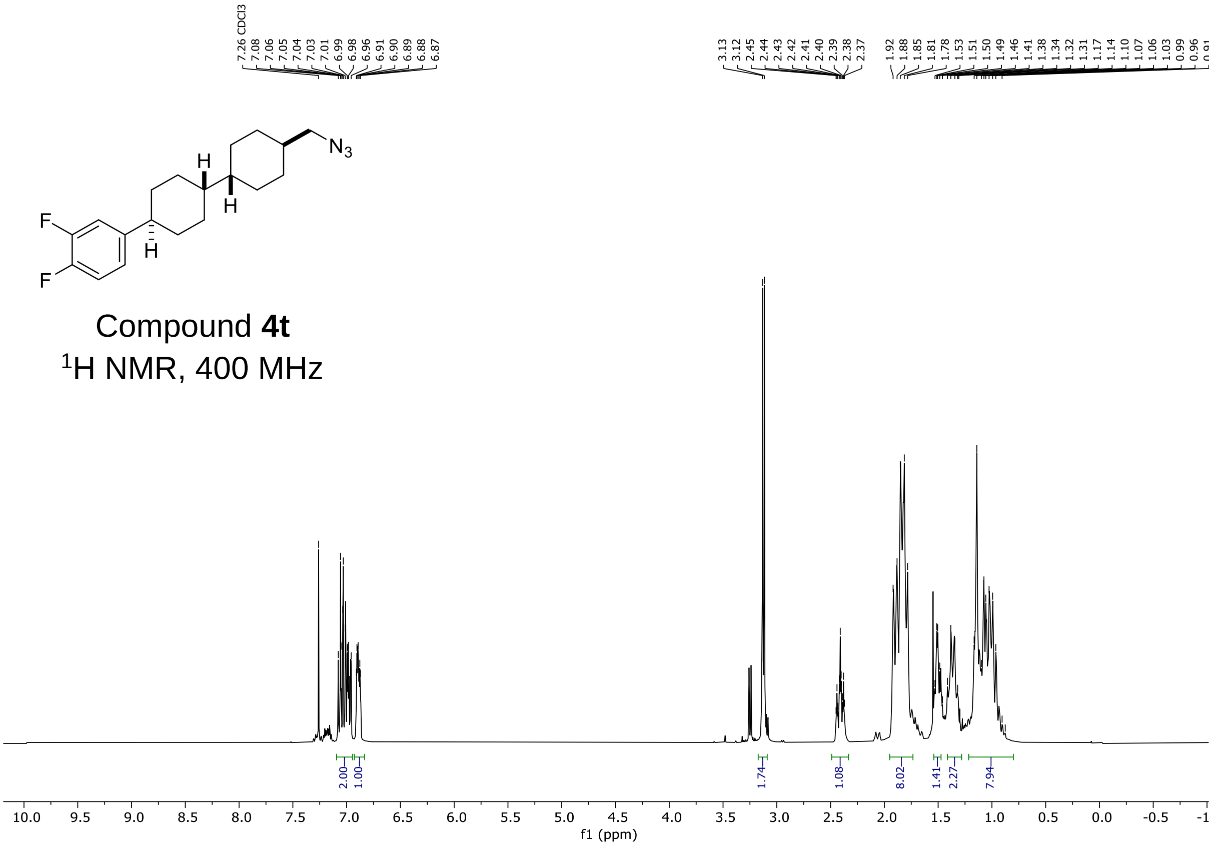

Compound **4t**
^{1}H NMR, 400 MHz

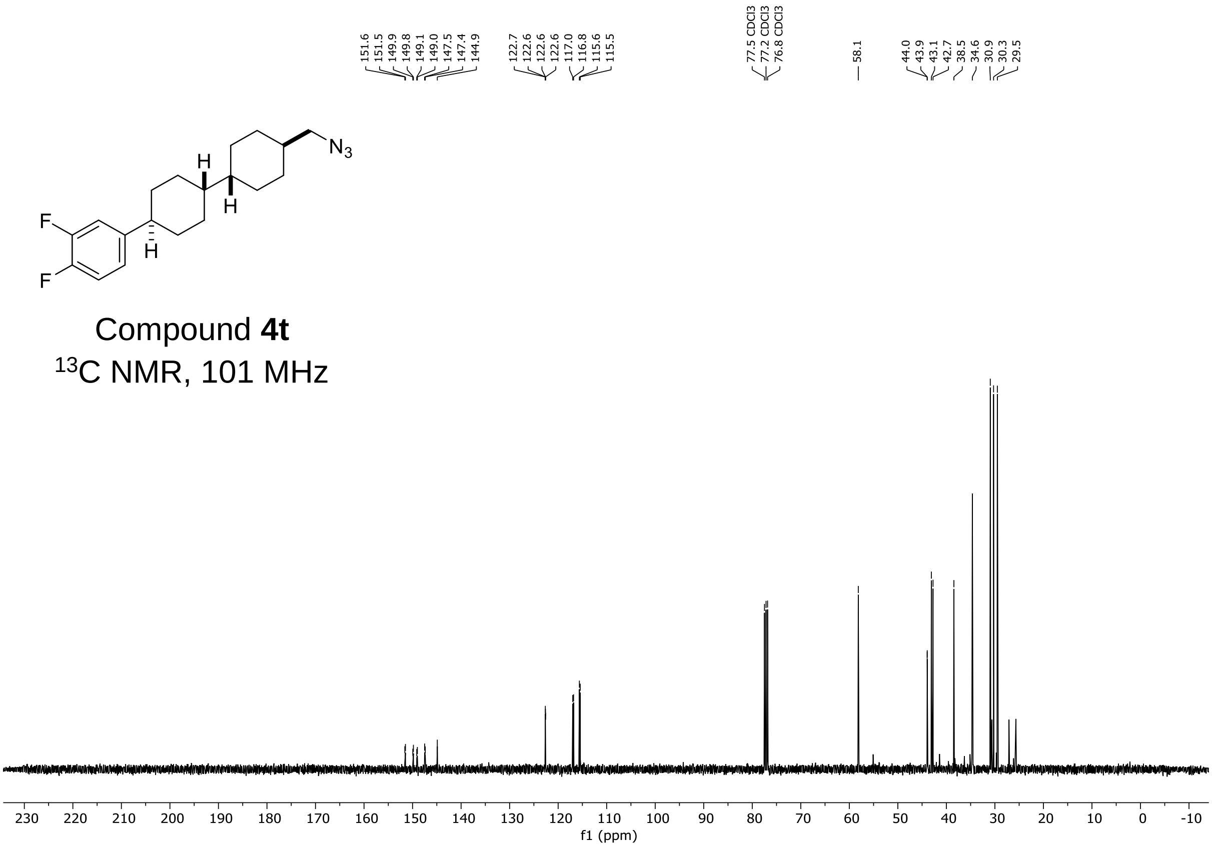

Compound **4t**
^{13}C NMR, 101 MHz

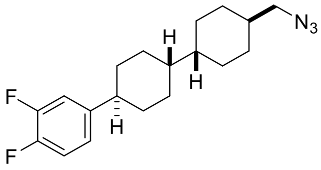

Compound **4t**

^{19}F NMR, 377 MHz

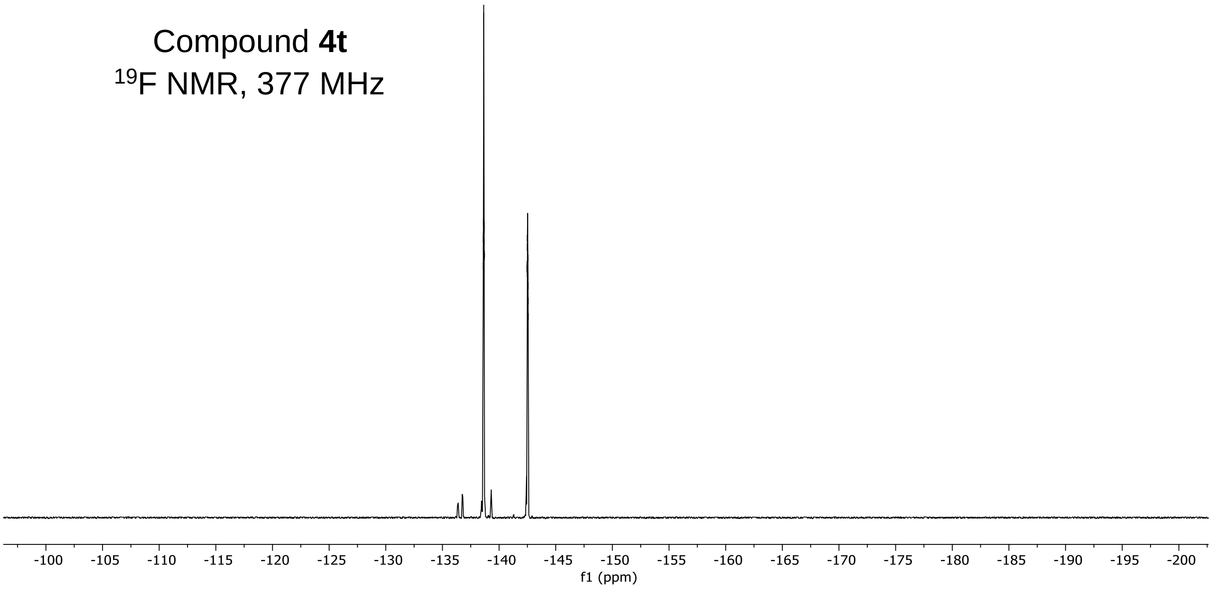

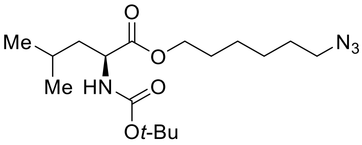

Compound **4u**
^{1}H NMR, 400 MHz

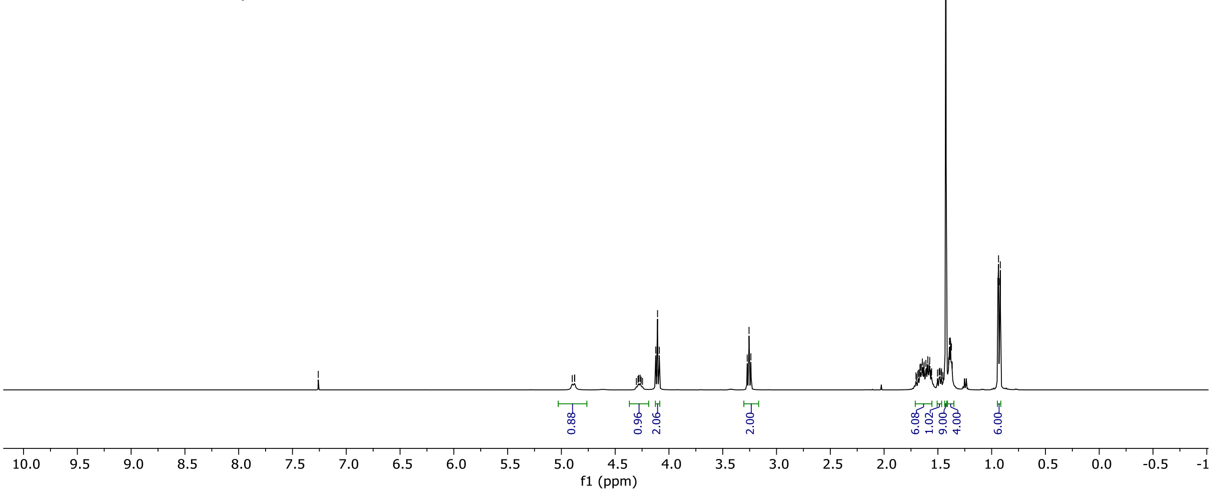

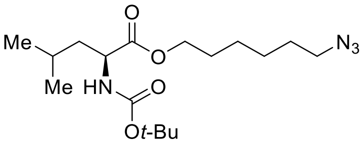

Compound **4u**
^{13}C NMR, 101 MHz

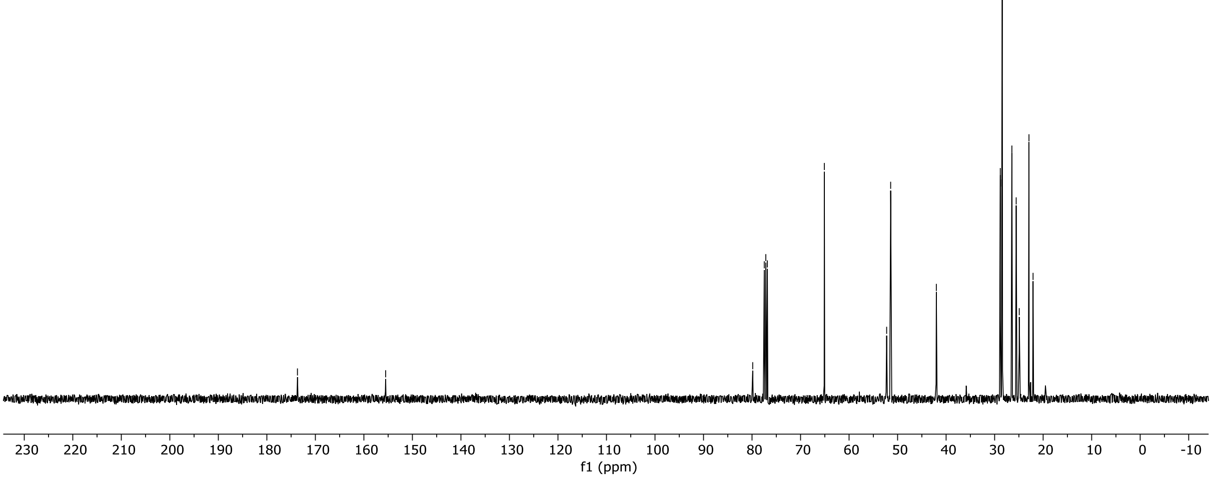

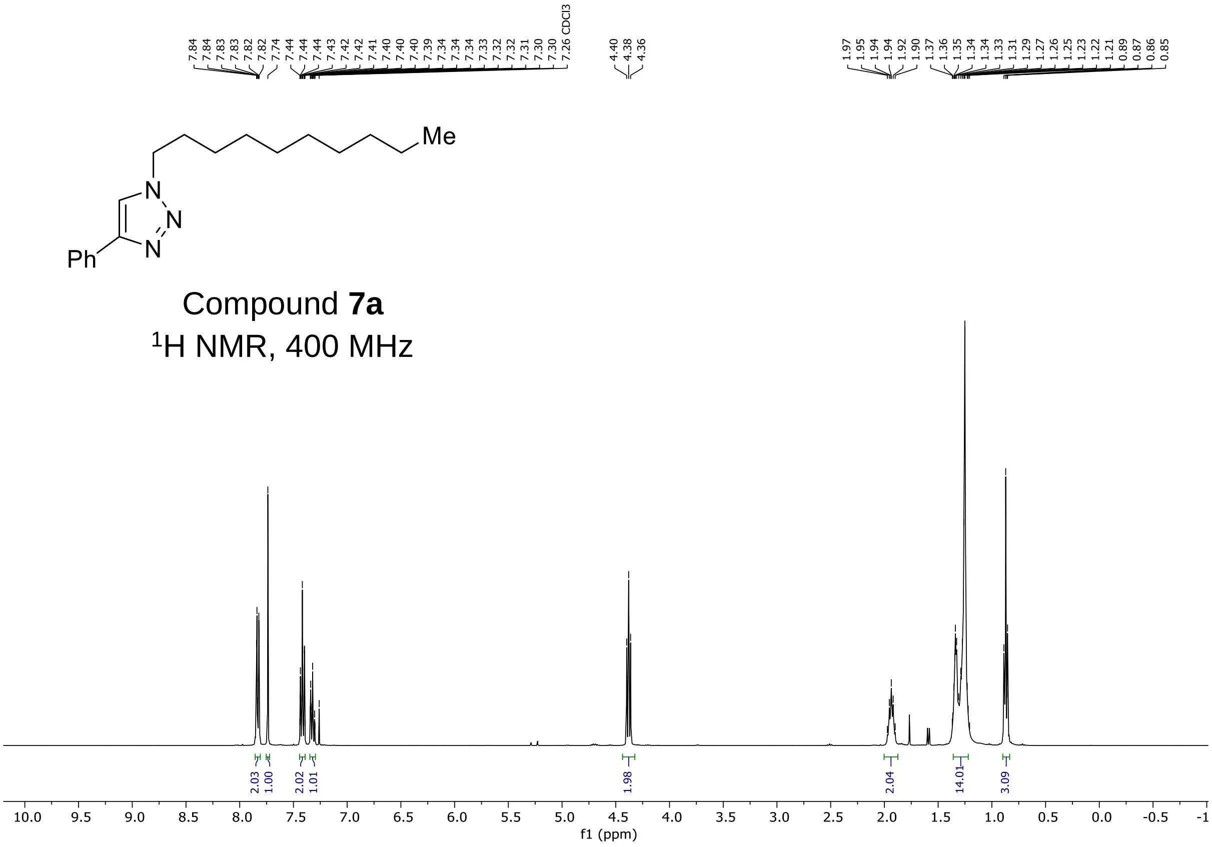
7.84 7.84 7.83 7.83 7.82 7.82 7.74 7.44 7.44 7.44 7.43 7.42 7.42 7.41 7.40 7.40 7.39 7.34 7.34 7.34 7.33 7.32 7.32 7.31 7.30 7.26 CDCl3
4.40 4.38 4.36
1.97 1.95 1.94 1.94 1.92 1.90 1.37 1.36 1.35 1.34 1.34 1.33 1.31 1.29 1.27 1.26 1.25 1.23 1.22 1.21 0.89 0.87 0.86 0.85
Me
N
N
N
Ph
Compound **7a**
^{1}H NMR, 400 MHz
2.03 1.00 2.02 1.01 1.98 2.04 14.01 3.09
10.0 9.5 9.0 8.5 8.0 7.5 7.0 6.5 6.0 5.5 5.0 4.5 4.0 3.5 3.0 2.5 2.0 1.5 1.0 0.5 0.0 -0.5 -1
f1 (ppm)

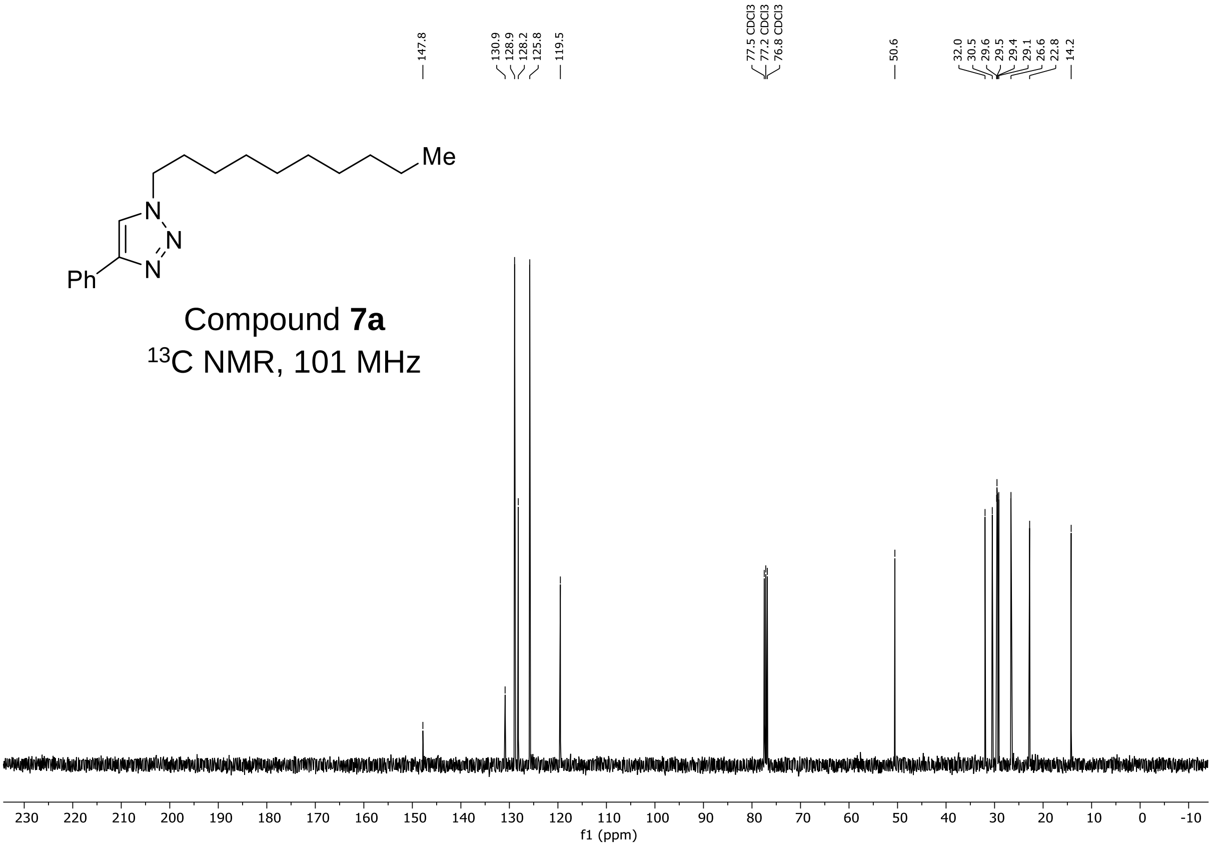
147.8
130.9 128.9 128.2 125.8 119.5
77.5 CDCl3 77.2 CDCl3 76.8 CDCl3
50.6
32.0 30.5 29.6 29.5 29.4 29.1 26.6 22.8 14.2
Me
N
N
N
Ph
Compound **7a**
^{13}C NMR, 101 MHz
230 220 210 200 190 180 170 160 150 140 130 120 110 100 90 80 70 60 50 40 30 20 10 0 -10
f1 (ppm)

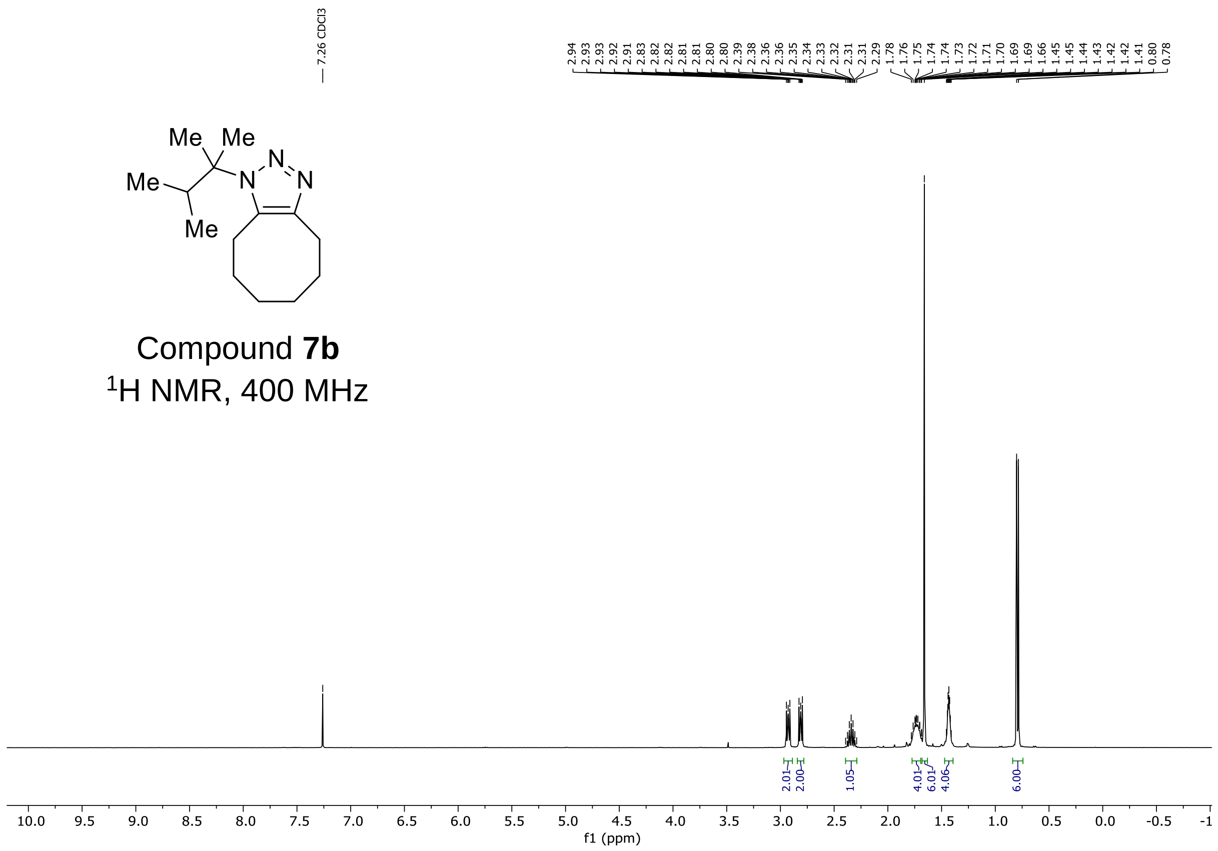
7.26 CDCl3
2.94
2.93
2.93
2.92
2.91
2.83
2.82
2.82
2.81
2.81
2.80
2.80
2.39
2.38
2.36
2.36
2.35
2.34
2.33
2.32
2.31
2.31
2.29
1.78
1.76
1.75
1.75
1.74
1.74
1.73
1.72
1.71
1.70
1.69
1.69
1.66
1.45
1.45
1.44
1.43
1.42
1.42
1.41
0.80
0.78
Me
Me
N
N
N
Me
Me
Compound 7b
1H NMR, 400 MHz
2.01
2.00
1.05
4.01
6.01
4.06
6.00
10.0 9.5 9.0 8.5 8.0 7.5 7.0 6.5 6.0 5.5 5.0 4.5 4.0 3.5 3.0 2.5 2.0 1.5 1.0 0.5 0.0 -0.5 -1
f1 (ppm)

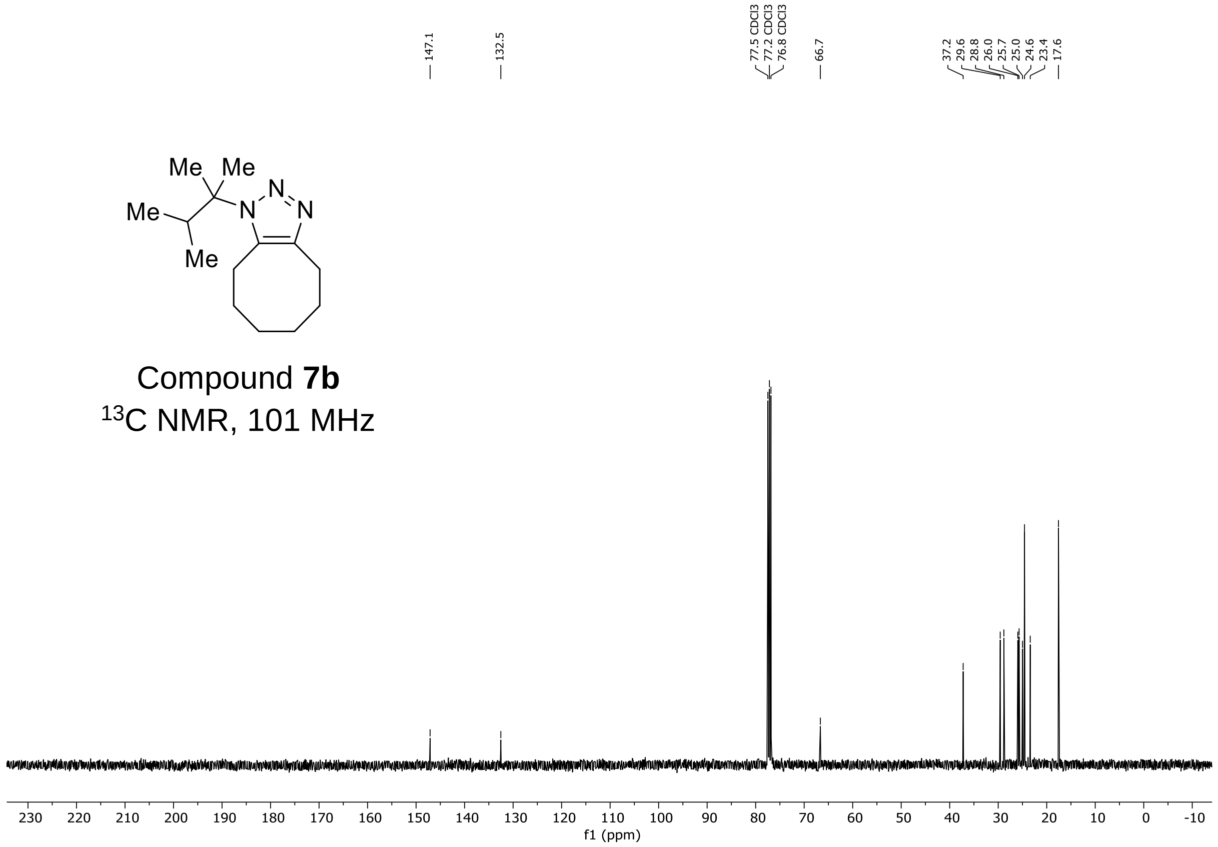
147.1
132.5
77.5 CDCl3
77.2 CDCl3
76.8 CDCl3
66.7
37.2
29.6
28.8
26.0
25.7
25.0
24.6
23.4
17.6
Me
Me
N
N
N
Me
Me
Compound 7b
13C NMR, 101 MHz
230 220 210 200 190 180 170 160 150 140 130 120 110 100 90 80 70 60 50 40 30 20 10 0 -10
f1 (ppm)

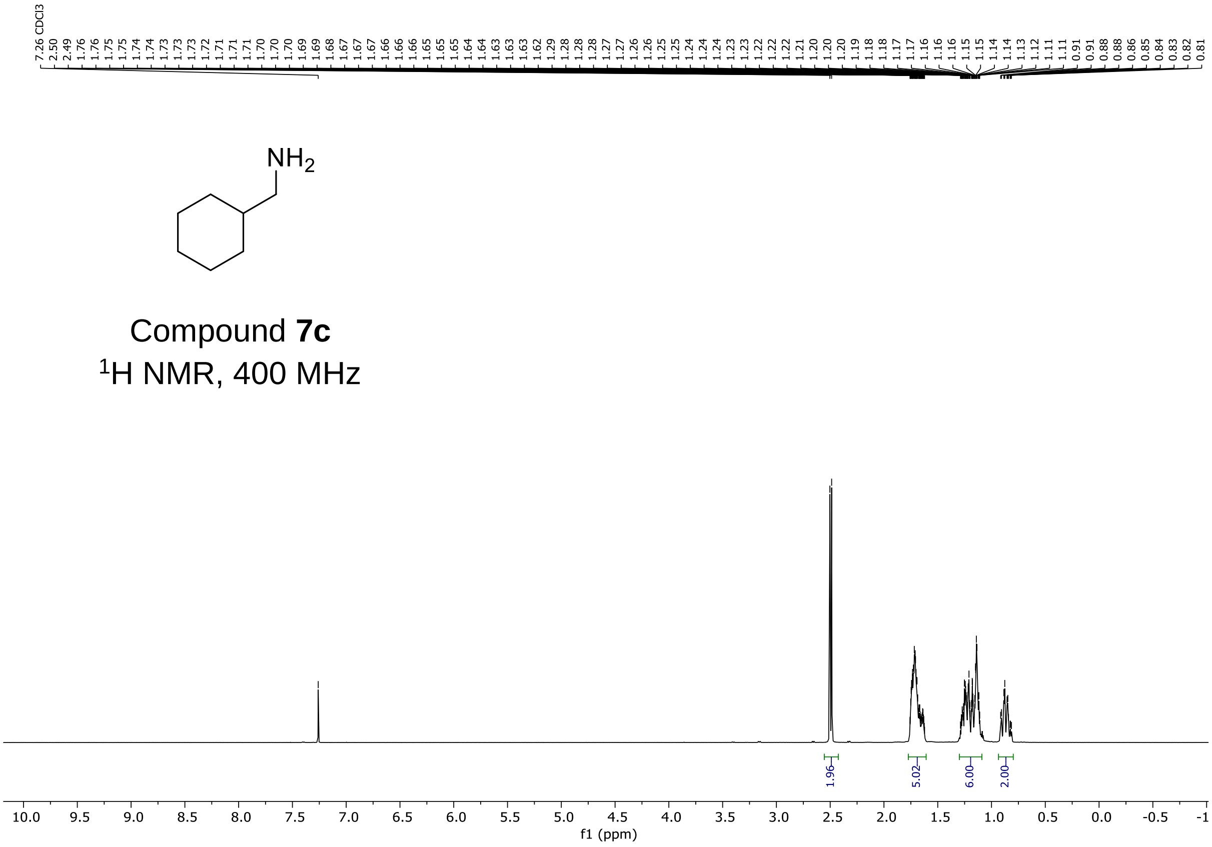

Compound **7c**
^{1}H NMR, 400 MHz

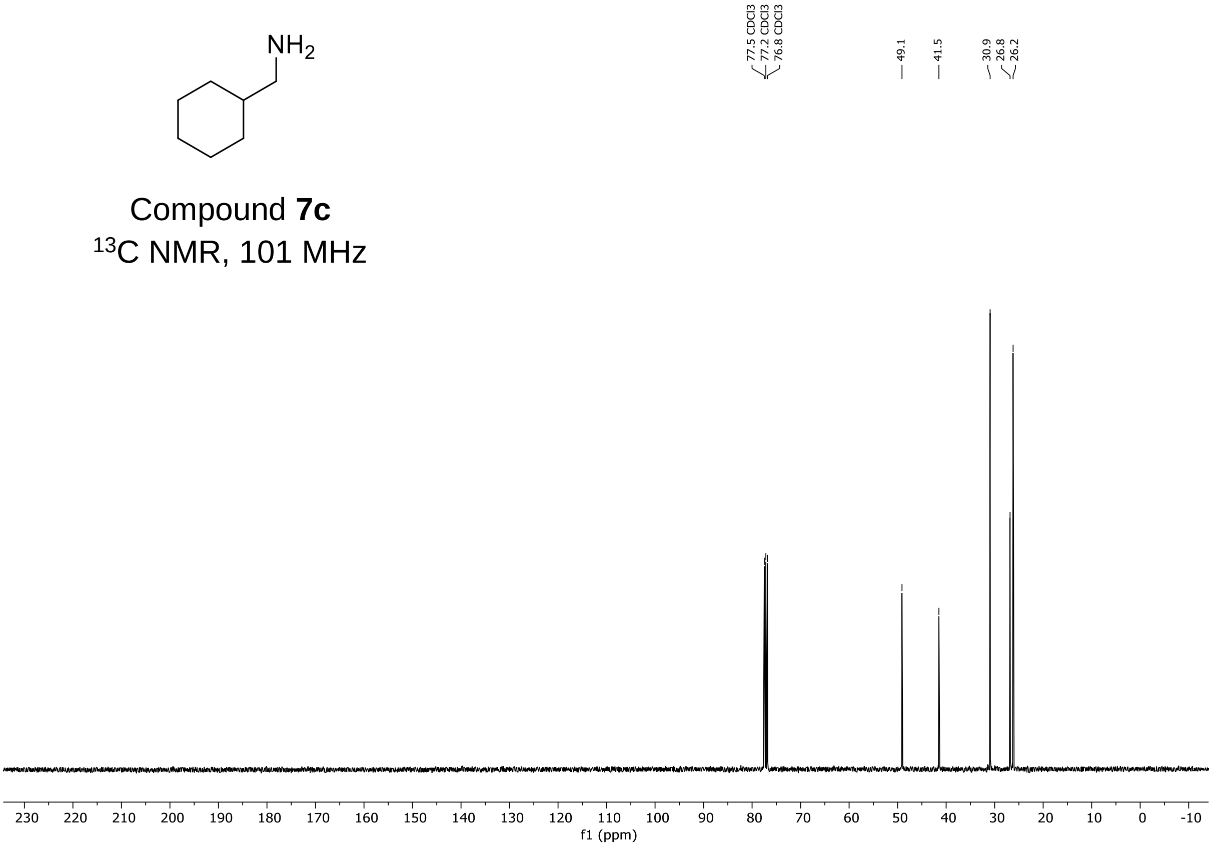

Compound **7c**
^{13}C NMR, 101 MHz

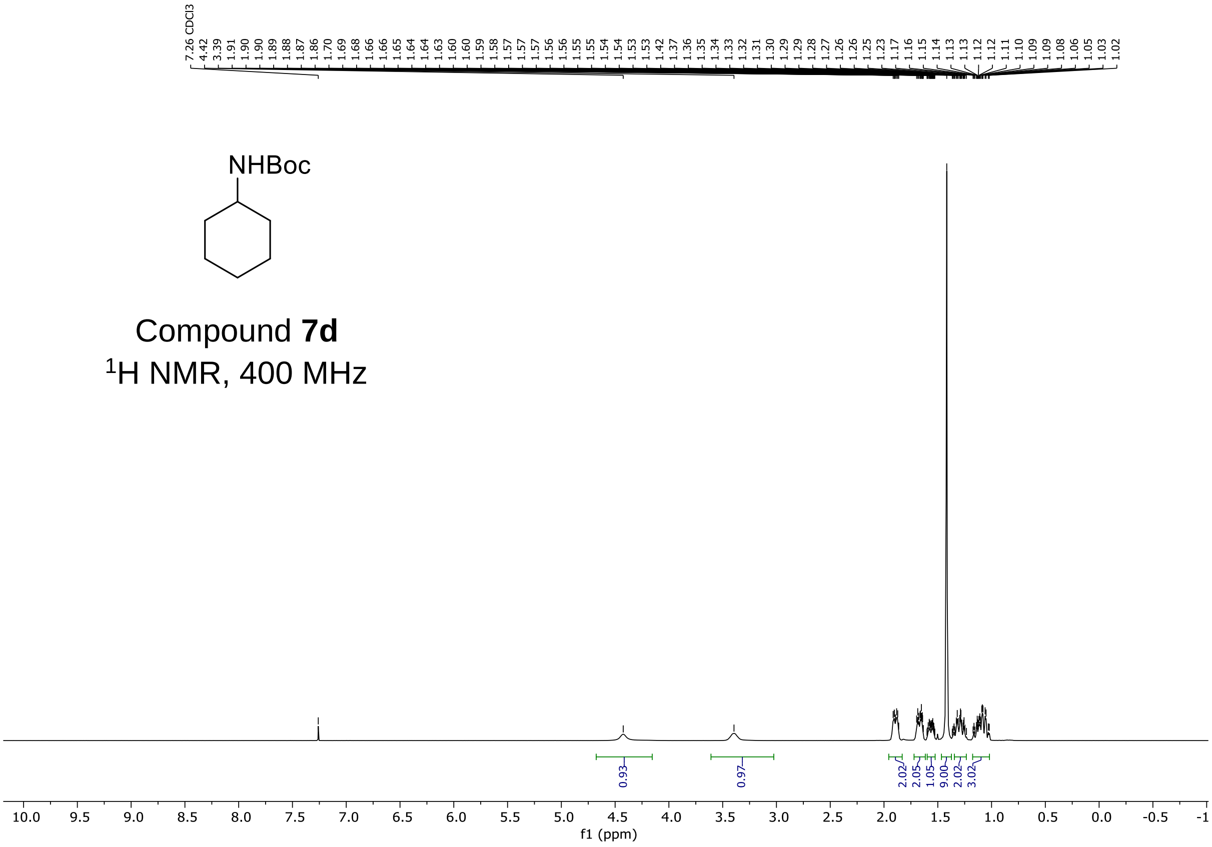
7.26 CDCl3
4.42
3.39
1.91 1.90 1.90 1.89 1.88 1.87 1.86 1.70 1.69 1.68 1.66 1.66 1.65 1.64 1.64 1.63 1.60 1.60 1.59 1.58 1.57 1.57 1.57 1.56 1.56 1.55 1.55 1.54 1.54 1.53 1.53 1.42 1.37 1.36 1.35 1.34 1.33 1.32 1.31 1.30 1.29 1.29 1.28 1.27 1.26 1.26 1.25 1.23 1.17 1.16 1.15 1.14 1.13 1.13 1.12 1.12 1.11 1.10 1.09 1.09 1.08 1.06 1.05 1.03 1.02
NHBoc
Compound **7d**
^{1}H NMR, 400 MHz
0.93
0.97
2.02
2.05
1.05
9.00
2.02
3.02
10.0 9.5 9.0 8.5 8.0 7.5 7.0 6.5 6.0 5.5 5.0 4.5 4.0 3.5 3.0 2.5 2.0 1.5 1.0 0.5 0.0 -0.5 -1
f1 (ppm)

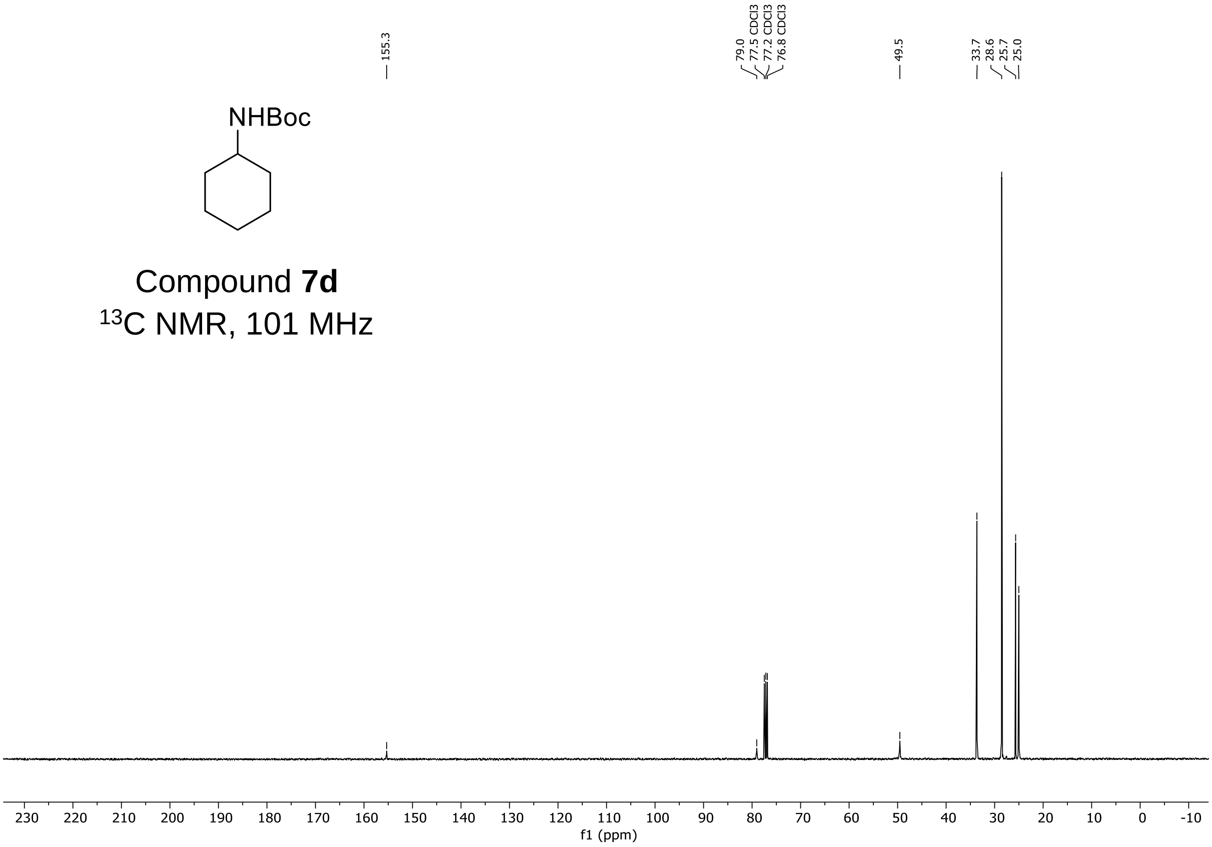
155.3
79.0
77.5 CDCl3
77.2 CDCl3
76.8 CDCl3
49.5
33.7
28.6
25.7
25.0
NHBoc
Compound **7d**
^{13}C NMR, 101 MHz
230 220 210 200 190 180 170 160 150 140 130 120 110 100 90 80 70 60 50 40 30 20 10 0 -10
f1 (ppm)

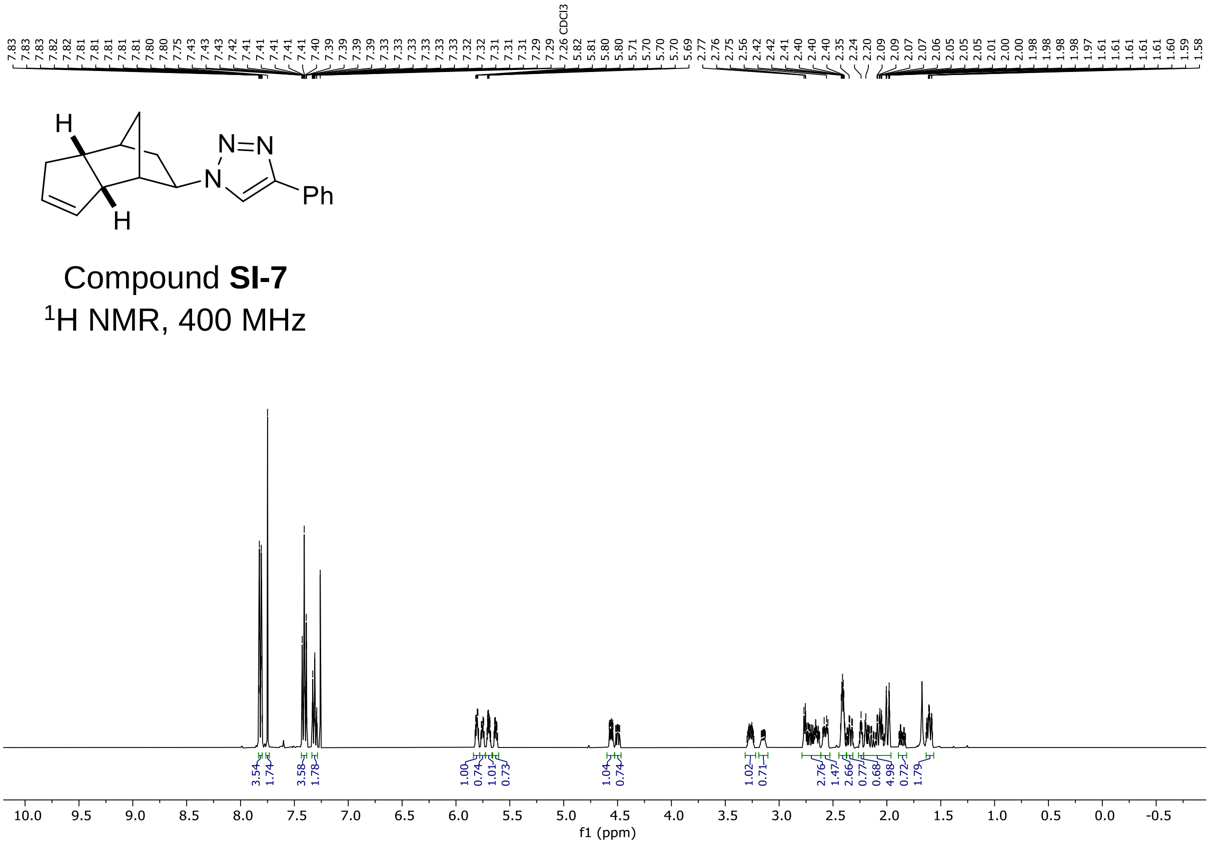

Compound **SI-7**

^{1}H NMR, 400 MHz

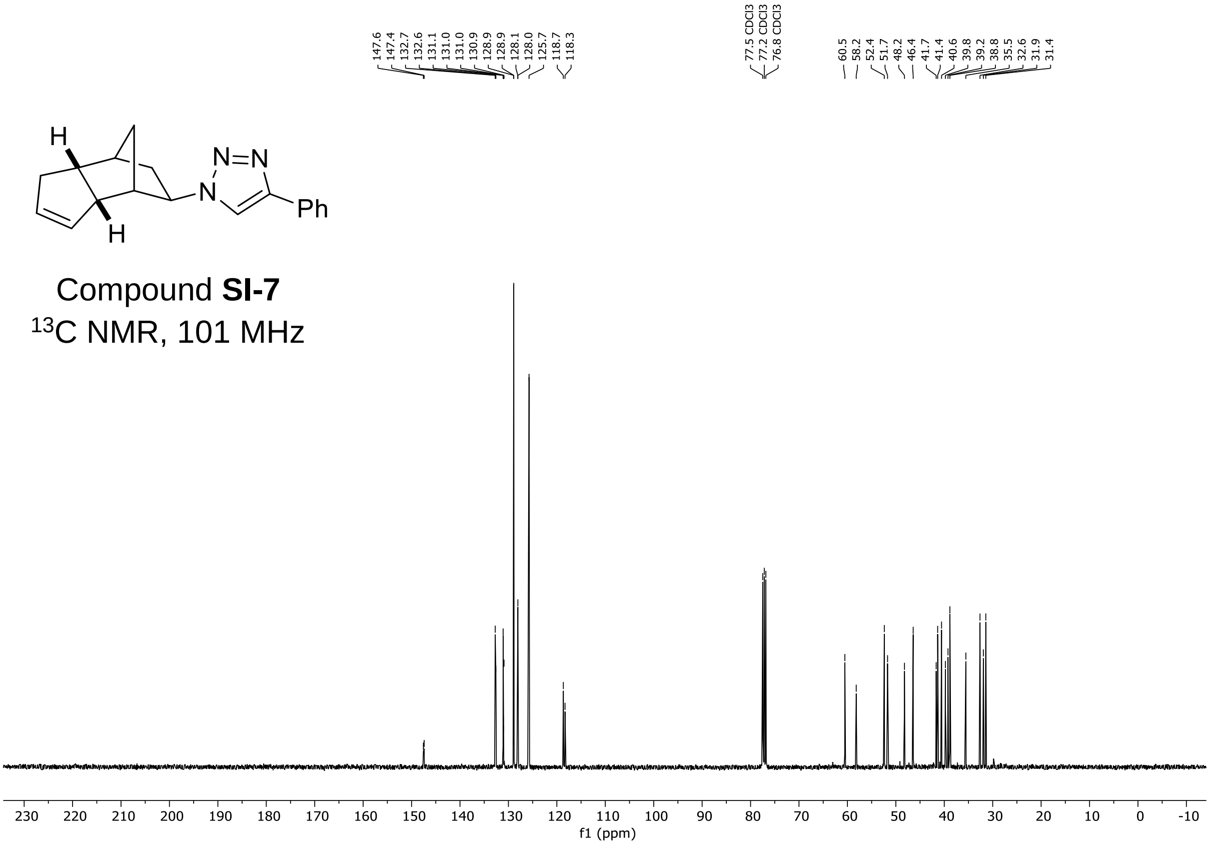

Compound **SI-7**

^{13}C NMR, 101 MHz

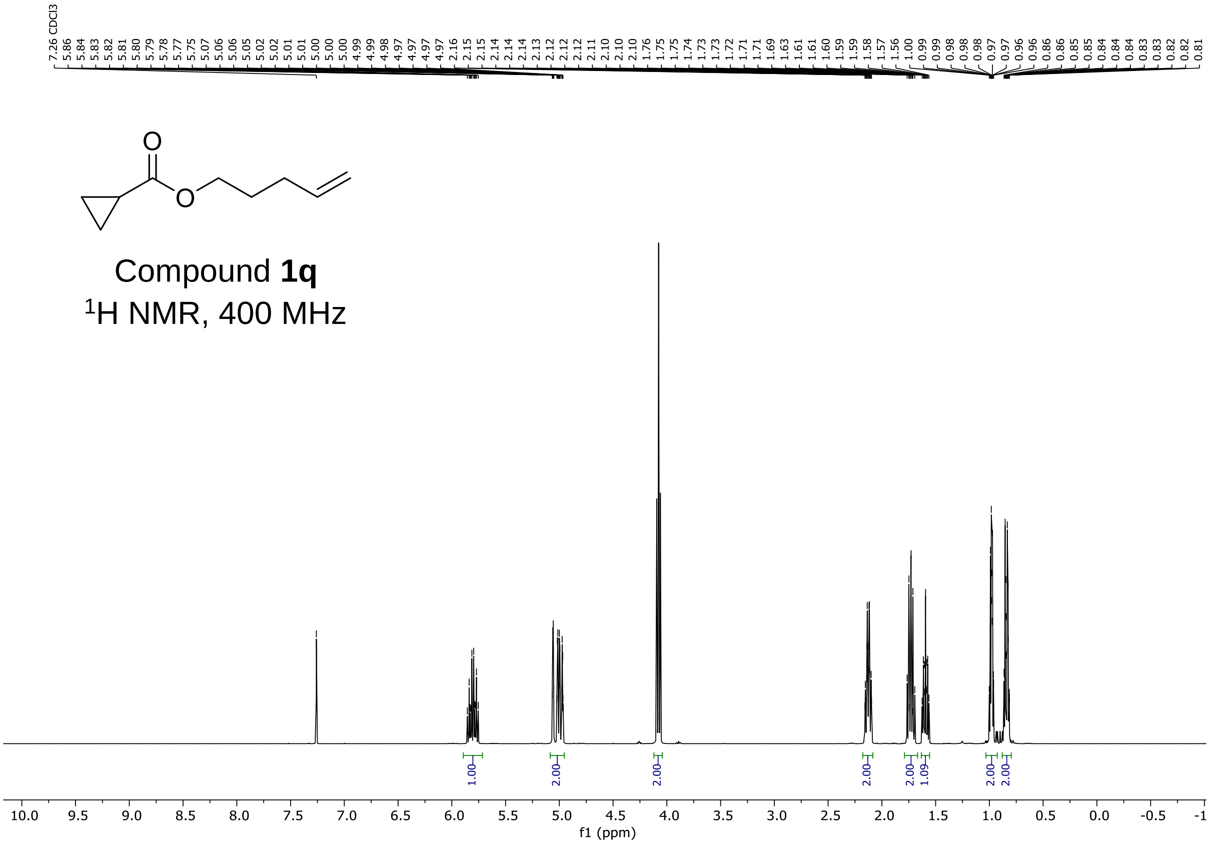

7.26 CDCl3
5.86
5.84
5.83
5.82
5.81
5.80
5.79
5.78
5.77
5.75
5.07
5.06
5.06
5.05
5.02
5.02
5.01
5.01
5.00
5.00
5.00
4.99
4.99
4.98
4.97
4.97
4.97
4.97
2.16
2.15
2.15
2.14
2.14
2.14
2.13
2.12
2.12
2.12
2.11
2.10
2.10
2.10
1.76
1.75
1.75
1.74
1.73
1.73
1.72
1.71
1.71
1.69
1.63
1.61
1.61
1.60
1.59
1.59
1.58
1.57
1.56
1.00
0.99
0.99
0.98
0.98
0.98
0.97
0.97
0.96
0.96
0.86
0.86
0.85
0.85
0.84
0.84
0.84
0.83
0.83
0.82
0.82
0.81
O
O
Compound **1q**
^{1}H NMR, 400 MHz
1.00
2.00
2.00
2.00
2.00
1.09
2.00
2.00
10.0 9.5 9.0 8.5 8.0 7.5 7.0 6.5 6.0 5.5 5.0 4.5 4.0 3.5 3.0 2.5 2.0 1.5 1.0 0.5 0.0 -0.5 -1
f1 (ppm)

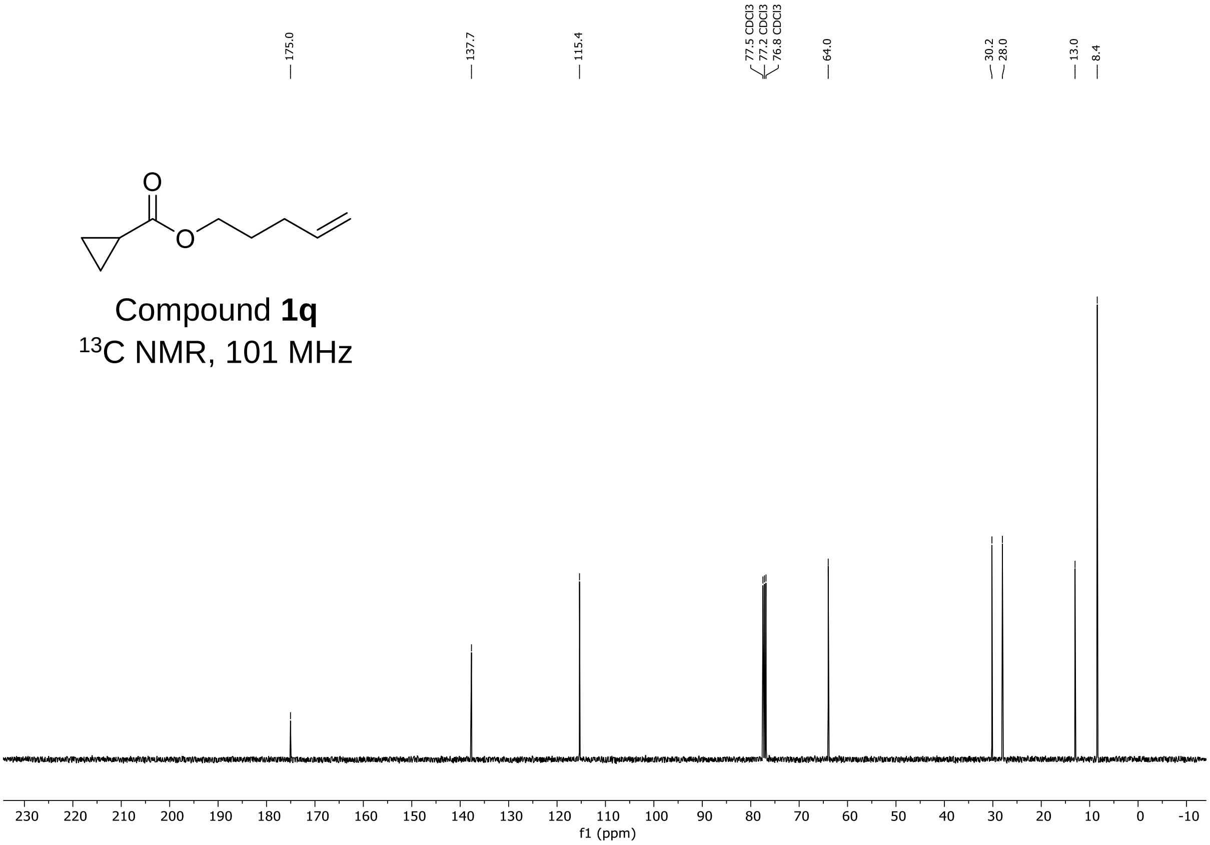

175.0
137.7
115.4
77.5 CDCl3
77.2 CDCl3
76.8 CDCl3
64.0
30.2
28.0
13.0
8.4
O
O
Compound **1q**
^{13}C NMR, 101 MHz
230 220 210 200 190 180 170 160 150 140 130 120 110 100 90 80 70 60 50 40 30 20 10 0 -10
f1 (ppm)

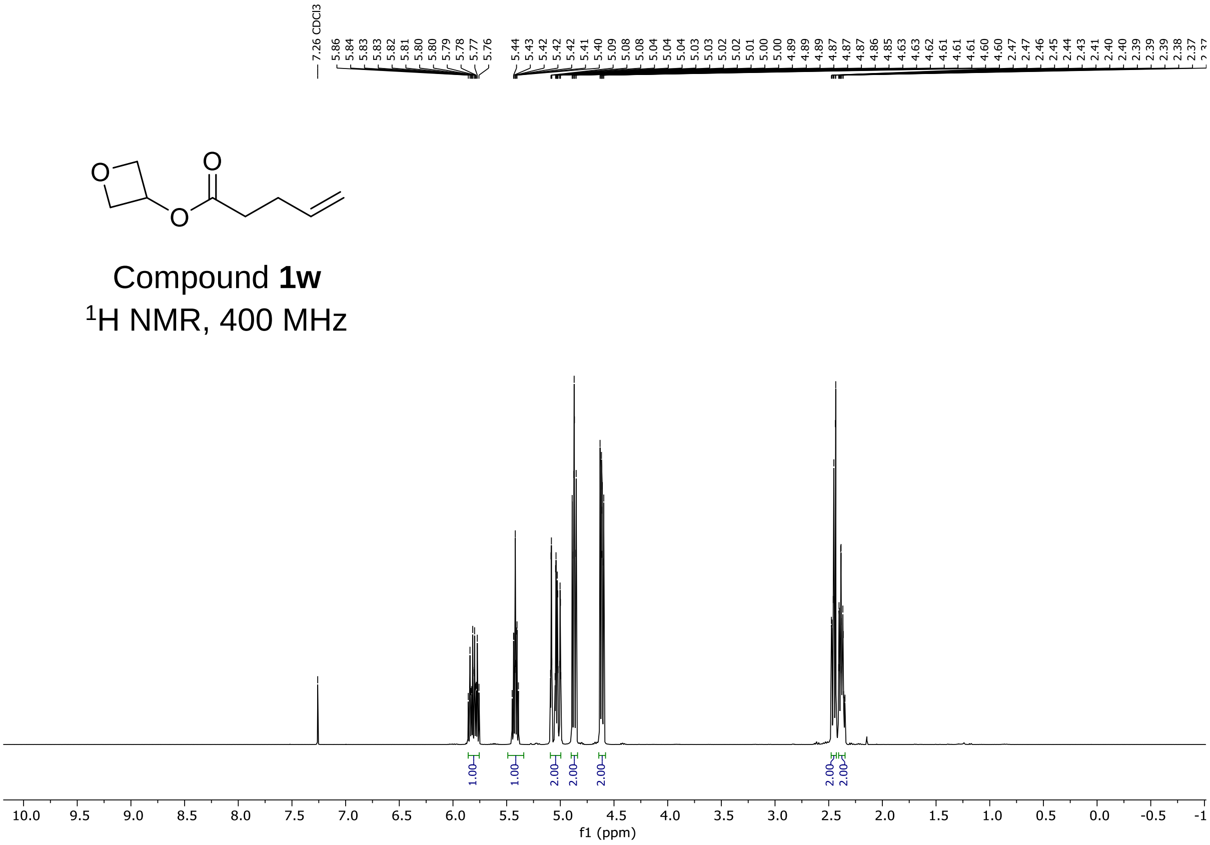

Compound **1w**
^{1}H NMR, 400 MHz

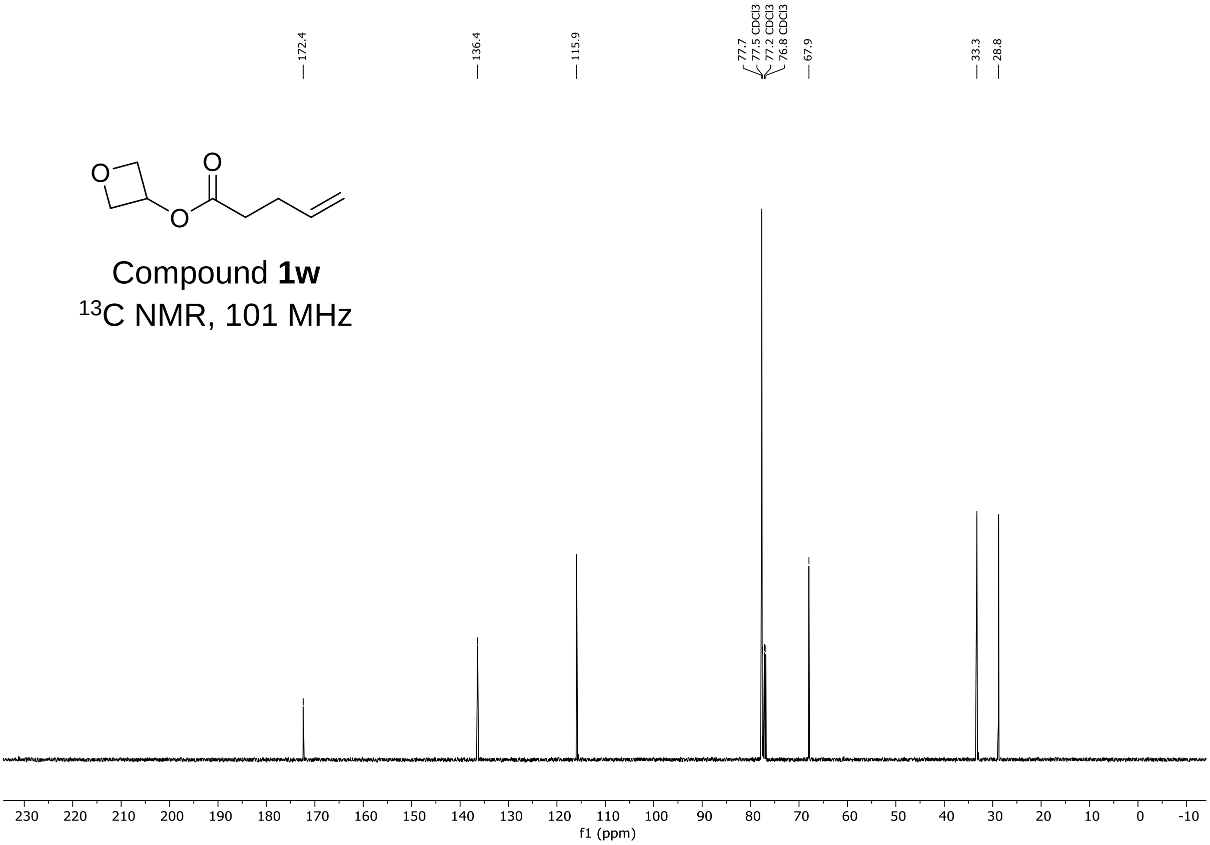

Compound **1w**
^{13}C NMR, 101 MHz

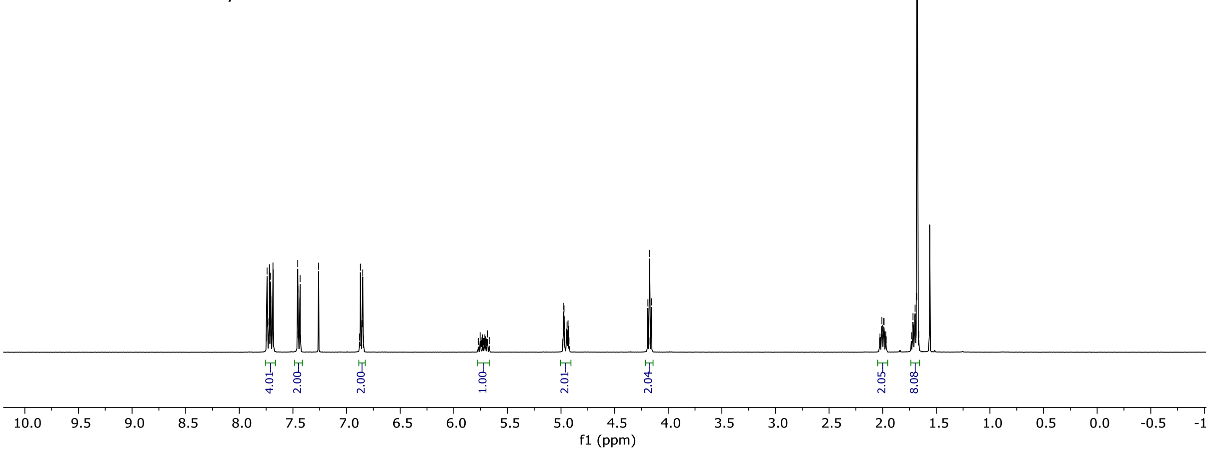

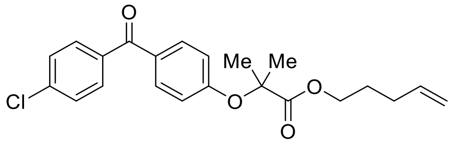

Compound **3a**
^{1}H NMR, 400 MHz

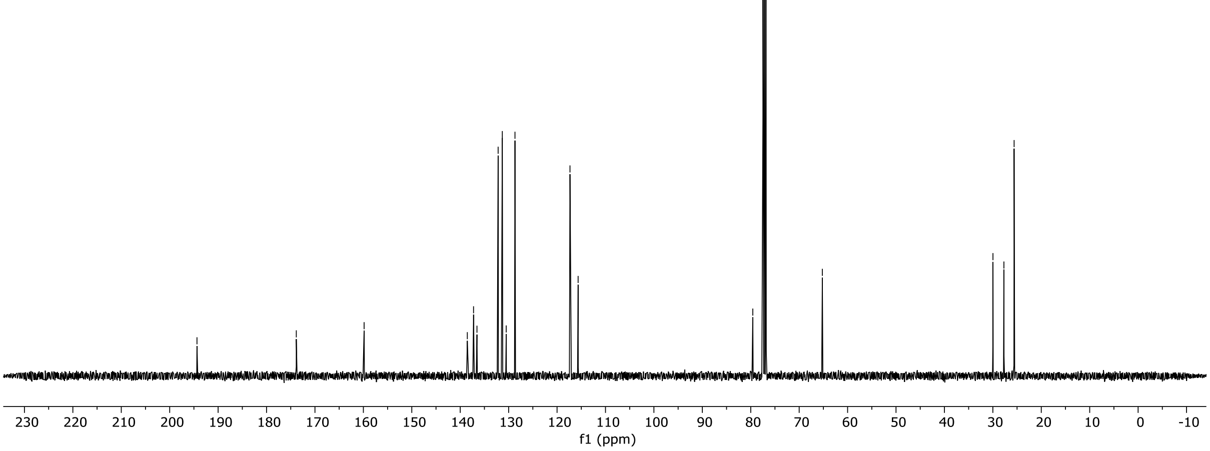

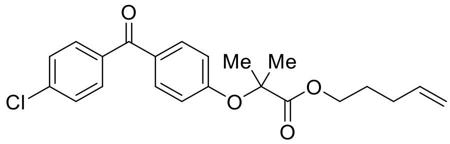

Compound **3a**
^{13}C NMR, 101 MHz

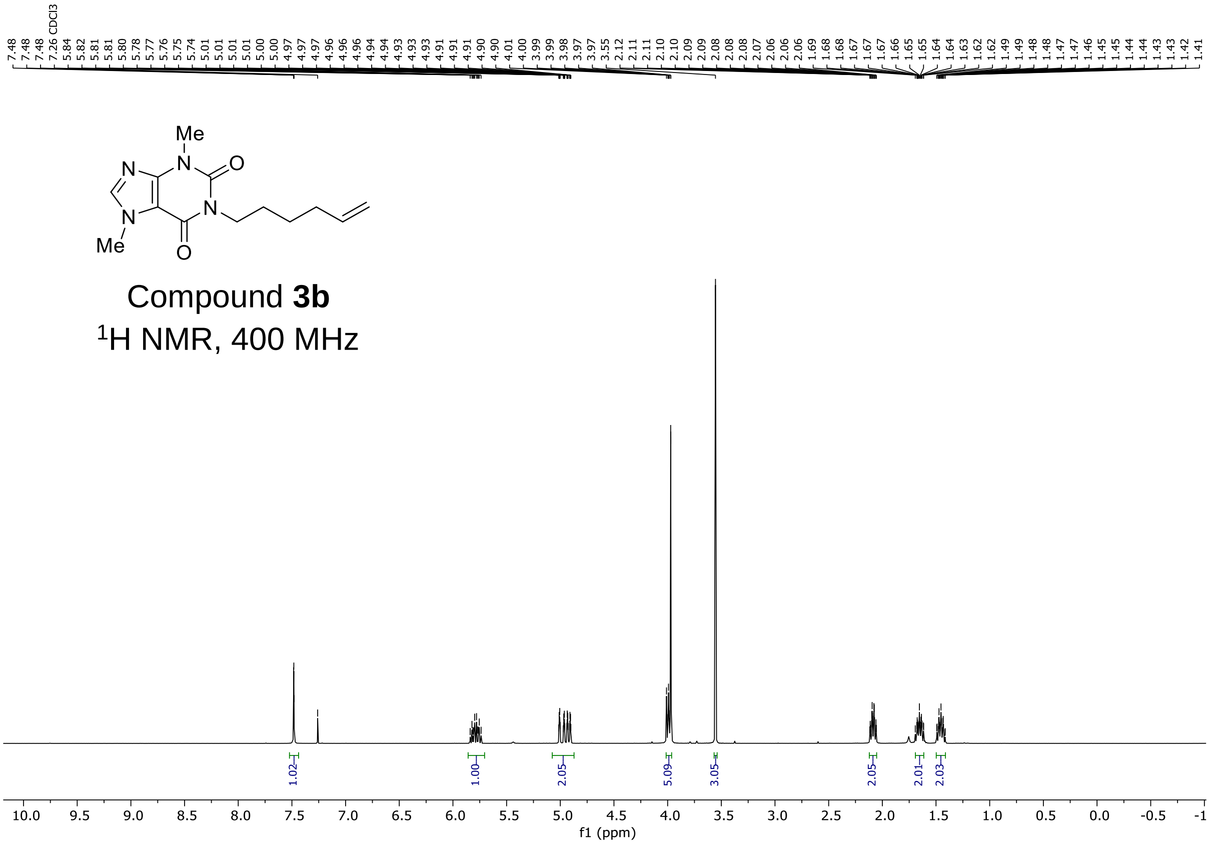
7.48
7.48
7.48
7.26 CDCl3
5.84
5.82
5.81
5.81
5.80
5.78
5.77
5.76
5.75
5.74
5.01
5.01
5.01
5.01
5.00
5.00
4.97
4.97
4.97
4.96
4.96
4.96
4.94
4.94
4.93
4.93
4.93
4.91
4.91
4.91
4.90
4.90
4.01
4.00
3.99
3.99
3.98
3.97
3.97
3.55
2.12
2.11
2.11
2.10
2.10
2.09
2.09
2.08
2.08
2.08
2.07
2.06
2.06
2.06
1.69
1.68
1.67
1.67
1.67
1.66
1.65
1.65
1.64
1.64
1.63
1.62
1.62
1.49
1.49
1.48
1.48
1.47
1.47
1.46
1.45
1.45
1.44
1.44
1.43
1.43
1.42
1.41
Me
N
O
N
N
N
Me
O
Compound 3b
1H NMR, 400 MHz
1.02
1.00
2.05
5.09
3.05
2.05
2.01
2.03
10.0
9.5
9.0
8.5
8.0
7.5
7.0
6.5
6.0
5.5
5.0
4.5
4.0
3.5
3.0
2.5
2.0
1.5
1.0
0.5
0.0
-0.5
-1
f1 (ppm)

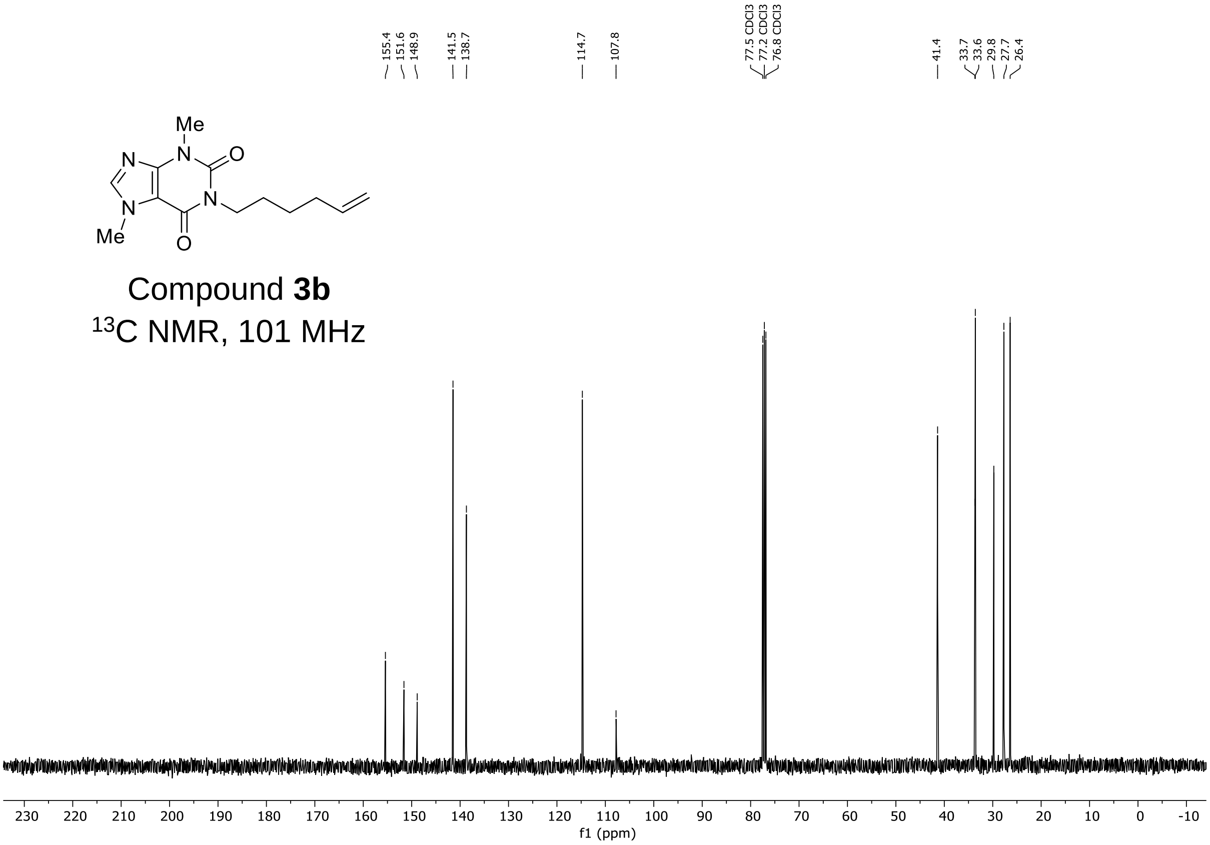
155.4
151.6
148.9
141.5
138.7
114.7
107.8
77.5 CDCl3
77.2 CDCl3
76.8 CDCl3
41.4
33.7
33.6
29.8
27.7
26.4
Me
N
O
N
N
N
Me
O
Compound 3b
13C NMR, 101 MHz
230
220
210
200
190
180
170
160
150
140
130
120
110
100
90
80
70
60
50
40
30
20
10
0
-10
f1 (ppm)

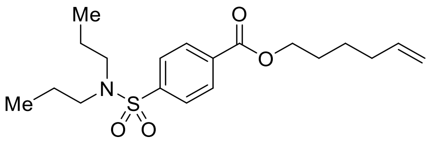

Compound **3c**
^{1}H NMR, 400 MHz

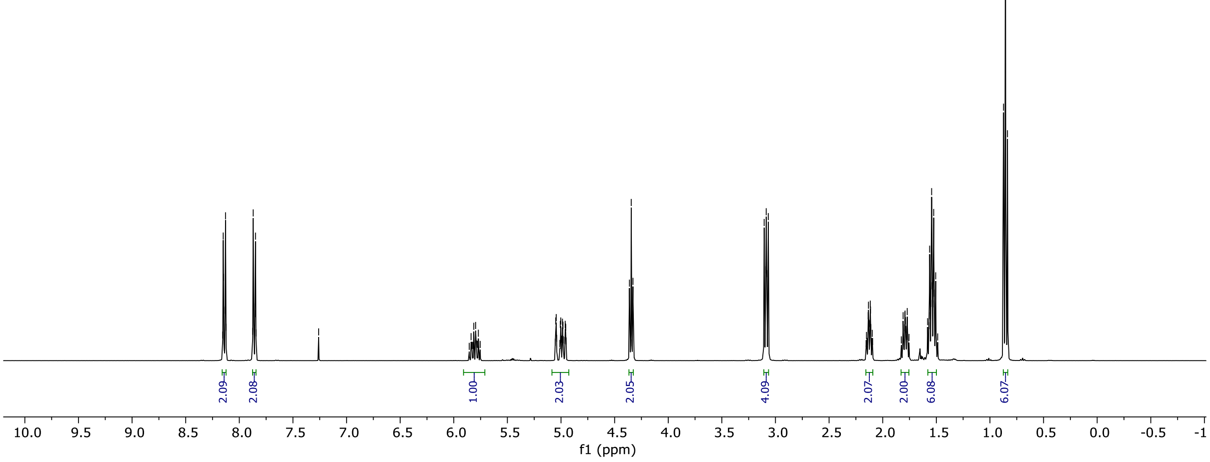

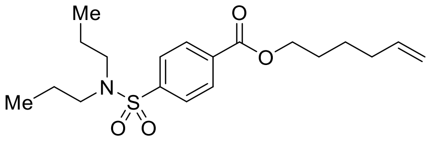

Compound **3c**
^{13}C NMR, 101 MHz

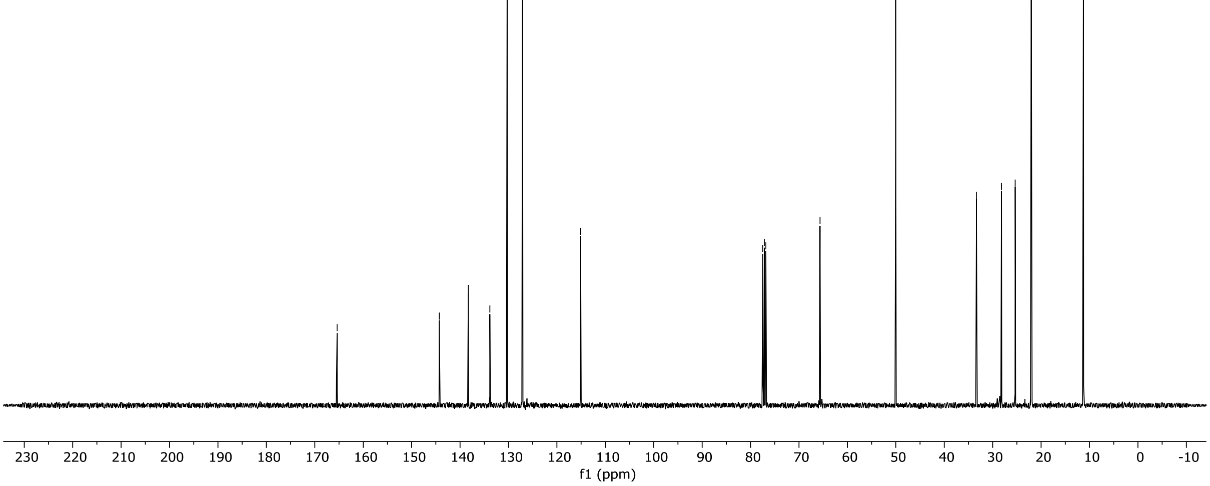

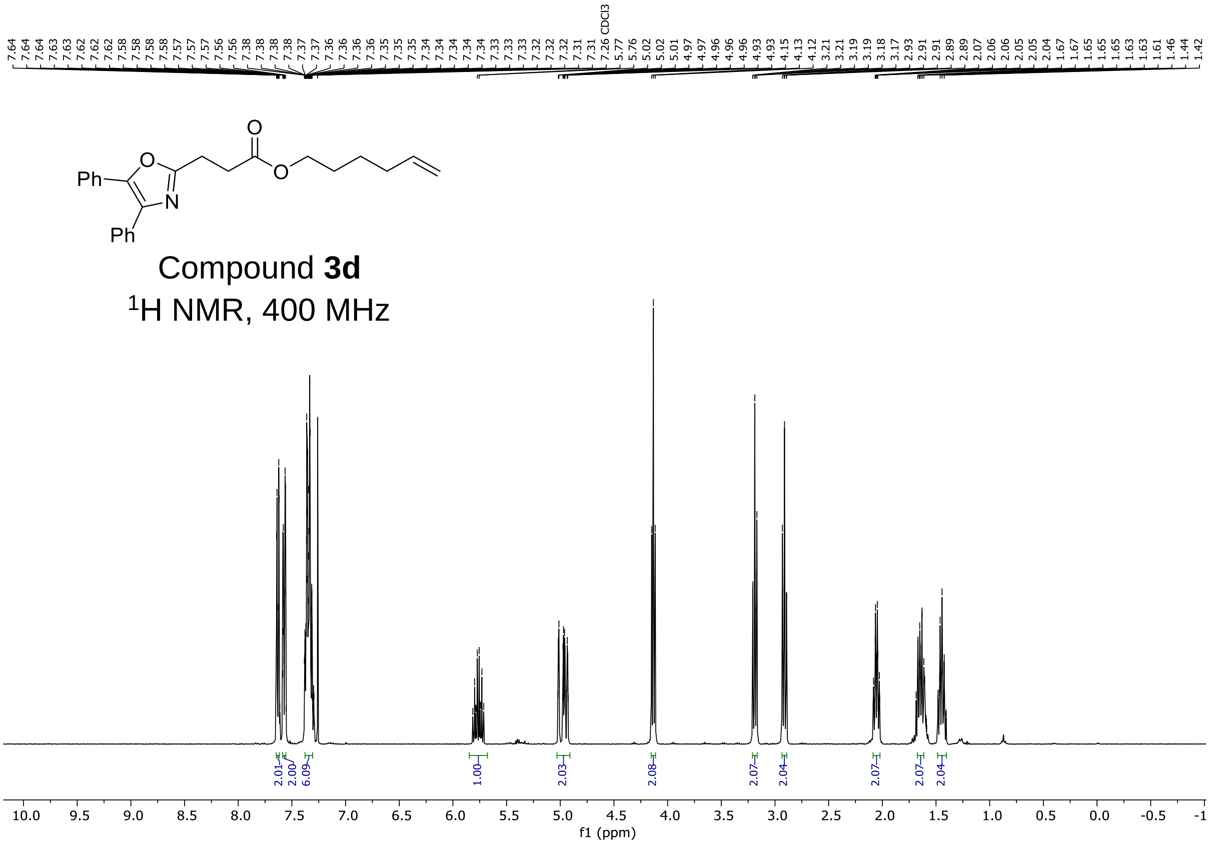
7.64 7.64 7.63 7.63 7.62 7.62 7.58 7.58 7.58 7.57 7.57 7.57 7.56 7.56 7.38 7.38 7.38 7.37 7.37 7.36 7.36 7.36 7.35 7.35 7.34 7.34 7.34 7.34 7.34 7.33 7.33 7.33 7.32 7.32 7.32 7.31 7.26 CDCl3 5.77 5.76 5.02 5.02 5.01 4.97 4.97 4.96 4.96 4.96 4.93 4.93 4.15 4.13 4.12 3.21 3.21 3.19 3.19 3.18 3.17 2.93 2.91 2.91 2.89 2.89 2.07 2.06 2.06 2.05 2.05 2.04 1.67 1.65 1.65 1.65 1.63 1.63 1.61 1.46 1.44 1.42
Ph
O
N
Ph
O
O
Compound 3d
1H NMR, 400 MHz
2.01 2.00 6.09 1.00 2.03 2.08 2.07 2.04 2.07 2.07 2.04
f1 (ppm)

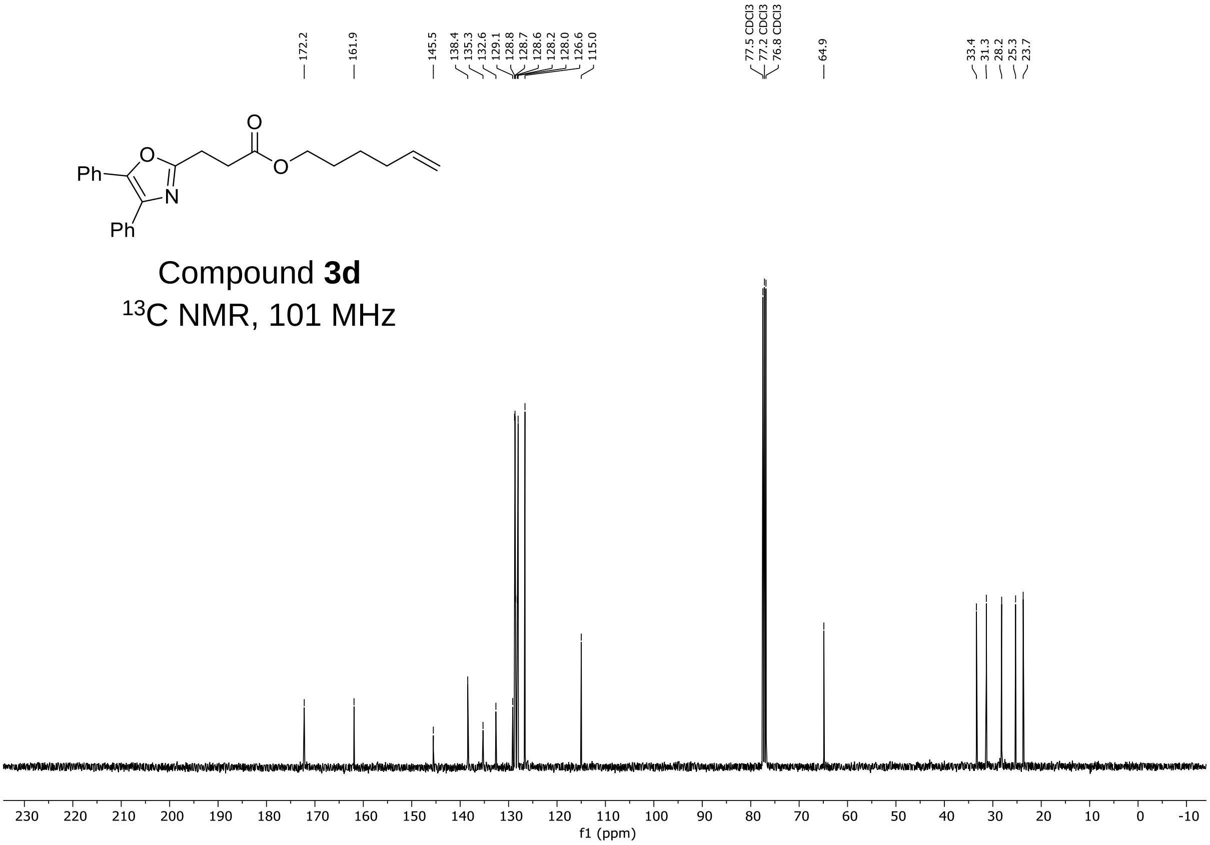
172.2 161.9 145.5 138.4 135.3 132.6 129.1 128.8 128.7 128.6 128.2 128.0 126.6 115.0 77.5 CDCl3 77.2 CDCl3 76.8 CDCl3 64.9 33.4 31.3 28.2 25.3 23.7
Ph
O
N
Ph
O
O
Compound 3d
13C NMR, 101 MHz
f1 (ppm)

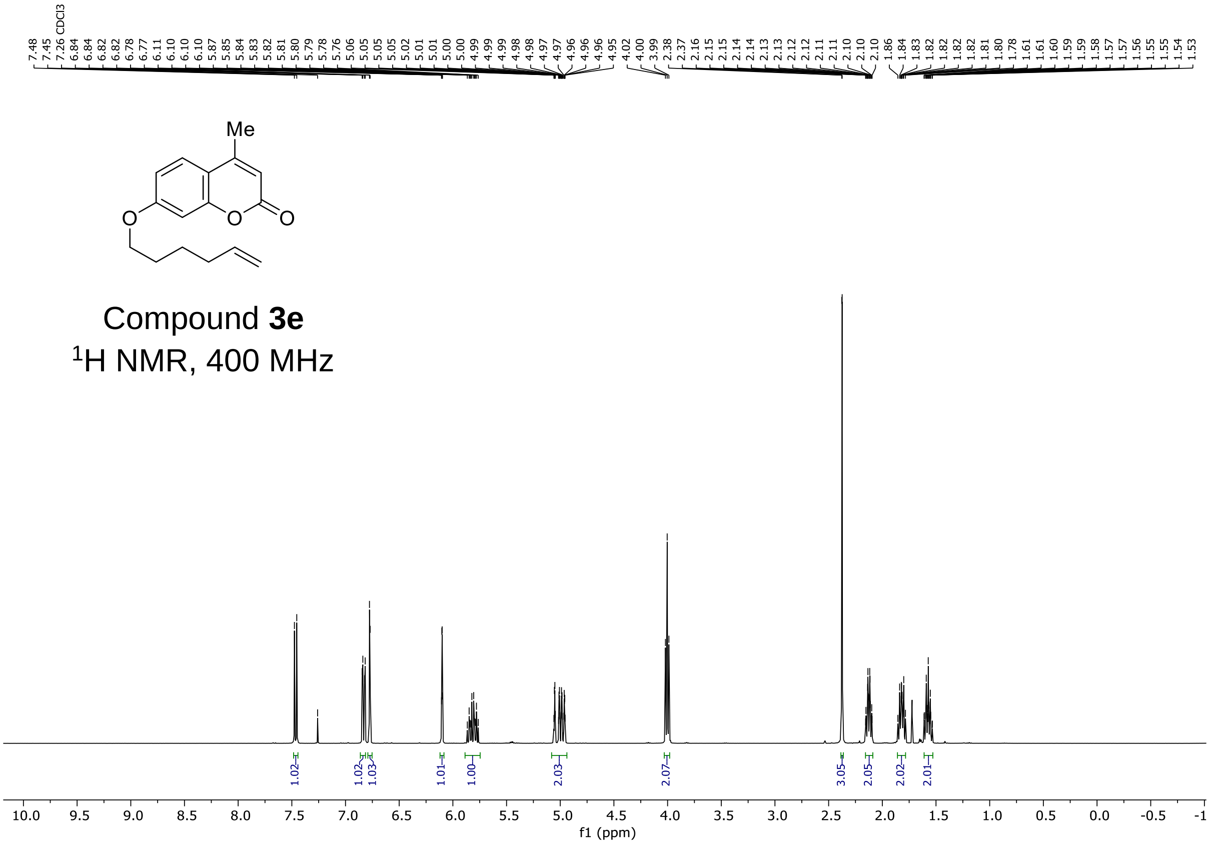

Compound **3e**
^{1}H NMR, 400 MHz

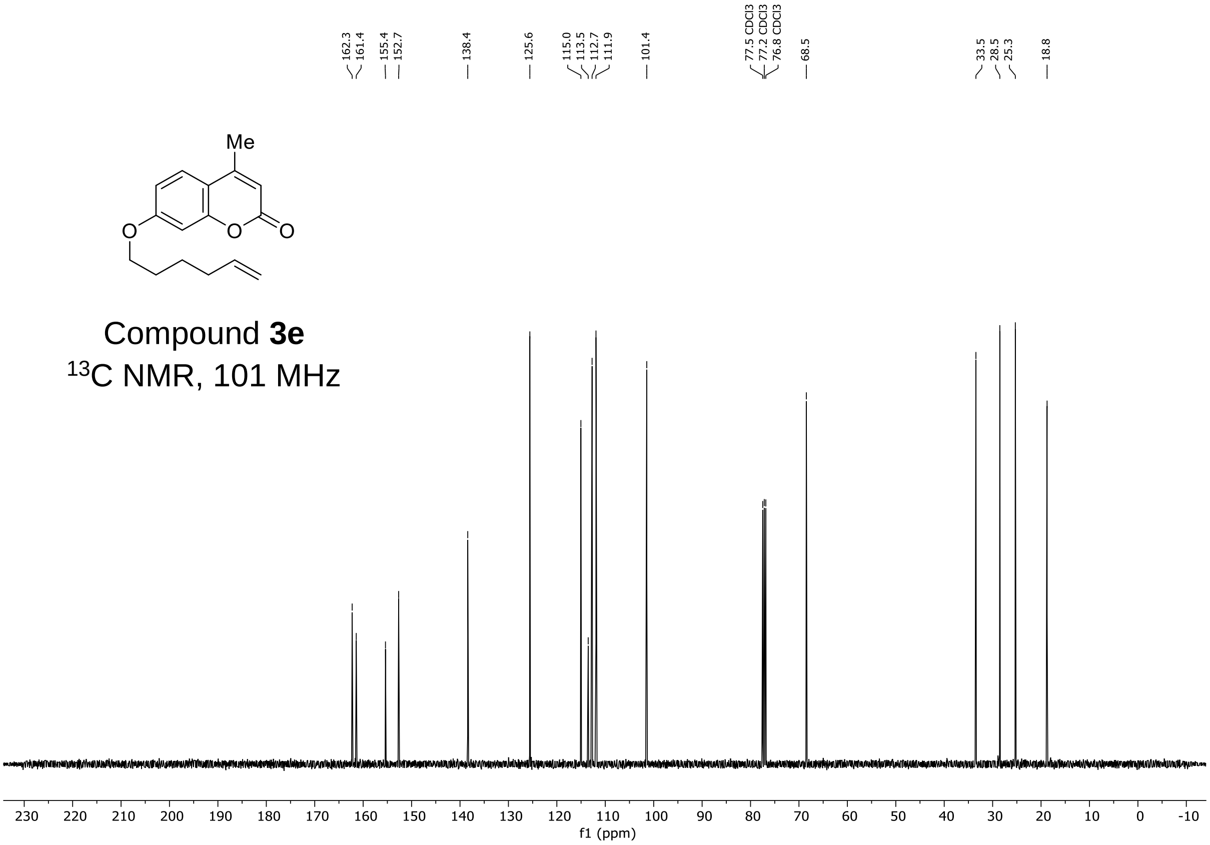

Compound **3e**
^{13}C NMR, 101 MHz

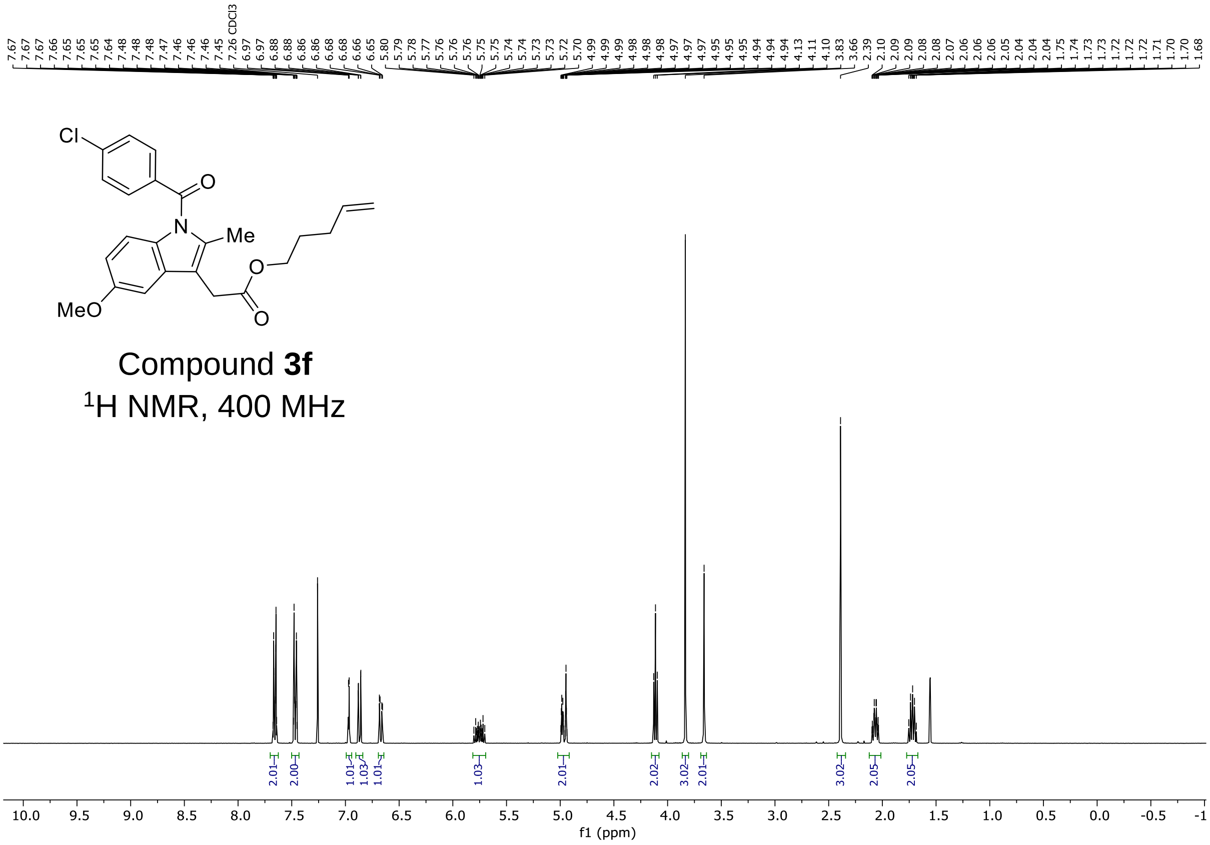

Compound **3f**
^{1}H NMR, 400 MHz

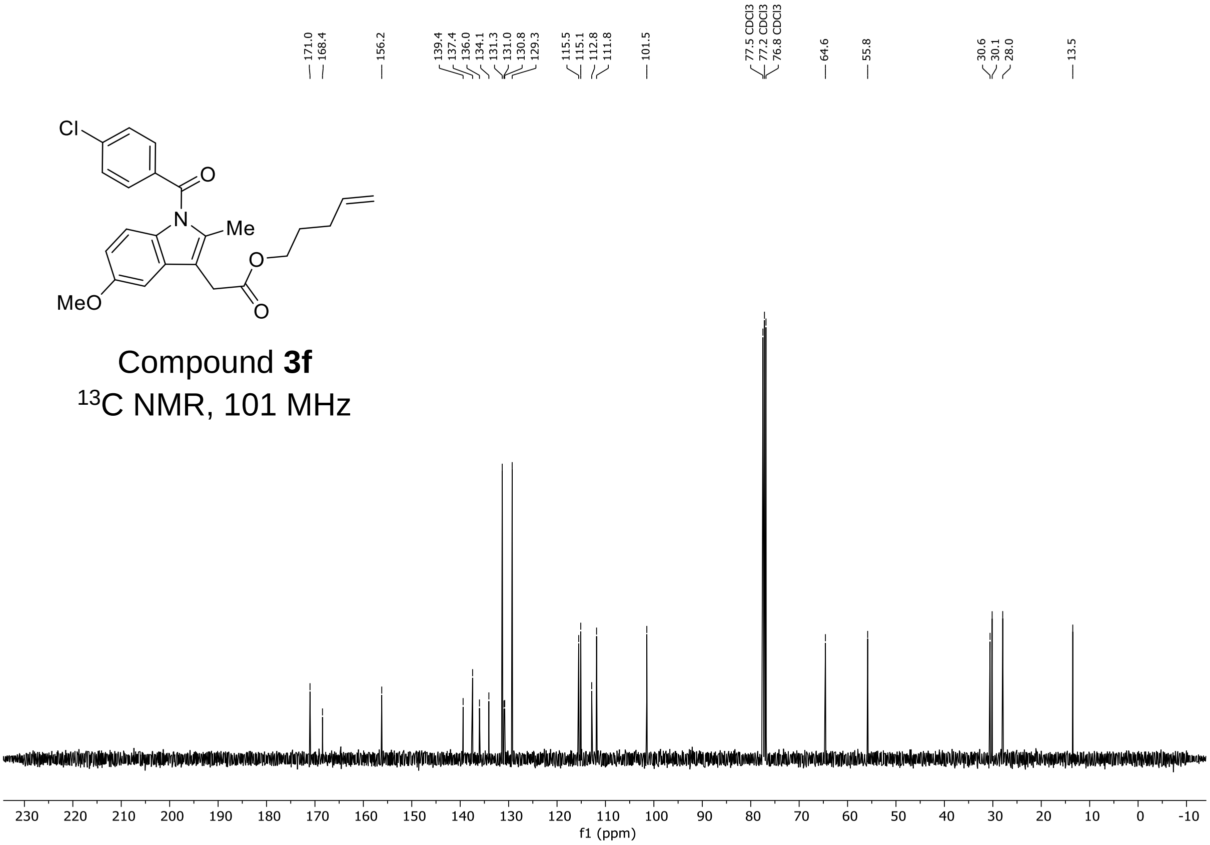

Compound **3f**
^{13}C NMR, 101 MHz

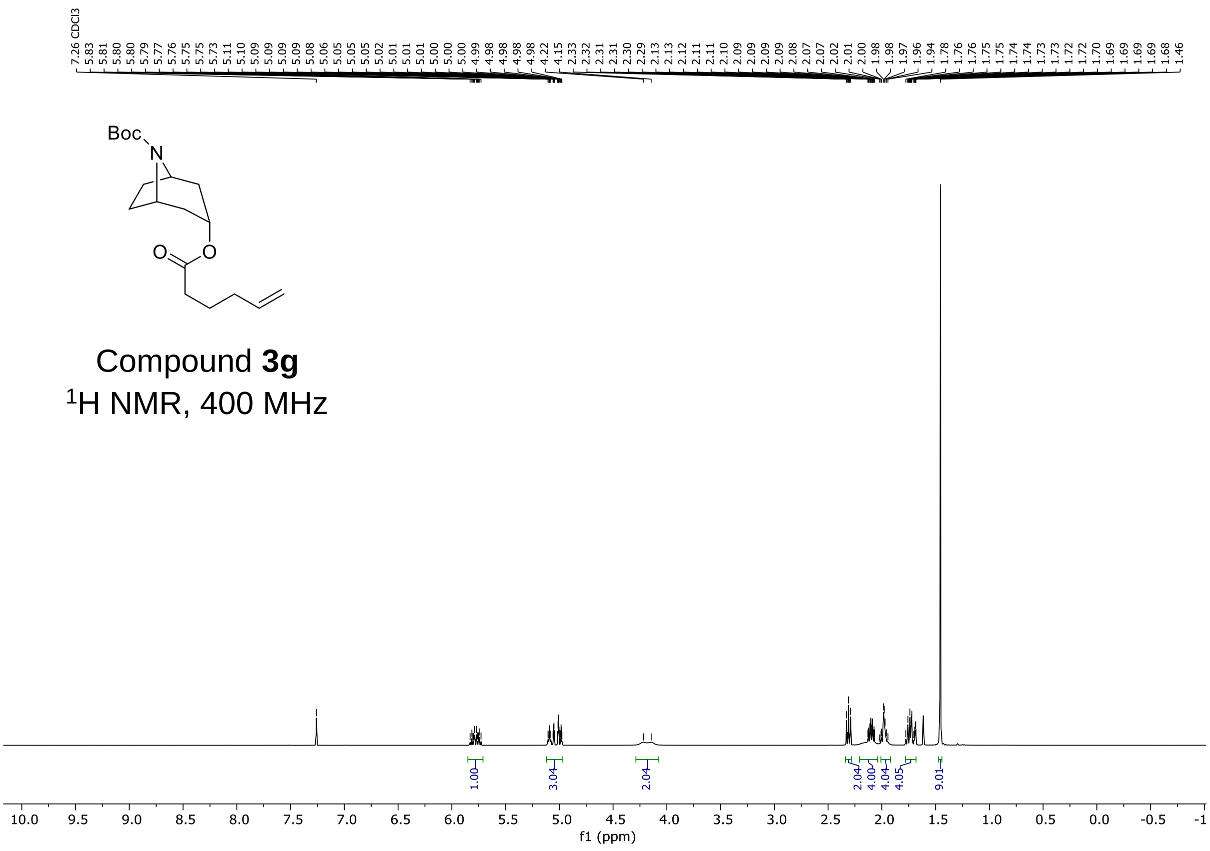

Compound **3g**
^{1}H NMR, 400 MHz

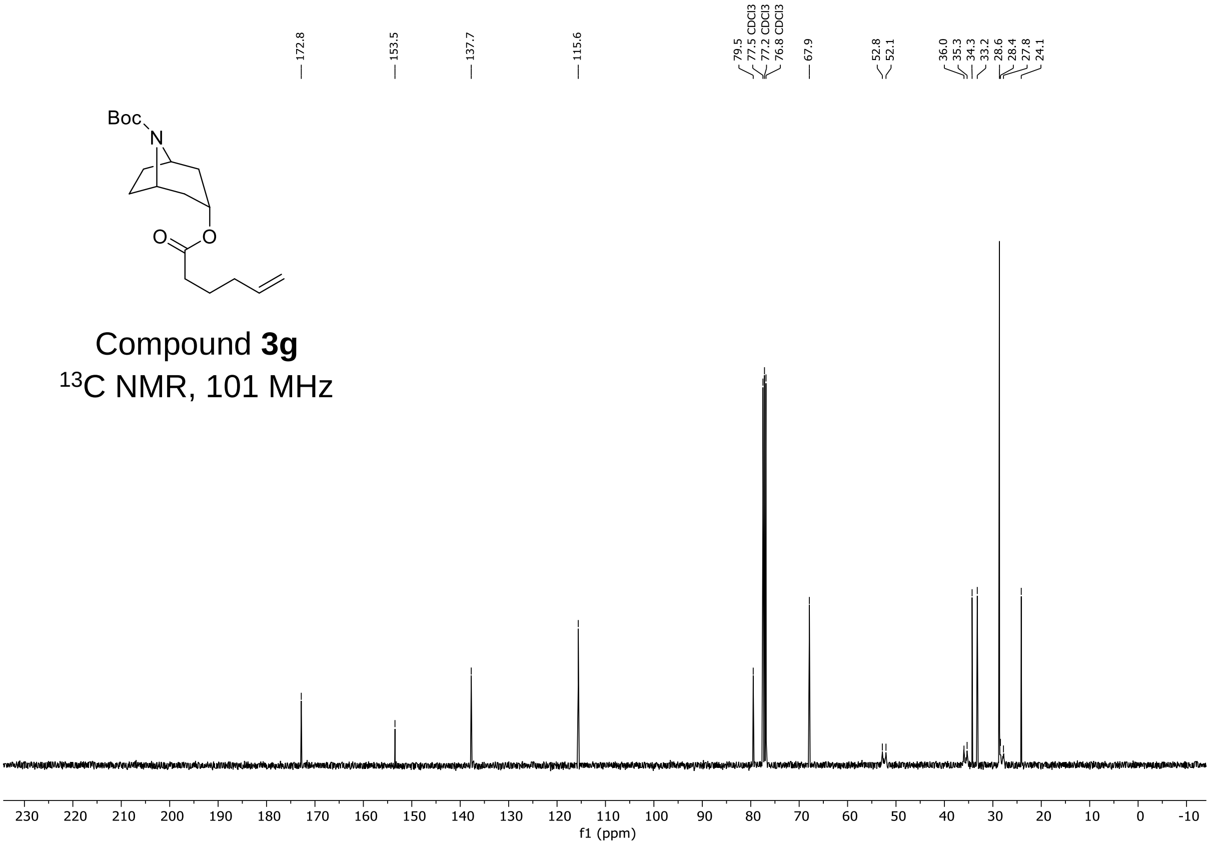

Compound **3g**
^{13}C NMR, 101 MHz

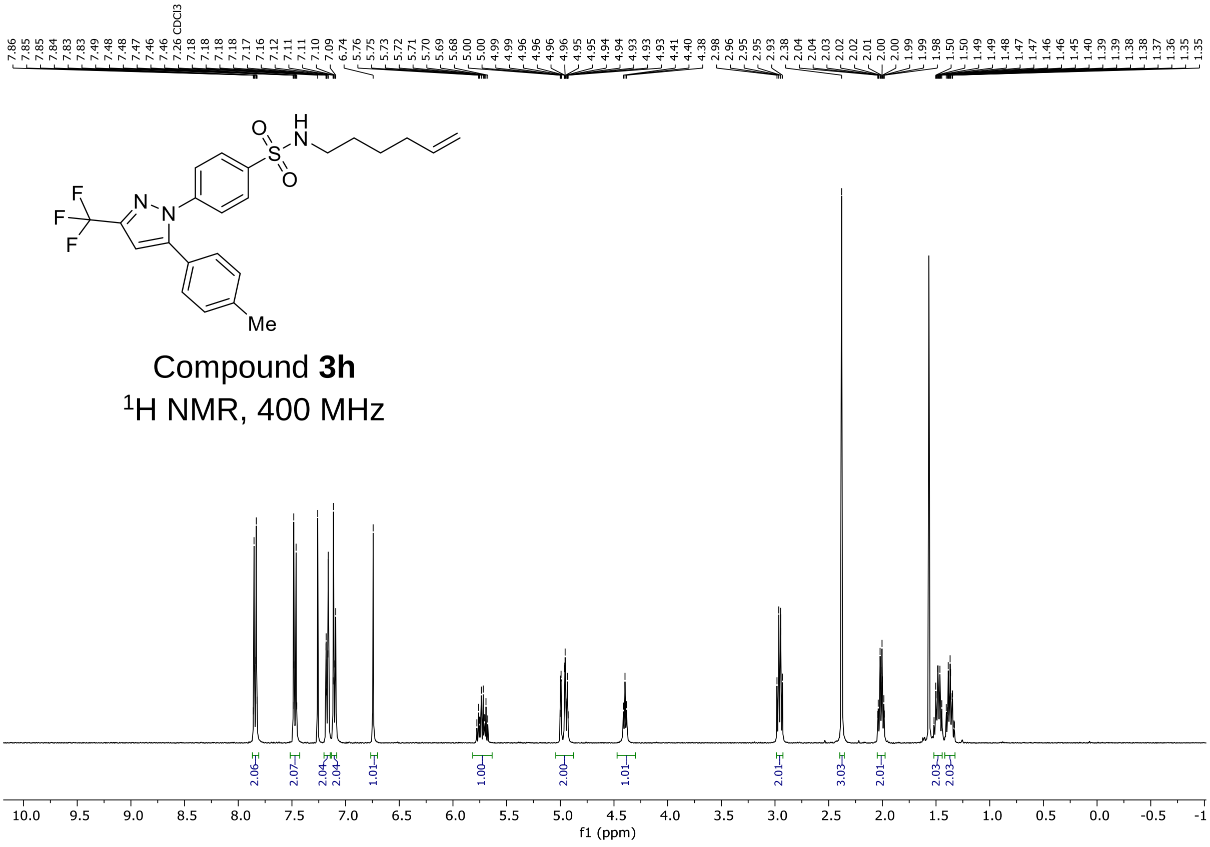
7.86 7.85 7.85 7.84 7.83 7.83 7.49 7.48 7.48 7.47 7.46 7.46 7.26 CDCl3 7.18 7.18 7.18 7.18 7.17 7.16 7.12 7.11 7.11 7.10 7.09 6.74 5.76 5.75 5.73 5.72 5.71 5.70 5.69 5.68 5.00 5.00 4.99 4.99 4.96 4.96 4.96 4.96 4.95 4.95 4.94 4.94 4.93 4.93 4.93 4.93 4.41 4.40 4.38 2.98 2.96 2.95 2.95 2.93 2.38 2.04 2.04 2.03 2.02 2.02 2.01 2.00 2.00 1.99 1.99 1.98 1.50 1.50 1.49 1.49 1.48 1.47 1.47 1.46 1.45 1.40 1.39 1.39 1.38 1.38 1.37 1.36 1.35 1.35
Compound **3h**
^{1}H NMR, 400 MHz
2.06 2.07 2.04 2.04 1.01 1.00 2.00 1.01 2.01 3.03 2.01 2.03 2.03
f1 (ppm)

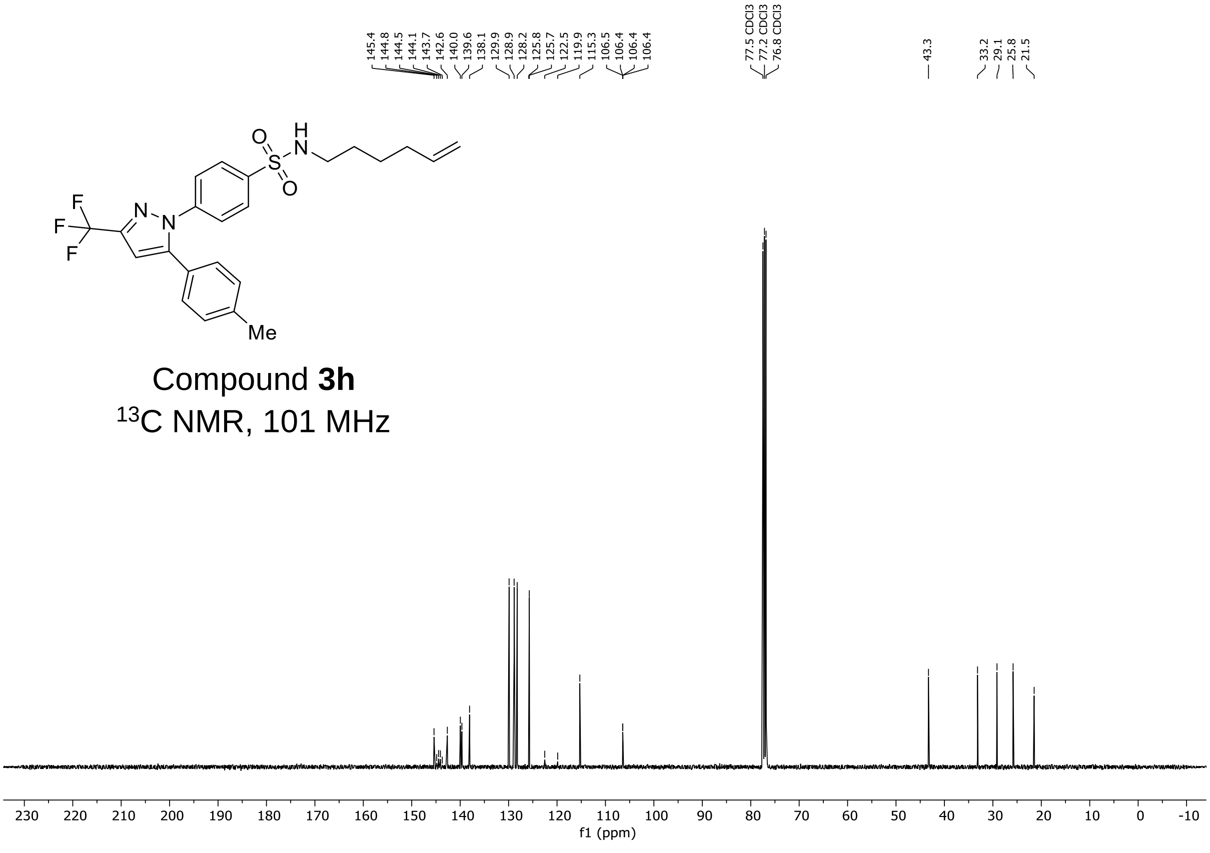
145.4 144.8 144.5 144.1 143.7 142.6 140.0 139.6 138.1 129.9 128.9 128.2 125.8 125.7 122.5 119.9 115.3 106.5 106.4 106.4 106.4
77.5 CDCl3 77.2 CDCl3 76.8 CDCl3
43.3
33.2 29.1 25.8 21.5
Compound **3h**
^{13}C NMR, 101 MHz
f1 (ppm)

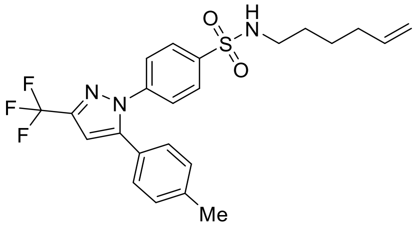

Compound **3h**
^{19}F NMR, 376 MHz

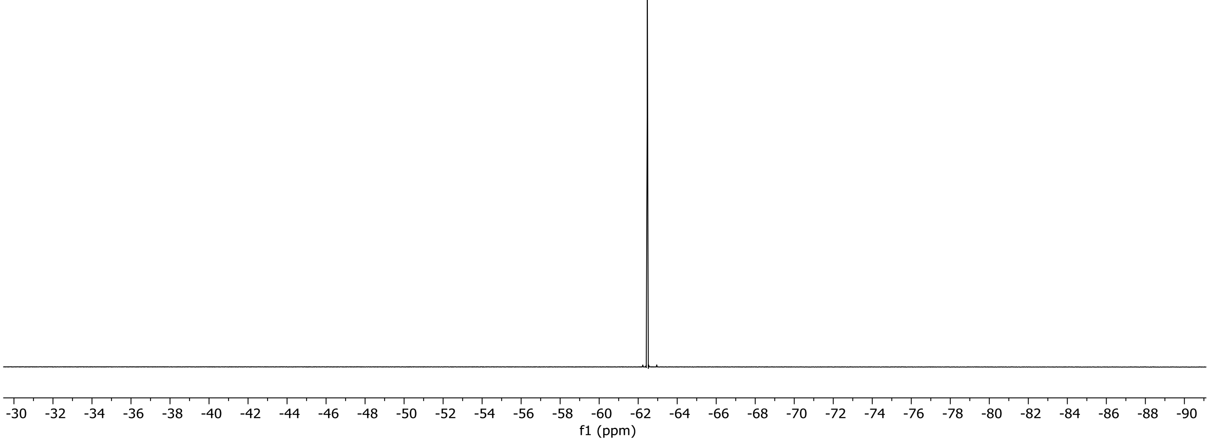

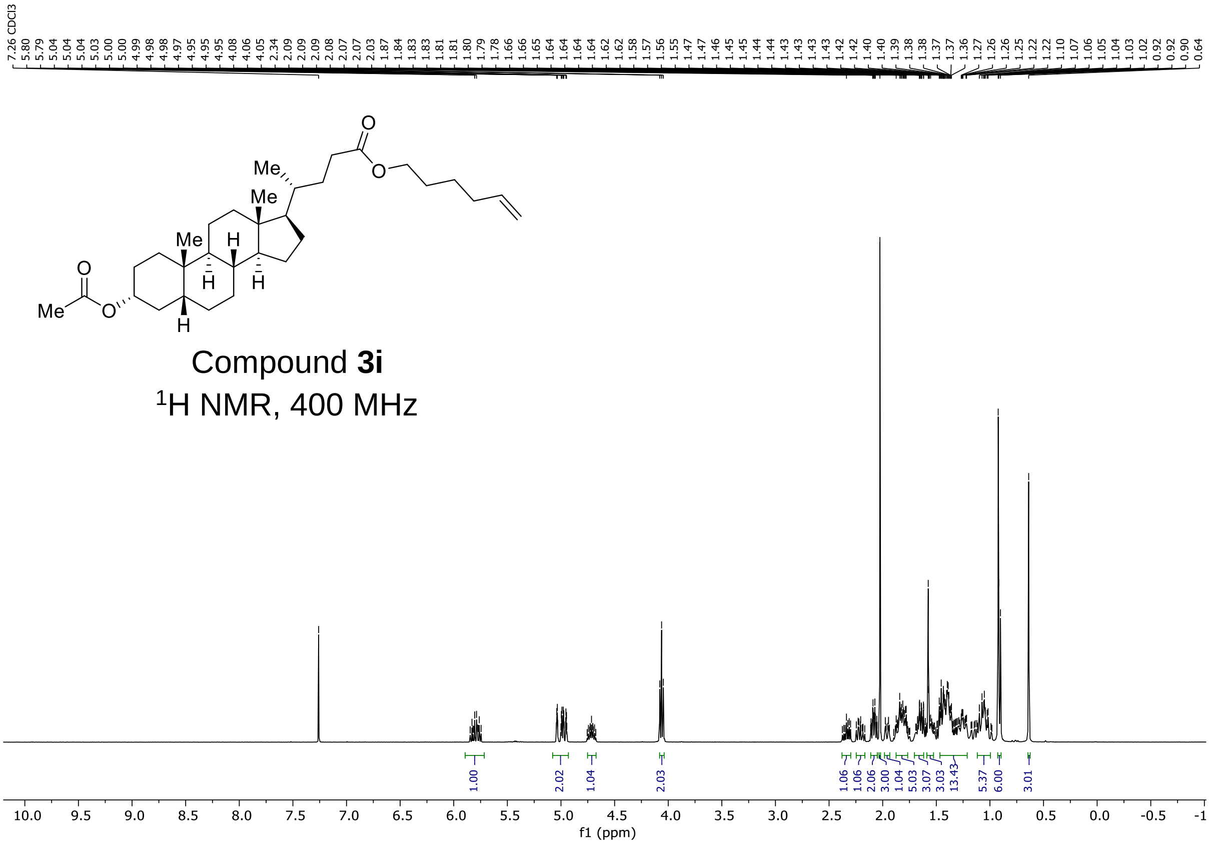
7.26 CDCl3
5.80 5.79 5.04 5.04 5.04 5.03 5.00 5.00 4.99 4.98 4.98 4.97 4.95 4.95 4.95 4.08 4.06 4.05 2.34 2.09 2.09 2.09 2.08 2.07 2.07 2.03 1.87 1.84 1.83 1.83 1.81 1.81 1.80 1.79 1.78 1.66 1.66 1.65 1.64 1.64 1.64 1.64 1.62 1.62 1.58 1.57 1.56 1.55 1.47 1.46 1.45 1.45 1.44 1.44 1.43 1.43 1.43 1.43 1.42 1.42 1.40 1.40 1.39 1.38 1.38 1.37 1.37 1.36 1.27 1.26 1.26 1.25 1.22 1.22 1.10 1.07 1.06 1.05 1.04 1.03 1.02 0.92 0.92 0.90 0.64
Me
O
Compound **3i**
^{1}H NMR, 400 MHz
1.00 2.02 1.04 2.03 1.06 1.06 2.06 3.00 1.04 5.03 3.07 3.03 13.43 5.37 6.00 3.01
f1 (ppm)

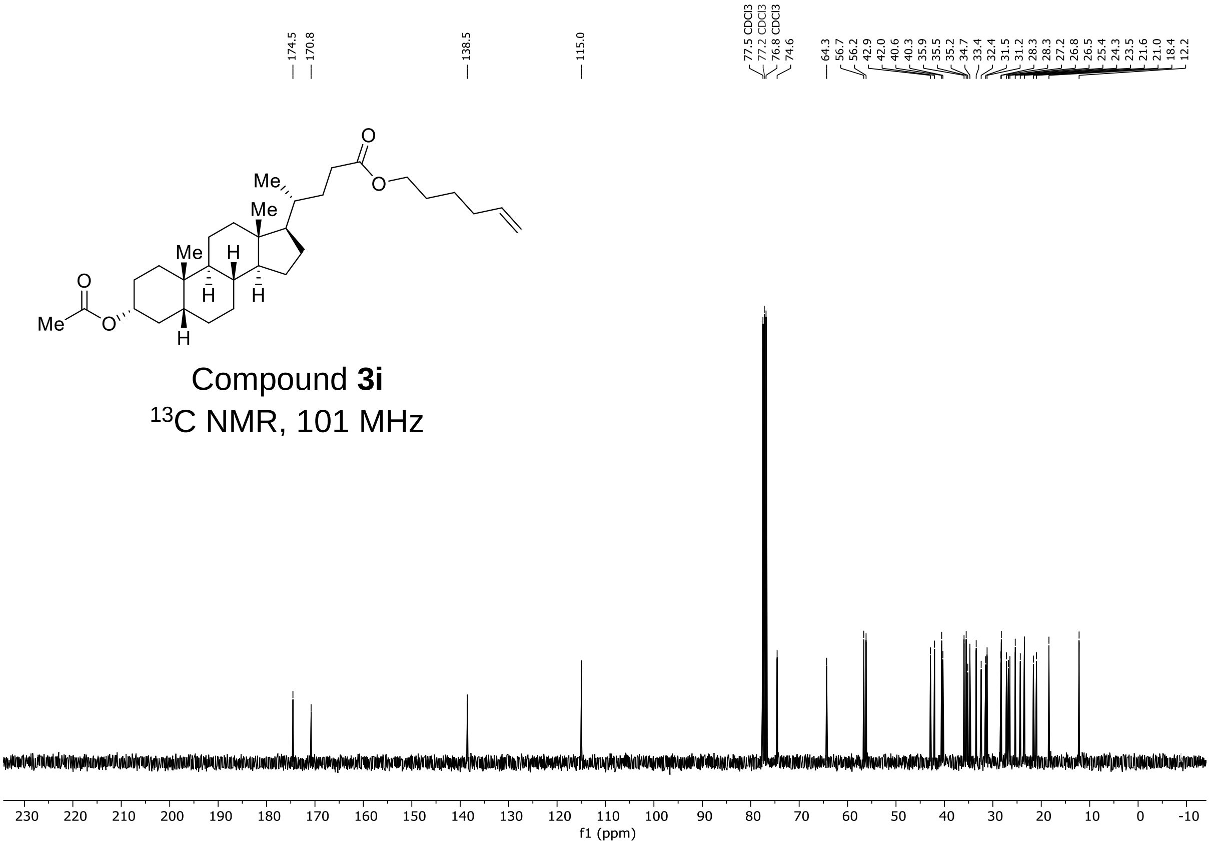
174.5 170.8 138.5 115.0 77.5 CDCl3 77.2 CDCl3 76.8 CDCl3 74.6 64.3 56.7 56.2 42.9 42.0 40.6 40.3 35.9 35.5 35.2 34.7 33.4 32.4 31.5 31.2 28.3 28.3 27.2 26.8 26.5 25.4 24.3 23.5 21.6 21.0 18.4 12.2
Compound **3i**
^{13}C NMR, 101 MHz
f1 (ppm)

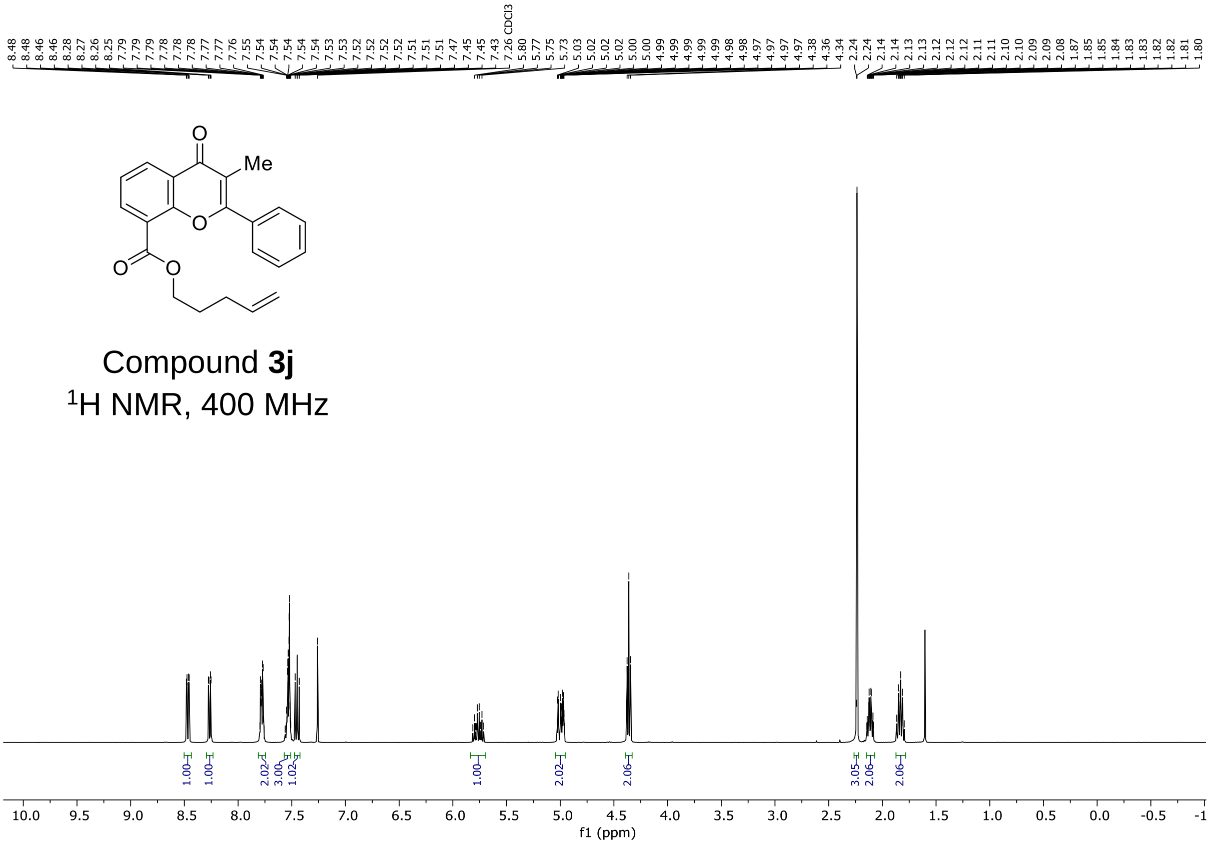

Compound **3j**
^{1}H NMR, 400 MHz

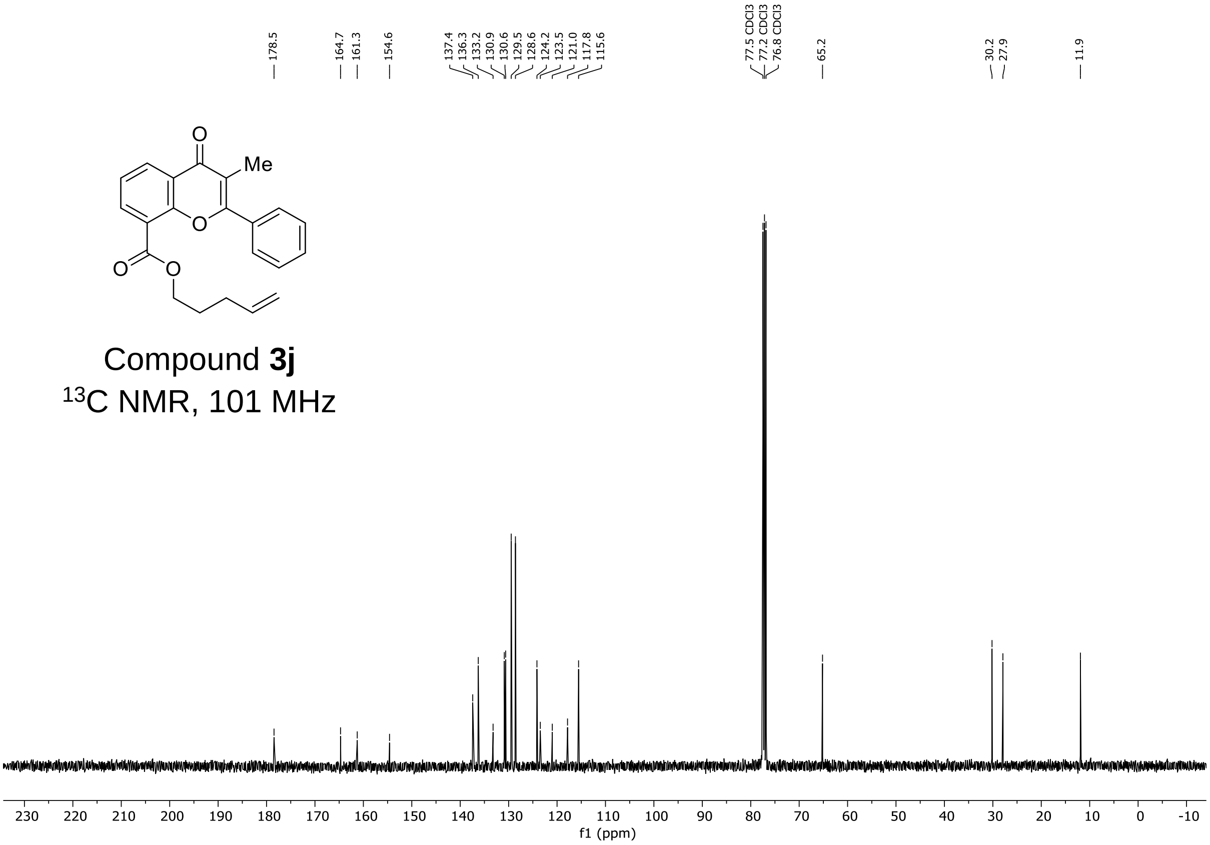

Compound **3j**
^{13}C NMR, 101 MHz

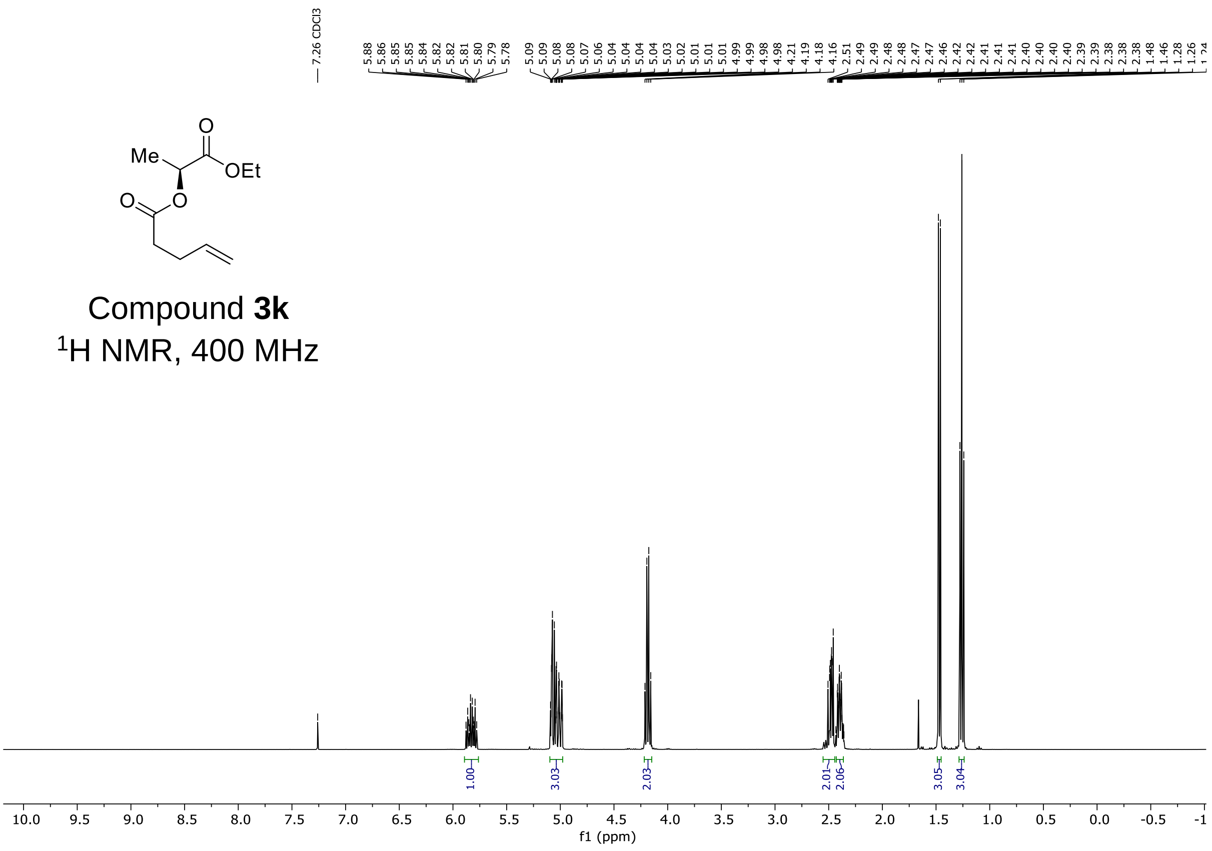
Me
O
OEt
O
O
Compound **3k**
^{1}H NMR, 400 MHz
7.26 CDCl3
1.00
3.03
2.03
2.01
2.06
3.05
3.04
f1 (ppm)

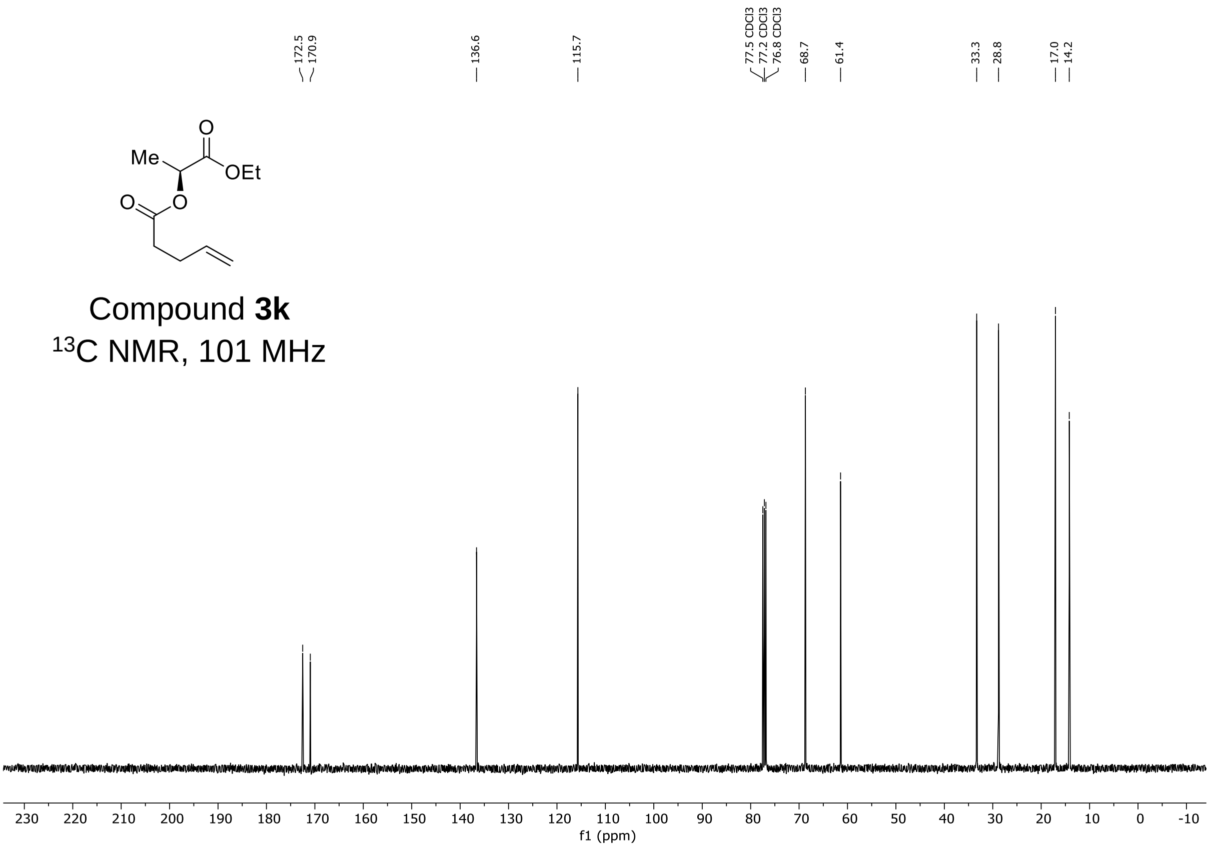
Me
O
OEt
O
O
Compound **3k**
^{13}C NMR, 101 MHz
172.5
170.9
136.6
115.7
77.5 CDCl3
77.2 CDCl3
76.8 CDCl3
68.7
61.4
33.3
28.8
17.0
14.2
f1 (ppm)

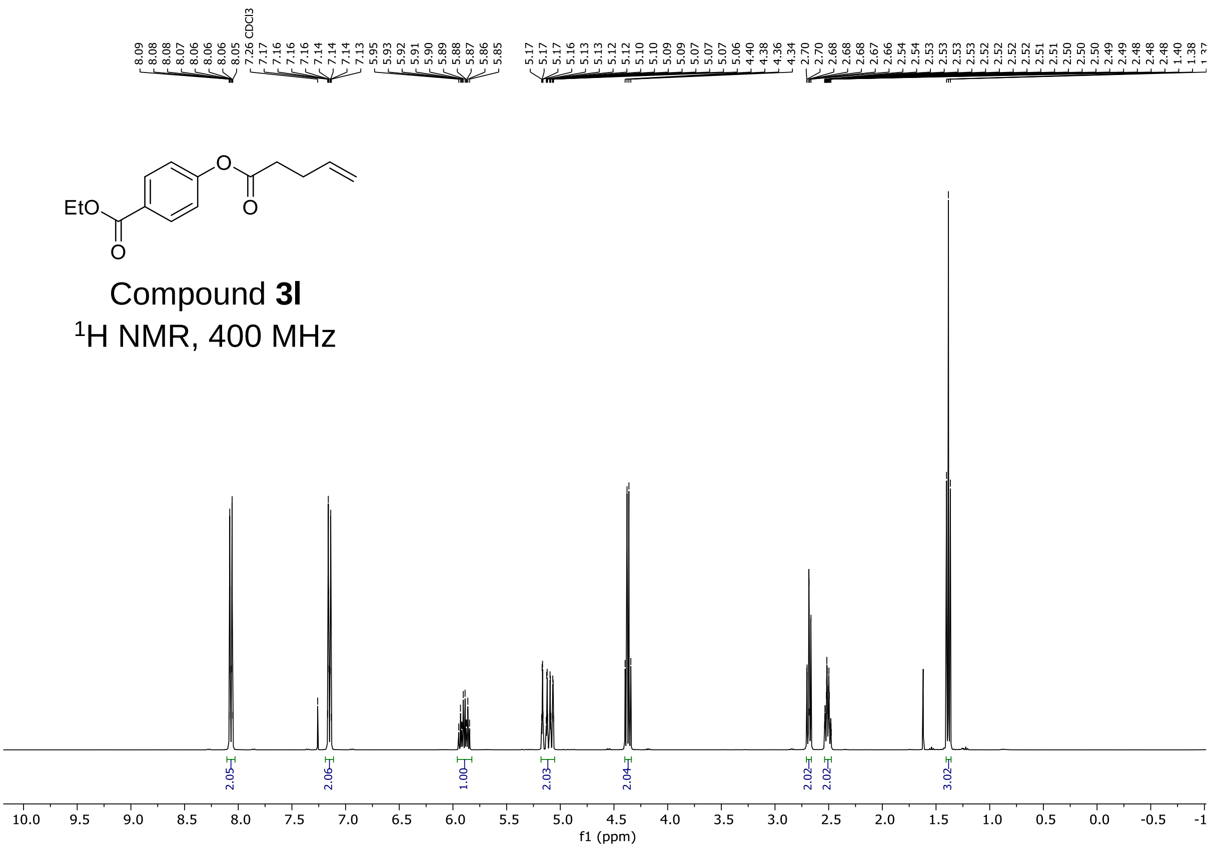
EtO
O
O
O
Compound 3l
1H NMR, 400 MHz
7.26 CDCl3
2.05
2.06
1.00
2.03
2.04
2.02
2.02
3.02
f1 (ppm)

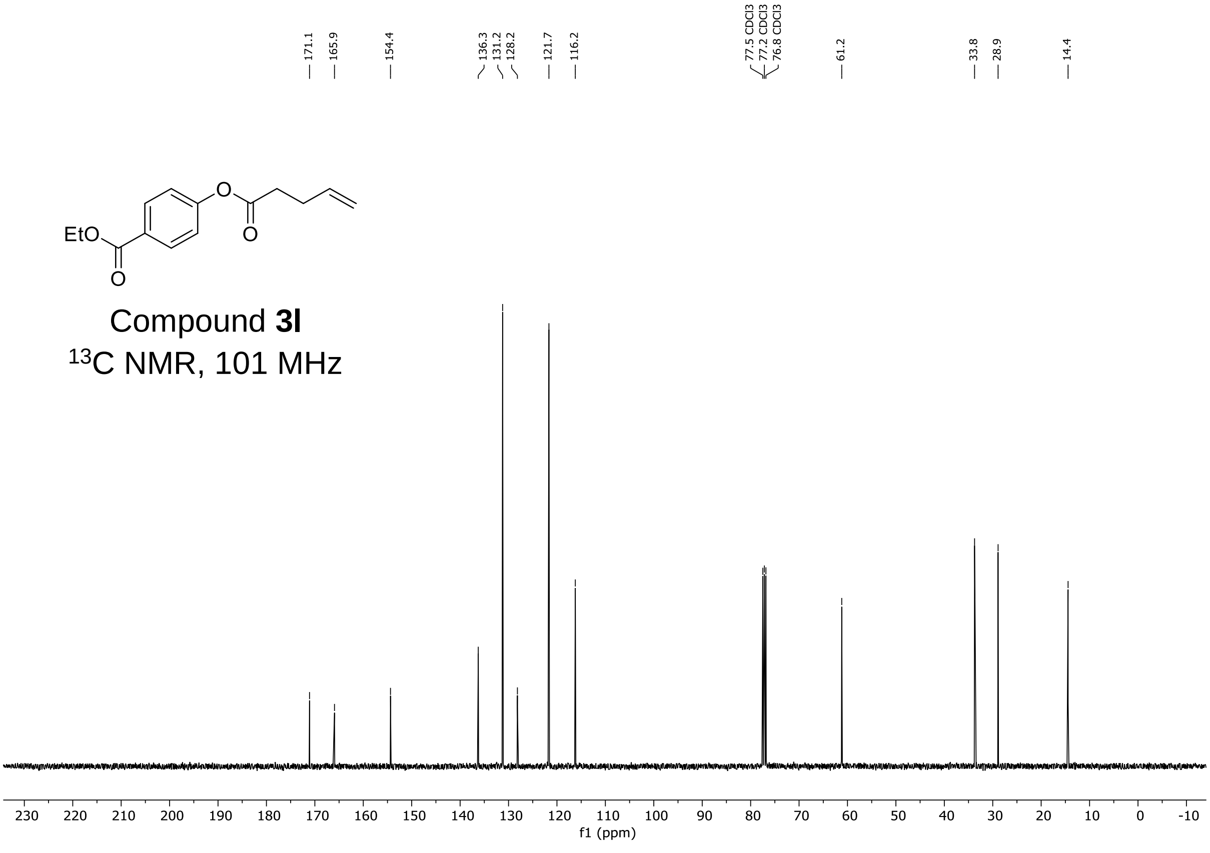
171.1
165.9
154.4
136.3
131.2
128.2
121.7
116.2
77.5 CDCl3
77.2 CDCl3
76.8 CDCl3
61.2
33.8
28.9
14.4
EtO
O
O
O
Compound 3l
13C NMR, 101 MHz
f1 (ppm)

Compound **3m**

^{1}H NMR, 400 MHz

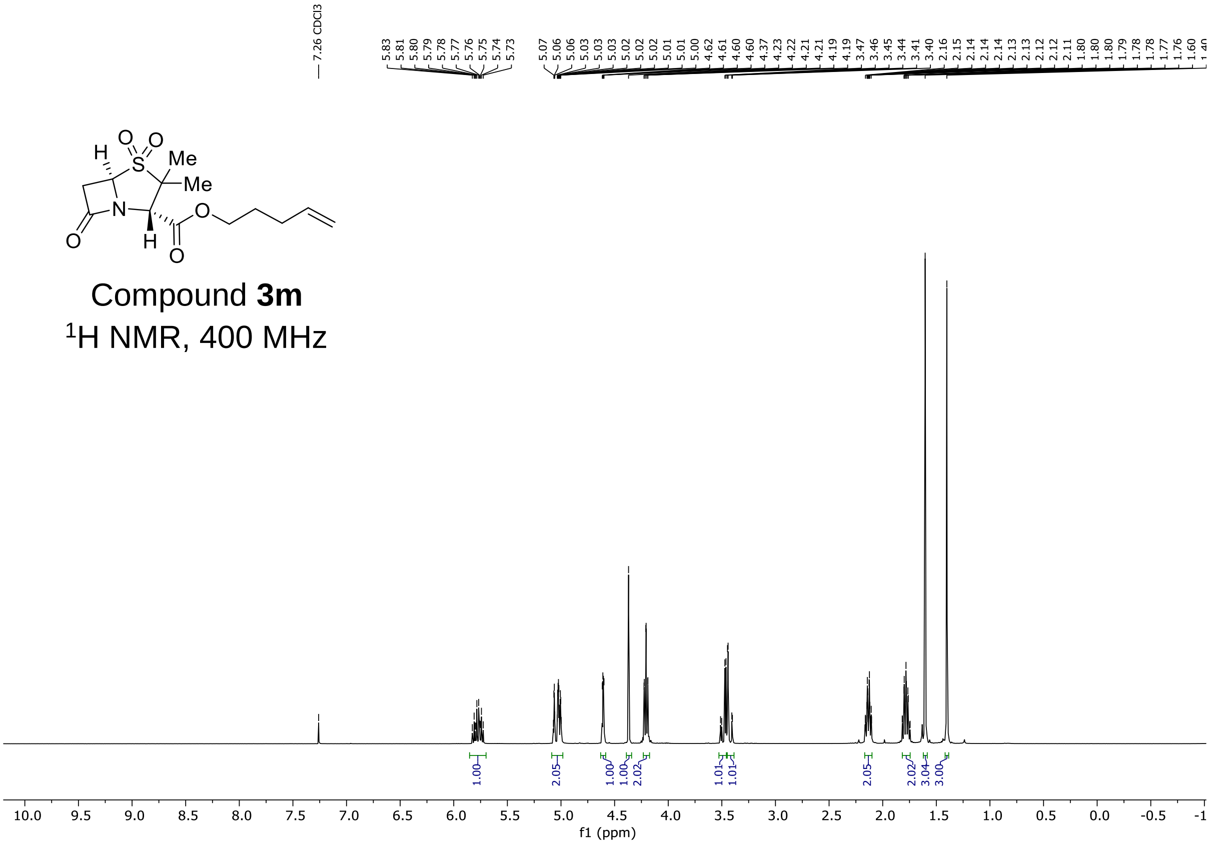

Compound **3m**

^{13}C NMR, 101 MHz

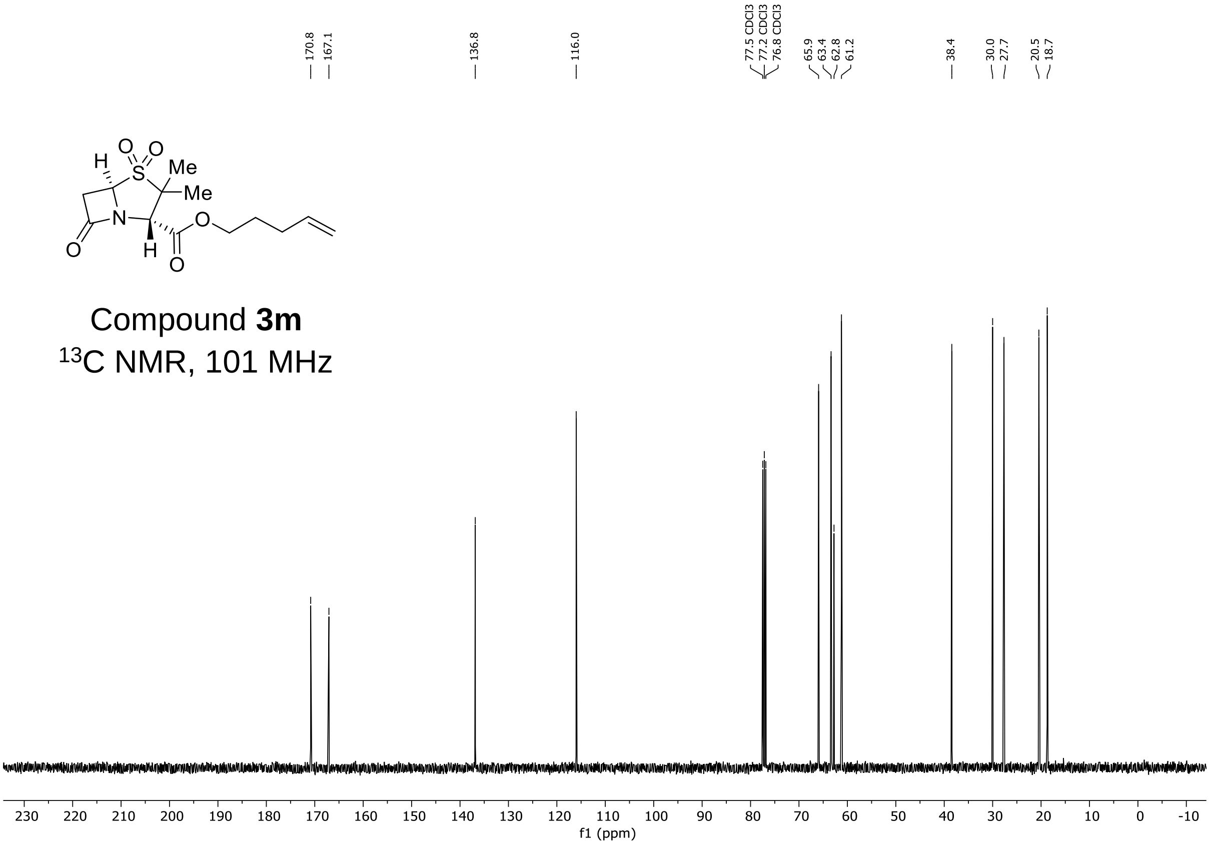

Compound **3o**
^{1}H NMR, 400 MHz

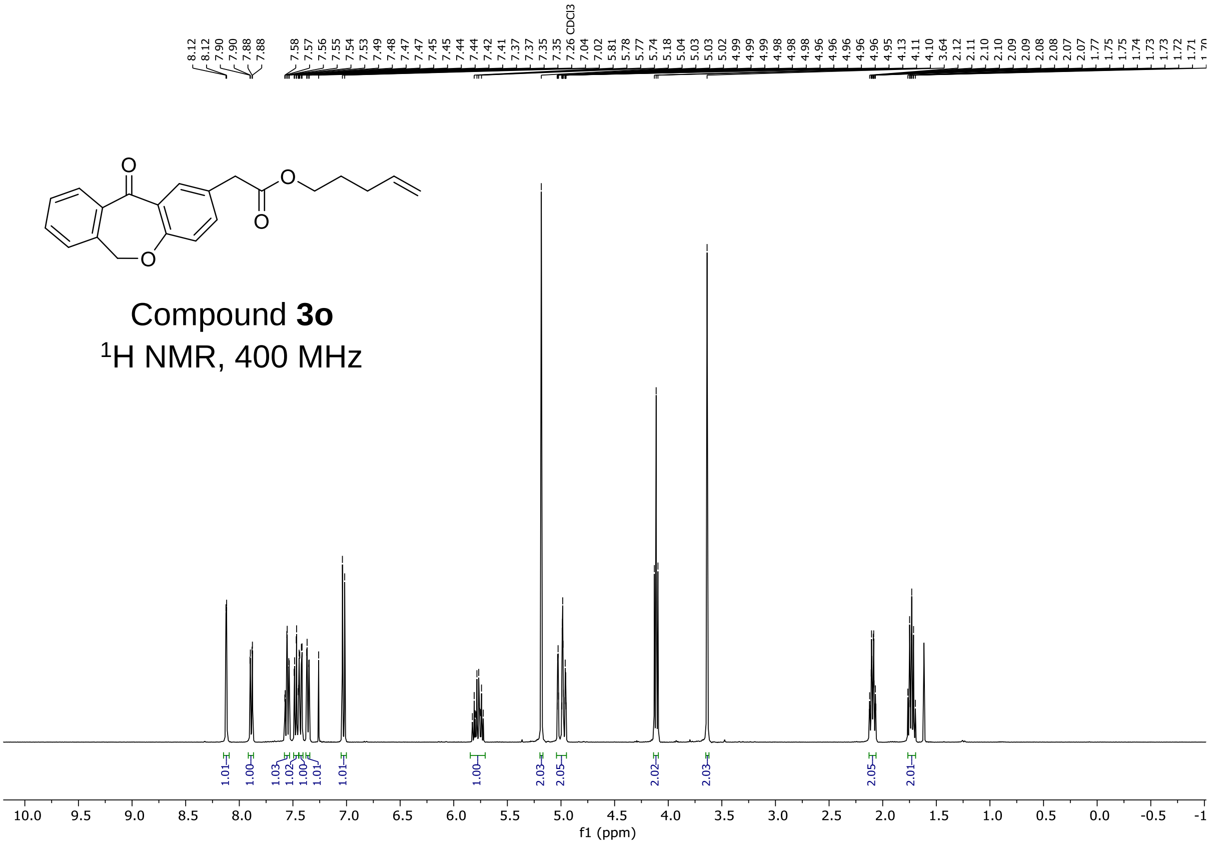

Compound **3o**
^{13}C NMR, 101 MHz

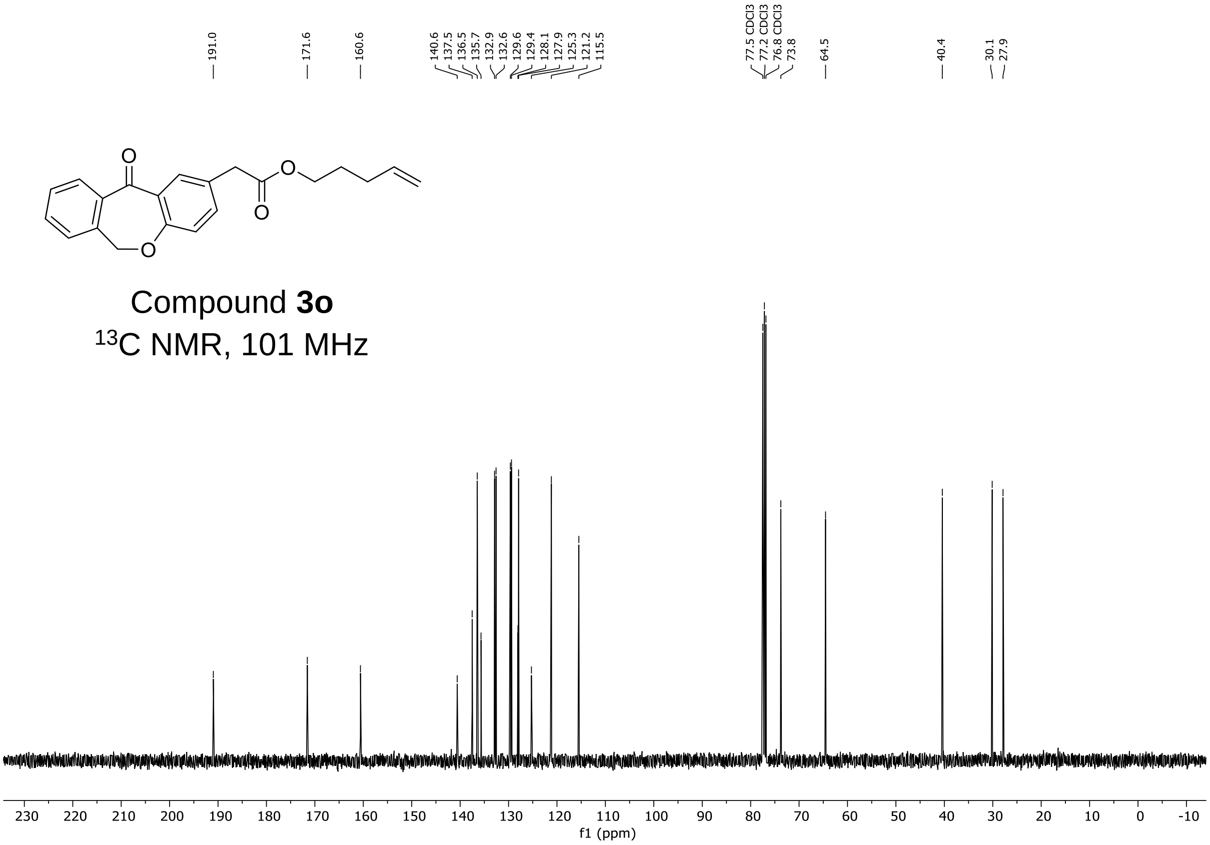

Compound **3p**
^{1}H NMR, 400 MHz

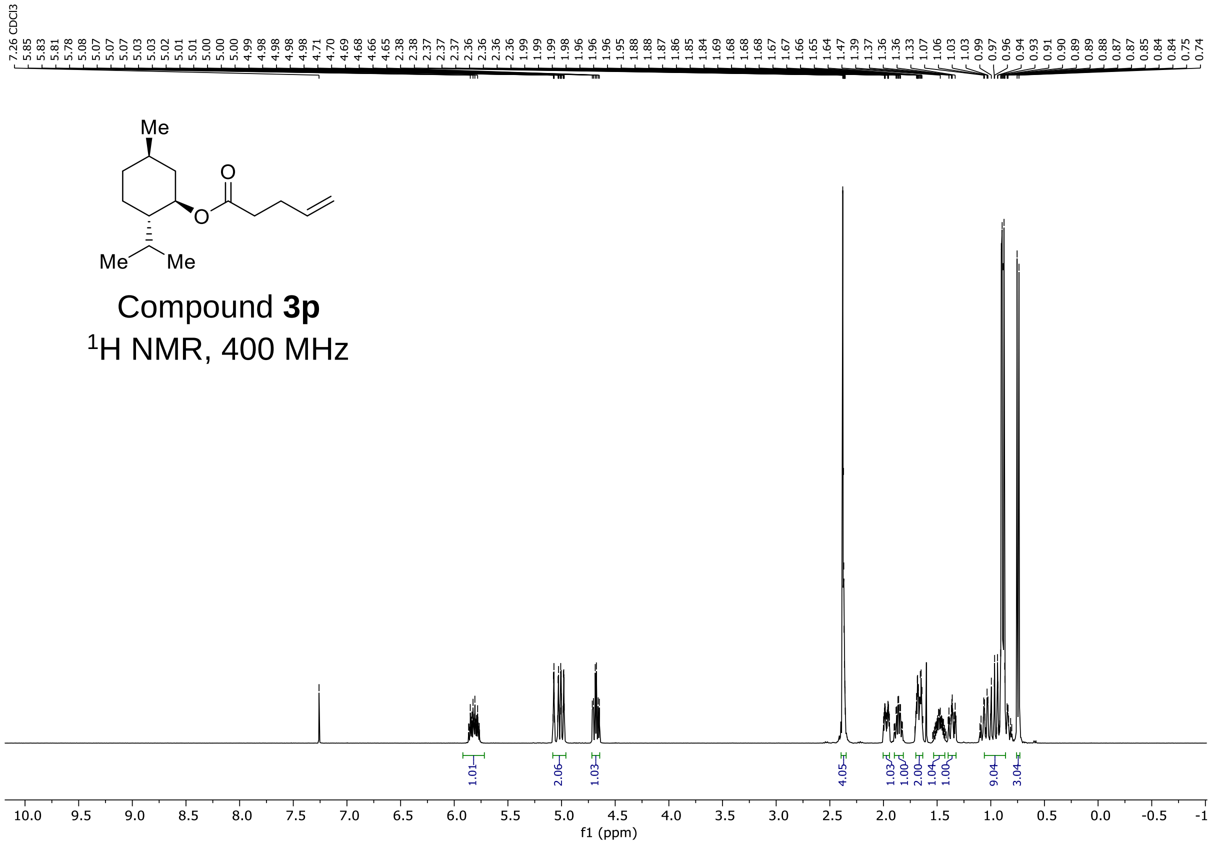

Compound **3p**
^{13}C NMR, 101 MHz

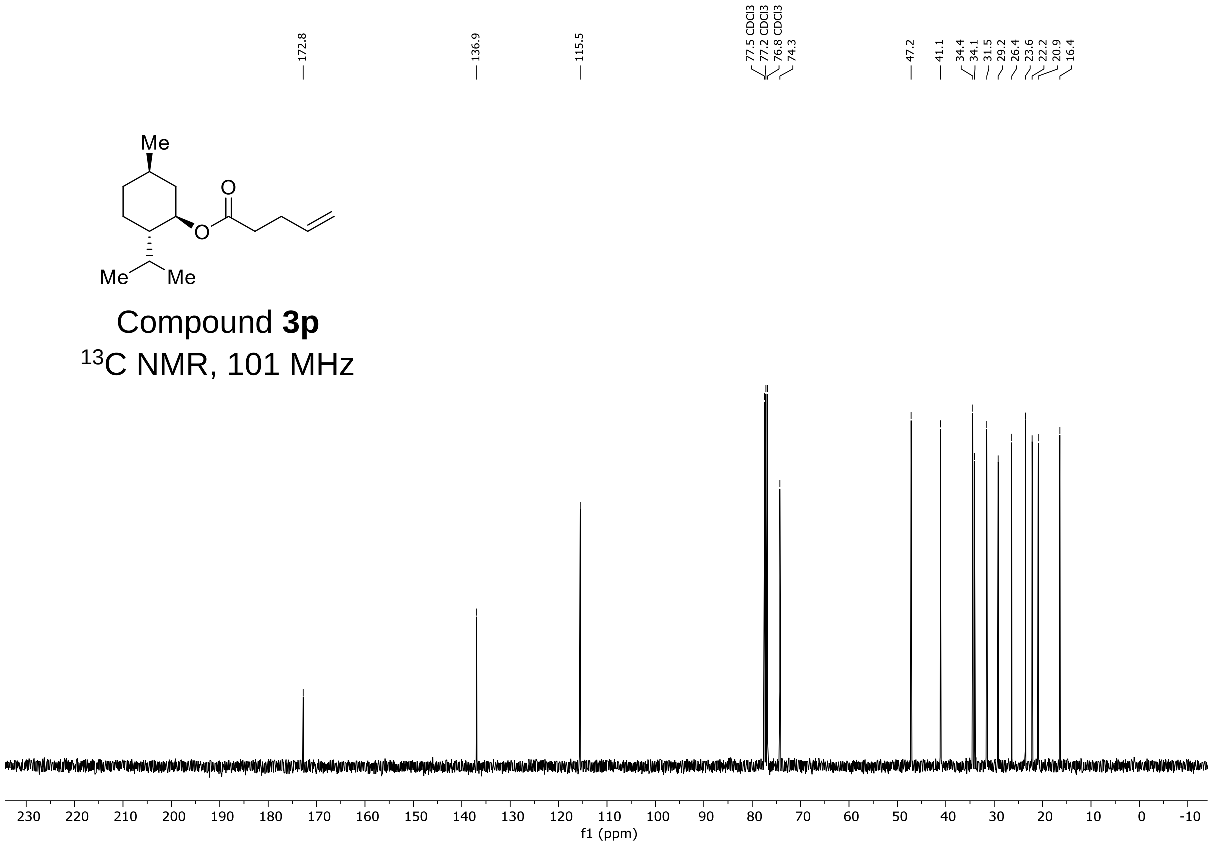

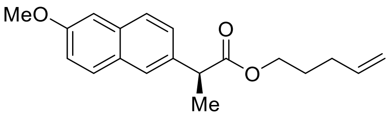

Compound **3q**
^{1}H NMR, 400 MHz

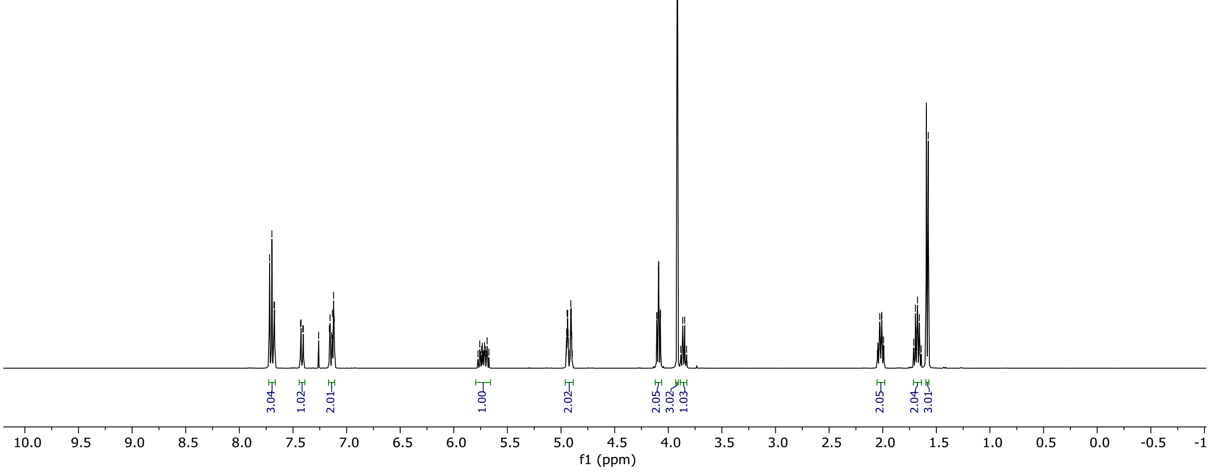

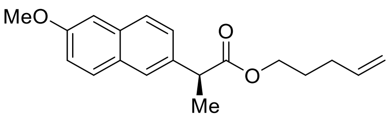

Compound **3q**
^{13}C NMR, 101 MHz

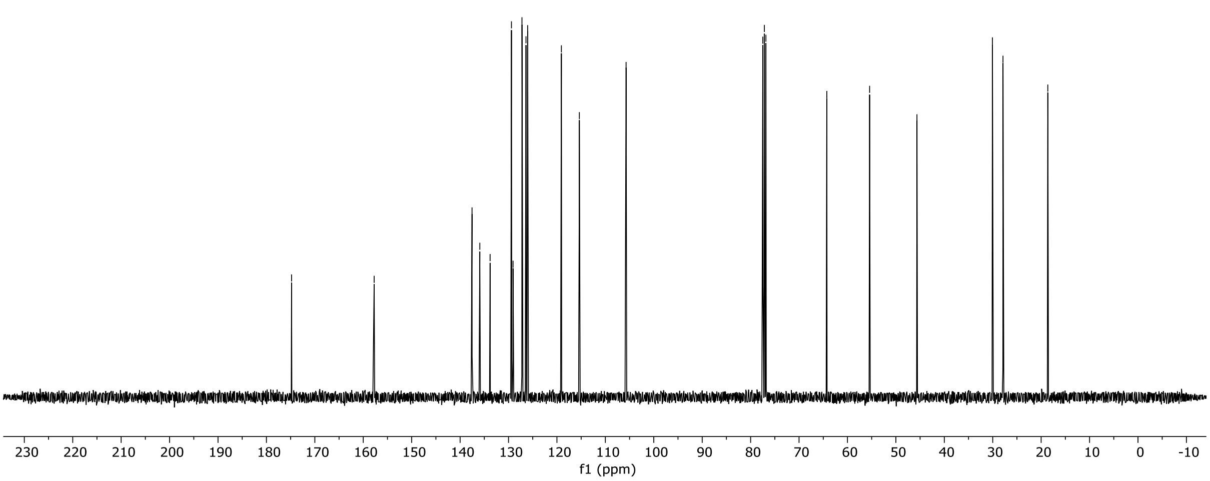

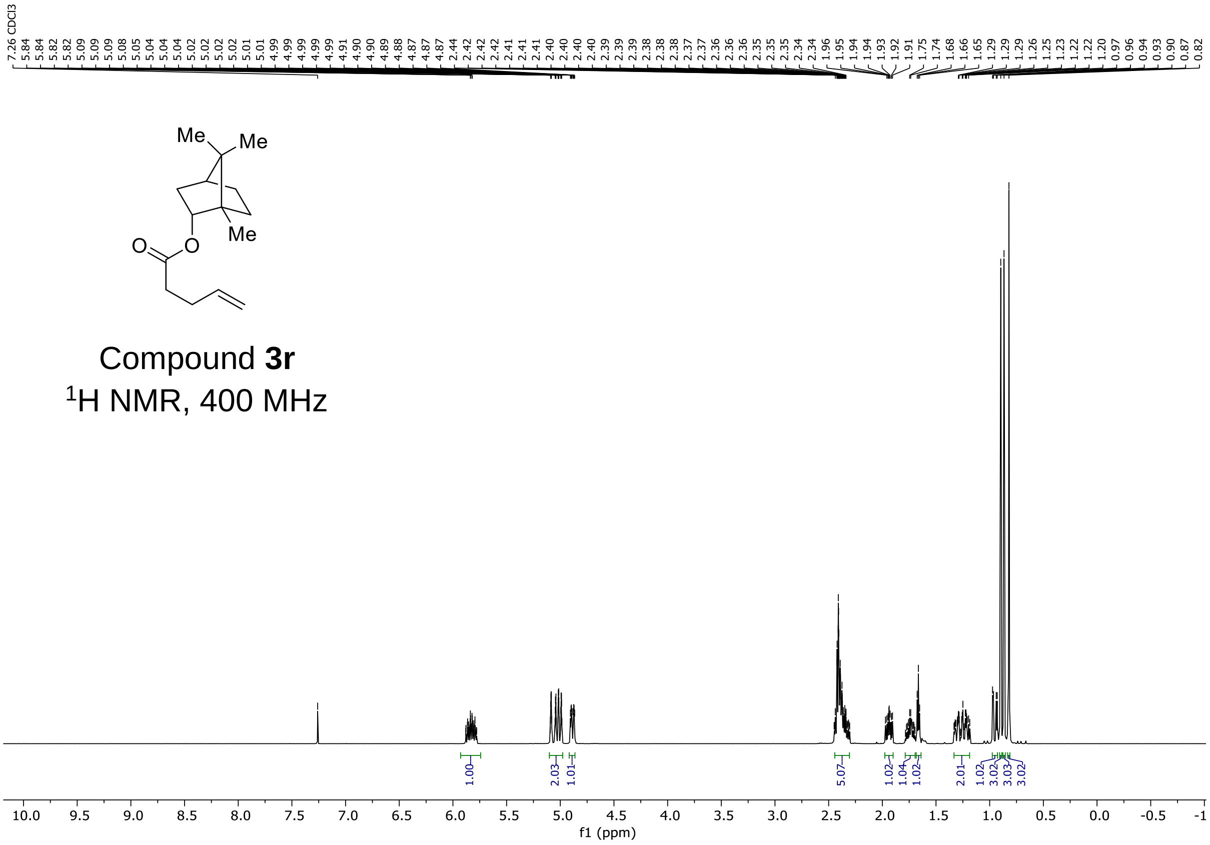

Compound **3r**
^{1}H NMR, 400 MHz

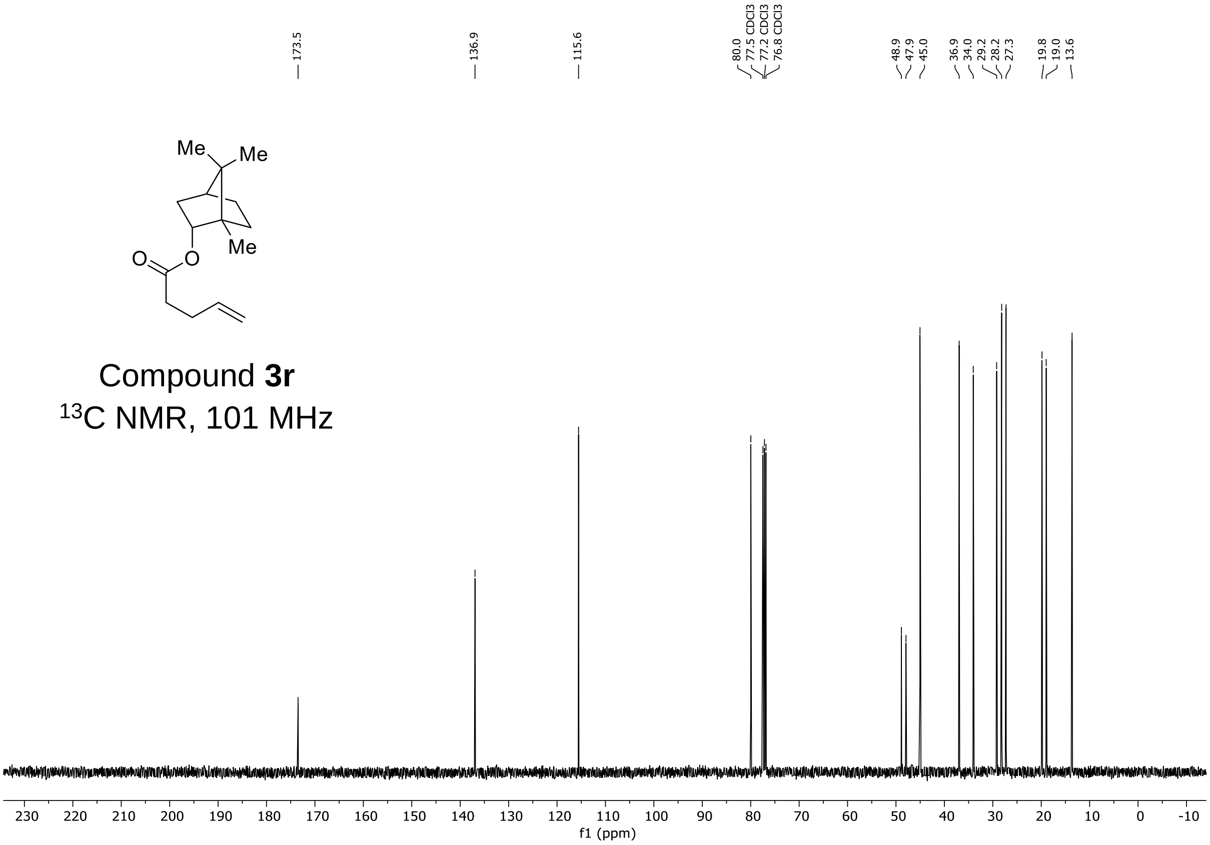

Compound **3r**
^{13}C NMR, 101 MHz

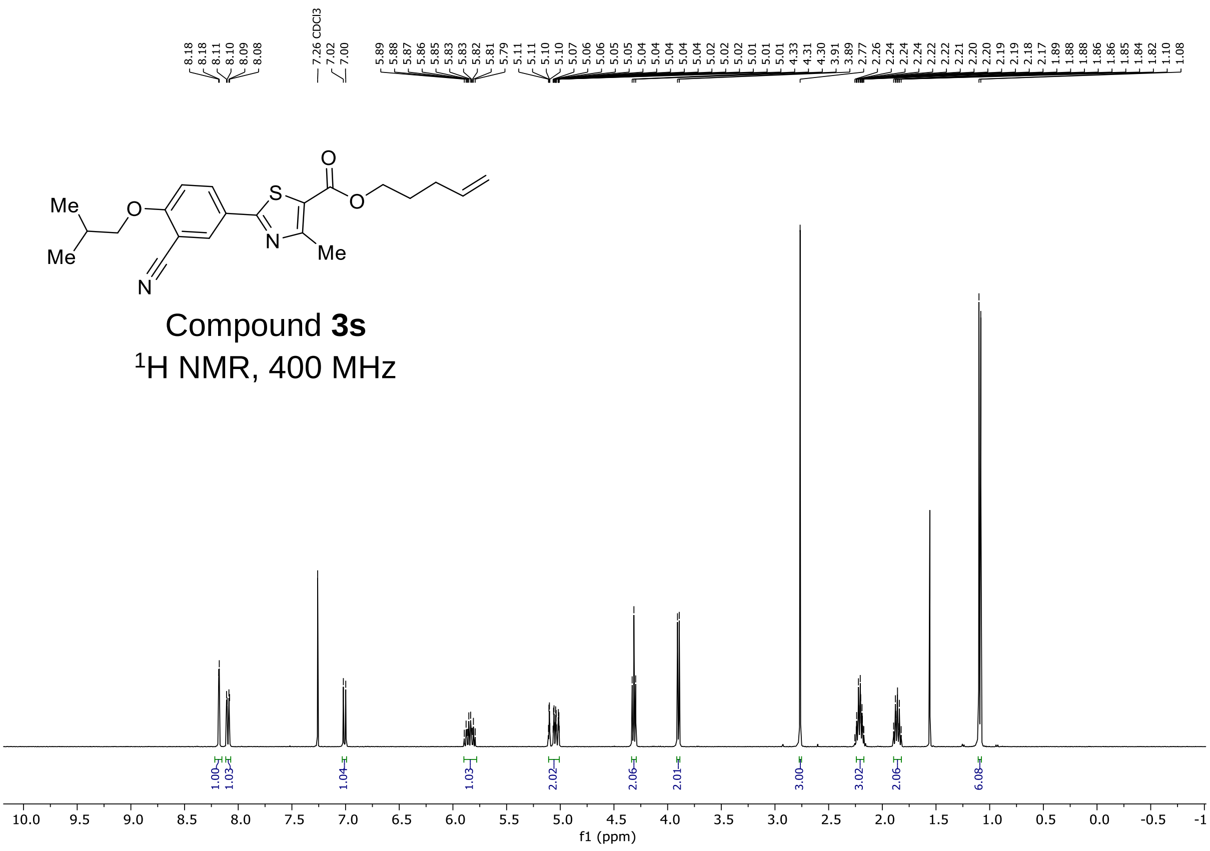
Compound **3s**
^{1}H NMR, 400 MHz
7.26 CDCl3
f1 (ppm)

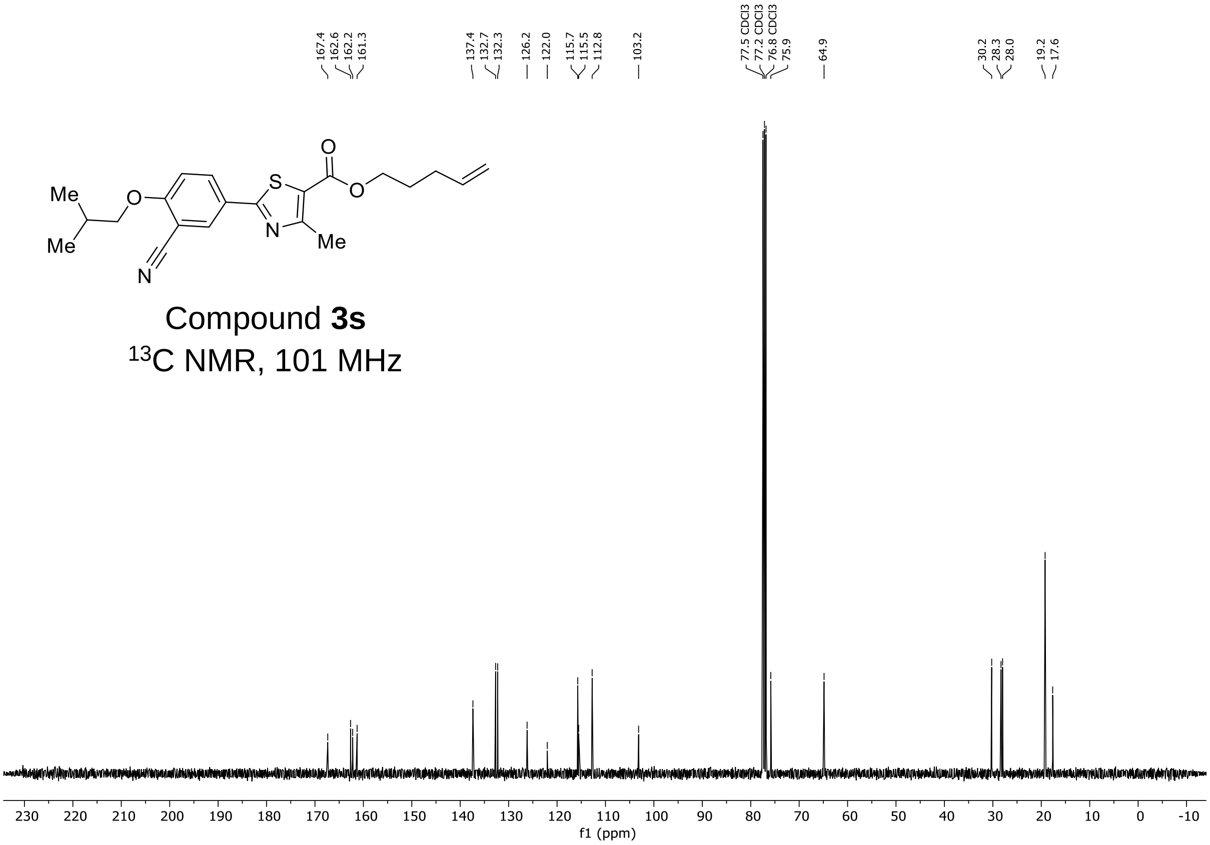
Compound **3s**
^{13}C NMR, 101 MHz
167.4
162.6
162.2
161.3
137.4
132.7
132.3
126.2
122.0
115.7
115.5
112.8
103.2
77.5 CDCl3
77.2 CDCl3
76.8 CDCl3
75.9
64.9
30.2
28.3
28.0
19.2
17.6
f1 (ppm)

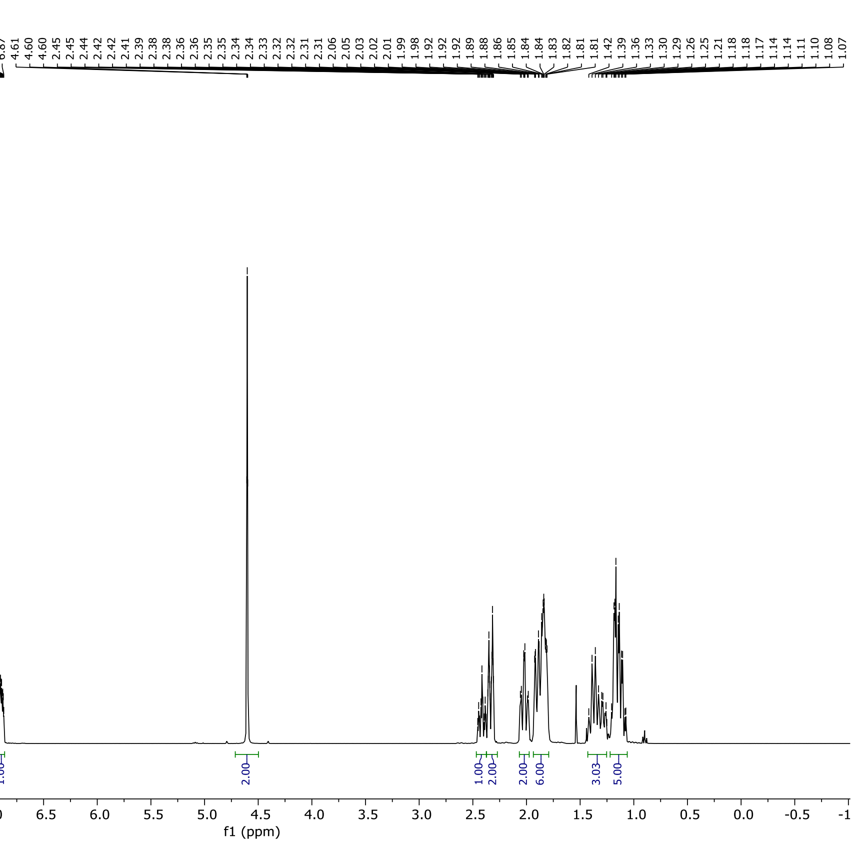

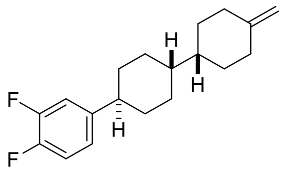

Compound **3t**
^{1}H NMR, 400 MHz

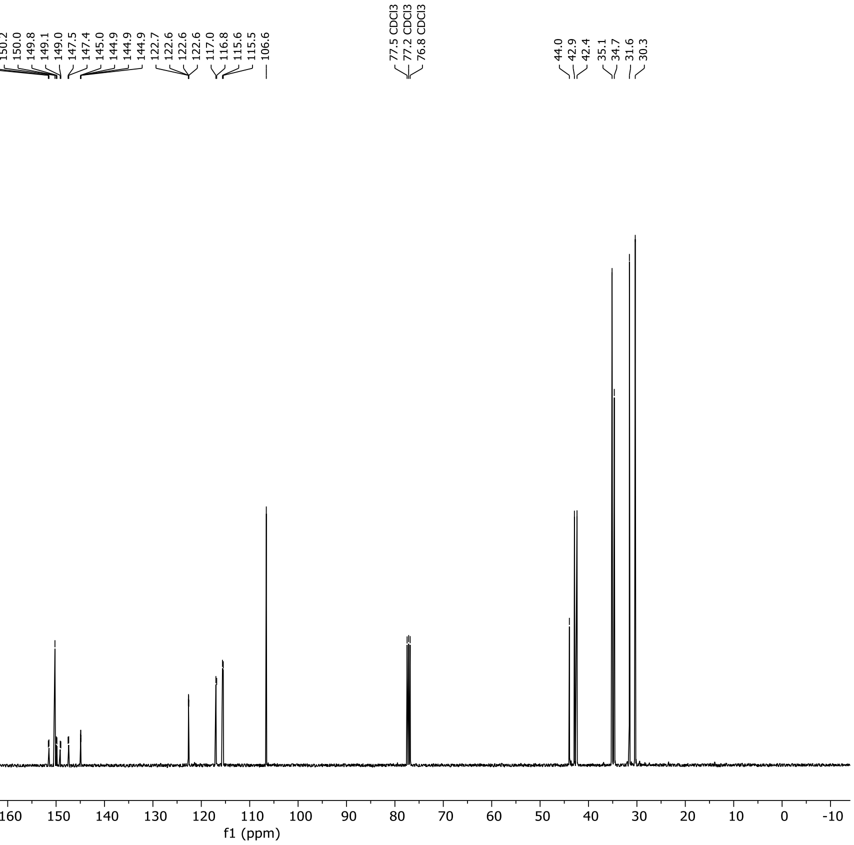

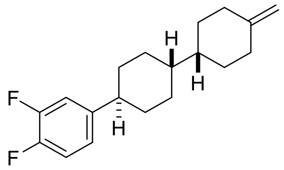

Compound **3t**
^{13}C NMR, 101 MHz

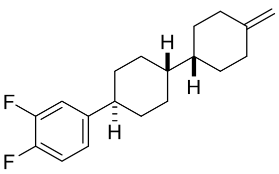

Compound **3t**
^{19}F NMR, 376 MHz

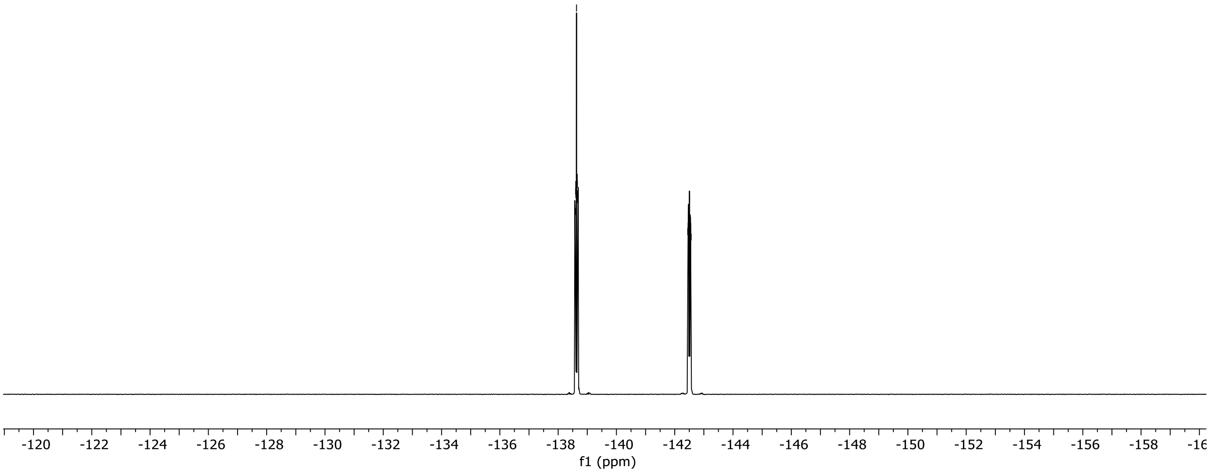

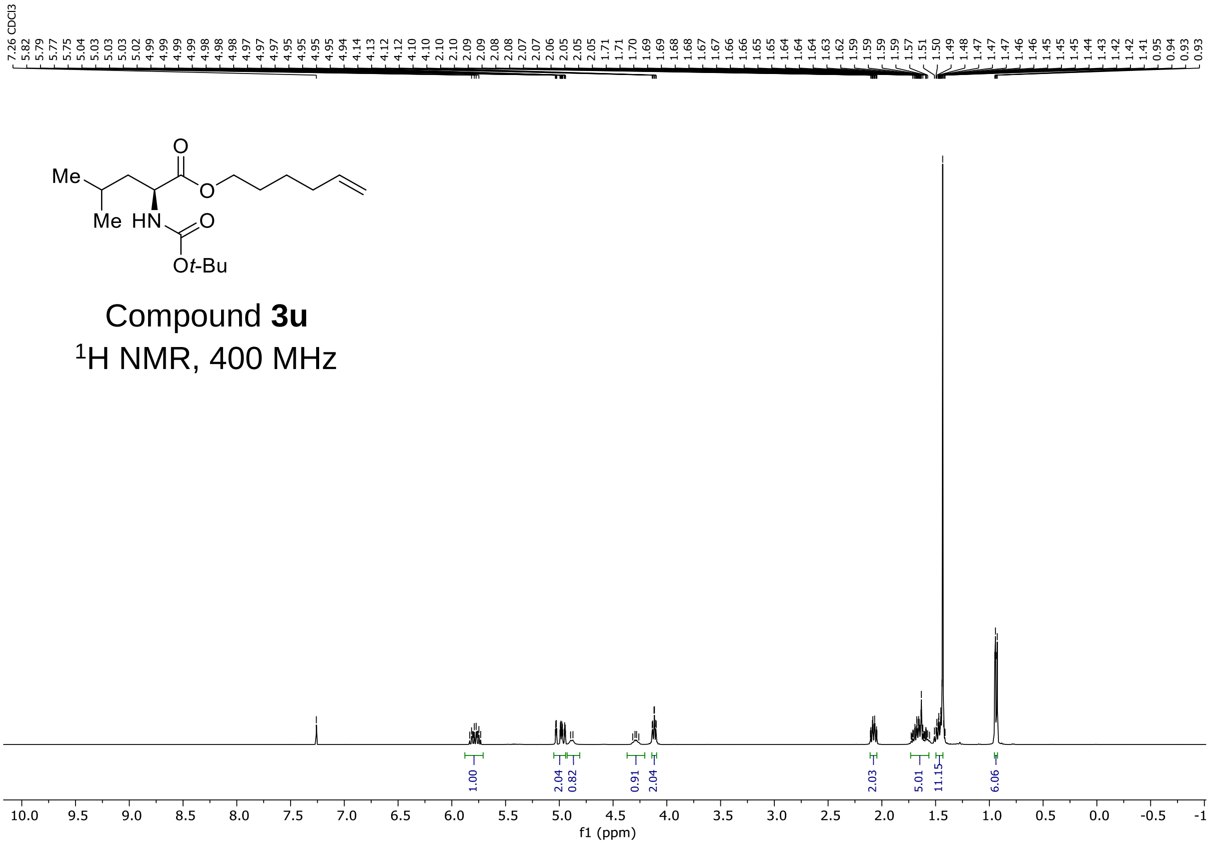
7.26 CDCl3
5.82 5.79 5.77 5.75 5.04 5.03 5.03 5.03 5.02 4.99 4.99 4.99 4.99 4.98 4.98 4.97 4.97 4.97 4.95 4.95 4.95 4.95 4.94 4.14 4.13 4.12 4.12 4.10 4.10 2.10 2.10 2.09 2.09 2.08 2.08 2.07 2.07 2.06 2.05 2.05 2.05 1.71 1.71 1.70 1.69 1.69 1.68 1.68 1.67 1.67 1.66 1.66 1.65 1.65 1.64 1.64 1.64 1.63 1.62 1.59 1.59 1.59 1.59 1.57 1.51 1.50 1.49 1.48 1.47 1.47 1.47 1.46 1.45 1.45 1.45 1.44 1.43 1.42 1.41 0.95 0.94 0.93 0.93
Me
Me
HN
O
O
O
Ot-Bu
Compound 3u
1H NMR, 400 MHz
1.00
2.04
0.82
0.91
2.04
2.03
5.01
11.15
6.06
10.0 9.5 9.0 8.5 8.0 7.5 7.0 6.5 6.0 5.5 5.0 4.5 4.0 3.5 3.0 2.5 2.0 1.5 1.0 0.5 0.0 -0.5 -1
f1 (ppm)

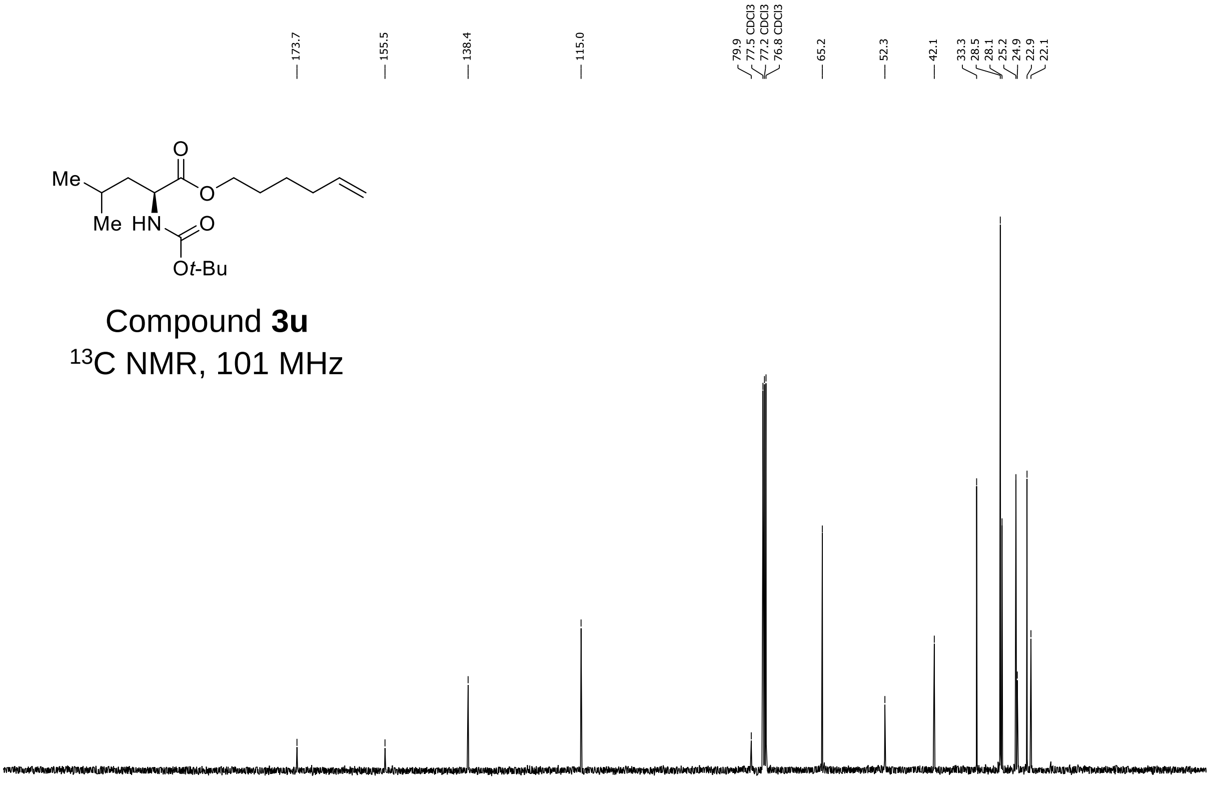
173.7
155.5
138.4
115.0
79.9
77.5 CDCl3
77.2 CDCl3
76.8 CDCl3
65.2
52.3
42.1
33.3
28.5
28.1
25.2
24.9
22.9
22.1
Me
Me
HN
O
O
O
Ot-Bu
Compound 3u
13C NMR, 101 MHz
230 220 210 200 190 180 170 160 150 140 130 120 110 100 90 80 70 60 50 40 30 20 10 0 -10
f1 (ppm)

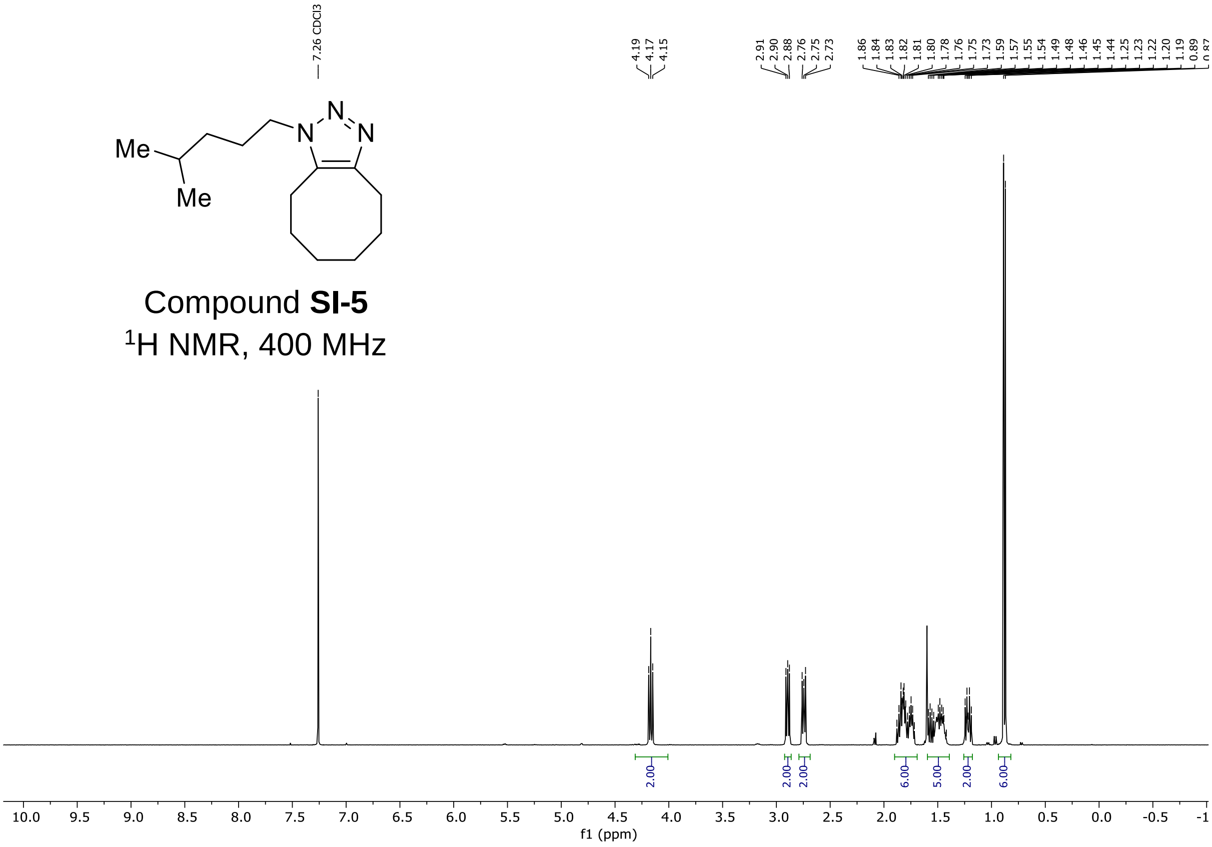

Compound **SI-5**
^{1}H NMR, 400 MHz

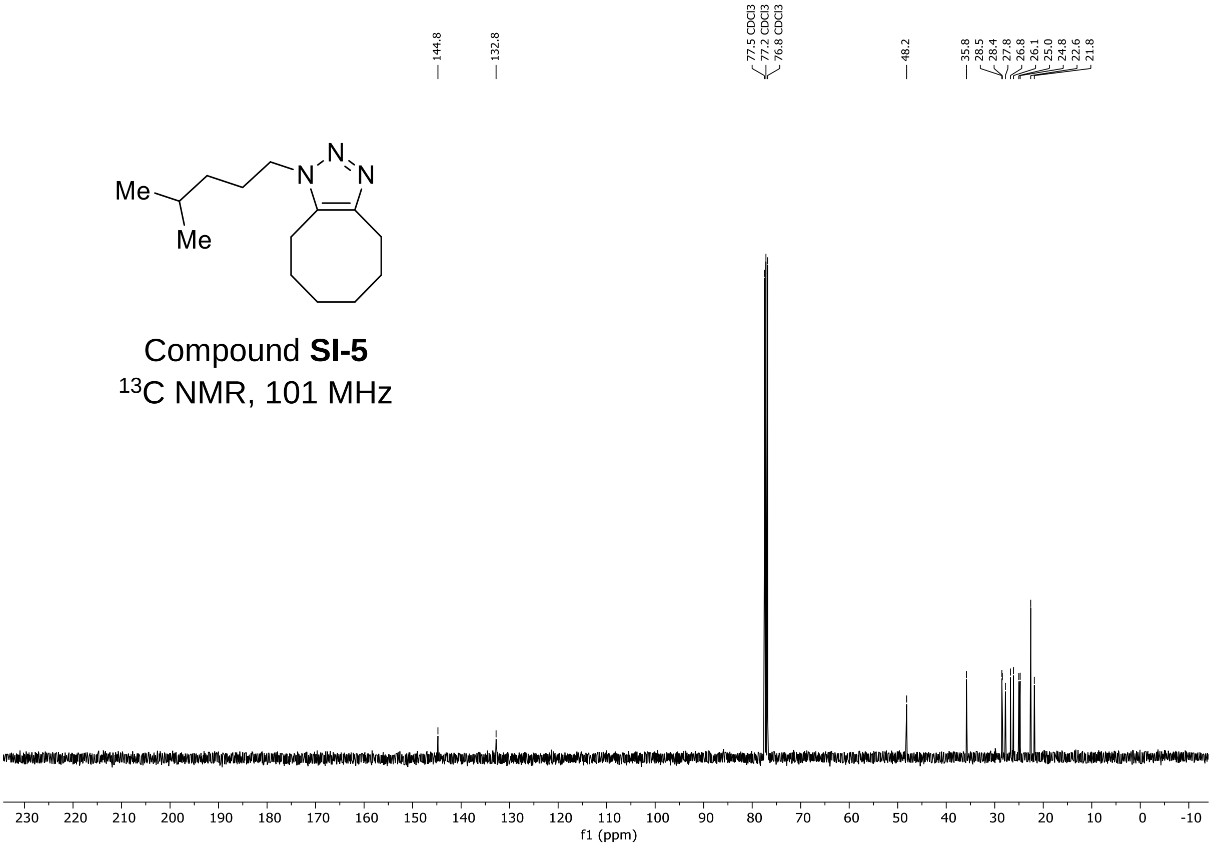

Compound **SI-5**
^{13}C NMR, 101 MHz

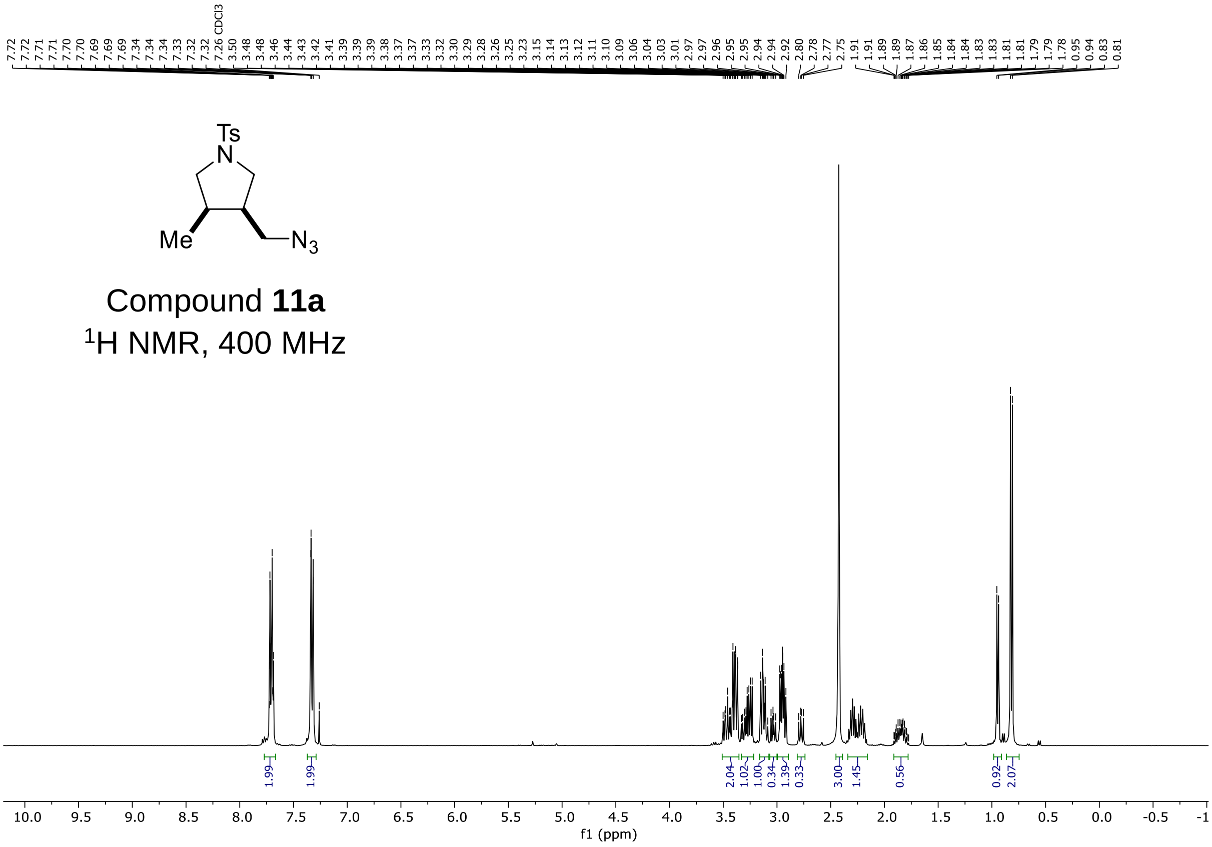
Ts
N
Me
N_3
Compound **11a**
^{1}H NMR, 400 MHz
f1 (ppm)

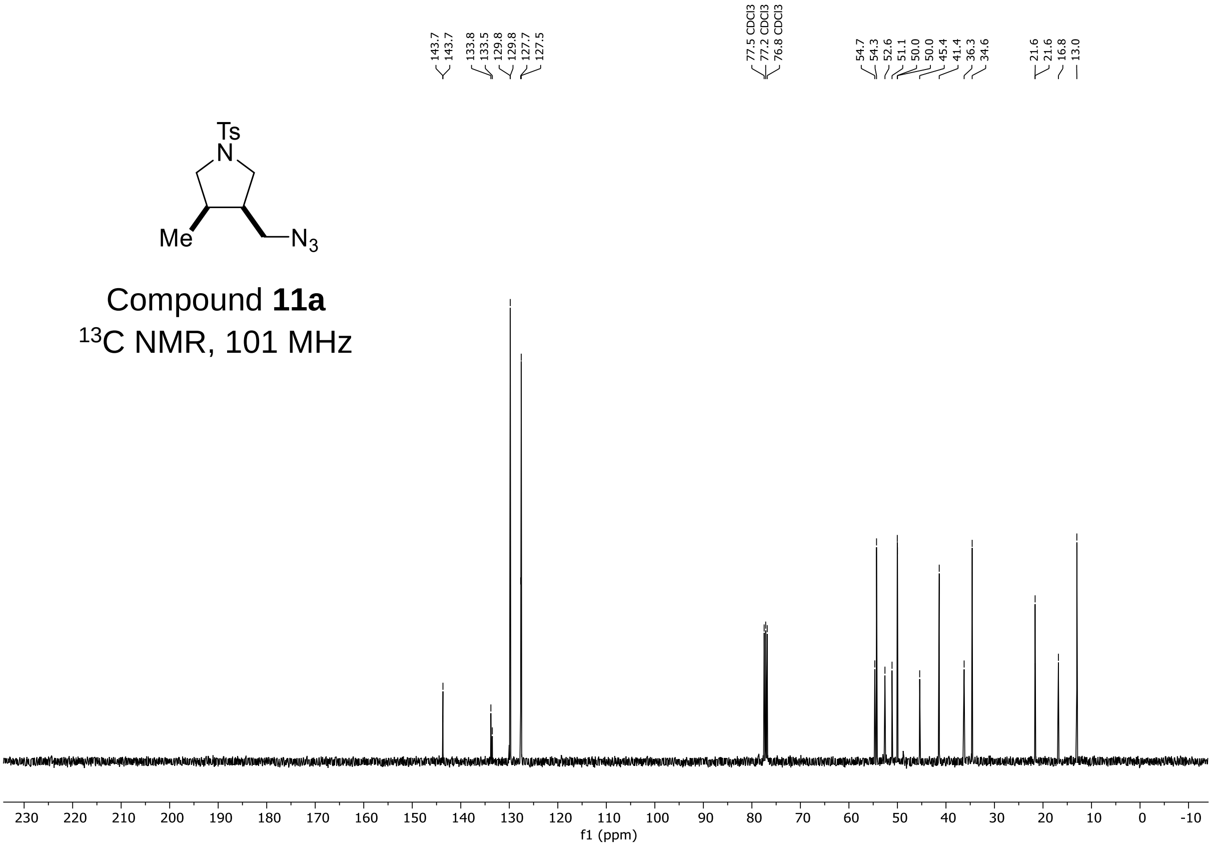
Ts
N
Me
N_3
Compound **11a**
^{13}C NMR, 101 MHz
f1 (ppm)

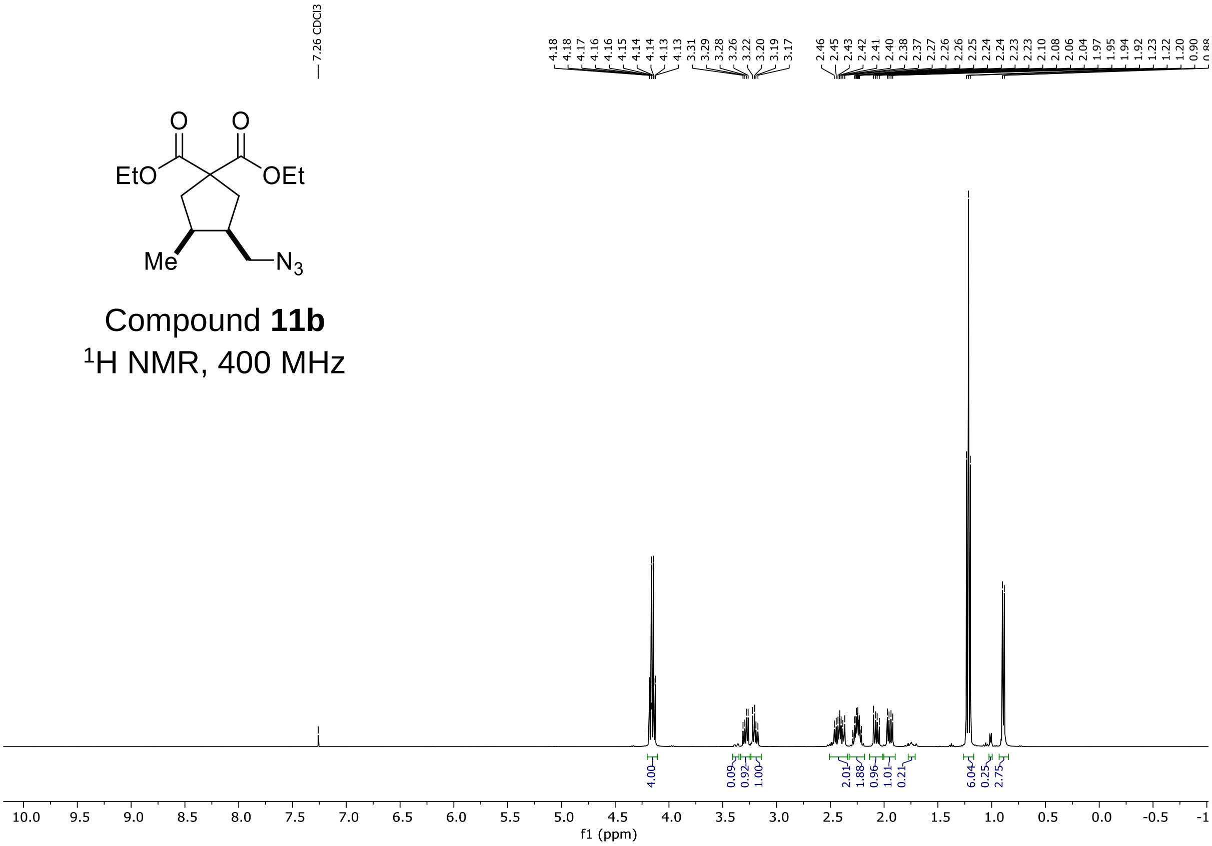

Compound **11b**
^{1}H NMR, 400 MHz

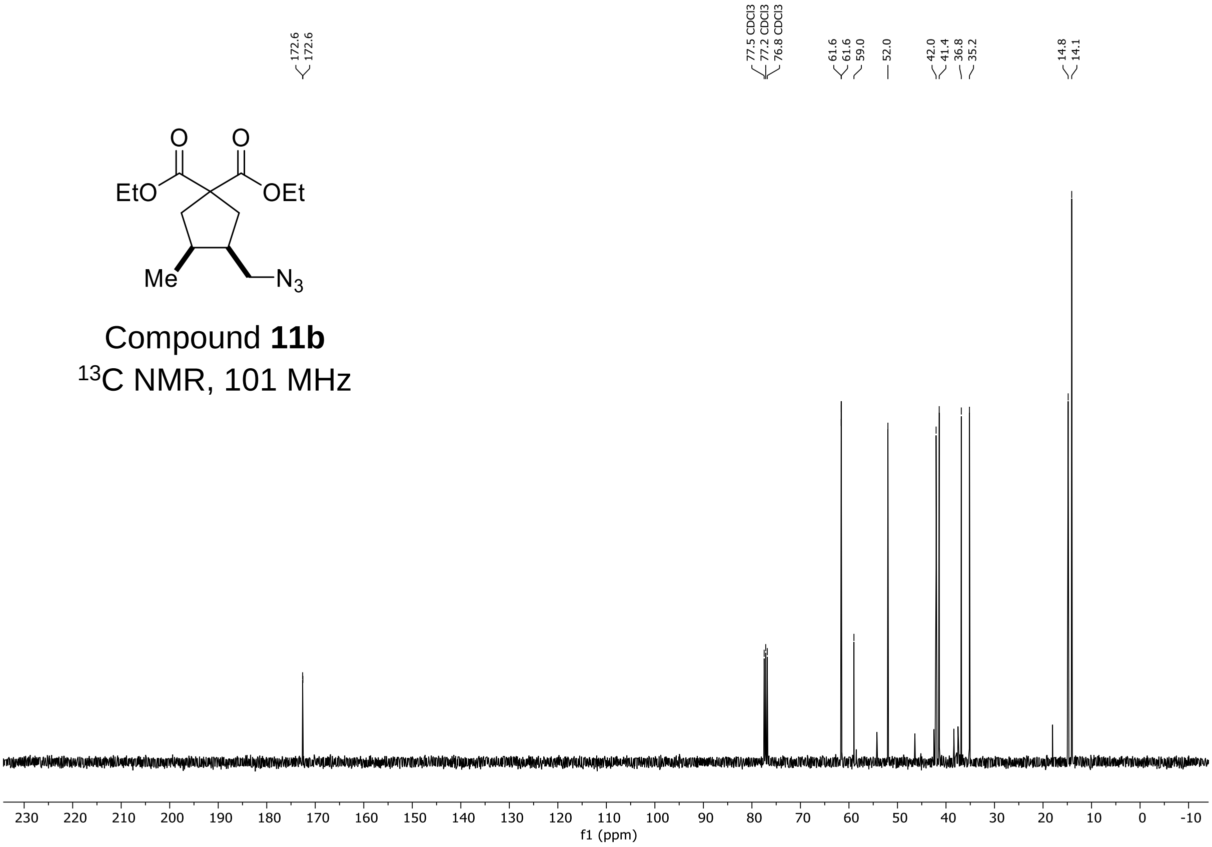

Compound **11b**
^{13}C NMR, 101 MHz

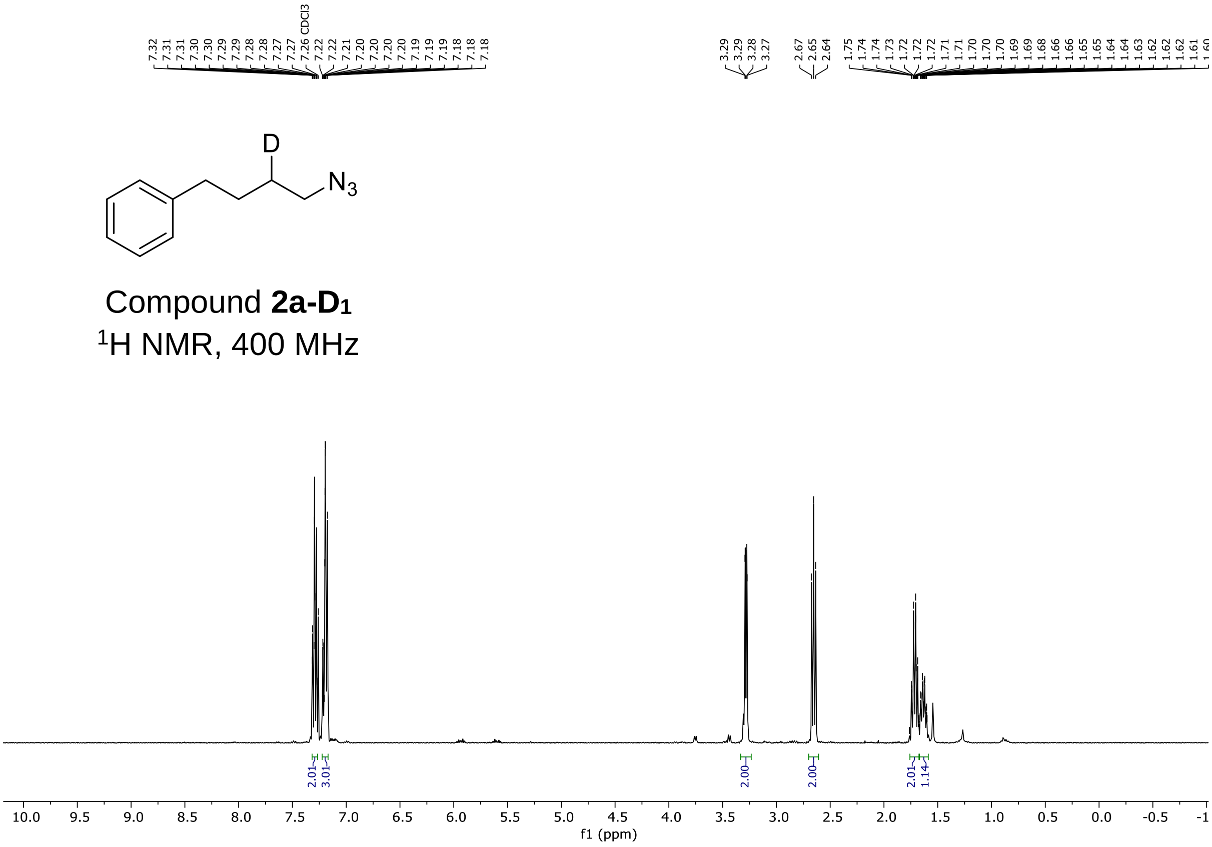

Compound **2a-D1**
^{1}H NMR, 400 MHz

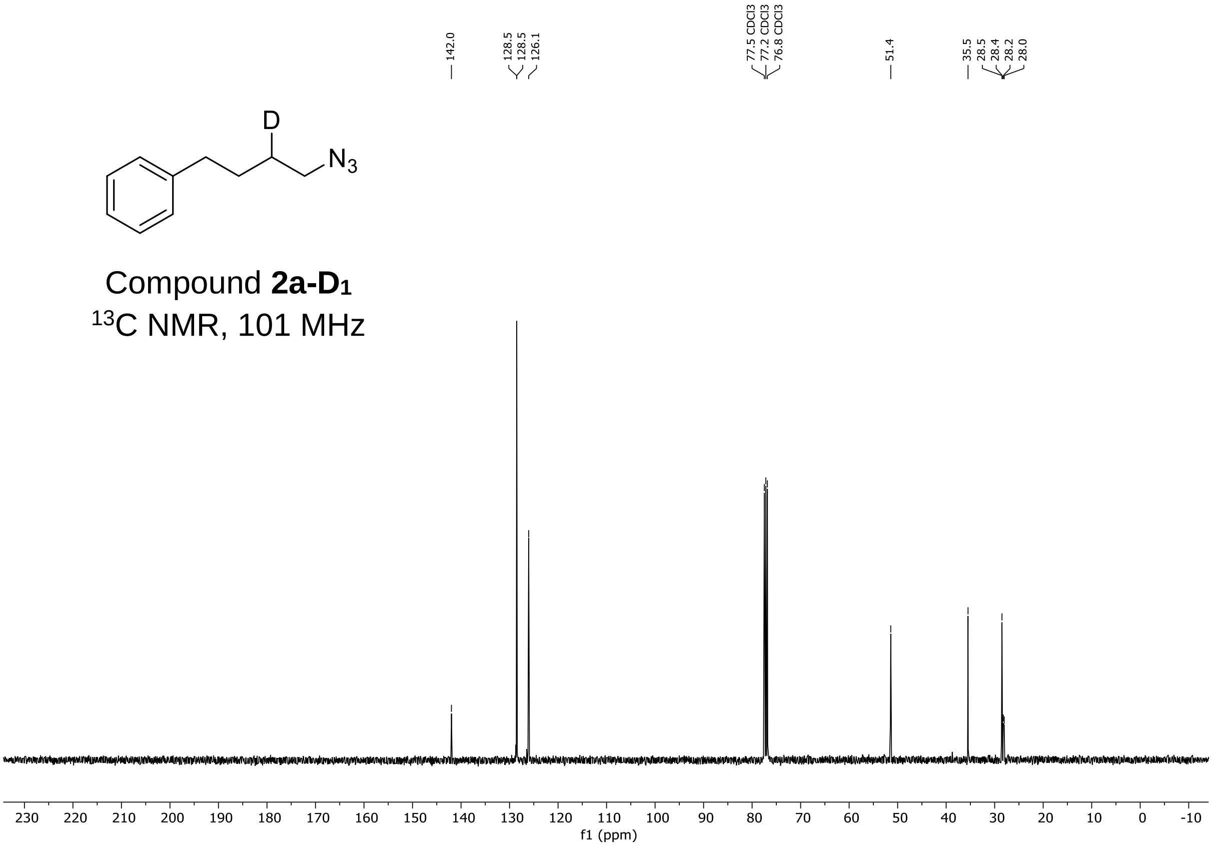

Compound **2a-D1**
^{13}C NMR, 101 MHz

13. X-Ray Crystallographic Data

Compound SI-7:

1-((3aR,4S,5R,7S,7aR)-3a,4,5,6,7,7a-hexahydro-1H-4,7-methanoinden-5-yl)-4-phenyl-1H-1,2,3-triazole

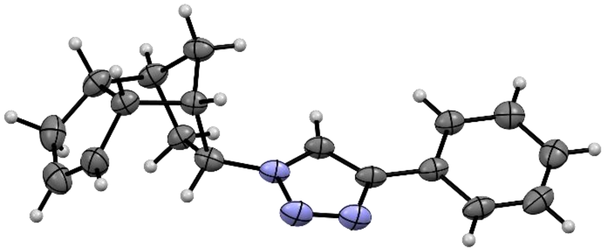

Note: Only one of two orientations is shown. The 3a,4,5,6,7,7a-hexahydro-4,7-methanoindene moiety is disordered in an 80:20 ratio. Thermal ellipsoids are shown at the 50% probability level.

CCDC 2295827

Bond precision:	C–C = 0.0030 A		Wavelength = 1.54184
Cell:	a=13.1800(3)	b=10.1227(2)	c=10.9249(2)
	alpha=90	beta=92.096(2)	gamma=90
Temperature:	100 K		

	Calculated	Reported
Volume	1456.59(5)	1456.59(5)
Space group	I a	I 1 a 1
Hall group	I -2ya	I -2ya
Moiety formula	C18 H19 N3	C18 H19 N3
Sum formula	C18 H19 N3	C18 H19 N3
Mr	277.36	277.36

Dx,g cm–3	1.265	1.265
Z	4	4
Mu (mm–1)	0.590	0.590
F000	592.0	592.0
F000’	593.55	
h,k,lmax	16,12,13	16,12,13
Nref	3170[1588]	2460
Tmin,Tmax	0.856,0.915	0.386,1.000
Tmin’	0.828	

Correction method= # Reported T Limits: Tmin=0.386 Tmax=1.000
AbsCorr = GAUSSIAN

Data completeness= 1.55/0.78 Theta(max)= 79.886

R(reflections)= 0.0364(2387) wR2(reflections)= 0.0946(2460)
S = 1.044 Npar= 281

14. References

(1) Cegielslca, B.; Kacprzak, K. M. Simple and Convenient Protocol for Staining of Organic Azides on TLC Plates by Ninhydrin. A New Application of an Old Reagent. *Chem. Anal. (Warsaw)* **2009**, *54*, 807-812.
(2) (a) Conrow, R. E.; Dean, W. D. Diazidomethane Explosion. *Org. Process Res. Dev.* **2008**, *12*, 1285-1286. (b) Hassner, A.; Stern, M.; Gottlieb, H. E.; Frolow, F. Synthetic methods. 33. Utility of a Polymeric Azide Reagent in the Formation of Di- and Triazidomethane. Their NMR Spectra and the X-ray Structure of Derived Triazoles. *J. Org. Chem.* **1990**, *55*, 2304-2306.
(3) (a) Bräse, S.; Gil, C.; Knepper, K.; Zimmermann, V. Organic Azides: An Exploding Diversity of a Unique Class of Compounds. *Angew. Chem. Int. Ed.* **2005**, *44*, 5188-5240. (b) Sharma, A.; Hartwig, J. F. Metal-catalysed azidation of tertiary C–H bonds suitable for late-stage functionalization. *Nature* **2015**, *517*, 600-604.
(4) Jelier, B. J.; Tripet, P. F.; Pietrasiak, E.; Franzoni, I.; Jeschke, G.; Togni, A. Radical Trifluoromethoxylation of Arenes Triggered by a Visible-Light-Mediated N−O Bond Redox Fragmentation. *Angew. Chem. Int. Ed.* **2018**, *57*, 13784-13789.
(5) Fischer, D. M.; Lindner, H.; Amberg, W. M.; Carreira, E. M. Intermolecular Organophotocatalytic Cyclopropanation of Unactivated Olefins. *J. Am. Chem. Soc.* **2023**, *145*, 774-780.
(6) Xie, Y.; Sun, P.-W.; Li, Y.; Wang, S.; Ye, M.; Li, Z. Ligand-Promoted Iron(III)-Catalyzed Hydrofluorination of Alkenes. *Angew. Chem. Int. Ed.* **2019**, *58*, 7097-7101
(7) Zhao, Q.; Lu, L.; Shen, Q. Direct Monofluoromethylthiolation with S-(Fluoromethyl) Benzenesulfonothioate. *Angew. Chem. Int. Ed.* **2017**, *56*, 11575-11578.
(8) Zhang, C.; Li, Z.; Zhu, L.; Yu, L.; Wang, Z.; Li, C. Silver-Catalyzed Radical Phosphonofluorination of Unactivated Alkenes. *J. Am. Chem. Soc.* **2013**, *135*, 14082-14085.
(9) Schlummer, B.; Hartwig, J. F. Brønsted Acid-Catalyzed Intramolecular Hydroamination of Protected Alkenylamines. Synthesis of Pyrrolidines and Piperidines. *Org. Lett.* **2002**, *4*, 1471-1474.
(10) Rajabi, J.; Lorion, M. M.; Ly, V. L.; Liron, F.; Oble, J.; Prestat, G.; Poli, G. Dormant versus Evolving Aminopalladated Intermediates: Toward a Unified Mechanistic Scenario in Pd^{II}-Catalyzed Aminations. *Chem. Eur. J* **2014**, *20*, 1539-1546.

(11) Stang, E. M.; White, M. C. Molecular Complexity via C–H Activation: A Dehydrogenative Diels–Alder Reaction. *J. Am. Chem. Soc.* **2011**, *133*, 14892-14895.
(12) Chevella, D.; Macharla, A. K.; Banothu, R.; Gajula, K. S.; Amrutham, V.; Boosa, M.; Nama, N. Synthesis of non-symmetrical alkyl carbonates from alcohols and DMC over the nanocrystalline ZSM-5 zeolite. *Green Chem.* **2019**, *21*, 2938-2945.
(13) Tzur, E.; Ivry, E.; Diesendruck, C. E.; Vidavsky, Y.; Goldberg, I.; Lemcoff, N. G. Stability and activity of cis-dichloro ruthenium olefin metathesis precatalysts bearing chelating sulfur alkylidenes. *J. Organomet. Chem.* **2014**, *769*, 24-28.
(14) Zhou, J.; Reidy, M.; O'Reilly, C.; Jarikote, D. V.; Negi, A.; Samali, A.; Szegezdi, E.; Murphy, P. V. Decorated Macrocycles via Ring-Closing Double-Reductive Amination. Identification of an Apoptosis Inducer of Leukemic Cells That at Least Partially Antagonizes a 5-HT2 Receptor. *Org. Lett.* **2015**, *17*, 1672-1675.
(15) Ma, G.; Wan, W.; Li, J.; Hu, Q.; Jiang, H.; Zhu, S.; Wang, J.; Hao, J. An efficient regioselective hydrodifluoromethylation of unactivated alkenes with $TMSCF_2CO_2Et$ at ambient temperature. *Chem. Commun.* **2014**, *50*, 9749-9752.
(16) Fraunhoffer, K. J.; Bachovchin, D. A.; White, M. C. Hydrocarbon Oxidation vs C−C Bond-Forming Approaches for Efficient Syntheses of Oxygenated Molecules. *Org. Lett.* **2005**, *7*, 223-226.
(17) Jeschke, P.; Mueller, M.; Escher, I.; Malsam, O.; Haack, K.-J.; Braun, R.; Arnold, C. Substituted oxyarenes. Patent WO2004099197A3, 2011.
(18) Siu, J. C.; Parry, J. B.; Lin, S. Aminoxyl-Catalyzed Electrochemical Diazidation of Alkenes Mediated by a Metastable Charge-Transfer Complex. *J. Am. Chem. Soc.* **2019**, *141*, 2825-2831.
(19) Soulard, V.; Villa, G.; Vollmar, D. P.; Renaud, P. Radical Deuteration with D_2O: Catalysis and Mechanistic Insights. *J. Am. Chem. Soc.* **2018**, *140*, 155-158.
(20) Shyam, R.; Charbonnel, N.; Job, A.; Blavignac, C.; Forestier, C.; Taillefumier, C.; Faure, S. 1,2,3-Triazolium-Based Cationic Amphipathic Peptoid Oligomers Mimicking Antimicrobial Helical Peptides. *ChemMedChem* **2018**, *13*, 1513-1516.
(21) Park, H.; Hong, Y.-L.; Kim, Y. B.; Choi, T.-L. Synthesis of Small and Large Fused Bicyclic Compounds by Tandem Dienyne Ring-Closing Metathesis. *Org. Lett.* **2010**, *12*, 3442-3445.
(22) Frontier, A. J.; Danishefsky, S. J.; Koppel, G. A.; Meng, D. A useful α, α′ -annulation reaction of enamines. *Tetrahedron* **1998**, *54*, 12721-12736.

(23) Law, J. A.; Bartfield, N. M.; Frederich, J. H. Site-Specific Alkene Hydromethylation via Protonolysis of Titanacyclobutanes. *Angew. Chem. Int. Ed.* **2021**, *60*, 14360-14364.

(24) Young, P. C.; Hadfield, M. S.; Arrowsmith, L.; Macleod, K. M.; Mudd, R. J.; Jordan-Hore, J. A.; Lee, A.-L. Divergent Outcomes of Gold(I)-Catalyzed Indole Additions to 3,3-Disubstituted Cyclopropenes. *Org. Lett.* **2012**, *14*, 898-901.

(25) Zhu, Y.; Li, X.; Wang, X.; Huang, X.; Shen, T.; Zhang, Y.; Sun, X.; Zou, M.; Song, S.; Jiao, N. Silver-Catalyzed Decarboxylative Azidation of Aliphatic Carboxylic Acids. *Org. Lett.* **2015**, *17*, 4702-4705.

(26) Li, H.; Shen, S.-J.; Zhu, C.-L.; Xu, H. Direct Intermolecular Anti-Markovnikov Hydroazidation of Unactivated Olefins. *J. Am. Chem. Soc.* **2019**, *141*, 9415-9421.

(27) Youn, S. W.; Pastine, S. J.; Sames, D. Ru(III)-Catalyzed Cyclization of Arene-Alkene Substrates via Intramolecular Electrophilic Hydroarylation. *Org. Lett.* **2004**, *6*, 581-584.

(28) Kobayashi, Y.; Yoshida, S.; Nakayama, Y. Total Synthesis of Korormicin. *Eur. J. Org. Chem.* **2001**, *2001*, 1873-1881.

(29) Hassner, A.; Fibiger, R.; Andisik, D. Synthetic methods. 19. Lewis Acid Catalyzed Conversion of Alkenes and Alcohols to Azides. *J. Org. Chem.* **1984**, *49*, 4237-4244.

(30) Yasukawa, N.; Yokoyama, H.; Masuda, M.; Monguchi, Y.; Sajiki, H.; Sawama, Y. Highly-functionalized arene synthesis based on palladium on carbon-catalyzed aqueous dehydrogenation of cyclohexadienes and cyclohexenes. *Green Chem.* **2018**, *20*, 1213-1217.

(31) Pedersen, H.; Sinning, S.; Bülow, A.; Wiborg, O.; Falborg, L.; Bols, M. Combinatorial synthesis of benztropine libraries and their evaluation as monoamine transporter inhibitors. *Organic & Biomolecular Chemistry* **2004**, *2*, 2861-2869.

(32) Chen, J.; Li, J.; Plutschack, M. B.; Berger, F.; Ritter, T. Regio- and Stereoselective Thianthrenation of Olefins To Access Versatile Alkenyl Electrophiles. *Angew. Chem. Int. Ed.* **2020**, *59*, 5616-5620.
